# Supplementary material for: Trichalcogenasupersumanenes and its concave-convex supramolecular assembly with fullerenes
Source: Nat Commun. 2023 Jun 10;14:3446. doi: 10.1038/s41467-023-39086-0 (PMC10257710; doi:10.1038/s41467-023-39086-0)
Supplement: Supplementary file 1 — Supplementary Information [file 41467_2023_39086_MOESM1_ESM.pdf]

Supplementary Information

Trichalcogenasupersumanenes and its concave-convex supramolecular assembly with fullerenes

Yixun Sun†, Xin wang†, Bo Yang†, Muhua Chen, Ziyi Guo, Yiting Wang, Ji Li, Mingyu Xu, Yunjie Zhang, Huaming Sun, Jingshuang Dang, Juan Fan, Jing Li*, and Junfa Wei*

School of Chemistry and Chemical Engineering, Shaanxi Normal University, Xi'an 710119, P. R. China

† These authors contributed equally.
* E-mail: li_jing@snnu.edu.cn; weijf@snnu.edu.cn

Table of Content

1. Experimental Section 3
1.1 General 3
1.2 Synthetic procedures and characterization data 4
1.2.1 Synthesis of 2-chloro-1-(2,7-dichloro-9H-fluoren-4-yl)ethan-1-one[4] 4
1.2.2 Synthesis of 1-(2,7-dichloro-9*H*-fluoren-4-yl)ethan-1-one: 4
1.2.3 Synthesis of 1-(9,9-dibutyl-2,7-dichloro-9*H*-fluoren-4-yl)ethan-1-one (2): 5
1.2.4 Synthesis of 1,3,5-tris(9,9-dibutyl-2,7-dichloro-9*H*-fluoren-4-yl)benzene (TFB, 3): 5
1.2.5 Synthesis of hexachloro-trifluorenocoronene (TFC, 4): 6
1.2.6 Synthesis of trithiasupersumanene (1a): 7
1.2.7 Synthesis of triselenosupersumanene (1b): 7
1.2.8 Synthesis of 1-(9,9-dimethyl-2,7-dichloro-9*H*-fluoren-4-yl)ethan-1-one (2-Me): 8
1.2.9 Synthesis of trithiasupersumanene (1a-Me): 9
2. Crystallographic details 10
2.1 X-ray Crystallography for (1a) 10
2.2 X-ray Crystallography for (1b) 15
3. Photophysical Properties 21
4. Electrochemical Properties 23
5. Thermogravimetric Analysis (TGA) 25
6. Association with Fullerenes 26
6.1 X-ray Crystallography for (1a-Me@C_{60}) 26
6.2 X-ray Crystallography for (1a-Me@C_{70}) 28
6.3 Job plot analyses 32
6.4 Determination of binding constant 33
7. DFT calculations 36
7.1 Optimized Structures and Bowl to Bowl Inversion Energy 36
7.2 Aromaticity Calculations 41
7.3 UV-Vis Absorption Spectra Calculation 43
7.4 Electrostatic Potential Surfaces and Molecular Dipole Moments Calculation 49
7.5 Binding energies of 1a-Me@C_{60} and 1a-Me@C_{70} 50
8. Attached NMR spectra and HRMS spectra 52
9. Coordinates of geometry optimized structures 68
10. References 87

1. Experimental Section

1.1 General

All reagents and starting materials were obtained from commercial suppliers and used without further purification unless otherwise noted. All air or moisture sensitive reactions were carried out under an argon atmosphere by standard Schlenk techniques. Anhydrous toluene and Et_2O were distilled from sodium-benzophenone immediately prior to use. Anhydrous dichloromethane (DCM) was distilled from CaH_2. The reactions were monitored using analytical thin layer chromatography (TLC, GF-254). Tributyltinselenide, $(Bu_3Sn)_2Se$, was prepared according to literature procedure in 50 mmol scale.[1] Flash chromatography was performed using silica gel (200-300 mesh) with freshly distilled solvents.

The ^1H NMR and ^{13}C NMR spectra were recorded on a JEOL 400 MHz or a Bruker Avance 600 MHz spectrophotometer using $CDCl_3$, Toluene-d_8, or 1,2-dichlorobenzene-d_4 as a solvent. Chemical shifts (δ) are reported in parts per million (ppm) using TMS as an internal standard. The terms m, s, d, t and q represent multiplet, singlet, doublet, triplet and quartet, respectively. The term (br.) is used when the peak is broad, and the correct multiplicity cannot be surely assigned. Coupling constants (*J*) are given in Hertz (Hz). The high-resolution mass spectra were recorded on a Bruker Avance spectrometer (maXis) (Operation Mode: APCI Positive Ion Mode, Analyzer Type: TOF). The crystal structure was recorded on Bruker D8 Venture X-ray single crystal diffraction spectrometer. Microwave reactions were performed with Xianghu XH-100A Microwave Irradiation Equipment.

UV-vis absorption spectra were recorded on a Hitachi U-3900/3900H spectrophotometer. Fluorescence spectra were performed on a fluorolog-3 fluorescence spectrophotometer (Horiba JY, USA). The fluorescence lifetime was measured using a FLSP920 fluorescence spectrophotometer. Absolute fluorescence quantum yields were measured on a HAMAMATSU Absolute PL Quantum Yield Measurement System C9920-02G at room temperature. Thermogravimetric analysis (TGA) measurements were performed on a STA 409 PC instrument under a dry nitrogen flow, heating from room temperature to 800 ºC, at a heating rate of 10 ºC/min.

Cyclic voltammetry (CV) and differential pulse voltammetry (DPV) were performed on a CHI 660B electrochemical analyzer at room temperature in inert atmosphere with a three-electrode configuration in CH_2Cl_2 solution (purchased from Sigma-Aldrich) containing the substrate (typically 2×10^{-3} M) and 0.1 M tetrabutylammonium hexafluorophosphate (n-Bu_4NPF_6) as the supporting electrolyte. A platinum disc, a platinum plate, and a silver wire electrode were served as the working electrode, the counter electrode and the quasi-reference electrode (QRE), respectively. All potentials were calibrated versus an aqueous SCE by the addition of ferrocene as an internal standard taking $E_{1/2}(Fc/Fc^+) = 0.424$ V *vs*. SCE.[2] The scan rate was 0.1 V/s. The HOMO and LUMO energy values were estimated from the onset potentials of the first oxidation and reduction event, respectively. The HOMO and LUMO energy levels were calculated according to the following equations:

$$E_{HOMO}\ (eV) = -\ [E_{onset}^{ox}\ (vs\ SCE) - E_{1/2}(Fc/Fc^+) + 4.8] = -\ [E_{onset}^{ox}\ (vs\ SCE) + 4.38]$$

$$E_{LUMO}\ (eV) = -\ [E_{onset}^{re}\ (vs\ SCE) - E_{1/2}(Fc/Fc^+) + 4.8] = -\ [E_{onset}^{re}\ (vs\ SCE) + 4.38]$$

Where $E_{1/2}(Fc/Fc^+)$ is the half-wave potential of the Fc/Fc^+ couple against the SCE electrode. Theoretical calculations were carried out using the Gaussian 09, Revision D.01program.[3]

1.2 Synthetic procedures and characterization data

1.2.1 Synthesis of 2-chloro-1-(2,7-dichloro-9H-fluoren-4-yl)ethan-1-one[4]

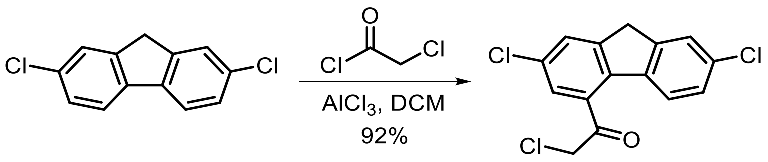

A mixture of chloroacetyl chloride (6.78 g, 60 mmol), $AlCl_3$ (9.33 g, 70 mmol), and dichloromethane (100 mL) was cooled to -5 °C. A solution of 2,7-dichloro-9*H*-fluorene (11.76 g, 50 mmol) dissolved in dichloromethane (50 mL) was added. After 3-10 h, the deep-red reaction mixture was transferred to 15% aqueous hydrochloric acid (200 mL). After phase separation, the organic phase was washed twice with water. Dichloromethane was distilled off, while ethanol was added at the same rate. The crystallized product was isolated by filtration to give pure 2-chloro-1-(2,7-dichloro-9*H*-fluoren-4-yl)ethan-1-one (14.35 g, 92%); M.p.: 121-122 °C.

2-Chloro-1-(2,7-dichloro-9*H*-fluoren-4-yl)ethan-1-one

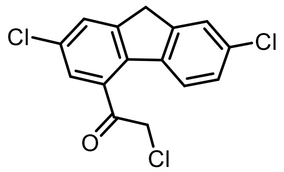

^{1}**H NMR** (600 MHz, $CDCl_3$): δ 7.79 (d, J = 8.5 Hz, 1H), 7.59-7.56 (m, 1H), 7.44 (d, J = 1.9 Hz, 1H), 7.43 (d, J = 1.5 Hz, 1H), 7.26 (dd, J = 8.6, 2.0 Hz, 1H), 4.67 (s, 2H), 3.80 (s, 2H); ^{13}**C NMR** (151 MHz, $CDCl_3$) δ 194.4, 147.1, 145.5, 137.6, 137.2, 134.1, 132.5, 132.3, 128.7, 127.5, 126.5, 125.1, 124.9, 48.0, 36.6; **HRMS** (APCI-TOF, positive mode, methanol/chloroform) *m/z* calcd for $C_{15}H_{10}Cl_3O$ $(M+H)^+$: 310.9792, found: 310.9787.

1.2.2 Synthesis of 1-(2,7-dichloro-9*H*-fluoren-4-yl)ethan-1-one:

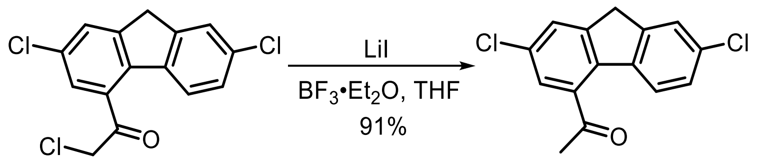

To a solution of 2-chloro-1-(2,7-dichloro-9*H*-fluoren-4-yl)ethan-1-one (7.80 g, 25 mmol), LiI (11.10 g, 83 mmol) in THF (150 mL) was added BF_3•Et_2O (3.8 mL, 30 mmol) slowly under argon atmosphere and the mixture was stirred for 8 h at room temperature. The reaction was quenched with saturated $NaHCO_3$ solution, and extracted with dichloromethane, and dried with Na_2SO_4. The solvent was removed under vacuum, and the residue was purified by column chromatography (silica gel, PE/DCM = 6:1) to afford the pure product 1-(2,7-dichloro-9*H*-fluoren-4-yl)ethan-1-one (6.30 g, 91%); M.p.: 122-123 °C.

1-(2,7-Dichloro-9*H*-fluoren-4-yl)ethan-1-one

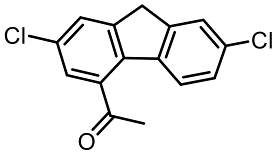

^{1}**H NMR** (600 MHz, $CDCl_3$): δ 7.98 (d, J = 8.5 Hz, 1H), 7.62-7.58 (m, 1H), 7.56 (d, J = 1.8 Hz, 1H), 7.49 (s, 1H), 7.30 (dd, J = 8.5, 2.0 Hz, 1H), 3.87 (s, 2H), 2.70 (s, 3H); ^{13}**C NMR** (151 MHz, $CDCl_3$): δ 201.2, 146.8, 145.5, 137.8, 136.7, 136.1, 133.7, 132.1, 128.0, 127.3, 127.2, 125.4, 124.9, 36.6, 29.9; **HRMS** (APCI-TOF, positive mode, methanol/chloroform) *m/z* calcd for $C_{15}H_{11}Cl_2O$ $(M+H)^+$: 277.0181, found: 277.0178.

1.2.3 Synthesis of 1-(9,9-dibutyl-2,7-dichloro-9*H*-fluoren-4-yl)ethan-1-one (2):

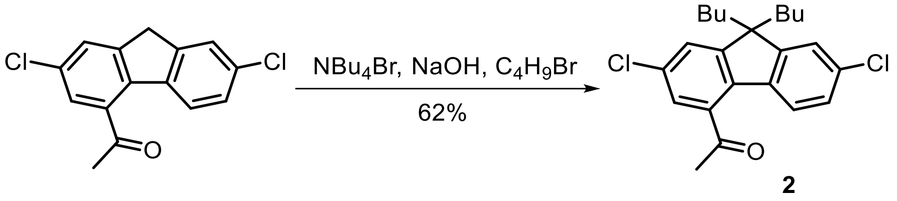

Under argon atmosphere, to a mixture of sodium hydroxide (10 mL, 50 wt%), tetrabutylammonium bromide (3.22 g, 10 mmol) and butyl bromide (64.20 mL, 600 mmol), 1-(2,7-dichloro-9*H*-fluoren-4-yl)ethan-1-one (5.54 g, 20 mmol) was added at 100 °C. After reacting at this temperature for 4 h, the reactant was cooled to room temperature and then extracted with CH_2Cl_2. The combined organic layer was washed with dilute HCl, H_2O and brine successively, and then dried over anhydrous $NaSO_4$. After removing the solvent and the excess butyl bromide in vacuo, the residue was purified by column chromatography (eluent: PE/DCM = 6:1) to give white solid **2** (4.82 g, 62%); M.p.: 109-110 °C.

1-(9,9-dibutyl-2,7-dichloro-9*H*-fluoren-4-yl)ethan-1-one (2)

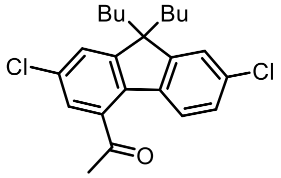

^{1}H NMR (600 MHz, $CDCl_3$): δ 7.94 (d, J = 8.4 Hz, 1H), 7.53 (d, J = 1.8 Hz, 1H), 7.41 (d, J = 1.8 Hz, 1H), 7.28 (d, J = 1.7 Hz, 1H), 7.27 – 7.25 (m, 1H), 2.70 (s, 3H), 1.95 – 1.90 (m, 4H), 1.08 (dd, J = 14.8, 7.4 Hz, 4H), 0.68 (t, J = 7.4 Hz, 6H), 0.52 (dd, J = 14.8, 7.4 Hz, 4H); **^{13}C NMR** (151 MHz, $CDCl_3$): δ 201.3, 154.8, 153.2, 137.2, 136.2, 136.0, 134.2, 132.6, 127.3, 127.3, 125.7, 125.4, 122.7, 54.7, 40.3, 30.0, 25.7, 22.9, 13.8; HRMS (APCI-TOF, positive mode, methanol/chloroform): *m/z* calcd for $C_{23}H_{27}Cl_2O$ $(M+H)^+$: 389.1433, found: 389.1429.

1.2.4 Synthesis of 1,3,5-tris(9,9-dibutyl-2,7-dichloro-9*H*-fluoren-4-yl)benzene (TFB, 3):

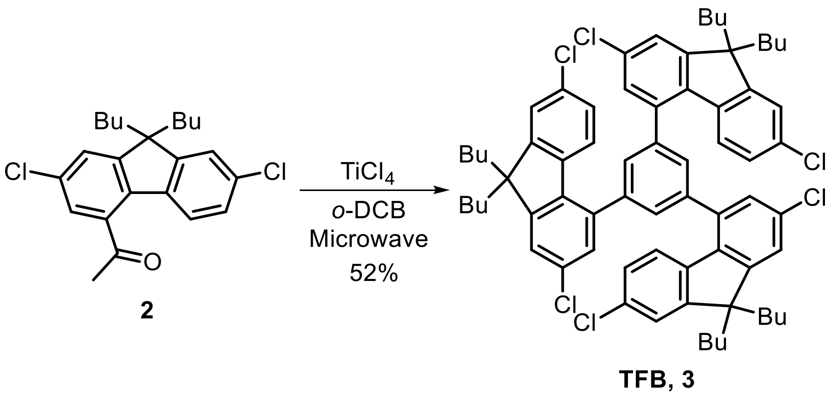

TFB, 3

In a glove box, 1-(9,9-dibutyl-2,7-dichloro-9*H*-uoren-4-yl)ethan-1-one **2** (390 mg, 1.0 mmol, 1.0 equiv.), $TiCl_4$ (0.7 mL, 6.6 mmol, 6.6 equiv.) and 5 mL dry *o*-dichlorobenzene were added to a 10 mL microwave vial and the vial was capped. The vessel was removed from the glove box and placed into a microwave reactor where it was heated at 180 °C for 3 h. After cooling down, the mixture was poured over concentrated hydrochloric acid/ice to quench the reaction and then extracted with CH_2Cl_2. The combined organic phase was washed with saturated aqueous $NaHCO_3$ and dried over Na_2SO_4. The organic solvent was removed at reduced pressure to give a yellow-brown solid. The above procedure was repeated 4 times and the crude material from each reaction was combined. The combined crude material

was purified by column chromatography over silica gel (eluent: petroleum ether) to afford compound **3** (970 mg, 52%) as an off-white powder; M.p.: 147-148 °C.

1,3,5-Tris(9,9-dibutyl-2,7-dichloro-9H-fluoren-4-yl)benzene (3)

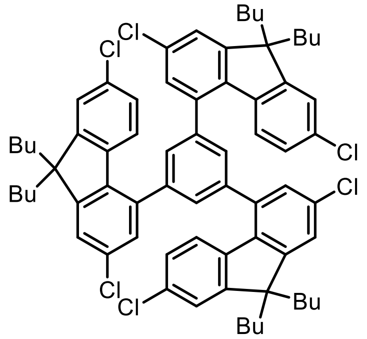

^{1}H NMR (600 MHz, $CDCl_3$): δ 7.67 (d, *J* = 14.9 Hz, 3H), 7.32 (s, 6H), 7.29 (d, *J* = 1.9 Hz, 3H), 7.12 (d, *J* = 8.2 Hz, 3H), 6.89 (d, *J* = 8.0 Hz, 3H), 1.98 – 1.91 (m, 12H), 1.13 – 1.07 (m, 12H), 0.69 (t, *J* = 7.4 Hz, 18H), 0.60 (d, *J* = 7.5 Hz, 12H); **^{13}C NMR** (151 MHz, $CDCl_3$): δ 152.5, 152.1, 139.7, 137.3, 136.3, 134.7, 132.3, 131.9, 128.2, 127.9, 125.7, 122.3, 122.0, 121.5, 54.0, 39.2, 24.8, 21.9, 12.7; **HRMS** (APCI-TOF, positive mode, methanol/chloroform): *m/z* calcd for $C_{69}H_{73}Cl_6$ $(M+H)^+$: 1111.3838, found: 1111.3836.

1.2.5 Synthesis of trifluorenocoronene (TFC, 4):

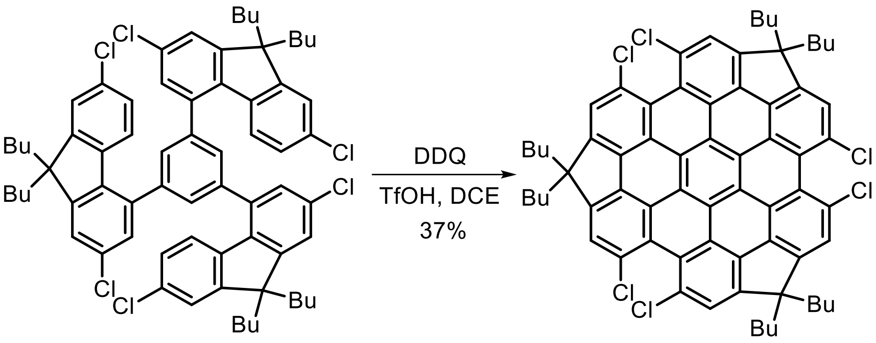

TFB, 3 **TFC, 4**

To a mixture of **3** (220 mg, 0.2 mmol, 1.0 equiv.) and DDQ (360 mg, 1.6 mmol, 8.0 equiv.) in 1,2-dichloroethane (20 mL) was added trifluoromethanesulfonic acid (1 mL) under argon atmosphere, and the mixture was stirred at 50 °C for 6 h. After cooling to room temperature, the reaction mixture was quenched by adding saturated aqueous $NaHCO_3$ (20 mL), and then the mixture was extracted with CH_2Cl_2 (3 × 30ml). The combined organic layer was dried over Na_2SO_4, and the solvent was removed under reduced pressure. The crude product was purified by silica gel column chromatography (eluent: petroleum ether) to give the trifluorenocoronene **4** (80 mg, 37%) as a yellow powder. M.p.: >300 °C.

Trifluorenocoronene (TFC, 4)

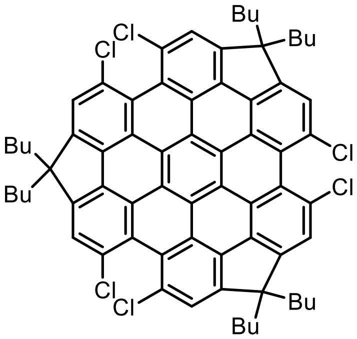

^{1}H NMR (600 MHz, Tol-*d*$_8$): δ 8.28 (s, 6H), 2.26 (dd, *J* = 9.8, 6.6 Hz, 12H), 1.16 (dd, *J* = 14.6, 7.3 Hz, 12H), 1.08 – 1.02 (m, 12H), 0.66 (t, *J* = 7.3 Hz, 18H); **^{13}C NMR** (151 MHz, Tol-*d*$_8$): δ 151.7, 135.0, 133.9, 127.3, 126.9, 126.1, 122.0, 63.1, 38.8, 27.9, 23.9, 14.4; **HRMS** (APCI-TOF, positive mode, methanol/chloroform): *m/z* calcd for $C_{69}H_{61}Cl_6$ $(M+H)^+$: 1099.2899, found: 1099.2898.

1.2.6 Synthesis of trithiasupersumanene (1a):

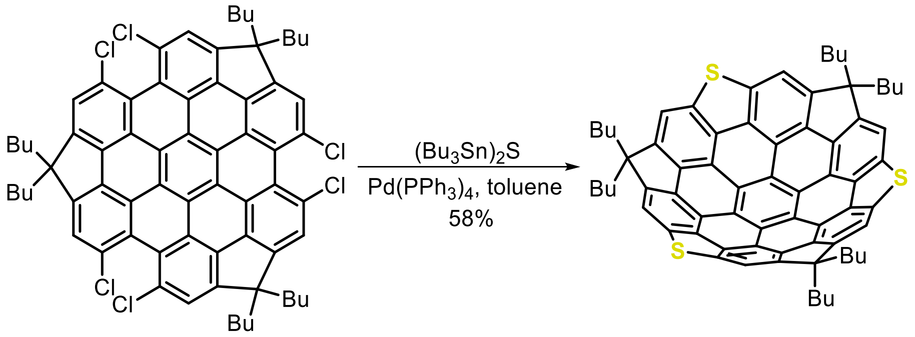

TFC, 4 **1a**

In a glovebox, to an oven-dried pressure vessel with a Teflon screw cap was added compound **4** (110 mg, 0.1 mmol, 1.0 equiv.), $Bu_3SnSSnBu_3$ (245 mg, 0.4 mmol, 4.0 equiv.) and $Pd(PPh_3)_4$ (115 mg, 0.1 mmol, 1.0 equiv.) and 5 mL dry and degassed toluene. After the vessel was resealed and moved out from the glovebox, the mixture was heated and stirred at 150 °C for 48 h. On cooling to room temperature, the reaction mixture was quenched with saturated aqueous KF solution (30 mL) and extracted with CH_2Cl_2 (3 × 20ml). The combined organic phase was dried over anhydrous Na_2SO_4 before being filtered and concentrated down to a solid under reduced pressure. The crude solid was adsorbed onto silica gel and subjected to silica gel column chromatography (eluent: petroleum ether) to give the trithiasupersumanene **1a** (57 mg, 58% yield) as a yellowish powder. M.p.: >300 °C.

Trithiasupersumanene (1a)

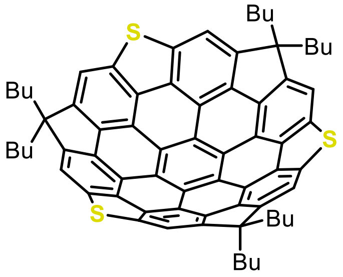

^{1}H NMR (600 MHz, $CDCl_3$): δ 8.06 (s, 6H), 2.70 – 2.66 (m, 6H), 2.13 – 2.09 (m, 6H), 2.05 – 2.00 (m, 6H), 1.58 – 1.56 (m, 6H), 1.05 (t, J = 7.4 Hz, 9H), 0.59 (dd, J = 14.7, 7.4 Hz, 6H), -0.02 (t, J = 7.3 Hz, 9H), -1.01 – -1.07 (m, 6H). **^{13}C NMR** (151 MHz, $CDCl_3$): δ 153.0, 142.9, 139.8, 134.8, 130.8, 128.6, 119.6, 64.4, 42.1, 35.8, 28.7, 26.1, 23.6, 22.5, 14.3, 13.2; **HRMS** (APCI-TOF, positive mode, methanol/chloroform): m/z calcd for $C_{69}H_{61}S_3$ $(M+H)^+$: 985.3930, found: 985.3945.

1.2.7 Synthesis of triselenosupersumanene (1b):

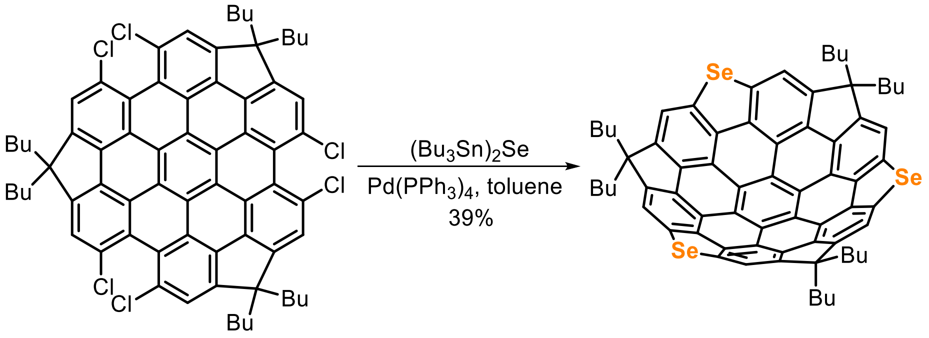

TFC, 4 **1b**

In a glovebox, to an oven-dried pressure vessel with a Teflon screw cap was added compound **4** (110 mg, 0.1 mmol, 1.0 equiv.), $Bu_3SnSeSnBu_3$[1] (263 mg, 0.4 mmol, 4.0 equiv.) and $Pd(PPh_3)_4$ (115 mg, 0.1

mmol, 1.0 equiv.) and 5 mL dry and degassed toluene. After the vessel was resealed and moved out from the glovebox, the mixture was heated and stirred at 150 °C for 48 h. On cooling to room temperature, the reaction mixture was quenched with saturated aqueous KF solution (30 mL) and extracted with CH_2Cl_2 (3 × 20ml). The combined organic phase was dried over anhydrous Na_2SO_4 before being filtered and concentrated down to a solid under reduced pressure. The crude solid was adsorbed onto silica gel and subjected to silica gel column chromatography (eluent: petroleum ether) to give the triselenosupersumanene **1b** (44 mg, 39% yield) as a yellowish powder. M.p.: >300 °C.

Triselenosupersumanene (1b)

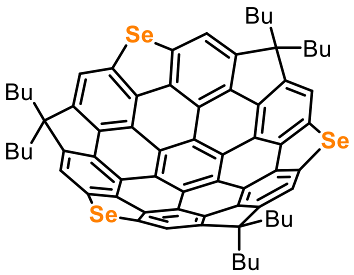

^{1}H NMR (600 MHz, $CDCl_3$) δ 8.22 (s, 6H), 2.72 – 2.65 (m, 6H), 2.18 – 2.12 (m, 6H), 2.00 (td, J = 12.0, 10.2, 6.2 Hz, 6H), 1.63 – 1.49 (m, 9H), 1.04 (t, J = 7.3 Hz, 9H), 0.60 (h, J = 7.4 Hz, 6H), 0.01 (t, J = 7.3 Hz, 9H), -0.92 – -1.01 (m, 6H); **^{13}C NMR** (151 MHz, $CDCl_3$): δ 151.8, 142.0, 139.0, 136.1, 129.4, 128.3, 121.7, 64.4, 41.6, 36.5, 28.5, 25.8, 23.6, 22.4, 14.3, 13.2; **HRMS** (APCI-TOF, positive mode, methanol/chloroform): *m/z* calcd for $C_{69}H_{61}Se_3$ $(M+H)^+$: 1127.2296, found: 1127.2289.

1.2.8 Synthesis of 1-(9,9-dimethyl-2,7-dichloro-9*H*-fluoren-4-yl)ethan-1-one (2-Me):

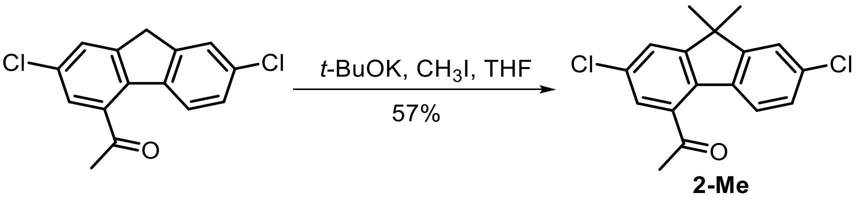

To an oven-dried pressure vessel with a Teflon screw cap add 1-(2,7-dichloro-9H-fluoren-4-yl)ethan-1-one (5.55 g, 20 mmol) and potassium tert-butoxide (6.74 g, 60 mmol, 3.0 equiv.) in 150 mL anhydrous tetrahydrofuran under argon atmosphere. Cool the solution to 0 °C and iodomethane (8.53 g, 60 mmol, 3.0 equiv.) was added, then recover the solution to room temperature. After stirring the solution for 12 hours, add 100 mL deionized water to the solution and then extracted with CH_2Cl_2. The combined organic phase was dried over anhydrous Na_2SO_4 before being filtered and concentrated down to a liquid under reduced pressure. The crude liqud was adsorbed onto silica gel and subjected to silica gel column chromatography (eluent: PE/DCM = 5:1) to give **2-Me** (3.5 g, 57% yield) as a brown liquid.

1-(2,7-dichloro-9,9-dimethyl-9*H*-fluoren-4-yl)ethan-1-one (2-Me)

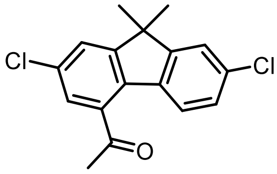

^{1}H NMR (600 MHz, $CDCl_3$) δ 7.85 (d, J = 8.4 Hz, 1H), 7.44 (dd, J = 13.8, 2.0 Hz, 2H), 7.30 (d, J = 2.1 Hz, 1H), 7.20 – 7.18 (m, 1H), 2.63 (s, 3H), 1.39 (s, 6H); **^{13}C NMR** (151 MHz, $CDCl_3$) δ 200.3, 156.3, 154.9, 135.4, 134.1, 133.2, 133.2, 131.7, 126.5, 126.2, 124.9, 124.5, 121.8, 76.2, 76.0, 75.8, 45.4, 28.9, 26.0; **HRMS** (APCI-TOF, positive mode, methanol/chloroform): *m/z* calcd for $C_{17}H_{15}Cl_2O$ $(M+H)^+$: 305.0494, found: 305.0495.

1.2.9 Synthesis of trithiasupersumanene (1a-Me):

Trithiasupersumanene **1a-Me** was obtained according to the similar protocol with **1a**.

1,3,5-Tris(9,9-dimethyl-2,7-dichloro-9H-fluoren-4-yl)benzene (3-Me) :

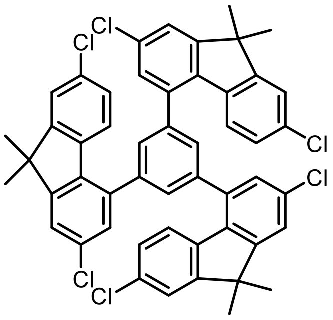

^{1}H NMR (600 MHz, $CDCl_3$) δ 7.77 (s, 3H), 7.47 (dd, *J* = 11.0, 2.0 Hz, 6H), 7.43 – 7.37 (m, 3H), 7.31 – 7.17 (m, 3H), 7.11 – 6.87 (m, 3H), 1.57 (s, 18H); **^{13}C NMR** (151 MHz, $CDCl_3$) δ 155.1, 154.9, 139.8, 139.5, 136.6, 135.3, 132.7, 132.4, 132.0, 128.1, 127.9, 125.9, 125.7, 122.4, 122.2, 121.6, 76.2, 76.0, 75.8, 45.8, 26.1; **HRMS** (APCI-TOF, positive mode, methanol/chloroform): *m/z* calcd for $C_{51}H_{37}Cl_6$ $(M+H)^+$: 859.1021, found: 859.1020; **yield:** 56%; **M.p.:** >300 °C.

Hexachloro-trifluorenocoronene (TFC, 4-Me):

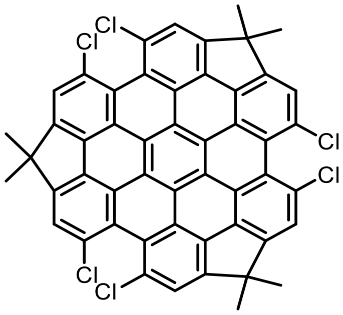

Due to the poor solubility of compound **4-Me**, high quality of ^{1}H NMR and ^{13}C NMR spectrum can not be obtained. **HRMS** (APCI-TOF, positive mode, methanol/chloroform): *m/z* calcd for $C_{51}H_{25}Cl_6$ $(M+H)^+$: 847.0082, found: 847.0081; **yield:** 39%; **M.p.:** >300 °C.

Trithiasupersumanene (1a-Me):

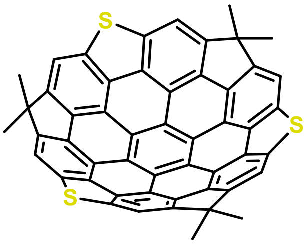

^{1}H NMR (600 MHz, Tol-*d*$_8$) δ 7.70 (s, 6H), 2.15 (s, 9H), 0.99 (s, 9H); **^{13}C NMR** (150 MHz, Tol-*d*$_8$): δ 155.9, 143.4, 138.4, 137.0, 135.0, 130.5, 118.0, 55.4, 29.1, 24.0; **HRMS** (APCI-TOF, positive mode, methanol/chloroform): *m/z* calcd for $C_{51}H_{25}S_3$ $(M+H)^+$: 733.1113, found: 733.1120; **yield:** 28%; **M.p.:** >300 °C.

2. Crystallographic details

High quality single crystals of compound **1a** and **1b** were grown by diffusing methanol into their solution in chloroform. After two weeks, orange crystals suitable for X-ray structural determination were obtained. The X-ray diffraction data were collected on Bruker D8 Venture X-ray single crystal diffractometer using Cu radiation at 153 K. Absorption correction was carried out by a multi-scan method. The structure was solved by direct methods with SHELXT[5] program and refined by least-square methods with SHELXL[6] program contained in Olex2 suite[7]. Details of the crystal and refinement results were listed in Supplementary Table 1 and Table 2.

2.1 X-ray Crystallography for (1a)

Supplementary Table 1. Crystallographic data and structure refinement details for **1a**

Empirical formula	$C_{69}H_{60}S_3{\cdot}(CHCl_3)$
Formula weight	1104.71
Temperature/K	153.0
Crystal system	monoclinic
Space group	$P2_1/c$
a/Å	25.7504(12)
b/Å	23.7050(11)
c/Å	29.4867(13)
α/°	90
β/°	112.839(2)
γ/°	90
Volume/$Å^3$	16587.9(13)
Z	12
$\rho_{calc}g/cm^3$	1.327
μ/mm^{-1}	2.891
F(000)	6960.0
Crystal size/mm^3	0.5 × 0.4 × 0.3
Radiation	CuKα (λ = 1.54178)
2Θ range for data collection/°	4.946 to 136.698
Index ranges	$-31 \le h \le 30, -28 \le k \le 28, -35 \le l \le 35$
Reflections collected	259328
Independent reflections	30259 [R_{int} = 0.0516, R_{sigma} = 0.0250]
Data/restraints/parameters	30259/920/2269
Goodness-of-fit on F^2	1.053
Final R indexes [I>=2σ (I)]	R_1 = 0.0534, wR_2 = 0.1514
Final R indexes [all data]	R_1 = 0.0604, wR_2 = 0.1585
Largest diff. peak/hole / e $Å^{-3}$	1.56/-0.96

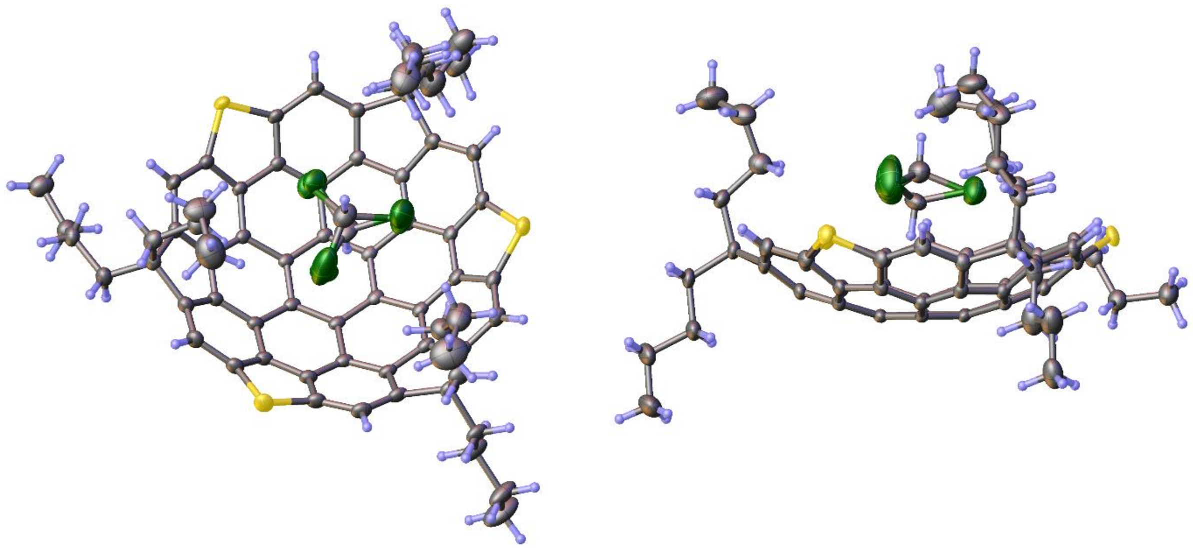

Supplementary Figure 1. Crystal structure of **1a**: top view and side view. Thermal ellipsoids are shown at 30% probability.

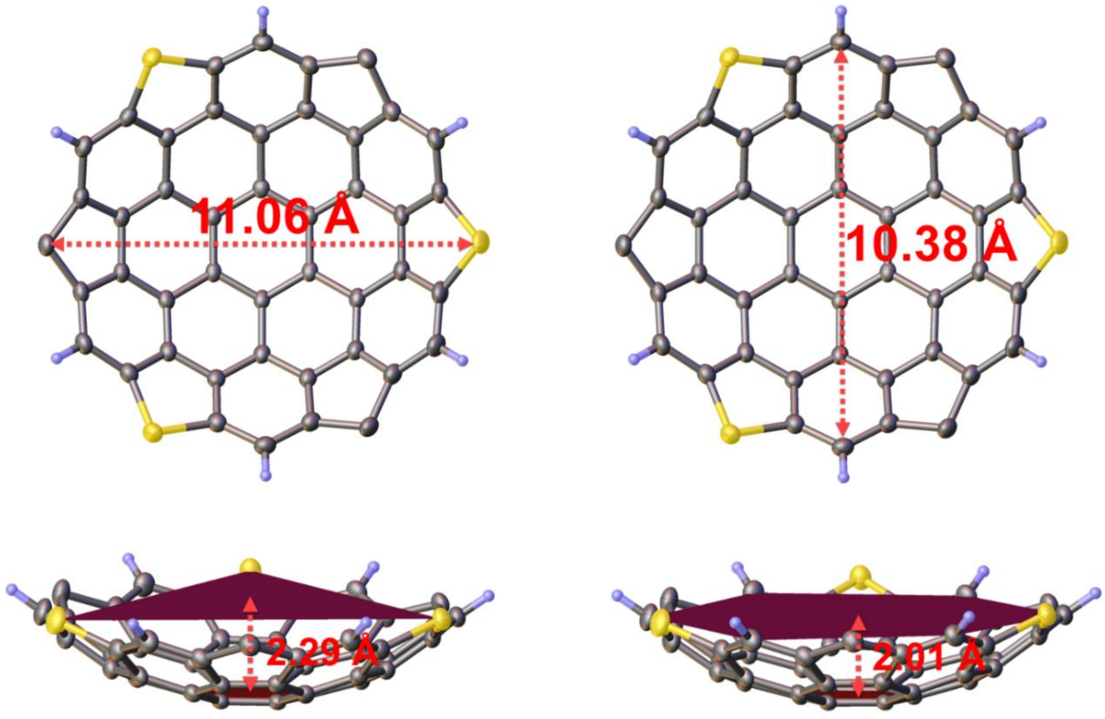

Supplementary Figure 2. The diameters and depths of **1a**. Butyl groups and solvent molecules are omitted for clarity. Thermal ellipsoids are shown at 30% probability.

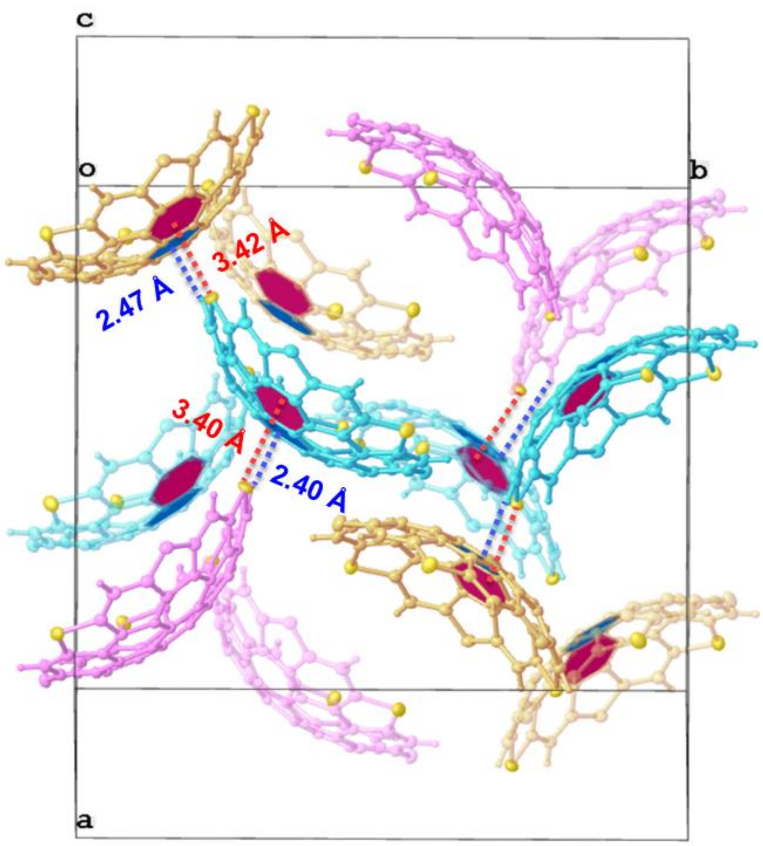

Supplementary Figure 3. Three types of crystallographic independent molecules of **1a**. C-H···π interactions and S···π interactions in one unit cell of **1a**. Butyl groups and solvent molecules are omitted for clarity; thermal ellipsoids are shown at 30% probability.

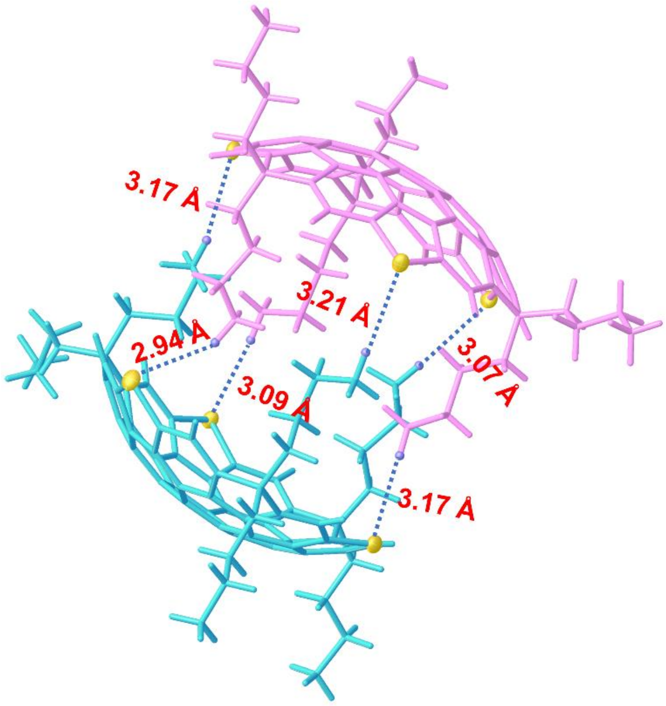

Supplementary Figure 4. Concave-concave packing motifs of **1a** stabilized with six C-H···S interactions. Solvent molecules are omitted for clarity.

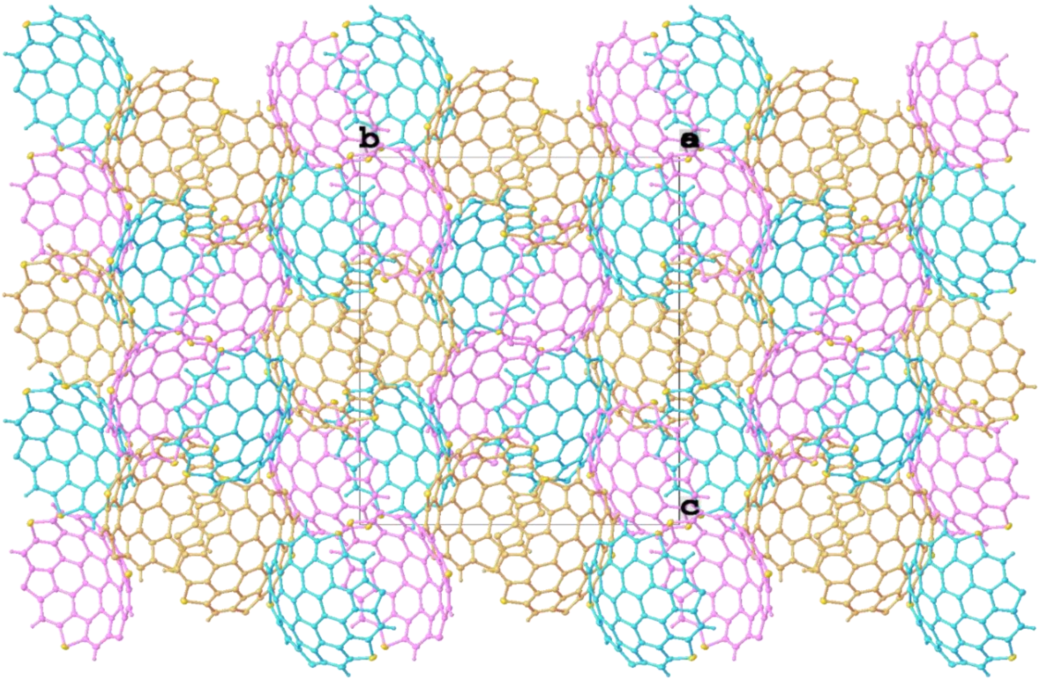

Supplementary Figure 5. View along *a*-axis.

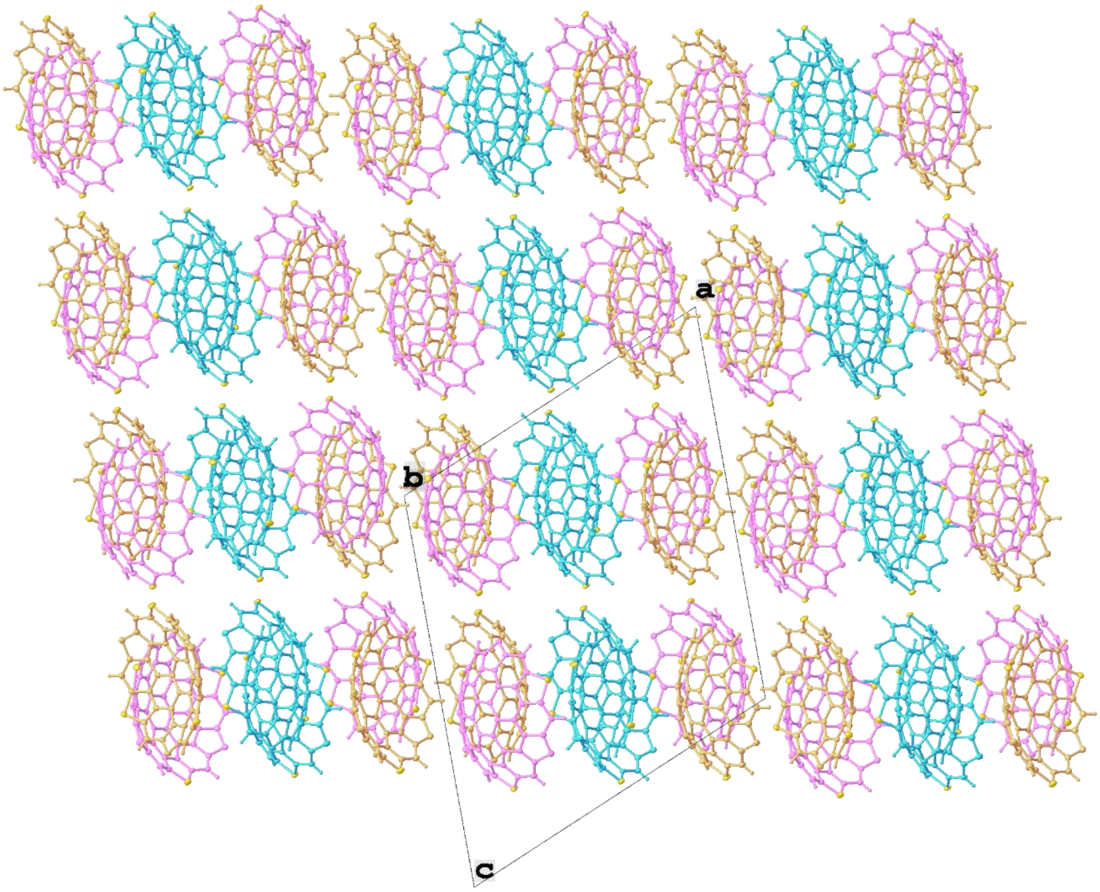

Supplementary Figure 6. View along *b*-axis.

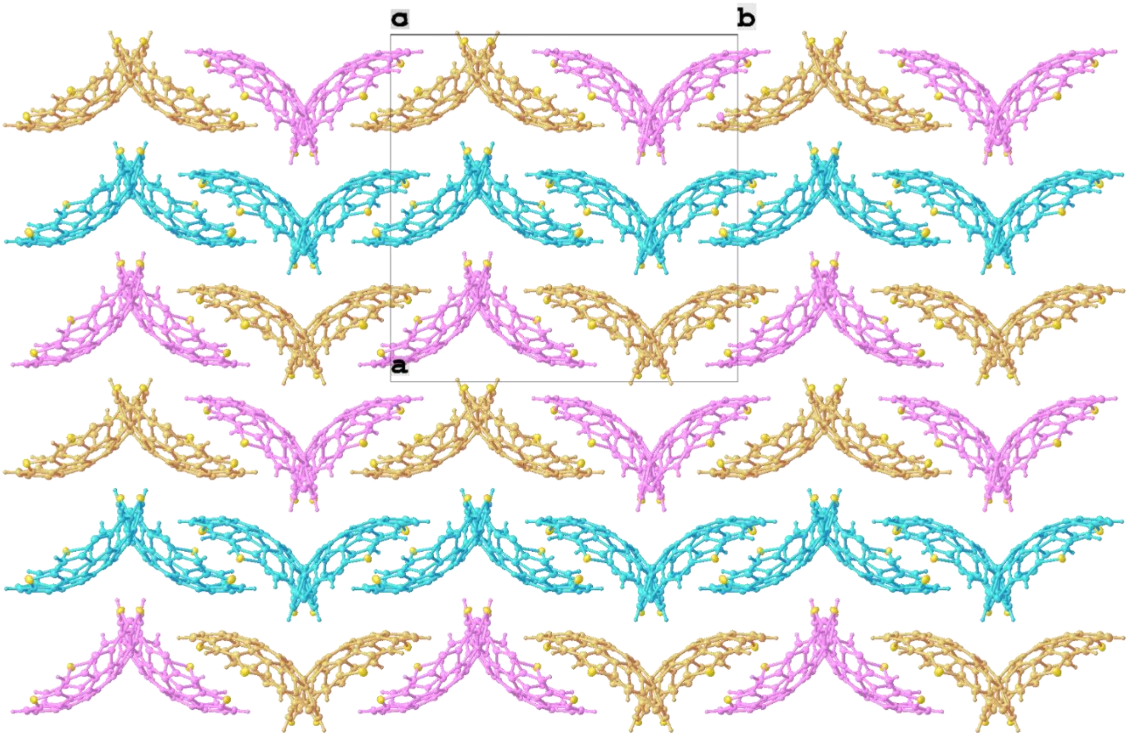

Supplementary Figure 7. View along *c*-axis.

Average bond lengths and average BLA values for (1a)

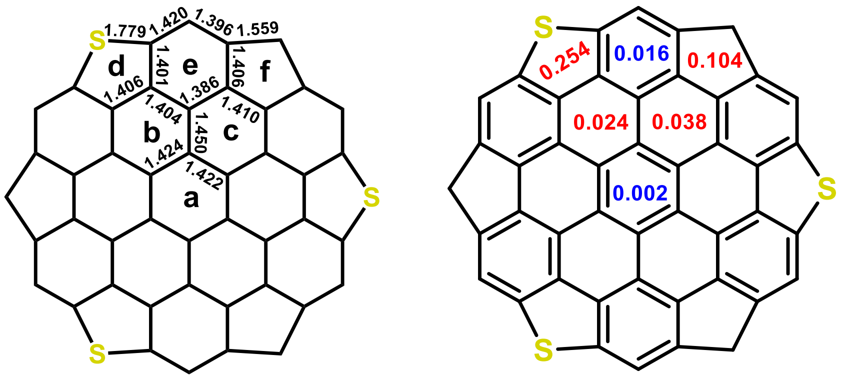

Supplementary Figure 8. Left: average bond lengths in Angstrom (Å) based on the result of X-ray crystal structure analysis; right: average bond length alternation (BLA) of rings based on the result of X-ray crystal structure analysis. The mean values were calculated by averaging the all values of equivalent position. The pronounced aromatic character with tiny BLA values of rings a and e were denoted as blue color.

Average POAV angles for (1a)

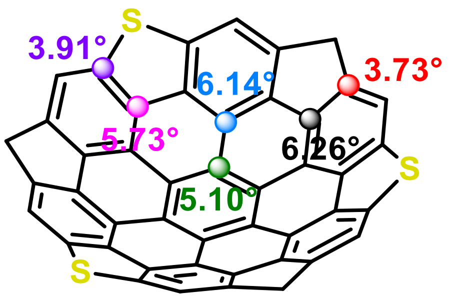

Supplementary Figure 9. POAV angles of **1a** determined from the X-ray structure. The mean values were calculated by averaging the all values of equivalent position. POAV angles were calculated based on the three sigma bond angles at a conjugated carbon atom. [8] Butyl groups were omitted for clarity.

2.2 X-ray Crystallography for (1b)

Supplementary Table 2. Crystallographic data and structure refinement details for **1b**

Empirical formula	$C_{69}H_{60}Se_3{\cdot}(CHCl_3)$
Formula weight	1245.41
Temperature/K	153.15
Crystal system	monoclinic
Space group	$P2_1/c$
a/Å	16.5069(9)
b/Å	23.6255(13)
c/Å	29.7702(15)
α/°	90
β/°	103.380(2)
γ/°	90
Volume/Å^3	11294.8(10)
Z	8
ρ_{calc}g/cm^3	1.465
μ/mm^{-1}	4.006
F(000)	5072.0
Crystal size/mm^3	0.5 × 0.4 × 0.3
Radiation	CuKα (λ = 1.54178)
2Θ range for data collection/°	4.826 to 136.61
Index ranges	-19 ≤ h ≤ 19, -28 ≤ k ≤ 28, -35 ≤ l ≤ 35
Reflections collected	189744
Independent reflections	20658 [R_{int} = 0.0531, R_{sigma} = 0.0253]
Data/restraints/parameters	20658/13/1457
Goodness-of-fit on F^2	1.023
Final R indexes [I>=2σ (I)]	R_1 = 0.0363, wR_2 = 0.0949
Final R indexes [all data]	R_1 = 0.0394, wR_2 = 0.0976
Largest diff. peak/hole / e Å^{-3}	1.23/-0.94

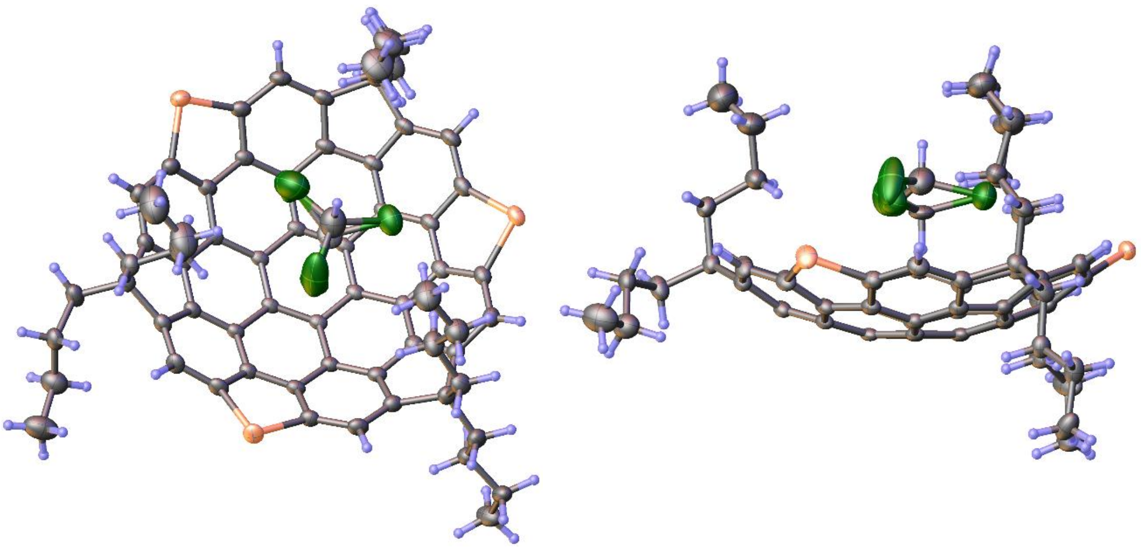

Supplementary Figure 10. Crystal structure of **1b**: top view and side view. Thermal ellipsoids are shown at 30% probability.

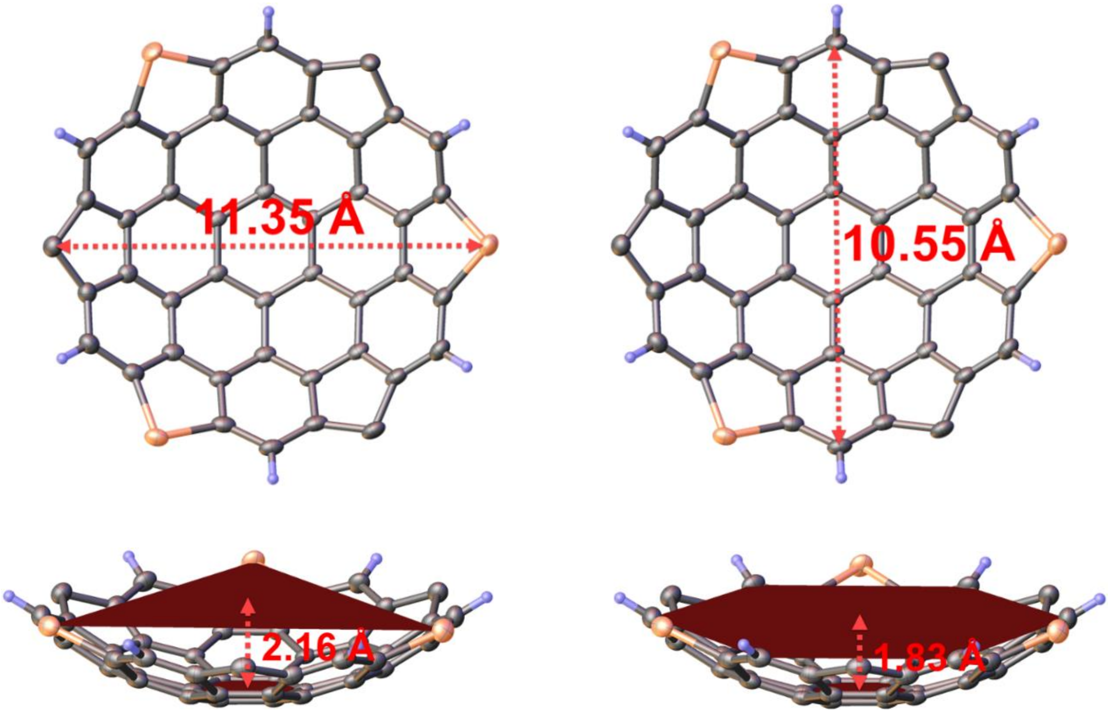

Supplementary Figure 11. The average diameters and depths of **1b**. Butyl groups and solvent molecules are omitted for clarity; thermal ellipsoids are shown at 30% probability.

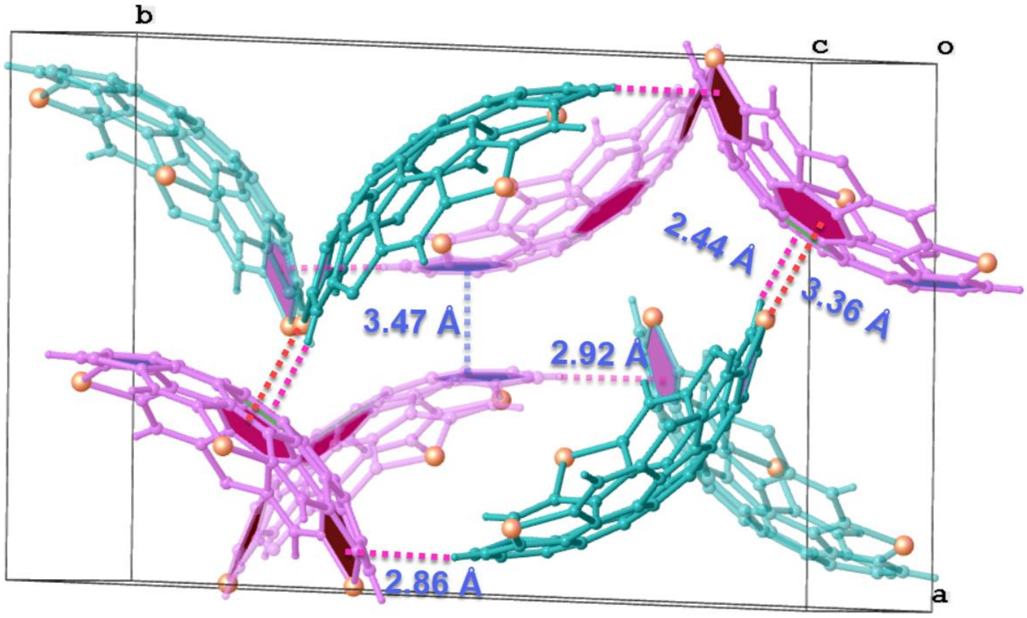

Supplementary Figure 12. Two types of crystallographic independent molecules of **1b**. C-H···π interactions and Se···π interactions in one unit cell of **1b**. Butyl groups and solvent molecules are omitted for clarity; thermal ellipsoids are shown at 30% probability.

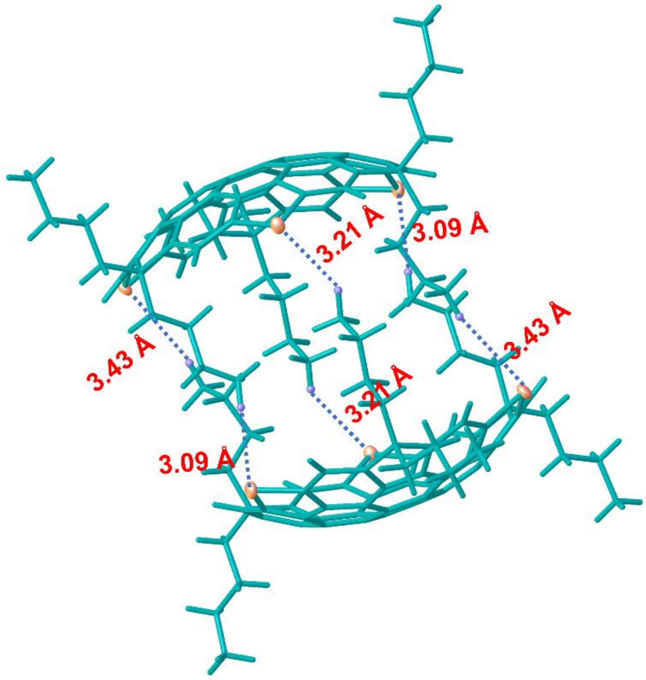

Supplementary Figure 13. Concave-concave packing motifs of **1b** stabilized with six C-H···Se interactions. Solvent molecules are omitted for clarity.

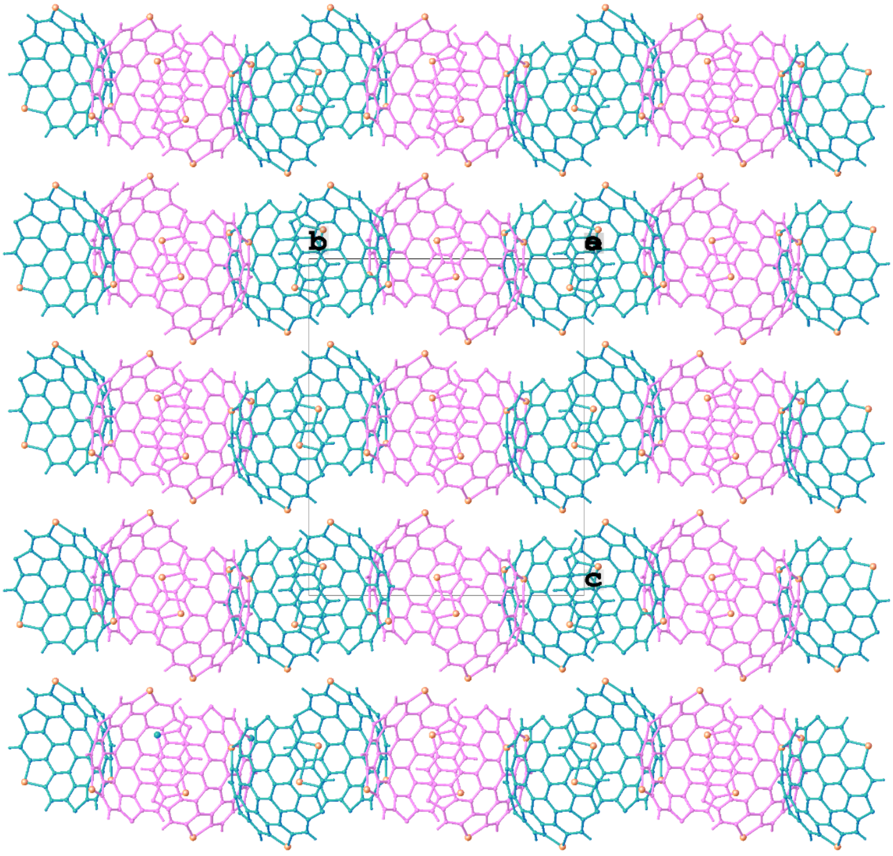

Supplementary Figure 14. View along a-axis.

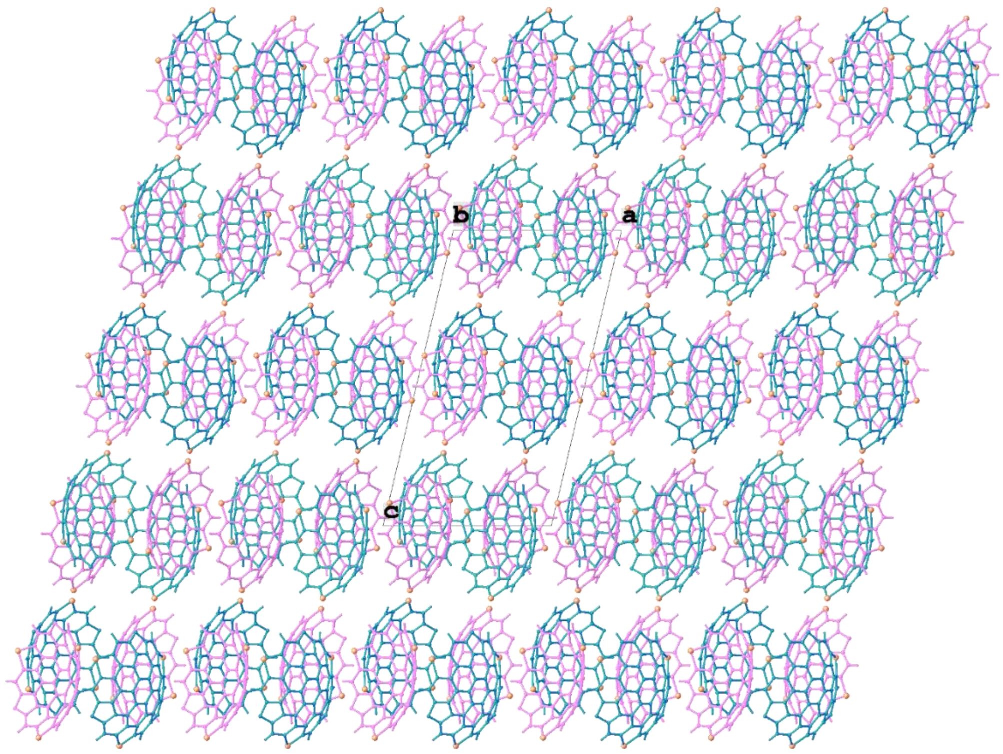

Supplementary Figure 15. View along b-axis.

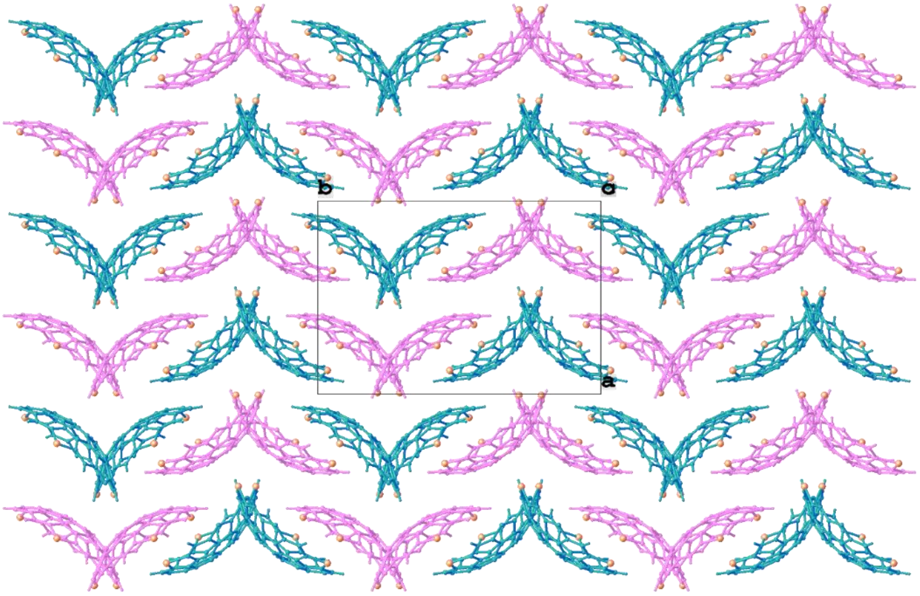

Supplementary Figure 16. View along c-axis.

Average bond lengths and average BLA values for (1b)

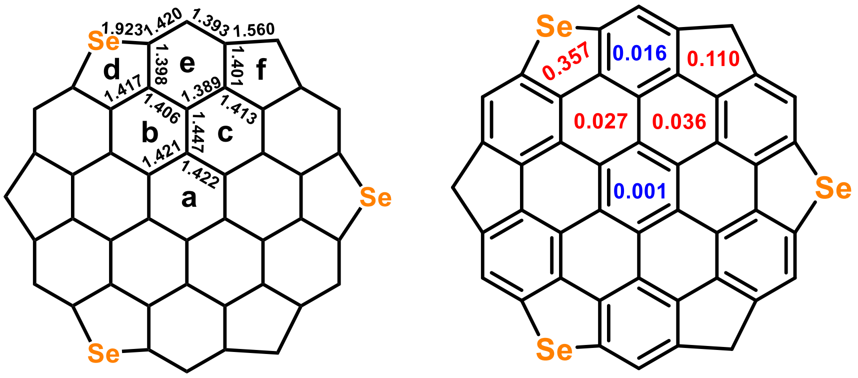

Supplementary Figure 17. Left: average bond lengths in Angstrom (Å) based on the result of X-ray crystal structure analysis; right: average bond length alternation (BLA) of rings based on the result of X-ray crystal structure analysis. The mean values were calculated by averaging the all values of equivalent position. The pronounced aromatic character with tiny BLA values of rings a and e were denoted as blue colour.

Average POAV angles for (1b)

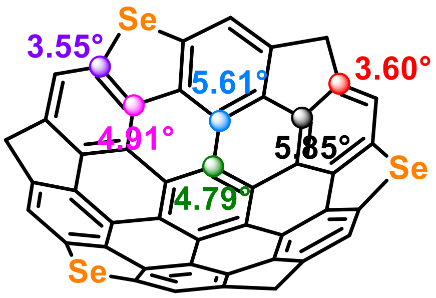

Supplementary Figure 18. POAV angles of 1b determined from the X-ray structure. The mean values were calculated by averaging the all values of equivalent position. POAV angles were calculated based on the three sigma bond angles at a conjugated carbon atom.[8] Butyl groups were omitted for clarity.

Average bond lengths and average BLA values for *p*-HBC

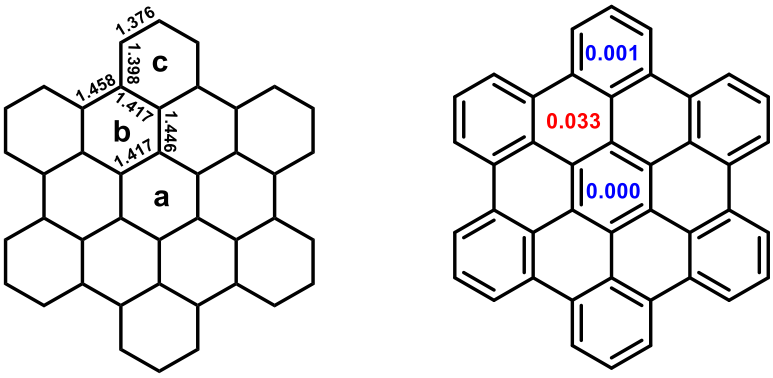

Supplementary Figure 19. Left: average bond lengths in Angstrom (Å) based on the result of X-ray crystal structure analysis; right: average bond length alternation (BLA) of rings based on the result of X-ray crystal structure analysis. [9] The mean values were calculated by averaging the all values of equivalent position. The pronounced aromatic character with tiny BLA values of rings a and c were denoted as blue color.

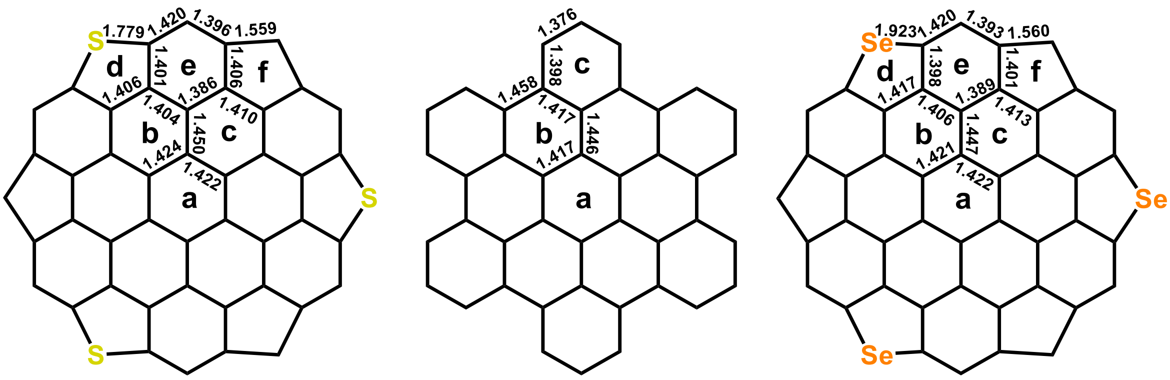

Supplementary Figure 20. The comparison of average bond lengths (Å) in **1a**, **1b** and ***p*-HBC** based on the result of X-ray crystal structure analysis.

3. Photophysical Properties

UV-Vis absorption spectra were measured on a Hitachi U-3900/3900H spectrophotometer with dilute dichloromethane solution (1.0×10^{-5} M) in spectral grade solvent at room temperature with a 1 cm square quarts cell. Emission spectra were measured on a fluorolog-3 fluorescence spectrophotometer (Horiba JY, USA) spectrophotometer with dilute solutions (1.0×10^{-6} M) in spectral grade solvent in a 1 cm square quartz cell upon the excitation at 400 nm for both **1a** and **1b**. Absolute fluorescence quantum yields were determined on a HAMAMATSU Absolute PL Quantum Yield Measurement System C9920-02G calibrated integrating sphere system with diluted and deaerated solutions (10^{-6} M order) in degassed spectral grade solvent at room temperature. The lifetimes of **1a** were measured at room temperature with a FLSP920 fluorescence spectrophotometer with diluted and deaerated chloroform solutions (10^{-6} M order). The results are summarized in Supplementary Table 3.

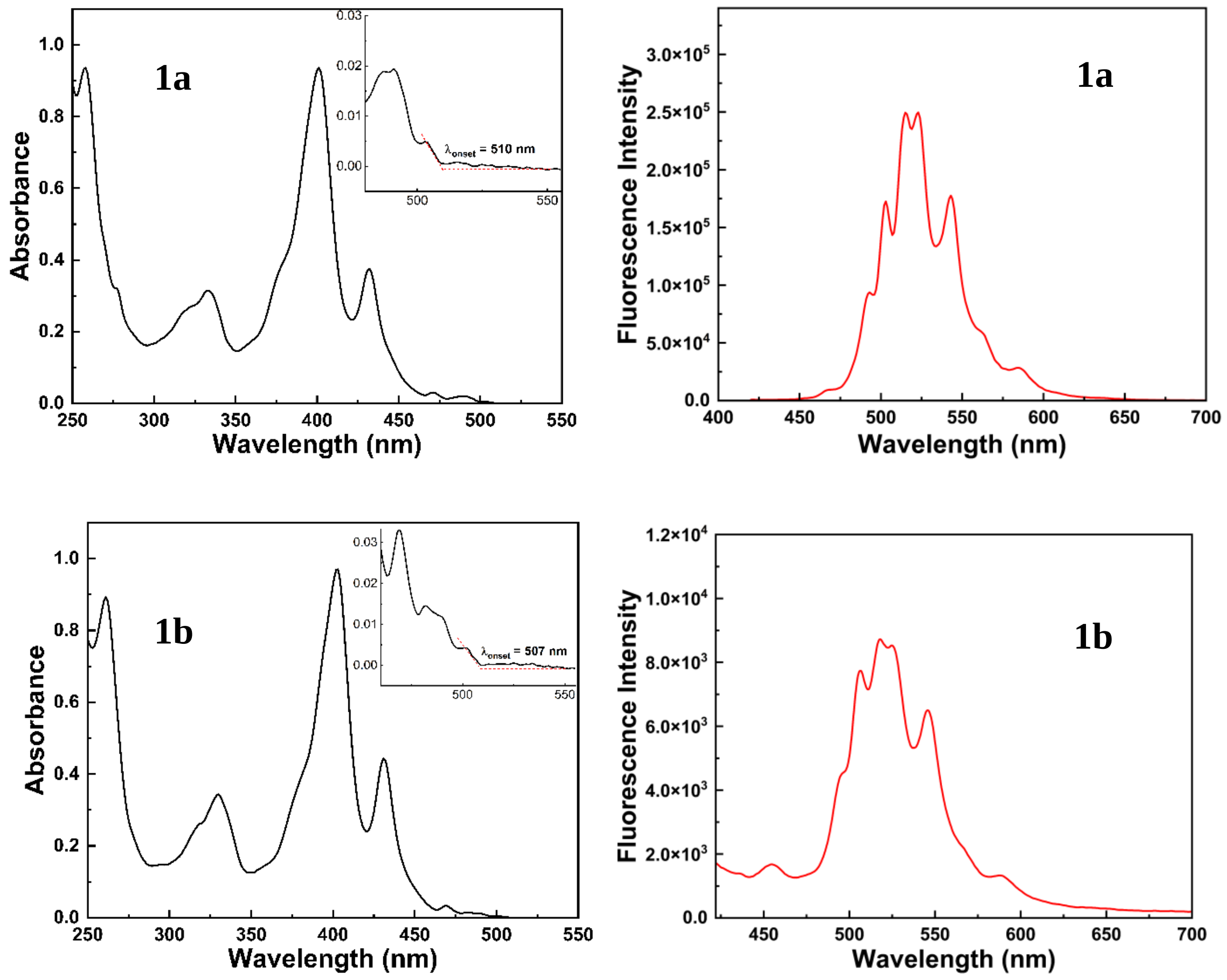

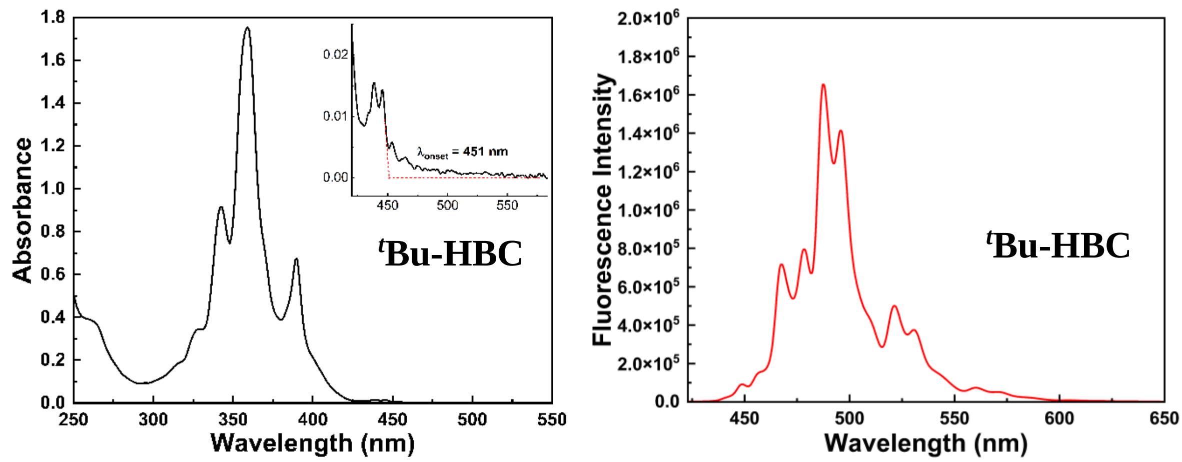

Supplementary Figure 21. UV-Vis absorption (black, 1.0× 10^{-5} M in CH_2Cl_2), and emission (red, 1.0× 10^{-6} M in CH_2Cl_2, λ_{ex} = 400 nm) spectra.

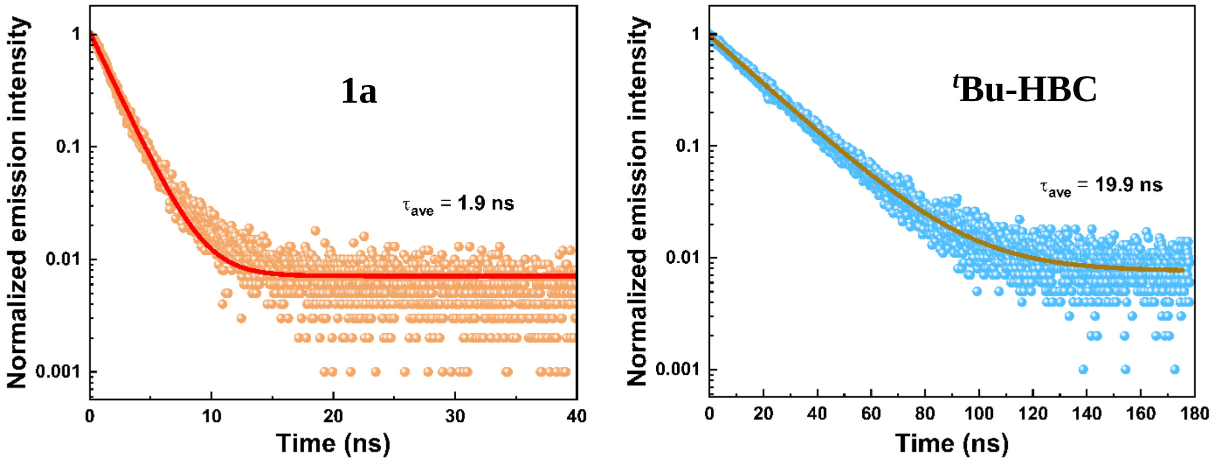

Supplementary Figure 22. Time-resolved emission decay of **1a** and ᵗBu-HBC in CH_2Cl_2 solution.

Supplementary Table 3. Summary of optical properties of **1a**, **1b** and **ᵗBu-HBC**

Compd	λ_{max}^{abs} [nm][a]	λ_{onset} [nm]	λ_{max}^{em} [nm][a]	Stokes shift [nm][b]	Φ_f[c]	τ_f [ns][d]	k_f [ns^{-1}][e]	k_{nr} [ns^{-1}][f]	E_g^{opt} [ev][g]
**1a**	401	510	515,523	117	0.043	1.9	0.023	0.504	2.43
**1b**	403	507	514,521	115	—	—	—	—	2.45
**ᵗBu-HBC**	359	451	487	128	0.045	19.9	0.0023	0.048	2.75

[a] Measured in dilute CH_2Cl_2 solution (1.0 × 10^{-5} M for absorption and 1.0 × 10^{-6} M for emission). [b] Stokes shift = (λ_{max}^{em} - λ_{max}^{abs}) nm. [c] Absolute fluorescence quantum yield determined with a calibrated integrating sphere system. [d] τ_f: area-weighted mean fluorescence lifetime, $\tau_f = \Sigma(A_n\tau_n^2)/\Sigma(A_n\tau_n)$, where A_n is the coefficient of the exponential functions for the *n*-th component. k_f: fluorescence emission rate constant, $k_f = \Phi_f/\tau_f$. k_{nr}: nonradiative decay rate constant, $k_{nr} = (1-\Phi_f)/\tau_f$. [g] Calculated by the onset of absorption according to E_g^{opt}= (1240/λ_{onset}) eV.

4. Electrochemical Properties

Cyclic voltammetry (CV) and differential pulse voltammetry (DPV) were performed on a CHI 660B electrochemical analyzer at room temperature in inert atmosphere with a three-electrode configuration in CH_2Cl_2 solution (purchased from Sigma-Aldrich) containing the substrate (typically 2×10^{-3} M) and 0.1 M tetrabutylammonium hexafluorophosphate (n-Bu_4NPF_6) as the supporting electrolyte. A platinum disc, a platinum plate, and a silver wire electrode were served as the working electrode, the counter electrode and the quasi-reference electrode (QRE), respectively. All potentials were calibrated versus an aqueous SCE by the addition of ferrocene as an internal standard taking $E_{1/2}(Fc/Fc^+) = 0.424$ V vs. SCE.[2] The scan rate was 0.1 V/s. The HOMO and LUMO energy values were estimated from the onset potentials of the first oxidation and reduction event, respectively. The HOMO and LUMO energy levels were calculated according to the following equations:

$$E_{HOMO}\ (\text{eV}) = -\ [E_{onset}^{ox}\ (vs\ \text{SCE}) - E_{1/2}(\text{Fc/Fc}^+) + 4.8] = -\ [E_{onset}^{ox}\ (vs\ \text{SCE}) + 4.38]$$

$$E_{LUMO}\ (\text{eV}) = -\ [E_{onset}^{re}\ (vs\ \text{SCE}) - E_{1/2}(\text{Fc/Fc}^+) + 4.8] = -\ [E_{onset}^{re}\ (vs\ \text{SCE}) + 4.38]$$

Where $E_{1/2}(Fc/Fc^+)$ is the half-wave potential of the Fc/Fc^+ couple against the SCE electrode.

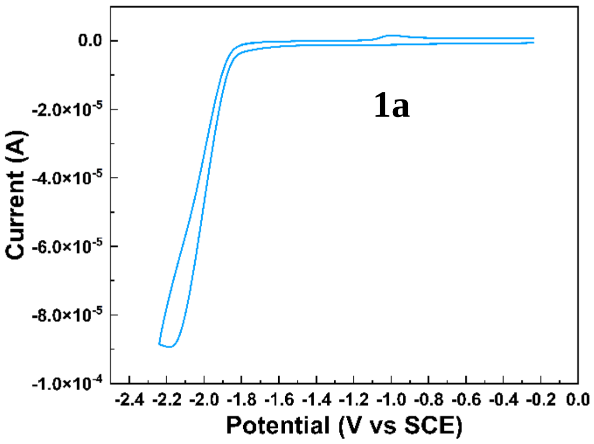

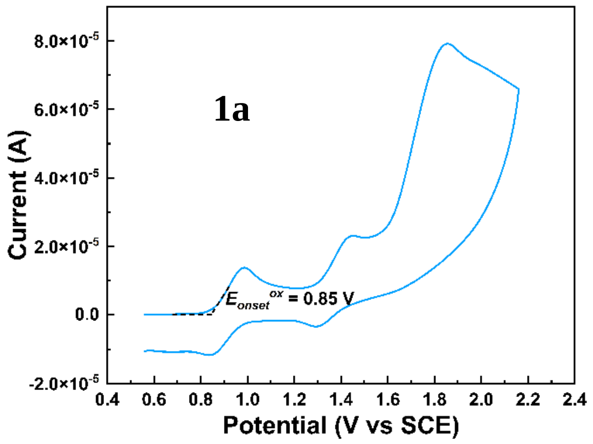

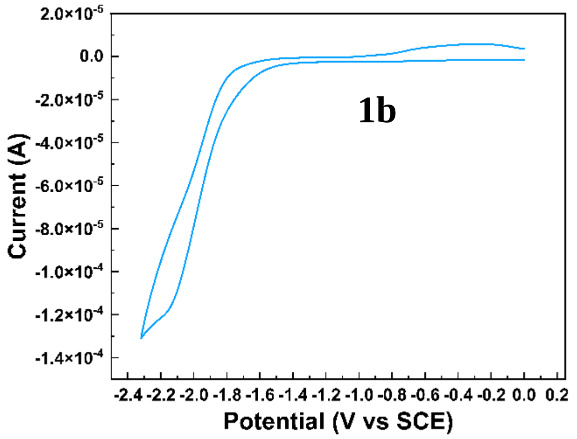

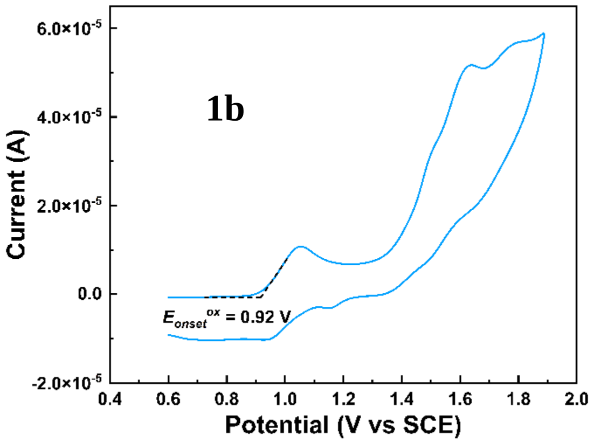

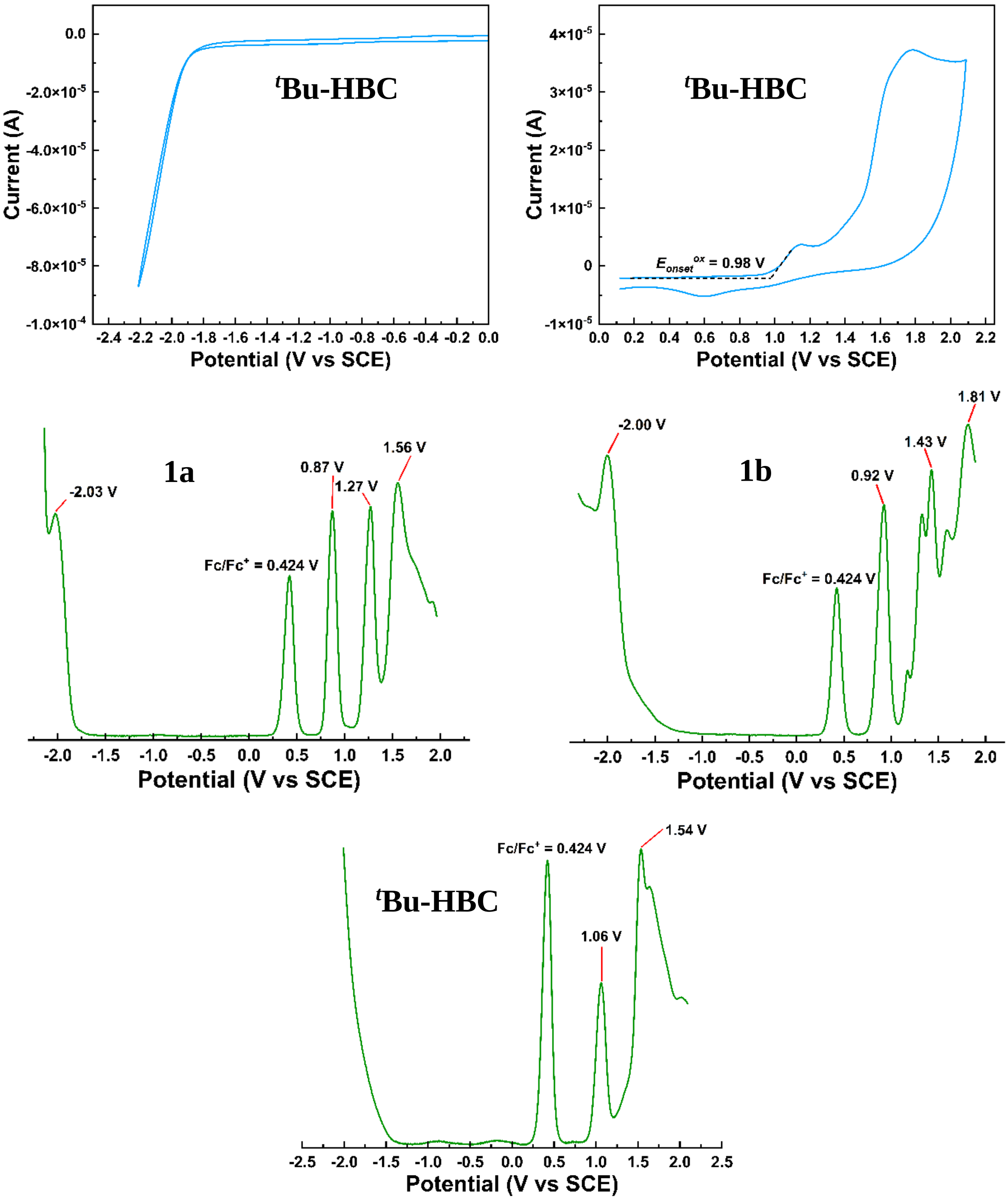

Supplementary Figure 23. Cyclic voltammogram and differential pulse voltammogram of **1a**, **1b** and ^{t}Bu-HBC in CH_2Cl_2 (0.1 mol/L *n*-Bu_4NPF_6) at a scan rate of 0.1 V/s.

Supplementary Table 4. Summary of electrochemical properties of **1a**, **1b** and **^{t}Bu-HBC**

Compd	$E_{1/2}^{re}$ [V][a]	E_{LUMO} [eV][b]	$E_{1/2}^{ox1}$ [V][a]	$E_{1/2}^{ox2}$ [V][a]	E_{onset}^{ox} [V]	E_{HOMO} [eV][c]	$E_{g}^{electro}$ [eV][d]
**1a**	-2.03	-2.35	0.87	1.27	0.85	-5.23	2.88
**1b**	-2.00	-2.38	0.92	—	0.92	-5.30	2.92
**^{t}Bu-HBC**	—	—	1.06	1.54	0.98	-5.36	—

[a] The half-wave potential (V vs SCE) measured in CH_2Cl_2 solution (2×10^{-3} M) by DPV at a scan rate of 0.1 V/s using ferrocene as an internal standard taking $E_{1/2}$ (Fc/Fc^+) = 0.424 V vs SCE.[2] [b] LUMO estimated by the approximate onset of the first reduction peaks and calculated according to $E_{LUMO} = -(4.38 + E_{1/2}^{re})$ eV. [c] HOMO estimated by the onset of the first oxidation peaks and calculated according to $E_{HOMO} = -(4.38 + E_{onset}^{ox})$ eV. [d] Calculated according to $E_g^{electro} = (E_{LUMO} - E_{HOMO})$ eV.

5. Thermogravimetric Analysis (TGA)

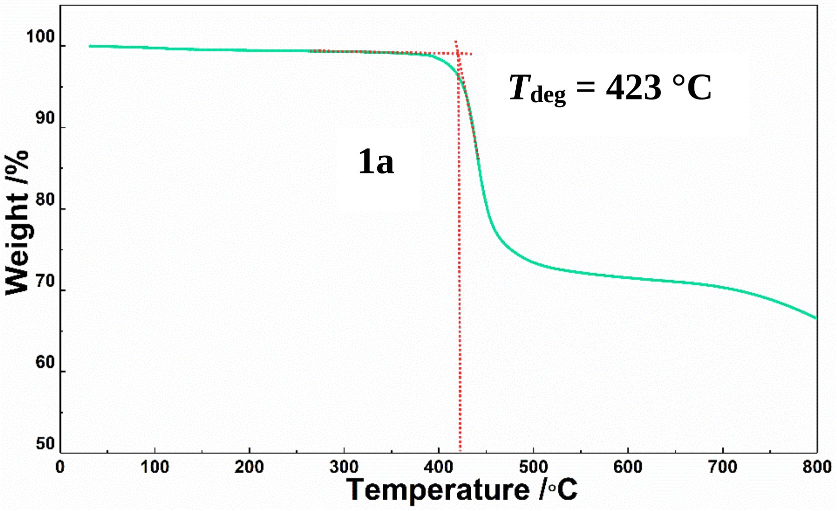

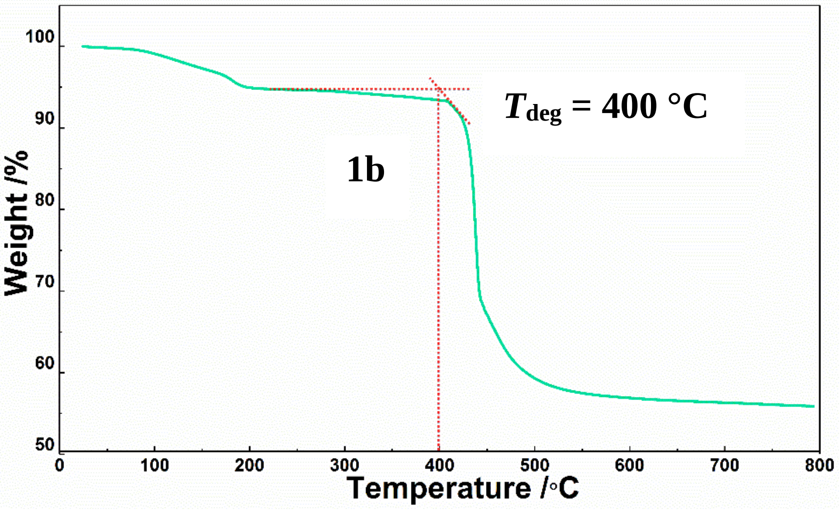

Supplementary Figure 24. TGA curves of compounds **1a** and **1b** under nitrogen flow.

6. Association with Fullerenes

Single crystals of compound **1a-Me@C$_{60}$** was grown by diffusing ethanol into its solution in *o*-dichlorobenzene, **1a-Me@C$_{70}$** was grown by diffusing ethanol into its solution in toluene.

6.1 X-ray Crystallography for (1a-Me@C$_{60}$)

Supplementary Table 5. Crystallographic data and structure refinement details for **1a-Me@C$_{60}$**

Empirical formula	$C_{111}H_{24}S_3{\cdot}(C_6H_4Cl_2)_2$
Formula weight	1747.46
Temperature/K	152.00
Crystal system	triclinic
Space group	P-1
a/Å	13.2216(10)
b/Å	13.4167(10)
c/Å	21.2250(16)
α/°	92.016(3)
β/°	97.445(3)
γ/°	109.711(3)
Volume/Å^3	3502.0(5)
Z	2
ρ_{calc}g/cm^3	1.657
μ/mm^{-1}	2.910
F(000)	1772.0
Crystal size/mm^3	0.4 × 0.3 × 0.2
Radiation	CuKα (λ = 1.54178)
2Θ range for data collection/°	4.214 to 136.566
Index ranges	$-15 \leq h \leq 15, -16 \leq k \leq 14, -25 \leq l \leq 25$
Reflections collected	104498
Independent reflections	12460 [R_{int} = 0.0550, R_{sigma} = 0.0254]
Data/restraints/parameters	12460/0/1177
Goodness-of-fit on F^2	1.072
Final R indexes [I>=2σ (I)]	R_1 = 0.0500, wR_2 = 0.1134
Final R indexes [all data]	R_1 = 0.0557, wR_2 = 0.1166
Largest diff. peak/hole / e Å^{-3}	1.34/-1.44

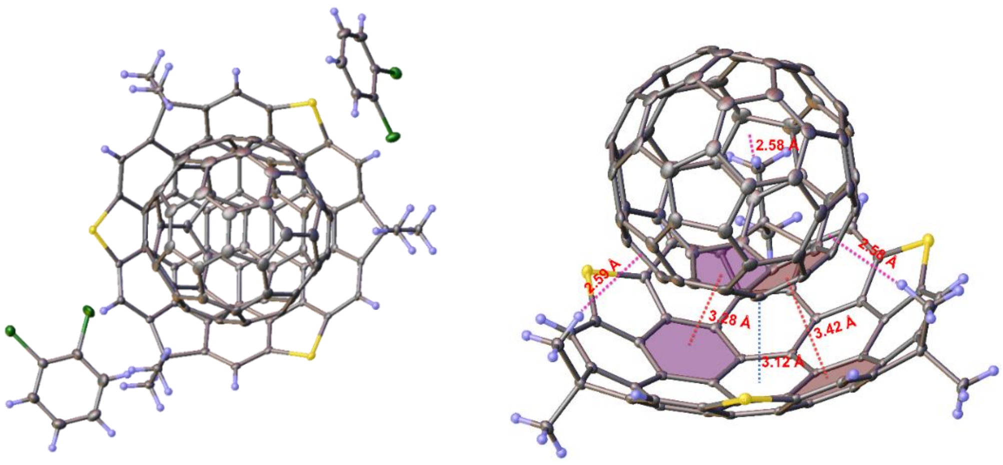

Supplementary Figure 25. Crystal structure of **1a-Me@C_{60}**: top view and side view. Thermal ellipsoids are shown at 30% probability. There are C-H···π interactions and π···π interactions between **1a-Me** and C_{60}.

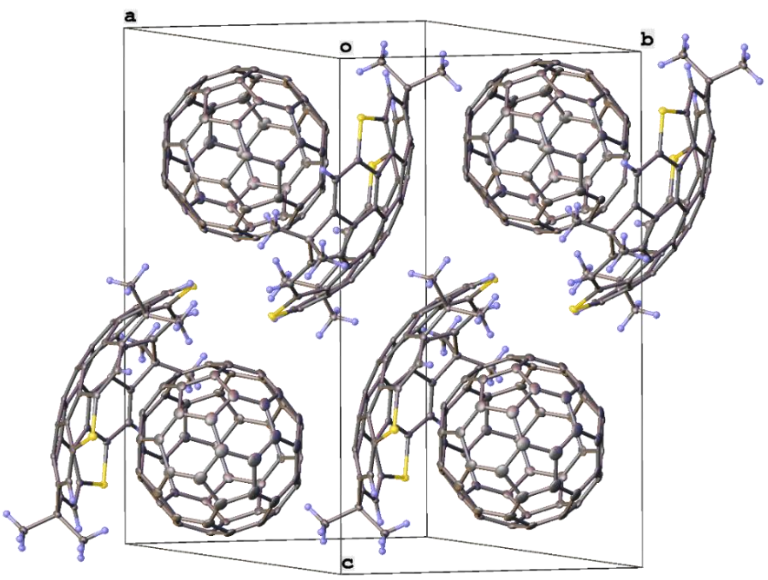

Supplementary Figure 26. One type of crystallographic independent molecules of **1a-Me@C_{60}**. There are two complete complex molecules in one unit cell. Thermal ellipsoids are shown at 30% probability. Solvent molecules are omitted for clarity.

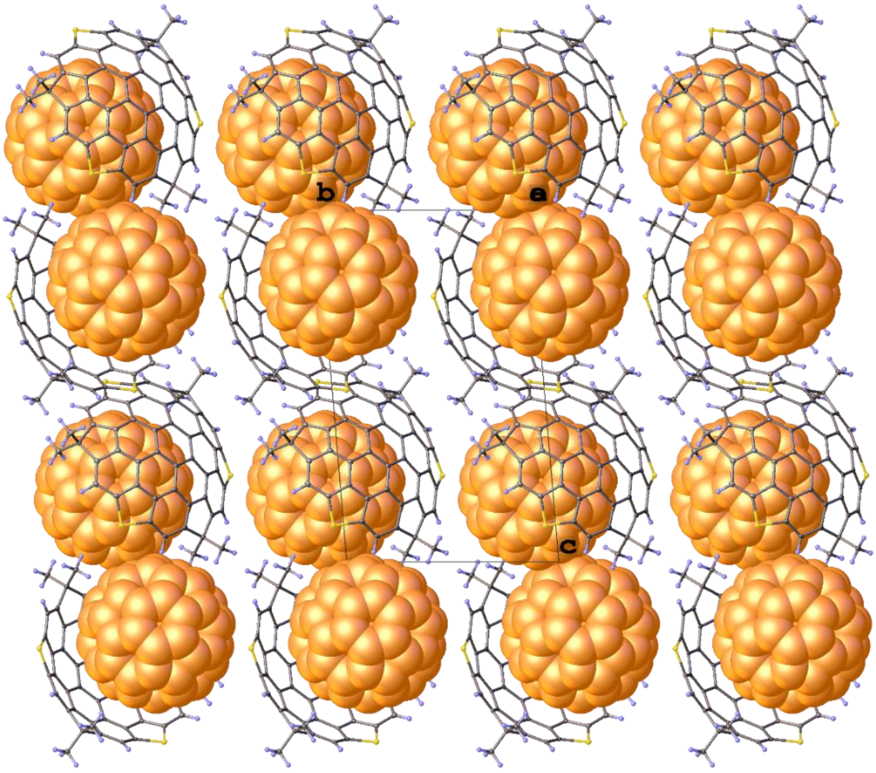

Supplementary Figure 27. View along a-axis.

6.2 X-ray Crystallography for (1a-Me@C_{70})

Supplementary Table 6. Crystallographic data and structure refinement details for **1a-Me@C_{70}**

Empirical formula	$C_{121}S_3H_{24}$
Formula weight	1573.58
Temperature/K	293(2)
Crystal system	monoclinic
Space group	$P2_1/c$
a/Å	20.312(4)
b/Å	33.234(9)
c/Å	27.770(6)
α/°	90
β/°	127.915(13)
γ/°	90
Volume/Å^3	14789(6)
Z	8
ρ_{calc}g/cm^3	1.413
μ/mm^{-1}	1.396
F(000)	6384.0
Crystal size/mm^3	0.2 × 0.15 × 0.13
Radiation	CuKα (λ = 1.54178)
2Θ range for data collection/°	4.83 to 132.098
Index ranges	$-24 \leq h \leq 18$, $0 \leq k \leq 39$, $0 \leq l \leq 32$
Reflections collected	25643
Independent reflections	25643 [R_{int} = ?, R_{sigma} = 0.0899]
Data/restraints/parameters	25643/11851/1833
Goodness-of-fit on F^2	1.039
Final R indexes [I>=2σ (I)]	R_1 = 0.1505, wR_2 = 0.2966
Final R indexes [all data]	R_1 = 0.1907, wR_2 = 0.3150
Largest diff. peak/hole / e Å^{-3}	1.15/-0.52

a) b)

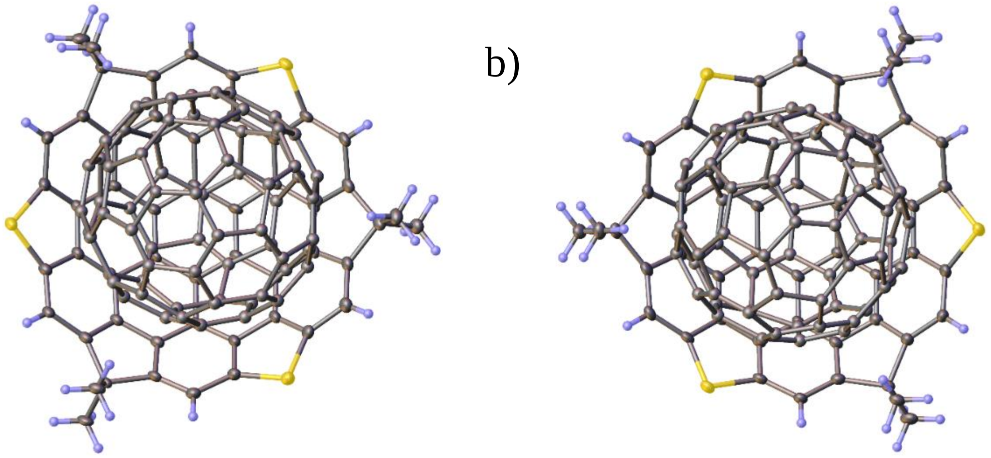

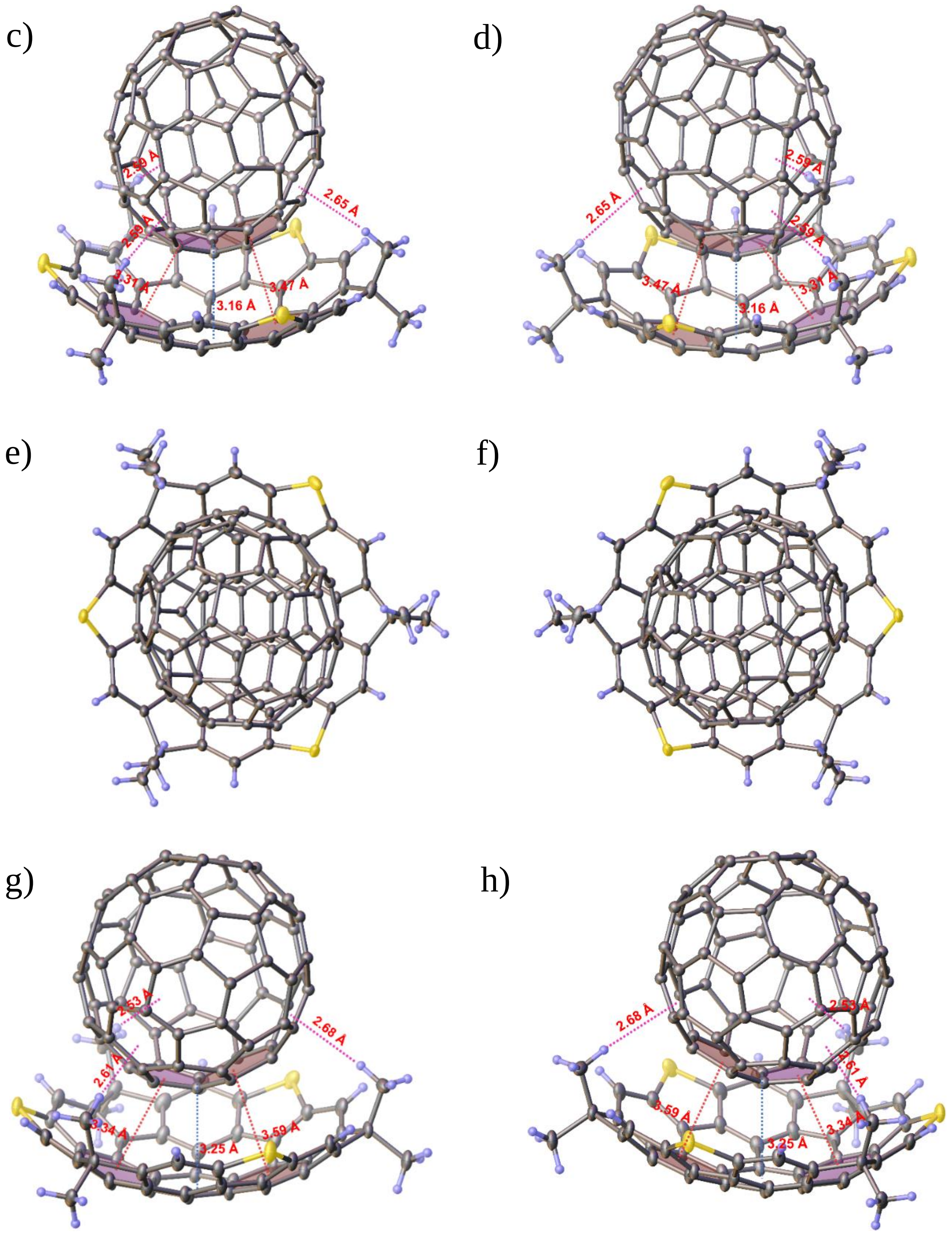

Supplementary Figure 28. Crystal structure of **1a-Me@C$_{70}$**: top view and side view. Thermal ellipsoids are shown at 20% probability. There are two association modes as racemic mixtures including upright (a-d) and latericumbent (e-h) conformation of C$_{70}$ with C-H···π interactions and π···π interactions between **1a-Me** and C$_{70}$.

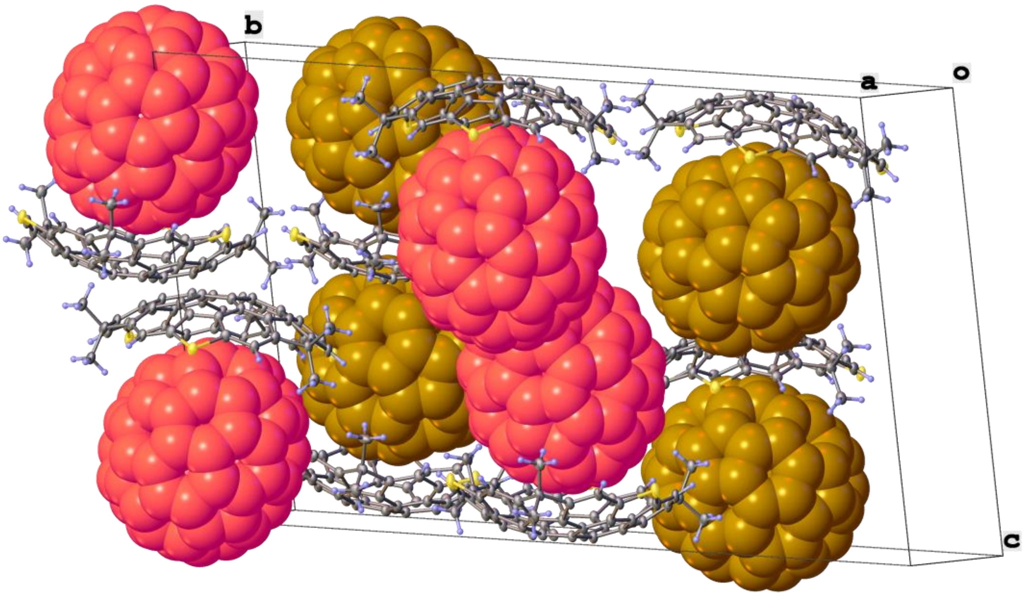

Supplementary Figure 29. Two types of crystallographic independent molecules of **1a-Me@C_{70}**. There are eight complete complex molecules in one unit cell. Thermal ellipsoids are shown at 20% probability.

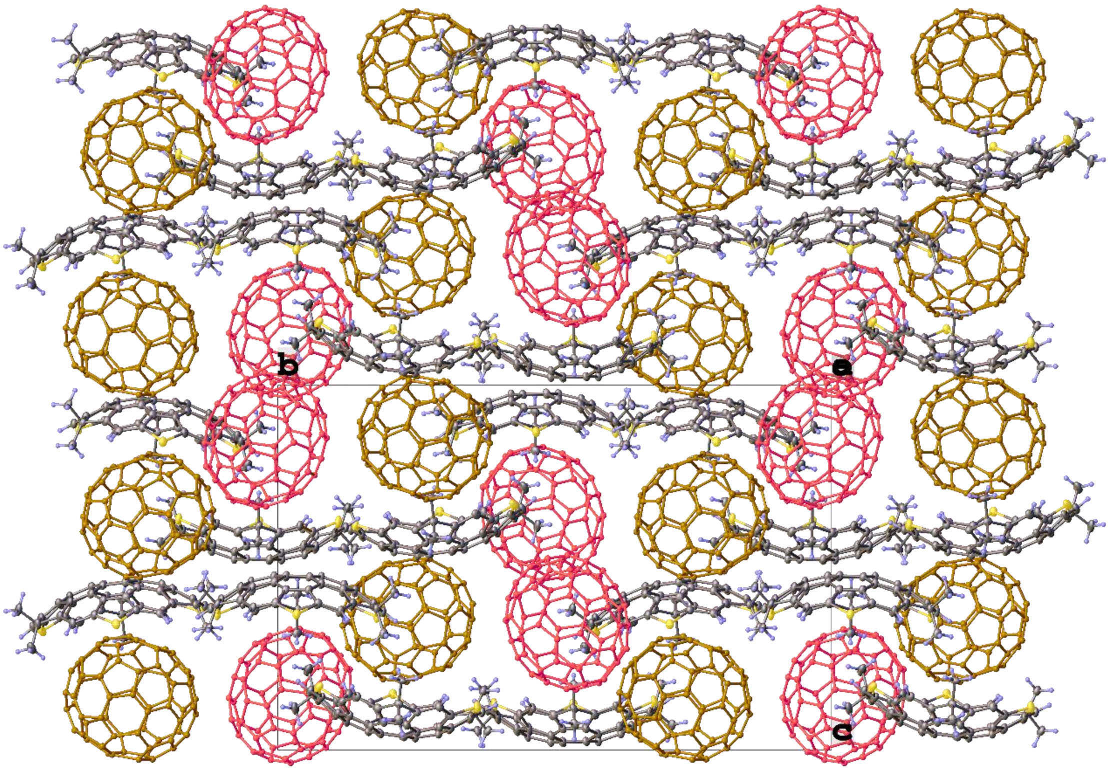

Supplementary Figure 30. View along a-axis.

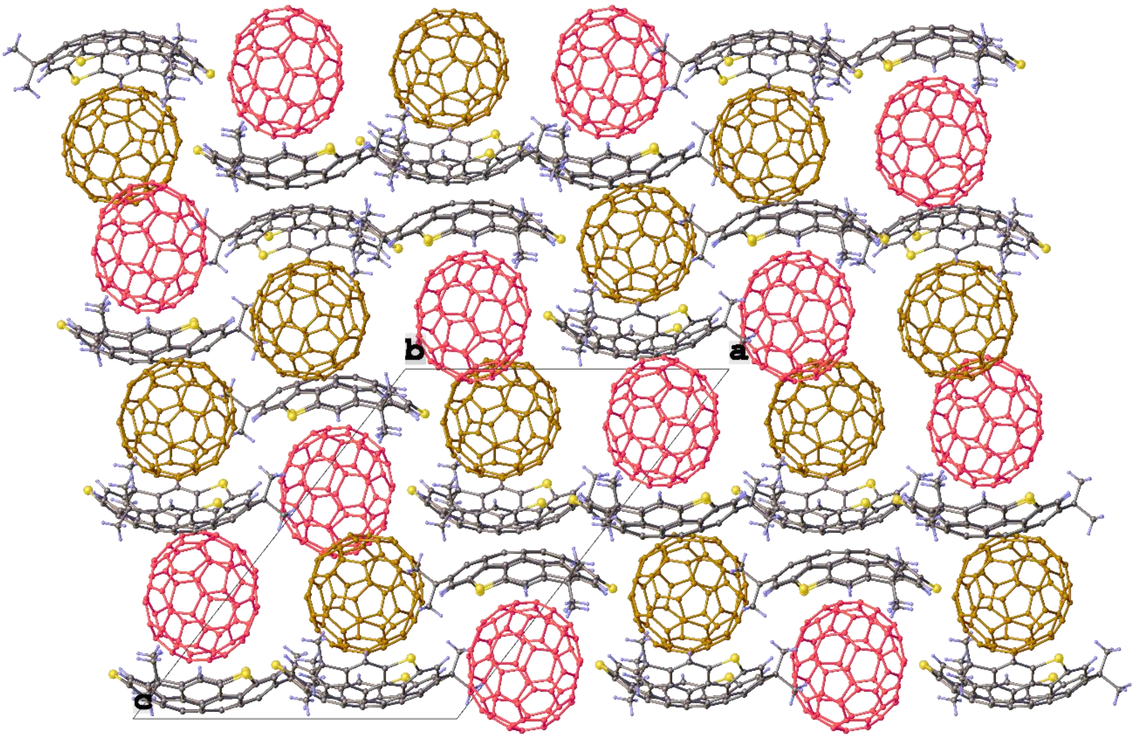

Supplementary Figure 31. View along b-axis.

6.3 Job plot analyses

A solution of fullerene (C_{60} or C_{70}) in *o*-DCB (1.2×10^{-5} M) and a solution of **1a-Me** in *o*-DCB (1.2×10^{-5} M) were mixed in different ratio to prepare 11 samples (Supplementary Figure 32 and Figure 33), while the sum of the concentrations of fullerene and **1a-Me** was kept constant at 1.2×10^{-5} M for UV/Vis spectra.

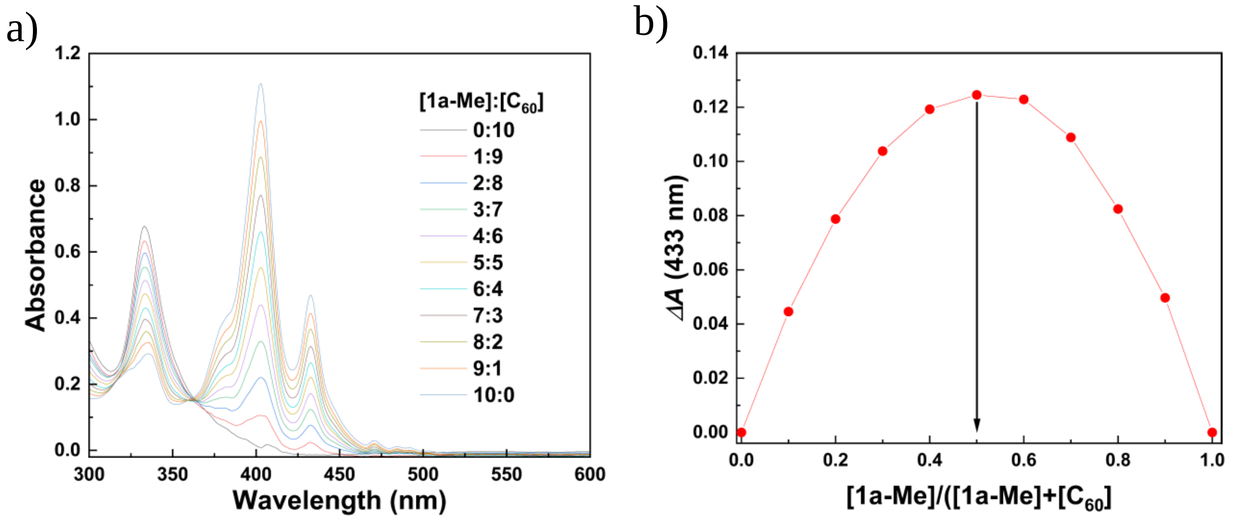

Supplementary Figure 32. a) UV/Vis absorption spectra of **1a-Me** and C_{60} mixed in different ratio in *o*-DCB at 298 K. b) Job's plot based on the absorption change at 433 nm, indicating a 1:1 binding stoichiometry between **1a-Me** and C_{60} in *o*-DCB.

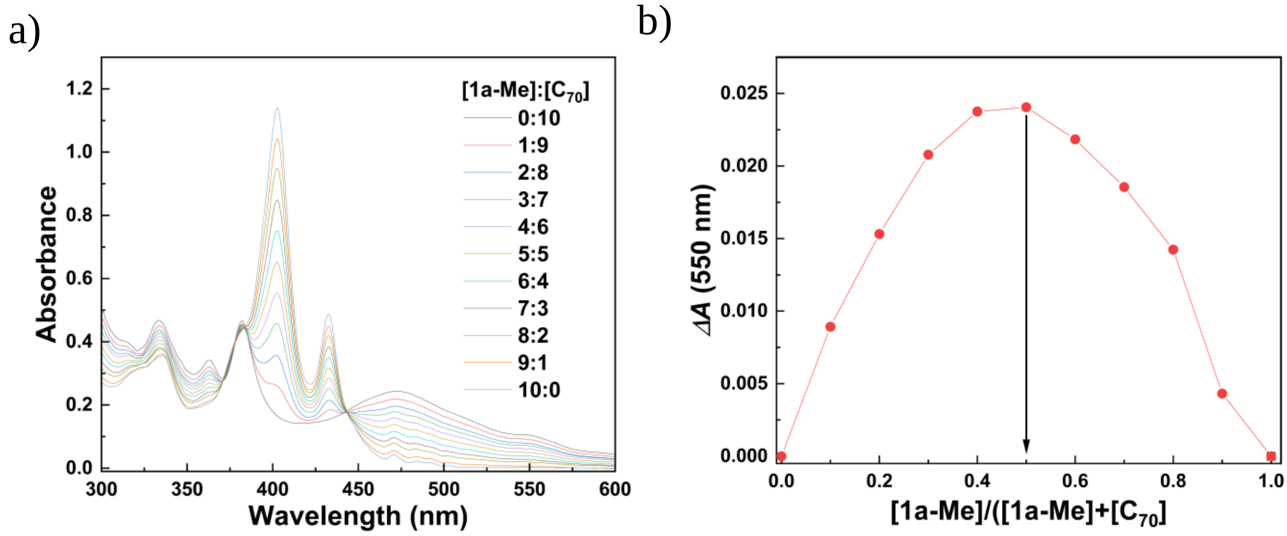

Supplementary Figure 33. a) UV/Vis absorption spectra of **1a-Me** and C_{70} mixed in different ratio in *o*-DCB at 298 K. b) Job's plot based on the absorption change at 550 nm, indicating a 1:1 binding stoichiometry between **1a-Me** and C_{70} in *o*-DCB.

6.4 Determination of binding constant

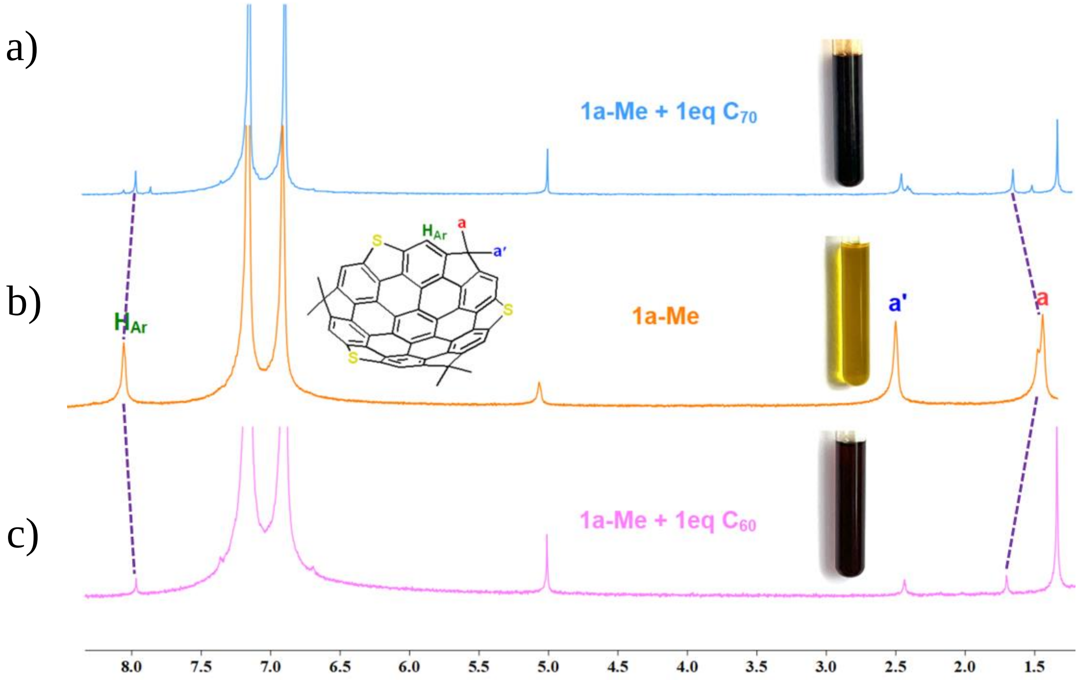

Supplementary Figure 34. Truncated ^{1}H NMR spectra (400 MHz, 1,2-dichlorobenzene-d_4, 298 K) of (a) **1a-Me** with 1.0 equiv. of C_{70}, (b) free **1a-Me** and (c) **1a-Me** with 1.0 equiv. of C_{60}, $[1a\text{-}Me]_0$ = 5 mM.

Because the association constant could not be determined by NMR titration due the low solubility of complexes, we used fluorescence titration for quantitative analysis. To determine the association constant (K_a) of **1a-Me** with C_{60} and C_{70}, fluorescence spectral titration analysis was carried out in 1,2-diclorobenzene. Stock solution A and B were prepared as shown in Supplementary Table 7. Titration was performed by successive addition of solution B into 2.0 mL of solution A. Excitation wavelength of 407 nm was used for emission spectra of **1a-Me**. Due to the competition absorption of C_{60} and C_{70} at the excitation and emission wavelength of **1a-Me**, the fluorescence intensity F_{exp} was calibrated to F_{cal} according to a well-established method.[10]

$$\boldsymbol{F_{cal} = F_{exp} \times \frac{1 - e^{-\varepsilon_1 c_1 l}}{\varepsilon_1 c_1 l} \times \frac{\varepsilon_1 c_1 l + \varepsilon_2 c_2 l}{1 - e^{(-\varepsilon_1 c_1 l - \varepsilon_2 c_2 l)}} \times \frac{\varepsilon_3 c_3 l}{1 - e^{-\varepsilon_3 c_3 l}}} \qquad \mathbf{(1)}$$

where F_{exp} was the experimental fluorescence intensity; F_{cal} was the fluorescence intensity after calibration; $\varepsilon_1 c_1 l$ and $\varepsilon_2 c_2 l$ were the absorbance of the host **1a-Me** and fullerene guest at excitation wavelength (λ_{ex}=407nm), respectively, while $\varepsilon_3 c_3 l$ was the absorbance of fullerene guest at emission wavelength of host **1a-Me** (λ_{em} =514nm).

On the basis of the 1:1 complex model, association constant K_a is calculated by non-linear curve fitting (1stopt software with universal global optimation) using the following equation:

$$\frac{F_0}{F_{cal}} = \frac{2}{1 - \frac{1}{[H]_0}\left([G]_0 + \frac{1}{K_a} - \sqrt{\left([H]_0 + [G]_0 + \frac{1}{K_a}\right)^2 - 4[H]_0[G]_0}\right)} \qquad (2)$$

where F_0, F_{cal}, H_0, G_0, and K_a are fluorescence intensity of the **1a-Me** before the addition of C_{60} or C_{70}, fluorescence intensity after calibration, initial concentration of **1a-Me**, initial concentration of C_{60} or C_{70}, and the binding constant respectively.[11]

Supplementary Table 7. Concentrations of stock solutions for each titration experiment in 1,2-diclorobenzene.

Host molecule	C_{60}			C_{70}		
	Solution **A**	Solution **B**		Solution **A**	Solution **B**	
	[host] μM	[host] μM	[C_{60}] μM	[host] μM	[host] μM	[host] μM
**1a-Me**	6	6	120	6	6	120

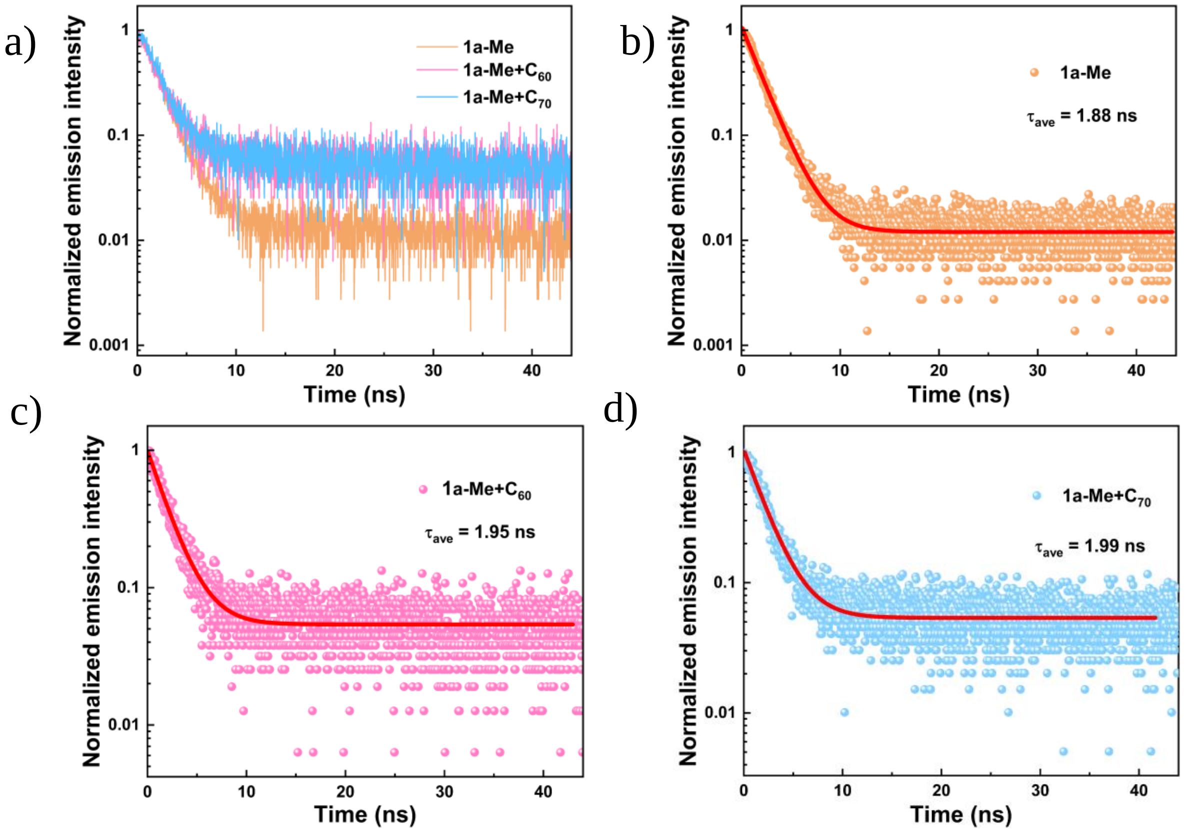

Supplementary Figure 35. a) Normalized photoluminescence decay (λ_{exc}= 407 nm) of **1a-Me** in *o*-DCB (6×10^{-6} M) without fullerene and mixed with 1:1 ratio of of C_{60} or C_{70} (6×10^{-6} M). b, c, d) The respective fluorescence lifetime of **1a-Me**, **1a-Me+C_{60}**, **1a-Me+C_{70}**.

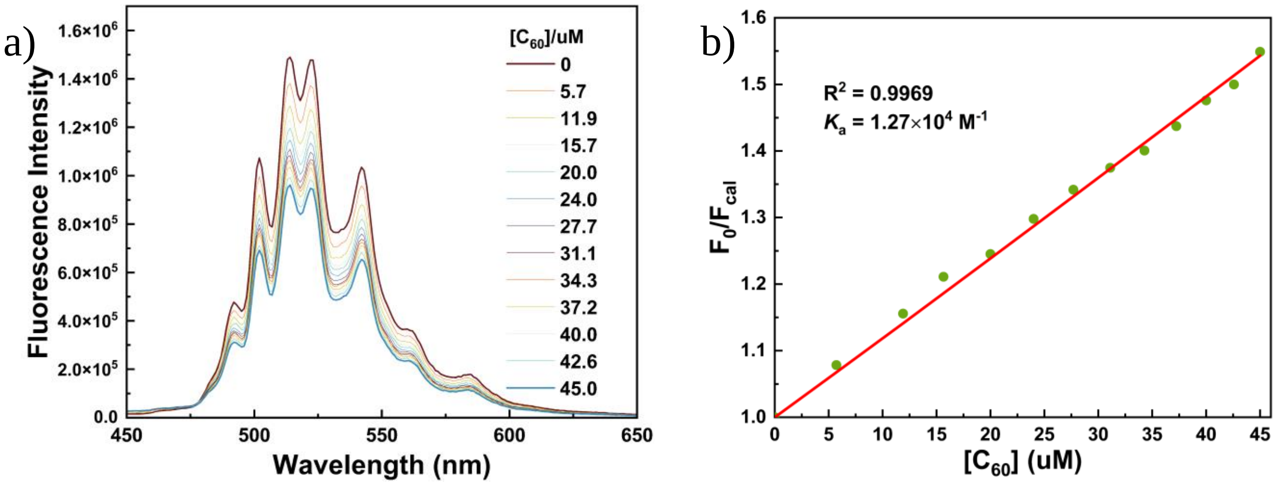

Supplementary Figure 36. a) Fluorescence spectrum of **1a-Me** upon addition of 0–45.0 µM of C_{60}, while the concentrations of **1a-Me** was kept constant at 6 µM. b) Nonlinear curve regression for a titration experiment of **1a-Me** with C_{60} using 1:1 binding model. Based on this data set, K_a is calculated to be 1.27 $\times 10^4$ M^{-1}.

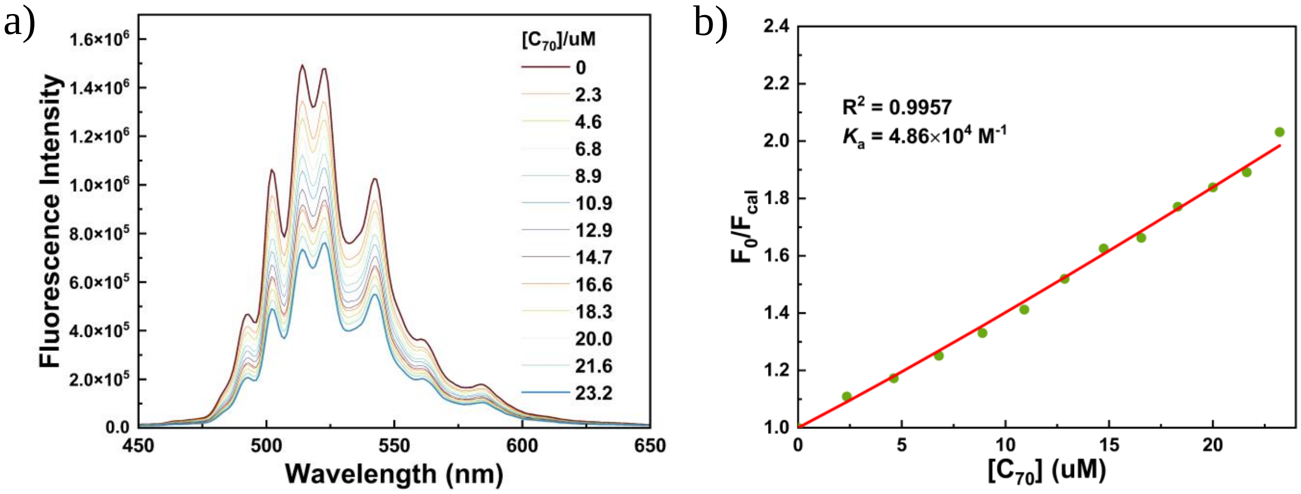

Supplementary Figure 37. a) Fluorescence spectrum of **1a-Me** upon addition of 0–23.2 µM of C_{70}, while the concentrations of **1a-Me** was kept constant at 6 µM. b) Nonlinear curve regression for a titration experiment of **1a-Me** with C_{70} using 1:1 binding model. Based on this data set, K_a is calculated to be 4.86 $\times 10^4$ M^{-1}.

7. DFT calculations

Quantum chemical computations were performed for **1a** and **1b** with all butyls replaced by hydrogen atoms (**1a'** and **1b'**). This only has a marginal influence on the electronic properties of the systems, but significantly speeds up computational explorations. Geometric optimization was performed at B3LYP/6-311G(d,p) level of theory using the Gaussian 09, Revision D.01 program,[3] and some calculated results were processed by Multiwfn.[12] Electrostatic potential[13] calculations were conducted at B3LYP/6-311G(d,p) level. NICS(1)$_{zz}$[14] were calculated using the gauge invariant atomic orbital (GIAO) approach at the GIAO-B3LYP/6-311G(d,p) level. Due to the bowl-shape structure, we used the average of NICS(1)$_{zz}$ (cocave) and NICS(-1)$_{zz}$ (convex) to evaluate the aromaticity character of each individual rings. AICD plot[15] was calculated by using the method developed by Herges at B3LYP/6-311G(d,p) level. TD-DFT calculations were conducted at PBE/def2tzvp level. Inversion barrier and dipole moment calculations were conducted at M062x/6-31G(d) level.

7.1 Optimized Structures and Bowl to Bowl Inversion Energy

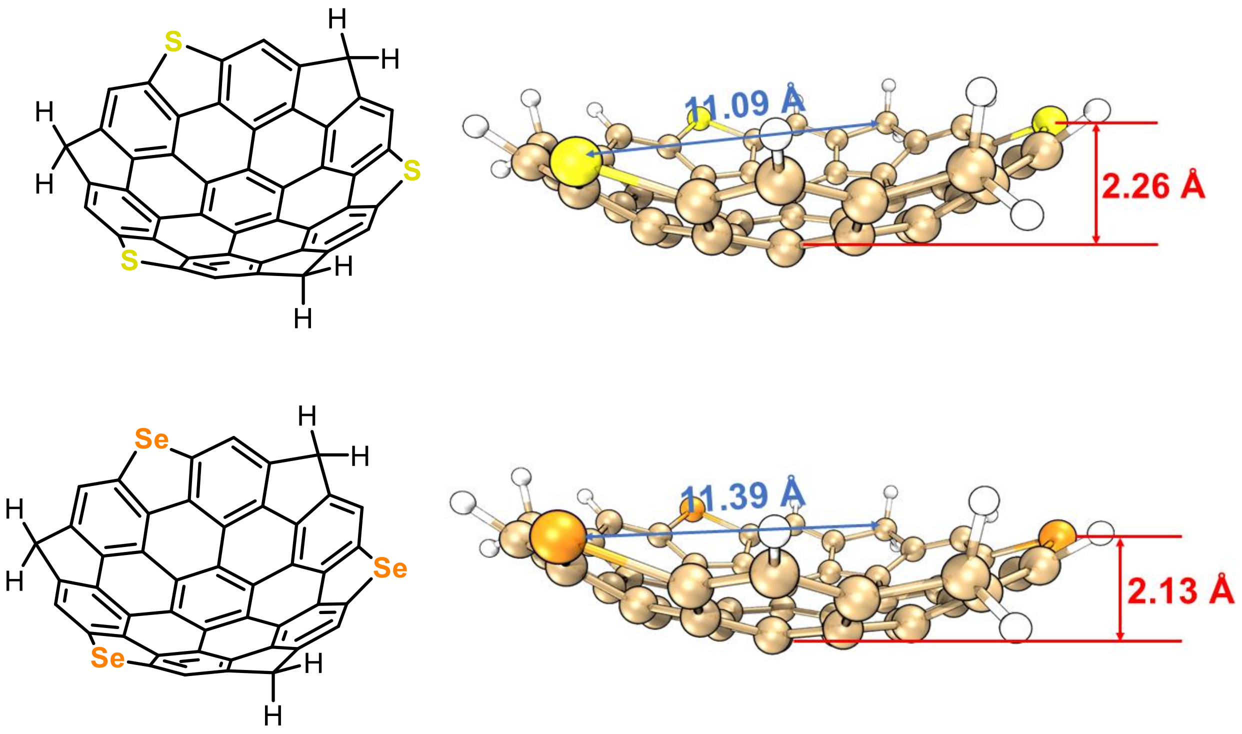

Supplementary Figure 38. Calculated geometry of **1a'** and **1b'**.

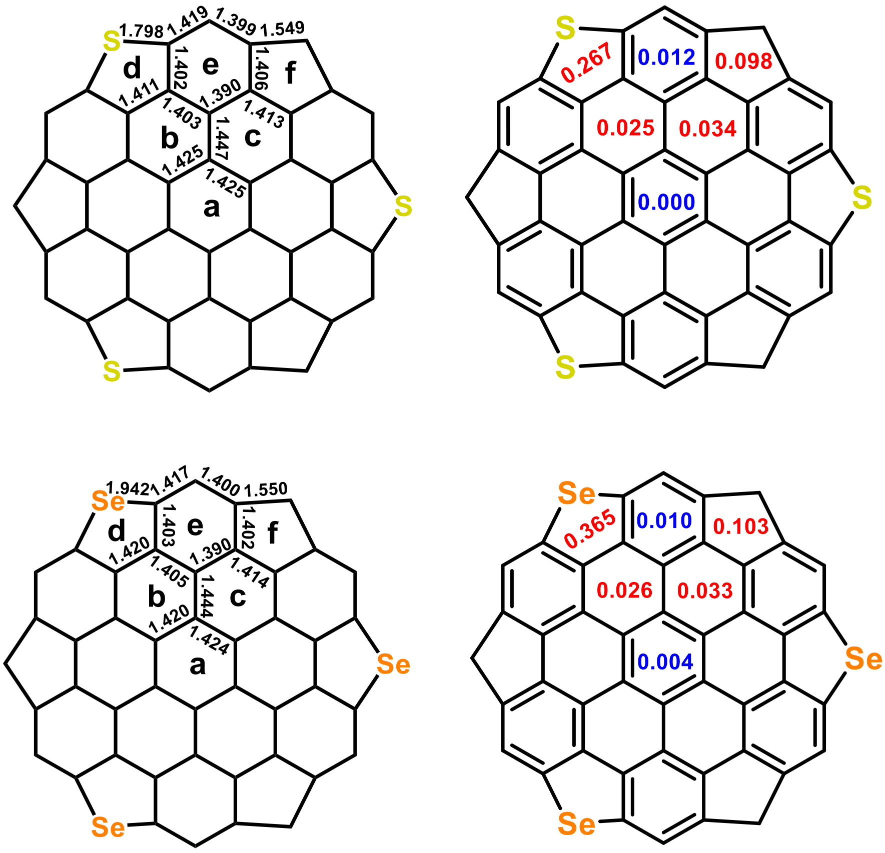

Supplementary Figure 39. Calculated bond lengths and bond length alternation (BLA) of **1a'** and **1b'**.

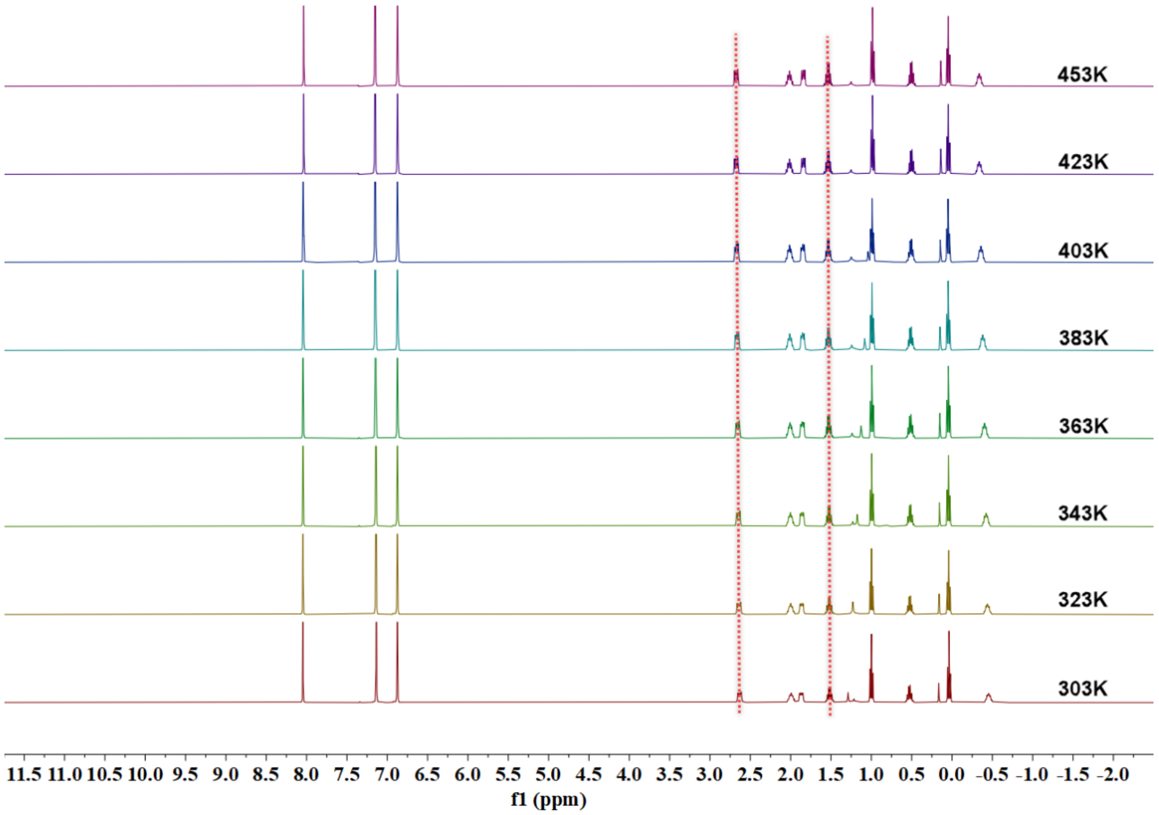

Supplementary Figure 40. Variable-temperature ^{1}H NMR (600MHz) spectra of **1a** in 1,2-dichlorobenzene-*d*$_4$ recorded from 303K to 453K.

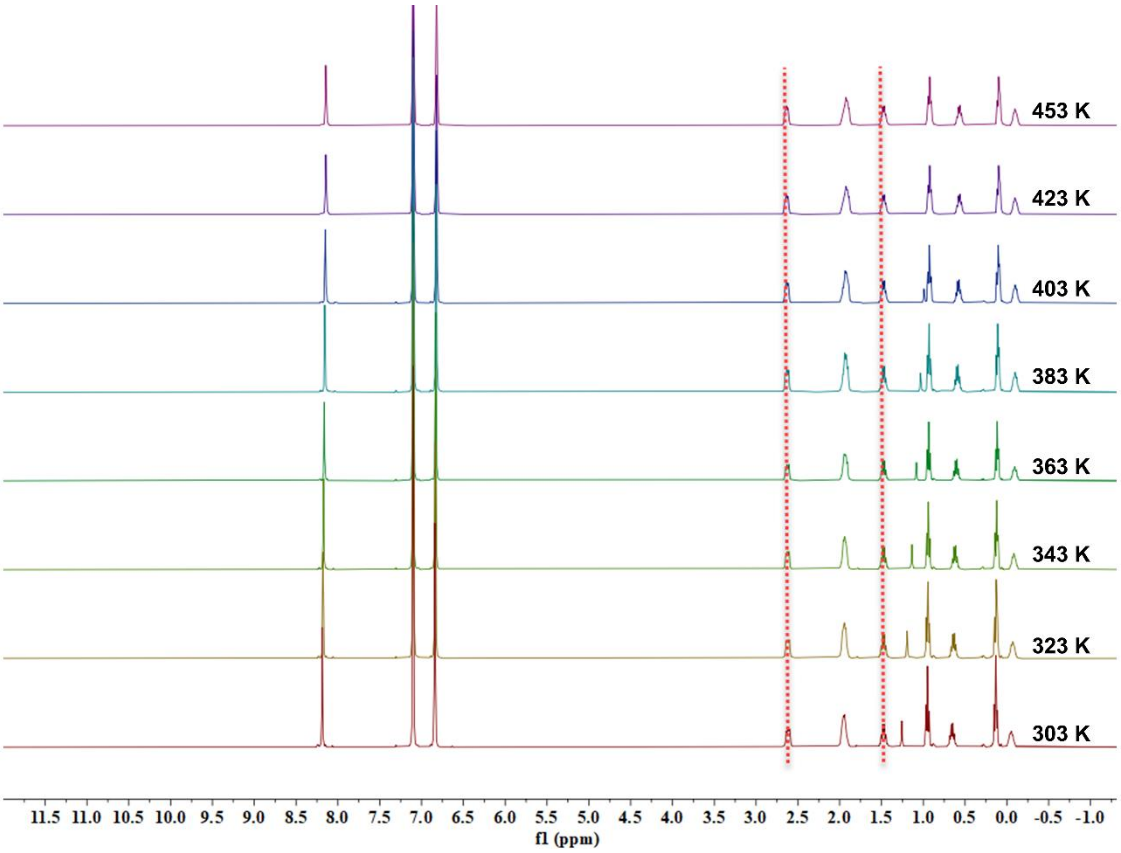

Supplementary Figure 41. Variable-temperature ^{1}H NMR (600MHz) spectra of **1b** in 1,2-dichlorobenzene-d_4 recorded from 303K to 453K.

Supplementary Table 8. Calculated total energies (M062x/6-31G(d)) for **1a'**.

Parameter	Ground state (Hartree/Particle)	Transition state (Hartree/Particle)
Zero-point correction	0.421780	0.417934
Thermal correction to Energy	0.448199	0.445421
Thermal correction to Enthalpy	0.449143	0.446366
**Thermal correction to Gibbs Free Energy**	**0.370492**	**0.364923**
Sum of electronic and zero-point Energies	-2915.602536	-2915.488794
Sum of electronic and thermal Energies	-2915.576117	-2915.461307
Sum of electronic and thermal Enthalpies	-2915.575173	-2915.460362
Sum of electronic and thermal Free Energies	-2915.653824	-2915.541805
**Single point energy**	**-2916.024292**	**-2915.906839**

Supplementary Table 9. Calculated total energies (M062x/6-31G(d)) for **1b'**.

Parameter	Ground state (Hartree/Particle)	Transition state (Hartree/Particle)
Zero-point correction	0.420988	0.423061
Thermal correction to Energy	0.448355	0.450249
Thermal correction to Enthalpy	0.449299	0.451193
**Thermal correction to Gibbs Free Energy**	**0.367163**	**0.369073**
Sum of electronic and zero-point Energies	-8919.431355	-8919.349402
Sum of electronic and thermal Energies	-8919.403988	-8919.322214
Sum of electronic and thermal Enthalpies	-8919.403044	-8919.321270
Sum of electronic and thermal Free Energies	-8919.485180	-8919.403390
**Single point energy**	**-8919.852537**	**-8919.773099**

Supplementary Table 10. Calculated inversion barrier in **1a'** and **1b'**.

Compd	Ground state (*GS*) (Hartree/Particle)	Transition state (*TS*) (Hartree/Particle)	Δ (*TS* - *GS*) (Hartree)	Δ (*TS* - *GS*) (kcal mol^{-1})
**1a'**	-2,915.653800	-2,915.541916	0.111884	70.2
**1b'**	-8,919.485374	-8,919.404026	0.081348	51.0

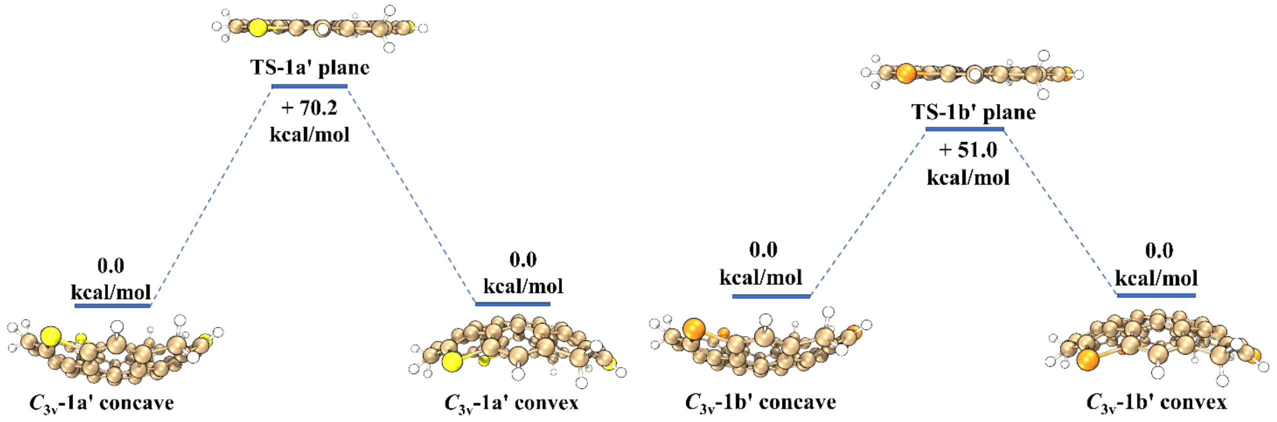

Supplementary Figure 42. DFT calculated geometry of **1a'**, **1b'** and the transition state **TS-1a'**, **TS-1b'** and the energy profile (M062x/6-31G(d)).

Bowl inversion kinetic equation

$$A \underset{k_{-1}}{\overset{k_1}{\rightleftharpoons}} A^*$$

t=0	$c_{A,0}$	0
t=t	$c_{A,0}-x$	x
t=∞	$c_{A,0}-x_e$	x_e

$$\frac{dx}{dt} = k_1 c_{A,0} - (k_1 + k_{-1})x$$

$$\Rightarrow \int_0^x \frac{dx}{k_1 c_{A,0} - (k_1 + k_{-1})x} = \int_0^t dt$$

$$\Rightarrow \ln c_{A,0} - \ln\left(c_{A,0} - \frac{k_1 + k_{-1}}{k_1}x\right) = (k_1 + k_{-1})t$$

$$\frac{dx}{dt} = 0 \Rightarrow k_1(c_{A,0} - x_e) = k_{-1}x_e$$

$$c_{A,0} = \frac{k_1 + k_{-1}}{k_1}x_e$$

⇒bowl inversion kinetic equation: $\ln\frac{x_e}{x_e - x} = (k_1 + k_{-1})t$

for bowl inversion, $k_1 = k_{-1} = k$

$$\Rightarrow \ln\frac{x_e}{x_e - x} = 2kt$$

where A the initial C_{3v} concave of **1a'** or **1b'**, and A* is C_{3v} convex of **1a'** or **1b'**.

When the reaction is 50% completed, $x = \frac{1}{2}x_e \Rightarrow t(50\%) = \frac{\ln 2}{2k}$

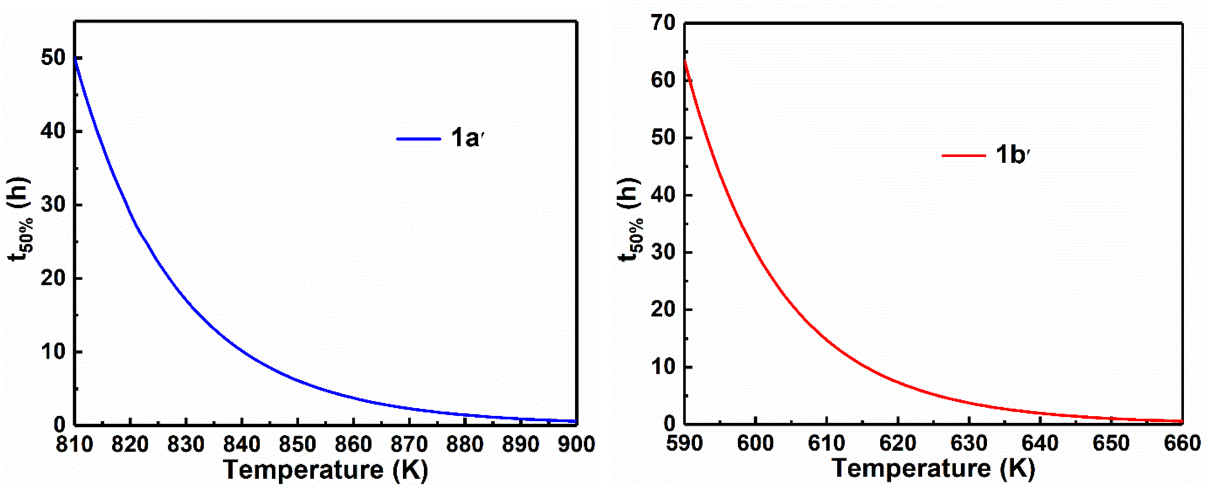

Supplementary Figure 43. DFT calculated geometry of ***C*₃v-1a'**, ***C*₃v-1b'** and the transition state **TS-1a'**, **TS-1b'** and the energy profile (M062x/6-31G(d)). The changing curve of t(50%) at various temperature was calculated according to Eyring equation: $k = \frac{k_B T}{h} e^{-(\Delta G/RT)}$, t(50%) =ln2/2k, where ΔG is the activation energy (kcal/mol), R is the gas constant 8.314 J· mol^{-1}· K^{-1}, T is the absolute temperature (K), K_B is the Boltzmann constant (1.38×10^{-23} J· K^{-1}), h is the Planck constant (6.626×10^{-34} J·S). The high energy barrier indicates that the inversion of ***C*₃v-1a'** and ***C*₃v-1b'** would be impossible at temperature below the thermal decomposition temperature in acceptable half-time.

7.2 Aromaticity Calculations

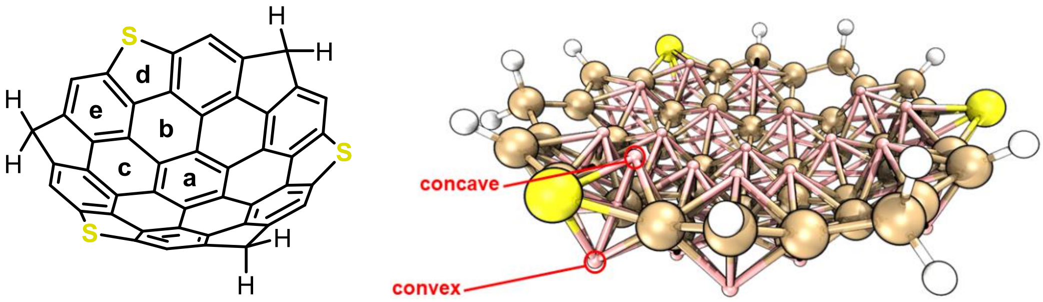

Supplementary Table 11. NICS calculation on **1a'** (GIAO-B3LYP/6-311G(d,p))

	NICS$_{iso}$			NICS$_{zz}$			
	NICS(0)	NICS(1)$_{convex}$	NICS(1)$_{concave}$	NICS(0)	NICS(1)$_{convex}$	NICS(1)$_{concave}$	NICS(1)$_{av}$
**a**	-12.29	-8.90	-18.58	-12.81	-24.38	-34.41	**-29.40**
**b**	1.84	1.19	-7.57	28.68	5.88	0.67	**3.28**
**c**	-2.36	-1.53	-10.84	16.62	-2.18	-9.68	**-5.93**
**d**	-7.57	-4.97	-10.14	15.07	-9.04	-11.33	**-10.19**
**e**	-11.10	-8.23	-16.07	-10.94	-21.59	-30.54	**-26.07**

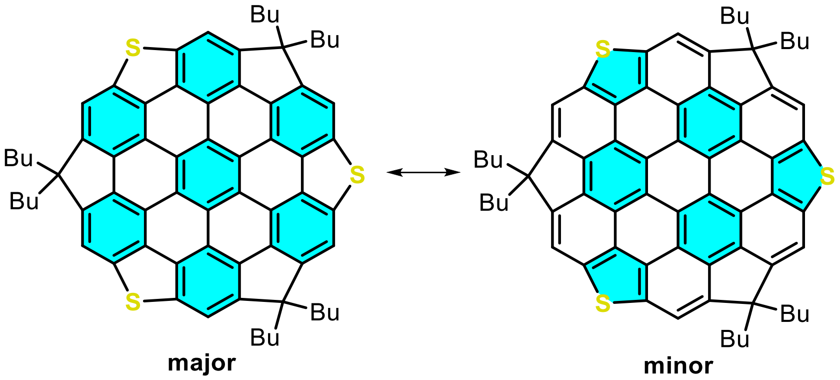

Supplementary Figure 44. Possible contributing structures in the resonance hybrid of **1a'**.

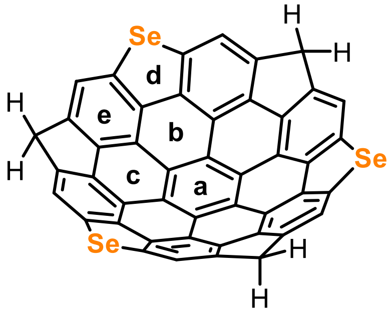

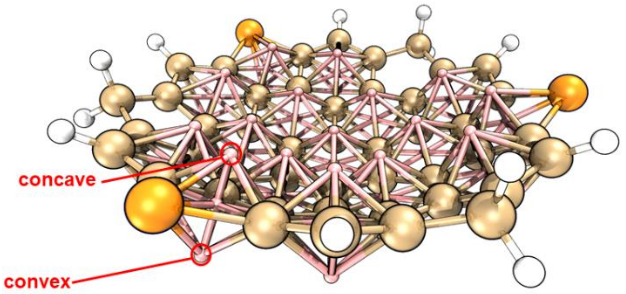

Supplementary Table 12. NICS calculation on **1b'** (GIAO-B3LYP/6-311G(d,p))

	$NICS_{iso}$			$NICS_{zz}$			
	NICS(0)	$NICS(1)_{convex}$	$NICS(1)_{concave}$	NICS(0)	$NICS(1)_{convex}$	$NICS(1)_{concave}$	$NICS(1)_{av}$
**a**	-12.33	-9.40	-18.35	-13.63	-25.79	-35.23	**-30.51**
**b**	1.86	0.84	-7.05	27.72	4.97	0.23	**2.60**
**c**	-1.64	-1.49	-9.87	18.15	-1.80	-8.30	**-5.05**
**d**	-6.07	-3.84	-8.24	20.59	-5.00	-7.22	**-6.11**
**e**	-10.73	-8.57	-15.49	-10.93	-22.47	-30.26	**-26.37**

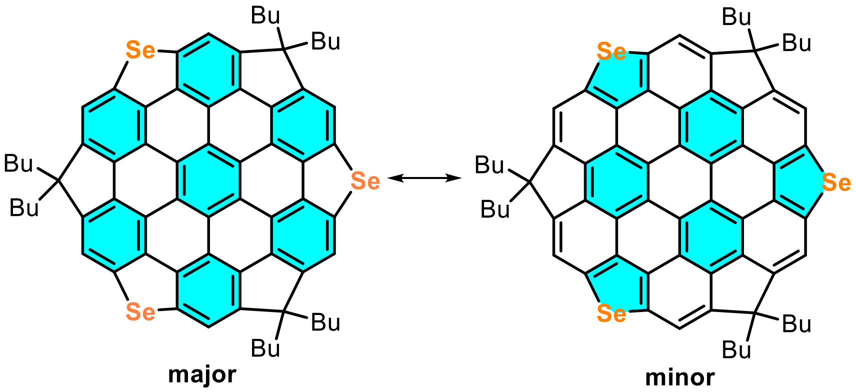

Supplementary Figure 45. Possible contributing structures in the resonancehybrid of **1b'**.

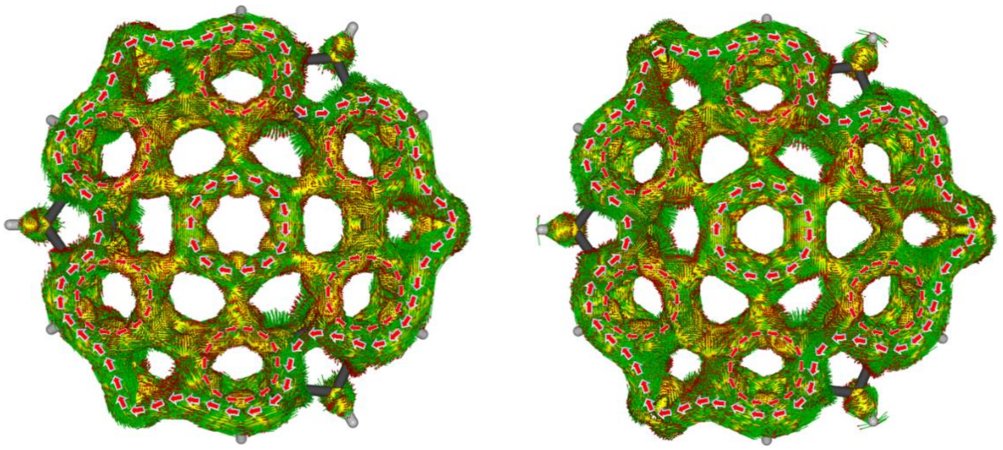

1a' **1b'**

Supplementary Figure 46. Calculated AICD (B3LYP/6-311G(d,p)) plots of **1a'** (left) and **1b'** (right) (isovalue = 0.03). Only contributions from π-electrons of the aromatic cores are considered. The magnetic field vector is perpendicular to the ring plane and points outward. Arrows indicate directions of induced ring current.

7.3 UV-Vis Absorption Spectra Calculation

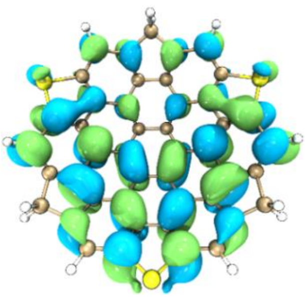

LUMO+7 (-0.85 eV)

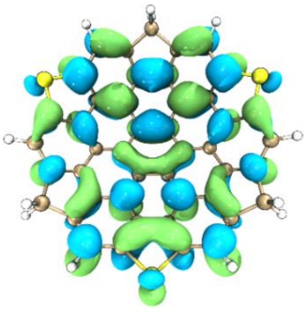

LUMO+6 (-0.85 eV)

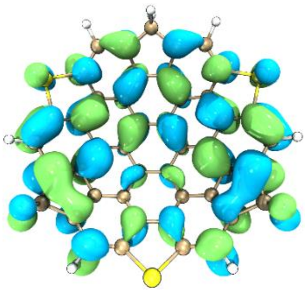

LUMO+5 (-0.95 eV)

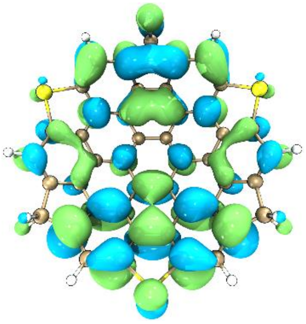

LUMO+4 (-0.95 eV)

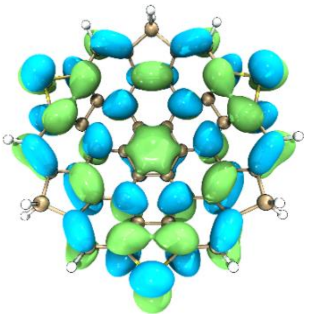

LUMO+3 (-1.27 eV)

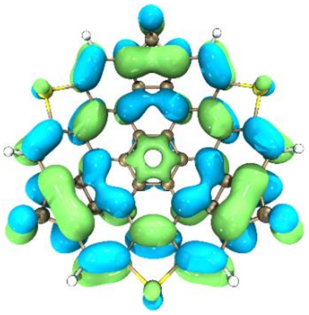

LUMO+2 (-1.56 eV)

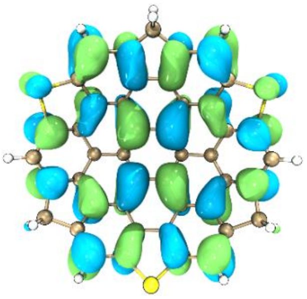

LUMO+1 (-2.01 eV)

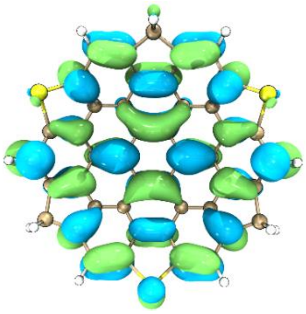

LUMO (-2.01 eV)

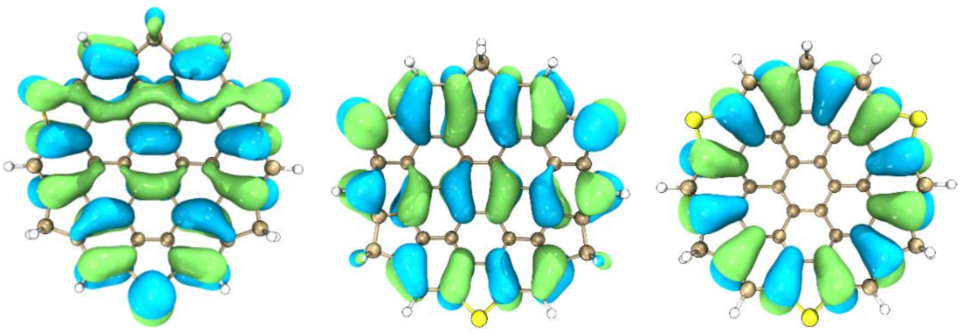

HOMO (-5.32 eV) | HOMO-1 (-5.32 eV) | HOMO-2 (-6.06 eV)

HOMO-LUMO Gap: 3.31 eV

Supplementary Figure 47. Kohn–Sham molecular orbitals (from HOMO-2 to LUMO+7) of **1a'** calculated at the B3LYP/6-311G(d,p) level of theory.

Supplementary Table 13. Major transitions corresponding to the UV spectrum of **1a'** calculated by TDDFT. (PBE/def2tzvp)

Excited state	Energy (eV)	Wavelength (nm)	Oscillator strength (f)	Description
S_1	2.7358	453.19	0.0005	HOMO→LUMO+1 (49.2%)<br>HOMO-1→LUMO (49.2%)
S_2	2.8216	439.41	0.0000	HOMO→LUMO (49.7%)<br>HOMO-1→LUMO+1(49.7%)
S_3	3.1953	388.02	0.4286	HOMO→LUMO+1 (39.1%)<br>HOMO-1→LUMO (39.1%)<br>HOMO-1→LUMO+2 (15.1%)
S_4	3.1953	388.02	0.4286	HOMO-1→LUMO+1 (39.1%)<br>HOMO→LUMO (39.1%)<br>HOMO→LUMO+2 (15.1%)
S_5	3.3245	372.94	0.0998	HOMO-1→LUMO+2 (77.5%)<br>HOMO→LUMO+1 (8.0%)<br>HOMO-1→LUMO (8.0%)
S_6	3.3245	372.94	0.0998	HOMO→LUMO+2 (77.5%)<br>HOMO-1→LUMO+1 (8.0%)<br>HOMO→LUMO (8.0%)
S_{15}	3.9697	312.33	0.1106	HOMO→LUMO+7 (37.0%)<br>HOMO-1→LUMO+6 (37.0%)<br>HOMO-1→LUMO+5 (6.9%)<br>HOMO→LUMO+4 (6.9%)

S_{16}	3.9697	312.33	0.1106	HOMO→LUMO+7 (37.0%)<br>HOMO-1→LUMO+6 (37.0%)<br>HOMO-1→LUMO+5 (6.9%)<br>HOMO→LUMO+4 (6.9%)

HOMO index: 165, LUMO index: 166

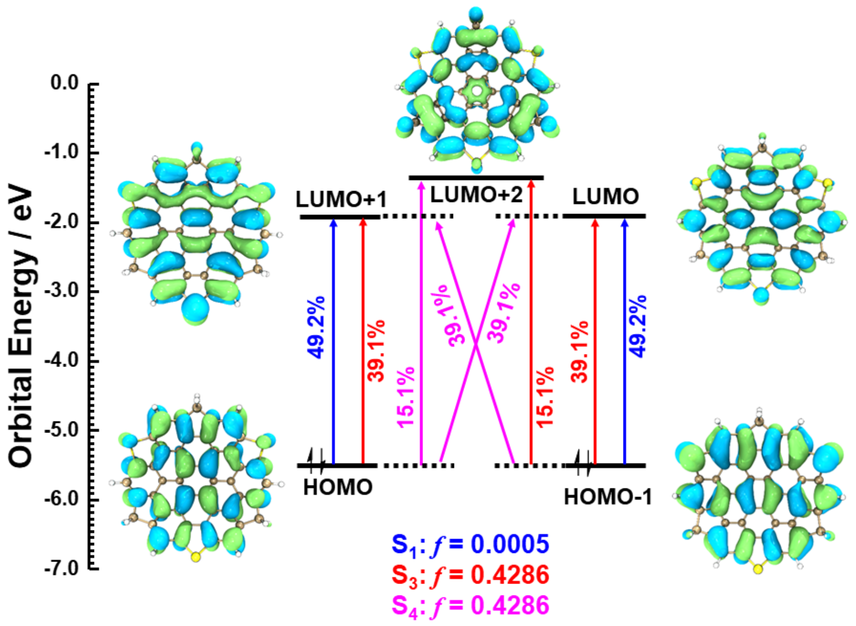

Supplementary Figure 48. Orbital correlation diagram and transition composition of the S_0→(S_1, S_3, S_4) excited states for **1a'** calculated at PBE/def2tzvp level of theory.

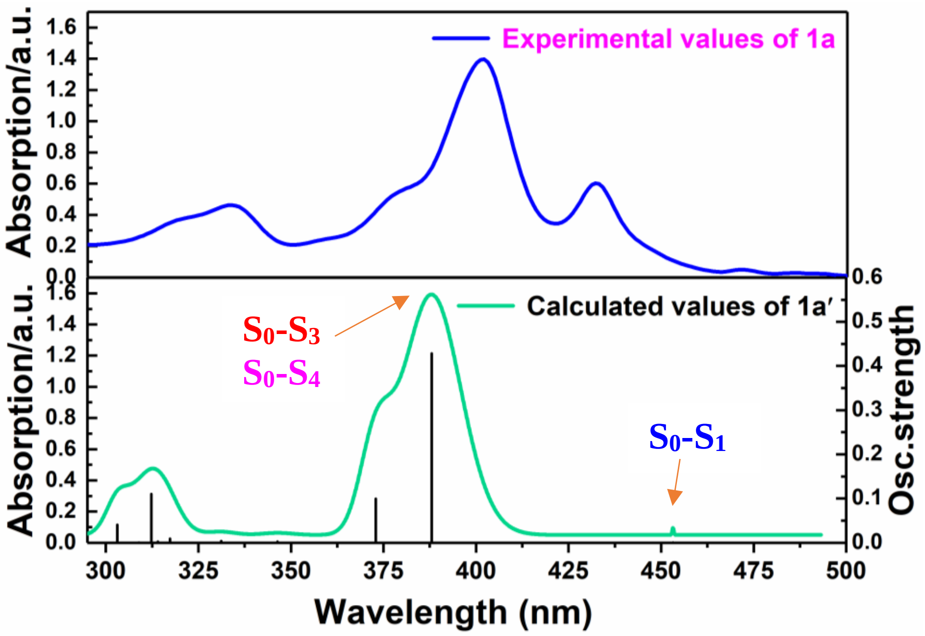

Supplementary Figure 49. The experimental (top) and calculated (bottom) UV-vis absorption spectra of **1a'**, and the main absorption spectra of different transition from state to excited states (bottom).

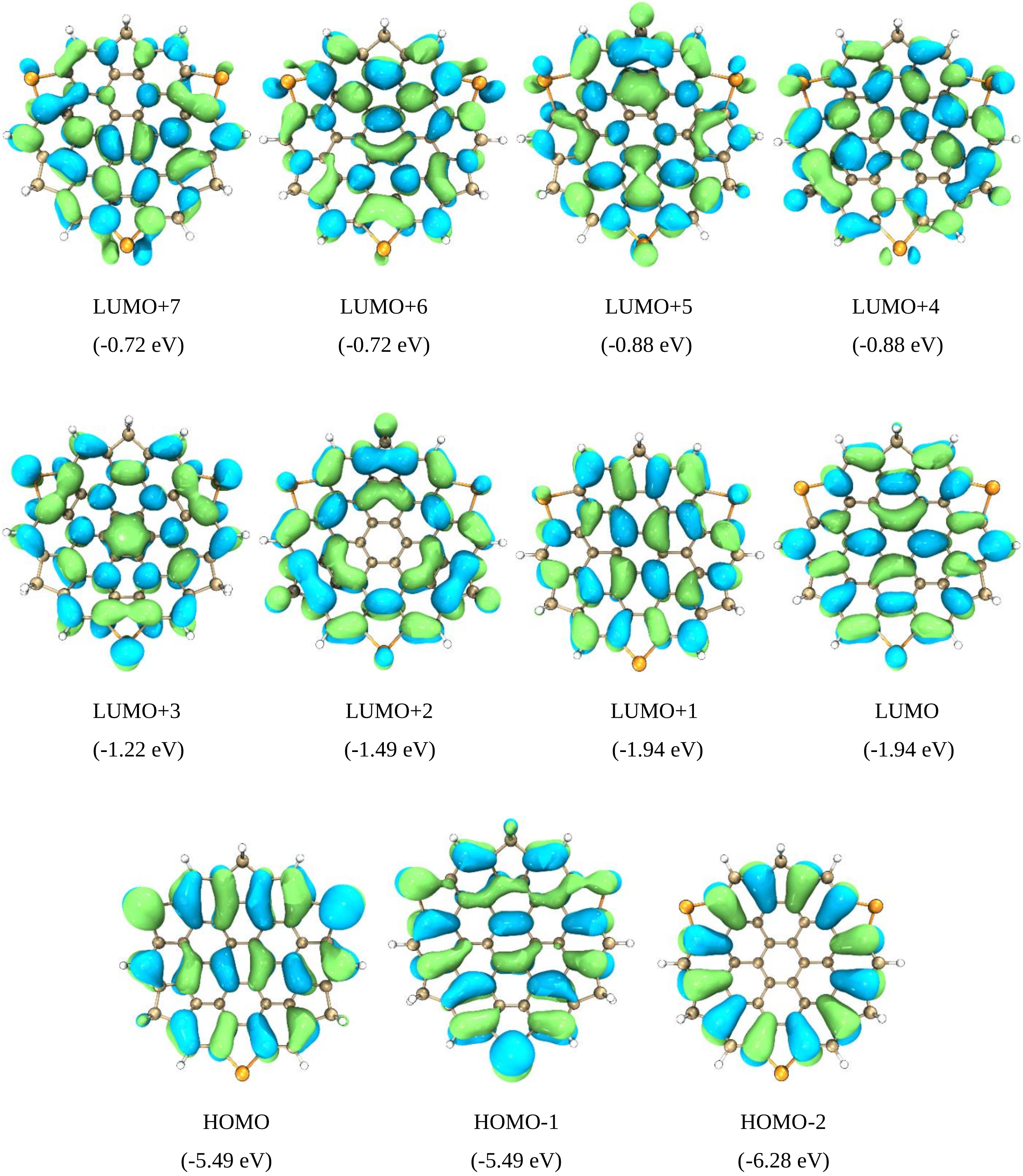

HOMO-LUMO Gap: 3.55 eV

Supplementary Figure 50. Kohn–Sham molecular orbitals (from HOMO-2 to LUMO+7) of **1b'** calculated at the B3LYP/6-311G(d,p) level of theory.

Supplementary Table 14. Major transitions corresponding to the UV spectrum of **1b'** calculated by TDDFT. (PBE/def2tzvp)

Excited state	Energy (eV)	Wavelength (nm)	Oscillator strength (f)	Description
S_1	2.7371	452.98	0.0007	HOMO→LUMO (49.0%)<br>HOMO-1→LUMO+1 (49.0%)
S_2	2.8166	440.19	0.0000	HOMO-1→LUMO (49.6%)<br>HOMO→LUMO+1(49.6%)
S_3	3.1788	390.03	0.4830	HOMO-1→LUMO+1 (42.0%)<br>HOMO→LUMO (42.0%)<br>HOMO→LUMO+2 (9.1%)
S_4	3.1788	390.03	0.4830	HOMO→LUMO+1 (42.0%)<br>HOMO-1→LUMO (42.0%)<br>HOMO-1→LUMO+2 (9.1%)
S_5	3.3258	372.80	0.0543	HOMO→LUMO+2 (80.7%)<br>HOMO-1→LUMO+1 (5.2%)<br>HOMO→LUMO (5.2%)
S_6	3.3258	372.80	0.0543	HOMO-1→LUMO+2 (80.7%)<br>HOMO→LUMO+1 (5.2%)<br>HOMO-1→LUMO (5.2%)
S_{18}	3.9918	310.60	0.0964	HOMO-1→LUMO+7 (32.9%)<br>HOMO→LUMO+6 (32.8%)<br>HOMO-5→LUMO+1 (10.9%)<br>HOMO-2→LUMO (5.1%)
S_{19}	3.9918	310.60	0.0964	HOMO→LUMO+7 (32.9%)<br>HOMO-1→LUMO+6 (32.8%)<br>HOMO-1→LUMO+5 (10.9%)<br>HOMO→LUMO+4 (5.1%)

HOMO index: 192, LUMO index: 193

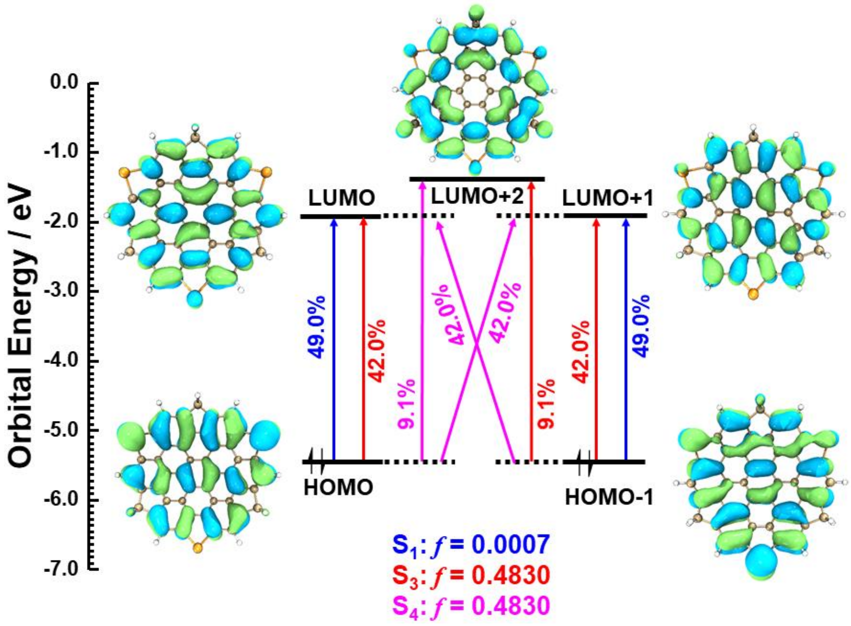

Supplementary Figure 51. Orbital correlation diagram and transition composition of the $S_0 \rightarrow (S_1, S_3, S_4)$ excited states for **1b'** calculated at PBE/def2tzvp level of theory.

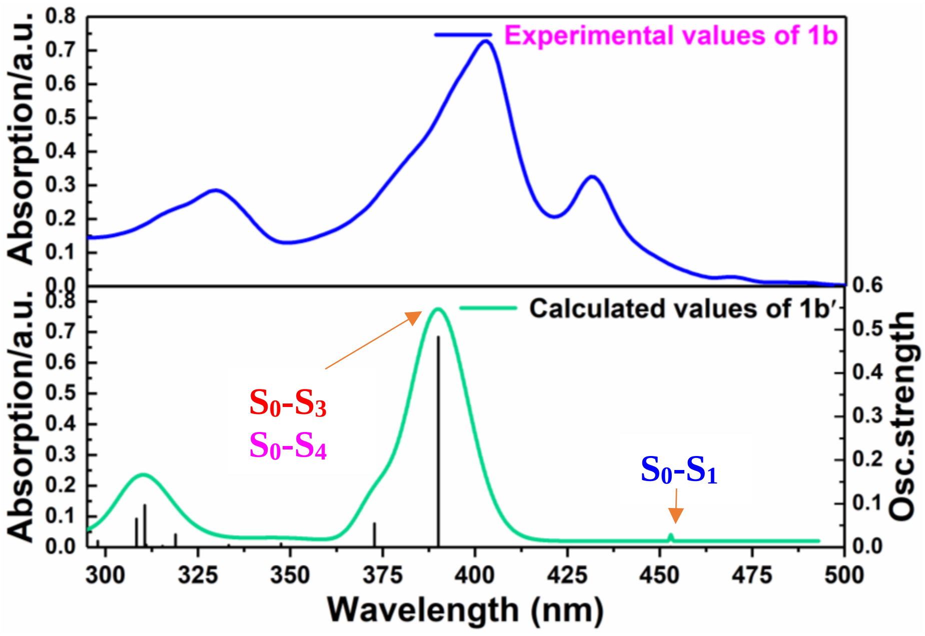

Supplementary Figure 52. The experimental (top) and calculated (bottom) UV-vis absorption spectra of **1b'**, and the main absorption spectra of different transition from state to excited states (bottom).

7.4 Electrostatic Potential Surfaces and Molecular Dipole Moments Calculation

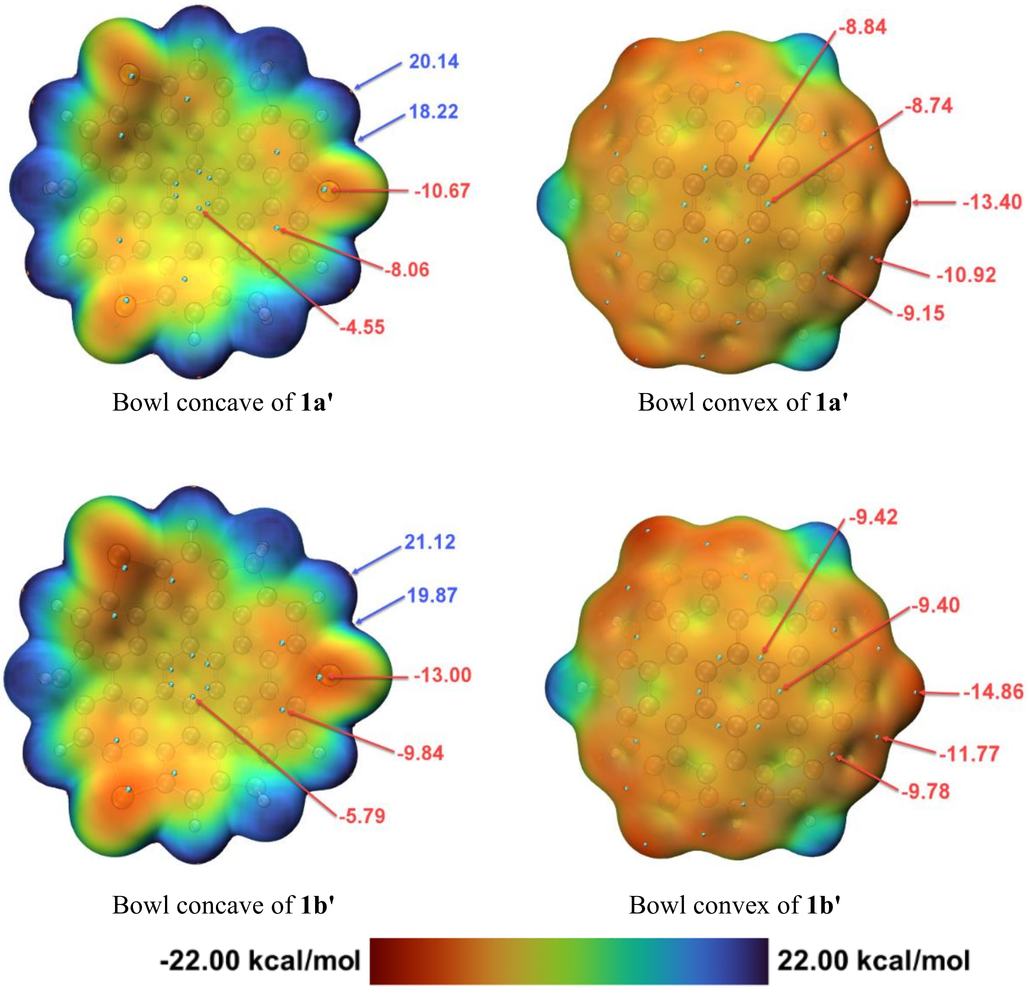

Supplementary Figure 53. Calculated electrostatic potentials on the 0.001 au. isodensity surface with surface maxima (blue) and minima points (red). (B3LYP/6-311G(d,p)).

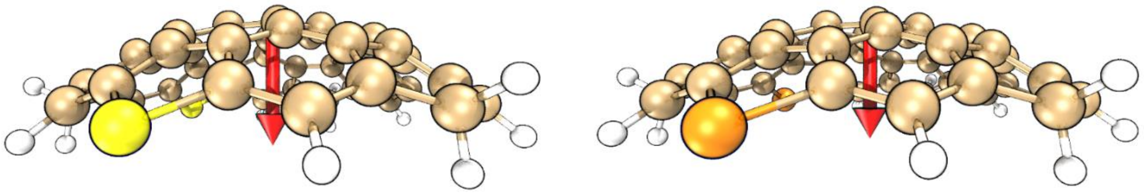

1a' (3.59D) **1b' (3.45D)**

Supplementary Figure 54. Molecular dipole moments (Debye) of **1a'** and **1b'.** (M062x/6-31G(d)).

7.5 Binding energies of 1a-Me@C_{60} and 1a-Me@C_{70}

All complexes evaluated were fully optimized at the B3LYP-D_3/6-311G(d,p) level without symmetry constrain. The B3LYP-D_3 functional was appropriate for large van der Waals interaction systems because the dispersion correction (D_3) was an add-on to standard Kohn–Sham density functional theory (DFT), which has been refined for broader range of applicability, regarding higher accuracy and less empiricism. [16] The B3LYP-D_3 functional has been demonstrated for a wide variety of noncovalent complexes. Binding energies E_{bind} for the association of C_{60} and C_{70} with **1a-Me** were calculated by B3LYP-D_3/6-311G(d,p) level. The counterpoise (CP) procedure was used to calculate the interaction energy in order to correct the basis set superposition error (BSSE) [17] and applied to only the optimized geometries of the complexes. E_{bind} can be expressed as the following equation:

$$E_{\mathrm{bind}} = E_{\mathrm{HG}}(\mathrm{b_{HG}}) - [E_{\mathrm{H}}(\mathrm{b_H}) + E_{\mathrm{G}}(\mathrm{b_G})] + E_{\mathrm{BSSE}}$$

$$E_{\mathrm{BSSE}} = [E_{\mathrm{H}}(\mathrm{b_H}) - E_{\mathrm{H}}(\mathrm{b_{HG}})] + [E_{\mathrm{G}}(\mathrm{b_G}) - E_{\mathrm{G}}(\mathrm{b_{HG}})]$$

where subscript refers to the geometry (H for buckybowl, G for fullerene, HG for complex) and the parentheses indicates the basis set used. $E_H(b_H)$ and $E_G(b_G)$ are assumed to be monomer energies evaluated using each basis set for E_H and E_G. $E_{HG}(b_{HG})$ refers to complex energy evaluated using the full basis set of system HG. E_{BSSE} refers to corrected energy for basis set superposition error.

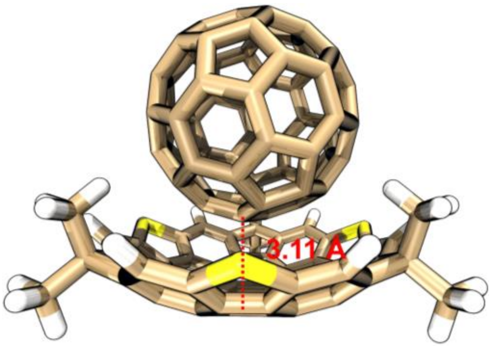

Supplementary Figure 55. Calculated structure of **1a-Me@C_{60}** at the B3LYP-D_3/6-311G(d,p) level of theory.

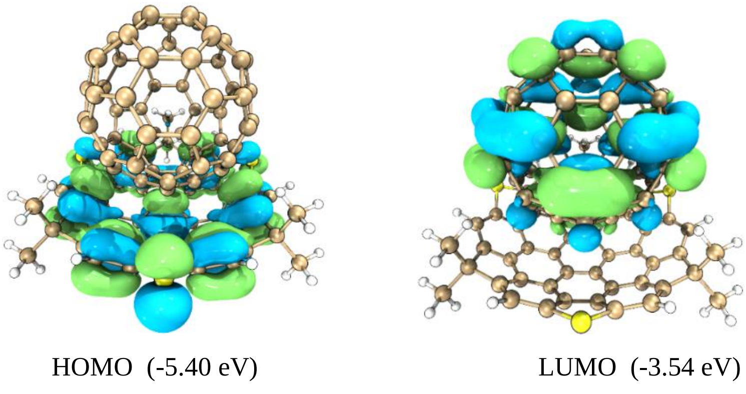

Supplementary Figure 56. (a) HOMO and (b) LUMO of **1a-Me@C_{60}**.

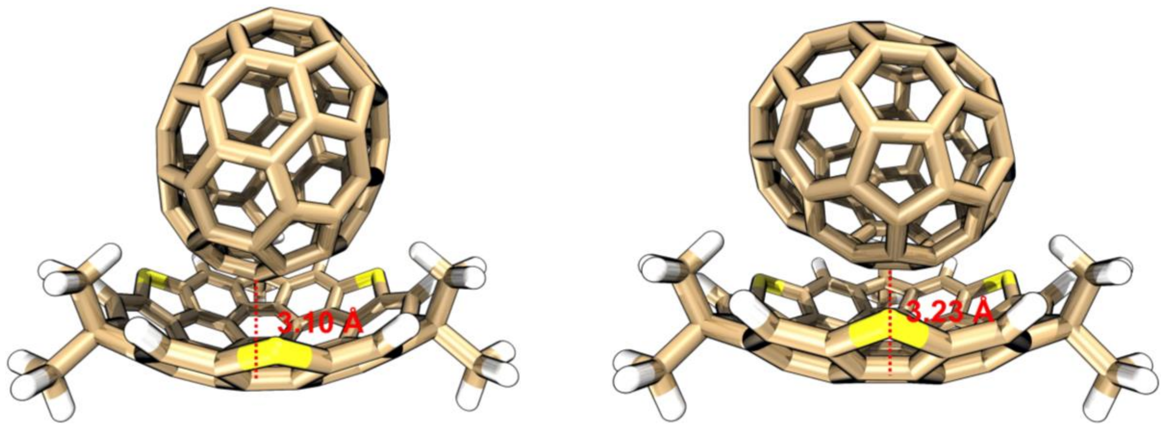

upright **latericumbent**

Supplementary Figure 57. Calculated structure of **1a-Me@C_{70}** at the B3LYP-D_3/6-311G(d,p) level of theory.

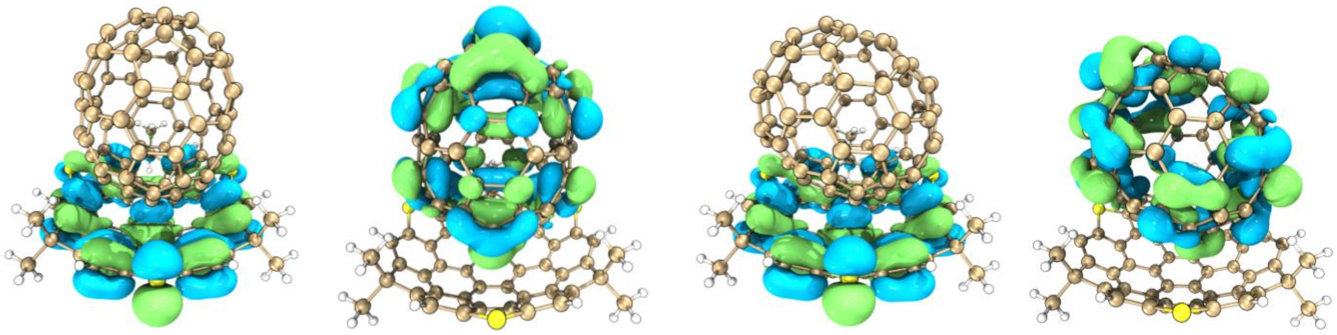

HOMO (-5.41 eV) LUMO (-3.53 eV) HOMO (-5.41 eV) LUMO (-3.55 eV)

HOMO-LUMO gap: 1.88 eV HOMO-LUMO gap: 1.86 eV

Supplementary Figure 58. (a) HOMO and (b) LUMO of **1a-Me@C_{70}**.

Supplementary Table 15. Equilibrium separations between fullerene and buckybowl (D, Å), their corresponding binding energies (E_{bind}, kcal/mol), and the HOMO-LUMO gap (E_{gap}, eV).

Complex	D (Å)	E_{bind} (kcal/mol)	E_{gap} (eV)
**1a-Me@C_{60}**	3.11	-34.51	1.86
**1a-Me@C70 (upright)**	3.10	-34.72	1.88
**1a-Me@C70 (latericumbent)**	3.23	-36.06	1.86

8. Attached NMR spectra and HRMS spectra

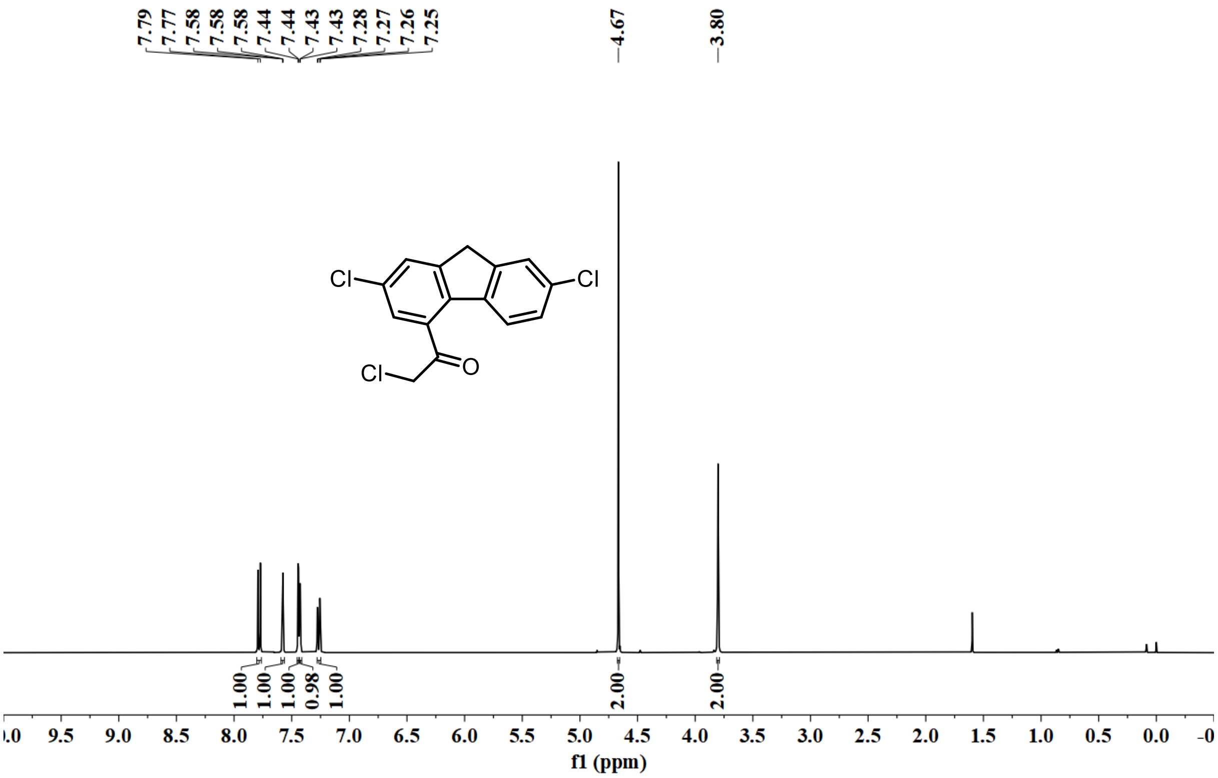

Supplementary Figure 59. ^{1}H NMR spectrum of 2-chloro-1-(2,7-dichloro-9*H*-fluoren-4-yl)ethan-1-one (600 MHz, $CDCl_3$, 298K).

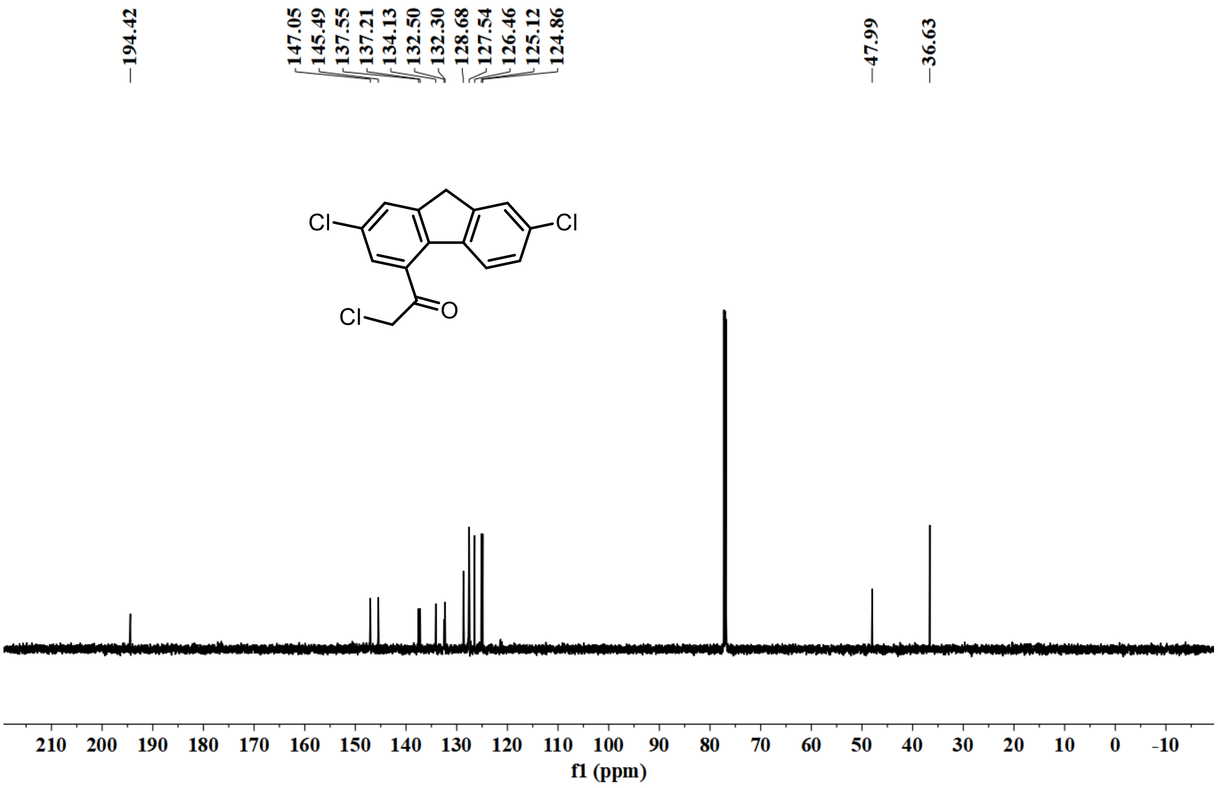

Supplementary Figure 60. ^{13}C NMR spectrum of 2-chloro-1-(2,7-dichloro-9*H*-fluoren-4-yl)ethan-1-one (151 MHz, $CDCl_3$, 298K).

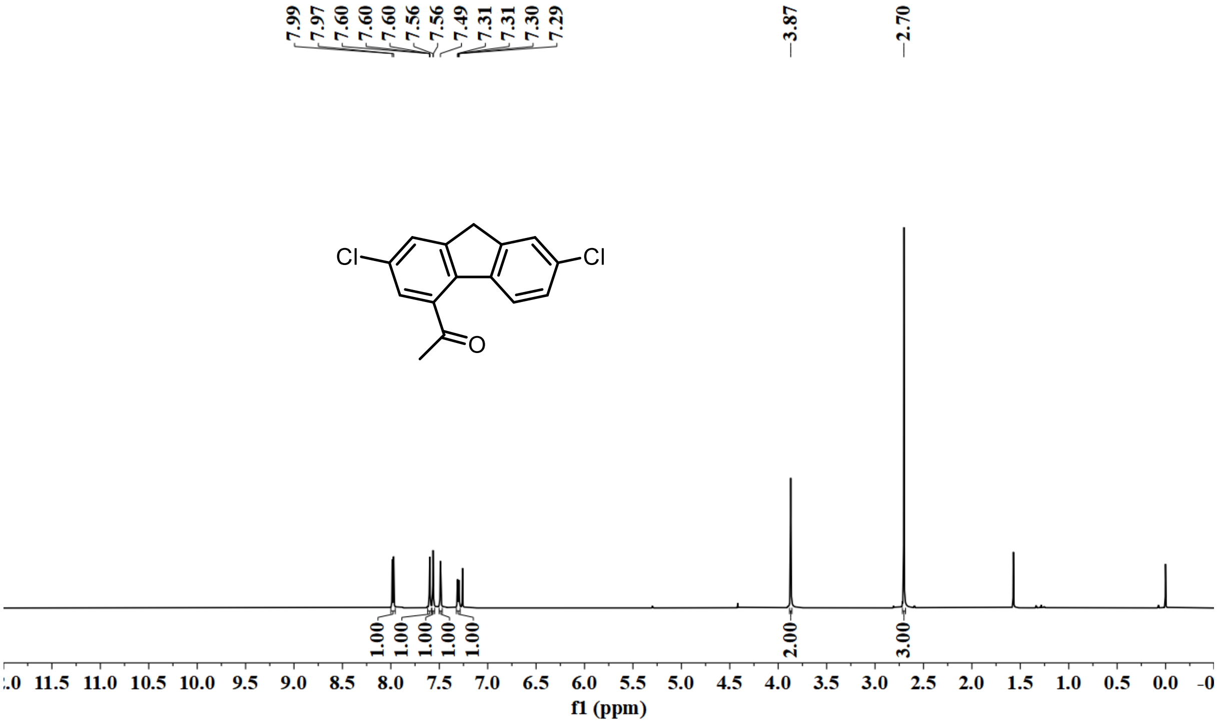

Supplementary Figure 61. ^{1}H NMR spectrum of 1-(2,7-dichloro-9*H*-fluoren-4-yl)ethan-1-one (600 MHz, $CDCl_3$, 298K).

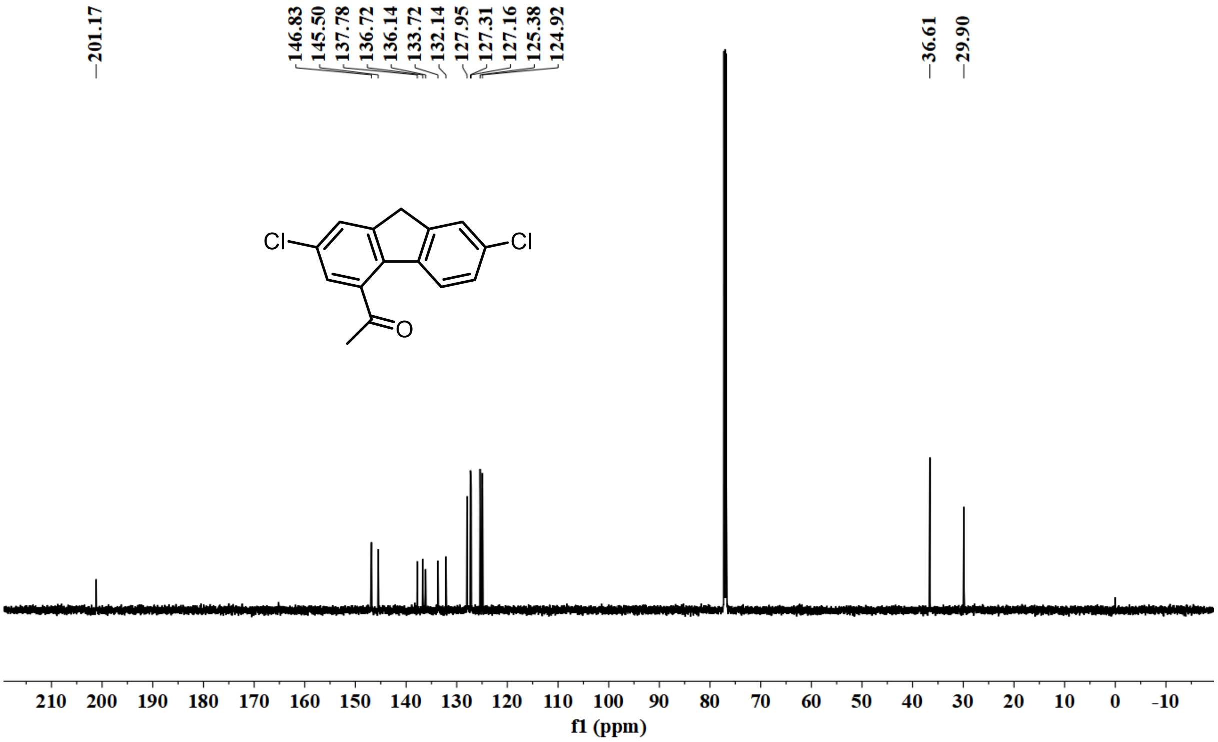

Supplementary Figure 62. ^{13}C NMR spectrum of 1-(2,7-dichloro-9*H*-fluoren-4-yl)ethan-1-one (151 MHz, $CDCl_3$, 298K).

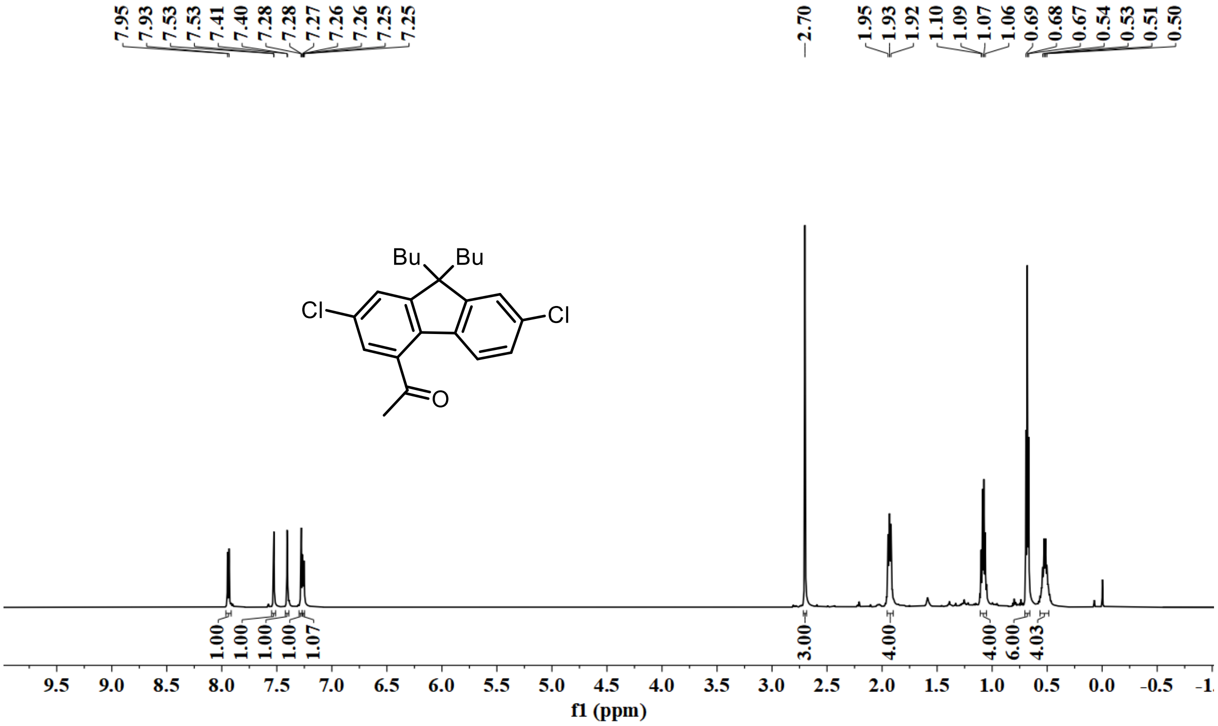

Supplementary Figure 63. ^{1}H NMR spectrum of 1-(9,9-dibutyl-2,7-dichloro-9*H*-fluoren-4-yl)ethan-1-one (**2**) (600 MHz, $CDCl_3$, 298K).

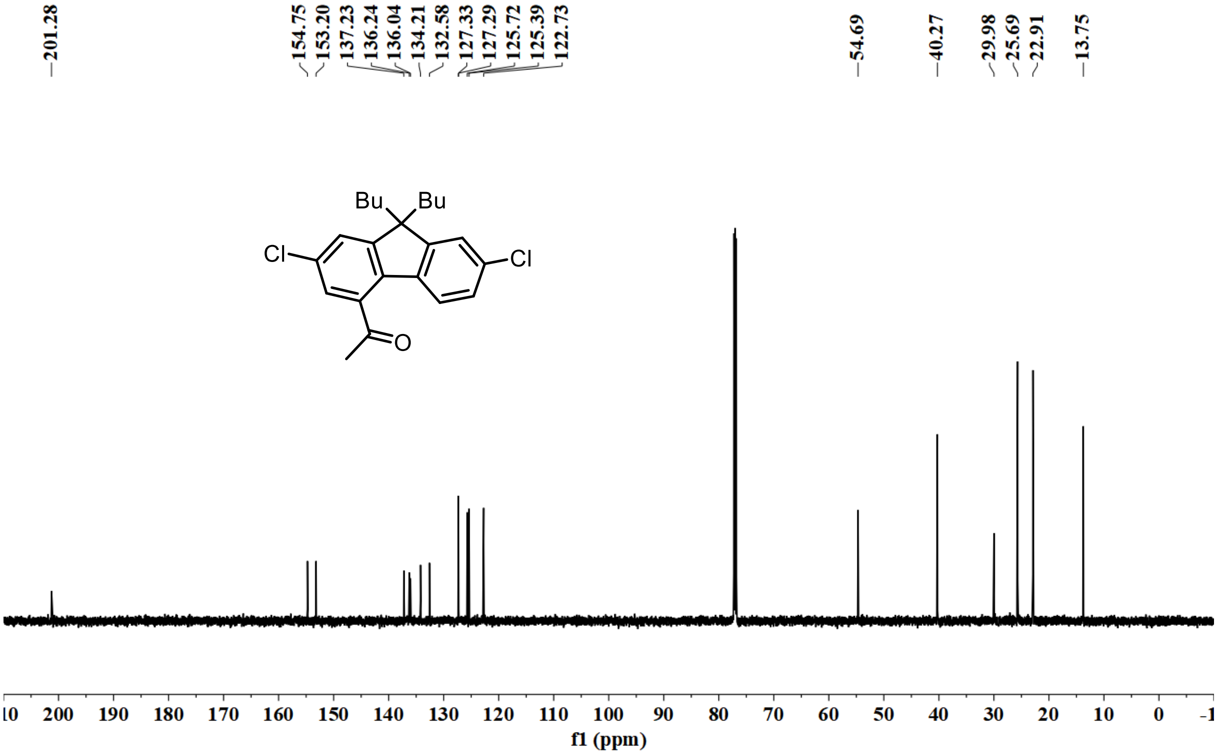

Supplementary Figure 64. ^{13}C NMR spectrum of 1-(9,9-dibutyl-2,7-dichloro-9*H*-fluoren-4-yl)ethan-1-one (**2**) (151 MHz, $CDCl_3$, 298K).

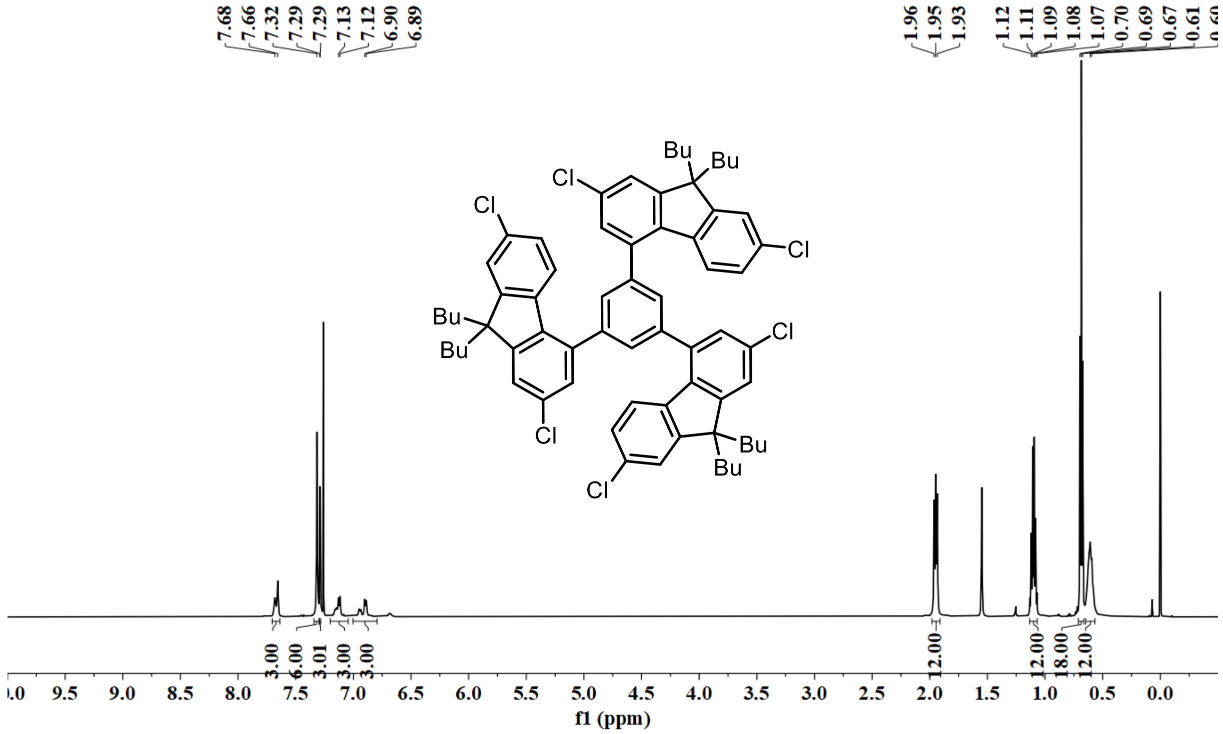

Supplementary Figure 65. ^{1}H NMR spectrum of **3** (600 MHz, $CDCl_3$, 298K).

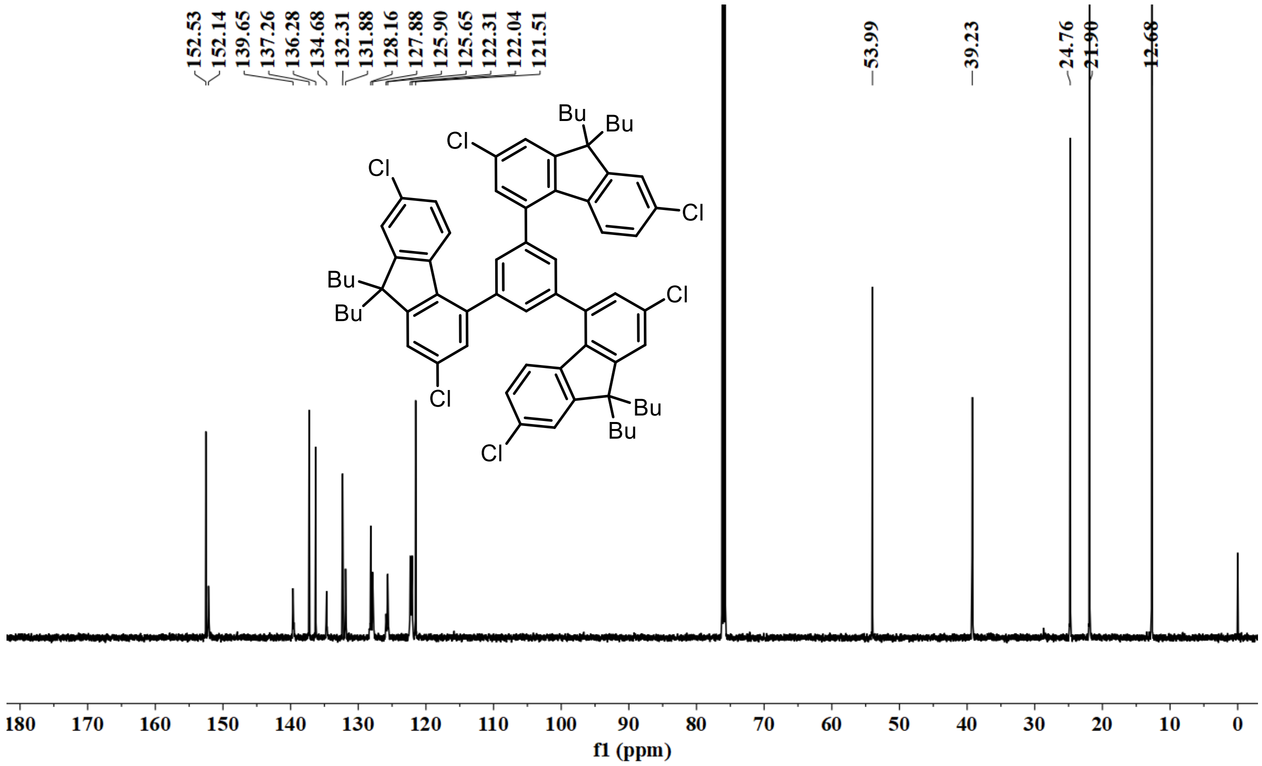

Supplementary Figure 66. ^{13}C NMR spectrum of **3** (151 MHz, $CDCl_3$, 298K).

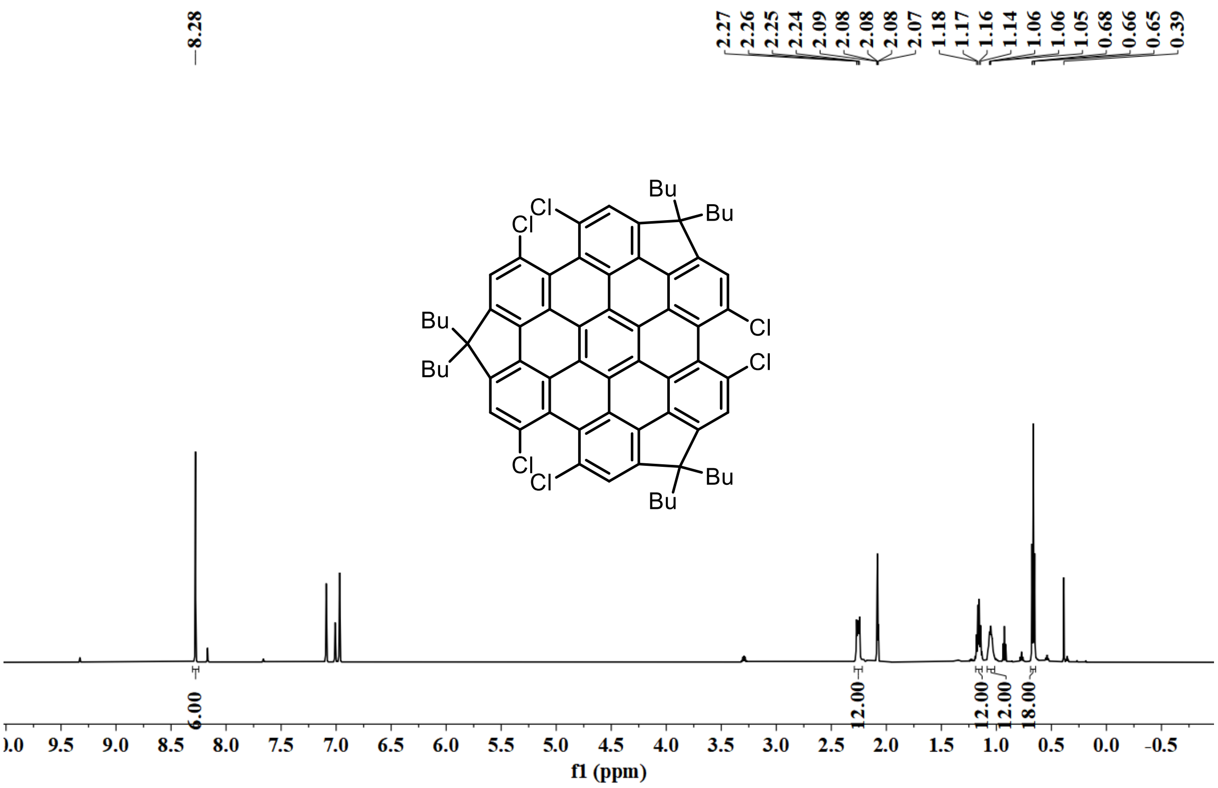

Supplementary Figure 67. ^{1}H NMR spectrum of **4** (600MHz, Tol-d_8, 298K).

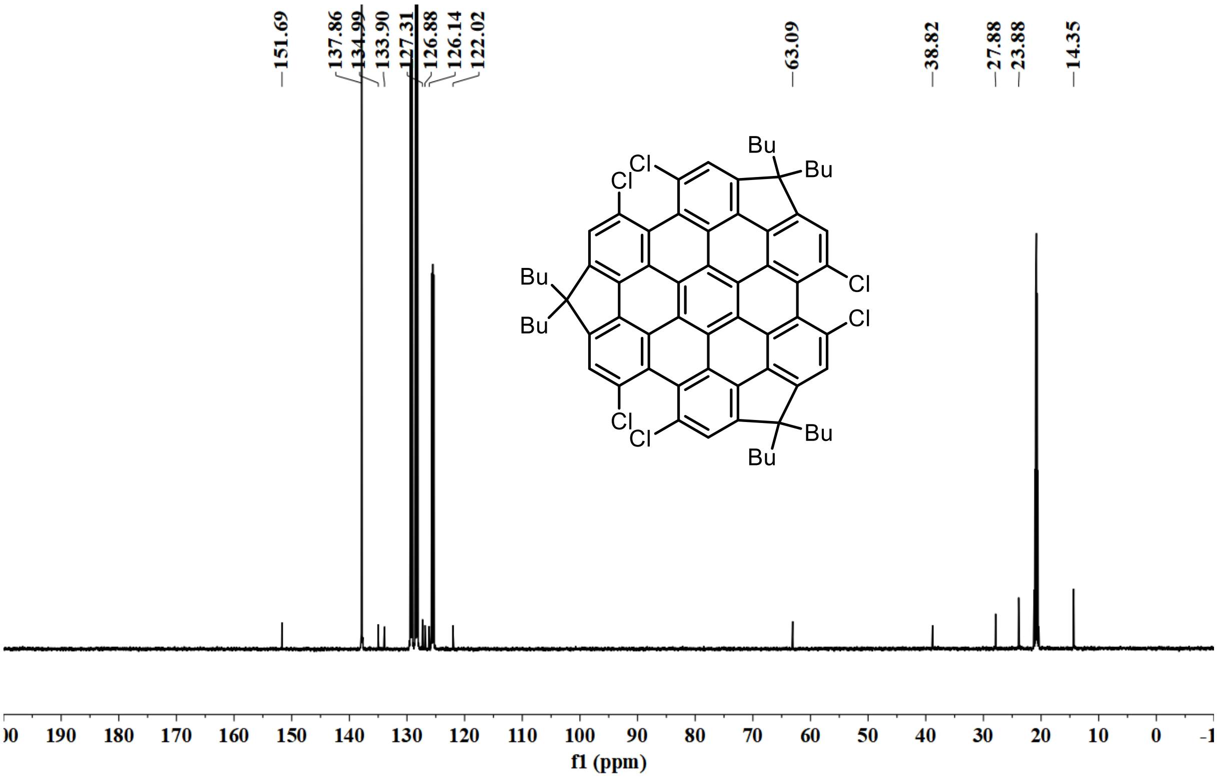

Supplementary Figure 68. ^{13}C NMR spectrum of **4** (151MHz, Tol-d_8, 298K).

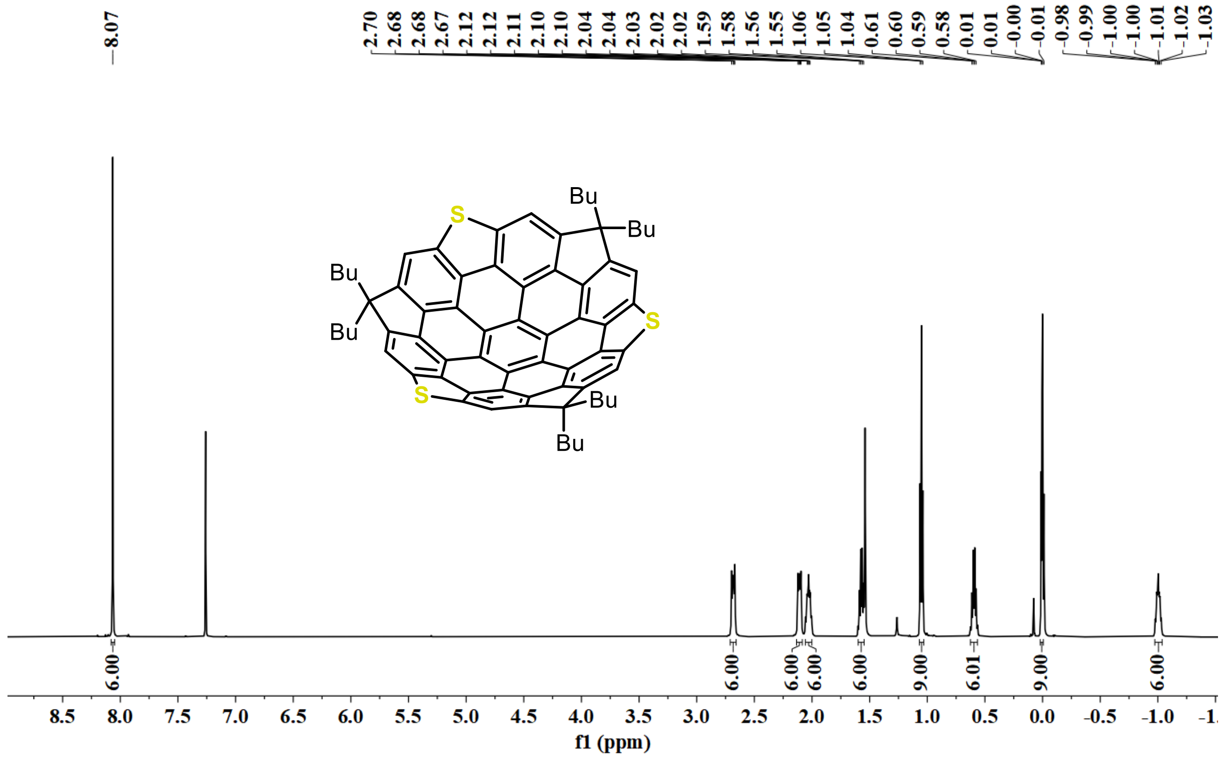

Supplementary Figure 69. ^{1}H NMR spectrum of **1a** (600 MHz, $CDCl_3$, 298K).

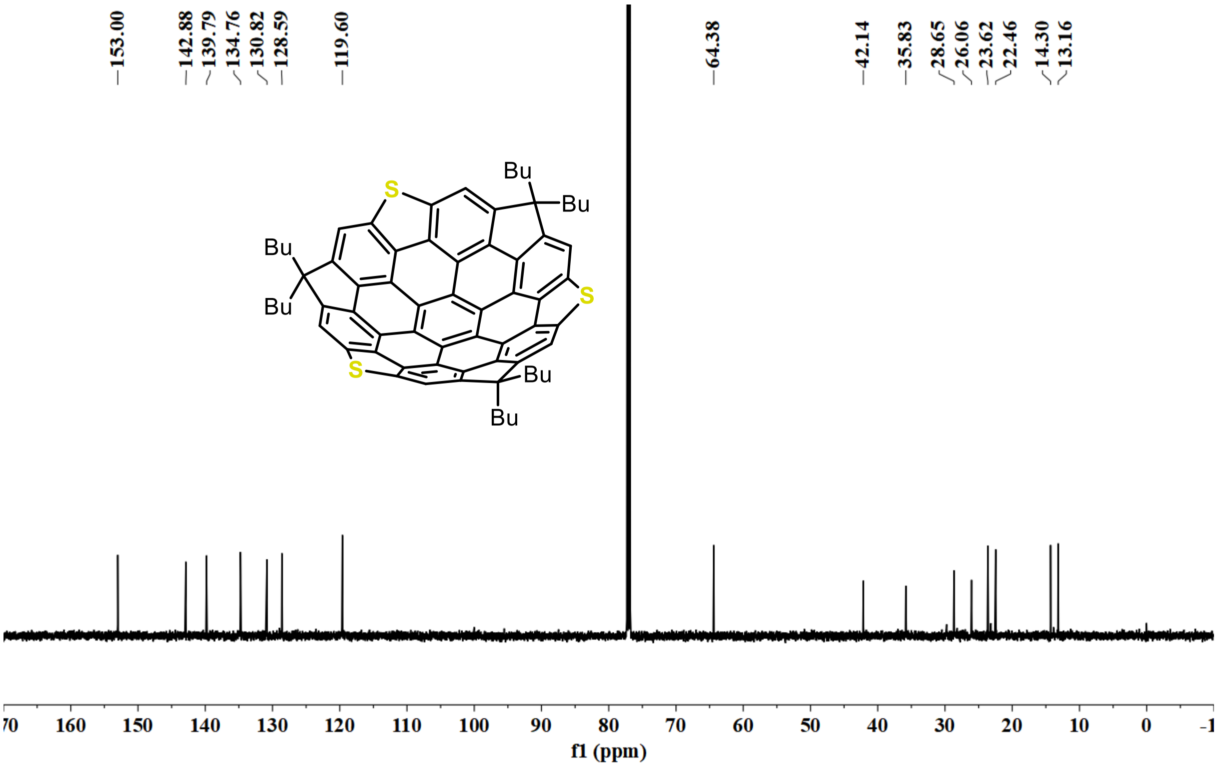

Supplementary Figure 70. ^{13}C NMR spectrum of **1a** (151 MHz, $CDCl_3$, 298K).

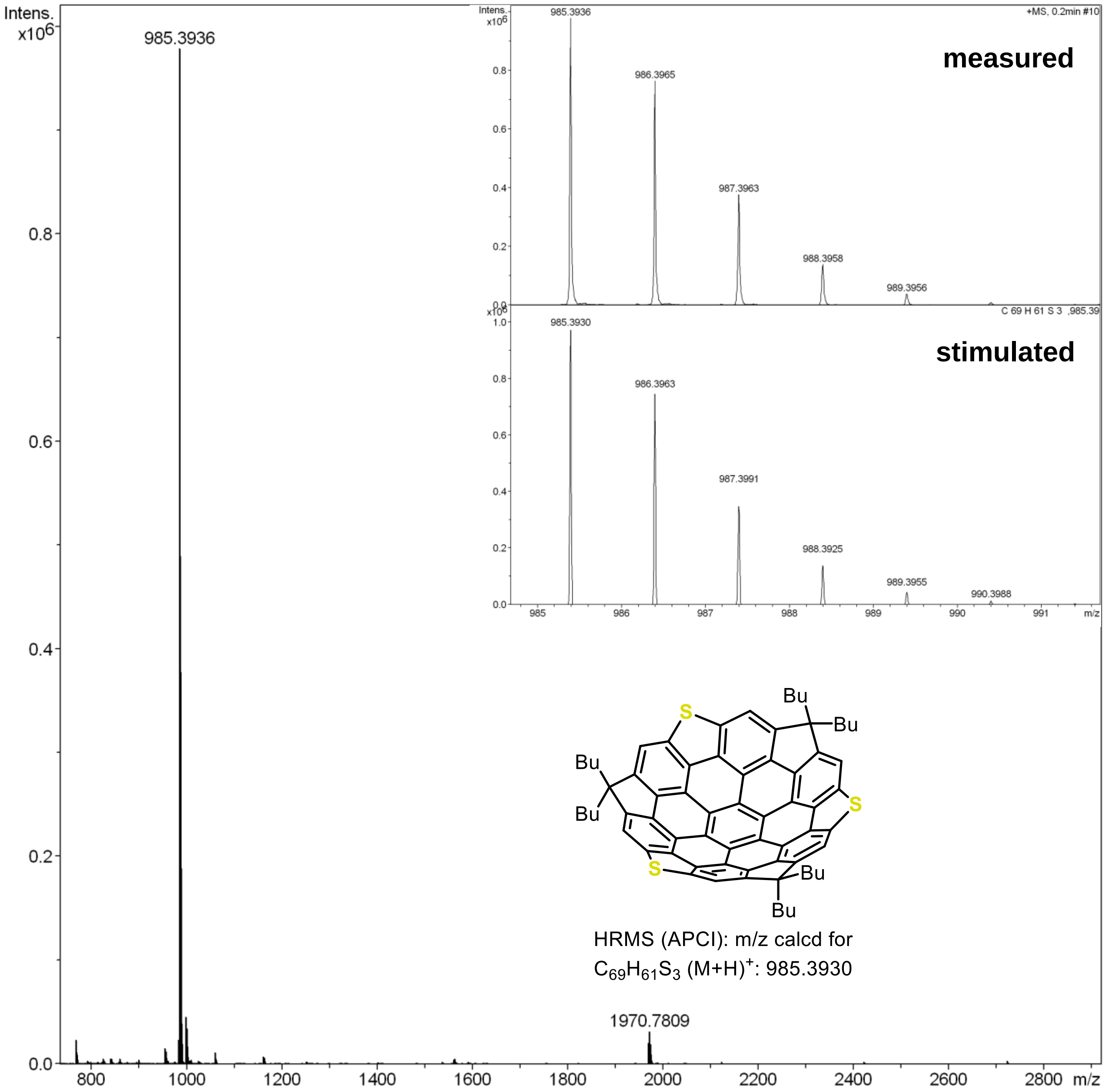

Supplementary Figure 71. HRMS (APCI-TOF, positive mode, methanol/chloroform) spectrum of **1a.** Measured mass spectrum (up) and simulated mass spectrum (down).

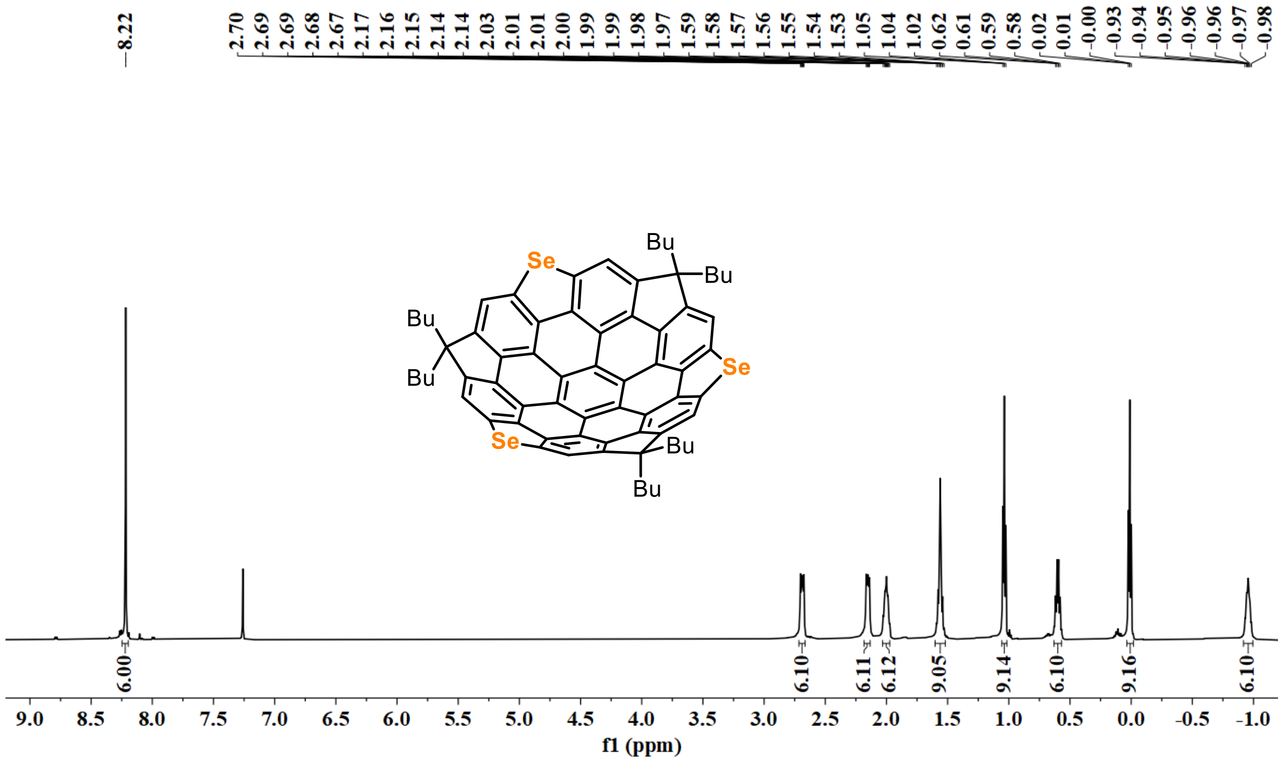

Supplementary Figure 72. ^{1}H NMR spectrum of **1b** (600 MHz, $CDCl_3$, 298K).

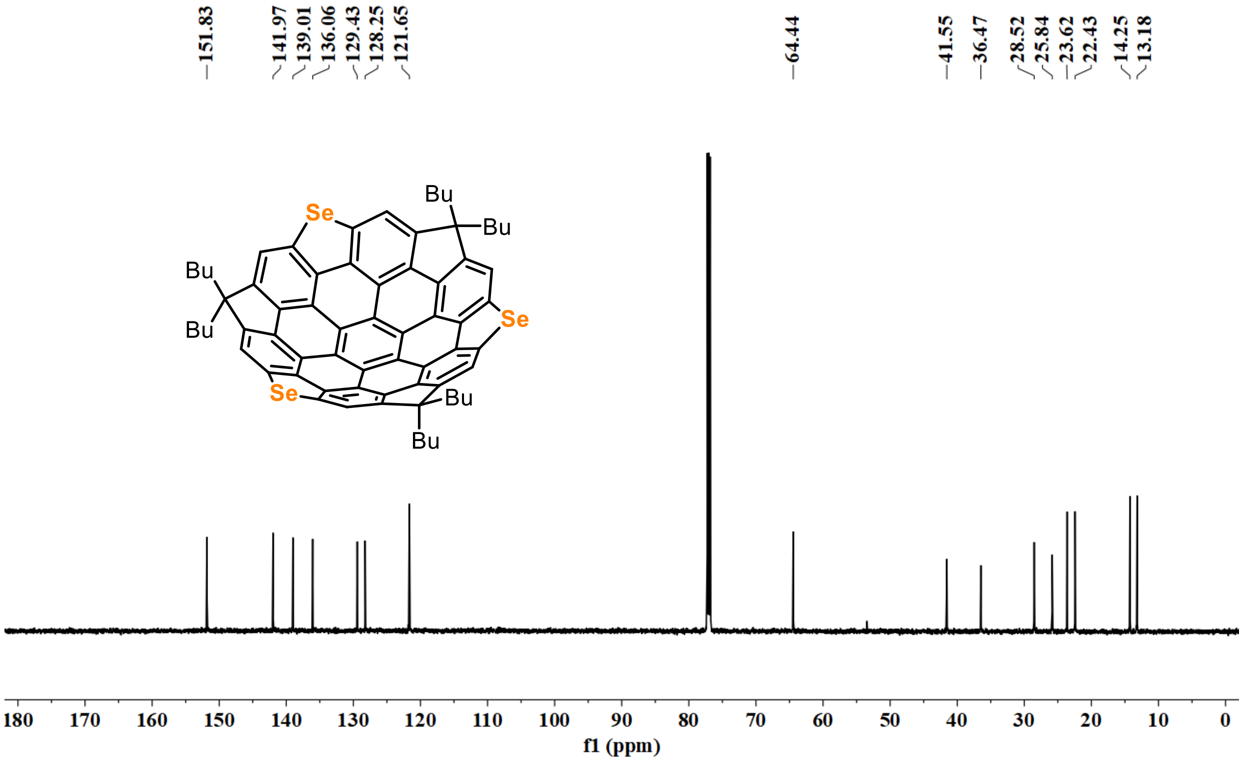

Supplementary Figure 73. ^{13}C NMR spectrum of **1b** (151 MHz, $CDCl_3$, 298K).

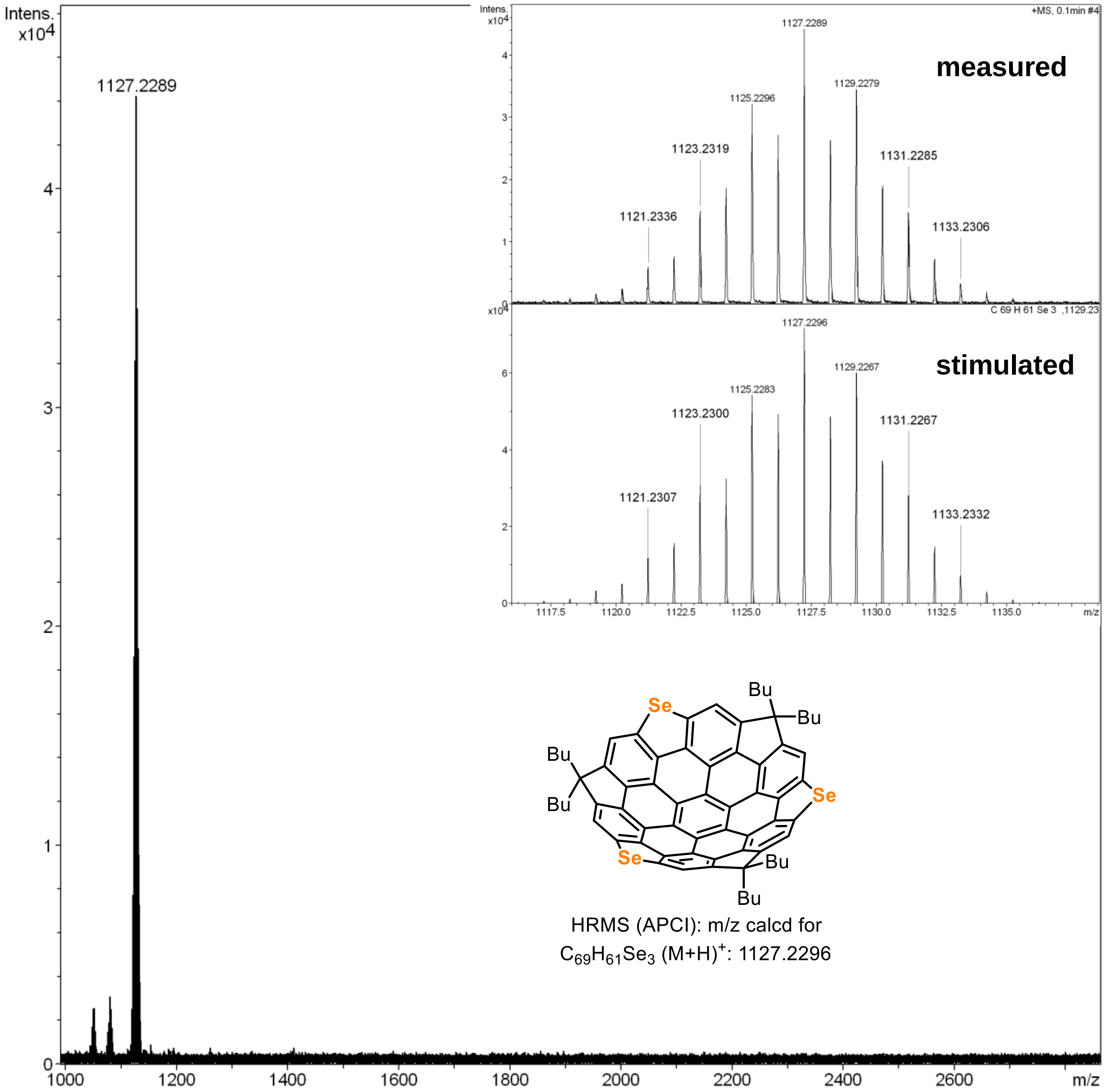

Supplementary Figure 74. HRMS (APCI-TOF, positive mode, methanol/chloroform) spectrum of **1b.** Measured mass spectrum (up) and simulated mass spectrum (down).

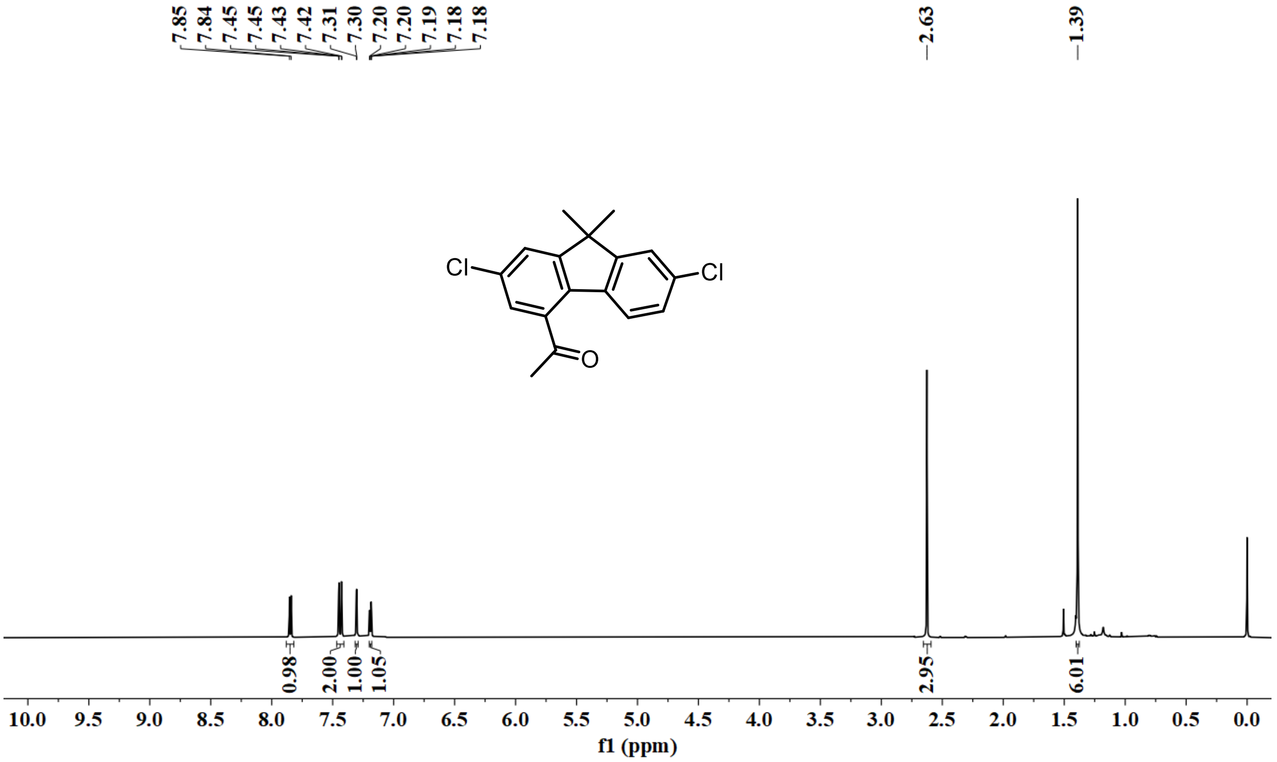

Supplementary Figure 75. ^{1}H NMR spectrum of 1-(2,7-dichloro-9,9-dimethyl-9*H*-fluoren-4-yl)ethan-1-one (**2-Me**) (600 MHz, $CDCl_3$, 298K).

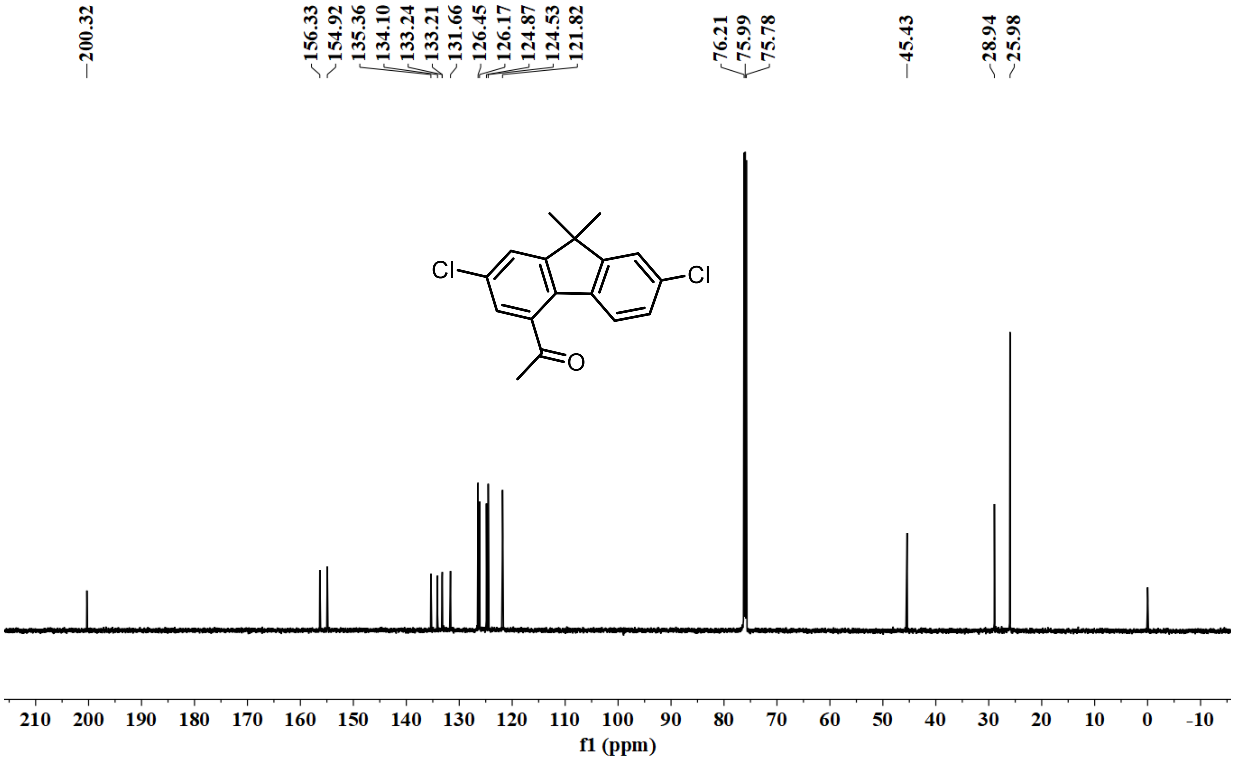

Supplementary Figure 76. ^{13}C NMR spectrum of 1-(2,7-dichloro-9,9-dimethyl-9*H*-fluoren-4-yl)ethan-1-one (**2-Me**) (151 MHz, $CDCl_3$, 298K).

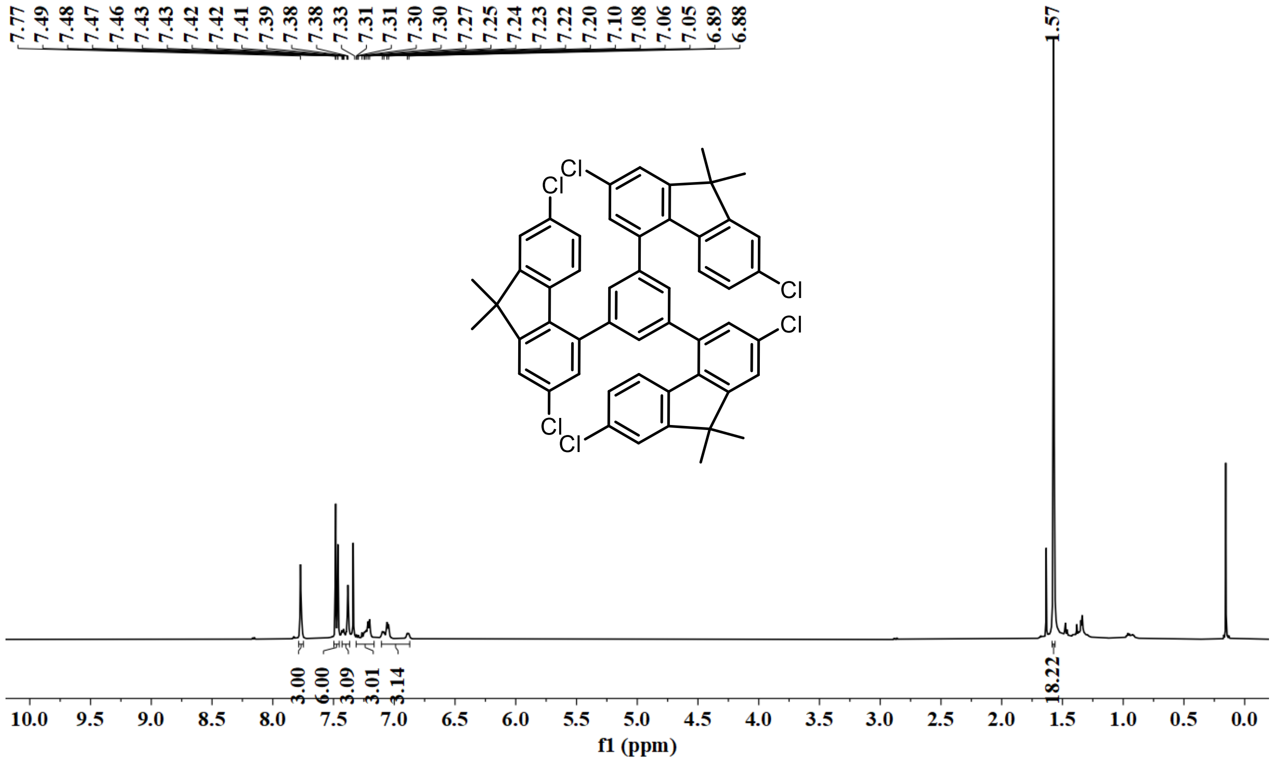

Supplementary Figure 77. ^{1}H NMR spectrum of **3-Me** (600 MHz, $CDCl_3$, 298K).

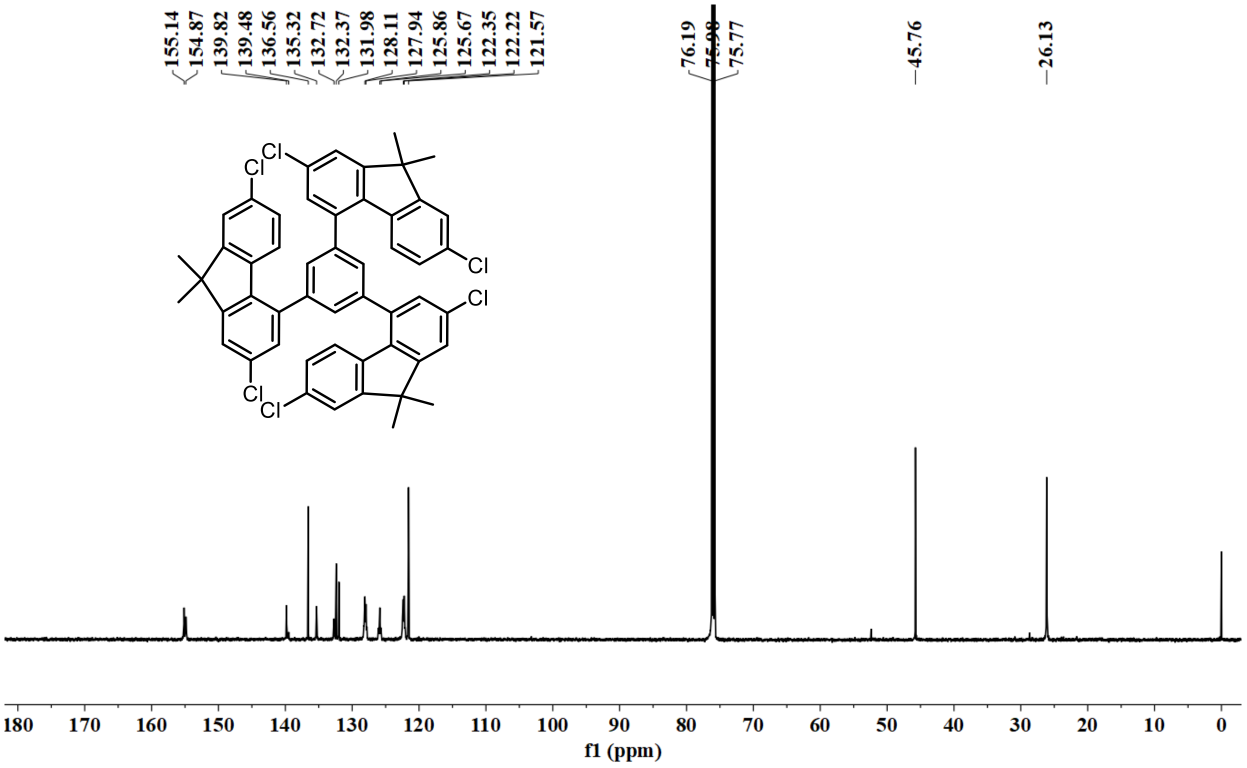

Supplementary Figure 78. ^{13}C NMR spectrum of **3-Me** (151 MHz, $CDCl_3$, 298K).

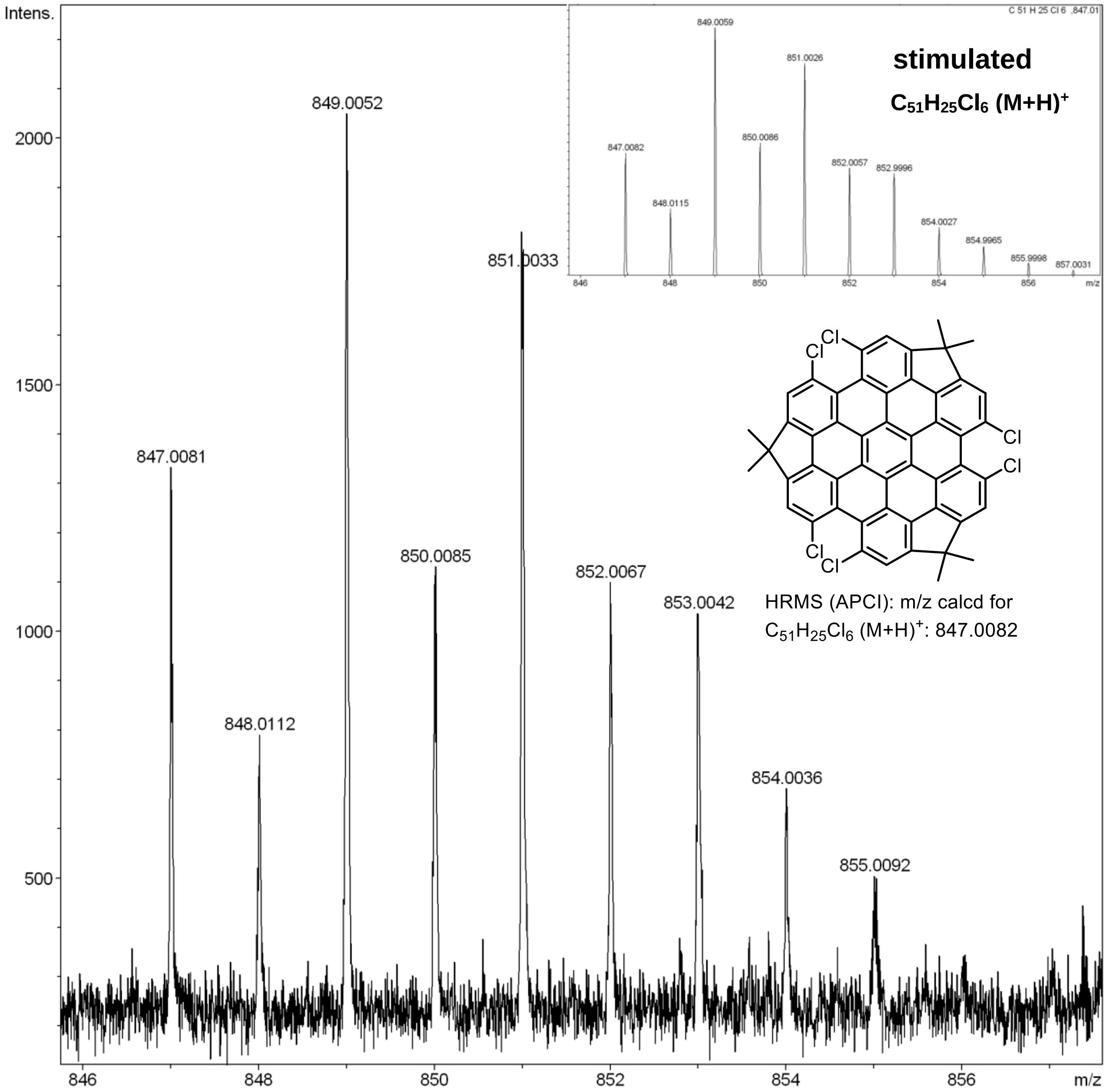

Supplementary Figure 79. HRMS (APCI-TOF, positive mode, methanol/chloroform) spectrum of **3-Me.**

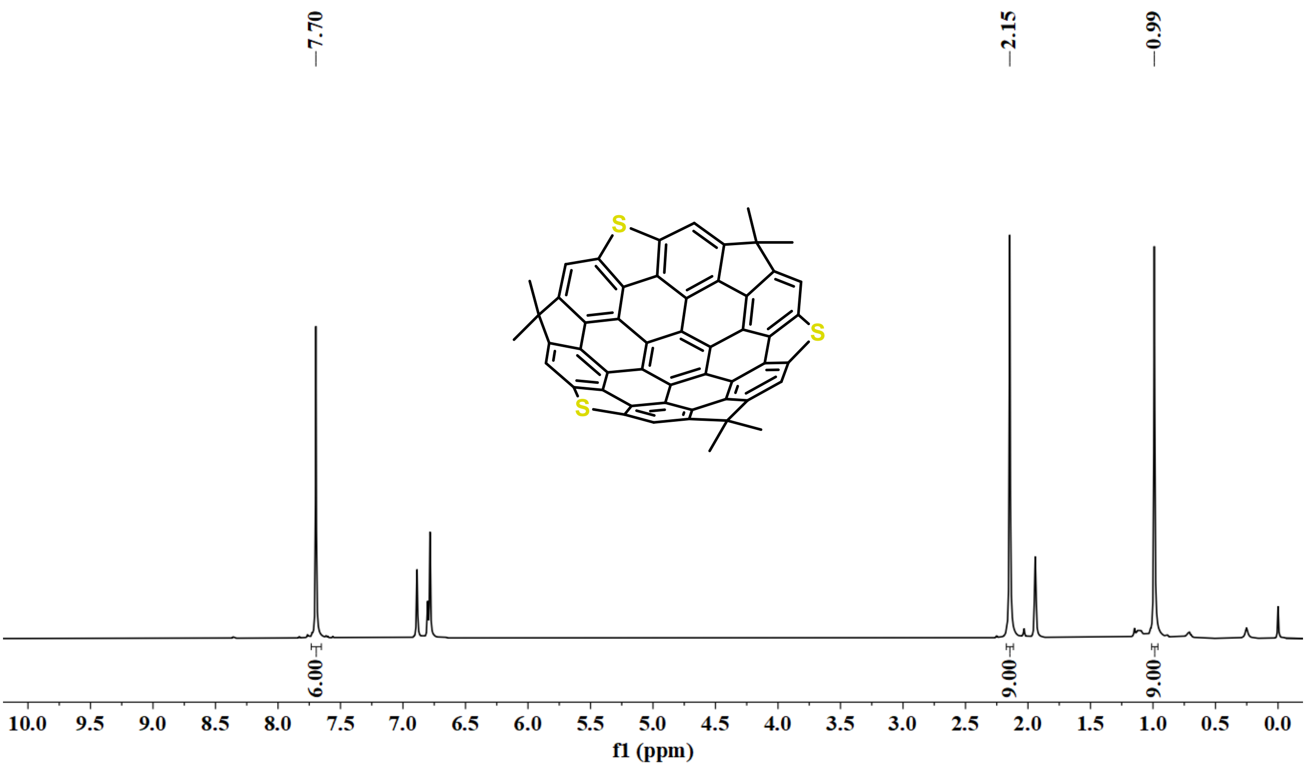

Supplementary Figure 80. ^{1}H NMR spectrum of **1a-Me** (600 MHz, Tol-d_8, 298K).

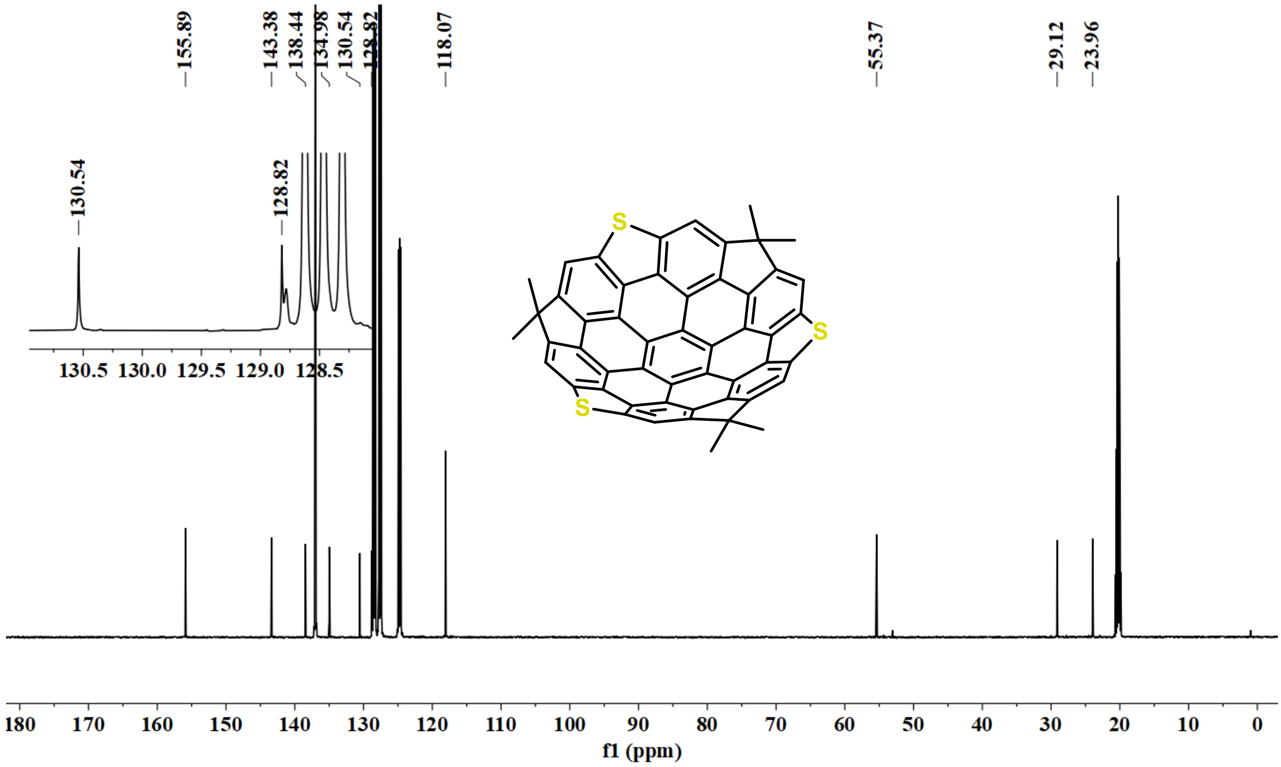

Supplementary Figure 81. ^{13}C NMR spectrum of **1a-Me** (151 MHz, Tol-d_8, 298K).

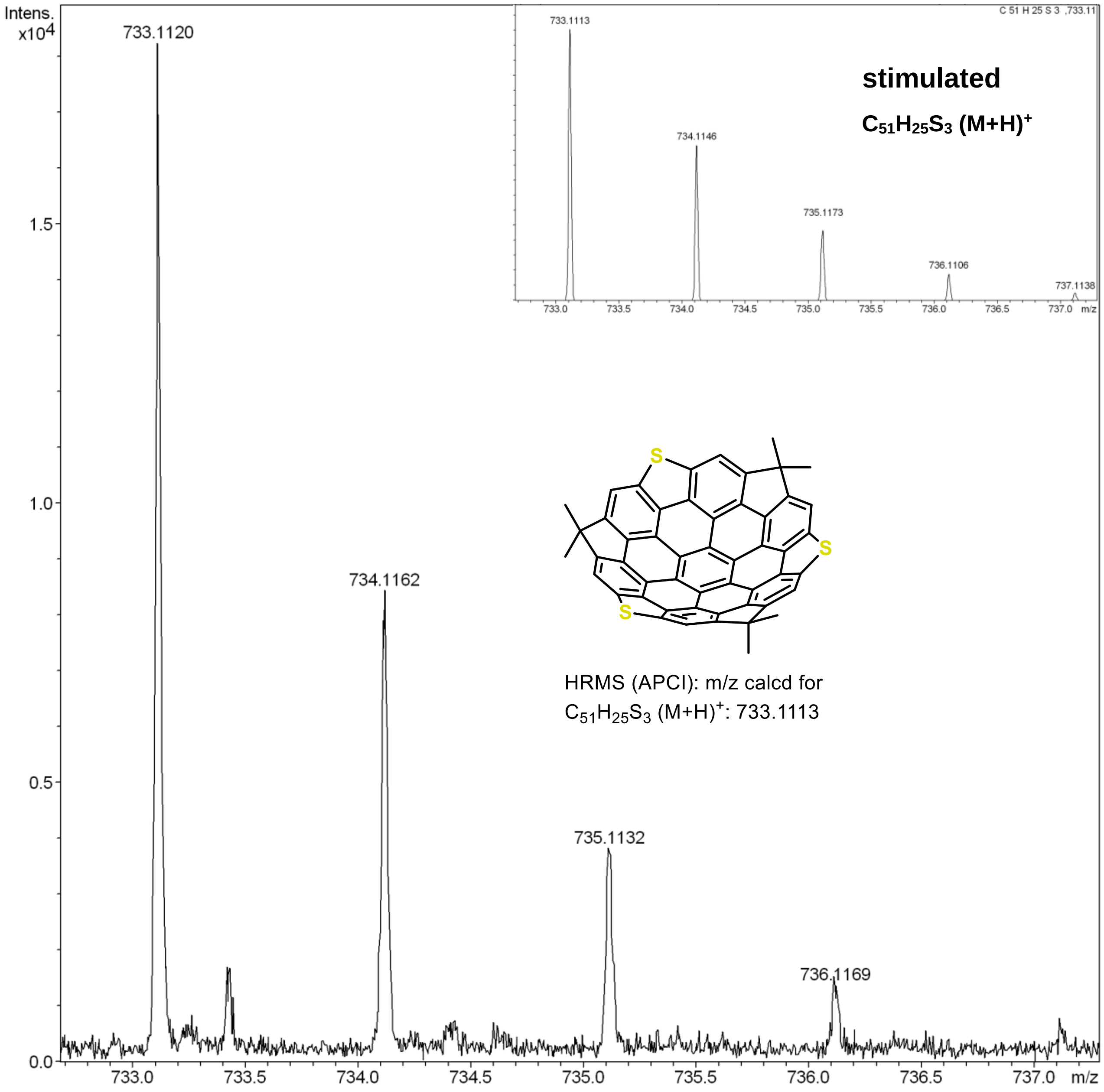

Supplementary Figure 82. HRMS (APCI-TOF, positive mode, methanol/chloroform) spectrum of **1a-Me.**

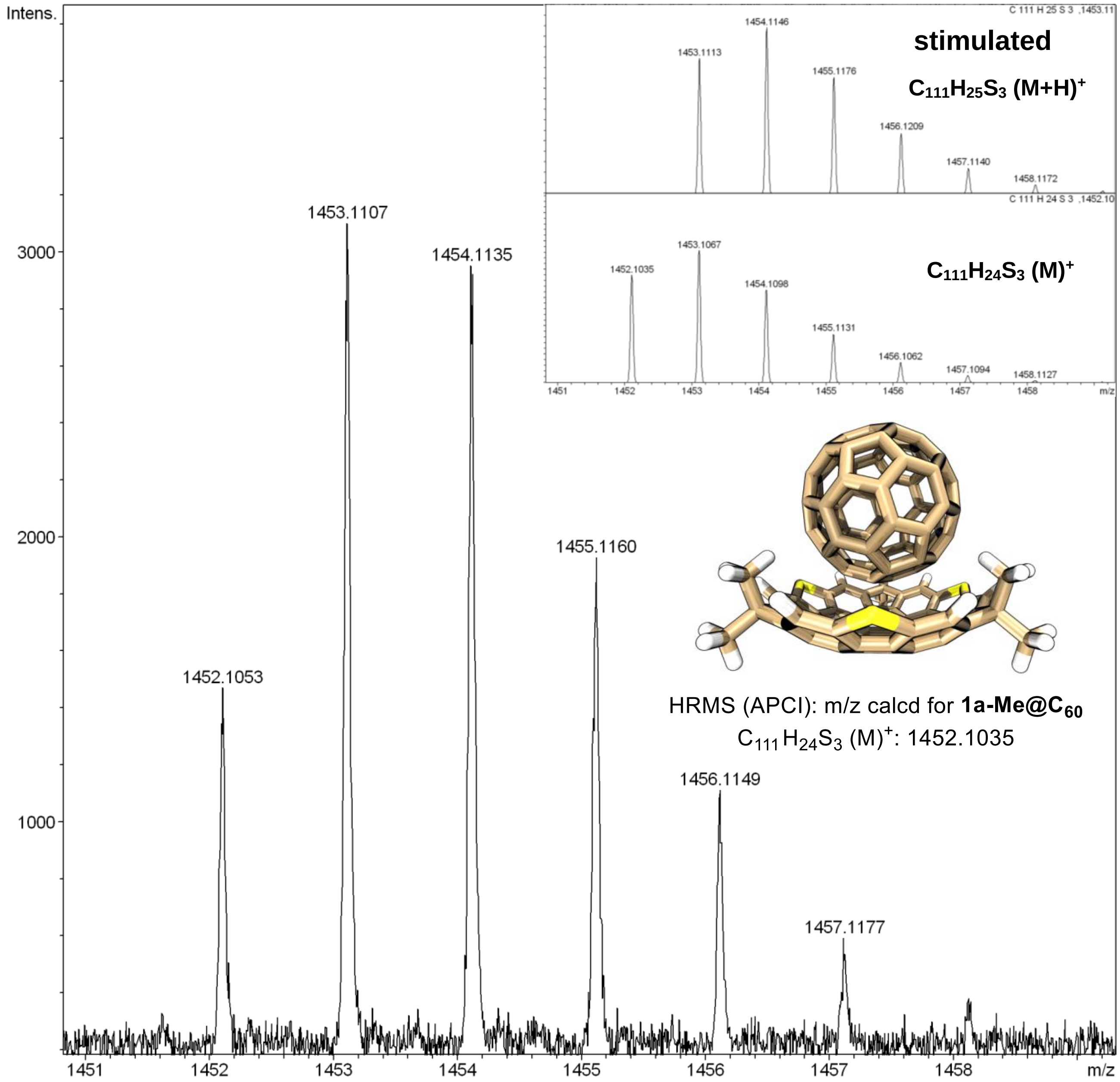

Supplementary Figure 83. HRMS (APCI-TOF, positive mode, methanol/*o*-DCB) spectrum of **1a-Me@C60.**

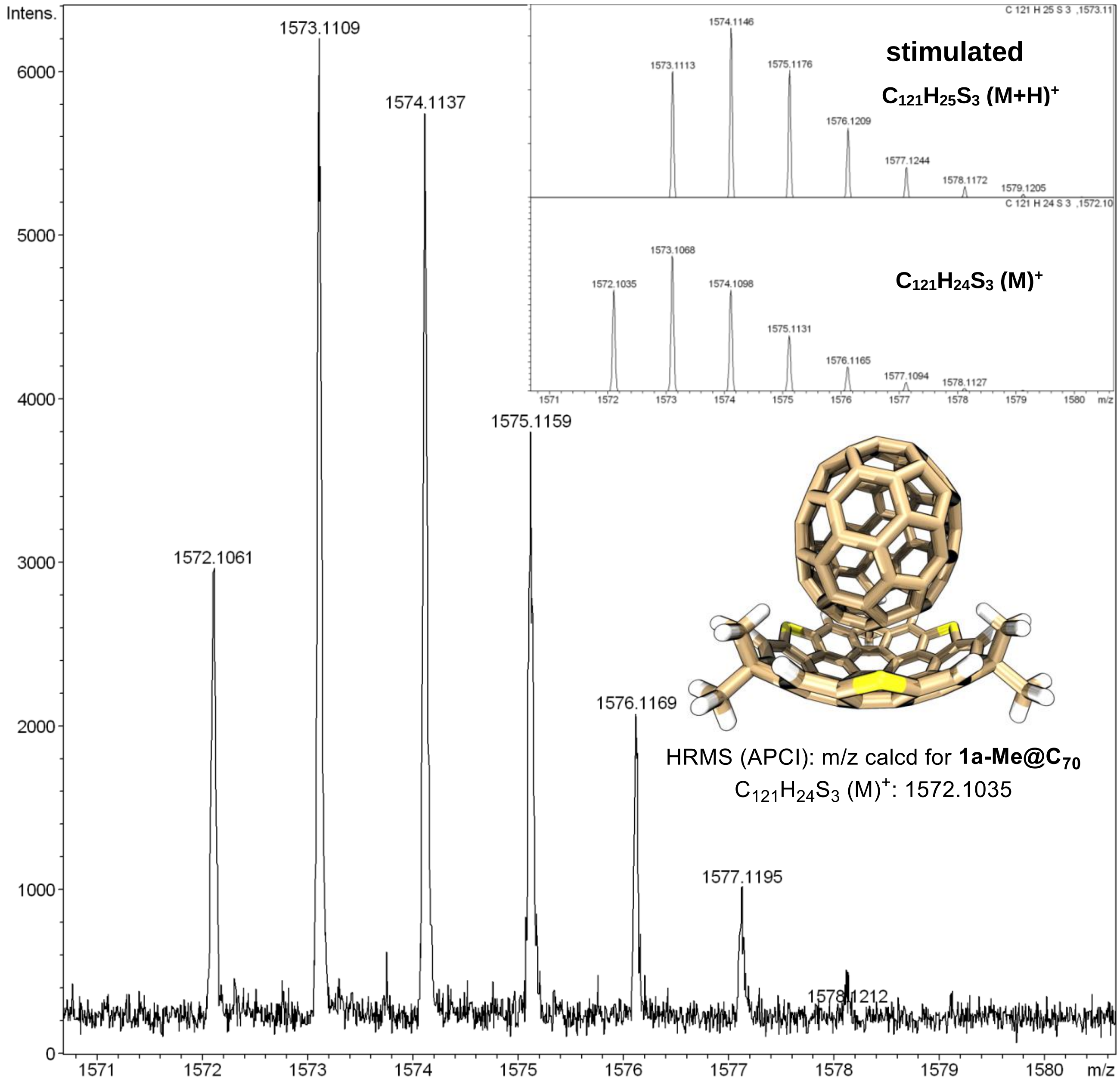

Supplementary Figure 84. HRMS (APCI-TOF, positive mode, methanol/*o*-DCB) spectrum of **1a-Me@C70.**

9. Coordinates of geometry optimized structures

Supplementary Table 16. Cartesian coordinates for NICS(1)$_{zz}$ calculations on **1a'**(optimized by B3LYP / 6-311G(d,p)).

Symbol	X	Y	Z
C	1.424648	-0.000083	1.301736
C	0.712396	-1.233740	1.301736
C	-0.712396	-1.233740	1.301736
C	-1.424648	-0.000083	1.301736
C	-0.712252	1.233823	1.301736
C	0.712252	1.233823	1.301736
C	-1.414088	2.440275	0.920560
C	-0.706633	3.578563	0.550916
C	0.706633	3.578563	0.550916
C	1.414088	2.440275	0.920560
C	-2.820384	0.004498	0.920560
C	-3.455512	1.180226	0.494535
C	-2.749862	2.402449	0.494535
C	-1.406296	-2.444773	0.920560
C	-2.745810	-2.401244	0.550916
C	-3.452443	-1.177319	0.550916
C	1.406296	-2.444773	0.920560
C	0.705651	-3.582674	0.494535
C	-0.705651	-3.582674	0.494535
C	2.820384	0.004498	0.920560
C	3.452443	-1.177319	0.550916
C	2.745810	-2.401244	0.550916
C	2.749862	2.402449	0.494535
C	3.455512	1.180226	0.494535
C	1.216813	4.611170	-0.255302
C	2.547033	4.544647	-0.683328
C	3.310469	3.411384	-0.301148
C	-3.310469	3.411384	-0.301148
C	-2.547033	4.544647	-0.683328
C	-1.216813	4.611170	-0.255302
C	-4.601797	-1.251793	-0.255302
C	-5.209296	-0.066528	-0.683328
C	-4.609580	1.161258	-0.301148
C	-1.299111	-4.572642	-0.301148
C	-2.662263	-4.478119	-0.683328
C	-3.384983	-3.359376	-0.255302
C	3.384983	-3.359376	-0.255302
C	2.662263	-4.478119	-0.683328
C	1.299111	-4.572642	-0.301148
C	4.609580	1.161258	-0.301148
C	5.209296	-0.066528	-0.683328
C	4.601797	-1.251793	-0.255302
C	0.000000	5.458971	-0.703880
S	-4.873622	2.813787	-0.958973
C	-4.727608	-2.729486	-0.703880

S	0.000000	-5.627574	-0.958973
C	4.727608	-2.729486	-0.703880
S	4.873622	2.813787	-0.958973
H	2.953915	5.272471	-1.378103
H	-2.953915	5.272471	-1.378103
H	-6.043051	-0.078070	-1.378103
H	-3.089136	-5.194401	-1.378103
H	3.089136	-5.194401	-1.378103
H	6.043051	-0.078070	-1.378103
H	0.000000	5.629847	-1.784204
H	0.000000	6.447186	-0.228362
H	-5.583427	-3.223593	-0.228362
H	-4.875591	-2.814924	-1.784204
H	5.583427	-3.223593	-0.228362
H	4.875591	-2.814924	-1.784204
Bq	0.000000	0.000000	1.301736
Bq	0.000000	0.000000	0.301736
Bq	0.000000	0.000000	2.301736
Bq	-2.093663	-1.208777	0.924404
Bq	-1.829589	-1.056314	-0.027972
Bq	-2.357737	-1.361240	1.876780
Bq	2.093663	-1.208777	0.924404
Bq	1.829589	-1.056314	-0.027972
Bq	2.357737	-1.361240	1.876780
Bq	0.000000	2.417554	0.924404
Bq	0.000000	2.112628	-0.027972
Bq	0.000000	2.722480	1.876780
Bq	0.000000	-2.420396	0.905610
Bq	0.000000	-2.095589	-0.040170
Bq	0.000000	-2.745203	1.851391
Bq	2.096124	1.210198	0.905610
Bq	1.814833	1.047795	-0.040170
Bq	2.377415	1.372601	1.851391
Bq	-2.096124	1.210198	0.905610
Bq	-1.814833	1.047795	-0.040170
Bq	-2.377415	1.372601	1.851391
Bq	-2.034019	-3.473138	0.121039
Bq	-1.767149	-2.978197	-0.705892
Bq	-2.300889	-3.968079	0.947969
Bq	2.034019	-3.473138	0.121039
Bq	1.767149	-2.978197	-0.705892
Bq	2.300889	-3.968079	0.947969
Bq	4.024835	-0.024943	0.121039
Bq	3.462769	-0.041297	-0.705892
Bq	4.586902	-0.008589	0.947969
Bq	1.990816	3.498081	0.121039
Bq	1.695620	3.019495	-0.705892
Bq	2.286012	3.976668	0.947969
Bq	-1.990816	3.498081	0.121039
Bq	-1.695620	3.019495	-0.705892
Bq	-2.286012	3.976668	0.947969

Bq	-4.024835	-0.024943	0.121039
Bq	-3.462769	-0.041297	-0.705892
Bq	-4.586902	-0.008589	0.947969
Bq	0.000000	-4.387641	-0.114440
Bq	0.000000	-4.974146	0.695506
Bq	0.000000	-3.801136	-0.924385
Bq	3.799809	2.193821	-0.114440
Bq	4.307737	2.487073	0.695506
Bq	3.291881	1.900568	-0.924385
Bq	-3.799809	2.193821	-0.114440
Bq	-4.307737	2.487073	0.695506
Bq	-3.291881	1.900568	-0.924385

Supplementary Table 17. Cartesian coordinates for NICS(1)$_{zz}$ calculations on **1b'**(optimized by B3LYP / 6-311G(d,p)).

Symbol	X	Y	Z
C	1.421620	0.001122	1.331015
C	0.709838	-1.231719	1.331015
C	-0.709838	-1.231719	1.331015
C	-1.421620	0.001122	1.331015
C	-0.711781	1.230598	1.331015
C	0.711781	1.230598	1.331015
C	-1.420567	2.437404	0.975569
C	-0.707150	3.585687	0.652314
C	0.707150	3.585687	0.652314
C	1.420567	2.437404	0.975569
C	-2.821137	0.011545	0.975569
C	-3.475395	1.186812	0.568315
C	-2.765507	2.416374	0.568315
C	-1.400571	-2.448949	0.975569
C	-2.751721	-2.405253	0.652314
C	-3.458871	-1.180434	0.652314
C	1.400571	-2.448949	0.975569
C	0.709888	-3.603186	0.568315
C	-0.709888	-3.603186	0.568315
C	2.821137	0.011545	0.975569
C	3.458871	-1.180434	0.652314
C	2.751721	-2.405253	0.652314
C	2.765507	2.416374	0.568315
C	3.475395	1.186812	0.568315
C	1.217082	4.666238	-0.081813
C	2.560530	4.633605	-0.475119
C	3.326174	3.488111	-0.143098
C	-3.326174	3.488111	-0.143098
C	-2.560530	4.633605	-0.475119
C	-1.217082	4.666238	-0.081813
C	-4.649621	-1.279095	-0.081813
C	-5.293085	-0.099319	-0.475119

C	-4.683879	1.136496	-0.143098
C	-1.357706	-4.624606	-0.143098
C	-2.732555	-4.534287	-0.475119
C	-3.432540	-3.387142	-0.081813
C	3.432540	-3.387142	-0.081813
C	2.732555	-4.534287	-0.475119
C	1.357706	-4.624606	-0.143098
C	4.683879	1.136496	-0.143098
C	5.293085	-0.099319	-0.475119
C	4.649621	-1.279095	-0.081813
C	0.000000	5.534449	-0.490442
Se	-5.064414	2.923941	-0.801240
C	-4.792974	-2.767225	-0.490442
Se	0.000000	-5.847882	-0.801240
C	4.792974	-2.767225	-0.490442
Se	5.064414	2.923941	-0.801240
H	2.982139	5.411560	-1.103785
H	-2.982139	5.411560	-1.103785
H	-6.177618	-0.123172	-1.103785
H	-3.195479	-5.288388	-1.103785
H	3.195479	-5.288388	-1.103785
H	6.177618	-0.123172	-1.103785
H	0.000000	5.753675	-1.562200
H	0.000000	6.500517	0.028393
H	-5.629613	-3.250258	0.028393
H	-4.982828	-2.876837	-1.562200
H	4.982828	-2.876837	-1.562200
H	5.629613	-3.250258	0.028393
Bq	0.000000	0.000000	1.331015
Bq	0.000000	0.000000	0.331015
Bq	0.000000	0.000000	2.331015
Bq	-2.093960	-1.208948	0.986299
Bq	-1.854100	-1.070465	0.025420
Bq	-2.333820	-1.347431	1.947179
Bq	2.093960	-1.208948	0.986299
Bq	1.854100	-1.070465	0.025420
Bq	2.333820	-1.347431	1.947179
Bq	0.000000	2.417896	0.986299
Bq	0.000000	2.140930	0.025420
Bq	0.000000	2.694863	1.947179
Bq	0.000000	-2.427952	0.958300
Bq	0.000000	-2.121936	0.006273
Bq	0.000000	-2.733967	1.910326
Bq	2.102668	1.213976	0.958300
Bq	1.837651	1.060968	0.006273
Bq	2.367685	1.366983	1.910326
Bq	-2.102668	1.213976	0.958300
Bq	-1.837651	1.060968	0.006273
Bq	-2.367685	1.366983	1.910326
Bq	-2.064163	-3.500571	0.249361
Bq	-1.826996	-3.048593	-0.610562

Bq	-2.301331	-3.952548	1.109285
Bq	2.064163	-3.500571	0.249361
Bq	1.826996	-3.048593	-0.610562
Bq	2.301331	-3.952548	1.109285
Bq	4.063665	-0.037333	0.249361
Bq	3.553657	-0.057928	-0.610562
Bq	4.573672	-0.016737	1.109285
Bq	1.999501	3.537903	0.249361
Bq	1.726661	3.106521	-0.610562
Bq	2.272341	3.969285	1.109285
Bq	-1.999501	3.537903	0.249361
Bq	-1.726661	3.106521	-0.610562
Bq	-2.272341	3.969285	1.109285
Bq	-4.063665	-0.037333	0.249361
Bq	-3.553657	-0.057928	-0.610562
Bq	-4.573672	-0.016737	1.109285
Bq	0.000000	-4.460693	0.009839
Bq	0.000000	-4.987447	0.859857
Bq	0.000000	-3.933940	-0.840179
Bq	3.863074	2.230347	0.009839
Bq	4.319256	2.493723	0.859857
Bq	3.406892	1.966970	-0.840179
Bq	-3.863074	2.230347	0.009839
Bq	-4.319256	2.493723	0.859857
Bq	-3.406892	1.966970	-0.840179

Supplementary Table 18. Cartesian coordinates of **1a'** (ground state) optimized structure (optimized by M062x/6-31G(d)).

Symbol	X	Y	Z
C	-0.14657273	-3.98730380	0.64314411
C	1.18422350	-4.00486548	0.20788748
C	1.86123414	-2.78883862	-0.19905859
C	1.12535214	-1.57162601	-0.21493137
C	-0.27051072	-1.59862355	0.17694450
C	-0.87829635	-2.77680157	0.62774509
C	3.28055581	-2.76020220	-0.20268316
C	1.81017205	-0.32817828	-0.23461949
C	3.23232008	-0.29948328	-0.23821773
C	3.96675538	-1.51426578	-0.22215140
C	3.90328970	0.93204919	0.13030613
C	3.18275633	2.05797932	0.54746569
C	1.76847341	2.02947985	0.55094140
C	1.09188056	0.87530744	0.13734563
C	-0.24028285	0.81120208	0.51613094
C	-0.92347552	-0.42949228	0.53620047
C	1.91188836	-5.11914954	0.59670987
C	3.32807824	-5.09057563	0.59319963
C	4.00817317	-3.94788790	0.20065265
C	5.33943120	-3.87666907	0.62905430

C	6.02167376	-2.63765358	0.60996329
C	5.36455474	-1.48494150	0.16252732
C	5.23885212	0.92169268	0.50233523
C	5.97163365	-0.29040080	0.51861845
C	5.92068208	-4.85156613	1.44645372
C	5.18213374	-5.99346904	1.84871633
C	3.85651352	-6.09482214	1.42151968
H	5.60239186	-6.69662511	2.56172418
S	7.45831490	-4.21330074	2.09306783
C	7.16691112	-2.58822724	1.41152617
C	7.74219728	-1.34445761	1.77664394
C	7.11349454	-0.18045183	1.32966986
H	8.57032866	-1.30670469	2.47820283
C	5.85719462	1.89755588	1.30176788
C	5.11352194	3.00354824	1.71850569
C	3.74926231	3.06474137	1.33611034
H	5.52367818	3.73300456	2.41066484
C	1.16576643	3.01270909	1.34243606
C	-0.19299227	2.89657364	1.73175830
C	-0.89352378	1.76141133	1.31874495
H	-0.62878437	3.60894355	2.42606143
C	-2.02041343	-2.77368258	1.43522928
C	-2.06465634	-0.36570742	1.35312679
C	-2.64354358	-1.55408604	1.80331743
H	-3.46901523	-1.54964710	2.50900659
C	0.10216651	-6.09615747	1.86146420
C	1.42858952	-6.14388116	1.42752233
C	-0.68387455	-4.98495392	1.46318625
H	-0.28557699	-6.81574635	2.57650008
C	2.66062835	-6.95686924	1.88730546
H	2.66619287	-7.09883294	2.97259194
H	2.67965522	-7.95401349	1.43064450
C	7.20875449	1.30183476	1.75856379
H	7.34602868	1.39940473	2.84006848
H	8.05554445	1.80725164	1.27827174
C	-2.21760861	1.11163531	1.78236878
H	-3.08652763	1.58226287	1.30623065
H	-2.35335369	1.20389029	2.86452903
S	2.43785780	4.09098785	1.98220072
S	-2.24277679	-4.40910729	2.11759774

No imaginary frequency.

Supplementary Table 19. Cartesian coordinates of **1a'** (transition state) optimized structure (optimized by M062x/6-31G(d)).

Symbol	X	Y	Z
C	-0.18143664	-4.00099031	1.26036257
C	1.19818851	-3.96144763	1.25597974
C	1.88234151	-2.73356133	1.23723265
C	1.16727543	-1.55092661	1.22321513
C	-0.23797013	-1.58615446	1.22674290

C	-0.91410519	-2.78929389	1.24485989
C	3.26452786	-2.70566567	1.23378589
C	1.83417556	-0.34006900	1.20575387
C	3.21604151	-0.31220507	1.20226894
C	3.93128568	-1.49510880	1.21630270
C	3.88810188	0.92233209	1.18342059
C	3.18414070	2.10936509	1.16768630
C	1.76838419	2.08077992	1.17120139
C	1.11284983	0.86633191	1.19036222
C	-0.25815709	0.82945842	1.19314553
C	-0.93961134	-0.40783591	1.21192394
C	1.91562831	-5.13015748	1.27133201
C	3.32799234	-5.10162777	1.26782736
C	3.99767016	-3.90491197	1.24904194
C	5.37779623	-3.88886014	1.24657626
C	6.06088108	-2.64857559	1.22759906
C	5.33683162	-1.47372966	1.21286641
C	5.25947220	0.94072009	1.17937926
C	5.99037933	-0.26809769	1.19467278
C	6.11006134	-5.06222737	1.26284586
C	5.38083387	-6.32369866	1.28316377
C	3.93994399	-6.34196898	1.28581743
H	5.94131915	-7.25315135	1.29659388
S	7.87709720	-4.45688817	1.25103841
C	7.44417137	-2.64000682	1.22499259
C	8.12062727	-1.34939541	1.20499496
C	7.36577027	-0.12192900	1.18901595
H	9.20584085	-1.32618587	1.20227615
C	6.02781582	2.09065202	1.16013233
C	5.29153624	3.32934129	1.14321257
C	3.83438090	3.33009848	1.14712162
H	5.81629658	4.27937578	1.12704196
C	1.06921960	3.27422634	1.15397109
C	-0.38678959	3.21470039	1.15726909
C	-1.07247050	1.94741739	1.17772952
H	-0.94959099	4.14274842	1.14376934
C	-2.29659708	-2.83658953	1.24904663
C	-2.31979390	-0.31750219	1.21298533
C	-3.02446998	-1.57431757	1.23251755
H	-4.10974401	-1.59474229	1.23510543
C	-0.08590220	-6.43417864	1.29680710
C	1.35458732	-6.39423576	1.29226992
C	-0.86553928	-5.20309211	1.28019503
H	-0.60822217	-7.38555610	1.31296879
C	2.66706315	-7.34288393	1.30384812
H	2.68213172	-7.97733131	2.19470754
H	2.67824914	-8.00414335	0.43262524
C	7.53122256	1.48880323	1.16481232
H	8.08836831	1.83720212	2.03920670
H	8.08309220	1.81085385	0.27705405
C	-2.55035774	1.28533556	1.18980905

H	-3.11927885	1.58475045	0.30490185
H	-3.11668211	1.61086022	2.06711854
S	2.42612729	4.55758964	1.13100079
S	-2.65568227	-4.66950191	1.27731143

Only one imaginary frequency (-91.22).

Supplementary Table 20. Cartesian coordinates of **1b'** (ground state) optimized structure (M062x/6-31G(d)).

Symbol	X	Y	Z
C	-0.15674841	-3.99605710	0.71961703
C	1.17889220	-4.00008832	0.29513910
C	1.86196879	-2.78418058	-0.09110142
C	1.12857042	-1.57107095	-0.10696574
C	-0.26981570	-1.60385428	0.26410568
C	-0.89173957	-2.78027899	0.70383684
C	3.28023633	-2.75556151	-0.09464221
C	1.81288408	-0.32856038	-0.12656500
C	3.23024262	-0.29996509	-0.13020116
C	3.96413204	-1.51383367	-0.11413234
C	3.89765639	0.93613149	0.21743403
C	3.18580869	2.07294943	0.62390918
C	1.76532226	2.04424464	0.62768296
C	1.09787972	0.87957086	0.22477556
C	-0.24539438	0.81494332	0.56014565
C	-0.92889260	-0.42616818	0.57989340
C	1.91183099	-5.12455443	0.64081244
C	3.32851800	-5.09596256	0.63743895
C	4.01374535	-3.94286276	0.28815147
C	5.35020165	-3.88485936	0.70609014
C	6.03554297	-2.64045826	0.68652317
C	5.36457463	-1.49016201	0.24977711
C	5.24420574	0.92578909	0.54575917
C	5.97727637	-0.28674493	0.56203480
C	5.93512190	-4.91832652	1.44667429
C	5.19551476	-6.07367077	1.79792387
C	3.85822736	-6.14677486	1.39927519
H	5.63126711	-6.82438061	2.45068987
C	7.23149784	-2.56458665	1.40958344
C	7.81702564	-1.31398437	1.72304387
C	7.15853546	-0.15450564	1.30515006
H	8.69309540	-1.26546472	2.36313094
C	5.90139574	1.92491589	1.27722613
C	5.17582632	3.05450158	1.66471266
C	3.80012386	3.11070758	1.33403422
H	5.61828909	3.82005157	2.29534610
C	1.11328868	3.05635501	1.34132812
C	-0.25725932	2.94461614	1.67929716

C	-0.93864572	1.78665318	1.29528355
H	-0.72703079	3.69157605	2.31246343
C	-2.08611403	-2.75290834	1.43279855
C	-2.11073143	-0.34182508	1.32900697
C	-2.71990334	-1.52694758	1.74972244
H	-3.59400777	-1.51387298	2.39419316
C	0.09140231	-6.17703523	1.80990775
C	1.42862743	-6.19595194	1.40488770
C	-0.69590837	-5.05245158	1.46266341
H	-0.31047198	-6.94497931	2.46435946
C	2.66177166	-7.02910373	1.82678635
H	2.66812087	-7.21893961	2.90480735
H	2.68047429	-8.00521160	1.32697895
C	7.27015222	1.33809659	1.69537125
H	7.44817094	1.46012619	2.76850719
H	8.09834716	1.83178833	1.17259066
C	-2.28043165	1.14500894	1.72030583
H	-3.13064791	1.60490121	1.20205243
H	-2.45762934	1.25955845	2.79440024
Se	2.43406960	4.28938190	1.96741428
Se	-2.41382582	-4.51230421	2.10566986
Se	7.63268537	-4.30922130	2.08170474

No imaginary frequency.

Supplementary Table 21. Cartesian coordinates of **1b'** (transition state) optimized structure (M062x/6-31G(d)).

Symbol	X	Y	Z
C	-0.19606449	-4.01496654	1.26184740
C	1.18827949	-3.96364344	1.25773082
C	1.88019095	-2.73582155	1.23847747
C	1.16423090	-1.55176139	1.22349233
C	-0.24457320	-1.59388317	1.22749251
C	-0.93333986	-2.79571103	1.24617239
C	3.26676951	-2.70785699	1.23466184
C	1.83326219	-0.33708414	1.20459099
C	3.21682422	-0.30920379	1.20072585
C	3.93436074	-1.49584930	1.21581097
C	3.88468578	0.93183831	1.18132388
C	3.18813756	2.12916730	1.16585400
C	1.76354963	2.10043930	1.16972530
C	1.11587465	0.87600633	1.18894683
C	-0.25715119	0.82932828	1.19284249
C	-0.93898794	-0.40860140	1.21219923
C	1.91523569	-5.12920432	1.27267626
C	3.32834354	-5.10066885	1.26894085
C	4.00765740	-3.90673143	1.25015043
C	5.39299277	-3.90224902	1.24703265

C	6.08038481	-2.65422605	1.22743802
C	5.34373399	-1.48120538	1.21225986
C	5.25848063	0.94051530	1.17785179
C	5.98979215	-0.26891876	1.19345521
C	6.09305533	-5.10169146	1.26301442
C	5.35807872	-6.34735024	1.28273032
C	3.92532228	-6.34914124	1.28581763
H	5.90957079	-7.28242422	1.29528148
C	7.46847918	-2.60452877	1.22373035
C	8.12849614	-1.31750524	1.20370532
C	7.36404441	-0.10570716	1.18794377
H	9.21363923	-1.28373748	1.20082040
C	6.04128369	2.08159415	1.15959849
C	5.32328246	3.32146002	1.14344924
C	3.87695106	3.33512071	1.14677447
H	5.85738396	4.26653999	1.12842736
C	1.02641316	3.27756726	1.15441692
C	-0.41829743	3.20557184	1.15872654
C	-1.08560244	1.93782614	1.17861712
H	-0.99025068	4.12827903	1.14654752
C	-2.32230181	-2.80215744	1.24942326
C	-2.31871495	-0.30121292	1.21360607
C	-3.03359231	-1.54278008	1.23295958
H	-4.11922934	-1.55264230	1.23542382
C	-0.06217020	-6.45688723	1.29663221
C	1.36951565	-6.40081539	1.29243636
C	-0.84693577	-5.24181523	1.28094144
H	-0.57533391	-7.41349676	1.31172012
C	2.66708914	-7.34501980	1.30310195
H	2.68226382	-7.98168163	2.19290810
H	2.67821313	-8.00704319	0.43192183
C	7.53301187	1.48992369	1.16441906
H	8.09115924	1.83879179	2.03852323
H	8.08641131	1.81306818	0.27748289
C	-2.55219601	1.28638806	1.19112786
H	-3.12285517	1.58681964	0.30715463
H	-3.11936614	1.61237965	2.06825224
Se	2.42392373	4.68070745	1.13096082
Se	-2.76111384	-4.73339522	1.27797022
Se	7.98501983	-4.51651445	1.25054786

Only one imaginary frequency (-74.85).

Supplementary Table 22. Cartesian coordinates of **1a-Me@C_{60}** optimized structure (optimized by B3LYP-D_3/6-311G(d,p)).

Symbol	X	Y	Z
S	-1.53473067	4.71830160	3.01122629
C	-2.28662195	4.54489654	1.38913437

C	-2.19717115	3.14781977	3.57840449
C	-3.08881633	3.39618472	1.39290962
C	-1.97062298	5.20984431	0.17228668
C	-3.04081804	2.63797127	2.58172973
C	-1.79466980	2.33971455	4.67688335
C	-3.56707801	2.81293484	0.20869241
C	-2.46148543	4.66314815	-1.01431408
H	-1.27669201	6.04213980	0.16571279
C	-3.48123029	1.30504740	2.58399607
C	-2.24549607	1.01837605	4.71265937
H	-1.06976769	2.70852565	5.39296896
C	-3.94398142	1.41648982	0.15971063
C	-3.24987997	3.49932400	-0.95646292
C	-2.14686887	4.92082356	-2.52434266
C	-3.90577814	0.65353037	1.36397656
C	-3.08297977	0.55412879	3.68270502
C	-1.84304685	-0.22880828	5.56679517
C	-3.97377931	0.75445072	-1.10088676
C	-3.27212411	2.84054644	-2.20621654
C	-2.49385846	3.53075017	-3.15423520
C	-0.71149018	5.38413472	-2.78267654
C	-3.13673580	5.97897503	-3.07309516
C	-3.90497525	-0.76927597	1.30614997
C	-3.08261330	-0.85736403	3.62547567
C	-2.24513975	-1.40283789	4.61472668
C	-0.36381580	-0.24557353	5.96078006
C	-2.72219790	-0.28003182	6.83994467
C	-3.97249634	-0.66990287	-1.15882581
C	-3.61869180	1.49858824	-2.29027131
C	-2.04682785	2.85091273	-4.28850932
H	-0.53621031	5.50513633	-3.85512918
H	0.01652610	4.67569718	-2.39655533
H	-0.53401196	6.35301095	-2.30800711
H	-2.97505454	6.94060122	-2.57872868
H	-4.17043975	5.67266702	-2.90149821
H	-2.99026526	6.11215545	-4.14833050
C	-3.94157408	-1.43192743	0.04387912
C	-3.47985214	-1.51704034	2.46934275
C	-1.79277329	-2.71637429	4.47203283
H	-0.13133452	-1.14917030	6.53073534
H	-0.12736719	0.61416742	6.59341972
H	0.28549075	-0.21645693	5.09023374
H	-3.78306874	-0.26877881	6.58239722
H	-2.51320335	0.58186749	7.47934530
H	-2.51441513	-1.19131910	7.40714896
C	-3.61479088	-1.31425665	-2.40442993
C	-3.19049858	0.84376013	-3.45620573
H	-1.36289496	3.31291202	-4.99069975
C	-2.39976200	1.47881248	-4.42280460
C	-3.56194436	-2.82694044	-0.02056630
C	-3.03815779	-2.84503864	2.35927373

H	-1.06756852	-3.14121114	5.15606459
C	-2.19424789	-3.43314347	3.31162866
C	-3.18793160	-0.56580756	-3.51305271
C	-3.26461710	-2.65774673	-2.42939981
S	-1.72859755	0.22384339	-5.51951016
C	-3.24244449	-3.41587354	-1.23734247
C	-3.08475295	-3.50443840	1.11283695
S	-1.53076653	-4.95238115	2.61983528
C	-2.39431784	-1.11807086	-4.52706642
C	-2.48284164	-3.26625650	-3.42890655
C	-2.45224454	-4.56996885	-1.38852382
C	-2.28189859	-4.64867631	1.01664463
C	-2.03708483	-2.49535443	-4.50398831
C	-2.13143597	-4.70167085	-2.91317057
C	-1.96353339	-5.21187080	-0.24973328
H	-1.35073444	-2.89664233	-5.24030928
C	-0.69200485	-5.13464465	-3.20055594
C	-3.11258599	-5.71642152	-3.55173673
H	-1.26836637	-6.03992620	-0.32302705
H	-0.50767076	-5.15893680	-4.27798797
H	-0.51416224	-6.14145007	-2.81276995
H	0.03048814	-4.46217631	-2.74621290
H	-4.14876768	-5.43009376	-3.36162414
H	-2.94841524	-6.71432482	-3.13640486
H	-2.95983834	-5.76093924	-4.63341059
C	2.86303383	2.93818622	1.91854429
C	4.00594072	2.17457039	2.38634585
C	3.17640294	3.44909365	0.59562679
C	1.57145301	2.50674205	2.20943146
C	5.02507765	2.21338853	1.35321157
C	3.81161604	1.01213345	3.12807940
C	4.51217666	3.00041077	0.24617550
C	2.18469298	3.50628742	-0.38032463
C	1.36917315	1.29327968	2.98101833
C	0.53736400	2.56809778	1.19248746
C	5.80755965	1.08911075	1.10431683
C	2.46530412	0.56274974	3.43149367
C	4.62723490	-0.16065201	2.86786306
C	4.80378526	2.63079659	-1.06431298
C	2.48890459	3.11830138	-1.74670777
C	0.83800246	3.05590896	-0.07533027
C	0.21025575	0.60505517	2.44003444
C	-0.30403661	1.39342335	1.33609112
C	6.11100999	0.70365263	-0.26194295
C	5.60478888	-0.12269973	1.87754235
C	2.44952885	-0.88842376	3.36041247
C	3.78608771	-1.33526004	3.01306829
C	3.77108099	2.69173502	-2.08242005
C	5.61953451	1.45808885	-1.32346824
C	1.33170578	2.42717346	-2.28500047
C	0.31272378	2.38805806	-1.25173603

C	0.19536698	-0.78408085	2.37160318
C	-0.80692197	0.75593345	0.20668481
C	6.09519537	-0.74620957	-0.33314333
C	5.78200436	-1.25702757	0.98913332
C	1.33834084	-1.54716550	2.84153244
C	3.95570051	-2.42321232	2.16049863
C	3.94800785	1.55746574	-2.97125779
C	5.09081516	0.79486765	-2.50177697
C	1.49882571	1.34393752	-3.14220058
C	-0.49447862	1.26573220	-1.11584601
C	-0.33465767	-1.44932710	1.19643400
C	-0.82240231	-0.69360969	0.13540932
C	5.58834369	-1.38205234	-1.46290107
C	4.97498830	-2.38303283	1.12755148
C	1.51494684	-2.68342953	1.95459399
C	2.79706279	-3.11246808	1.62151237
C	2.83641771	0.89813721	-3.48981068
C	5.07574226	-0.59535303	-2.57021124
C	0.65568678	0.17071642	-2.99887703
C	-0.31730518	0.13273732	-2.00558763
C	0.48106299	-2.62203904	0.93758987
C	-0.51980220	-1.07790178	-1.23095339
C	4.74687739	-2.55634739	-1.31886925
C	4.44636567	-3.04674931	-0.05067534
C	3.10061769	-3.49828231	0.25453534
C	2.82057616	-0.55232829	-3.56104304
C	3.91714485	-1.28326800	-3.11101167
C	1.47318142	-1.00085787	-3.25742062
C	0.77234404	-2.98920793	-0.37227101
C	0.26219720	-2.19919050	-1.47769083
C	3.71436991	-2.49480379	-2.33713321
C	2.10899887	-3.43695962	-0.72132620
C	1.28092835	-2.15938180	-2.51115570
C	2.42285233	-2.92424399	-2.04372093

No imaginary frequency.

Supplementary Table 23. Cartesian coordinates of **1a-Me@C_{70}** (upright) optimized structure (optimized by B3LYP-D_3/6-311G(d,p)).

Symbol	X	Y	Z
S	2.72575100	-0.02699400	-5.38940500
C	1.33943100	-0.72239000	0.21276300
C	1.33913000	0.72743000	0.20529300
C	1.14483300	1.16732600	-1.16250900
C	1.02535000	-0.00895600	-2.00167300
C	1.14535500	-1.17647200	-1.15039200
C	-2.00712800	-0.73509800	-3.74306600
C	-0.69748200	-1.17817600	-3.31241400

C	0.12591400	-0.01464600	-3.06495800
C	-0.58581900	-2.29046200	-2.49495700
C	-1.77275700	-3.00931000	-2.08417900
C	-1.57881800	-3.44746800	-0.73318300
C	-0.26976900	-3.00311600	-0.30271500
C	0.35625300	-2.29449800	-1.39697000
C	-4.15692000	-3.00977100	-1.74458100
C	-3.17956000	-1.46865700	-3.42720700
C	-3.05782900	-2.65195300	-2.56580700
C	3.77628700	1.42544000	2.78011000
C	4.33045100	-1.41879000	-2.05536200
C	4.58090700	-0.71784300	-0.81387000
C	3.84832800	-3.46461500	-0.83036800
C	4.44392000	-1.42445800	0.41534000
C	4.30148500	-0.70495700	1.63871100
C	4.00450800	-0.72214300	-3.23026300
C	4.58134200	0.70786400	-0.82089300
C	4.06482700	-2.82112400	0.38161400
C	3.06671100	-4.62257000	-0.99715800
C	4.00493300	0.68864300	-3.23721300
C	4.33129500	1.39666000	-2.06924900
C	4.44485500	1.42665400	0.40129400
C	3.85046600	3.45478300	-0.86444700
C	4.06670700	2.82318700	0.35383500
C	3.30012700	-1.32067900	-4.28334400
C	4.30194200	0.71930900	1.63169000
C	3.97796200	-2.76207300	-2.04938100
C	3.28304400	-3.41552100	-3.08428800
C	1.46655700	-5.26522500	-2.92855700
H	1.37957600	-5.34493800	-4.01559200
H	0.71231800	-4.56620300	-2.57665500
H	1.24564300	-6.24971700	-2.50710800
C	2.93613500	-2.69507300	-4.22832300
H	2.31474300	-3.12966500	-5.00250400
C	2.93771600	2.65225300	-4.25469700
H	2.31652500	3.07947700	-5.03313100
C	3.30099700	1.27719000	-4.29620600
C	2.87869700	-4.82418500	-2.53603700
H	3.84822200	5.92201300	-4.18573500
H	4.92425500	5.55043500	-2.82748300
C	3.97952200	2.74013500	-2.07647300
C	3.90460300	-5.87058000	-3.03882000
H	3.69715100	-6.84777100	-2.59452700
H	4.92224600	-5.57936700	-2.77139800
H	3.84711000	-5.96433900	-4.12661100
C	3.28484300	3.38370700	-3.11773500
C	0.74062400	-1.41071300	1.26466800
C	0.10818400	-0.67906100	2.33957500
C	-0.08294300	-2.57039700	0.99957500
C	0.10793900	0.70547700	2.33249800
C	-1.08156400	-1.39736100	2.74649300

C	-1.19914400	-2.56275800	1.92117200
C	-1.08215900	1.42739100	2.73194800
C	0.74009700	1.42630400	1.25010000
C	-2.22938400	-0.71740100	3.22712100
C	-2.46911700	-3.07658700	1.55365300
C	-1.20026500	2.58414800	1.89459600
C	-2.22968500	0.75193500	3.21956400
C	-0.08404500	2.58279300	0.97299500
C	-3.46626200	-1.39652300	3.08832100
C	-3.58461800	-2.56025200	2.26132000
C	-2.66678700	-3.53771200	0.17277700
C	-2.47047300	3.09358400	1.52177900
C	-3.46687000	1.42902000	3.07369300
C	-0.27113400	3.00190300	-0.33371200
C	-4.72108900	-0.67709000	3.03069700
C	-4.91371300	-2.56574500	1.68765200
C	-3.96383400	-3.44585300	-0.39344200
C	-3.58574400	2.58407300	2.23471000
C	-2.66836800	3.54037700	0.13623900
C	-4.72138000	0.70848500	3.02351600
C	0.35522500	2.28239700	-1.42067200
C	-1.58036100	3.44122100	-0.76874400
C	-5.63013600	-1.40624700	2.17336500
C	-5.09981500	-3.00042100	0.38544000
C	-4.91485400	2.58310100	1.66106100
C	-3.96535900	3.44208800	-0.42901300
C	-5.63074500	1.42835500	2.15873200
C	-0.58681700	2.26666600	-2.51855800
C	-1.77410000	2.98909200	-2.11515100
C	-6.50291500	-0.71858400	1.33228700
C	-6.01069200	-2.29558600	-0.49029000
C	-5.41258500	-2.29285800	-1.80754900
C	-5.10115600	3.00420900	0.35442700
C	-4.15825600	2.99199600	-1.77556100
C	-6.50322900	0.73167400	1.32478900
C	-0.69798800	1.14588100	-3.32444500
C	-3.05901000	2.62622300	-2.59308500
C	-6.69799200	-1.17381400	-0.03064900
C	-5.52821500	-1.17639500	-2.61988700
C	-6.01175700	2.29003000	-0.51398400
C	-5.41361800	2.27393900	-1.83114500
C	-6.69853700	1.17272800	-0.04277000
C	-2.00745100	0.69776800	-3.75043300
C	-3.18020600	1.43402500	-3.44217200
C	-6.81887200	-0.00493700	-0.88062900
C	-4.39217300	-0.73361500	-3.39999000
C	-6.24683900	-0.01137700	-2.15132000
C	-5.52869600	1.14904800	-2.63184600
C	-4.39247000	0.69874000	-3.40733700
C	3.28319800	0.72492400	3.87366100
C	3.33973400	2.75599200	2.67902400

C	3.28273400	-0.68781400	3.88062700
C	2.36119300	1.23447500	4.80556200
C	3.48975500	3.45883400	1.46494700
C	2.41303800	3.30832400	3.57427500
C	3.77536500	-1.39939100	2.79403100
C	2.36036300	-1.18756300	4.81747200
C	1.88288300	0.02787300	5.67763200
C	1.91606500	2.55084500	4.66980600
C	2.69042800	4.60286700	1.33957200
S	1.80107300	4.84731700	2.88040700
C	3.33785900	-2.73055400	2.70599300
C	1.91437800	-2.50493000	4.69466300
C	0.37512300	0.02975500	5.94178700
C	2.64858500	0.03419800	7.02292300
H	1.13216400	2.95006200	5.30261800
C	2.47912200	5.20993000	0.07127100
C	3.48746100	-3.44542700	1.49890900
C	2.41083000	-3.27347200	3.60663300
H	1.13018500	-2.89735700	5.33134600
H	0.08799000	-0.84974600	6.52434900
H	-0.19371000	0.02564100	5.01629700
H	0.08875600	0.91495100	6.51603400
H	2.38923800	0.92457700	7.60205700
H	3.72779600	0.03287900	6.85771300
H	2.38842300	-0.85015000	7.61086800
C	3.06940000	4.61142100	-1.04260100
H	1.78804800	6.03815400	-0.03272600
C	2.68760400	-4.59027000	1.38482500
S	1.79796000	-4.81889000	2.92792000
C	2.88085100	4.79774800	-2.58328500
C	2.47615600	-5.20979200	0.12256100
C	1.46841000	5.23479300	-2.97914200
C	3.90645400	5.83906200	-3.09710500
H	1.78474100	-6.03871800	0.02673300
H	0.71439400	4.54040200	-2.61764500
H	1.37984600	5.30163600	-4.06691200
H	1.24856200	6.22430800	-2.56903400
H	3.69931700	6.82060900	-2.66237900

No imaginary frequency.

Supplementary Table 24. Cartesian coordinates of **1a-Me@C$_{70}$** (latericumbent) optimized structure (optimized by B3LYP-D$_3$/6-311G(d,p)).

Symbol	X	Y	Z
S	2.38822224	-1.56695901	-5.14239634
S	2.68724635	-3.09401110	4.30588934
C	-1.76943398	-2.88934436	-2.98817318
C	-2.13139408	-1.58738228	-3.50813022

C	-1.00731506	-0.71470815	-3.33331521
C	0.05602007	-1.47025614	-2.70653816
C	-0.40779289	-2.82506426	-2.50165415
C	-2.71279100	-3.65027547	-2.31494113
C	-2.33786893	-4.38438650	-1.12482405
C	-3.45104101	-4.29850456	-0.20455998
C	-4.50972714	-3.53932457	-0.83540503
C	-4.05522813	-3.13696551	-2.13470612
C	-0.04923382	-3.51749429	-1.34793106
C	-1.03080284	-4.31897641	-0.64563102
C	-0.78734883	-4.17570339	0.77754909
C	0.34873820	-3.29003425	0.95266510
C	0.80321921	-2.88154220	-0.36090499
C	1.25820916	-1.58437308	-0.56834001
C	0.86978809	-0.86406205	-1.76198309
C	0.65661698	0.52361304	-1.40810007
C	0.89932400	0.66798607	-0.00217097
C	1.26129310	-0.63258601	0.52253107
C	0.82262910	-1.01885306	1.77903816
C	0.35989014	-2.37303419	2.00050818
C	-0.76645195	-2.29537425	2.90641624
C	-0.98040704	-0.90662416	3.25395226
C	-0.00361002	-0.12012105	2.55732121
C	-1.49109225	1.77339400	2.61043322
C	-2.46737926	0.98920088	3.30694727
C	-3.77311740	1.54614184	3.02674325
C	-3.60601345	2.69465994	2.16329619
C	-2.18762036	2.82427203	1.90090717
C	-1.74637635	3.20667709	0.64581708
C	-0.59175023	2.55850311	0.06047704
C	-0.83359924	2.41302408	-1.34111006
C	-2.13547136	2.97502304	-1.63434008
C	-2.70360344	3.48385705	-0.40473400
C	-4.07122953	3.35346496	-0.16180898
C	-4.92413355	2.71889386	-1.15062205
C	-5.91247555	1.92447374	-0.44710100
C	-5.67146457	2.06714576	0.97663210
C	-4.53439154	2.95135690	1.15285311
C	-5.83069449	0.96803267	1.81901316
C	-5.52551035	-1.35740947	1.99590518
C	-4.68259832	-0.73128738	2.97018725
C	-4.86115742	0.70204071	2.85985224
C	-4.03951712	-3.25807552	1.93808517
C	-3.19594710	-2.62812642	2.91133124
C	-1.85250197	-3.14112138	2.74161123
C	-1.86402091	-4.10259545	1.66006315
C	-3.21997901	-4.16033553	1.15605512
C	-5.54133237	-1.23432847	-2.07807912
C	-5.71974544	0.19741963	-2.19292513
C	-6.30396453	0.69010963	-0.96339404
C	-3.61551731	0.31076176	-3.44542922

C	-2.49205728	1.18109790	-3.26923120
C	-2.95036738	2.36981795	-2.58013215
C	-4.37138948	2.24164786	-2.33772013
C	-4.77455943	0.95570574	-2.86411517
C	0.17061589	1.60268709	0.77737909
C	-0.30123412	1.19003203	2.10518518
C	-2.28112317	-0.40253021	3.51455929
C	-3.43408319	-1.29664334	3.33929727
C	-5.14182124	-2.56929754	1.36461913
C	-4.46186923	-1.90421745	-2.71227916
C	-3.46093221	-1.09952633	-3.42574321
C	-1.18543015	0.66634393	-3.06851619
C	-0.32224813	1.30767804	-2.06676311
C	3.54967122	0.11786619	-3.53595122
C	4.17676620	1.12498730	-1.35820407
C	4.49410130	-0.16897578	-0.85312503
C	3.75040117	1.24991428	-2.73346016
C	3.13062205	2.40816933	-3.17966020
C	3.87072332	-1.16294289	-3.03920119
C	4.38764637	-1.30520586	-1.74090609
C	3.91317112	2.19578636	-0.46037500
C	4.47500540	-1.75237289	1.04617511
C	2.30014199	2.49347228	-4.31080228
C	4.53538832	-0.39478979	0.55141907
C	2.59028990	4.26543943	-0.09241297
C	2.70341316	0.13541114	-4.65225530
C	4.37119144	-2.59721496	-1.23269705
C	3.25366900	3.38249740	-0.95734504
C	4.04432139	-2.03135094	2.35331220
C	3.50956842	-3.27378306	2.71673223
C	1.59174977	5.14115743	-0.53843001
C	2.89022797	3.48077339	-2.29346113
C	1.67929585	3.93241535	-4.29490128
C	1.24052074	5.20393241	-1.91744311
H	0.40440165	5.81709939	-2.24509813
C	3.29236335	-2.23111900	-3.73492124
C	2.07055104	1.34211218	-5.06873333
H	1.35601799	1.33573414	-5.88818340
C	3.83303947	-3.71259307	-1.90173310
C	1.90097784	4.34264839	-2.79820917
C	0.22101674	3.98871926	-4.75054931
H	0.13673276	3.67729023	-5.79789841
H	-0.16393435	5.01205731	-4.67630730
H	-0.41381626	3.34152718	-4.15100127
C	3.83401955	-4.88615015	-0.86457003
C	2.53537886	4.85578747	-5.19871934
H	2.17892777	5.88972553	-5.12902533
H	2.46633587	4.53329644	-6.24370743
H	3.58726493	4.83182653	-4.89822332
C	4.41622046	-2.82145997	0.16309104
C	3.27811242	-3.53981510	-3.17226119

H	2.75951743	-4.35070318	-3.67782623
C	2.61798152	-5.80931731	-0.94750404
H	2.57125254	-6.31527035	-1.91885510
H	1.71369641	-5.21805431	-0.82226203
H	2.66252756	-6.58417436	-0.17442998
C	3.44782048	-4.34083014	1.77716416
H	2.95637950	-5.27550924	2.03597418
C	3.91423351	-4.09657809	0.48374407
C	5.14005469	-5.70213516	-1.04342604
H	5.11949172	-6.24561416	-1.99481311
H	5.24692474	-6.42816719	-0.22975898
H	6.01590171	-5.04641403	-1.03852504
C	-5.38624624	-2.71852356	-0.07670097
C	-6.22810946	-0.31316744	1.27597312
C	-6.46171645	-0.44777547	-0.08451097
C	-5.99975037	-1.63067452	-0.77988803
C	4.25240124	0.67363327	1.45036114
C	3.90510723	0.35634322	2.81672123
C	3.94537014	1.96760734	0.94458110
C	3.29930113	1.30615425	3.62779229
C	3.75519830	-0.97285488	3.23952127
C	3.30190103	2.92550137	1.81401116
C	2.99279903	2.58981433	3.12423526
C	2.53594109	1.01841419	4.77150937
C	2.97728726	-1.31918095	4.35210635
C	2.60613991	4.02842441	1.29785413
C	2.01244892	3.22317432	3.90706631
C	1.87164996	2.36531425	5.21299841
C	2.36499616	-0.31483092	5.15438640
C	1.61556080	4.69581140	2.02998818
C	1.30896880	4.30367635	3.36512928
C	2.73519299	3.01741635	6.32445750
C	0.43515587	2.19750314	5.71360947
H	1.69754213	-0.58979798	5.96748045
S	0.72532567	5.78335842	0.90399110
H	0.48292971	4.76829433	3.89798231
H	2.83502603	2.33580730	7.17628856
H	2.26526089	3.94385238	6.67388451
H	3.73640904	3.25711642	5.95413646
H	0.43203490	1.72567111	6.70302349
H	-0.15535214	1.57103206	5.04472040
H	-0.05916723	3.17028019	5.80791346

No imaginary frequency.

10. References

(1) Maeda, H., Takashima, M., Sakata, K., Watanabe, T.; Honda, M., Segi, M. *Tetrahedron Lett.* **52**, 415–417 (2011).

(2) Rashidnadimi, S., Hung, T. H.; Wong, K. T.; Bard, A. J. *J. Am. Chem. Soc.* **130**, 634–639 (2008).

(3) Frisch, M. J., Trucks, G. W., Schlegel, H. B., Scuseria, G. E., Robb, M. A., Cheeseman, J. R., Scalmani, G., Barone, V., Mennucci, B., Petersson, G. A., Nakatsuji, H., Caricato, M., Li, X., Hratchian, H. P., Izmaylov, A. F., Bloino, J., Zheng, G., Sonnenberg, J. L., Hada, M., Ehara, M., Toyota, K., Fukuda, R., Hasegawa, J., Ishida, M., Nakajima, T., Honda, Y., Kitao, O., Nakai, H., Vreven, T., Montgomery, Jr., J. A., Peralta, J. E., Ogliaro, F., Bearpark, M., Heyd, J. J., Brothers, E., Kudin, K. N., Staroverov, V. N., Keith, T., Kobayashi, R., Normand, J., Raghavachari, K., Rendell, A., Burant, J. C., Iyengar, S. S., Tomasi, J., Cossi, M., Rega, N., Millam, J. M., Klene, M., Knox, J. E., Cross, J. B., Bakken, V., Adamo, C., Jaramillo, J., Gomperts, R., Stratmann, R. E., Yazyev, O., Austin, A. J., Cammi, R., Pomelli, C., Ochterski, J. W., Martin, R. L., Morokuma, K., Zakrzewski, V. G., Voth, G. A., Salvador, P., Dannenberg, J. J., Dapprich, S., Daniels, A. D., Farkas, O., Foresman, J. B., Ortiz, J. V., Cioslowski, J. & Fox, D. J. *Gaussian 09, Revision D.01*, Gaussian, Inc., Wallingford CT, 2010.

(4) Beutler, U., Fuenfschilling, P. C., Steinkemper, A. *Org. Process Res. Dev.* **11**, 341–345 (2007).

(5) Müller, P., Herbst-Irmer, R., Spek, A. L., Schneider, T. R., Sawaya, M. R. *Crystal Structure Refinement: A Crystallographer's Guide to SHELXL*, Oxford Univ. Press, New York, **2006**.

(6) Sheldrick, G. M. Acta Crystallogr. A **71**, 3 (2015).

(7) Dolomanov, O. V., Bourhis, L. J., Gildea, R. J., Howard, J. A. K., Puschmann, H. *J. Appl. Crystallogr.*, **42**, 339, (2009).

(8) Haddon, R. C. *J. Phys. Chem. A* **105**, 4164–4165 (2001).

(9) Goddard, R., Haenel, M. W., Herndon, W. C., Krüger, C., Zander, M. *J. Am. Chem. Soc.* **117**, 30–41, (1995).

(10) (a) Zheng, M., Bai, F., Li, F., Li, Y., Zhu, D. *J. Appl. Polym. Sci.* **70**, 599–603 (1998). (b) Yang, D., Li, M., Chen C. *Chem. Commun.* **53**, 9336–9339 (2017).

(11) Campbell, K., Zappas, A., Bunz, U., Thio, Y. S., Bucknall, D. G. *J. Photochem. Photobiol. A: Chem.* **249**, 41–46 (2012).

(12) Lu, T., Chen, F. *J. Comput. Chem.* **33**, 580–592 (2012).

(13) Lu, T., Chen, F. *J. Mol. Graph. Model.* **38**, 314–323 (2012).

(14) (a) Fallah-Bagher-Shaidaei, H., Wannere, S. S., Corminboeuf, C., Puchta, R.; Schleyer, P. v. R. *Org. Lett.* **8**, 863–866 (2006). (b) Reisi-Vanani, A., Rezaei, A. A. *J. Mol. Graphics Modell.* **61**, 85–88 (2015).

(15) Geuenich, D., Hess, K., Kohler, F., Herges, R. *Chem. Rev.* **105**, 3758–3772 (2005).

(16) Grimme, S. *J. Comput. Chem.* **27**, 1787–1799 (2006).

(17) Galano, A., Alvarez-Idaboy, J. R. *J. Comput. Chem.* **27**, 1203–1210 (2006).
