# Supplementary material for: Trichalcogenasupersumanenes and its concave-convex supramolecular assembly with fullerenes
Source: Nat Commun. 2023 Jun 10;14:3446. doi: 10.1038/s41467-023-39086-0 (PMC10257710; doi:10.1038/s41467-023-39086-0)
Supplement: Supplementary file 3 — Description of Additional Supplementary Files [file 41467_2023_39086_MOESM3_ESM.pdf]

Description of Additional Supplementary Files

File Name: Supplementary Data 1
Description: The CIF files of single crystals
