# Supplementary material for: Trichalcogenasupersumanenes and its concave-convex supramolecular assembly with fullerenes
Source: Nat Commun. 2023 Jun 10;14:3446. doi: 10.1038/s41467-023-39086-0 (PMC10257710; doi:10.1038/s41467-023-39086-0)

checkCIF/PLATON report

Structure factors have been supplied for datablock(s) z

THIS REPORT IS FOR GUIDANCE ONLY. IF USED AS PART OF A REVIEW PROCEDURE FOR PUBLICATION, IT SHOULD NOT REPLACE THE EXPERTISE OF AN EXPERIENCED CRYSTALLOGRAPHIC REFEREE.

No syntax errors found. CIF dictionary Interpreting this report

Datablock: z

Bond precision: C-C = 0.0031 A Wavelength=1.54178

Cell:	a=25.7504(12)	b=23.7050(11)	c=29.4867(13)
	alpha=90	beta=112.839(2)	gamma=90
Temperature:	153 K		

	Calculated	Reported
Volume	16587.9(13)	16587.9(13)
Space group	P 21/c	P 1 21/c 1
Hall group	-P 2ybc	-P 2ybc
Moiety formula	2(C69 H60 S3), C67.90 H57.24 S3, 3(C H Cl3), 0.552(C2 H5) [+ so	C69 H60 S3, C1 H1 Cl3
Sum formula	C210 H183 Cl9 S9 [+ solvent]	C70 H61 Cl3 S3
Mr	3314.15	1104.71
Dx,g cm-3	1.327	1.327
Z	4	12
Mu (mm-1)	2.891	2.891
F000	6960.0	6960.0
F000’	6999.80	
h,k,lmax	31,28,35	31,28,35
Nref	30459	30313
Tmin,Tmax	0.525,0.749	0.601,0.753
Tmin’	0.300	

Correction method= # Reported T Limits: Tmin=0.601 Tmax=0.753
AbsCorr = MULTI-SCAN

Data completeness= 0.995 Theta(max)= 68.349

R(reflections)= 0.0480(26370)

wR2(reflections)= 0.1428(30313)

S = 1.055 Npar= 2291

The following ALERTS were generated. Each ALERT has the format
test-name_ALERT_alert-type_alert-level.
Click on the hyperlinks for more details of the test.

Alert level C

```
DIFMX02_ALERT_1_C  The maximum difference density is > 0.1*ZMAX*0.75
            The relevant atom site should be identified.
PLAT094_ALERT_2_C Ratio of Maximum / Minimum Residual Density ....       2.35 Report
PLAT097_ALERT_2_C Large Reported Max.  (Positive) Residual Density       1.66 eA-3
PLAT213_ALERT_2_C Atom C1B             has ADP max/min Ratio .....        3.7 prolat
PLAT220_ALERT_2_C NonSolvent   Resd 1  C   Ueq(max)/Ueq(min) Range        5.3 Ratio
PLAT221_ALERT_2_C Solv./Anion  Resd 2  C   Ueq(max)/Ueq(min) Range        6.4 Ratio
PLAT221_ALERT_2_C Solv./Anion  Resd 3  C   Ueq(max)/Ueq(min) Range        6.3 Ratio
PLAT222_ALERT_3_C NonSolvent Resd 1  H   Uiso(max)/Uiso(min) Range        5.1 Ratio
PLAT223_ALERT_4_C Solv./Anion  Resd 2  H   Ueq(max)/Ueq(min) Range        5.8 Ratio
PLAT223_ALERT_4_C Solv./Anion  Resd 3  H   Ueq(max)/Ueq(min) Range        6.0 Ratio
PLAT234_ALERT_4_C Large Hirshfeld Difference C63      --C19A     .       0.25 Ang.
PLAT241_ALERT_2_C High   'MainMol' Ueq as Compared to Neighbors of       C199 Check
PLAT242_ALERT_2_C Low    'MainMol' Ueq as Compared to Neighbors of        C91 Check
PLAT242_ALERT_2_C Low    'MainMol' Ueq as Compared to Neighbors of        C11 Check
PLAT329_ALERT_4_C Carbon Atom Hybridisation Unclear for .........        C185 Check
PLAT329_ALERT_4_C Carbon Atom Hybridisation Unclear for .........        C205 Check
PLAT360_ALERT_2_C Short  C(sp3)-C(sp3) Bond  C199     - C203     .       1.37 Ang.
PLAT906_ALERT_3_C Large K Value in the Analysis of Variance ......      2.470 Check
PLAT911_ALERT_3_C Missing FCF Refl Between Thmin & STh/L=    0.600         51 Report
PLAT971_ALERT_2_C Check Calcd Resid. Dens.  1.64Ang From C51A            1.64 eA-3
```

Alert level G

```
PLAT002_ALERT_2_G Number of Distance or Angle Restraints on AtSite         54 Note
PLAT003_ALERT_2_G Number of Uiso or Uij Restrained non-H Atoms ...         53 Report
PLAT042_ALERT_1_G Calc. and Reported MoietyFormula Strings  Differ     Please Check
PLAT045_ALERT_1_G Calculated and Reported Z Differ by a Factor ...      0.333 Check
PLAT083_ALERT_2_G SHELXL Second Parameter in WGHT  Unusually Large       8.00 Why ?
PLAT172_ALERT_4_G The CIF-Embedded .res File Contains DFIX Records          6 Report
PLAT176_ALERT_4_G The CIF-Embedded .res File Contains SADI Records         38 Report
PLAT177_ALERT_4_G The CIF-Embedded .res File Contains DELU Records          8 Report
PLAT178_ALERT_4_G The CIF-Embedded .res File Contains SIMU Records          8 Report
PLAT186_ALERT_4_G The CIF-Embedded .res File Contains ISOR Records          1 Report
PLAT191_ALERT_3_G A Non-default SADI Restraint Value has been used     0.0400 Report
PLAT191_ALERT_3_G A Non-default SADI Restraint Value has been used     0.0400 Report
PLAT191_ALERT_3_G A Non-default SADI Restraint Value has been used     0.0400 Report
PLAT191_ALERT_3_G A Non-default SADI Restraint Value has been used     0.0400 Report
PLAT191_ALERT_3_G A Non-default SADI Restraint Value has been used     0.0400 Report
PLAT191_ALERT_3_G A Non-default SADI Restraint Value has been used     0.0400 Report
PLAT191_ALERT_3_G A Non-default SADI Restraint Value has been used     0.0400 Report
PLAT191_ALERT_3_G A Non-default SADI Restraint Value has been used     0.0400 Report
PLAT191_ALERT_3_G A Non-default SADI Restraint Value has been used     0.0400 Report
PLAT191_ALERT_3_G A Non-default SADI Restraint Value has been used     0.0400 Report
PLAT191_ALERT_3_G A Non-default SADI Restraint Value has been used     0.0400 Report
```

```
PLAT191_ALERT_3_G A Non-default SADI Restraint Value has been used     0.0400 Report
PLAT191_ALERT_3_G A Non-default SADI Restraint Value has been used     0.0400 Report
PLAT191_ALERT_3_G A Non-default SADI Restraint Value has been used     0.0400 Report
PLAT191_ALERT_3_G A Non-default SADI Restraint Value has been used     0.0400 Report
PLAT191_ALERT_3_G A Non-default SADI Restraint Value has been used     0.0400 Report
PLAT231_ALERT_4_G Hirshfeld Test (Solvent)   Cl6      --C51A     .        5.2 s.u.
PLAT231_ALERT_4_G Hirshfeld Test (Solvent)   Cl9      --C51      .        7.7 s.u.
PLAT301_ALERT_3_G Main Residue  Disorder .............(Resd  1  )         11% Note
PLAT302_ALERT_4_G Anion/Solvent/Minor-Residue Disorder (Resd  2  )          3% Note
PLAT302_ALERT_4_G Anion/Solvent/Minor-Residue Disorder (Resd  3  )          1% Note
PLAT302_ALERT_4_G Anion/Solvent/Minor-Residue Disorder (Resd  4  )         75% Note
PLAT302_ALERT_4_G Anion/Solvent/Minor-Residue Disorder (Resd  5  )        100% Note
PLAT302_ALERT_4_G Anion/Solvent/Minor-Residue Disorder (Resd  6  )        100% Note
PLAT302_ALERT_4_G Anion/Solvent/Minor-Residue Disorder (Resd  7  )        100% Note
PLAT302_ALERT_4_G Anion/Solvent/Minor-Residue Disorder (Resd  8  )        100% Note
PLAT302_ALERT_4_G Anion/Solvent/Minor-Residue Disorder (Resd  9  )        100% Note
PLAT304_ALERT_4_G Non-Integer Number of Atoms in ..... (Resd  3  )     128.14 Check
PLAT304_ALERT_4_G Non-Integer Number of Atoms in ..... (Resd  5  )       2.56 Check
PLAT304_ALERT_4_G Non-Integer Number of Atoms in ..... (Resd  6  )       3.20 Check
PLAT304_ALERT_4_G Non-Integer Number of Atoms in ..... (Resd  7  )       2.44 Check
PLAT304_ALERT_4_G Non-Integer Number of Atoms in ..... (Resd  8  )       1.80 Check
PLAT304_ALERT_4_G Non-Integer Number of Atoms in ..... (Resd  9  )       3.86 Check
PLAT333_ALERT_2_G Large Aver C6-Ring C-C Dist C1       -C46      .       1.42 Ang.
PLAT333_ALERT_2_G Large Aver C6-Ring C-C Dist C1       -C46      .       1.42 Ang.
PLAT333_ALERT_2_G Large Aver C6-Ring C-C Dist C6       -C50      .       1.42 Ang.
PLAT333_ALERT_2_G Large Aver C6-Ring C-C Dist C8       -C90      .       1.42 Ang.
PLAT333_ALERT_2_G Large Aver C6-Ring C-C Dist C16      -C46      .       1.42 Ang.
PLAT333_ALERT_2_G Large Aver C6-Ring C-C Dist C4       -C32      .       1.42 Ang.
PLAT333_ALERT_2_G Large Aver C6-Ring C-C Dist C4       -C54      .       1.43 Ang.
PLAT333_ALERT_2_G Large Aver C6-Ring C-C Dist C18      -C84      .       1.42 Ang.
PLAT333_ALERT_2_G Large Aver C6-Ring C-C Dist C22      -C58      .       1.42 Ang.
PLAT333_ALERT_2_G Large Aver C6-Ring C-C Dist C22      -C62      .       1.42 Ang.
PLAT333_ALERT_2_G Large Aver C6-Ring C-C Dist C26      -C150     .       1.42 Ang.
PLAT333_ALERT_2_G Large Aver C6-Ring C-C Dist C12      -C42      .       1.42 Ang.
PLAT333_ALERT_2_G Large Aver C6-Ring C-C Dist C12      -C134     .       1.42 Ang.
PLAT333_ALERT_2_G Large Aver C6-Ring C-C Dist C12      -C134     .       1.42 Ang.
PLAT333_ALERT_2_G Large Aver C6-Ring C-C Dist C34      -C76      .       1.43 Ang.
PLAT333_ALERT_2_G Large Aver C6-Ring C-C Dist C42      -C168     .       1.43 Ang.
PLAT367_ALERT_2_G Long?  C(sp?)-C(sp?) Bond  C171     - C185     .       1.61 Ang.
PLAT410_ALERT_2_G Short  Intra H...H Contact  H15B     ..H47D     .       2.14 Ang.
                                                     x,y,z  =      1_555 Check
PLAT413_ALERT_2_G Short Inter XH3 .. XHn     H18B     ..H1AC     .       1.84 Ang.
                                          1-x,-1/2+y,3/2-z  =      2_646 Check
PLAT432_ALERT_2_G Short Inter X...Y Contact  Cl5A     ..C191     .       3.18 Ang.
                                                -x,1-y,1-z  =      3_566 Check
PLAT432_ALERT_2_G Short Inter X...Y Contact  C48      ..C215     .       3.07 Ang.
                                                     x,y,z  =      1_555 Check
PLAT432_ALERT_2_G Short Inter X...Y Contact  C171     ..C205     .       2.66 Ang.
                                                     x,y,z  =      1_555 Check
PLAT432_ALERT_2_G Short Inter X...Y Contact  C185     ..C205     .       1.83 Ang.
                                                     x,y,z  =      1_555 Check
PLAT432_ALERT_2_G Short Inter X...Y Contact  C185     ..C210     .       2.56 Ang.
                                                     x,y,z  =      1_555 Check
PLAT605_ALERT_4_G Largest Solvent Accessible VOID in the Structure         39 A**3
PLAT720_ALERT_4_G Number of Unusual/Non-Standard Labels ..........          8 Note
PLAT773_ALERT_2_G Check long C-C Bond in CIF: C185     --C205            1.83 Ang.
PLAT790_ALERT_4_G Centre of Gravity not Within Unit Cell: Resd.  #          2 Note
```

```
              C69 H60 S3
PLAT860_ALERT_3_G Number of Least-Squares Restraints ............        717 Note
PLAT910_ALERT_3_G Missing # of FCF Reflection(s) Below Theta(Min).          1 Note
PLAT912_ALERT_4_G Missing # of FCF Reflections Above STh/L=  0.600         95 Note
PLAT913_ALERT_3_G Missing # of Very Strong Reflections in FCF ....          1 Note
PLAT933_ALERT_2_G Number of HKL-OMIT Records in Embedded .res File         46 Note
PLAT978_ALERT_2_G Number C-C Bonds with Positive Residual Density.          1 Info
```

```
   0 ALERT level A = Most likely a serious problem - resolve or explain
   0 ALERT level B = A potentially serious problem, consider carefully
  20 ALERT level C = Check. Ensure it is not caused by an omission or oversight
  77 ALERT level G = General information/check it is not something unexpected

   3 ALERT type 1 CIF construction/syntax error, inconsistent or missing data
  41 ALERT type 2 Indicator that the structure model may be wrong or deficient
  23 ALERT type 3 Indicator that the structure quality may be low
  30 ALERT type 4 Improvement, methodology, query or suggestion
   0 ALERT type 5 Informative message, check
```

It is advisable to attempt to resolve as many as possible of the alerts in all categories. Often the minor alerts point to easily fixed oversights, errors and omissions in your CIF or refinement strategy, so attention to these fine details can be worthwhile. In order to resolve some of the more serious problems it may be necessary to carry out additional measurements or structure refinements. However, the purpose of your study may justify the reported deviations and the more serious of these should normally be commented upon in the discussion or experimental section of a paper or in the "special_details" fields of the CIF. checkCIF was carefully designed to identify outliers and unusual parameters, but every test has its limitations and alerts that are not important in a particular case may appear. Conversely, the absence of alerts does not guarantee there are no aspects of the results needing attention. It is up to the individual to critically assess their own results and, if necessary, seek expert advice.

Publication of your CIF in IUCr journals

A basic structural check has been run on your CIF. These basic checks will be run on all CIFs submitted for publication in IUCr journals (*Acta Crystallographica*, *Journal of Applied Crystallography*, *Journal of Synchrotron Radiation*); however, if you intend to submit to *Acta Crystallographica Section C* or *E* or *IUCrData*, you should make sure that full publication checks are run on the final version of your CIF prior to submission.

Publication of your CIF in other journals

Please refer to the *Notes for Authors* of the relevant journal for any special instructions relating to CIF submission.

PLATON version of 28/11/2022; check.def file version of 28/11/2022

Datablock z - ellipsoid plot

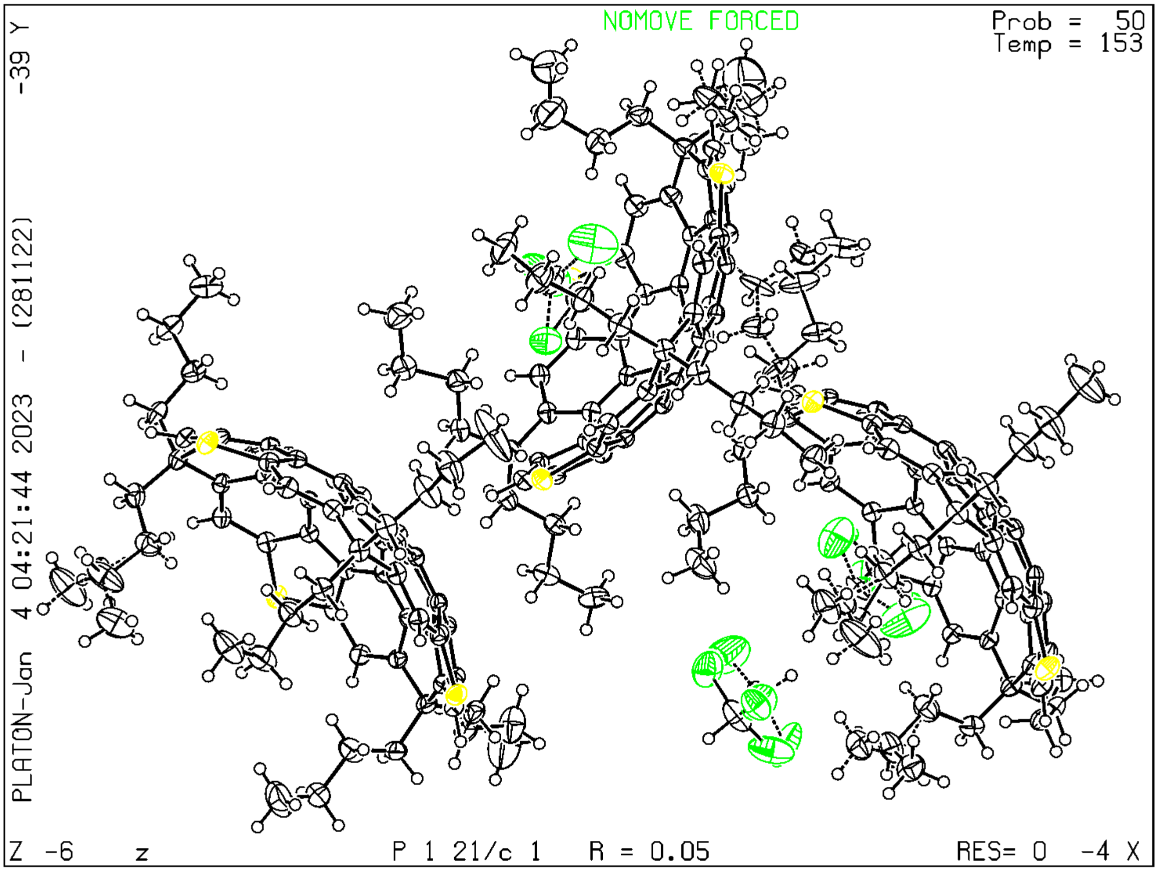

Supplement: Supplementary file 4 — Supplementary Data 1 [file 41467_2023_39086_MOESM4_ESM.zip › 1a/1a_cifreport.pdf]
