# Supplementary material for: Trichalcogenasupersumanenes and its concave-convex supramolecular assembly with fullerenes
Source: Nat Commun. 2023 Jun 10;14:3446. doi: 10.1038/s41467-023-39086-0 (PMC10257710; doi:10.1038/s41467-023-39086-0)
Supplement: Supplementary file 4 — Supplementary Data 1 [file 41467_2023_39086_MOESM4_ESM.zip › 1a/1a_tables.html]

z


# z

Table 1 Crystal data and structure refinement for z.

| Identification code | z |
| Empirical formula | C70H61Cl3S3 |
| Formula weight | 1104.71 |
| Temperature/K | 153.00 |
| Crystal system | monoclinic |
| Space group | P21/c |
| a/Å | 25.7504(12) |
| b/Å | 23.7050(11) |
| c/Å | 29.4867(13) |
| α/° | 90 |
| β/° | 112.839(2) |
| γ/° | 90 |
| Volume/Å3 | 16587.9(13) |
| Z | 12 |
| ρcalcg/cm3 | 1.327 |
| μ/mm‑1 | 2.891 |
| F(000) | 6960.0 |
| Crystal size/mm3 | 0.4 × 0.2 × 0.1 |
| Radiation | CuKα (λ = 1.54178) |
| 2Θ range for data collection/° | 4.946 to 136.698 |
| Index ranges | -31 ≤ h ≤ 30, -28 ≤ k ≤ 28, -35 ≤ l ≤ 35 |
| Reflections collected | 259572 |
| Independent reflections | 30313 [Rint = 0.0516, Rsigma = 0.0251] |
| Data/restraints/parameters | 30313/717/2291 |
| Goodness-of-fit on F2 | 1.055 |
| Final R indexes [I>=2σ (I)] | R1 = 0.0480, wR2 = 0.1365 |
| Final R indexes [all data] | R1 = 0.0546, wR2 = 0.1428 |
| Largest diff. peak/hole / e Å-3 | 1.66/-0.71 |

Table 2 Fractional Atomic Coordinates (×104) and Equivalent Isotropic Displacement Parameters (Å2×103) for z. Ueq is defined as 1/3 of the trace of the orthogonalised UIJ tensor.

| Atom | *x* | *y* | *z* | U(eq) |
| --- | --- | --- | --- | --- |
| S1 | 4896.9(2) | 657.7(2) | 5236.6(2) | 28.27(10) |
| S4 | 182.7(2) | 2125.5(2) | 5838.6(2) | 29.69(11) |
| S6 | 3358.4(2) | 2791.3(2) | 7183.2(2) | 30.81(11) |
| S8 | -3405.0(2) | 2778.5(2) | 3653.8(2) | 31.37(11) |
| S9 | -1770.6(2) | 788.5(2) | 1666.4(2) | 32.31(11) |
| S5 | -828.2(2) | -284.3(2) | 5048.6(2) | 32.74(11) |
| S3 | 2359.7(2) | 5491.6(2) | 7081.2(2) | 33.54(11) |
| S7 | 5707.8(2) | -416.2(2) | 8623.6(2) | 36.13(12) |
| S2 | 1630.8(2) | 4200.0(2) | 3761.4(2) | 37.71(12) |
| Cl1 | 4294(3) | 637(2) | 6373(2) | 49.6(9) |
| Cl4 | 2314.6(19) | -589(2) | 7217.9(16) | 59.5(7) |
| Cl6 | 505.8(4) | 3672.3(3) | 5467.7(3) | 69.23(19) |
| Cl8 | 3675(3) | 1191(3) | 6900(3) | 94.3(17) |
| Cl9 | 1184.0(9) | 4654.7(11) | 5853.3(9) | 57.5(6) |
| Cl5 | 940.6(11) | 4300.8(14) | 4856.2(10) | 63.0(6) |
| Cl3 | 4362(4) | 229(3) | 7321(2) | 124.2(18) |
| Cl7 | 2746(2) | -1171.8(10) | 6582.6(11) | 94.9(10) |
| Cl2 | 2102.0(11) | -203.5(13) | 6224.2(8) | 79.3(7) |
| C1 | 5668.7(7) | 1251.4(8) | 6987.3(6) | 20.7(3) |
| C4 | -1113.5(7) | 1565.1(7) | 4196.2(6) | 21.5(3) |
| C6 | 5786.3(7) | 1091.7(8) | 7482.6(6) | 21.6(3) |
| C8 | 5227.3(7) | 1901.8(7) | 7564.8(6) | 21.9(3) |
| C10 | 5750.7(7) | 838.8(8) | 6658.7(6) | 21.3(3) |
| C12 | 1883.9(7) | 2912.7(8) | 5667.5(6) | 22.3(3) |
| C14 | 5163.0(7) | 1386.8(8) | 5942.9(6) | 21.4(3) |
| C16 | 5109.9(7) | 2060.9(7) | 7069.4(6) | 20.9(3) |
| C18 | -1493.0(7) | 2019.3(7) | 3989.0(6) | 21.5(3) |
| C20 | 5487.9(7) | 901.4(8) | 6146.4(6) | 21.6(3) |
| C22 | -902.5(7) | 1035.1(8) | 3076.7(6) | 22.9(3) |
| C24 | 5566.5(7) | 1421.1(8) | 7772.4(6) | 22.1(3) |
| C26 | -1640.0(7) | 2172.9(7) | 3487.8(6) | 20.8(3) |
| C28 | -534.4(7) | 349.2(8) | 3739.6(7) | 24.8(4) |
| C30 | -631.9(7) | 716.5(8) | 4064.5(6) | 23.4(3) |
| C32 | -879.0(7) | 1267.3(7) | 3901.3(6) | 21.3(3) |
| C34 | 2316.8(7) | 3600.6(8) | 6336.7(6) | 21.7(3) |
| C36 | 4641.7(7) | 2437.4(7) | 6821.9(6) | 22.7(3) |
| C38 | -1018.1(7) | 1425.1(7) | 3403.2(6) | 21.0(3) |
| C40 | 2000.9(7) | 3099.1(7) | 6156.7(6) | 22.0(3) |
| C42 | 2090.7(7) | 3226.0(8) | 5359.9(6) | 22.3(3) |
| C44 | 2407.2(7) | 3728.9(8) | 5541.3(6) | 22.7(3) |
| C46 | 5335.0(7) | 1736.5(7) | 6781.8(6) | 20.4(3) |
| C48 | 2477.1(7) | 4120.9(8) | 5192.5(6) | 25.5(4) |
| C50 | 5982.4(7) | 525.9(8) | 7640.5(6) | 24.4(4) |
| C52 | 2687.1(7) | 4499.8(8) | 6156.3(6) | 24.3(4) |
| C54 | -1092.6(7) | 1309.5(8) | 4648.7(6) | 23.4(3) |
| C56 | 6042.0(7) | 137.1(8) | 7313.3(6) | 24.9(4) |
| C58 | -669.2(7) | 508.9(8) | 3245.3(7) | 24.7(4) |
| C60 | 4869.9(8) | 1876.3(8) | 8226.4(6) | 26.1(4) |
| C62 | -1182.5(7) | 1080.3(8) | 2564.2(6) | 23.9(3) |
| C64 | 2295.6(7) | 3873.2(8) | 6769.5(6) | 23.6(3) |
| C66 | -1662.9(7) | 1929.0(7) | 2665.2(6) | 22.2(3) |
| C68 | -1564.6(7) | 1526.4(8) | 2359.5(6) | 23.0(3) |
| C70 | 4417.0(8) | 2485.1(7) | 6313.8(7) | 23.8(3) |
| C72 | -652.7(7) | 464.0(8) | 4487.1(7) | 25.4(4) |
| C74 | 4788.1(8) | 1329.1(8) | 5453.1(6) | 24.3(4) |
| C76 | 2518.3(7) | 3918.4(8) | 6027.3(6) | 22.5(3) |
| C78 | 5924.5(7) | 294.1(8) | 6821.9(6) | 23.0(3) |
| C80 | 5095.1(7) | 1806.3(7) | 6251.3(6) | 20.7(3) |
| C82 | 5557.0(7) | 1177.0(8) | 8219.3(6) | 24.4(4) |
| C84 | -1849.2(7) | 2209.6(8) | 4237.8(6) | 23.6(3) |
| C86 | 5379.9(7) | 440.2(8) | 5827.6(6) | 25.4(4) |
| C88 | 1661.4(7) | 2876.9(8) | 6409.3(6) | 23.3(3) |
| C90 | 4871.7(7) | 2125.8(8) | 7802.3(6) | 23.6(3) |
| C92 | 4647.4(7) | 2172.0(7) | 6028.0(6) | 22.8(3) |
| C94 | 1649.8(7) | 3149.9(8) | 6820.6(6) | 24.5(4) |
| C96 | 4396.8(8) | 2454.8(8) | 7539.7(7) | 24.6(4) |
| C98 | -2142.6(7) | 2510.5(7) | 3243.4(6) | 22.8(3) |
| C100 | 1203.4(7) | 2516.4(8) | 6168.4(7) | 24.7(4) |
| C102 | 1968.6(7) | 3642.8(8) | 7003.9(6) | 24.9(4) |
| C104 | 2630.2(7) | 4758.2(8) | 6561.3(7) | 26.2(4) |
| C106 | -2388.5(7) | 2539.0(7) | 2735.5(7) | 23.9(3) |
| C108 | 5215.7(8) | 1409.4(8) | 8438.1(6) | 25.8(4) |
| C110 | 2218.6(8) | 4020.5(8) | 4683.7(7) | 27.3(4) |
| C112 | 5938.1(7) | 292.7(9) | 8062.7(6) | 26.8(4) |
| C114 | -2148.8(7) | 2247.5(8) | 2445.0(6) | 23.7(3) |
| C116 | -2347.9(8) | 2510.0(8) | 3982.3(7) | 24.8(4) |
| C118 | 2734.3(7) | 4866.8(8) | 5808.5(7) | 26.9(4) |
| C120 | 5544.0(8) | -112.6(8) | 6008.1(7) | 27.7(4) |
| C122 | -878.3(7) | 761.3(8) | 4780.7(6) | 25.5(4) |
| C124 | 3865.2(8) | 2657.9(8) | 6029.5(7) | 26.6(4) |
| C126 | -1439.0(7) | 1503.3(8) | 4876.9(6) | 25.9(4) |
| C128 | -2492.8(8) | 2659.4(7) | 3489.3(7) | 23.9(3) |
| C130 | 751.4(8) | 2463.2(8) | 6318.4(7) | 27.3(4) |
| C132 | -1245.8(8) | 618.9(9) | 2253.6(7) | 28.4(4) |
| C134 | 1430.2(7) | 2512.2(8) | 5440.1(6) | 23.5(3) |
| C136 | 1200.4(8) | 2442.3(8) | 4935.6(7) | 25.5(4) |
| C138 | 2632.9(8) | 4675.4(9) | 5328.6(7) | 27.8(4) |
| C140 | 1906.2(8) | 3524.5(8) | 4505.2(6) | 26.4(4) |
| C142 | 2434.6(7) | 4441.9(8) | 6869.0(6) | 25.3(4) |
| C144 | 656.5(8) | 2235.7(8) | 4662.0(7) | 27.7(4) |
| C146 | 4329.2(8) | 1708.9(8) | 5238.7(6) | 26.3(4) |
| C148 | 3936.8(8) | 2498.7(8) | 7679.8(7) | 28.5(4) |
| C150 | -1401.2(7) | 1879.0(7) | 3194.9(6) | 20.5(3) |
| C152 | -2959.4(8) | 2660.0(8) | 2461.3(7) | 26.5(4) |
| C154 | 1088.8(8) | 2332.7(8) | 5685.5(7) | 24.9(4) |
| C156 | 4283.7(8) | 2614.1(7) | 7052.2(7) | 24.3(4) |
| C158 | -2794.3(8) | 2520.7(8) | 4143.1(7) | 27.8(4) |
| C160 | -2748.9(8) | 2247.0(9) | 4584.9(7) | 30.2(4) |
| C162 | -1819.8(8) | 1951.4(8) | 4668.2(6) | 25.3(4) |
| C164 | 5728.2(7) | 619.6(9) | 8351.4(6) | 26.8(4) |
| C166 | 4422.1(8) | 1915.8(8) | 8387.4(7) | 28.2(4) |
| C168 | 1850.4(7) | 3125.5(8) | 4835.3(6) | 24.4(4) |
| C170 | 2591.8(8) | 5341.9(9) | 6597.9(7) | 30.1(4) |
| C172 | 1412.8(7) | 2744.3(8) | 4630.8(7) | 25.6(4) |
| C174 | -2254.1(8) | 1952.1(8) | 4845.8(7) | 28.1(4) |
| C176 | 1761.5(8) | 3947.4(9) | 7305.8(7) | 28.0(4) |
| C178 | 751.6(8) | 2753.0(8) | 6739.3(7) | 28.5(4) |
| C180 | -1602.3(8) | 1183.6(9) | 5199.2(7) | 29.3(4) |
| C182 | 3727.3(8) | 2786.6(8) | 6783.1(7) | 27.3(4) |
| C184 | -3307.4(8) | 2789.9(8) | 2711.1(7) | 28.4(4) |
| C186 | 1207.9(8) | 3104.8(8) | 6988.1(7) | 27.4(4) |
| C188 | 2104.8(9) | 4459.1(10) | 4342.5(7) | 34.2(4) |
| C190 | -2447.4(8) | 1761.2(9) | 1667.0(7) | 28.5(4) |
| C192 | -1947.9(8) | 1434.7(8) | 1876.5(7) | 26.9(4) |
| C194 | 4257.5(8) | 2130.7(8) | 5538.6(6) | 25.4(4) |
| C196 | 3948.9(8) | 2235.1(9) | 8116.1(7) | 30.5(4) |
| C198 | 541.9(8) | 2127.9(8) | 5430.4(7) | 27.5(4) |
| C200 | -1010.4(8) | 84.2(9) | 2439.2(7) | 31.1(4) |
| C202 | 3509.4(8) | 2816.7(8) | 6264.2(7) | 29.5(4) |
| C204 | 1897.9(8) | 4518.8(9) | 7395.2(7) | 30.1(4) |
| C206 | -3065.7(8) | 2786.5(8) | 3231.2(7) | 27.8(4) |
| C208 | -614.8(8) | -121.7(8) | 4558.0(7) | 29.7(4) |
| C13 | 317.2(8) | 2068.0(8) | 4912.5(7) | 29.7(4) |
| C105 | 2237.0(8) | 4765.9(8) | 7165.1(7) | 28.9(4) |
| C53 | -2549.4(7) | 2168.5(8) | 1963.6(7) | 25.8(4) |
| C107 | 5023.3(8) | 1116.4(9) | 8756.6(6) | 29.1(4) |
| C27 | 5819.6(8) | -183.8(8) | 6516.6(7) | 26.3(4) |
| C109 | 5192.9(8) | 558.4(9) | 8880.6(7) | 32.7(4) |
| C55 | -1030.4(8) | 424.0(9) | 5099.5(7) | 30.0(4) |
| C111 | 1529.0(8) | 3547.8(9) | 4012.8(7) | 30.2(4) |
| C7 | -2107.6(8) | 1496.1(9) | 5256.7(7) | 29.8(4) |
| C113 | 1021.4(8) | 2748.7(9) | 4141.9(7) | 29.2(4) |
| C57 | 1302.3(8) | 3574.1(9) | 7381.3(7) | 29.6(4) |
| C115 | -3095.1(8) | 2491.1(8) | 1917.4(7) | 28.6(4) |
| C29 | -1390.6(8) | 637.7(9) | 5321.5(7) | 31.8(4) |
| C117 | 1081.1(8) | 3153.5(9) | 3820.8(7) | 31.6(4) |
| C59 | 4541.9(8) | 1478.9(9) | 8808.4(7) | 30.6(4) |
| C119 | -523.1(8) | -485.6(8) | 4212.0(7) | 31.4(4) |
| C15 | 5929.5(8) | -291.4(9) | 8131.1(7) | 30.6(4) |
| C121 | -718.5(8) | 33.5(8) | 2946.3(7) | 27.8(4) |
| C61 | 5545.7(8) | 308.4(9) | 8666.1(7) | 31.3(4) |
| C123 | 2695.6(8) | 5455.9(9) | 5825.4(7) | 31.1(4) |
| C31 | 523.9(8) | 2353.9(9) | 4109.7(7) | 30.4(4) |
| C125 | -489.6(8) | -240.9(8) | 3793.7(7) | 29.3(4) |
| C63 | 3743.1(8) | 2512.1(8) | 5483.5(7) | 29.6(4) |
| C127 | 5972.7(8) | -678.7(9) | 7778.7(7) | 32.0(4) |
| C2 | 6025.5(8) | -450.5(8) | 7361.6(7) | 28.8(4) |
| C129 | -3196.5(8) | 3014.8(9) | 1580.9(7) | 33.0(4) |
| C65 | 760.4(9) | 3906.2(9) | 7303.4(8) | 34.7(4) |
| C131 | 1073.2(9) | 1456.7(9) | 4037.0(7) | 33.4(4) |
| C33 | 2519.0(9) | 5131.5(9) | 4999.6(7) | 33.3(4) |
| C133 | 5942.6(8) | -716.2(8) | 6852.2(7) | 29.7(4) |
| C67 | 526.8(9) | 1792.7(9) | 3838.3(7) | 34.6(4) |
| C135 | 2253.2(9) | 5024.9(10) | 4495.8(7) | 37.5(5) |
| C17 | -2604.8(9) | 1093.1(9) | 5174.1(7) | 33.7(4) |
| C137 | 1015.8(10) | 880.8(9) | 3793.5(8) | 37.8(5) |
| C69 | 3180.7(8) | 2198.6(10) | 5234.4(7) | 35.8(5) |
| C139 | 2629.8(9) | 5708.1(9) | 6229.0(8) | 33.8(4) |
| C35 | -53.8(8) | 2639.6(10) | 3851.1(8) | 35.2(4) |
| C141 | 1524.9(9) | 3312.5(10) | 7905.3(7) | 33.8(4) |
| C71 | 3097.2(9) | 1671.1(10) | 5489.2(9) | 42.1(5) |
| C143 | 6519.0(9) | -1165.5(9) | 6386.9(8) | 36.1(4) |
| C9 | -3622.0(8) | 2112.4(10) | 1711.2(8) | 37.6(5) |
| C145 | 6876.6(9) | -747.0(10) | 6243.4(8) | 39.9(5) |
| C73 | -2736.2(10) | 3459.4(9) | 1740.6(8) | 37.5(5) |
| C147 | -131.2(10) | 3212.1(10) | 4042.7(10) | 43.7(5) |
| C37 | 2622.5(9) | 5685.9(9) | 5308.5(7) | 35.8(5) |
| C149 | 4024.7(9) | 1121.0(10) | 8753.9(8) | 36.6(5) |
| C75 | 2035.6(9) | 2924.4(10) | 8042.1(7) | 34.8(4) |
| C151 | 5443.8(10) | -1133.6(9) | 6683.9(8) | 37.0(4) |
| C19 | 3714(2) | 3059.2(15) | 5186.2(17) | 38.5(10) |
| C153 | 6491.1(9) | -1017.9(10) | 6886.0(8) | 36.3(4) |
| C77 | -1937.4(9) | 1769.4(10) | 5770.8(7) | 35.3(4) |
| C155 | -510.3(8) | -486.3(8) | 3294.9(7) | 32.0(4) |
| C39 | 4749.3(9) | 1788.4(10) | 9313.0(7) | 37.1(5) |
| C157 | -3596.0(10) | 1564.8(11) | 1980.1(10) | 47.5(5) |
| C79 | -2851.3(10) | 803.3(11) | 4673.5(8) | 41.5(5) |
| C159 | 103.7(9) | -634.1(10) | 3357.2(8) | 38.3(5) |
| C5 | -2873.3(10) | 3958.8(10) | 1389.2(9) | 42.4(5) |
| C161 | 2120.3(11) | 6093.8(10) | 5101.8(9) | 44.3(5) |
| C81 | 479.8(9) | 4204.5(10) | 6811.3(8) | 40.1(5) |
| C163 | 1572.7(12) | 573.3(10) | 3942.7(10) | 47.1(6) |
| C41 | -895.4(10) | -997.3(9) | 3109.2(8) | 40.1(5) |
| C165 | 2217.5(10) | 2691.1(11) | 8561.6(8) | 44.6(5) |
| C83 | 169.3(10) | -850.2(12) | 2893.4(9) | 48.4(6) |
| C167 | 4229.5(13) | 3436.0(10) | 5377.8(9) | 42.0(7) |
| C21 | 3749.5(10) | 809.2(11) | 8267.5(9) | 45.6(5) |
| C169 | 2686.5(12) | 2260.1(12) | 8693.8(10) | 52.0(6) |
| C85 | 4874.4(10) | -882.9(12) | 6612.5(10) | 50.3(6) |
| C43 | 2508.9(10) | 1435.8(13) | 5249.8(11) | 54.8(7) |
| C173 | -740.8(11) | 3412.8(11) | 3797.6(10) | 51.1(6) |
| C87 | 6837.0(11) | -826.0(13) | 5719.0(9) | 53.0(6) |
| C175 | 5238.7(11) | 2195.8(12) | 9421.3(9) | 49.9(6) |
| C11 | 1555.9(11) | 5848.9(12) | 5063.5(10) | 52.9(6) |
| C177 | -2406.3(13) | 4393.1(11) | 1534.0(10) | 52.9(6) |
| C89 | -1486.4(11) | -938.8(11) | 3098.7(10) | 48.9(6) |
| C179 | -1418.6(11) | 2135.8(14) | 5944.3(10) | 55.9(7) |
| C45 | -3266.7(13) | 336.2(13) | 4651.0(11) | 59.1(7) |
| C181 | -1864.1(12) | -1431.0(12) | 2828.8(11) | 58.3(7) |
| C91 | -1305.9(12) | 2422.0(15) | 6428.4(11) | 63.9(8) |
| C183 | 5403.3(13) | 2476.5(15) | 9924.2(11) | 63.5(8) |
| C23 | 778.8(12) | -858.4(17) | 2939.7(11) | 68.0(9) |
| C93 | -62.0(12) | 4498.9(14) | 6762.2(12) | 61.4(7) |
| C189 | 4132(2) | 4006.2(14) | 5127.0(14) | 60.6(11) |
| C95 | 2392.2(14) | 955.2(16) | 5531.5(15) | 75.2(9) |
| C191 | -818.8(13) | 4014.1(13) | 3925.2(14) | 66.1(8) |
| C3 | -328.7(14) | 4841.0(16) | 6303.7(14) | 75.4(9) |
| C193 | 5888.4(17) | 2887.0(19) | 10044.5(14) | 88.0(13) |
| C97 | -3755.4(14) | 511.9(17) | 4771.0(14) | 76.9(10) |
| C195 | -2417.2(15) | -1427.3(19) | 2887.4(16) | 91.7(13) |
| C49 | 4660(3) | 4363.1(14) | 5264.1(19) | 88.3(17) |
| C197 | 845.3(17) | -1111(2) | 2489.6(15) | 104.2(16) |
| C99 | -773.0(17) | 2749(3) | 6641.7(19) | 132(2) |
| C199 | 1045.2(17) | 6205.7(19) | 4788(2) | 98.7(14) |
| C101 | 2301(3) | -738(3) | 6610.2(18) | 66.1(14) |
| C203 | 1013(2) | 6747(2) | 4938(2) | 108.5(15) |
| C51 | 1003(2) | 4138(2) | 5429.0(18) | 56.8(15) |
| C207 | 3970(2) | 587(2) | 6805(2) | 42.6(15) |
| C171 | 3180.4(11) | 5971.2(12) | 5358.1(9) | 48.0(6) |
| C185 | 3191.5(13) | 6171.7(13) | 4842.0(10) | 61.6(7) |
| C205 | 3688(3) | 5669(3) | 4764(2) | 79(2) |
| C210 | 3655(3) | 5817(4) | 4271(2) | 86(2) |
| C211 | 3768(3) | 6115(4) | 4801(3) | 74(2) |
| C1A | 3864(6) | 6320(8) | 4396(5) | 136(5) |
| C212 | 4247(3) | 824(3) | 6922(2) | 66(2) |
| Cl3A | 4221(3) | 655(3) | 6354(2) | 66.2(15) |
| Cl1A | 4475.6(10) | 320.5(17) | 7357.6(10) | 59.9(8) |
| Cl8A | 3741(2) | 1258.2(16) | 6929(2) | 65.0(10) |
| C51A | 698(4) | 4341(3) | 5336(3) | 65(2) |
| Cl9A | 783(5) | 4416(4) | 4810(2) | 131(3) |
| Cl5A | 1085(4) | 4746(4) | 5811(3) | 132(3) |
| C213 | 2581(4) | -529(4) | 6747(3) | 61(2) |
| Cl4A | 2929(2) | -1126(3) | 6623.7(18) | 92.5(17) |
| Cl7A | 2372(4) | -572(4) | 7177(3) | 82(2) |
| Cl2A | 2339(4) | -60(3) | 6311(3) | 124(2) |
| C25 | 3351(3) | 337(3) | 8262(2) | 59.2(15) |
| C103 | 2868(2) | 534(3) | 8382(3) | 76.6(19) |
| C25A | 3150(5) | 575(5) | 8234(4) | 70(3) |
| C214 | 3199(6) | 151(6) | 8631(5) | 109(6) |
| C19A | 3858(17) | 3007(10) | 5203(16) | 60(10) |
| C215 | 3524(11) | 3550(8) | 5143(9) | 54(6) |
| C1B | 3676(11) | 3999(8) | 4854(7) | 47(6) |
| C49A | 4227(9) | 4322(11) | 5062(8) | 38(5) |
| C187 | -4142(3) | 1230(3) | 1830(4) | 74(3) |
| C209 | -4121(3) | 712(3) | 2015(3) | 109(2) |
| C216 | -4167(6) | 1281(9) | 1734(6) | 49(5) |
| C1C | -4412(6) | 1117(8) | 1245(5) | 89(5) |
| C47 | 4384(4) | -1289(4) | 6548(4) | 55(2) |
| C201 | 4433.4(18) | -1507.2(19) | 7042.4(17) | 57.9(13) |
| C217 | 3950(4) | -1226(4) | 6570(4) | 67(3) |
| C47A | 4500(7) | -1409(7) | 6553(11) | 64(5) |

Table 3 Anisotropic Displacement Parameters (Å2×103) for z. The Anisotropic displacement factor exponent takes the form: -2π2[h2a\*2U11+2hka\*b\*U12+…].

| Atom | U11 | U22 | U33 | U23 | U13 | U12 |
| --- | --- | --- | --- | --- | --- | --- |
| S1 | 35.6(2) | 31.3(2) | 16.23(19) | -2.82(16) | 8.25(17) | 6.05(18) |
| S4 | 27.8(2) | 32.7(2) | 31.9(2) | 0.13(18) | 15.22(18) | -5.19(18) |
| S6 | 31.2(2) | 33.7(2) | 32.5(2) | -2.00(19) | 17.76(19) | 5.87(18) |
| S8 | 30.2(2) | 36.7(3) | 32.3(2) | 4.02(19) | 17.79(19) | 9.70(19) |
| S9 | 36.0(2) | 37.6(3) | 20.7(2) | -6.65(18) | 8.03(18) | 5.32(19) |
| S5 | 34.0(2) | 31.9(2) | 31.7(2) | 10.33(19) | 12.01(19) | 2.86(19) |
| S3 | 41.3(3) | 30.9(2) | 31.8(2) | -6.21(19) | 18.0(2) | -4.7(2) |
| S7 | 44.7(3) | 40.8(3) | 27.8(2) | 13.2(2) | 19.4(2) | 9.5(2) |
| S2 | 45.2(3) | 45.3(3) | 19.2(2) | 5.71(19) | 8.79(19) | -5.9(2) |
| Cl1 | 39.8(12) | 52.2(16) | 66(2) | -11.6(12) | 30.8(11) | -14.1(12) |
| Cl4 | 61.8(10) | 74.2(13) | 49.1(12) | -3.1(9) | 28.6(9) | -25.0(9) |
| Cl6 | 76.3(5) | 57.8(4) | 75.5(5) | 8.8(4) | 31.5(4) | -4.6(3) |
| Cl8 | 98(3) | 117(4) | 79(2) | -3(3) | 45(2) | 44(3) |
| Cl9 | 51.9(9) | 56.7(9) | 51.6(9) | -13.3(6) | 6.7(7) | -8.1(6) |
| Cl5 | 68.2(10) | 74.3(12) | 53.2(11) | 1.6(9) | 30.7(9) | -3.6(8) |
| Cl3 | 197(5) | 106(3) | 108(3) | 14(2) | 101(3) | 51(3) |
| Cl7 | 160(3) | 58.8(10) | 86.0(13) | -22.8(10) | 70.2(16) | -29.5(13) |
| Cl2 | 79.1(13) | 99.0(16) | 54.4(8) | 22.8(8) | 19.9(8) | 7.3(10) |
| C1 | 17.7(7) | 27.1(9) | 18.2(8) | -2.8(7) | 8.0(6) | -4.3(6) |
| C4 | 20.7(8) | 22.8(8) | 20.5(8) | -3.3(7) | 7.4(6) | -5.1(6) |
| C6 | 16.8(7) | 30.5(9) | 17.1(8) | -2.4(7) | 6.3(6) | -4.6(6) |
| C8 | 22.0(8) | 24.8(9) | 20.2(8) | -6.0(7) | 9.5(6) | -7.4(7) |
| C10 | 17.8(7) | 28.3(9) | 19.3(8) | -1.4(7) | 8.9(6) | -1.5(6) |
| C12 | 20.6(8) | 24.6(9) | 22.6(8) | 2.3(7) | 9.5(7) | 4.1(7) |
| C14 | 22.9(8) | 25.9(9) | 18.8(8) | 0.7(7) | 11.7(6) | -1.7(7) |
| C16 | 21.9(8) | 22.7(8) | 19.9(8) | -4.6(6) | 10.3(6) | -7.0(6) |
| C18 | 22.0(8) | 21.4(8) | 22.6(8) | -3.2(7) | 10.2(7) | -4.5(6) |
| C20 | 22.5(8) | 26.6(9) | 18.4(8) | 0.0(7) | 11.0(6) | -0.4(7) |
| C22 | 20.3(8) | 26.1(9) | 23.2(8) | -2.4(7) | 9.5(7) | -2.0(7) |
| C24 | 19.3(8) | 30.8(9) | 16.2(8) | -4.4(7) | 6.7(6) | -6.1(7) |
| C26 | 22.5(8) | 19.3(8) | 21.7(8) | -2.1(6) | 9.9(7) | -3.6(6) |
| C28 | 21.0(8) | 26.3(9) | 24.8(9) | -0.4(7) | 6.4(7) | 1.4(7) |
| C30 | 19.7(8) | 25.8(9) | 22.4(8) | 0.2(7) | 5.7(6) | -0.5(7) |
| C32 | 18.1(7) | 23.7(8) | 21.9(8) | -2.4(7) | 7.4(6) | -3.6(6) |
| C34 | 18.7(7) | 27.9(9) | 18.8(8) | 3.2(7) | 7.6(6) | 3.6(7) |
| C36 | 27.2(8) | 19.2(8) | 23.5(8) | -3.2(7) | 12.0(7) | -4.2(7) |
| C38 | 18.0(7) | 22.9(8) | 21.6(8) | -2.8(7) | 7.3(6) | -3.5(6) |
| C40 | 20.6(8) | 24.6(9) | 21.8(8) | 3.9(7) | 9.4(6) | 4.3(6) |
| C42 | 20.2(8) | 26.6(9) | 21.6(8) | 2.3(7) | 9.6(6) | 4.4(7) |
| C44 | 18.6(7) | 29.4(9) | 20.7(8) | 3.5(7) | 8.2(6) | 2.8(7) |
| C46 | 19.4(7) | 24.1(8) | 19.9(8) | -2.2(6) | 9.8(6) | -5.4(6) |
| C48 | 22.8(8) | 33.5(10) | 22.0(8) | 3.1(7) | 10.6(7) | 0.1(7) |
| C50 | 19.4(8) | 35.9(10) | 17.3(8) | 1.1(7) | 6.6(6) | 0.5(7) |
| C52 | 20.7(8) | 30.2(9) | 21.9(8) | 2.4(7) | 8.1(7) | -1.2(7) |
| C54 | 22.0(8) | 27.0(9) | 19.5(8) | -1.3(7) | 6.0(6) | -3.0(7) |
| C56 | 21.3(8) | 32.3(10) | 20.6(8) | 2.6(7) | 7.6(6) | 4.2(7) |
| C58 | 21.7(8) | 27.2(9) | 24.4(9) | -2.9(7) | 8.3(7) | 0.6(7) |
| C60 | 28.4(9) | 31.7(10) | 20.4(8) | -7.5(7) | 11.9(7) | -5.9(7) |
| C62 | 22.6(8) | 28.8(9) | 21.5(8) | -1.5(7) | 10.0(7) | -0.7(7) |
| C64 | 21.0(8) | 30.2(9) | 19.0(8) | 1.7(7) | 7.1(6) | 1.9(7) |
| C66 | 22.7(8) | 23.4(8) | 22.4(8) | 0.8(7) | 11.0(7) | -2.4(7) |
| C68 | 24.8(8) | 26.5(9) | 20.9(8) | 0.7(7) | 12.2(7) | -1.0(7) |
| C70 | 29.7(9) | 20.0(8) | 24.9(9) | 0.0(7) | 14.1(7) | -1.8(7) |
| C72 | 22.4(8) | 27.9(9) | 23.0(8) | 2.4(7) | 5.5(7) | 1.1(7) |
| C74 | 30.1(9) | 28.6(9) | 16.9(8) | -0.2(7) | 12.3(7) | 0.5(7) |
| C76 | 18.8(7) | 28.8(9) | 19.9(8) | 3.0(7) | 7.4(6) | 1.3(7) |
| C78 | 21.1(8) | 30.5(9) | 18.8(8) | 0.2(7) | 9.3(6) | 2.2(7) |
| C80 | 22.3(8) | 22.8(8) | 20.1(8) | -1.2(6) | 11.7(6) | -4.3(6) |
| C82 | 21.8(8) | 34.5(10) | 16.6(8) | -3.2(7) | 6.9(6) | -4.7(7) |
| C84 | 26.3(8) | 23.4(9) | 23.4(8) | -3.1(7) | 12.1(7) | -3.0(7) |
| C86 | 27.0(8) | 32.1(10) | 18.5(8) | -1.8(7) | 10.3(7) | 2.9(7) |
| C88 | 23.7(8) | 25.4(9) | 22.4(8) | 5.3(7) | 10.6(7) | 3.6(7) |
| C90 | 25.5(8) | 26.7(9) | 20.8(8) | -6.9(7) | 11.4(7) | -6.0(7) |
| C92 | 27.0(8) | 22.0(8) | 21.9(8) | 1.2(7) | 12.4(7) | -2.3(7) |
| C94 | 25.5(8) | 28.1(9) | 21.4(8) | 5.0(7) | 10.8(7) | 3.0(7) |
| C96 | 29.5(9) | 23.8(9) | 23.7(8) | -7.2(7) | 14.0(7) | -3.9(7) |
| C98 | 26.6(8) | 19.2(8) | 24.8(9) | 0.2(7) | 12.4(7) | -1.2(7) |
| C100 | 27.1(9) | 23.9(9) | 25.1(9) | 3.8(7) | 12.4(7) | 2.0(7) |
| C102 | 26.2(8) | 30.2(9) | 19.2(8) | 2.1(7) | 9.8(7) | 1.4(7) |
| C104 | 23.3(8) | 31.5(10) | 21.7(8) | -0.7(7) | 6.5(7) | -3.5(7) |
| C106 | 26.5(8) | 21.3(8) | 25.4(9) | 2.7(7) | 11.7(7) | 1.2(7) |
| C108 | 27.3(8) | 34.7(10) | 16.5(8) | -4.3(7) | 9.6(7) | -5.0(7) |
| C110 | 27.5(9) | 34.5(10) | 21.9(8) | 2.2(7) | 11.8(7) | -1.6(7) |
| C112 | 24.0(8) | 37.1(10) | 18.1(8) | 3.4(7) | 6.7(7) | 2.7(7) |
| C114 | 26.0(8) | 24.2(9) | 23.0(8) | 2.9(7) | 11.8(7) | -0.7(7) |
| C116 | 28.5(9) | 23.2(9) | 26.4(9) | -2.5(7) | 14.7(7) | -0.5(7) |
| C118 | 24.0(8) | 33.2(10) | 23.1(9) | 1.5(7) | 8.5(7) | -4.4(7) |
| C120 | 33.6(9) | 28.6(9) | 22.0(8) | -4.2(7) | 12.2(7) | 4.2(7) |
| C122 | 22.6(8) | 30.3(9) | 21.3(8) | 2.0(7) | 6.0(7) | -1.3(7) |
| C124 | 33.7(9) | 22.3(9) | 24.6(9) | 1.3(7) | 12.2(7) | 3.1(7) |
| C126 | 26.5(8) | 31.2(10) | 20.1(8) | -1.9(7) | 9.0(7) | -3.7(7) |
| C128 | 27.4(9) | 20.1(8) | 26.4(9) | -1.3(7) | 12.9(7) | 0.7(7) |
| C130 | 27.2(9) | 27.5(9) | 29.9(9) | 5.4(7) | 14.2(7) | -0.6(7) |
| C132 | 29.2(9) | 34.3(10) | 22.5(9) | -4.1(7) | 10.9(7) | 2.8(8) |
| C134 | 23.8(8) | 23.3(8) | 24.5(8) | -0.3(7) | 10.8(7) | 3.4(7) |
| C136 | 25.6(8) | 25.8(9) | 26.8(9) | -1.3(7) | 11.9(7) | 2.3(7) |
| C138 | 26.3(9) | 33.8(10) | 23.7(9) | 3.0(7) | 10.2(7) | -3.6(7) |
| C140 | 26.5(8) | 35.6(10) | 19.5(8) | 1.0(7) | 11.5(7) | 0.8(7) |
| C142 | 22.5(8) | 33.3(10) | 19.9(8) | -0.2(7) | 7.9(7) | -1.1(7) |
| C144 | 27.4(9) | 28.8(9) | 26.9(9) | -4.6(7) | 10.5(7) | -1.1(7) |
| C146 | 32.1(9) | 29.6(9) | 17.4(8) | 2.5(7) | 9.9(7) | 3.0(7) |
| C148 | 30.8(9) | 28.5(9) | 30.6(9) | -6.1(8) | 16.9(8) | -0.3(7) |
| C150 | 21.0(8) | 20.0(8) | 22.1(8) | -1.6(6) | 10.3(6) | -3.8(6) |
| C152 | 28.7(9) | 24.4(9) | 27.0(9) | 5.0(7) | 11.4(7) | 3.9(7) |
| C154 | 26.4(8) | 23.7(9) | 25.5(9) | 1.8(7) | 11.0(7) | 1.0(7) |
| C156 | 29.4(9) | 20.2(8) | 26.0(9) | -4.7(7) | 13.7(7) | -1.9(7) |
| C158 | 29.8(9) | 27.0(9) | 31.1(9) | -2.3(7) | 16.7(8) | 2.3(7) |
| C160 | 31.8(9) | 34.8(10) | 30.7(9) | -2.6(8) | 19.5(8) | 0.9(8) |
| C162 | 28.3(9) | 27.9(9) | 21.7(8) | -4.9(7) | 11.9(7) | -2.5(7) |
| C164 | 23.9(8) | 38.4(10) | 16.9(8) | 2.1(7) | 6.6(7) | 0.4(7) |
| C166 | 32.9(9) | 33.4(10) | 23.4(9) | -6.4(7) | 16.6(7) | -4.5(8) |
| C168 | 23.1(8) | 29.5(9) | 22.4(8) | -0.6(7) | 10.7(7) | 2.5(7) |
| C170 | 31.1(9) | 33.3(10) | 27.3(9) | -3.4(8) | 12.9(8) | -3.6(8) |
| C172 | 25.4(8) | 30.0(9) | 23.5(9) | -1.2(7) | 11.9(7) | 2.2(7) |
| C174 | 32.1(9) | 32.8(10) | 23.5(9) | -3.1(7) | 15.2(7) | -2.9(8) |
| C176 | 29.4(9) | 36.0(10) | 21.1(8) | -0.2(7) | 12.6(7) | 1.3(8) |
| C178 | 28.0(9) | 33.3(10) | 29.3(9) | 4.2(8) | 16.7(8) | 0.2(7) |
| C180 | 30.1(9) | 38.7(11) | 20.3(8) | 0.8(7) | 11.2(7) | -2.6(8) |
| C182 | 31.6(9) | 23.3(9) | 31.1(9) | -3.1(7) | 16.6(8) | 2.9(7) |
| C184 | 28.5(9) | 28.7(9) | 29.3(9) | 5.5(7) | 12.7(8) | 8.3(7) |
| C186 | 30.7(9) | 32.5(10) | 22.8(8) | 4.5(7) | 14.6(7) | 3.0(8) |
| C188 | 37.3(10) | 43.8(12) | 21.2(9) | 5.0(8) | 11.3(8) | -4.4(9) |
| C190 | 28.6(9) | 36.1(10) | 20.3(8) | 0.1(7) | 9.0(7) | 0.7(8) |
| C192 | 29.0(9) | 31.9(10) | 21.8(8) | -1.8(7) | 12.1(7) | -0.5(7) |
| C194 | 30.9(9) | 25.3(9) | 21.7(8) | 2.9(7) | 12.1(7) | 1.5(7) |
| C196 | 34.5(10) | 35.0(10) | 29.6(9) | -5.4(8) | 20.9(8) | -0.8(8) |
| C198 | 27.8(9) | 25.4(9) | 32.2(10) | 0.6(7) | 14.8(8) | -2.0(7) |
| C200 | 34.1(10) | 30.6(10) | 28.1(9) | -7.5(8) | 11.5(8) | 3.8(8) |
| C202 | 32.7(9) | 26.7(9) | 29.6(9) | 0.9(7) | 12.4(8) | 8.1(7) |
| C204 | 32.7(9) | 36.2(10) | 24.2(9) | -4.2(8) | 14.2(8) | 0.1(8) |
| C206 | 30.2(9) | 24.9(9) | 33.0(10) | 2.3(7) | 17.3(8) | 5.5(7) |
| C208 | 26.3(9) | 29.7(10) | 29.0(9) | 5.8(8) | 6.3(7) | 1.5(7) |
| C13 | 27.7(9) | 29.7(10) | 32.2(10) | -4.2(8) | 12.0(8) | -5.5(7) |
| C105 | 29.3(9) | 32.6(10) | 24.6(9) | -3.2(7) | 10.1(7) | -0.7(8) |
| C53 | 25.7(9) | 29.5(9) | 22.5(8) | 4.9(7) | 9.6(7) | 1.9(7) |
| C107 | 30.7(9) | 41.3(11) | 17.8(8) | -2.3(7) | 12.2(7) | -4.2(8) |
| C27 | 27.7(9) | 28.2(9) | 24.5(9) | 0.2(7) | 11.8(7) | 5.1(7) |
| C109 | 35.2(10) | 45.3(12) | 20.3(9) | 4.6(8) | 13.6(8) | -1.8(9) |
| C55 | 28.2(9) | 34.3(10) | 24.8(9) | 5.8(8) | 7.1(7) | 0.2(8) |
| C111 | 32.9(9) | 38.3(11) | 20.7(8) | 2.0(8) | 11.7(7) | 0.9(8) |
| C7 | 31.1(9) | 38.5(11) | 23.4(9) | 0.7(8) | 14.3(7) | -0.9(8) |
| C113 | 27.8(9) | 35.4(10) | 24.8(9) | -4.6(8) | 10.7(7) | 0.0(8) |
| C57 | 32.6(9) | 36.2(10) | 25.7(9) | -0.4(8) | 17.3(8) | -0.2(8) |
| C115 | 28.2(9) | 32.3(10) | 25.0(9) | 2.9(7) | 10.1(7) | 4.3(8) |
| C29 | 32.6(10) | 39.6(11) | 23.3(9) | 7.7(8) | 11.3(8) | -1.0(8) |
| C117 | 32.3(9) | 40.9(11) | 20.1(8) | -1.0(8) | 8.7(7) | -0.2(8) |
| C59 | 35.0(10) | 39.9(11) | 22.7(9) | -3.3(8) | 17.6(8) | -5.0(8) |
| C119 | 31.0(9) | 25.1(9) | 33.2(10) | 3.0(8) | 7.0(8) | 3.0(7) |
| C15 | 30.5(9) | 39.1(11) | 22.8(9) | 9.6(8) | 10.8(7) | 7.3(8) |
| C121 | 26.7(9) | 26.9(9) | 29.0(9) | -3.5(7) | 10.0(7) | 3.5(7) |
| C61 | 32.9(9) | 40.4(11) | 20.6(8) | 5.8(8) | 10.4(7) | 2.0(8) |
| C123 | 32.1(9) | 32.2(10) | 27.9(9) | 3.7(8) | 10.5(8) | -6.4(8) |
| C31 | 28.8(9) | 35.7(10) | 26.1(9) | -4.1(8) | 9.9(8) | -3.0(8) |
| C125 | 24.9(9) | 26.8(9) | 32.1(10) | -1.0(8) | 6.6(7) | 3.2(7) |
| C63 | 35.5(10) | 29.3(10) | 23.5(9) | 2.0(7) | 10.9(8) | 8.0(8) |
| C127 | 36.3(10) | 32.2(10) | 28.4(9) | 8.4(8) | 13.4(8) | 8.7(8) |
| C2 | 29.0(9) | 32.2(10) | 25.3(9) | 4.6(8) | 10.8(7) | 7.3(7) |
| C129 | 34.2(10) | 39.2(11) | 25.5(9) | 8.0(8) | 11.2(8) | 10.7(8) |
| C65 | 36.2(10) | 40.9(11) | 35.2(10) | -2.4(9) | 22.8(9) | 1.6(9) |
| C131 | 35.8(10) | 35.0(11) | 31.9(10) | -6.7(8) | 16.0(8) | -6.6(8) |
| C33 | 38.1(10) | 34.7(11) | 27.7(10) | 4.9(8) | 13.4(8) | -6.3(8) |
| C133 | 35.4(10) | 29.2(10) | 25.7(9) | 3.4(7) | 13.1(8) | 7.5(8) |
| C67 | 34.6(10) | 41.0(12) | 28.2(9) | -9.1(8) | 12.2(8) | -6.4(9) |
| C135 | 45.8(11) | 39.2(12) | 26.8(10) | 9.7(8) | 13.3(9) | -5.9(9) |
| C17 | 35.7(10) | 41.3(11) | 28.2(10) | 3.2(8) | 16.8(8) | -4.9(9) |
| C137 | 50.3(12) | 36.0(11) | 35.8(11) | -7.5(9) | 26.1(10) | -9.3(9) |
| C69 | 29.8(10) | 43.9(12) | 28.6(10) | -4.6(8) | 6.0(8) | 10.9(9) |
| C139 | 37.7(10) | 29.0(10) | 33.6(10) | -2.6(8) | 12.6(8) | -8.0(8) |
| C35 | 28.9(9) | 43.0(12) | 30.3(10) | -1.9(9) | 7.6(8) | -1.4(8) |
| C141 | 37.6(10) | 43.5(12) | 26.6(9) | 2.0(8) | 19.3(8) | -1.5(9) |
| C71 | 32.7(10) | 45.2(13) | 45.3(12) | -7.2(10) | 11.7(9) | -2.8(9) |
| C143 | 39.1(11) | 33.6(11) | 37.8(11) | -1.4(8) | 17.5(9) | 9.1(9) |
| C9 | 28.1(9) | 46.7(12) | 37.0(11) | -4.1(9) | 11.8(8) | 2.2(9) |
| C145 | 38.0(11) | 45.6(12) | 36.6(11) | -4.1(9) | 15.1(9) | 1.4(9) |
| C73 | 49.4(12) | 35.3(11) | 28.7(10) | 4.4(8) | 16.3(9) | 3.8(9) |
| C147 | 37.6(11) | 38.3(12) | 55.7(14) | -3.2(10) | 18.7(10) | -1.4(9) |
| C37 | 44.3(11) | 34.0(11) | 28.6(10) | 3.5(8) | 13.6(9) | -10.1(9) |
| C149 | 35.8(10) | 47.9(12) | 32.3(10) | 0.1(9) | 20.0(9) | -6.6(9) |
| C75 | 39.1(10) | 41.6(11) | 28.2(10) | 2.5(8) | 17.9(8) | 0.6(9) |
| C151 | 46.9(12) | 30.7(10) | 35.7(11) | -0.9(8) | 18.4(9) | -0.8(9) |
| C19 | 59(3) | 32.5(14) | 27.8(17) | 12.0(11) | 20(2) | 21.7(13) |
| C153 | 39.7(11) | 39.3(11) | 31.3(10) | 5.8(8) | 15.5(8) | 14.0(9) |
| C77 | 34.9(10) | 49.9(13) | 26.2(9) | -3.6(9) | 17.3(8) | -3.5(9) |
| C155 | 34.9(10) | 26.1(10) | 31.0(10) | -1.7(8) | 8.4(8) | 5.1(8) |
| C39 | 41.1(11) | 52.6(13) | 24.8(9) | -8.1(9) | 20.7(8) | -6.8(10) |
| C157 | 45.8(12) | 45.4(13) | 55.8(14) | -4.6(11) | 24.7(11) | -7.1(10) |
| C79 | 44.9(12) | 48.2(13) | 32.0(11) | -4.2(9) | 15.5(9) | -9.3(10) |
| C159 | 37.1(11) | 37.2(11) | 37.1(11) | -4.5(9) | 10.5(9) | 10.5(9) |
| C5 | 48.9(12) | 44.2(13) | 45.3(12) | 14.2(10) | 30.7(10) | 12.8(10) |
| C161 | 57.2(14) | 32.7(11) | 39.7(12) | 8.1(9) | 15.1(10) | -2.4(10) |
| C81 | 36.1(11) | 45.9(13) | 41.4(12) | 2.3(10) | 18.4(9) | 7.5(9) |
| C163 | 67.7(16) | 34.3(12) | 50.9(13) | 4.2(10) | 35.8(12) | 3.3(11) |
| C41 | 46.7(12) | 25.9(10) | 39.0(11) | -1.9(9) | 7.1(9) | -0.8(9) |
| C165 | 46.4(12) | 56.6(15) | 35.2(11) | 12.6(10) | 20.7(10) | 3.3(11) |
| C83 | 46.1(13) | 54.6(15) | 42.1(12) | -8.1(11) | 14.6(10) | 16.2(11) |
| C167 | 75.6(18) | 26.3(12) | 37.6(13) | 2.5(10) | 36.9(13) | 3.0(11) |
| C21 | 42.5(12) | 54.3(14) | 39.3(12) | -7.5(10) | 14.9(10) | -11.7(11) |
| C169 | 55.9(14) | 56.6(15) | 42.4(13) | 14.7(11) | 18.0(11) | 7.1(12) |
| C85 | 43.8(12) | 56.1(15) | 56.5(15) | -9.0(12) | 25.7(11) | -8.2(11) |
| C43 | 32.1(11) | 71.2(18) | 59.4(15) | -17.1(13) | 15.7(11) | -5.1(11) |
| C173 | 47.4(13) | 49.1(14) | 55.6(15) | -1.4(12) | 18.6(11) | -0.6(11) |
| C87 | 51.2(14) | 70.1(18) | 41.1(13) | -3.4(12) | 21.6(11) | -4.9(12) |
| C175 | 57.6(14) | 65.2(16) | 37.1(12) | -20.2(11) | 29.6(11) | -21.7(12) |
| C11 | 48.8(13) | 50.7(15) | 54.5(15) | 7.2(12) | 15.0(11) | 1.2(11) |
| C177 | 80.5(18) | 36.0(12) | 55.5(15) | 0.4(11) | 40.9(14) | -0.5(12) |
| C89 | 50.8(13) | 47.2(14) | 45.7(13) | -2.5(11) | 15.4(11) | -13.6(11) |
| C179 | 45.9(13) | 82(2) | 49.8(14) | -28.5(14) | 29.2(11) | -21.4(13) |
| C45 | 67.9(17) | 53.1(16) | 51.9(15) | -7.8(12) | 18.5(13) | -19.9(13) |
| C181 | 51.1(14) | 46.3(14) | 60.7(16) | 6.2(12) | 3.4(12) | -7.4(12) |
| C91 | 50.3(14) | 85(2) | 59.3(17) | -38.4(16) | 24.8(13) | -12.8(14) |
| C183 | 60.1(16) | 90(2) | 49.7(15) | -38.1(15) | 32.2(13) | -24.4(15) |
| C23 | 52.8(15) | 99(2) | 54.7(16) | -13.8(16) | 24.1(13) | 19.4(16) |
| C93 | 51.1(15) | 66.0(18) | 73.4(19) | 9.4(15) | 31.1(14) | 20.7(13) |
| C189 | 117(3) | 31.8(16) | 62(2) | 10.3(15) | 67(2) | 13.5(18) |
| C95 | 56.9(17) | 78(2) | 94(2) | -17.8(19) | 33.5(17) | -26.8(16) |
| C191 | 58.1(16) | 52.8(16) | 90(2) | 1.5(15) | 31.5(16) | 8.4(13) |
| C3 | 55.2(17) | 75(2) | 86(2) | 16.3(18) | 16.8(16) | 25.2(16) |
| C193 | 93(2) | 113(3) | 69(2) | -52(2) | 41.7(19) | -55(2) |
| C97 | 60.9(18) | 89(2) | 82(2) | -11.1(19) | 29.3(16) | -35.8(17) |
| C195 | 57.5(18) | 104(3) | 95(3) | 17(2) | 8.9(18) | -31.9(19) |
| C49 | 174(5) | 34.9(17) | 107(3) | -18.5(19) | 111(4) | -29(2) |
| C197 | 82(2) | 171(5) | 71(2) | -21(3) | 41.8(19) | 39(3) |
| C99 | 69(2) | 211(6) | 131(4) | -126(4) | 58(2) | -73(3) |
| C199 | 74(2) | 82(3) | 135(4) | 32(3) | 36(2) | 21(2) |
| C101 | 62(3) | 80(3) | 50(2) | -7(2) | 15(2) | -34(3) |
| C203 | 99(3) | 83(3) | 138(4) | 13(3) | 40(3) | 0(2) |
| C51 | 47(3) | 62(3) | 59(3) | 3(2) | 18(2) | 9(2) |
| C207 | 35(3) | 46(3) | 51(3) | -20(2) | 21(2) | -24(2) |
| C171 | 50.7(13) | 53.1(14) | 41.4(12) | 4.8(10) | 19.1(10) | -18.1(11) |
| C185 | 79.8(18) | 63.7(16) | 48.5(14) | -2.3(12) | 32.6(13) | -34.5(14) |
| C205 | 82(3) | 83(4) | 60(3) | 21(3) | 14(2) | -19(3) |
| C210 | 94(5) | 102(5) | 73(4) | -3(3) | 43(3) | 9(4) |
| C211 | 80(4) | 88(4) | 62(3) | 5(3) | 35(3) | -25(3) |
| C1A | 127(8) | 168(9) | 130(7) | 22(7) | 68(6) | -22(7) |
| C212 | 63(4) | 79(5) | 60(3) | -17(3) | 28(3) | -9(4) |
| Cl3A | 61(3) | 93(3) | 51.7(17) | -4.5(14) | 29.9(14) | 5.9(15) |
| Cl1A | 46.3(12) | 82.8(15) | 39.5(10) | -1.2(8) | 4.4(9) | -1.8(9) |
| Cl8A | 80.1(17) | 41.3(12) | 82(2) | -23.9(12) | 40.2(14) | -11.4(12) |
| C51A | 44(4) | 64(4) | 77(5) | 17(4) | 13(3) | 23(4) |
| Cl9A | 173(6) | 115(4) | 60.0(18) | 27(2) | -5(3) | -80(4) |
| Cl5A | 181(6) | 120(4) | 127(4) | -7(3) | 93(4) | -16(4) |
| C213 | 55(5) | 67(5) | 63(5) | -3(4) | 25(4) | -22(4) |
| Cl4A | 80(2) | 143(4) | 67.6(19) | 48(2) | 43.5(15) | 60(3) |
| Cl7A | 99(4) | 99(4) | 63(2) | 18(2) | 47(2) | 13(3) |
| Cl2A | 149(5) | 113(4) | 149(5) | 67(4) | 100(4) | 26(4) |
| C25 | 60(3) | 53(3) | 62(3) | -10(2) | 21(2) | -16(2) |
| C103 | 49(3) | 95(5) | 90(4) | -3(3) | 31(3) | -27(3) |
| C25A | 48(6) | 67(7) | 91(7) | -31(5) | 23(5) | -17(5) |
| C214 | 111(11) | 125(11) | 110(9) | -28(7) | 63(8) | -78(9) |
| C19A | 56(18) | 51(12) | 37(14) | -10(11) | -21(13) | 18(11) |
| C215 | 78(12) | 36(10) | 53(11) | 12(8) | 34(10) | -5(8) |
| C1B | 93(15) | 38(9) | 22(9) | -18(7) | 37(10) | -44(10) |
| C49A | 48(12) | 38(13) | 28(10) | -15(9) | 14(9) | -12(9) |
| C187 | 82(5) | 77(4) | 86(5) | -31(4) | 57(4) | -42(4) |
| C209 | 97(4) | 76(4) | 147(6) | 7(4) | 41(4) | -33(3) |
| C216 | 33(6) | 72(9) | 39(6) | 25(6) | 9(5) | -3(5) |
| C1C | 90(9) | 121(13) | 69(7) | -26(8) | 45(7) | -58(9) |
| C47 | 40(4) | 74(5) | 52(3) | -5(4) | 20(3) | -13(3) |
| C201 | 51(2) | 56(3) | 74(3) | 11(2) | 34(2) | 6.3(18) |
| C217 | 49(5) | 68(6) | 79(6) | 5(5) | 18(4) | -11(4) |
| C47A | 38(7) | 56(7) | 81(9) | 9(6) | 6(6) | -2(5) |

Table 4 Bond Lengths for z.

| Atom | Atom | Length/Å |  | Atom | Atom | Length/Å |
| --- | --- | --- | --- | --- | --- | --- |
| S1 | C74 | 1.7772(19) |  | C136 | C172 | 1.415(3) |
| S1 | C86 | 1.7795(18) |  | C138 | C33 | 1.406(3) |
| S4 | C130 | 1.783(2) |  | C140 | C168 | 1.404(3) |
| S4 | C198 | 1.7800(19) |  | C140 | C111 | 1.400(3) |
| S6 | C148 | 1.775(2) |  | C142 | C105 | 1.399(3) |
| S6 | C182 | 1.7788(19) |  | C144 | C13 | 1.403(3) |
| S8 | C158 | 1.780(2) |  | C144 | C31 | 1.554(3) |
| S8 | C206 | 1.7781(19) |  | C146 | C194 | 1.394(3) |
| S9 | C132 | 1.7803(19) |  | C148 | C196 | 1.420(3) |
| S9 | C192 | 1.777(2) |  | C152 | C184 | 1.397(3) |
| S5 | C208 | 1.778(2) |  | C152 | C115 | 1.556(3) |
| S5 | C55 | 1.781(2) |  | C154 | C198 | 1.401(3) |
| S3 | C170 | 1.782(2) |  | C156 | C182 | 1.403(3) |
| S3 | C105 | 1.784(2) |  | C158 | C160 | 1.419(3) |
| S7 | C15 | 1.7793(19) |  | C160 | C174 | 1.394(3) |
| S7 | C61 | 1.784(2) |  | C162 | C174 | 1.407(3) |
| S2 | C188 | 1.783(2) |  | C164 | C61 | 1.402(3) |
| S2 | C111 | 1.778(2) |  | C166 | C196 | 1.393(3) |
| Cl1 | C207 | 1.777(7) |  | C166 | C59 | 1.553(3) |
| Cl4 | C101 | 1.813(7) |  | C168 | C172 | 1.386(3) |
| Cl6 | C51 | 1.728(5) |  | C170 | C139 | 1.424(3) |
| Cl6 | C51A | 1.748(8) |  | C172 | C113 | 1.402(3) |
| Cl8 | C207 | 1.692(7) |  | C174 | C7 | 1.557(3) |
| Cl9 | C51 | 1.683(5) |  | C176 | C204 | 1.398(3) |
| Cl5 | C51 | 1.678(5) |  | C176 | C57 | 1.561(3) |
| Cl3 | C207 | 1.692(8) |  | C178 | C186 | 1.395(3) |
| Cl7 | C101 | 1.564(7) |  | C180 | C7 | 1.563(3) |
| Cl2 | C101 | 1.646(6) |  | C180 | C29 | 1.396(3) |
| C1 | C6 | 1.423(2) |  | C182 | C202 | 1.412(3) |
| C1 | C10 | 1.449(2) |  | C184 | C206 | 1.414(3) |
| C1 | C46 | 1.423(2) |  | C186 | C57 | 1.556(3) |
| C4 | C18 | 1.423(2) |  | C188 | C135 | 1.420(3) |
| C4 | C32 | 1.424(2) |  | C190 | C192 | 1.421(3) |
| C4 | C54 | 1.447(2) |  | C190 | C53 | 1.394(3) |
| C6 | C24 | 1.427(2) |  | C194 | C63 | 1.559(3) |
| C6 | C50 | 1.445(3) |  | C198 | C13 | 1.415(3) |
| C8 | C16 | 1.423(2) |  | C200 | C121 | 1.394(3) |
| C8 | C24 | 1.421(3) |  | C204 | C105 | 1.423(3) |
| C8 | C90 | 1.452(2) |  | C208 | C119 | 1.424(3) |
| C10 | C20 | 1.403(2) |  | C53 | C115 | 1.559(3) |
| C10 | C78 | 1.390(3) |  | C107 | C109 | 1.397(3) |
| C12 | C40 | 1.424(2) |  | C107 | C59 | 1.564(3) |
| C12 | C42 | 1.426(2) |  | C27 | C133 | 1.559(3) |
| C12 | C134 | 1.452(3) |  | C109 | C61 | 1.422(3) |
| C14 | C20 | 1.412(2) |  | C55 | C29 | 1.420(3) |
| C14 | C74 | 1.398(2) |  | C111 | C117 | 1.420(3) |
| C14 | C80 | 1.403(2) |  | C7 | C17 | 1.539(3) |
| C16 | C36 | 1.449(3) |  | C7 | C77 | 1.547(3) |
| C16 | C46 | 1.424(2) |  | C113 | C117 | 1.398(3) |
| C18 | C26 | 1.424(2) |  | C113 | C31 | 1.560(3) |
| C18 | C84 | 1.451(2) |  | C57 | C65 | 1.540(3) |
| C20 | C86 | 1.398(3) |  | C57 | C141 | 1.553(3) |
| C22 | C38 | 1.447(2) |  | C115 | C129 | 1.547(3) |
| C22 | C58 | 1.390(3) |  | C115 | C9 | 1.541(3) |
| C22 | C62 | 1.403(2) |  | C59 | C149 | 1.534(3) |
| C24 | C82 | 1.448(2) |  | C59 | C39 | 1.556(3) |
| C26 | C98 | 1.453(2) |  | C119 | C125 | 1.396(3) |
| C26 | C150 | 1.422(2) |  | C15 | C127 | 1.423(3) |
| C28 | C30 | 1.388(3) |  | C121 | C155 | 1.560(3) |
| C28 | C58 | 1.413(3) |  | C123 | C139 | 1.400(3) |
| C28 | C125 | 1.408(3) |  | C123 | C37 | 1.560(3) |
| C30 | C32 | 1.450(2) |  | C31 | C67 | 1.554(3) |
| C30 | C72 | 1.402(3) |  | C31 | C35 | 1.540(3) |
| C32 | C38 | 1.420(2) |  | C125 | C155 | 1.563(3) |
| C34 | C40 | 1.422(3) |  | C63 | C69 | 1.537(3) |
| C34 | C64 | 1.450(2) |  | C63 | C19 | 1.551(3) |
| C34 | C76 | 1.427(2) |  | C63 | C19A | 1.529(15) |
| C36 | C70 | 1.385(3) |  | C127 | C2 | 1.398(3) |
| C36 | C156 | 1.404(2) |  | C2 | C133 | 1.565(3) |
| C38 | C150 | 1.427(2) |  | C129 | C73 | 1.518(3) |
| C40 | C88 | 1.450(2) |  | C65 | C81 | 1.521(3) |
| C42 | C44 | 1.426(3) |  | C131 | C67 | 1.522(3) |
| C42 | C168 | 1.445(2) |  | C131 | C137 | 1.523(3) |
| C44 | C48 | 1.448(2) |  | C33 | C135 | 1.396(3) |
| C44 | C76 | 1.421(2) |  | C33 | C37 | 1.562(3) |
| C46 | C80 | 1.451(2) |  | C133 | C151 | 1.543(3) |
| C48 | C110 | 1.405(3) |  | C133 | C153 | 1.552(3) |
| C48 | C138 | 1.387(3) |  | C17 | C79 | 1.525(3) |
| C50 | C56 | 1.385(3) |  | C137 | C163 | 1.514(4) |
| C50 | C112 | 1.407(2) |  | C69 | C71 | 1.516(3) |
| C52 | C76 | 1.451(3) |  | C35 | C147 | 1.513(3) |
| C52 | C104 | 1.399(3) |  | C141 | C75 | 1.525(3) |
| C52 | C118 | 1.386(3) |  | C71 | C43 | 1.508(3) |
| C54 | C122 | 1.406(3) |  | C143 | C145 | 1.521(3) |
| C54 | C126 | 1.388(3) |  | C143 | C153 | 1.541(3) |
| C56 | C78 | 1.410(2) |  | C9 | C157 | 1.509(3) |
| C56 | C2 | 1.402(3) |  | C145 | C87 | 1.521(3) |
| C58 | C121 | 1.406(3) |  | C73 | C5 | 1.522(3) |
| C60 | C90 | 1.385(3) |  | C147 | C173 | 1.527(3) |
| C60 | C108 | 1.406(3) |  | C37 | C161 | 1.539(3) |
| C60 | C166 | 1.410(3) |  | C37 | C171 | 1.542(3) |
| C62 | C68 | 1.411(3) |  | C149 | C21 | 1.522(3) |
| C62 | C132 | 1.395(3) |  | C75 | C165 | 1.522(3) |
| C64 | C102 | 1.392(2) |  | C151 | C85 | 1.519(3) |
| C64 | C142 | 1.396(3) |  | C19 | C167 | 1.516(6) |
| C66 | C68 | 1.402(2) |  | C77 | C179 | 1.507(3) |
| C66 | C114 | 1.388(3) |  | C155 | C159 | 1.559(3) |
| C66 | C150 | 1.446(2) |  | C155 | C41 | 1.526(3) |
| C68 | C192 | 1.400(2) |  | C39 | C175 | 1.520(3) |
| C70 | C92 | 1.415(2) |  | C157 | C187 | 1.523(7) |
| C70 | C124 | 1.402(3) |  | C157 | C216 | 1.520(13) |
| C72 | C122 | 1.405(3) |  | C79 | C45 | 1.523(4) |
| C72 | C208 | 1.402(3) |  | C159 | C83 | 1.530(3) |
| C74 | C146 | 1.424(3) |  | C5 | C177 | 1.513(4) |
| C78 | C27 | 1.406(3) |  | C161 | C11 | 1.528(4) |
| C80 | C92 | 1.387(3) |  | C81 | C93 | 1.516(3) |
| C82 | C108 | 1.390(3) |  | C41 | C89 | 1.517(4) |
| C82 | C164 | 1.400(3) |  | C165 | C169 | 1.513(4) |
| C84 | C116 | 1.405(3) |  | C83 | C23 | 1.522(4) |
| C84 | C162 | 1.384(3) |  | C167 | C189 | 1.514(4) |
| C86 | C120 | 1.416(3) |  | C21 | C25 | 1.514(5) |
| C88 | C94 | 1.385(3) |  | C21 | C25A | 1.607(10) |
| C88 | C100 | 1.406(3) |  | C85 | C47 | 1.541(7) |
| C90 | C96 | 1.403(3) |  | C85 | C47A | 1.546(14) |
| C92 | C194 | 1.405(3) |  | C43 | C95 | 1.507(5) |
| C94 | C102 | 1.410(3) |  | C173 | C191 | 1.507(4) |
| C94 | C186 | 1.410(2) |  | C175 | C183 | 1.527(3) |
| C96 | C148 | 1.402(3) |  | C11 | C199 | 1.509(4) |
| C96 | C156 | 1.403(3) |  | C89 | C181 | 1.530(4) |
| C98 | C106 | 1.383(3) |  | C179 | C91 | 1.503(3) |
| C98 | C128 | 1.404(2) |  | C45 | C97 | 1.493(5) |
| C100 | C130 | 1.402(3) |  | C181 | C195 | 1.500(5) |
| C100 | C154 | 1.406(3) |  | C91 | C99 | 1.487(4) |
| C102 | C176 | 1.403(3) |  | C183 | C193 | 1.513(4) |
| C104 | C142 | 1.412(3) |  | C23 | C197 | 1.525(4) |
| C104 | C170 | 1.394(3) |  | C93 | C3 | 1.495(5) |
| C106 | C114 | 1.415(3) |  | C189 | C49 | 1.517(6) |
| C106 | C152 | 1.405(3) |  | C199 | C203 | 1.372(7) |
| C108 | C107 | 1.404(3) |  | C171 | C185 | 1.605(3) |
| C110 | C140 | 1.406(3) |  | C185 | C205 | 1.825(5) |
| C110 | C188 | 1.397(3) |  | C185 | C211 | 1.541(8) |
| C112 | C164 | 1.405(3) |  | C205 | C210 | 1.466(6) |
| C112 | C15 | 1.401(3) |  | C211 | C1A | 1.398(11) |
| C114 | C53 | 1.406(3) |  | C212 | Cl3A | 1.699(8) |
| C116 | C128 | 1.399(3) |  | C212 | Cl1A | 1.682(8) |
| C116 | C158 | 1.404(3) |  | C212 | Cl8A | 1.667(8) |
| C118 | C138 | 1.410(3) |  | C51A | Cl9A | 1.657(10) |
| C118 | C123 | 1.402(3) |  | C51A | Cl5A | 1.670(10) |
| C120 | C27 | 1.398(3) |  | C213 | Cl4A | 1.785(9) |
| C122 | C55 | 1.401(3) |  | C213 | Cl7A | 1.564(11) |
| C124 | C202 | 1.397(3) |  | C213 | Cl2A | 1.629(9) |
| C124 | C63 | 1.555(3) |  | C25 | C103 | 1.493(8) |
| C126 | C162 | 1.414(3) |  | C25A | C214 | 1.510(15) |
| C126 | C180 | 1.402(3) |  | C19A | C215 | 1.519(15) |
| C128 | C206 | 1.405(3) |  | C215 | C1B | 1.508(13) |
| C130 | C178 | 1.418(3) |  | C1B | C49A | 1.516(14) |
| C132 | C200 | 1.420(3) |  | C187 | C209 | 1.338(10) |
| C134 | C136 | 1.381(3) |  | C216 | C1C | 1.385(18) |
| C134 | C154 | 1.404(3) |  | C47 | C201 | 1.505(11) |
| C136 | C144 | 1.405(3) |  | C217 | C47A | 1.501(15) |

Table 5 Bond Angles for z.

| Atom | Atom | Atom | Angle/˚ |  | Atom | Atom | Atom | Angle/˚ |
| --- | --- | --- | --- | --- | --- | --- | --- | --- |
| C74 | S1 | C86 | 93.17(8) |  | C82 | C164 | C112 | 120.39(16) |
| C198 | S4 | C130 | 93.03(9) |  | C82 | C164 | C61 | 122.05(17) |
| C148 | S6 | C182 | 92.95(9) |  | C61 | C164 | C112 | 114.68(18) |
| C206 | S8 | C158 | 92.98(9) |  | C60 | C166 | C59 | 106.87(16) |
| C192 | S9 | C132 | 93.23(9) |  | C196 | C166 | C60 | 119.33(17) |
| C208 | S5 | C55 | 93.27(9) |  | C196 | C166 | C59 | 132.80(17) |
| C170 | S3 | C105 | 92.93(9) |  | C140 | C168 | C42 | 120.95(17) |
| C15 | S7 | C61 | 93.17(9) |  | C172 | C168 | C42 | 120.72(16) |
| C111 | S2 | C188 | 93.19(9) |  | C172 | C168 | C140 | 114.98(16) |
| C6 | C1 | C10 | 118.59(16) |  | C104 | C170 | S3 | 108.38(14) |
| C46 | C1 | C6 | 120.33(15) |  | C104 | C170 | C139 | 121.18(18) |
| C46 | C1 | C10 | 118.80(15) |  | C139 | C170 | S3 | 129.28(16) |
| C18 | C4 | C32 | 119.77(15) |  | C168 | C172 | C136 | 120.19(16) |
| C18 | C4 | C54 | 119.10(15) |  | C168 | C172 | C113 | 125.50(18) |
| C32 | C4 | C54 | 118.86(16) |  | C113 | C172 | C136 | 110.86(17) |
| C1 | C6 | C24 | 119.55(16) |  | C160 | C174 | C162 | 118.92(17) |
| C1 | C6 | C50 | 119.09(16) |  | C160 | C174 | C7 | 132.15(17) |
| C24 | C6 | C50 | 119.03(15) |  | C162 | C174 | C7 | 107.53(16) |
| C16 | C8 | C90 | 118.39(16) |  | C102 | C176 | C57 | 107.12(16) |
| C24 | C8 | C16 | 120.32(15) |  | C204 | C176 | C102 | 119.26(17) |
| C24 | C8 | C90 | 118.85(15) |  | C204 | C176 | C57 | 132.16(17) |
| C20 | C10 | C1 | 120.92(16) |  | C186 | C178 | C130 | 117.65(16) |
| C78 | C10 | C1 | 120.44(15) |  | C126 | C180 | C7 | 107.38(17) |
| C78 | C10 | C20 | 115.28(16) |  | C29 | C180 | C126 | 119.10(18) |
| C40 | C12 | C42 | 119.89(16) |  | C29 | C180 | C7 | 132.04(17) |
| C40 | C12 | C134 | 119.11(15) |  | C156 | C182 | S6 | 108.54(14) |
| C42 | C12 | C134 | 118.61(15) |  | C156 | C182 | C202 | 121.12(17) |
| C74 | C14 | C20 | 114.65(16) |  | C202 | C182 | S6 | 128.92(15) |
| C74 | C14 | C80 | 121.93(16) |  | C152 | C184 | C206 | 117.91(17) |
| C80 | C14 | C20 | 120.28(15) |  | C94 | C186 | C57 | 107.04(16) |
| C8 | C16 | C36 | 118.96(15) |  | C178 | C186 | C94 | 119.39(17) |
| C8 | C16 | C46 | 119.66(16) |  | C178 | C186 | C57 | 132.49(16) |
| C46 | C16 | C36 | 118.94(15) |  | C110 | C188 | S2 | 108.06(15) |
| C4 | C18 | C26 | 120.07(15) |  | C110 | C188 | C135 | 121.23(18) |
| C4 | C18 | C84 | 118.80(15) |  | C135 | C188 | S2 | 129.10(16) |
| C26 | C18 | C84 | 118.68(16) |  | C53 | C190 | C192 | 117.63(16) |
| C10 | C20 | C14 | 120.17(16) |  | C68 | C192 | S9 | 108.27(14) |
| C86 | C20 | C10 | 121.89(17) |  | C68 | C192 | C190 | 121.31(17) |
| C86 | C20 | C14 | 114.91(15) |  | C190 | C192 | S9 | 129.04(14) |
| C58 | C22 | C38 | 120.51(16) |  | C92 | C194 | C63 | 107.82(15) |
| C58 | C22 | C62 | 115.08(16) |  | C146 | C194 | C92 | 118.93(17) |
| C62 | C22 | C38 | 121.02(16) |  | C146 | C194 | C63 | 131.77(17) |
| C6 | C24 | C82 | 118.49(16) |  | C166 | C196 | C148 | 117.79(17) |
| C8 | C24 | C6 | 120.07(15) |  | C154 | C198 | S4 | 108.42(14) |
| C8 | C24 | C82 | 119.19(15) |  | C154 | C198 | C13 | 121.28(17) |
| C18 | C26 | C98 | 118.86(15) |  | C13 | C198 | S4 | 128.86(14) |
| C150 | C26 | C18 | 120.12(15) |  | C121 | C200 | C132 | 117.54(17) |
| C150 | C26 | C98 | 118.74(15) |  | C124 | C202 | C182 | 117.49(17) |
| C30 | C28 | C58 | 120.42(17) |  | C176 | C204 | C105 | 117.67(17) |
| C30 | C28 | C125 | 125.05(17) |  | C128 | C206 | S8 | 108.44(14) |
| C125 | C28 | C58 | 111.08(17) |  | C128 | C206 | C184 | 121.09(16) |
| C28 | C30 | C32 | 120.63(16) |  | C184 | C206 | S8 | 129.13(15) |
| C28 | C30 | C72 | 115.16(17) |  | C72 | C208 | S5 | 108.09(15) |
| C72 | C30 | C32 | 121.06(16) |  | C72 | C208 | C119 | 120.94(18) |
| C4 | C32 | C30 | 118.64(15) |  | C119 | C208 | S5 | 129.91(15) |
| C38 | C32 | C4 | 120.31(16) |  | C144 | C13 | C198 | 117.54(17) |
| C38 | C32 | C30 | 118.84(15) |  | C142 | C105 | S3 | 108.43(14) |
| C40 | C34 | C64 | 119.32(15) |  | C142 | C105 | C204 | 120.72(18) |
| C40 | C34 | C76 | 120.00(15) |  | C204 | C105 | S3 | 129.60(15) |
| C76 | C34 | C64 | 118.32(16) |  | C114 | C53 | C115 | 107.83(15) |
| C70 | C36 | C16 | 120.77(16) |  | C190 | C53 | C114 | 118.84(17) |
| C70 | C36 | C156 | 114.93(16) |  | C190 | C53 | C115 | 131.90(17) |
| C156 | C36 | C16 | 120.94(16) |  | C108 | C107 | C59 | 107.01(17) |
| C32 | C38 | C22 | 119.03(16) |  | C109 | C107 | C108 | 119.22(17) |
| C32 | C38 | C150 | 119.88(15) |  | C109 | C107 | C59 | 132.37(17) |
| C150 | C38 | C22 | 118.81(15) |  | C78 | C27 | C133 | 107.73(15) |
| C12 | C40 | C88 | 118.36(16) |  | C120 | C27 | C78 | 118.74(17) |
| C34 | C40 | C12 | 120.19(15) |  | C120 | C27 | C133 | 132.24(18) |
| C34 | C40 | C88 | 118.92(15) |  | C107 | C109 | C61 | 117.83(17) |
| C12 | C42 | C44 | 119.80(15) |  | C122 | C55 | S5 | 108.32(15) |
| C12 | C42 | C168 | 118.99(16) |  | C122 | C55 | C29 | 120.57(18) |
| C44 | C42 | C168 | 118.96(16) |  | C29 | C55 | S5 | 129.76(15) |
| C42 | C44 | C48 | 118.72(15) |  | C140 | C111 | S2 | 108.15(15) |
| C76 | C44 | C42 | 120.32(15) |  | C140 | C111 | C117 | 121.12(18) |
| C76 | C44 | C48 | 118.78(17) |  | C117 | C111 | S2 | 129.38(15) |
| C1 | C46 | C16 | 120.06(15) |  | C174 | C7 | C180 | 101.84(14) |
| C1 | C46 | C80 | 119.04(15) |  | C17 | C7 | C174 | 111.86(16) |
| C16 | C46 | C80 | 118.63(15) |  | C17 | C7 | C180 | 111.41(17) |
| C110 | C48 | C44 | 120.77(17) |  | C17 | C7 | C77 | 108.46(15) |
| C138 | C48 | C44 | 120.27(16) |  | C77 | C7 | C174 | 111.28(17) |
| C138 | C48 | C110 | 115.67(17) |  | C77 | C7 | C180 | 111.95(16) |
| C56 | C50 | C6 | 120.84(15) |  | C172 | C113 | C31 | 107.68(16) |
| C56 | C50 | C112 | 115.08(18) |  | C117 | C113 | C172 | 118.51(18) |
| C112 | C50 | C6 | 120.77(16) |  | C117 | C113 | C31 | 132.42(17) |
| C104 | C52 | C76 | 121.31(16) |  | C186 | C57 | C176 | 101.95(14) |
| C118 | C52 | C76 | 120.37(16) |  | C65 | C57 | C176 | 112.44(17) |
| C118 | C52 | C104 | 115.16(17) |  | C65 | C57 | C186 | 112.57(16) |
| C122 | C54 | C4 | 120.82(16) |  | C65 | C57 | C141 | 108.41(15) |
| C126 | C54 | C4 | 120.49(16) |  | C141 | C57 | C176 | 111.15(16) |
| C126 | C54 | C122 | 115.39(16) |  | C141 | C57 | C186 | 110.26(16) |
| C50 | C56 | C78 | 120.15(17) |  | C152 | C115 | C53 | 101.66(14) |
| C50 | C56 | C2 | 125.05(17) |  | C129 | C115 | C152 | 111.67(16) |
| C2 | C56 | C78 | 111.32(16) |  | C129 | C115 | C53 | 111.83(15) |
| C22 | C58 | C28 | 120.56(16) |  | C9 | C115 | C152 | 111.57(16) |
| C22 | C58 | C121 | 125.02(16) |  | C9 | C115 | C53 | 111.99(16) |
| C121 | C58 | C28 | 111.00(17) |  | C9 | C115 | C129 | 108.11(16) |
| C90 | C60 | C108 | 120.91(16) |  | C180 | C29 | C55 | 118.11(17) |
| C90 | C60 | C166 | 124.42(18) |  | C113 | C117 | C111 | 117.87(17) |
| C108 | C60 | C166 | 111.26(17) |  | C166 | C59 | C107 | 101.97(14) |
| C22 | C62 | C68 | 119.98(16) |  | C166 | C59 | C39 | 109.75(17) |
| C132 | C62 | C22 | 121.83(17) |  | C149 | C59 | C166 | 112.96(16) |
| C132 | C62 | C68 | 114.99(16) |  | C149 | C59 | C107 | 112.01(17) |
| C102 | C64 | C34 | 119.92(16) |  | C149 | C59 | C39 | 108.84(15) |
| C102 | C64 | C142 | 115.45(16) |  | C39 | C59 | C107 | 111.20(16) |
| C142 | C64 | C34 | 121.39(16) |  | C125 | C119 | C208 | 117.77(18) |
| C68 | C66 | C150 | 120.94(16) |  | C112 | C15 | S7 | 108.05(14) |
| C114 | C66 | C68 | 115.13(16) |  | C112 | C15 | C127 | 121.51(17) |
| C114 | C66 | C150 | 120.49(16) |  | C127 | C15 | S7 | 129.01(16) |
| C66 | C68 | C62 | 120.35(16) |  | C58 | C121 | C155 | 107.20(16) |
| C192 | C68 | C62 | 114.73(16) |  | C200 | C121 | C58 | 118.90(17) |
| C192 | C68 | C66 | 121.88(16) |  | C200 | C121 | C155 | 132.78(17) |
| C36 | C70 | C92 | 120.38(16) |  | C164 | C61 | S7 | 108.21(15) |
| C36 | C70 | C124 | 124.87(16) |  | C164 | C61 | C109 | 120.76(19) |
| C124 | C70 | C92 | 111.05(16) |  | C109 | C61 | S7 | 129.78(16) |
| C30 | C72 | C122 | 120.06(17) |  | C118 | C123 | C37 | 107.25(17) |
| C30 | C72 | C208 | 122.07(18) |  | C139 | C123 | C118 | 119.30(18) |
| C208 | C72 | C122 | 115.24(17) |  | C139 | C123 | C37 | 132.31(19) |
| C14 | C74 | S1 | 108.47(13) |  | C144 | C31 | C113 | 101.73(14) |
| C14 | C74 | C146 | 121.23(16) |  | C67 | C31 | C144 | 110.02(17) |
| C146 | C74 | S1 | 128.77(14) |  | C67 | C31 | C113 | 112.14(16) |
| C34 | C76 | C52 | 118.69(16) |  | C35 | C31 | C144 | 112.04(16) |
| C44 | C76 | C34 | 119.80(16) |  | C35 | C31 | C113 | 112.28(17) |
| C44 | C76 | C52 | 119.18(16) |  | C35 | C31 | C67 | 108.56(16) |
| C10 | C78 | C56 | 120.84(16) |  | C28 | C125 | C155 | 106.97(16) |
| C10 | C78 | C27 | 124.79(16) |  | C119 | C125 | C28 | 118.86(18) |
| C27 | C78 | C56 | 110.88(16) |  | C119 | C125 | C155 | 133.28(18) |
| C14 | C80 | C46 | 120.64(16) |  | C124 | C63 | C194 | 101.46(14) |
| C92 | C80 | C14 | 115.14(15) |  | C69 | C63 | C124 | 112.12(16) |
| C92 | C80 | C46 | 120.68(15) |  | C69 | C63 | C194 | 111.96(16) |
| C108 | C82 | C24 | 120.05(17) |  | C69 | C63 | C19 | 107.7(2) |
| C108 | C82 | C164 | 115.65(16) |  | C19 | C63 | C124 | 110.2(2) |
| C164 | C82 | C24 | 121.05(16) |  | C19 | C63 | C194 | 113.4(2) |
| C116 | C84 | C18 | 120.80(16) |  | C19A | C63 | C124 | 112.7(18) |
| C162 | C84 | C18 | 120.38(16) |  | C19A | C63 | C194 | 99.6(11) |
| C162 | C84 | C116 | 115.42(16) |  | C19A | C63 | C69 | 117.2(18) |
| C20 | C86 | S1 | 108.24(13) |  | C2 | C127 | C15 | 117.05(18) |
| C20 | C86 | C120 | 121.26(16) |  | C56 | C2 | C133 | 107.48(16) |
| C120 | C86 | S1 | 129.00(14) |  | C127 | C2 | C56 | 119.39(18) |
| C94 | C88 | C40 | 120.15(17) |  | C127 | C2 | C133 | 131.70(19) |
| C94 | C88 | C100 | 115.31(16) |  | C73 | C129 | C115 | 116.19(16) |
| C100 | C88 | C40 | 121.08(16) |  | C81 | C65 | C57 | 116.58(16) |
| C60 | C90 | C8 | 120.19(17) |  | C67 | C131 | C137 | 112.43(17) |
| C60 | C90 | C96 | 115.35(16) |  | C138 | C33 | C37 | 107.59(16) |
| C96 | C90 | C8 | 120.99(16) |  | C135 | C33 | C138 | 118.68(19) |
| C80 | C92 | C70 | 120.57(16) |  | C135 | C33 | C37 | 132.45(19) |
| C80 | C92 | C194 | 125.06(16) |  | C27 | C133 | C2 | 101.65(15) |
| C194 | C92 | C70 | 110.52(16) |  | C151 | C133 | C27 | 112.12(16) |
| C88 | C94 | C102 | 121.05(16) |  | C151 | C133 | C2 | 110.68(16) |
| C88 | C94 | C186 | 124.48(17) |  | C151 | C133 | C153 | 110.40(17) |
| C186 | C94 | C102 | 110.96(16) |  | C153 | C133 | C27 | 111.69(16) |
| C90 | C96 | C156 | 120.33(16) |  | C153 | C133 | C2 | 110.00(16) |
| C148 | C96 | C90 | 122.27(17) |  | C131 | C67 | C31 | 115.99(16) |
| C148 | C96 | C156 | 114.76(17) |  | C33 | C135 | C188 | 118.04(18) |
| C106 | C98 | C26 | 120.61(16) |  | C79 | C17 | C7 | 116.15(16) |
| C106 | C98 | C128 | 115.54(16) |  | C163 | C137 | C131 | 112.77(19) |
| C128 | C98 | C26 | 120.63(16) |  | C71 | C69 | C63 | 116.77(17) |
| C88 | C100 | C154 | 120.34(16) |  | C123 | C139 | C170 | 117.13(19) |
| C130 | C100 | C88 | 122.12(17) |  | C147 | C35 | C31 | 116.84(17) |
| C130 | C100 | C154 | 114.70(17) |  | C75 | C141 | C57 | 116.83(16) |
| C64 | C102 | C94 | 120.63(16) |  | C43 | C71 | C69 | 112.5(2) |
| C64 | C102 | C176 | 124.45(18) |  | C145 | C143 | C153 | 112.04(19) |
| C176 | C102 | C94 | 111.21(16) |  | C157 | C9 | C115 | 116.16(18) |
| C52 | C104 | C142 | 119.83(17) |  | C143 | C145 | C87 | 113.2(2) |
| C170 | C104 | C52 | 122.31(17) |  | C129 | C73 | C5 | 112.46(18) |
| C170 | C104 | C142 | 115.06(17) |  | C35 | C147 | C173 | 111.3(2) |
| C98 | C106 | C114 | 120.52(16) |  | C123 | C37 | C33 | 101.79(16) |
| C98 | C106 | C152 | 124.75(16) |  | C161 | C37 | C123 | 111.76(18) |
| C152 | C106 | C114 | 110.87(16) |  | C161 | C37 | C33 | 111.77(17) |
| C82 | C108 | C60 | 120.78(16) |  | C161 | C37 | C171 | 111.76(19) |
| C82 | C108 | C107 | 124.41(19) |  | C171 | C37 | C123 | 107.81(17) |
| C107 | C108 | C60 | 111.06(17) |  | C171 | C37 | C33 | 111.48(19) |
| C48 | C110 | C140 | 120.41(17) |  | C21 | C149 | C59 | 115.79(17) |
| C188 | C110 | C48 | 121.54(18) |  | C165 | C75 | C141 | 112.48(17) |
| C188 | C110 | C140 | 115.02(17) |  | C85 | C151 | C133 | 115.59(18) |
| C164 | C112 | C50 | 120.12(18) |  | C167 | C19 | C63 | 116.0(3) |
| C15 | C112 | C50 | 121.81(18) |  | C143 | C153 | C133 | 114.95(17) |
| C15 | C112 | C164 | 115.26(17) |  | C179 | C77 | C7 | 116.78(17) |
| C66 | C114 | C106 | 120.55(16) |  | C121 | C155 | C125 | 101.74(15) |
| C66 | C114 | C53 | 125.07(17) |  | C159 | C155 | C121 | 109.17(17) |
| C53 | C114 | C106 | 110.77(16) |  | C159 | C155 | C125 | 108.07(16) |
| C128 | C116 | C84 | 120.36(16) |  | C41 | C155 | C121 | 112.41(16) |
| C128 | C116 | C158 | 115.05(17) |  | C41 | C155 | C125 | 114.40(18) |
| C158 | C116 | C84 | 121.74(17) |  | C41 | C155 | C159 | 110.60(17) |
| C52 | C118 | C138 | 120.36(18) |  | C175 | C39 | C59 | 116.71(16) |
| C52 | C118 | C123 | 124.80(18) |  | C9 | C157 | C187 | 116.5(4) |
| C123 | C118 | C138 | 111.60(17) |  | C9 | C157 | C216 | 107.0(8) |
| C27 | C120 | C86 | 117.91(17) |  | C45 | C79 | C17 | 113.01(19) |
| C72 | C122 | C54 | 120.30(16) |  | C83 | C159 | C155 | 115.38(18) |
| C55 | C122 | C54 | 122.09(17) |  | C177 | C5 | C73 | 112.8(2) |
| C55 | C122 | C72 | 114.67(18) |  | C11 | C161 | C37 | 114.82(19) |
| C70 | C124 | C63 | 107.76(15) |  | C93 | C81 | C65 | 112.6(2) |
| C202 | C124 | C70 | 119.34(17) |  | C89 | C41 | C155 | 116.22(19) |
| C202 | C124 | C63 | 131.66(17) |  | C169 | C165 | C75 | 114.1(2) |
| C54 | C126 | C162 | 120.36(16) |  | C23 | C83 | C159 | 113.1(2) |
| C54 | C126 | C180 | 124.62(18) |  | C189 | C167 | C19 | 112.8(3) |
| C180 | C126 | C162 | 111.18(16) |  | C149 | C21 | C25A | 108.1(5) |
| C98 | C128 | C206 | 121.60(17) |  | C25 | C21 | C149 | 115.7(3) |
| C116 | C128 | C98 | 120.47(16) |  | C151 | C85 | C47 | 118.2(4) |
| C116 | C128 | C206 | 114.68(16) |  | C151 | C85 | C47A | 103.1(7) |
| C100 | C130 | S4 | 108.46(14) |  | C95 | C43 | C71 | 113.6(2) |
| C100 | C130 | C178 | 120.94(17) |  | C191 | C173 | C147 | 112.9(2) |
| C178 | C130 | S4 | 129.28(14) |  | C39 | C175 | C183 | 112.07(19) |
| C62 | C132 | S9 | 108.22(14) |  | C199 | C11 | C161 | 115.6(3) |
| C62 | C132 | C200 | 121.54(17) |  | C41 | C89 | C181 | 111.8(2) |
| C200 | C132 | S9 | 128.74(15) |  | C91 | C179 | C77 | 113.7(2) |
| C136 | C134 | C12 | 120.51(16) |  | C97 | C45 | C79 | 115.4(3) |
| C136 | C134 | C154 | 115.25(16) |  | C195 | C181 | C89 | 112.1(3) |
| C154 | C134 | C12 | 120.84(16) |  | C99 | C91 | C179 | 116.3(3) |
| C134 | C136 | C144 | 125.10(17) |  | C193 | C183 | C175 | 113.6(2) |
| C134 | C136 | C172 | 120.93(17) |  | C83 | C23 | C197 | 112.8(3) |
| C144 | C136 | C172 | 110.89(16) |  | C3 | C93 | C81 | 114.7(3) |
| C48 | C138 | C118 | 121.01(17) |  | C167 | C189 | C49 | 113.9(3) |
| C48 | C138 | C33 | 124.73(17) |  | C203 | C199 | C11 | 119.9(4) |
| C33 | C138 | C118 | 110.66(18) |  | Cl7 | C101 | Cl4 | 116.5(4) |
| C168 | C140 | C110 | 120.09(16) |  | Cl7 | C101 | Cl2 | 120.5(3) |
| C111 | C140 | C110 | 114.91(17) |  | Cl2 | C101 | Cl4 | 114.5(4) |
| C111 | C140 | C168 | 121.93(18) |  | Cl9 | C51 | Cl6 | 114.5(3) |
| C64 | C142 | C104 | 120.26(16) |  | Cl5 | C51 | Cl6 | 115.4(3) |
| C64 | C142 | C105 | 122.38(17) |  | Cl5 | C51 | Cl9 | 118.1(3) |
| C105 | C142 | C104 | 114.61(18) |  | Cl8 | C207 | Cl1 | 115.3(4) |
| C136 | C144 | C31 | 107.66(16) |  | Cl8 | C207 | Cl3 | 115.2(4) |
| C13 | C144 | C136 | 118.83(17) |  | Cl3 | C207 | Cl1 | 113.2(4) |
| C13 | C144 | C31 | 132.39(17) |  | C37 | C171 | C185 | 113.5(2) |
| C194 | C146 | C74 | 117.60(16) |  | C171 | C185 | C205 | 101.3(3) |
| C96 | C148 | S6 | 108.69(14) |  | C211 | C185 | C171 | 114.9(4) |
| C96 | C148 | C196 | 120.70(18) |  | C210 | C205 | C185 | 101.2(4) |
| C196 | C148 | S6 | 129.48(15) |  | C1A | C211 | C185 | 122.2(9) |
| C26 | C150 | C38 | 119.86(15) |  | Cl1A | C212 | Cl3A | 117.1(5) |
| C26 | C150 | C66 | 119.06(15) |  | Cl8A | C212 | Cl3A | 115.1(5) |
| C38 | C150 | C66 | 118.75(15) |  | Cl8A | C212 | Cl1A | 118.2(5) |
| C106 | C152 | C115 | 107.86(15) |  | Cl9A | C51A | Cl6 | 117.2(5) |
| C184 | C152 | C106 | 118.92(17) |  | Cl9A | C51A | Cl5A | 118.0(6) |
| C184 | C152 | C115 | 131.82(17) |  | Cl5A | C51A | Cl6 | 117.7(5) |
| C134 | C154 | C100 | 120.04(16) |  | Cl7A | C213 | Cl4A | 118.1(6) |
| C198 | C154 | C100 | 115.03(16) |  | Cl7A | C213 | Cl2A | 122.4(7) |
| C198 | C154 | C134 | 121.89(17) |  | Cl2A | C213 | Cl4A | 116.8(5) |
| C96 | C156 | C36 | 120.06(17) |  | C103 | C25 | C21 | 113.0(5) |
| C182 | C156 | C36 | 122.11(17) |  | C214 | C25A | C21 | 113.2(9) |
| C182 | C156 | C96 | 114.73(16) |  | C215 | C19A | C63 | 119.6(19) |
| C116 | C158 | S8 | 108.31(14) |  | C1B | C215 | C19A | 113.9(17) |
| C116 | C158 | C160 | 121.03(17) |  | C215 | C1B | C49A | 122(2) |
| C160 | C158 | S8 | 129.30(15) |  | C209 | C187 | C157 | 118.2(7) |
| C174 | C160 | C158 | 117.89(17) |  | C1C | C216 | C157 | 125.9(15) |
| C84 | C162 | C126 | 120.86(16) |  | C201 | C47 | C85 | 109.7(7) |
| C84 | C162 | C174 | 124.90(17) |  | C217 | C47A | C85 | 108.5(11) |
| C174 | C162 | C126 | 110.86(16) |  |  |  |  |  |

Table 6 Torsion Angles for z.

| A | B | C | D | Angle/˚ |  | A | B | C | D | Angle/˚ |
| --- | --- | --- | --- | --- | --- | --- | --- | --- | --- | --- |
| S1 | C74 | C146 | C194 | 162.36(14) |  | C104 | C52 | C76 | C34 | -3.1(2) |
| S1 | C86 | C120 | C27 | -164.45(14) |  | C104 | C52 | C76 | C44 | 159.52(16) |
| S4 | C130 | C178 | C186 | -163.36(15) |  | C104 | C52 | C118 | C138 | -161.81(17) |
| S4 | C198 | C13 | C144 | 162.94(16) |  | C104 | C52 | C118 | C123 | -3.9(3) |
| S6 | C148 | C196 | C166 | 164.02(16) |  | C104 | C142 | C105 | S3 | -4.8(2) |
| S6 | C182 | C202 | C124 | -163.51(15) |  | C104 | C142 | C105 | C204 | 163.63(17) |
| S8 | C158 | C160 | C174 | 163.02(16) |  | C104 | C170 | C139 | C123 | 1.3(3) |
| S9 | C132 | C200 | C121 | -163.52(15) |  | C106 | C98 | C128 | C116 | 163.21(17) |
| S5 | C208 | C119 | C125 | 164.80(15) |  | C106 | C98 | C128 | C206 | 4.6(3) |
| S5 | C55 | C29 | C180 | -162.93(15) |  | C106 | C114 | C53 | C190 | -162.07(17) |
| S3 | C170 | C139 | C123 | -164.97(16) |  | C106 | C114 | C53 | C115 | 5.9(2) |
| S7 | C15 | C127 | C2 | 164.83(15) |  | C106 | C152 | C184 | C206 | -0.7(3) |
| S2 | C188 | C135 | C33 | 163.94(17) |  | C106 | C152 | C115 | C53 | 9.69(19) |
| S2 | C111 | C117 | C113 | -163.70(16) |  | C106 | C152 | C115 | C129 | -109.69(17) |
| C1 | C6 | C24 | C8 | 0.7(2) |  | C106 | C152 | C115 | C9 | 129.21(17) |
| C1 | C6 | C24 | C82 | 163.50(15) |  | C108 | C60 | C90 | C8 | 0.9(3) |
| C1 | C6 | C50 | C56 | -1.7(2) |  | C108 | C60 | C90 | C96 | 160.11(17) |
| C1 | C6 | C50 | C112 | -160.15(16) |  | C108 | C60 | C166 | C196 | -160.79(17) |
| C1 | C10 | C20 | C14 | 3.6(2) |  | C108 | C60 | C166 | C59 | 9.2(2) |
| C1 | C10 | C20 | C86 | -155.71(16) |  | C108 | C82 | C164 | C112 | 162.58(17) |
| C1 | C10 | C78 | C56 | -2.1(2) |  | C108 | C82 | C164 | C61 | 2.8(3) |
| C1 | C10 | C78 | C27 | 154.90(17) |  | C108 | C107 | C109 | C61 | -1.4(3) |
| C1 | C46 | C80 | C14 | 3.7(2) |  | C108 | C107 | C59 | C166 | 12.53(19) |
| C1 | C46 | C80 | C92 | 161.45(16) |  | C108 | C107 | C59 | C149 | 133.59(17) |
| C4 | C18 | C26 | C98 | 161.60(15) |  | C108 | C107 | C59 | C39 | -104.38(18) |
| C4 | C18 | C26 | C150 | -1.0(2) |  | C110 | C48 | C138 | C118 | 160.64(17) |
| C4 | C18 | C84 | C116 | -158.42(16) |  | C110 | C48 | C138 | C33 | 4.1(3) |
| C4 | C18 | C84 | C162 | -0.2(2) |  | C110 | C140 | C168 | C42 | 2.6(3) |
| C4 | C32 | C38 | C22 | -163.59(15) |  | C110 | C140 | C168 | C172 | 162.06(17) |
| C4 | C32 | C38 | C150 | -0.9(2) |  | C110 | C140 | C111 | S2 | 5.6(2) |
| C4 | C54 | C122 | C72 | 3.4(3) |  | C110 | C140 | C111 | C117 | -162.30(18) |
| C4 | C54 | C122 | C55 | -156.15(17) |  | C110 | C188 | C135 | C33 | 0.2(3) |
| C4 | C54 | C126 | C162 | 0.4(3) |  | C112 | C50 | C56 | C78 | 161.18(16) |
| C4 | C54 | C126 | C180 | 156.36(17) |  | C112 | C50 | C56 | C2 | 4.1(3) |
| C6 | C1 | C10 | C20 | 160.24(16) |  | C112 | C164 | C61 | S7 | 5.1(2) |
| C6 | C1 | C10 | C78 | 1.9(2) |  | C112 | C164 | C61 | C109 | -163.32(18) |
| C6 | C1 | C46 | C16 | -0.9(2) |  | C112 | C15 | C127 | C2 | 0.2(3) |
| C6 | C1 | C46 | C80 | -163.51(15) |  | C114 | C66 | C68 | C62 | -162.67(16) |
| C6 | C24 | C82 | C108 | -162.42(16) |  | C114 | C66 | C68 | C192 | -3.4(2) |
| C6 | C24 | C82 | C164 | -3.7(2) |  | C114 | C66 | C150 | C26 | -1.7(2) |
| C6 | C50 | C56 | C78 | 1.6(3) |  | C114 | C66 | C150 | C38 | 160.98(16) |
| C6 | C50 | C56 | C2 | -155.50(17) |  | C114 | C106 | C152 | C184 | 161.14(17) |
| C6 | C50 | C112 | C164 | -3.3(3) |  | C114 | C106 | C152 | C115 | -6.9(2) |
| C6 | C50 | C112 | C15 | 156.82(17) |  | C114 | C53 | C115 | C152 | -9.29(19) |
| C8 | C16 | C36 | C70 | -162.57(16) |  | C114 | C53 | C115 | C129 | 109.97(18) |
| C8 | C16 | C36 | C156 | -4.3(2) |  | C114 | C53 | C115 | C9 | -128.51(17) |
| C8 | C16 | C46 | C1 | 0.9(2) |  | C116 | C84 | C162 | C126 | 160.14(17) |
| C8 | C16 | C46 | C80 | 163.61(15) |  | C116 | C84 | C162 | C174 | 2.8(3) |
| C8 | C24 | C82 | C108 | 0.5(2) |  | C116 | C128 | C206 | S8 | 5.8(2) |
| C8 | C24 | C82 | C164 | 159.28(16) |  | C116 | C128 | C206 | C184 | -162.16(18) |
| C8 | C90 | C96 | C148 | 155.14(17) |  | C116 | C158 | C160 | C174 | -2.0(3) |
| C8 | C90 | C96 | C156 | -5.5(3) |  | C118 | C52 | C76 | C34 | -162.05(16) |
| C10 | C1 | C6 | C24 | -162.55(15) |  | C118 | C52 | C76 | C44 | 0.6(2) |
| C10 | C1 | C6 | C50 | 0.0(2) |  | C118 | C52 | C104 | C142 | 163.56(16) |
| C10 | C1 | C46 | C16 | 161.72(15) |  | C118 | C52 | C104 | C170 | 3.5(3) |
| C10 | C1 | C46 | C80 | -0.9(2) |  | C118 | C138 | C33 | C135 | -161.76(19) |
| C10 | C20 | C86 | S1 | 165.69(13) |  | C118 | C138 | C33 | C37 | 6.9(2) |
| C10 | C20 | C86 | C120 | -1.5(3) |  | C118 | C123 | C139 | C170 | -1.5(3) |
| C10 | C78 | C27 | C120 | 3.1(3) |  | C118 | C123 | C37 | C33 | 9.9(2) |
| C10 | C78 | C27 | C133 | -165.43(16) |  | C118 | C123 | C37 | C161 | 129.35(18) |
| C12 | C40 | C88 | C94 | -162.31(16) |  | C118 | C123 | C37 | C171 | -107.5(2) |
| C12 | C40 | C88 | C100 | -4.3(2) |  | C120 | C27 | C133 | C2 | -157.20(19) |
| C12 | C42 | C44 | C48 | -162.87(16) |  | C120 | C27 | C133 | C151 | -39.0(3) |
| C12 | C42 | C44 | C76 | 0.1(2) |  | C120 | C27 | C133 | C153 | 85.6(3) |
| C12 | C42 | C168 | C140 | 160.40(16) |  | C122 | C54 | C126 | C162 | -159.26(16) |
| C12 | C42 | C168 | C172 | 2.1(3) |  | C122 | C54 | C126 | C180 | -3.3(3) |
| C12 | C134 | C136 | C144 | -156.25(18) |  | C122 | C72 | C208 | S5 | -4.3(2) |
| C12 | C134 | C136 | C172 | 2.1(3) |  | C122 | C72 | C208 | C119 | 164.98(17) |
| C12 | C134 | C154 | C100 | -3.5(3) |  | C122 | C55 | C29 | C180 | 2.2(3) |
| C12 | C134 | C154 | C198 | 155.80(17) |  | C124 | C70 | C92 | C80 | -158.27(16) |
| C14 | C20 | C86 | S1 | 5.40(19) |  | C124 | C70 | C92 | C194 | 0.7(2) |
| C14 | C20 | C86 | C120 | -161.79(16) |  | C124 | C63 | C69 | C71 | -53.8(2) |
| C14 | C74 | C146 | C194 | -1.8(3) |  | C124 | C63 | C19 | C167 | 56.6(3) |
| C14 | C80 | C92 | C70 | 159.10(16) |  | C124 | C63 | C19A | C215 | -62(4) |
| C14 | C80 | C92 | C194 | 3.3(3) |  | C126 | C54 | C122 | C72 | 163.00(16) |
| C16 | C8 | C24 | C6 | -0.7(2) |  | C126 | C54 | C122 | C55 | 3.4(3) |
| C16 | C8 | C24 | C82 | -163.35(15) |  | C126 | C162 | C174 | C160 | -161.47(17) |
| C16 | C8 | C90 | C60 | 163.00(16) |  | C126 | C162 | C174 | C7 | 6.7(2) |
| C16 | C8 | C90 | C96 | 5.0(2) |  | C126 | C180 | C7 | C174 | 10.45(19) |
| C16 | C36 | C70 | C92 | -1.0(3) |  | C126 | C180 | C7 | C17 | 129.85(17) |
| C16 | C36 | C70 | C124 | 155.37(17) |  | C126 | C180 | C7 | C77 | -108.50(18) |
| C16 | C36 | C156 | C96 | 4.0(3) |  | C126 | C180 | C29 | C55 | -1.9(3) |
| C16 | C36 | C156 | C182 | -155.06(17) |  | C128 | C98 | C106 | C114 | -161.00(17) |
| C16 | C46 | C80 | C14 | -159.23(16) |  | C128 | C98 | C106 | C152 | -5.2(3) |
| C16 | C46 | C80 | C92 | -1.4(2) |  | C128 | C116 | C158 | S8 | -4.1(2) |
| C18 | C4 | C32 | C30 | -162.27(15) |  | C128 | C116 | C158 | C160 | 163.74(18) |
| C18 | C4 | C32 | C38 | 0.6(2) |  | C130 | S4 | C198 | C154 | 5.37(15) |
| C18 | C4 | C54 | C122 | 158.74(16) |  | C130 | S4 | C198 | C13 | -160.82(19) |
| C18 | C4 | C54 | C126 | 0.2(2) |  | C130 | C100 | C154 | C134 | 160.89(17) |
| C18 | C26 | C98 | C106 | -161.97(16) |  | C130 | C100 | C154 | C198 | 0.2(2) |
| C18 | C26 | C98 | C128 | -3.1(2) |  | C130 | C178 | C186 | C94 | -1.2(3) |
| C18 | C26 | C150 | C38 | 0.7(2) |  | C130 | C178 | C186 | C57 | 165.20(19) |
| C18 | C26 | C150 | C66 | 163.12(15) |  | C132 | S9 | C192 | C68 | 6.69(14) |
| C18 | C84 | C116 | C128 | -3.7(3) |  | C132 | S9 | C192 | C190 | -159.78(18) |
| C18 | C84 | C116 | C158 | 156.21(17) |  | C132 | C62 | C68 | C66 | 160.76(17) |
| C18 | C84 | C162 | C126 | 0.8(3) |  | C132 | C62 | C68 | C192 | 0.1(2) |
| C18 | C84 | C162 | C174 | -156.53(18) |  | C132 | C200 | C121 | C58 | -0.3(3) |
| C20 | C10 | C78 | C56 | -161.58(16) |  | C132 | C200 | C121 | C155 | 165.84(19) |
| C20 | C10 | C78 | C27 | -4.6(2) |  | C134 | C12 | C40 | C34 | -161.41(16) |
| C20 | C14 | C74 | S1 | -4.73(18) |  | C134 | C12 | C40 | C88 | 0.3(2) |
| C20 | C14 | C74 | C146 | 162.31(16) |  | C134 | C12 | C42 | C44 | 161.47(15) |
| C20 | C14 | C80 | C46 | -2.8(2) |  | C134 | C12 | C42 | C168 | -1.3(2) |
| C20 | C14 | C80 | C92 | -161.79(16) |  | C134 | C136 | C144 | C13 | -2.1(3) |
| C20 | C86 | C120 | C27 | -0.2(3) |  | C134 | C136 | C144 | C31 | 167.34(18) |
| C22 | C38 | C150 | C26 | 162.99(15) |  | C134 | C136 | C172 | C168 | -1.3(3) |
| C22 | C38 | C150 | C66 | 0.5(2) |  | C134 | C136 | C172 | C113 | -161.47(17) |
| C22 | C58 | C121 | C200 | 1.6(3) |  | C134 | C154 | C198 | S4 | -164.48(15) |
| C22 | C58 | C121 | C155 | -167.87(17) |  | C134 | C154 | C198 | C13 | 3.0(3) |
| C22 | C62 | C68 | C66 | 0.6(3) |  | C136 | C134 | C154 | C100 | -162.81(17) |
| C22 | C62 | C68 | C192 | -160.06(16) |  | C136 | C134 | C154 | C198 | -3.5(3) |
| C22 | C62 | C132 | S9 | 164.72(14) |  | C136 | C144 | C13 | C198 | 1.2(3) |
| C22 | C62 | C132 | C200 | -2.5(3) |  | C136 | C144 | C31 | C113 | -10.3(2) |
| C24 | C6 | C50 | C56 | 160.92(16) |  | C136 | C144 | C31 | C67 | 108.71(18) |
| C24 | C6 | C50 | C112 | 2.5(2) |  | C136 | C144 | C31 | C35 | -130.43(18) |
| C24 | C8 | C16 | C36 | 161.96(15) |  | C136 | C172 | C113 | C117 | 161.58(17) |
| C24 | C8 | C16 | C46 | -0.1(2) |  | C136 | C172 | C113 | C31 | -6.6(2) |
| C24 | C8 | C90 | C60 | 0.7(2) |  | C138 | C48 | C110 | C140 | -162.24(17) |
| C24 | C8 | C90 | C96 | -157.37(16) |  | C138 | C48 | C110 | C188 | -2.9(3) |
| C24 | C82 | C108 | C60 | 1.0(3) |  | C138 | C118 | C123 | C139 | 162.63(18) |
| C24 | C82 | C108 | C107 | 157.22(17) |  | C138 | C118 | C123 | C37 | -6.6(2) |
| C24 | C82 | C164 | C112 | 2.9(3) |  | C138 | C33 | C135 | C188 | 0.8(3) |
| C24 | C82 | C164 | C61 | -156.82(17) |  | C138 | C33 | C37 | C123 | -10.1(2) |
| C26 | C18 | C84 | C116 | 3.8(2) |  | C138 | C33 | C37 | C161 | -129.49(18) |
| C26 | C18 | C84 | C162 | 162.09(16) |  | C138 | C33 | C37 | C171 | 104.6(2) |
| C26 | C98 | C106 | C114 | -1.1(3) |  | C140 | C110 | C188 | S2 | -5.5(2) |
| C26 | C98 | C106 | C152 | 154.68(18) |  | C140 | C110 | C188 | C135 | 161.30(19) |
| C26 | C98 | C128 | C116 | 3.3(3) |  | C140 | C168 | C172 | C136 | -160.35(17) |
| C26 | C98 | C128 | C206 | -155.24(17) |  | C140 | C168 | C172 | C113 | -3.2(3) |
| C28 | C30 | C32 | C4 | 162.93(16) |  | C140 | C111 | C117 | C113 | 1.4(3) |
| C28 | C30 | C32 | C38 | -0.2(2) |  | C142 | C64 | C102 | C94 | 159.02(16) |
| C28 | C30 | C72 | C122 | -164.81(16) |  | C142 | C64 | C102 | C176 | 2.7(3) |
| C28 | C30 | C72 | C208 | -4.1(3) |  | C142 | C104 | C170 | S3 | 5.5(2) |
| C28 | C58 | C121 | C200 | 160.63(17) |  | C142 | C104 | C170 | C139 | -163.31(18) |
| C28 | C58 | C121 | C155 | -8.8(2) |  | C144 | C136 | C172 | C168 | 159.84(17) |
| C28 | C125 | C155 | C121 | -13.41(19) |  | C144 | C136 | C172 | C113 | -0.3(2) |
| C28 | C125 | C155 | C159 | 101.47(18) |  | C144 | C31 | C67 | C131 | -58.4(2) |
| C28 | C125 | C155 | C41 | -134.86(17) |  | C144 | C31 | C35 | C147 | 61.7(2) |
| C30 | C28 | C58 | C22 | -0.2(3) |  | C146 | C194 | C63 | C124 | 154.7(2) |
| C30 | C28 | C58 | C121 | -160.30(16) |  | C146 | C194 | C63 | C69 | 35.0(3) |
| C30 | C28 | C125 | C119 | -2.6(3) |  | C146 | C194 | C63 | C19 | -87.1(3) |
| C30 | C28 | C125 | C155 | 168.09(17) |  | C146 | C194 | C63 | C19A | -89.6(19) |
| C30 | C32 | C38 | C22 | -0.7(2) |  | C148 | S6 | C182 | C156 | -5.27(14) |
| C30 | C32 | C38 | C150 | 161.89(15) |  | C148 | S6 | C182 | C202 | 160.97(19) |
| C30 | C72 | C122 | C54 | 1.0(3) |  | C148 | C96 | C156 | C36 | -161.05(17) |
| C30 | C72 | C122 | C55 | 162.03(16) |  | C148 | C96 | C156 | C182 | -0.5(2) |
| C30 | C72 | C208 | S5 | -165.96(14) |  | C150 | C26 | C98 | C106 | 0.9(2) |
| C30 | C72 | C208 | C119 | 3.4(3) |  | C150 | C26 | C98 | C128 | 159.74(16) |
| C32 | C4 | C18 | C26 | 0.4(2) |  | C150 | C66 | C68 | C62 | -3.5(3) |
| C32 | C4 | C18 | C84 | 162.39(15) |  | C150 | C66 | C68 | C192 | 155.72(17) |
| C32 | C4 | C54 | C122 | -4.1(2) |  | C150 | C66 | C114 | C106 | 1.5(3) |
| C32 | C4 | C54 | C126 | -162.65(16) |  | C150 | C66 | C114 | C53 | -155.09(17) |
| C32 | C30 | C72 | C122 | -4.7(3) |  | C152 | C106 | C114 | C66 | -158.94(17) |
| C32 | C30 | C72 | C208 | 156.03(17) |  | C152 | C106 | C114 | C53 | 0.7(2) |
| C32 | C38 | C150 | C26 | 0.3(2) |  | C152 | C184 | C206 | S8 | -164.85(15) |
| C32 | C38 | C150 | C66 | -162.20(15) |  | C152 | C184 | C206 | C128 | 0.3(3) |
| C34 | C40 | C88 | C94 | -0.3(2) |  | C152 | C115 | C129 | C73 | 53.4(2) |
| C34 | C40 | C88 | C100 | 157.72(16) |  | C152 | C115 | C9 | C157 | -59.1(2) |
| C34 | C64 | C102 | C94 | -0.9(3) |  | C154 | C100 | C130 | S4 | 3.9(2) |
| C34 | C64 | C102 | C176 | -157.28(17) |  | C154 | C100 | C130 | C178 | -164.16(17) |
| C34 | C64 | C142 | C104 | -3.5(3) |  | C154 | C134 | C136 | C144 | 3.1(3) |
| C34 | C64 | C142 | C105 | 156.72(17) |  | C154 | C134 | C136 | C172 | 161.43(17) |
| C36 | C16 | C46 | C1 | -161.20(15) |  | C154 | C198 | C13 | C144 | -1.7(3) |
| C36 | C16 | C46 | C80 | 1.5(2) |  | C156 | C36 | C70 | C92 | -160.44(16) |
| C36 | C70 | C92 | C80 | 1.1(3) |  | C156 | C36 | C70 | C124 | -4.1(3) |
| C36 | C70 | C92 | C194 | 160.05(16) |  | C156 | C96 | C148 | S6 | -3.5(2) |
| C36 | C70 | C124 | C202 | 2.5(3) |  | C156 | C96 | C148 | C196 | 165.48(17) |
| C36 | C70 | C124 | C63 | -166.26(17) |  | C156 | C182 | C202 | C124 | 1.2(3) |
| C36 | C156 | C182 | S6 | 164.35(14) |  | C158 | S8 | C206 | C128 | -6.81(15) |
| C36 | C156 | C182 | C202 | -3.2(3) |  | C158 | S8 | C206 | C184 | 159.85(19) |
| C38 | C22 | C58 | C28 | -0.8(3) |  | C158 | C116 | C128 | C98 | -161.11(17) |
| C38 | C22 | C58 | C121 | 156.40(17) |  | C158 | C116 | C128 | C206 | -1.1(2) |
| C38 | C22 | C62 | C68 | 2.9(3) |  | C158 | C160 | C174 | C162 | 1.7(3) |
| C38 | C22 | C62 | C132 | -155.89(17) |  | C158 | C160 | C174 | C7 | -163.04(19) |
| C40 | C12 | C42 | C44 | -0.9(2) |  | C160 | C174 | C7 | C180 | 155.7(2) |
| C40 | C12 | C42 | C168 | -163.63(16) |  | C160 | C174 | C7 | C17 | 36.7(3) |
| C40 | C12 | C134 | C136 | 161.72(16) |  | C160 | C174 | C7 | C77 | -84.8(3) |
| C40 | C12 | C134 | C154 | 3.5(3) |  | C162 | C84 | C116 | C128 | -163.00(17) |
| C40 | C34 | C64 | C102 | 0.1(2) |  | C162 | C84 | C116 | C158 | -3.1(3) |
| C40 | C34 | C64 | C142 | -158.68(16) |  | C162 | C126 | C180 | C7 | -7.2(2) |
| C40 | C34 | C76 | C44 | -0.7(2) |  | C162 | C126 | C180 | C29 | 160.52(17) |
| C40 | C34 | C76 | C52 | 161.85(15) |  | C162 | C174 | C7 | C180 | -10.24(19) |
| C40 | C88 | C94 | C102 | -0.6(3) |  | C162 | C174 | C7 | C17 | -129.32(17) |
| C40 | C88 | C94 | C186 | 156.37(17) |  | C162 | C174 | C7 | C77 | 109.19(17) |
| C40 | C88 | C100 | C130 | -155.52(17) |  | C164 | C82 | C108 | C60 | -158.83(17) |
| C40 | C88 | C100 | C154 | 4.4(3) |  | C164 | C82 | C108 | C107 | -2.6(3) |
| C42 | C12 | C40 | C34 | 0.8(2) |  | C164 | C112 | C15 | S7 | -5.7(2) |
| C42 | C12 | C40 | C88 | 162.59(15) |  | C164 | C112 | C15 | C127 | 161.85(18) |
| C42 | C12 | C134 | C136 | -0.8(2) |  | C166 | C60 | C90 | C8 | -156.43(17) |
| C42 | C12 | C134 | C154 | -158.94(16) |  | C166 | C60 | C90 | C96 | 2.8(3) |
| C42 | C44 | C48 | C110 | 2.7(3) |  | C166 | C60 | C108 | C82 | 158.29(17) |
| C42 | C44 | C48 | C138 | 161.30(16) |  | C166 | C60 | C108 | C107 | -0.8(2) |
| C42 | C44 | C76 | C34 | 0.6(2) |  | C166 | C59 | C149 | C21 | 55.1(3) |
| C42 | C44 | C76 | C52 | -161.80(16) |  | C166 | C59 | C39 | C175 | -52.1(3) |
| C42 | C168 | C172 | C136 | -0.8(3) |  | C168 | C42 | C44 | C48 | -0.1(2) |
| C42 | C168 | C172 | C113 | 156.27(18) |  | C168 | C42 | C44 | C76 | 162.91(16) |
| C44 | C42 | C168 | C140 | -2.5(2) |  | C168 | C140 | C111 | S2 | 165.78(15) |
| C44 | C42 | C168 | C172 | -160.81(17) |  | C168 | C140 | C111 | C117 | -2.1(3) |
| C44 | C48 | C110 | C140 | -2.6(3) |  | C168 | C172 | C113 | C117 | 2.7(3) |
| C44 | C48 | C110 | C188 | 156.70(18) |  | C168 | C172 | C113 | C31 | -165.52(17) |
| C44 | C48 | C138 | C118 | 0.9(3) |  | C170 | S3 | C105 | C142 | 6.64(14) |
| C44 | C48 | C138 | C33 | -155.62(18) |  | C170 | S3 | C105 | C204 | -160.40(19) |
| C46 | C1 | C6 | C24 | 0.1(2) |  | C170 | C104 | C142 | C64 | 161.21(17) |
| C46 | C1 | C6 | C50 | 162.60(15) |  | C170 | C104 | C142 | C105 | -0.5(2) |
| C46 | C1 | C10 | C20 | -2.7(2) |  | C172 | C136 | C144 | C13 | -162.23(18) |
| C46 | C1 | C10 | C78 | -161.01(15) |  | C172 | C136 | C144 | C31 | 7.2(2) |
| C46 | C16 | C36 | C70 | -0.4(2) |  | C172 | C113 | C117 | C111 | -1.5(3) |
| C46 | C16 | C36 | C156 | 157.90(16) |  | C172 | C113 | C31 | C144 | 10.1(2) |
| C46 | C80 | C92 | C70 | 0.2(3) |  | C172 | C113 | C31 | C67 | -107.37(18) |
| C46 | C80 | C92 | C194 | -155.62(17) |  | C172 | C113 | C31 | C35 | 130.08(17) |
| C48 | C44 | C76 | C34 | 163.62(16) |  | C174 | C7 | C17 | C79 | 54.1(2) |
| C48 | C44 | C76 | C52 | 1.2(2) |  | C174 | C7 | C77 | C179 | -62.5(3) |
| C48 | C110 | C140 | C168 | 0.0(3) |  | C176 | C204 | C105 | S3 | 164.37(15) |
| C48 | C110 | C140 | C111 | 160.57(17) |  | C176 | C204 | C105 | C142 | -1.3(3) |
| C48 | C110 | C188 | S2 | -165.89(15) |  | C176 | C57 | C65 | C81 | 55.8(2) |
| C48 | C110 | C188 | C135 | 0.9(3) |  | C176 | C57 | C141 | C75 | -60.8(2) |
| C48 | C138 | C33 | C135 | -3.1(3) |  | C178 | C186 | C57 | C176 | -155.1(2) |
| C48 | C138 | C33 | C37 | 165.52(18) |  | C178 | C186 | C57 | C65 | -34.4(3) |
| C50 | C6 | C24 | C8 | -161.85(15) |  | C178 | C186 | C57 | C141 | 86.8(2) |
| C50 | C6 | C24 | C82 | 1.0(2) |  | C180 | C126 | C162 | C84 | -159.82(17) |
| C50 | C56 | C78 | C10 | 0.3(3) |  | C180 | C126 | C162 | C174 | 0.4(2) |
| C50 | C56 | C78 | C27 | -159.57(16) |  | C180 | C7 | C17 | C79 | -59.2(2) |
| C50 | C56 | C2 | C127 | -3.3(3) |  | C180 | C7 | C77 | C179 | 50.7(3) |
| C50 | C56 | C2 | C133 | 164.62(16) |  | C182 | S6 | C148 | C96 | 4.99(15) |
| C50 | C112 | C164 | C82 | 0.6(3) |  | C182 | S6 | C148 | C196 | -162.70(19) |
| C50 | C112 | C164 | C61 | 161.75(17) |  | C184 | C152 | C115 | C53 | -156.3(2) |
| C50 | C112 | C15 | S7 | -166.69(14) |  | C184 | C152 | C115 | C129 | 84.4(2) |
| C50 | C112 | C15 | C127 | 0.8(3) |  | C184 | C152 | C115 | C9 | -36.8(3) |
| C52 | C104 | C142 | C64 | -0.3(3) |  | C186 | C94 | C102 | C64 | -158.56(17) |
| C52 | C104 | C142 | C105 | -161.96(17) |  | C186 | C94 | C102 | C176 | 0.6(2) |
| C52 | C104 | C170 | S3 | 166.46(14) |  | C186 | C57 | C65 | C81 | -58.7(2) |
| C52 | C104 | C170 | C139 | -2.3(3) |  | C186 | C57 | C141 | C75 | 51.5(2) |
| C52 | C118 | C138 | C48 | 0.9(3) |  | C188 | S2 | C111 | C140 | -7.35(15) |
| C52 | C118 | C138 | C33 | 160.42(17) |  | C188 | S2 | C111 | C117 | 159.23(19) |
| C52 | C118 | C123 | C139 | 3.1(3) |  | C188 | C110 | C140 | C168 | -160.62(18) |
| C52 | C118 | C123 | C37 | -166.18(17) |  | C188 | C110 | C140 | C111 | -0.1(2) |
| C54 | C4 | C18 | C26 | -162.32(15) |  | C190 | C53 | C115 | C152 | 156.5(2) |
| C54 | C4 | C18 | C84 | -0.3(2) |  | C190 | C53 | C115 | C129 | -84.3(3) |
| C54 | C4 | C32 | C30 | 0.5(2) |  | C190 | C53 | C115 | C9 | 37.2(3) |
| C54 | C4 | C32 | C38 | 163.34(15) |  | C192 | S9 | C132 | C62 | -6.63(14) |
| C54 | C122 | C55 | S5 | 164.95(14) |  | C192 | S9 | C132 | C200 | 159.37(18) |
| C54 | C122 | C55 | C29 | -3.0(3) |  | C192 | C190 | C53 | C114 | 1.9(3) |
| C54 | C126 | C162 | C84 | -0.9(3) |  | C192 | C190 | C53 | C115 | -162.53(19) |
| C54 | C126 | C162 | C174 | 159.32(17) |  | C194 | C63 | C69 | C71 | 59.5(2) |
| C54 | C126 | C180 | C7 | -165.11(17) |  | C194 | C63 | C19 | C167 | -56.3(4) |
| C54 | C126 | C180 | C29 | 2.7(3) |  | C194 | C63 | C19A | C215 | -169(3) |
| C56 | C50 | C112 | C164 | -162.91(17) |  | C196 | C166 | C59 | C107 | 155.1(2) |
| C56 | C50 | C112 | C15 | -2.8(3) |  | C196 | C166 | C59 | C149 | 34.8(3) |
| C56 | C78 | C27 | C120 | 162.09(17) |  | C196 | C166 | C59 | C39 | -86.9(3) |
| C56 | C78 | C27 | C133 | -6.5(2) |  | C198 | S4 | C130 | C100 | -5.27(15) |
| C56 | C2 | C133 | C27 | -8.99(19) |  | C198 | S4 | C130 | C178 | 161.46(18) |
| C56 | C2 | C133 | C151 | -128.25(17) |  | C200 | C121 | C155 | C125 | -154.1(2) |
| C56 | C2 | C133 | C153 | 109.48(18) |  | C200 | C121 | C155 | C159 | 91.9(2) |
| C58 | C22 | C38 | C32 | 1.3(2) |  | C200 | C121 | C155 | C41 | -31.2(3) |
| C58 | C22 | C38 | C150 | -161.57(16) |  | C202 | C124 | C63 | C194 | -155.5(2) |
| C58 | C22 | C62 | C68 | 162.18(16) |  | C202 | C124 | C63 | C69 | -35.9(3) |
| C58 | C22 | C62 | C132 | 3.4(2) |  | C202 | C124 | C63 | C19 | 84.1(3) |
| C58 | C28 | C30 | C32 | 0.7(3) |  | C202 | C124 | C63 | C19A | 98.9(14) |
| C58 | C28 | C30 | C72 | 160.84(16) |  | C204 | C176 | C57 | C186 | 153.5(2) |
| C58 | C28 | C125 | C119 | -161.49(17) |  | C204 | C176 | C57 | C65 | 32.7(3) |
| C58 | C28 | C125 | C155 | 9.2(2) |  | C204 | C176 | C57 | C141 | -89.0(3) |
| C58 | C121 | C155 | C125 | 13.29(19) |  | C206 | S8 | C158 | C116 | 6.21(15) |
| C58 | C121 | C155 | C159 | -100.76(18) |  | C206 | S8 | C158 | C160 | -160.30(19) |
| C58 | C121 | C155 | C41 | 136.12(18) |  | C208 | S5 | C55 | C122 | -5.72(14) |
| C60 | C90 | C96 | C148 | -3.9(3) |  | C208 | S5 | C55 | C29 | 160.80(19) |
| C60 | C90 | C96 | C156 | -164.50(17) |  | C208 | C72 | C122 | C54 | -160.99(16) |
| C60 | C108 | C107 | C109 | 160.22(17) |  | C208 | C72 | C122 | C55 | 0.0(2) |
| C60 | C108 | C107 | C59 | -7.9(2) |  | C208 | C119 | C125 | C28 | 1.5(3) |
| C60 | C166 | C196 | C148 | 1.3(3) |  | C208 | C119 | C125 | C155 | -166.22(19) |
| C60 | C166 | C59 | C107 | -12.95(19) |  | C13 | C144 | C31 | C113 | 157.1(2) |
| C60 | C166 | C59 | C149 | -133.34(17) |  | C13 | C144 | C31 | C67 | -83.9(3) |
| C60 | C166 | C59 | C39 | 105.01(18) |  | C13 | C144 | C31 | C35 | 37.0(3) |
| C62 | C22 | C38 | C32 | 159.44(16) |  | C105 | S3 | C170 | C104 | -6.89(15) |
| C62 | C22 | C38 | C150 | -3.4(2) |  | C105 | S3 | C170 | C139 | 160.71(19) |
| C62 | C22 | C58 | C28 | -160.22(16) |  | C53 | C190 | C192 | S9 | 163.49(15) |
| C62 | C22 | C58 | C121 | -3.0(3) |  | C53 | C190 | C192 | C68 | -1.4(3) |
| C62 | C68 | C192 | S9 | -5.12(19) |  | C53 | C115 | C129 | C73 | -59.8(2) |
| C62 | C68 | C192 | C190 | 162.60(17) |  | C53 | C115 | C9 | C157 | 54.1(2) |
| C62 | C132 | C200 | C121 | 0.8(3) |  | C107 | C109 | C61 | S7 | -163.92(16) |
| C64 | C34 | C40 | C12 | 162.21(15) |  | C107 | C109 | C61 | C164 | 1.7(3) |
| C64 | C34 | C40 | C88 | 0.6(2) |  | C107 | C59 | C149 | C21 | -59.4(2) |
| C64 | C34 | C76 | C44 | -163.12(15) |  | C107 | C59 | C39 | C175 | 60.0(3) |
| C64 | C34 | C76 | C52 | -0.6(2) |  | C27 | C133 | C151 | C85 | -52.0(2) |
| C64 | C102 | C176 | C204 | -1.8(3) |  | C27 | C133 | C153 | C143 | -53.2(2) |
| C64 | C102 | C176 | C57 | 166.06(17) |  | C109 | C107 | C59 | C166 | -153.4(2) |
| C64 | C142 | C105 | S3 | -166.03(14) |  | C109 | C107 | C59 | C149 | -32.3(3) |
| C64 | C142 | C105 | C204 | 2.4(3) |  | C109 | C107 | C59 | C39 | 89.7(3) |
| C66 | C68 | C192 | S9 | -165.44(14) |  | C55 | S5 | C208 | C72 | 5.71(14) |
| C66 | C68 | C192 | C190 | 2.3(3) |  | C55 | S5 | C208 | C119 | -162.33(18) |
| C66 | C114 | C53 | C190 | -3.5(3) |  | C111 | S2 | C188 | C110 | 7.32(16) |
| C66 | C114 | C53 | C115 | 164.38(17) |  | C111 | S2 | C188 | C135 | -158.1(2) |
| C68 | C62 | C132 | S9 | 4.9(2) |  | C111 | C140 | C168 | C42 | -156.57(18) |
| C68 | C62 | C132 | C200 | -162.26(17) |  | C111 | C140 | C168 | C172 | 2.9(3) |
| C68 | C66 | C114 | C106 | 160.73(16) |  | C7 | C180 | C29 | C55 | 162.27(19) |
| C68 | C66 | C114 | C53 | 4.2(3) |  | C7 | C17 | C79 | C45 | 169.7(2) |
| C68 | C66 | C150 | C26 | -159.71(16) |  | C7 | C77 | C179 | C91 | 175.5(2) |
| C68 | C66 | C150 | C38 | 2.9(2) |  | C113 | C31 | C67 | C131 | 54.0(2) |
| C70 | C36 | C156 | C96 | 163.42(16) |  | C113 | C31 | C35 | C147 | -52.0(2) |
| C70 | C36 | C156 | C182 | 4.4(3) |  | C57 | C176 | C204 | C105 | -163.27(19) |
| C70 | C92 | C194 | C146 | -160.84(17) |  | C57 | C65 | C81 | C93 | 177.7(2) |
| C70 | C92 | C194 | C63 | 6.9(2) |  | C57 | C141 | C75 | C165 | -179.90(19) |
| C70 | C124 | C202 | C182 | -0.8(3) |  | C115 | C152 | C184 | C206 | 164.01(19) |
| C70 | C124 | C63 | C194 | 11.29(19) |  | C115 | C129 | C73 | C5 | -179.78(17) |
| C70 | C124 | C63 | C69 | 130.91(17) |  | C115 | C9 | C157 | C187 | 171.0(4) |
| C70 | C124 | C63 | C19 | -109.1(2) |  | C115 | C9 | C157 | C216 | 176.7(9) |
| C70 | C124 | C63 | C19A | -94.3(14) |  | C29 | C180 | C7 | C174 | -155.1(2) |
| C72 | C30 | C32 | C4 | 3.9(2) |  | C29 | C180 | C7 | C17 | -35.7(3) |
| C72 | C30 | C32 | C38 | -159.20(16) |  | C29 | C180 | C7 | C77 | 85.9(3) |
| C72 | C122 | C55 | S5 | 4.32(19) |  | C117 | C113 | C31 | C144 | -155.8(2) |
| C72 | C122 | C55 | C29 | -163.68(17) |  | C117 | C113 | C31 | C67 | 86.7(3) |
| C72 | C208 | C119 | C125 | -1.9(3) |  | C117 | C113 | C31 | C35 | -35.8(3) |
| C74 | S1 | C86 | C20 | -6.81(14) |  | C59 | C166 | C196 | C148 | -165.66(19) |
| C74 | S1 | C86 | C120 | 159.08(17) |  | C59 | C107 | C109 | C61 | 163.13(19) |
| C74 | C14 | C20 | C10 | -161.12(16) |  | C59 | C149 | C21 | C25 | 163.6(4) |
| C74 | C14 | C20 | C86 | -0.5(2) |  | C59 | C149 | C21 | C25A | -167.8(5) |
| C74 | C14 | C80 | C46 | 156.03(16) |  | C59 | C39 | C175 | C183 | 179.7(2) |
| C74 | C14 | C80 | C92 | -2.9(2) |  | C119 | C125 | C155 | C121 | 155.3(2) |
| C74 | C146 | C194 | C92 | 2.0(3) |  | C119 | C125 | C155 | C159 | -89.8(3) |
| C74 | C146 | C194 | C63 | -162.26(18) |  | C119 | C125 | C155 | C41 | 33.9(3) |
| C76 | C34 | C40 | C12 | -0.1(2) |  | C15 | S7 | C61 | C164 | -7.01(15) |
| C76 | C34 | C40 | C88 | -161.72(15) |  | C15 | S7 | C61 | C109 | 160.00(19) |
| C76 | C34 | C64 | C102 | 162.64(16) |  | C15 | C112 | C164 | C82 | -160.79(17) |
| C76 | C34 | C64 | C142 | 3.9(2) |  | C15 | C112 | C164 | C61 | 0.4(2) |
| C76 | C44 | C48 | C110 | -160.61(17) |  | C15 | C127 | C2 | C56 | 0.9(3) |
| C76 | C44 | C48 | C138 | -2.0(2) |  | C15 | C127 | C2 | C133 | -163.54(19) |
| C76 | C52 | C104 | C142 | 3.6(3) |  | C121 | C155 | C159 | C83 | -66.8(2) |
| C76 | C52 | C104 | C170 | -156.50(17) |  | C121 | C155 | C41 | C89 | -65.8(3) |
| C76 | C52 | C118 | C138 | -1.6(3) |  | C61 | S7 | C15 | C112 | 7.19(15) |
| C76 | C52 | C118 | C123 | 156.28(18) |  | C61 | S7 | C15 | C127 | -159.09(18) |
| C78 | C10 | C20 | C14 | 162.97(15) |  | C123 | C118 | C138 | C48 | -159.73(18) |
| C78 | C10 | C20 | C86 | 3.7(2) |  | C123 | C118 | C138 | C33 | -0.2(2) |
| C78 | C56 | C2 | C127 | -162.10(17) |  | C123 | C37 | C161 | C11 | -56.5(3) |
| C78 | C56 | C2 | C133 | 5.8(2) |  | C123 | C37 | C171 | C185 | 173.9(2) |
| C78 | C27 | C133 | C2 | 9.24(19) |  | C31 | C144 | C13 | C198 | -165.1(2) |
| C78 | C27 | C133 | C151 | 127.47(17) |  | C31 | C113 | C117 | C111 | 163.1(2) |
| C78 | C27 | C133 | C153 | -108.01(18) |  | C31 | C35 | C147 | C173 | -173.0(2) |
| C80 | C14 | C20 | C10 | -0.8(2) |  | C125 | C28 | C30 | C32 | -156.43(17) |
| C80 | C14 | C20 | C86 | 159.87(16) |  | C125 | C28 | C30 | C72 | 3.7(3) |
| C80 | C14 | C74 | S1 | -164.70(13) |  | C125 | C28 | C58 | C22 | 159.88(16) |
| C80 | C14 | C74 | C146 | 2.3(3) |  | C125 | C28 | C58 | C121 | -0.3(2) |
| C80 | C92 | C194 | C146 | -3.0(3) |  | C125 | C155 | C159 | C83 | -176.7(2) |
| C80 | C92 | C194 | C63 | 164.76(17) |  | C125 | C155 | C41 | C89 | 49.6(3) |
| C82 | C108 | C107 | C109 | 2.0(3) |  | C63 | C124 | C202 | C182 | 164.75(19) |
| C82 | C108 | C107 | C59 | -166.11(17) |  | C63 | C69 | C71 | C43 | 173.13(19) |
| C82 | C164 | C61 | S7 | 165.92(14) |  | C63 | C19 | C167 | C189 | -169.7(2) |
| C82 | C164 | C61 | C109 | -2.5(3) |  | C63 | C19A | C215 | C1B | -179(3) |
| C84 | C18 | C26 | C98 | -0.4(2) |  | C127 | C2 | C133 | C27 | 156.9(2) |
| C84 | C18 | C26 | C150 | -163.05(15) |  | C127 | C2 | C133 | C151 | 37.6(3) |
| C84 | C116 | C128 | C98 | 0.1(3) |  | C127 | C2 | C133 | C153 | -84.7(3) |
| C84 | C116 | C128 | C206 | 160.09(17) |  | C2 | C56 | C78 | C10 | 160.31(16) |
| C84 | C116 | C158 | S8 | -165.03(14) |  | C2 | C56 | C78 | C27 | 0.4(2) |
| C84 | C116 | C158 | C160 | 2.8(3) |  | C2 | C133 | C151 | C85 | 60.8(2) |
| C84 | C162 | C174 | C160 | -2.2(3) |  | C2 | C133 | C153 | C143 | -165.26(18) |
| C84 | C162 | C174 | C7 | 165.93(17) |  | C129 | C115 | C9 | C157 | 177.77(18) |
| C86 | S1 | C74 | C14 | 6.58(13) |  | C129 | C73 | C5 | C177 | -177.65(19) |
| C86 | S1 | C74 | C146 | -159.18(17) |  | C65 | C57 | C141 | C75 | 175.16(18) |
| C86 | C120 | C27 | C78 | -0.6(3) |  | C65 | C81 | C93 | C3 | 174.9(3) |
| C86 | C120 | C27 | C133 | 164.67(18) |  | C33 | C37 | C161 | C11 | 56.9(3) |
| C88 | C94 | C102 | C64 | 1.2(3) |  | C33 | C37 | C171 | C185 | 63.0(3) |
| C88 | C94 | C102 | C176 | 160.41(17) |  | C133 | C151 | C85 | C47 | -167.9(5) |
| C88 | C94 | C186 | C178 | 1.8(3) |  | C133 | C151 | C85 | C47A | -168.8(11) |
| C88 | C94 | C186 | C57 | -167.74(17) |  | C67 | C31 | C35 | C147 | -176.58(19) |
| C88 | C100 | C130 | S4 | 164.82(14) |  | C67 | C131 | C137 | C163 | 172.98(18) |
| C88 | C100 | C130 | C178 | -3.2(3) |  | C135 | C33 | C37 | C123 | 156.4(2) |
| C88 | C100 | C154 | C134 | -0.4(3) |  | C135 | C33 | C37 | C161 | 37.0(3) |
| C88 | C100 | C154 | C198 | -161.11(17) |  | C135 | C33 | C37 | C171 | -88.9(3) |
| C90 | C8 | C16 | C36 | -0.1(2) |  | C17 | C7 | C77 | C179 | 174.1(2) |
| C90 | C8 | C16 | C46 | -162.21(15) |  | C17 | C79 | C45 | C97 | 57.2(3) |
| C90 | C8 | C24 | C6 | 161.34(15) |  | C137 | C131 | C67 | C31 | 172.69(17) |
| C90 | C8 | C24 | C82 | -1.4(2) |  | C69 | C63 | C19 | C167 | 179.2(2) |
| C90 | C60 | C108 | C82 | -1.8(3) |  | C69 | C63 | C19A | C215 | 71(4) |
| C90 | C60 | C108 | C107 | -160.86(17) |  | C69 | C71 | C43 | C95 | -172.8(2) |
| C90 | C60 | C166 | C196 | -1.6(3) |  | C139 | C123 | C37 | C33 | -157.4(2) |
| C90 | C60 | C166 | C59 | 168.42(17) |  | C139 | C123 | C37 | C161 | -37.9(3) |
| C90 | C96 | C148 | S6 | -165.10(14) |  | C139 | C123 | C37 | C171 | 85.3(3) |
| C90 | C96 | C148 | C196 | 3.9(3) |  | C35 | C31 | C67 | C131 | 178.62(17) |
| C90 | C96 | C156 | C36 | 0.9(3) |  | C35 | C147 | C173 | C191 | -172.0(2) |
| C90 | C96 | C156 | C182 | 161.46(17) |  | C141 | C57 | C65 | C81 | 179.07(19) |
| C92 | C70 | C124 | C202 | 160.67(17) |  | C141 | C75 | C165 | C169 | 174.3(2) |
| C92 | C70 | C124 | C63 | -8.1(2) |  | C9 | C115 | C129 | C73 | 176.50(17) |
| C92 | C194 | C63 | C124 | -10.89(19) |  | C9 | C157 | C187 | C209 | 171.8(6) |
| C92 | C194 | C63 | C69 | -130.63(17) |  | C9 | C157 | C216 | C1C | 60(2) |
| C92 | C194 | C63 | C19 | 107.3(3) |  | C145 | C143 | C153 | C133 | 100.8(2) |
| C92 | C194 | C63 | C19A | 104.8(19) |  | C37 | C123 | C139 | C170 | 164.5(2) |
| C94 | C88 | C100 | C130 | 3.5(3) |  | C37 | C33 | C135 | C188 | -164.4(2) |
| C94 | C88 | C100 | C154 | 163.42(17) |  | C37 | C161 | C11 | C199 | -170.5(3) |
| C94 | C102 | C176 | C204 | -160.06(17) |  | C37 | C171 | C185 | C205 | -109.1(3) |
| C94 | C102 | C176 | C57 | 7.8(2) |  | C37 | C171 | C185 | C211 | -144.4(4) |
| C94 | C186 | C57 | C176 | 12.53(19) |  | C149 | C59 | C39 | C175 | -176.2(2) |
| C94 | C186 | C57 | C65 | 133.22(17) |  | C149 | C21 | C25 | C103 | 59.8(6) |
| C94 | C186 | C57 | C141 | -105.58(17) |  | C149 | C21 | C25A | C214 | -63.0(11) |
| C96 | C148 | C196 | C166 | -2.4(3) |  | C151 | C133 | C153 | C143 | 72.3(2) |
| C96 | C156 | C182 | S6 | 4.3(2) |  | C151 | C85 | C47 | C201 | 80.1(6) |
| C96 | C156 | C182 | C202 | -163.23(18) |  | C151 | C85 | C47A | C217 | 170.0(14) |
| C98 | C26 | C150 | C38 | -161.96(15) |  | C19 | C63 | C69 | C71 | -175.2(2) |
| C98 | C26 | C150 | C66 | 0.5(2) |  | C19 | C167 | C189 | C49 | -172.6(3) |
| C98 | C106 | C114 | C66 | -0.1(3) |  | C153 | C133 | C151 | C85 | -177.20(19) |
| C98 | C106 | C114 | C53 | 159.56(17) |  | C153 | C143 | C145 | C87 | -170.9(2) |
| C98 | C106 | C152 | C184 | 3.3(3) |  | C77 | C7 | C17 | C79 | 177.17(19) |
| C98 | C106 | C152 | C115 | -164.73(17) |  | C77 | C179 | C91 | C99 | 174.8(4) |
| C98 | C128 | C206 | S8 | 165.51(14) |  | C155 | C159 | C83 | C23 | 166.7(2) |
| C98 | C128 | C206 | C184 | -2.4(3) |  | C155 | C41 | C89 | C181 | 170.5(2) |
| C100 | C88 | C94 | C102 | -159.80(17) |  | C39 | C59 | C149 | C21 | 177.2(2) |
| C100 | C88 | C94 | C186 | -2.9(3) |  | C39 | C175 | C183 | C193 | -179.8(3) |
| C100 | C130 | C178 | C186 | 1.9(3) |  | C159 | C155 | C41 | C89 | 171.85(19) |
| C100 | C154 | C198 | S4 | -4.2(2) |  | C159 | C83 | C23 | C197 | 176.2(3) |
| C100 | C154 | C198 | C13 | 163.25(18) |  | C161 | C37 | C171 | C185 | -62.9(3) |
| C102 | C64 | C142 | C104 | -163.14(16) |  | C161 | C11 | C199 | C203 | -57.5(6) |
| C102 | C64 | C142 | C105 | -2.9(3) |  | C41 | C155 | C159 | C83 | 57.4(3) |
| C102 | C94 | C186 | C178 | 160.76(17) |  | C41 | C89 | C181 | C195 | 170.6(3) |
| C102 | C94 | C186 | C57 | -8.8(2) |  | C171 | C37 | C161 | C11 | -177.4(2) |
| C102 | C176 | C204 | C105 | 1.0(3) |  | C171 | C185 | C205 | C210 | 174.7(5) |
| C102 | C176 | C57 | C186 | -12.18(19) |  | C171 | C185 | C211 | C1A | -173.8(10) |
| C102 | C176 | C57 | C65 | -132.96(17) |  | C19A | C63 | C69 | C71 | 173.6(14) |
| C102 | C176 | C57 | C141 | 105.29(18) |  | C19A | C215 | C1B | C49A | -72(3) |

Table 7 Hydrogen Atom Coordinates (Å×104) and Isotropic Displacement Parameters (Å2×103) for z.

| Atom | *x* | *y* | *z* | U(eq) |
| --- | --- | --- | --- | --- |
| H120 | 5469.1 | -425.33 | 5790.43 | 33 |
| H146 | 4080.76 | 1676.07 | 4903.21 | 32 |
| H160 | -3046.29 | 2264.13 | 4699.33 | 36 |
| H178 | 451.5 | 2709.08 | 6847.83 | 34 |
| H184 | -3694.29 | 2877.76 | 2536.8 | 34 |
| H190 | -2702.91 | 1704.06 | 1336.69 | 34 |
| H196 | 3644.47 | 2275.13 | 8219.55 | 37 |
| H200 | -1050.39 | -227.43 | 2225.71 | 37 |
| H202 | 3134.9 | 2940.29 | 6081.54 | 35 |
| H204 | 1768.65 | 4734.53 | 7602.2 | 36 |
| H13 | -50.03 | 1919.65 | 4740.59 | 36 |
| H109 | 5076.29 | 352.43 | 9101.07 | 39 |
| H29 | -1484.81 | 415.5 | 5546.7 | 38 |
| H117 | 830.16 | 3164.5 | 3484.9 | 38 |
| H119 | -485.91 | -881.56 | 4264.1 | 38 |
| H127 | 5965.91 | -1074.79 | 7824.89 | 38 |
| H12A | -3249.1 | 2885.11 | 1246.77 | 40 |
| H12B | -3552.9 | 3195.1 | 1557.17 | 40 |
| H65A | 483.71 | 3641.99 | 7343.86 | 42 |
| H65B | 850.15 | 4192.29 | 7567.5 | 42 |
| H13A | 1189.81 | 1405.16 | 4396.17 | 40 |
| H13B | 1372.44 | 1673.37 | 3982.2 | 40 |
| H67A | 219.88 | 1550.18 | 3851.26 | 42 |
| H67B | 438.86 | 1880 | 3487.89 | 42 |
| H135 | 2173.98 | 5322.24 | 4262.56 | 45 |
| H17G | -2479.11 | 797.85 | 5431.59 | 40 |
| H17H | -2909.48 | 1308.65 | 5221.07 | 40 |
| H13C | 751 | 647.49 | 3882.91 | 45 |
| H13D | 853.26 | 929.78 | 3431.66 | 45 |
| H69A | 2871.83 | 2464.69 | 5201.68 | 43 |
| H69B | 3141.67 | 2093.6 | 4897.77 | 43 |
| H139 | 2611.22 | 6106.29 | 6255.79 | 41 |
| H35A | -124.33 | 2679.4 | 3497.87 | 42 |
| H35B | -346.15 | 2383.46 | 3874.32 | 42 |
| H14G | 1621.48 | 3624.61 | 8147.07 | 41 |
| H14H | 1213.75 | 3096.63 | 7940.88 | 41 |
| H71A | 3370.88 | 1380.56 | 5483.8 | 50 |
| H71B | 3175.18 | 1760.39 | 5837.54 | 50 |
| H14A | 6679.11 | -1548.51 | 6405.25 | 43 |
| H14B | 6132.56 | -1169.06 | 6129.44 | 43 |
| H9A | -3948.94 | 2331.21 | 1712.34 | 45 |
| H9B | -3693.18 | 2023.62 | 1364 | 45 |
| H14C | 7274.87 | -788.12 | 6472.56 | 48 |
| H14D | 6755 | -359.29 | 6279.58 | 48 |
| H73A | -2378.22 | 3286.22 | 1760.22 | 45 |
| H73B | -2681.54 | 3595.69 | 2073.4 | 45 |
| H14I | 120.31 | 3488.6 | 3978.48 | 52 |
| H14J | -23.81 | 3190.35 | 4403.09 | 52 |
| H14E | 4137.95 | 840.23 | 9023.52 | 44 |
| H14F | 3739.12 | 1370.4 | 8797.45 | 44 |
| H75A | 1943.96 | 2606.61 | 7806.07 | 42 |
| H75B | 2353.1 | 3136.27 | 8013.95 | 42 |
| H15A | 5415.44 | -1305.9 | 6369.34 | 44 |
| H15B | 5528.77 | -1440.02 | 6930.34 | 44 |
| H19A | 3645.79 | 2952.15 | 4843.23 | 46 |
| H19B | 3384.45 | 3281.55 | 5177.11 | 46 |
| H15C | 6815.79 | -772.89 | 7071.27 | 44 |
| H15D | 6531.63 | -1370.27 | 7076.95 | 44 |
| H77A | -2258.05 | 2000.34 | 5770.09 | 42 |
| H77B | -1879.14 | 1463.86 | 6014.61 | 42 |
| H39A | 4427.32 | 1999.85 | 9332.88 | 45 |
| H39B | 4860.38 | 1499.58 | 9576.09 | 45 |
| H15E | -3464.05 | 1648.62 | 2336.14 | 57 |
| H15F | -3308.55 | 1321.11 | 1933.21 | 57 |
| H15G | -3516.11 | 1638.26 | 2331.39 | 57 |
| H15H | -3294.42 | 1320.11 | 1959.34 | 57 |
| H79A | -3044.91 | 1088.48 | 4418.44 | 50 |
| H79B | -2540.11 | 641.44 | 4597.06 | 50 |
| H15I | 339.97 | -292.78 | 3473.17 | 46 |
| H15J | 252.01 | -924.5 | 3617.17 | 46 |
| H5A | -2944.09 | 3820.04 | 1053.71 | 51 |
| H5B | -3223.14 | 4141.02 | 1380.24 | 51 |
| H16C | 2202.6 | 6432.46 | 5314.77 | 53 |
| H16D | 2084.67 | 6217.34 | 4770.33 | 53 |
| H81A | 744.43 | 4485.94 | 6773.85 | 48 |
| H81B | 396.82 | 3924.95 | 6542.78 | 48 |
| H16E | 1511.08 | 203.08 | 3782.38 | 71 |
| H16F | 1736.19 | 523.61 | 4300.64 | 71 |
| H16G | 1831.31 | 794.03 | 3841.53 | 71 |
| H41A | -925.27 | -1084.67 | 2771.62 | 48 |
| H41B | -712.99 | -1324.48 | 3319.38 | 48 |
| H16H | 2343.12 | 3008.17 | 8797.91 | 53 |
| H16I | 1887.2 | 2515.14 | 8598.27 | 53 |
| H83A | 13.78 | -1236.97 | 2821.73 | 58 |
| H83B | -53.64 | -607.13 | 2612.02 | 58 |
| H16A | 4341.03 | 3491.94 | 5735.95 | 50 |
| H16B | 4545.02 | 3245 | 5327.61 | 50 |
| H21A | 3538.92 | 1086.37 | 8010.73 | 55 |
| H21B | 4050.72 | 649.68 | 8175.95 | 55 |
| H21C | 3991.78 | 492.24 | 8250.8 | 55 |
| H21D | 3696.6 | 1068 | 7989.49 | 55 |
| H16J | 2575.38 | 1954.58 | 8450.86 | 78 |
| H16K | 2758.77 | 2105.62 | 9020.99 | 78 |
| H16L | 3029.29 | 2441.05 | 8695.02 | 78 |
| H85A | 4756.87 | -635.11 | 6319.49 | 60 |
| H85B | 4927.29 | -639.2 | 6899.73 | 60 |
| H85C | 4729.17 | -642.2 | 6315.04 | 60 |
| H85D | 4897.46 | -656.64 | 6901.9 | 60 |
| H43A | 2234.93 | 1742 | 5216 | 66 |
| H43B | 2449.34 | 1303.16 | 4914.86 | 66 |
| H17C | -864.05 | 3379.6 | 3436.19 | 61 |
| H17D | -984.12 | 3163.88 | 3900.4 | 61 |
| H87A | 6450.38 | -751 | 5487.94 | 79 |
| H87B | 6940.15 | -1214.28 | 5675.66 | 79 |
| H87C | 7095.09 | -563.5 | 5655.45 | 79 |
| H17A | 5133.16 | 2490.42 | 9163.24 | 60 |
| H17B | 5567.73 | 1989.08 | 9410.36 | 60 |
| H11A | 1505.67 | 5476.57 | 4899.9 | 63 |
| H11B | 1572.08 | 5785.23 | 5400.55 | 63 |
| H17I | -2506.13 | 4693.02 | 1286 | 79 |
| H17J | -2055.14 | 4212.54 | 1555.52 | 79 |
| H17K | -2355.12 | 4555.09 | 1854.38 | 79 |
| H89A | -1653.79 | -580.96 | 2933.26 | 59 |
| H89B | -1467.4 | -922.4 | 3440.31 | 59 |
| H17L | -1088.67 | 1900.67 | 5975.72 | 67 |
| H17M | -1461.03 | 2427.46 | 5692.03 | 67 |
| H45A | -3413.38 | 173.32 | 4315.53 | 71 |
| H45B | -3060.2 | 33.71 | 4882.15 | 71 |
| H18I | -1934.29 | -1409.49 | 2474.47 | 70 |
| H18J | -1666.81 | -1790.62 | 2958.2 | 70 |
| H91A | -1623.52 | 2680.39 | 6385.58 | 77 |
| H91B | -1303.29 | 2130.48 | 6669.73 | 77 |
| H18A | 5071.92 | 2679.58 | 9934.06 | 76 |
| H18B | 5507.56 | 2180.02 | 10180.57 | 76 |
| H23A | 1007.19 | -1080.19 | 3234.21 | 82 |
| H23B | 926.61 | -467.81 | 2988.22 | 82 |
| H93A | -335.6 | 4210.62 | 6772.16 | 74 |
| H93B | 17.3 | 4749.64 | 7049.9 | 74 |
| H18C | 3850.15 | 4215.61 | 5213.28 | 73 |
| H18D | 3970.85 | 3948.13 | 4766.18 | 73 |
| H95A | 2007.9 | 816.4 | 5352.82 | 113 |
| H95B | 2431.59 | 1087.38 | 5858.13 | 113 |
| H95C | 2661.23 | 649.43 | 5566.59 | 113 |
| H19H | -1215.59 | 4121.25 | 3757.95 | 99 |
| H19I | -584.57 | 4264.02 | 3818.69 | 99 |
| H19J | -707.04 | 4048.07 | 4281.86 | 99 |
| H3A | -433.83 | 4593.11 | 6015.91 | 113 |
| H3B | -60.78 | 5125.84 | 6287.26 | 113 |
| H3C | -666.34 | 5028.15 | 6307.53 | 113 |
| H19C | 5807 | 3157.28 | 9774.16 | 132 |
| H19D | 6234.1 | 2679.69 | 10089.17 | 132 |
| H19E | 5938.2 | 3089.81 | 10348.22 | 132 |
| H97A | -3935.94 | 843.31 | 4574.31 | 115 |
| H97B | -3623.14 | 605.54 | 5121.52 | 115 |
| H97C | -4028.08 | 202.12 | 4695.88 | 115 |
| H19M | -2655.63 | -1732.86 | 2691.38 | 138 |
| H19N | -2605.82 | -1064.63 | 2774.27 | 138 |
| H19O | -2351.45 | -1481.86 | 3234.84 | 138 |
| H49A | 4559.73 | 4737.58 | 5114.64 | 132 |
| H49B | 4923.37 | 4181.29 | 5143.49 | 132 |
| H49C | 4837.34 | 4401.07 | 5622.8 | 132 |
| H19P | 625.54 | -888.97 | 2197.4 | 156 |
| H19Q | 709.2 | -1501.05 | 2444.62 | 156 |
| H19R | 1243.71 | -1104.34 | 2537.54 | 156 |
| H99A | -758.48 | 3027.44 | 6401.13 | 197 |
| H99B | -451.23 | 2491.99 | 6724.62 | 197 |
| H99C | -757.94 | 2943.49 | 6939.83 | 197 |
| H19K | 1007.77 | 6224.9 | 4440.79 | 118 |
| H19L | 711.34 | 5999.86 | 4789.06 | 118 |
| H101 | 1953.73 | -975.73 | 6476.55 | 79 |
| H20D | 656.32 | 6917.63 | 4721.58 | 163 |
| H20E | 1328.04 | 6968.78 | 4924.76 | 163 |
| H20F | 1032.25 | 6742.09 | 5276.9 | 163 |
| H51 | 1350.9 | 3899.17 | 5546.5 | 68 |
| H207 | 3639.12 | 334.33 | 6638.93 | 51 |
| H17E | 3494 | 5703.29 | 5515.66 | 58 |
| H17F | 3244.44 | 6302.91 | 5577.13 | 58 |
| H18E | 3329.11 | 6564.32 | 4859.61 | 74 |
| H18F | 2814.66 | 6140.78 | 4572.47 | 74 |
| H18G | 2911.37 | 5947.6 | 4575.54 | 74 |
| H18H | 3072.39 | 6571.61 | 4787.96 | 74 |
| H20G | 4072.9 | 5723.57 | 5016.16 | 95 |
| H20H | 3567.62 | 5274.65 | 4777.67 | 95 |
| H21M | 3300.65 | 5675.9 | 4024.39 | 130 |
| H21N | 3972.94 | 5646.58 | 4217.13 | 130 |
| H21O | 3670.45 | 6228.27 | 4243.44 | 130 |
| H21P | 4048.33 | 6298.21 | 5096.11 | 89 |
| H21Q | 3860.52 | 5708.19 | 4827.13 | 89 |
| H1AA | 3542.28 | 6227.28 | 4092.45 | 204 |
| H1AB | 4206.02 | 6146.33 | 4387.81 | 204 |
| H1AC | 3912.49 | 6730.55 | 4423.09 | 204 |
| H212 | 4578.26 | 1084.59 | 7037.06 | 79 |
| H51A | 327.4 | 4536.11 | 5244.9 | 77 |
| H213 | 2934.67 | -318.57 | 6939.79 | 73 |
| H25A | 3202.81 | 159.91 | 7932.68 | 71 |
| H25B | 3563.24 | 46.38 | 8504.08 | 71 |
| H10A | 2664.71 | 833.04 | 8152.04 | 115 |
| H10B | 3009.92 | 680.66 | 8718.9 | 115 |
| H10C | 2612.5 | 217.31 | 8352.9 | 115 |
| H25C | 2917.49 | 896.25 | 8259.65 | 84 |
| H25D | 2953.41 | 396.67 | 7908.41 | 84 |
| H21E | 2842.38 | -53.32 | 8541.63 | 164 |
| H21F | 3285.51 | 347.47 | 8943.92 | 164 |
| H21G | 3501.85 | -117.07 | 8664.02 | 164 |
| H19F | 4262.2 | 3104.15 | 5368.05 | 72 |
| H19G | 3798.93 | 2870.4 | 4869.18 | 72 |
| H21H | 3118.08 | 3462.56 | 4975.54 | 64 |
| H21I | 3587.37 | 3700.53 | 5473.11 | 64 |
| H1BA | 3370.2 | 4283.07 | 4758.16 | 56 |
| H1BB | 3660.41 | 3820.05 | 4545.32 | 56 |
| H49D | 4183.98 | 4685.11 | 4892.14 | 57 |
| H49E | 4524.45 | 4102.45 | 5015.38 | 57 |
| H49F | 4328.3 | 4388.37 | 5414.51 | 57 |
| H18K | -4410.98 | 1455 | 1921.06 | 89 |
| H18L | -4302.37 | 1198.54 | 1466.28 | 89 |
| H20I | -4483.42 | 522.02 | 1844.39 | 163 |
| H20J | -4043.73 | 737.85 | 2366.57 | 163 |
| H20K | -3821.31 | 495.41 | 1968.12 | 163 |
| H21R | -4150.96 | 937.06 | 1929.01 | 59 |
| H21S | -4441.85 | 1535.9 | 1787.99 | 59 |
| H1CA | -4517.75 | 1452.78 | 1034.78 | 134 |
| H1CB | -4749.03 | 891.47 | 1193.62 | 134 |
| H1CC | -4142.76 | 891.68 | 1161.49 | 134 |
| H47A | 4020.47 | -1089.93 | 6386.74 | 66 |
| H47B | 4393.04 | -1609.23 | 6335.06 | 66 |
| H20A | 4129.4 | -1777.15 | 6998.61 | 87 |
| H20B | 4404.86 | -1191.48 | 7246.01 | 87 |
| H20C | 4798.23 | -1694.92 | 7204.65 | 87 |
| H21J | 3804.88 | -899.18 | 6353.62 | 101 |
| H21K | 4007.44 | -1122.12 | 6907.93 | 101 |
| H21L | 3677.48 | -1535.76 | 6459.26 | 101 |
| H47C | 4432.77 | -1597.36 | 6235.64 | 76 |
| H47D | 4689.52 | -1681 | 6821.92 | 76 |

Table 8 Atomic Occupancy for z.

| Atom | *Occupancy* |  | Atom | *Occupancy* |  | Atom | *Occupancy* |
| --- | --- | --- | --- | --- | --- | --- | --- |
| Cl1 | 0.488(10) |  | Cl4 | 0.640(8) |  | Cl8 | 0.488(10) |
| Cl9 | 0.609(9) |  | Cl5 | 0.609(9) |  | Cl3 | 0.488(10) |
| Cl7 | 0.640(8) |  | Cl2 | 0.640(8) |  | C19 | 0.893(5) |
| H19A | 0.893(5) |  | H19B | 0.893(5) |  | H15E | 0.739(9) |
| H15F | 0.739(9) |  | H15G | 0.261(9) |  | H15H | 0.261(9) |
| C167 | 0.893(5) |  | H16A | 0.893(5) |  | H16B | 0.893(5) |
| H21A | 0.646(10) |  | H21B | 0.646(10) |  | H21C | 0.354(10) |
| H21D | 0.354(10) |  | H85A | 0.671(8) |  | H85B | 0.671(8) |
| H85C | 0.329(8) |  | H85D | 0.329(8) |  | C189 | 0.893(5) |
| H18C | 0.893(5) |  | H18D | 0.893(5) |  | C49 | 0.893(5) |
| H49A | 0.893(5) |  | H49B | 0.893(5) |  | H49C | 0.893(5) |
| C101 | 0.640(8) |  | H101 | 0.640(8) |  | C51 | 0.609(9) |
| H51 | 0.609(9) |  | C207 | 0.488(10) |  | H207 | 0.488(10) |
| H18E | 0.552(8) |  | H18F | 0.552(8) |  | H18G | 0.448(8) |
| H18H | 0.448(8) |  | C205 | 0.552(8) |  | H20G | 0.552(8) |
| H20H | 0.552(8) |  | C210 | 0.552(8) |  | H21M | 0.552(8) |
| H21N | 0.552(8) |  | H21O | 0.552(8) |  | C211 | 0.448(8) |
| H21P | 0.448(8) |  | H21Q | 0.448(8) |  | C1A | 0.448(8) |
| H1AA | 0.448(8) |  | H1AB | 0.448(8) |  | H1AC | 0.448(8) |
| C212 | 0.512(10) |  | H212 | 0.512(10) |  | Cl3A | 0.512(10) |
| Cl1A | 0.512(10) |  | Cl8A | 0.512(10) |  | C51A | 0.391(9) |
| H51A | 0.391(9) |  | Cl9A | 0.391(9) |  | Cl5A | 0.391(9) |
| C213 | 0.360(8) |  | H213 | 0.360(8) |  | Cl4A | 0.360(8) |
| Cl7A | 0.360(8) |  | Cl2A | 0.360(8) |  | C25 | 0.646(10) |
| H25A | 0.646(10) |  | H25B | 0.646(10) |  | C103 | 0.646(10) |
| H10A | 0.646(10) |  | H10B | 0.646(10) |  | H10C | 0.646(10) |
| C25A | 0.354(10) |  | H25C | 0.354(10) |  | H25D | 0.354(10) |
| C214 | 0.354(10) |  | H21E | 0.354(10) |  | H21F | 0.354(10) |
| H21G | 0.354(10) |  | C19A | 0.107(5) |  | H19F | 0.107(5) |
| H19G | 0.107(5) |  | C215 | 0.107(5) |  | H21H | 0.107(5) |
| H21I | 0.107(5) |  | C1B | 0.107(5) |  | H1BA | 0.107(5) |
| H1BB | 0.107(5) |  | C49A | 0.107(5) |  | H49D | 0.107(5) |
| H49E | 0.107(5) |  | H49F | 0.107(5) |  | C187 | 0.739(9) |
| H18K | 0.739(9) |  | H18L | 0.739(9) |  | C209 | 0.739(9) |
| H20I | 0.739(9) |  | H20J | 0.739(9) |  | H20K | 0.739(9) |
| C216 | 0.261(9) |  | H21R | 0.261(9) |  | H21S | 0.261(9) |
| C1C | 0.261(9) |  | H1CA | 0.261(9) |  | H1CB | 0.261(9) |
| H1CC | 0.261(9) |  | C47 | 0.671(8) |  | H47A | 0.671(8) |
| H47B | 0.671(8) |  | C201 | 0.671(8) |  | H20A | 0.671(8) |
| H20B | 0.671(8) |  | H20C | 0.671(8) |  | C217 | 0.329(8) |
| H21J | 0.329(8) |  | H21K | 0.329(8) |  | H21L | 0.329(8) |
| C47A | 0.329(8) |  | H47C | 0.329(8) |  | H47D | 0.329(8) |

Table 9 Solvent masks information for z.

| Number | X | Y | Z | Volume | Electron count | Content |
| --- | --- | --- | --- | --- | --- | --- |
| 1 | 0.045 | 0.228 | 0.771 | 53.1 | 0.8 | ? |
| 2 | 0.045 | 0.272 | 0.271 | 53.1 | 0.8 | ? |
| 3 | -0.045 | 0.728 | 0.729 | 53.1 | 0.8 | ? |
| 4 | -0.045 | 0.772 | 0.229 | 53.1 | 0.8 | ? |
| 5 | 0.432 | 0.200 | 0.424 | 7.1 | 0.0 | ? |
| 6 | 0.432 | 0.300 | 0.924 | 7.1 | 0.0 | ? |
| 7 | 0.568 | 0.700 | 0.076 | 7.1 | 0.0 | ? |
| 8 | 0.568 | 0.800 | 0.576 | 7.1 | 0.0 | ? |

Experimental

Single crystals of C70H61Cl3S3
[z]
were
[].
A suitable crystal was selected and
[]
on a
Bruker APEX-II CCD
diffractometer. The crystal was kept at 153.00 K during data collection.
Using Olex2 [1], the structure was solved with the
SHELXT
[2] structure solution program using
Intrinsic Phasing
and refined with the
SHELXL
[3] refinement package using
Least Squares
minimisation.

1. Dolomanov, O.V., Bourhis, L.J., Gildea, R.J, Howard, J.A.K. & Puschmann, H.
   (2009), J. Appl. Cryst. 42, 339-341.
2. Sheldrick, G.M. (2015). Acta Cryst. A71, 3-8.
3. Sheldrick, G.M. (2015). Acta Cryst. C71, 3-8.

Crystal structure determination of
[z]

**Crystal Data**
for C70H61Cl3S3 (*M*=1104.71 g/mol):
monoclinic, space group P21/c (no. 14),
*a* = 25.7504(12) Å, *b* = 23.7050(11) Å, *c* = 29.4867(13) Å, *β* = 112.839(2)°,
*V*= 16587.9(13) Å3,
*Z* = 12,
*T* = 153.00 K,
μ(CuKα) = 2.891 mm-1,
*Dcalc* = 1.327 g/cm3,
259572 reflections measured (4.946° ≤ 2Θ ≤ 136.698°),
30313 unique (*R*int = 0.0516, Rsigma = 0.0251) which were used in all calculations.
The final *R*1 was 0.0480
(I > 2σ(I)) and *wR*2 was 0.1428 (all data).

Refinement model description

Number of restraints - 717,
number of constraints - unknown.

Details:

```
1. Fixed Uiso
```

This report has been created with Olex2, compiled on
2022.04.07 svn.rca3783a0 for OlexSys. Please
let us know
if there are any errors or if you would like to have additional features.
