# Supplementary material for: Trichalcogenasupersumanenes and its concave-convex supramolecular assembly with fullerenes
Source: Nat Commun. 2023 Jun 10;14:3446. doi: 10.1038/s41467-023-39086-0 (PMC10257710; doi:10.1038/s41467-023-39086-0)

# checkCIF/PLATON report

Structure factors have been supplied for datablock(s) s

THIS REPORT IS FOR GUIDANCE ONLY. IF USED AS PART OF A REVIEW PROCEDURE FOR PUBLICATION, IT SHOULD NOT REPLACE THE EXPERTISE OF AN EXPERIENCED CRYSTALLOGRAPHIC REFEREE.

No syntax errors found. CIF dictionary Interpreting this report

## Datablock: s

---

Bond precision: C-C = 0.0049 A Wavelength=1.54178

| | | | |
|---|---|---|---|
| Cell: | a=13.2216(10) | b=13.4167(10) | c=21.2250(16) |
| | alpha=92.016(3) | beta=97.445(3) | gamma=109.711(3) |
| Temperature: | 152 K | | |

| | Calculated | Reported |
|---|---|---|
| Volume | 3502.0(5) | 3502.0(5) |
| Space group | P -1 | P -1 |
| Hall group | -P 1 | -P 1 |
| Moiety formula | C60, C51 H24 S3, 2(C6 H4 Cl2) | C51 H24 S3, 2(C6 H4 Cl2), C60 |
| Sum formula | C123 H32 Cl4 S3 | C123 H32 Cl4 S3 |
| Mr | 1747.47 | 1747.46 |
| Dx,g cm-3 | 1.657 | 1.657 |
| Z | 2 | 2 |
| Mu (mm-1) | 2.910 | 2.910 |
| F000 | 1772.0 | 1772.0 |
| F000’ | 1781.20 | |
| h,k,lmax | 15,16,25 | 15,16,25 |
| Nref | 12839 | 12460 |
| Tmin,Tmax | 0.403,0.559 | 0.653,0.753 |
| Tmin’ | 0.272 | |

Correction method= # Reported T Limits: Tmin=0.653 Tmax=0.753
AbsCorr = MULTI-SCAN

Data completeness= 0.970 Theta(max)= 68.283

R(reflections)= 0.0500( 11141) wR2(reflections)= 0.1166( 12460)

S = 1.072 Npar= 1177

The following ALERTS were generated. Each ALERT has the format
**test-name_ALERT_alert-type_alert-level.**
Click on the hyperlinks for more details of the test.

### Alert level C

```
DIFMN02_ALERT_2_C  The minimum difference density is < -0.1*ZMAX*0.75
            _refine_diff_density_min given =     -1.444
            Test value =     -1.275
DIFMN03_ALERT_1_C  The minimum difference density is < -0.1*ZMAX*0.75
            The relevant atom site should be identified.
DIFMX02_ALERT_1_C  The maximum difference density is > 0.1*ZMAX*0.75
            The relevant atom site should be identified.
PLAT029_ALERT_3_C _diffrn_measured_fraction_theta_full value Low .      0.973 Why?
PLAT042_ALERT_1_C Calc. and Reported MoietyFormula Strings  Differ     Please Check
PLAT097_ALERT_2_C Large Reported Max.  (Positive) Residual Density       1.34 eA-3
PLAT098_ALERT_2_C Large Reported Min.  (Negative) Residual Density      -1.44 eA-3
PLAT244_ALERT_4_C Low    'Solvent' Ueq as Compared to Neighbors of       C105 Check
PLAT334_ALERT_2_C Small <C-C> Benzene Dist.   C51      -C115     .       1.37 Ang.
PLAT340_ALERT_3_C Low Bond Precision on  C-C Bonds ...............     0.0049 Ang.
PLAT906_ALERT_3_C Large K Value in the Analysis of Variance ......      2.532 Check
PLAT911_ALERT_3_C Missing FCF Refl Between Thmin & STh/L=    0.600        345 Report
```

### Alert level G

```
PLAT083_ALERT_2_G SHELXL Second Parameter in WGHT  Unusually Large       9.27 Why ?
PLAT154_ALERT_1_G The s.u.'s on the Cell Angles are Equal ..(Note)      0.003 Degree
PLAT333_ALERT_2_G Large Aver C6-Ring C-C Dist C1       -C16      .       1.42 Ang.
PLAT333_ALERT_2_G Large Aver C6-Ring C-C Dist C4       -C26      .       1.42 Ang.
PLAT333_ALERT_2_G Large Aver C6-Ring C-C Dist C6       -C20      .       1.42 Ang.
PLAT333_ALERT_2_G Large Aver C6-Ring C-C Dist C10      -C26      .       1.42 Ang.
PLAT343_ALERT_2_G Unusual sp?      Angle Range in Main Residue for         C2 Check
PLAT343_ALERT_2_G Unusual sp?      Angle Range in Main Residue for         C3 Check
PLAT343_ALERT_2_G Unusual sp?      Angle Range in Main Residue for         C5 Check
PLAT343_ALERT_2_G Unusual sp?      Angle Range in Main Residue for         C7 Check
PLAT343_ALERT_2_G Unusual sp?      Angle Range in Main Residue for        C11 Check
PLAT343_ALERT_2_G Unusual sp?      Angle Range in Main Residue for        C19 Check
PLAT343_ALERT_2_G Unusual sp?      Angle Range in Main Residue for        C25 Check
PLAT343_ALERT_2_G Unusual sp?      Angle Range in Main Residue for        C27 Check
PLAT343_ALERT_2_G Unusual sp?      Angle Range in Main Residue for        C33 Check
PLAT343_ALERT_2_G Unusual sp?      Angle Range in Main Residue for        C37 Check
PLAT343_ALERT_2_G Unusual sp?      Angle Range in Main Residue for        C43 Check
PLAT343_ALERT_2_G Unusual sp?      Angle Range in Main Residue for        C59 Check
PLAT343_ALERT_2_G Unusual sp?      Angle Range in Main Residue for        C61 Check
PLAT343_ALERT_2_G Unusual sp?      Angle Range in Main Residue for        C67 Check
PLAT343_ALERT_2_G Unusual sp?      Angle Range in Main Residue for        C69 Check
PLAT343_ALERT_2_G Unusual sp?      Angle Range in Main Residue for        C73 Check
PLAT343_ALERT_2_G Unusual sp?      Angle Range in Main Residue for        C75 Check
PLAT343_ALERT_2_G Unusual sp?      Angle Range in Main Residue for        C81 Check
PLAT343_ALERT_2_G Unusual sp?      Angle Range in Main Residue for        C89 Check
PLAT343_ALERT_2_G Unusual sp?      Angle Range in Main Residue for        C91 Check
PLAT343_ALERT_2_G Unusual sp?      Angle Range in Main Residue for        C95 Check
PLAT343_ALERT_2_G Unusual sp?      Angle Range in Main Residue for        C96 Check
PLAT343_ALERT_2_G Unusual sp?      Angle Range in Main Residue for        C97 Check
PLAT343_ALERT_2_G Unusual sp?      Angle Range in Main Residue for       C101 Check
PLAT343_ALERT_2_G Unusual sp?      Angle Range in Main Residue for       C108 Check
```

```
PLAT343_ALERT_2_G Unusual sp?      Angle Range in Main Residue for       C109 Check
PLAT343_ALERT_2_G Unusual sp?      Angle Range in Main Residue for       C110 Check
PLAT343_ALERT_2_G Unusual sp?      Angle Range in Main Residue for       C114 Check
PLAT343_ALERT_2_G Unusual sp?      Angle Range in Main Residue for       C116 Check
PLAT343_ALERT_2_G Unusual sp?      Angle Range in Main Residue for       C118 Check
PLAT343_ALERT_2_G Unusual sp?      Angle Range in Main Residue for       C120 Check
PLAT343_ALERT_2_G Unusual sp?      Angle Range in Main Residue for       C123 Check
PLAT790_ALERT_4_G Centre of Gravity not Within Unit Cell: Resd.  #          2 Note
              C51 H24 S3
PLAT790_ALERT_4_G Centre of Gravity not Within Unit Cell: Resd.  #          4 Note
              C6 H4 Cl2
PLAT912_ALERT_4_G Missing # of FCF Reflections Above STh/L=  0.600         35 Note
PLAT933_ALERT_2_G Number of HKL-OMIT Records in Embedded .res File         49 Note
PLAT978_ALERT_2_G Number C-C Bonds with Positive Residual Density.          3 Info
```

---

0 **ALERT level A** = Most likely a serious problem - resolve or explain
0 **ALERT level B** = A potentially serious problem, consider carefully
12 **ALERT level C** = Check. Ensure it is not caused by an omission or oversight
43 **ALERT level G** = General information/check it is not something unexpected

4 ALERT type 1 CIF construction/syntax error, inconsistent or missing data
43 ALERT type 2 Indicator that the structure model may be wrong or deficient
4 ALERT type 3 Indicator that the structure quality may be low
4 ALERT type 4 Improvement, methodology, query or suggestion
0 ALERT type 5 Informative message, check

---

---

It is advisable to attempt to resolve as many as possible of the alerts in all categories. Often the minor alerts point to easily fixed oversights, errors and omissions in your CIF or refinement strategy, so attention to these fine details can be worthwhile. In order to resolve some of the more serious problems it may be necessary to carry out additional measurements or structure refinements. However, the purpose of your study may justify the reported deviations and the more serious of these should normally be commented upon in the discussion or experimental section of a paper or in the "special_details" fields of the CIF. checkCIF was carefully designed to identify outliers and unusual parameters, but every test has its limitations and alerts that are not important in a particular case may appear. Conversely, the absence of alerts does not guarantee there are no aspects of the results needing attention. It is up to the individual to critically assess their own results and, if necessary, seek expert advice.

**Publication of your CIF in IUCr journals**

A basic structural check has been run on your CIF. These basic checks will be run on all CIFs submitted for publication in IUCr journals (*Acta Crystallographica*, *Journal of Applied Crystallography*, *Journal of Synchrotron Radiation*); however, if you intend to submit to *Acta Crystallographica Section C* or *E* or *IUCrData*, you should make sure that full publication checks are run on the final version of your CIF prior to submission.

**Publication of your CIF in other journals**

Please refer to the *Notes for Authors* of the relevant journal for any special instructions relating to CIF submission.

---

**PLATON version of 28/11/2022; check.def file version of 28/11/2022**

**Datablock s** - ellipsoid plot

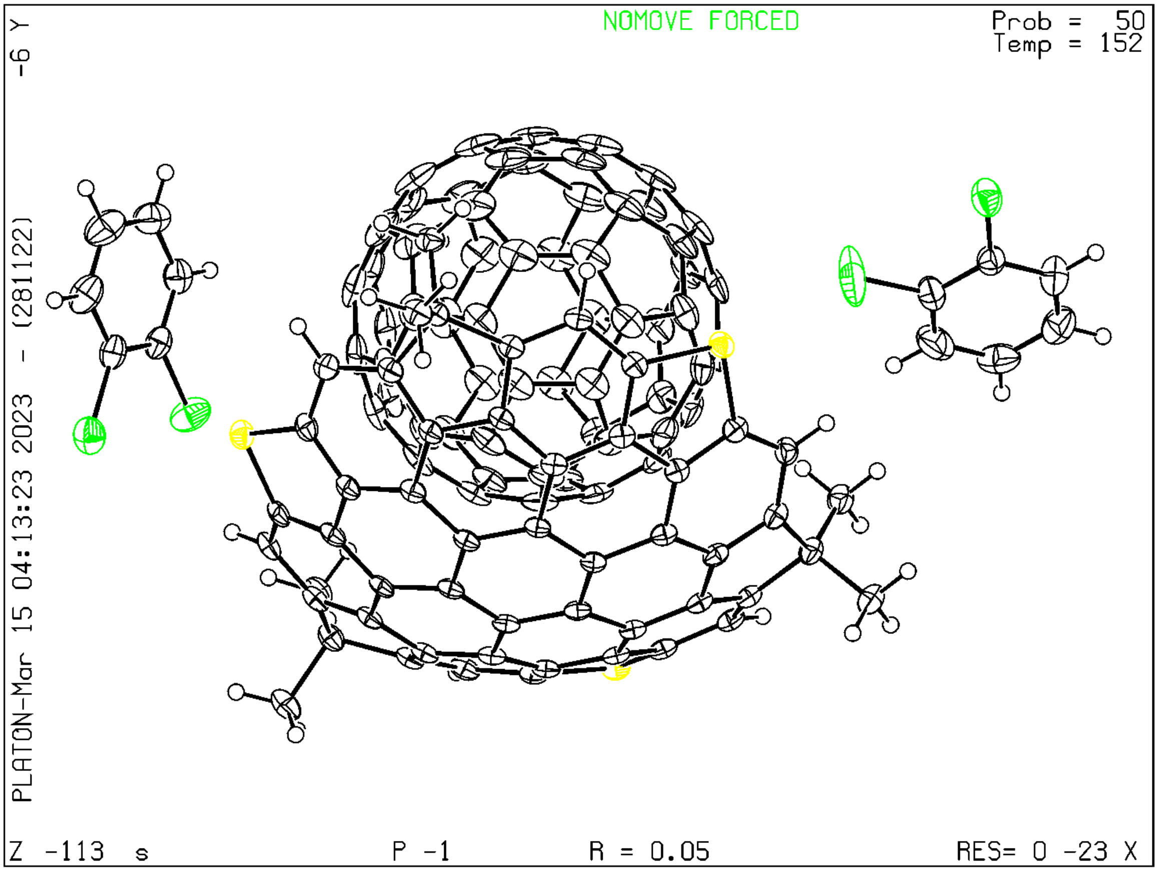

Supplement: Supplementary file 4 — Supplementary Data 1 [file 41467_2023_39086_MOESM4_ESM.zip › 1a-Me@C60/1a-Me@C60_cifreport.pdf]
