# Supplementary material for: Trichalcogenasupersumanenes and its concave-convex supramolecular assembly with fullerenes
Source: Nat Commun. 2023 Jun 10;14:3446. doi: 10.1038/s41467-023-39086-0 (PMC10257710; doi:10.1038/s41467-023-39086-0)
Supplement: Supplementary file 4 — Supplementary Data 1 [file 41467_2023_39086_MOESM4_ESM.zip › 1a-Me@C60/1a-Me@C60_tables.html]

s


# s

Table 1 Crystal data and structure refinement for s.

| Identification code | s |
| Empirical formula | C123H32Cl4S3 |
| Formula weight | 1747.46 |
| Temperature/K | 152.00 |
| Crystal system | triclinic |
| Space group | P-1 |
| a/Å | 13.2216(10) |
| b/Å | 13.4167(10) |
| c/Å | 21.2250(16) |
| α/° | 92.016(3) |
| β/° | 97.445(3) |
| γ/° | 109.711(3) |
| Volume/Å3 | 3502.0(5) |
| Z | 2 |
| ρcalcg/cm3 | 1.657 |
| μ/mm‑1 | 2.910 |
| F(000) | 1772.0 |
| Crystal size/mm3 | 0.4 × 0.3 × 0.2 |
| Radiation | CuKα (λ = 1.54178) |
| 2Θ range for data collection/° | 4.214 to 136.566 |
| Index ranges | -15 ≤ h ≤ 15, -16 ≤ k ≤ 14, -25 ≤ l ≤ 25 |
| Reflections collected | 104498 |
| Independent reflections | 12460 [Rint = 0.0550, Rsigma = 0.0254] |
| Data/restraints/parameters | 12460/0/1177 |
| Goodness-of-fit on F2 | 1.072 |
| Final R indexes [I>=2σ (I)] | R1 = 0.0500, wR2 = 0.1134 |
| Final R indexes [all data] | R1 = 0.0557, wR2 = 0.1166 |
| Largest diff. peak/hole / e Å-3 | 1.34/-1.44 |

Table 2 Fractional Atomic Coordinates (×104) and Equivalent Isotropic Displacement Parameters (Å2×103) for s. Ueq is defined as 1/3 of the trace of the orthogonalised UIJ tensor.

| Atom | *x* | *y* | *z* | U(eq) |
| --- | --- | --- | --- | --- |
| S1 | -1686.7(5) | 9509.5(6) | 1035.1(3) | 23.59(15) |
| S3 | 2207.4(6) | 9319.5(6) | 5117.7(3) | 24.75(15) |
| S2 | 5283.5(6) | 14852.2(6) | 2457.8(3) | 26.84(16) |
| Cl1 | 1078.2(7) | 4217.4(7) | 4954.5(4) | 44.2(2) |
| Cl4 | 5352.4(7) | 13677.3(8) | 346.9(4) | 47.0(2) |
| Cl2 | 6398.4(7) | 15966.3(7) | 1075.5(4) | 43.3(2) |
| Cl3 | 1257.2(12) | 5917.7(10) | 3976.5(7) | 98.2(6) |
| C1 | 2373(2) | 12067(2) | 4464.1(12) | 19.3(5) |
| C4 | 625(2) | 11487(2) | 3735.7(12) | 18.5(5) |
| C6 | 1536(2) | 13124(2) | 2940.2(12) | 19.2(5) |
| C8 | 2121(2) | 13097(2) | 3550.4(12) | 19.0(5) |
| C10 | -837(2) | 10568(2) | 2846.6(12) | 19.3(5) |
| C12 | 1665(2) | 12279(2) | 3944.7(12) | 18.7(5) |
| C14 | -1172(2) | 10415(2) | 2183.5(13) | 20.3(6) |
| C16 | 2080(2) | 11081(2) | 4724.2(12) | 19.9(6) |
| C18 | -727(2) | 11223(2) | 1791.3(12) | 20.6(6) |
| C20 | 2128(2) | 13753(2) | 2475.3(13) | 20.4(6) |
| C22 | 1686(2) | 13587(2) | 1834.5(13) | 21.2(6) |
| C24 | 1051(2) | 10303(2) | 4519.8(12) | 20.0(6) |
| C26 | 43(2) | 11517(2) | 3128.6(12) | 19.4(5) |
| C28 | 501(2) | 12339(2) | 2731.1(12) | 19.8(6) |
| C30 | 3926(2) | 13469(2) | 4180.9(12) | 19.4(5) |
| C32 | 655(2) | 12806(2) | 1626.7(13) | 21.3(6) |
| C34 | -1688(2) | 9396(2) | 1868.1(13) | 22.0(6) |
| C36 | -1120(2) | 9655(2) | 3164.7(13) | 20.8(6) |
| C38 | 3474(2) | 12654(2) | 4572.0(12) | 19.0(5) |
| C40 | 3832(2) | 14239(2) | 3204.1(13) | 21.1(6) |
| C42 | 69(2) | 12184(2) | 2058.2(12) | 20.3(6) |
| C44 | 4284(2) | 12305(2) | 4888.5(12) | 20.3(6) |
| C46 | 532(2) | 12501(2) | 970.4(13) | 22.7(6) |
| C48 | 5593(2) | 14240(2) | 3747.1(13) | 24.9(6) |
| C50 | -1658(2) | 8617(2) | 2870.1(13) | 22.5(6) |
| C52 | -869(2) | 10884(2) | 1144.2(13) | 22.3(6) |
| C54 | 3275(2) | 13690(2) | 3677.5(12) | 20.3(6) |
| C56 | 3978(2) | 11309(2) | 5117.3(12) | 21.9(6) |
| C58 | 5399(2) | 13142(2) | 4800.2(13) | 21.8(6) |
| C60 | -548(2) | 9626(2) | 3772.2(13) | 21.6(6) |
| C62 | -237(2) | 11527(2) | 720.7(13) | 23.3(6) |
| C64 | 3418(2) | 14313(2) | 1460.3(13) | 24.7(6) |
| C66 | -682(2) | 8574(2) | 3902.4(13) | 23.5(6) |
| C68 | 4950(2) | 14489(2) | 3224.2(13) | 23.8(6) |
| C70 | 85(2) | 8380(2) | 4352.6(13) | 24.9(6) |
| C72 | 5055(2) | 13714(2) | 4226.3(13) | 22.2(6) |
| C74 | 3259(2) | 14258(2) | 2601.0(13) | 21.8(6) |
| C76 | 2853(2) | 10693(2) | 5032.4(12) | 21.7(6) |
| C78 | 6271(2) | 12671(2) | 4694.7(14) | 27.0(6) |
| C80 | 3895(2) | 14517(2) | 2113.4(14) | 23.5(6) |
| C82 | 1493(2) | 13318(2) | 700.6(13) | 25.2(6) |
| C84 | -1949(2) | 8474(2) | 2208.7(13) | 23.3(6) |
| C86 | 2293(2) | 13841(2) | 1327.7(13) | 23.6(6) |
| C88 | -1411(2) | 6843(2) | 3149.1(14) | 29.6(7) |
| C90 | 958(2) | 9270(2) | 4657.0(12) | 23.3(6) |
| C92 | 5806(2) | 13939(2) | 5397.2(14) | 27.5(6) |
| C94 | 1985(3) | 12814(2) | 222.7(14) | 29.8(7) |
| C96 | 1111(2) | 8102(3) | 861.1(14) | 32.6(7) |
| C98 | -1613(2) | 7832(2) | 3388.7(13) | 24.6(6) |
| C100 | 312(2) | 10513(2) | 4051.6(12) | 20.1(6) |
| C102 | 6638(2) | 14071(3) | 787.0(14) | 30.5(7) |
| C104 | 106(3) | 3986(3) | 4282.9(15) | 31.6(7) |
| C106 | 1993(3) | 8116(3) | 515.5(14) | 34.9(7) |
| C108 | 1696(3) | 10048(3) | 877.7(14) | 33.4(7) |
| C110 | 994(3) | 7279(3) | 1299.3(16) | 35.5(7) |
| C112 | 3210(3) | 7716(3) | 3342.1(15) | 39.0(8) |
| C114 | 3344(3) | 11896(2) | 2251.6(16) | 35.1(7) |
| C116 | 2420(3) | 7312(3) | 741.7(14) | 35.9(8) |
| C118 | 2696(3) | 9079(3) | 360.7(14) | 36.4(8) |
| C120 | 2317(3) | 6487(2) | 1759.9(16) | 36.7(8) |
| C122 | 4389(3) | 11265(3) | 3330.8(15) | 36.2(8) |
| C123 | 1567(3) | 9773(3) | 2970.4(15) | 34.2(7) |
| C31 | -2690(2) | 7485(3) | 3669.6(14) | 30.4(7) |
| C63 | 1693(3) | 10593(3) | 2530.2(15) | 33.0(7) |
| C2 | 2048(3) | 6652(2) | 2386.4(16) | 37.3(8) |
| C65 | 1278(3) | 7124(3) | 2454.4(16) | 36.4(8) |
| C33 | 1796(3) | 6794(2) | 1228.1(15) | 36.5(8) |
| C67 | 1031(3) | 8722(3) | 2752.5(16) | 36.3(8) |
| C17 | 7105(3) | 15083(3) | 1097.8(15) | 33.1(7) |
| C69 | 4496(3) | 12094(2) | 2313.2(17) | 38.5(8) |
| C35 | 3288(3) | 11077(3) | 3265.1(14) | 33.8(7) |
| C71 | 1095(3) | 14166(2) | 380.0(14) | 32.6(7) |
| C9 | 1878(3) | 10833(3) | 1406.1(15) | 33.2(7) |
| C73 | 2962(3) | 9307(3) | 3643.3(13) | 34.9(7) |
| C37 | 2887(3) | 11602(2) | 1581.8(15) | 34.5(7) |
| C75 | 3596(3) | 10869(3) | 727.8(15) | 39.8(8) |
| C19 | 2747(3) | 11396(3) | 2713.0(15) | 33.3(7) |
| C77 | 2536(3) | 10064(3) | 547.0(14) | 36.4(8) |
| C39 | 971(2) | 9046(3) | 1039.7(15) | 33.4(7) |
| C79 | 738(2) | 7442(3) | 1901.8(16) | 36.3(8) |
| C5 | 1257(3) | 10311(3) | 1891.5(15) | 34.6(7) |
| C81 | 3036(3) | 6953(3) | 2838.8(16) | 39.6(8) |
| C41 | 3771(3) | 11624(3) | 1235.6(16) | 39.3(8) |
| C83 | 7201(3) | 13368(3) | 821.8(15) | 36.0(7) |
| C21 | 700(2) | 9211(3) | 1662.3(16) | 34.6(7) |
| C85 | 1460(3) | 7915(3) | 2980.7(15) | 36.0(8) |
| C43 | 2401(3) | 8199(3) | 3416.5(14) | 37.2(8) |
| C87 | 2553(3) | 10072(3) | 3423.8(14) | 33.1(7) |
| C11 | 5699(3) | 10478(3) | 3218.5(18) | 43.8(9) |
| C89 | 4112(3) | 9504(3) | 3712.5(14) | 38.6(8) |
| C45 | 4811(3) | 10456(3) | 3560.4(15) | 39.6(8) |
| C91 | 3918(3) | 6974(3) | 2495.9(17) | 44.1(9) |
| C23 | 585(2) | 8426(3) | 2081.2(16) | 35.5(8) |
| C93 | 3472(3) | 6690(3) | 1825.6(17) | 41.8(9) |
| C47 | 4261(3) | 8510(3) | 3524.5(15) | 41.8(9) |
| C95 | 5015(3) | 11786(3) | 2847.7(17) | 41.2(8) |
| C3 | 4408(3) | 10387(3) | 657.3(17) | 45.4(9) |
| C97 | 5842(3) | 9537(3) | 3041.9(19) | 49.0(10) |
| C49 | 5102(3) | 8532(3) | 3200.2(17) | 45.4(9) |
| C99 | 3525(3) | 7502(3) | 810.9(16) | 40.4(8) |
| C25 | 4759(3) | 11928(3) | 1687.4(18) | 42.3(9) |
| C101 | 4065(3) | 7177(3) | 1364.8(18) | 44.4(9) |
| C51 | -759(3) | 3047(3) | 4185.3(18) | 45.2(9) |
| C103 | 5816(3) | 11301(3) | 2779(2) | 48.3(10) |
| C13 | 8145(3) | 15396(3) | 1429.4(18) | 46.3(9) |
| C105 | 203(3) | 4722(3) | 3842.7(18) | 43.3(9) |
| C53 | 4261(3) | 8511(3) | 652.4(16) | 43.6(9) |
| C107 | 8235(3) | 13675(3) | 1157.3(17) | 45.7(9) |
| C27 | 5126(3) | 7984(3) | 1545(2) | 48.5(10) |
| C109 | 4929(3) | 7745(3) | 2671.8(19) | 47.8(10) |
| C55 | -1440(3) | 3585(3) | 3211.5(19) | 49.6(10) |
| C111 | 3841(3) | 9276(3) | 429.8(15) | 42.4(9) |
| C7 | 6081(3) | 11145(3) | 2176(2) | 52.0(10) |
| C113 | 5536(3) | 11462(3) | 1620(2) | 48.9(10) |
| C57 | 5247(3) | 8805(3) | 1107(2) | 50.2(10) |
| C115 | -1536(3) | 2847(3) | 3655(2) | 50.1(10) |
| C29 | 8708(3) | 14700(4) | 1455.5(19) | 53.8(10) |
| C117 | 5549(3) | 8265(3) | 2191(2) | 52.1(11) |
| C59 | 5357(3) | 10669(3) | 1092(2) | 50.4(10) |
| C119 | -570(3) | 4518(3) | 3303(2) | 55.6(11) |
| C15 | 6228(3) | 10158(4) | 1996(2) | 55.3(11) |
| C121 | 6107(3) | 9368(4) | 2422(2) | 54.7(11) |
| C61 | 5791(3) | 9864(3) | 1327(2) | 53.8(11) |

Table 3 Anisotropic Displacement Parameters (Å2×103) for s. The Anisotropic displacement factor exponent takes the form: -2π2[h2a\*2U11+2hka\*b\*U12+…].

| Atom | U11 | U22 | U33 | U23 | U13 | U12 |
| --- | --- | --- | --- | --- | --- | --- |
| S1 | 21.9(3) | 27.8(4) | 18.7(3) | -2.7(3) | -3.2(3) | 8.3(3) |
| S3 | 25.7(3) | 24.3(4) | 21.7(3) | 5.6(3) | -1.6(3) | 6.9(3) |
| S2 | 27.3(4) | 22.0(4) | 27.1(4) | 3.0(3) | 2.3(3) | 3.6(3) |
| Cl1 | 49.1(5) | 39.4(5) | 36.2(4) | 8.0(3) | 1.8(4) | 6.3(4) |
| Cl4 | 33.6(4) | 52.4(6) | 47.7(5) | -15.4(4) | -3.7(4) | 10.8(4) |
| Cl2 | 51.0(5) | 35.2(5) | 44.2(5) | 0.5(4) | 14.7(4) | 12.9(4) |
| Cl3 | 89.6(9) | 64.5(8) | 89.3(9) | 48.2(7) | -21.6(7) | -30.8(7) |
| C1 | 22.7(13) | 22.0(15) | 14.2(12) | -3.3(10) | 0.5(10) | 10.4(11) |
| C4 | 20.7(13) | 20.5(14) | 17.6(13) | -2.1(10) | 3.4(10) | 11.4(11) |
| C6 | 24.7(14) | 16.8(14) | 19.6(13) | -2.8(10) | 0.3(11) | 13.3(11) |
| C8 | 26.2(14) | 15.0(14) | 18.5(13) | -3.4(10) | 0.6(11) | 12.1(11) |
| C10 | 15.7(12) | 25.6(15) | 19.0(13) | -0.9(11) | 1.3(10) | 10.8(11) |
| C12 | 22.2(13) | 18.6(14) | 17.9(13) | -3.3(10) | 1.5(10) | 11.7(11) |
| C14 | 13.9(12) | 27.5(16) | 21.9(13) | -0.5(11) | 0.3(10) | 11.2(11) |
| C16 | 23.3(14) | 25.1(15) | 11.9(12) | -2.1(10) | 2.1(10) | 9.7(11) |
| C18 | 19.1(13) | 25.9(15) | 20.1(13) | 0.5(11) | -2.2(10) | 13.9(11) |
| C20 | 28.8(14) | 11.6(13) | 23.2(14) | -0.2(10) | 0.1(11) | 11.7(11) |
| C22 | 28.7(15) | 15.3(14) | 22.3(14) | 1.4(10) | -0.6(11) | 12.6(11) |
| C24 | 22.4(13) | 24.3(15) | 13.6(12) | 0.3(10) | 3.7(10) | 8.4(11) |
| C26 | 19.2(13) | 23.7(15) | 18.7(13) | -2.5(11) | 1.9(10) | 12.6(11) |
| C28 | 21.7(13) | 22.8(15) | 19.3(13) | -1.1(11) | 0.8(10) | 14.8(11) |
| C30 | 25.3(14) | 11.3(13) | 19.8(13) | -4.8(10) | -1.7(11) | 6.4(11) |
| C32 | 26.8(14) | 19.5(15) | 21.3(13) | 1.7(11) | -1.5(11) | 14.6(11) |
| C34 | 14.0(12) | 31.7(16) | 20.0(13) | -2.2(11) | -1.3(10) | 9.2(11) |
| C36 | 14.0(12) | 28.3(16) | 20.6(13) | -0.8(11) | 2.9(10) | 8.1(11) |
| C38 | 25.5(14) | 15.5(14) | 16.0(12) | -3.8(10) | -1.5(10) | 9.4(11) |
| C40 | 27.7(14) | 11.2(13) | 22.5(14) | -3.0(10) | -1.4(11) | 6.8(11) |
| C42 | 21.0(13) | 24.2(15) | 19.4(13) | -0.8(11) | -1.3(10) | 14.3(11) |
| C44 | 23.1(14) | 18.4(14) | 17.3(13) | -3.8(10) | -2.9(10) | 7.2(11) |
| C46 | 29.1(15) | 21.8(15) | 20.7(13) | 3.3(11) | -2.0(11) | 15.2(12) |
| C48 | 23.4(14) | 20.4(15) | 26.4(15) | -2.9(11) | -2.0(11) | 4.3(11) |
| C50 | 14.0(12) | 28.1(16) | 24.6(14) | 1.1(11) | 3.1(10) | 6.3(11) |
| C52 | 20.0(13) | 26.5(16) | 20.5(13) | -0.9(11) | -4.9(10) | 11.3(11) |
| C54 | 26.1(14) | 15.1(14) | 19.7(13) | -4.4(10) | -1.1(11) | 9.2(11) |
| C56 | 24.3(14) | 27.1(16) | 14.4(12) | -2.4(11) | -4.2(10) | 11.8(12) |
| C58 | 22.9(14) | 17.3(14) | 21.8(14) | -1.8(11) | -3.7(11) | 5.5(11) |
| C60 | 17.3(13) | 29.1(16) | 19.0(13) | 0.9(11) | 4.6(10) | 8.2(11) |
| C62 | 29.7(15) | 26.0(16) | 16.1(13) | 0.1(11) | -4.8(11) | 15.1(12) |
| C64 | 35.9(16) | 14.7(14) | 24.3(14) | 4.9(11) | 4.0(12) | 9.7(12) |
| C66 | 19.5(13) | 27.2(16) | 20.9(14) | 1.2(11) | 4.8(11) | 3.8(11) |
| C68 | 28.3(15) | 13.3(14) | 26.1(14) | -0.9(11) | 1.9(11) | 3.5(11) |
| C70 | 25.8(14) | 24.5(16) | 22.5(14) | 6.4(11) | 4.6(11) | 5.6(12) |
| C72 | 24.9(14) | 16.3(14) | 21.8(14) | -3.9(11) | -3.9(11) | 5.9(11) |
| C74 | 30.2(15) | 12.0(14) | 24.2(14) | -1.5(10) | -0.6(11) | 10.6(11) |
| C76 | 26.9(14) | 24.2(15) | 12.8(12) | -0.1(10) | 0.1(10) | 8.6(12) |
| C78 | 24.2(14) | 25.2(16) | 29.3(15) | -3.6(12) | -3.6(12) | 9.0(12) |
| C80 | 31.2(15) | 9.8(13) | 28.6(15) | 1.9(11) | 1.5(12) | 6.9(11) |
| C82 | 33.5(16) | 22.7(15) | 19.0(14) | 3.4(11) | -0.3(12) | 10.7(12) |
| C84 | 13.9(13) | 27.1(16) | 26.1(14) | -3.7(11) | -0.6(10) | 5.5(11) |
| C86 | 34.0(16) | 16.7(14) | 21.6(14) | 2.8(11) | 0.0(12) | 12.2(12) |
| C88 | 27.5(15) | 28.0(17) | 29.3(16) | 2.1(12) | 3.2(12) | 5.0(12) |
| C90 | 23.3(14) | 30.7(17) | 15.5(13) | 4.1(11) | 3.2(11) | 8.2(12) |
| C92 | 29.6(15) | 23.5(16) | 25.5(15) | -5.0(12) | -6.2(12) | 8.6(12) |
| C94 | 40.5(18) | 27.7(17) | 22.1(14) | 3.5(12) | 4.0(13) | 13.1(14) |
| C96 | 28.7(16) | 36.8(19) | 22.4(15) | -3.1(13) | -13.1(12) | 5.5(13) |
| C98 | 20.2(14) | 25.6(16) | 24.0(14) | 1.1(11) | 2.2(11) | 2.9(11) |
| C100 | 20.1(13) | 26.4(15) | 16.2(12) | -1.4(11) | 4.5(10) | 10.8(11) |
| C102 | 28.9(16) | 34.4(18) | 23.8(15) | 0.7(12) | 6.9(12) | 4.1(13) |
| C104 | 32.7(17) | 29.8(17) | 33.6(17) | 2.8(13) | 9.3(13) | 10.8(13) |
| C106 | 49(2) | 35.6(19) | 16.3(14) | -7.9(12) | -8.6(13) | 15.8(15) |
| C108 | 45.1(19) | 36.6(19) | 22.3(15) | 6.6(13) | -7.6(13) | 23.4(15) |
| C110 | 28.6(16) | 28.5(18) | 35.6(17) | -3.7(13) | -9.2(13) | -2.2(13) |
| C112 | 64(2) | 29.8(19) | 24.1(16) | 11.1(13) | -5.6(15) | 20.5(16) |
| C114 | 56(2) | 17.1(16) | 36.3(18) | -0.2(13) | 7.1(15) | 17.4(14) |
| C116 | 55(2) | 26.7(17) | 23.1(15) | -11.5(12) | -3.0(14) | 14.8(15) |
| C118 | 55(2) | 42(2) | 13.4(14) | -1.3(13) | 1.7(13) | 19.8(16) |
| C120 | 56(2) | 8.6(15) | 39.2(18) | -2.7(12) | -5.6(15) | 8.6(14) |
| C122 | 50(2) | 22.5(17) | 28.1(16) | -11.6(13) | -8.0(14) | 9.0(14) |
| C123 | 37.7(18) | 47(2) | 29.1(16) | 5.0(14) | 16.8(14) | 24.2(16) |
| C31 | 22.6(15) | 36.2(18) | 27.3(15) | 2.9(13) | 4.6(12) | 3.5(13) |
| C63 | 40.4(18) | 41(2) | 32.6(17) | 2.0(14) | 12.0(14) | 30.9(16) |
| C2 | 55(2) | 12.2(15) | 37.2(18) | 7.4(13) | -0.5(15) | 3.8(14) |
| C65 | 38.7(18) | 23.7(17) | 38.6(18) | 10.9(13) | 8.4(14) | -1.8(14) |
| C33 | 44.1(19) | 20.7(17) | 32.9(17) | -9.2(13) | -8.8(14) | 2.3(14) |
| C67 | 28.7(16) | 46(2) | 37.7(18) | 10.8(15) | 19.1(14) | 12.1(15) |
| C17 | 34.8(17) | 33.0(18) | 27.8(16) | 0.6(13) | 11.4(13) | 4.7(14) |
| C69 | 50(2) | 11.8(15) | 44.6(19) | -2.3(13) | 3.6(16) | 0.9(14) |
| C35 | 55(2) | 28.2(18) | 22.9(15) | -8.8(12) | 2.3(14) | 22.0(15) |
| C71 | 46.0(19) | 25.2(17) | 26.1(15) | 5.3(12) | -2.8(13) | 14.4(14) |
| C9 | 47.9(19) | 33.3(19) | 29.9(16) | 8.8(13) | 1.3(14) | 29.7(16) |
| C73 | 53(2) | 40(2) | 13.8(13) | 2.9(12) | 3.1(13) | 20.1(16) |
| C37 | 59(2) | 20.0(16) | 32.2(17) | 8.4(13) | 5.9(15) | 23.0(15) |
| C75 | 61(2) | 33.4(19) | 27.6(16) | 15.9(14) | 19.1(16) | 13.9(16) |
| C19 | 55(2) | 23.8(17) | 30.5(16) | -2.7(13) | 6.0(14) | 26.7(15) |
| C77 | 57(2) | 38(2) | 16.1(14) | 9.1(13) | 1.2(14) | 19.8(16) |
| C39 | 26.5(16) | 45(2) | 28.2(16) | 1.1(14) | -11.2(12) | 17.3(14) |
| C79 | 20.3(15) | 37.5(19) | 36.7(18) | 3.6(14) | 0.9(13) | -7.6(13) |
| C5 | 31.2(16) | 49(2) | 36.1(17) | 4.3(15) | 2.8(13) | 30.5(15) |
| C81 | 66(2) | 19.6(17) | 35.2(18) | 9.0(13) | -3.7(16) | 20.7(16) |
| C41 | 60(2) | 21.0(17) | 36.5(18) | 13.6(13) | 14.9(16) | 10.2(15) |
| C83 | 41.7(19) | 32.5(19) | 32.5(17) | 1.6(14) | 10.7(14) | 9.3(15) |
| C21 | 17.8(14) | 51(2) | 38.6(18) | 3.9(15) | -1.4(12) | 18.1(14) |
| C85 | 40.1(18) | 36.4(19) | 28.5(16) | 12.2(14) | 15.6(14) | 4.7(15) |
| C43 | 57(2) | 34.9(19) | 21.1(15) | 12.2(13) | 10.8(14) | 15.1(16) |
| C87 | 51(2) | 38.5(19) | 17.9(14) | -0.7(12) | 11.7(13) | 23.6(16) |
| C11 | 28.3(17) | 42(2) | 48(2) | -13.3(16) | -22.5(15) | 6.7(15) |
| C89 | 54(2) | 44(2) | 17.1(14) | -4.3(13) | -13.1(14) | 23.1(17) |
| C45 | 45(2) | 37(2) | 28.9(17) | -13.8(14) | -18.0(14) | 14.4(16) |
| C91 | 67(2) | 31(2) | 43(2) | -0.5(15) | -11.8(18) | 35.8(18) |
| C23 | 14.5(14) | 47(2) | 42.4(19) | 8.9(15) | 6.5(13) | 5.7(13) |
| C93 | 65(2) | 21.0(18) | 45(2) | -5.9(14) | -3.6(17) | 28.0(17) |
| C47 | 59(2) | 42(2) | 25.9(16) | 1.2(14) | -18.1(15) | 28.7(18) |
| C95 | 41.4(19) | 19.8(17) | 47(2) | -10.5(14) | -6.1(16) | -2.6(14) |
| C3 | 60(2) | 43(2) | 35.3(19) | 10.2(15) | 32.8(18) | 10.5(18) |
| C97 | 26.5(17) | 63(3) | 55(2) | -11.9(19) | -19.6(16) | 24.1(17) |
| C49 | 48(2) | 48(2) | 44(2) | -4.7(16) | -25.0(17) | 33.1(18) |
| C99 | 60(2) | 36(2) | 31.9(17) | -12.6(14) | 7.7(16) | 27.4(17) |
| C25 | 48(2) | 18.0(17) | 52(2) | 6.1(14) | 16.3(17) | -3.8(14) |
| C101 | 62(2) | 38(2) | 47(2) | -10.5(16) | 4.5(18) | 37.5(19) |
| C51 | 46(2) | 38(2) | 43(2) | 6.7(16) | 10.0(16) | 2.4(16) |
| C103 | 25.1(17) | 41(2) | 60(2) | -14.7(18) | -9.5(16) | -4.0(15) |
| C13 | 35.6(19) | 47(2) | 43(2) | -9.9(17) | 3.3(16) | 0.5(16) |
| C105 | 42(2) | 32(2) | 52(2) | 13.6(16) | 4.8(16) | 7.6(15) |
| C53 | 56(2) | 53(2) | 33.9(18) | -5.9(16) | 22.4(16) | 29.8(19) |
| C107 | 42(2) | 59(3) | 41(2) | 7.3(17) | 6.2(16) | 23.9(18) |
| C27 | 43(2) | 58(3) | 61(2) | -7.5(19) | 11.8(18) | 39.4(19) |
| C109 | 51(2) | 53(2) | 52(2) | -3.0(18) | -13.2(18) | 42(2) |
| C55 | 40(2) | 56(3) | 53(2) | -9.1(19) | -8.6(17) | 23.6(18) |
| C111 | 58(2) | 49(2) | 24.2(16) | 1.0(15) | 24.3(15) | 17.9(18) |
| C7 | 18.8(16) | 49(2) | 72(3) | -8(2) | 4.9(17) | -7.6(15) |
| C113 | 33.4(19) | 36(2) | 64(3) | 3.0(18) | 23.1(18) | -10.2(15) |
| C57 | 38(2) | 64(3) | 59(2) | -7(2) | 24.6(18) | 26.4(19) |
| C115 | 38(2) | 45(2) | 58(2) | -3.3(18) | 6.3(17) | 3.8(17) |
| C29 | 33.5(19) | 72(3) | 47(2) | -4(2) | -3.8(16) | 11.6(19) |
| C117 | 36(2) | 66(3) | 67(3) | -11(2) | -4.9(18) | 41(2) |
| C59 | 37(2) | 51(2) | 58(2) | 3.5(19) | 32.3(18) | -0.1(17) |
| C119 | 60(3) | 52(3) | 56(2) | 18(2) | -4(2) | 24(2) |
| C15 | 14.1(16) | 76(3) | 71(3) | -8(2) | 8.0(16) | 9.3(17) |
| C121 | 20.6(17) | 74(3) | 73(3) | -9(2) | -5.7(17) | 26.7(18) |
| C61 | 29.9(18) | 63(3) | 74(3) | -1(2) | 32.5(19) | 14.9(18) |

Table 4 Bond Lengths for s.

| Atom | Atom | Length/Å |  | Atom | Atom | Length/Å |
| --- | --- | --- | --- | --- | --- | --- |
| S1 | C34 | 1.780(3) |  | C110 | C79 | 1.393(5) |
| S1 | C52 | 1.784(3) |  | C112 | C81 | 1.394(5) |
| S3 | C76 | 1.778(3) |  | C112 | C43 | 1.448(5) |
| S3 | C90 | 1.787(3) |  | C112 | C47 | 1.431(5) |
| S2 | C68 | 1.782(3) |  | C114 | C69 | 1.443(5) |
| S2 | C80 | 1.782(3) |  | C114 | C37 | 1.456(5) |
| Cl1 | C104 | 1.733(3) |  | C114 | C19 | 1.385(5) |
| Cl4 | C102 | 1.729(3) |  | C116 | C33 | 1.454(5) |
| Cl2 | C17 | 1.737(4) |  | C116 | C99 | 1.383(5) |
| Cl3 | C105 | 1.719(4) |  | C118 | C77 | 1.456(5) |
| C1 | C12 | 1.452(4) |  | C118 | C111 | 1.433(5) |
| C1 | C16 | 1.402(4) |  | C120 | C2 | 1.448(5) |
| C1 | C38 | 1.385(4) |  | C120 | C33 | 1.393(5) |
| C4 | C12 | 1.429(4) |  | C120 | C93 | 1.445(5) |
| C4 | C26 | 1.422(4) |  | C122 | C35 | 1.379(5) |
| C4 | C100 | 1.448(4) |  | C122 | C45 | 1.449(5) |
| C6 | C8 | 1.428(4) |  | C122 | C95 | 1.451(5) |
| C6 | C20 | 1.453(4) |  | C123 | C63 | 1.450(5) |
| C6 | C28 | 1.420(4) |  | C123 | C67 | 1.377(5) |
| C8 | C12 | 1.420(4) |  | C123 | C87 | 1.442(5) |
| C8 | C54 | 1.447(4) |  | C63 | C19 | 1.439(5) |
| C10 | C14 | 1.406(4) |  | C63 | C5 | 1.388(4) |
| C10 | C26 | 1.449(4) |  | C2 | C65 | 1.388(5) |
| C10 | C36 | 1.382(4) |  | C2 | C81 | 1.443(5) |
| C14 | C18 | 1.405(4) |  | C65 | C79 | 1.447(5) |
| C14 | C34 | 1.403(4) |  | C65 | C85 | 1.452(5) |
| C16 | C24 | 1.409(4) |  | C67 | C85 | 1.452(5) |
| C16 | C76 | 1.397(4) |  | C67 | C23 | 1.456(5) |
| C18 | C42 | 1.404(4) |  | C17 | C13 | 1.379(5) |
| C18 | C52 | 1.400(4) |  | C69 | C95 | 1.396(5) |
| C20 | C22 | 1.388(4) |  | C69 | C25 | 1.444(5) |
| C20 | C74 | 1.403(4) |  | C35 | C19 | 1.446(4) |
| C22 | C32 | 1.414(4) |  | C35 | C87 | 1.452(5) |
| C22 | C86 | 1.408(4) |  | C9 | C37 | 1.377(5) |
| C24 | C90 | 1.393(4) |  | C9 | C5 | 1.452(5) |
| C24 | C100 | 1.406(4) |  | C73 | C43 | 1.450(5) |
| C26 | C28 | 1.429(4) |  | C73 | C87 | 1.381(5) |
| C28 | C42 | 1.448(4) |  | C73 | C89 | 1.439(5) |
| C30 | C38 | 1.412(4) |  | C37 | C41 | 1.452(5) |
| C30 | C54 | 1.388(4) |  | C75 | C77 | 1.445(5) |
| C30 | C72 | 1.405(4) |  | C75 | C41 | 1.393(5) |
| C32 | C42 | 1.392(4) |  | C75 | C3 | 1.448(5) |
| C32 | C46 | 1.411(4) |  | C39 | C21 | 1.442(5) |
| C34 | C84 | 1.419(4) |  | C79 | C23 | 1.446(5) |
| C36 | C50 | 1.411(4) |  | C5 | C21 | 1.442(5) |
| C36 | C60 | 1.417(4) |  | C81 | C91 | 1.446(6) |
| C38 | C44 | 1.411(4) |  | C41 | C25 | 1.443(5) |
| C40 | C54 | 1.405(4) |  | C83 | C107 | 1.376(5) |
| C40 | C68 | 1.395(4) |  | C21 | C23 | 1.384(5) |
| C40 | C74 | 1.406(4) |  | C85 | C43 | 1.380(5) |
| C44 | C56 | 1.385(4) |  | C11 | C45 | 1.451(5) |
| C44 | C58 | 1.562(4) |  | C11 | C97 | 1.385(5) |
| C46 | C62 | 1.392(4) |  | C11 | C103 | 1.453(6) |
| C46 | C82 | 1.561(4) |  | C89 | C45 | 1.378(5) |
| C48 | C68 | 1.429(4) |  | C89 | C47 | 1.463(5) |
| C48 | C72 | 1.392(4) |  | C91 | C93 | 1.453(5) |
| C50 | C84 | 1.395(4) |  | C91 | C109 | 1.381(6) |
| C50 | C98 | 1.560(4) |  | C93 | C101 | 1.378(6) |
| C52 | C62 | 1.420(4) |  | C47 | C49 | 1.373(6) |
| C56 | C76 | 1.421(4) |  | C95 | C103 | 1.437(5) |
| C58 | C72 | 1.561(4) |  | C3 | C111 | 1.456(5) |
| C58 | C78 | 1.526(4) |  | C3 | C59 | 1.387(6) |
| C58 | C92 | 1.542(4) |  | C97 | C49 | 1.454(6) |
| C60 | C66 | 1.403(4) |  | C97 | C121 | 1.434(6) |
| C60 | C100 | 1.387(4) |  | C49 | C109 | 1.453(5) |
| C64 | C80 | 1.423(4) |  | C99 | C101 | 1.452(5) |
| C64 | C86 | 1.391(4) |  | C99 | C53 | 1.457(5) |
| C66 | C70 | 1.397(4) |  | C25 | C113 | 1.390(6) |
| C66 | C98 | 1.563(4) |  | C101 | C27 | 1.446(6) |
| C70 | C90 | 1.414(4) |  | C51 | C115 | 1.373(5) |
| C74 | C80 | 1.397(4) |  | C103 | C7 | 1.398(6) |
| C82 | C86 | 1.566(4) |  | C13 | C29 | 1.376(6) |
| C82 | C94 | 1.526(4) |  | C105 | C119 | 1.385(5) |
| C82 | C71 | 1.549(4) |  | C53 | C111 | 1.392(5) |
| C88 | C98 | 1.522(4) |  | C53 | C57 | 1.445(6) |
| C96 | C106 | 1.451(5) |  | C107 | C29 | 1.393(6) |
| C96 | C110 | 1.450(5) |  | C27 | C57 | 1.448(6) |
| C96 | C39 | 1.388(5) |  | C27 | C117 | 1.399(6) |
| C98 | C31 | 1.547(4) |  | C109 | C117 | 1.442(6) |
| C102 | C17 | 1.387(4) |  | C55 | C115 | 1.376(6) |
| C102 | C83 | 1.384(5) |  | C55 | C119 | 1.372(6) |
| C104 | C51 | 1.374(5) |  | C7 | C113 | 1.453(6) |
| C104 | C105 | 1.369(5) |  | C7 | C15 | 1.447(6) |
| C106 | C116 | 1.445(5) |  | C113 | C59 | 1.456(5) |
| C106 | C118 | 1.393(5) |  | C57 | C61 | 1.390(6) |
| C108 | C9 | 1.450(4) |  | C117 | C121 | 1.447(6) |
| C108 | C77 | 1.384(5) |  | C59 | C61 | 1.458(6) |
| C108 | C39 | 1.446(5) |  | C15 | C121 | 1.399(6) |
| C110 | C33 | 1.439(5) |  | C15 | C61 | 1.449(6) |

Table 5 Bond Angles for s.

| Atom | Atom | Atom | Angle/˚ |  | Atom | Atom | Atom | Angle/˚ |
| --- | --- | --- | --- | --- | --- | --- | --- | --- |
| C34 | S1 | C52 | 93.20(13) |  | C35 | C122 | C45 | 119.7(3) |
| C76 | S3 | C90 | 93.17(13) |  | C35 | C122 | C95 | 120.3(3) |
| C68 | S2 | C80 | 93.06(14) |  | C45 | C122 | C95 | 108.2(3) |
| C16 | C1 | C12 | 121.0(2) |  | C67 | C123 | C63 | 120.0(3) |
| C38 | C1 | C12 | 120.4(2) |  | C67 | C123 | C87 | 120.2(3) |
| C38 | C1 | C16 | 115.1(2) |  | C87 | C123 | C63 | 107.6(3) |
| C12 | C4 | C100 | 119.1(2) |  | C19 | C63 | C123 | 108.4(3) |
| C26 | C4 | C12 | 119.8(2) |  | C5 | C63 | C123 | 119.4(3) |
| C26 | C4 | C100 | 118.9(2) |  | C5 | C63 | C19 | 120.7(3) |
| C8 | C6 | C20 | 118.4(2) |  | C65 | C2 | C120 | 119.7(3) |
| C28 | C6 | C8 | 120.0(2) |  | C65 | C2 | C81 | 119.8(3) |
| C28 | C6 | C20 | 119.2(2) |  | C81 | C2 | C120 | 108.0(3) |
| C6 | C8 | C54 | 118.7(2) |  | C2 | C65 | C79 | 120.3(3) |
| C12 | C8 | C6 | 119.7(2) |  | C2 | C65 | C85 | 120.2(3) |
| C12 | C8 | C54 | 119.0(2) |  | C79 | C65 | C85 | 107.8(3) |
| C14 | C10 | C26 | 120.5(2) |  | C110 | C33 | C116 | 108.1(3) |
| C36 | C10 | C14 | 115.2(2) |  | C120 | C33 | C110 | 120.2(3) |
| C36 | C10 | C26 | 120.9(2) |  | C120 | C33 | C116 | 119.6(3) |
| C4 | C12 | C1 | 118.3(2) |  | C123 | C67 | C85 | 119.8(3) |
| C8 | C12 | C1 | 118.9(2) |  | C123 | C67 | C23 | 120.5(3) |
| C8 | C12 | C4 | 120.4(2) |  | C85 | C67 | C23 | 107.6(3) |
| C18 | C14 | C10 | 120.4(3) |  | C102 | C17 | Cl2 | 121.0(3) |
| C34 | C14 | C10 | 121.8(3) |  | C13 | C17 | Cl2 | 119.3(3) |
| C34 | C14 | C18 | 115.3(2) |  | C13 | C17 | C102 | 119.7(3) |
| C1 | C16 | C24 | 120.5(2) |  | C114 | C69 | C25 | 108.2(3) |
| C76 | C16 | C1 | 122.1(3) |  | C95 | C69 | C114 | 119.4(3) |
| C76 | C16 | C24 | 114.6(3) |  | C95 | C69 | C25 | 120.4(4) |
| C42 | C18 | C14 | 120.2(2) |  | C122 | C35 | C19 | 120.2(3) |
| C52 | C18 | C14 | 114.7(3) |  | C122 | C35 | C87 | 120.1(3) |
| C52 | C18 | C42 | 122.2(3) |  | C19 | C35 | C87 | 107.7(3) |
| C22 | C20 | C6 | 120.3(3) |  | C108 | C9 | C5 | 107.8(3) |
| C22 | C20 | C74 | 115.0(3) |  | C37 | C9 | C108 | 120.4(3) |
| C74 | C20 | C6 | 121.1(2) |  | C37 | C9 | C5 | 120.0(3) |
| C20 | C22 | C32 | 120.4(3) |  | C87 | C73 | C43 | 119.9(3) |
| C20 | C22 | C86 | 124.8(3) |  | C87 | C73 | C89 | 120.1(3) |
| C86 | C22 | C32 | 111.1(2) |  | C89 | C73 | C43 | 108.0(3) |
| C90 | C24 | C16 | 115.4(2) |  | C9 | C37 | C114 | 120.7(3) |
| C90 | C24 | C100 | 121.9(3) |  | C9 | C37 | C41 | 119.8(3) |
| C100 | C24 | C16 | 119.9(3) |  | C41 | C37 | C114 | 107.5(3) |
| C4 | C26 | C10 | 118.7(2) |  | C77 | C75 | C3 | 108.1(3) |
| C4 | C26 | C28 | 119.9(2) |  | C41 | C75 | C77 | 120.1(3) |
| C28 | C26 | C10 | 119.0(2) |  | C41 | C75 | C3 | 119.7(4) |
| C6 | C28 | C26 | 120.2(2) |  | C114 | C19 | C63 | 120.2(3) |
| C6 | C28 | C42 | 118.8(2) |  | C114 | C19 | C35 | 119.6(3) |
| C26 | C28 | C42 | 118.4(2) |  | C63 | C19 | C35 | 108.0(3) |
| C54 | C30 | C38 | 120.4(2) |  | C108 | C77 | C118 | 120.5(3) |
| C54 | C30 | C72 | 124.9(3) |  | C108 | C77 | C75 | 119.8(3) |
| C72 | C30 | C38 | 111.1(2) |  | C75 | C77 | C118 | 107.8(3) |
| C42 | C32 | C22 | 120.8(2) |  | C96 | C39 | C108 | 120.3(3) |
| C42 | C32 | C46 | 124.4(3) |  | C96 | C39 | C21 | 120.0(3) |
| C46 | C32 | C22 | 110.7(3) |  | C21 | C39 | C108 | 108.1(3) |
| C14 | C34 | S1 | 108.0(2) |  | C110 | C79 | C65 | 119.9(3) |
| C14 | C34 | C84 | 121.4(2) |  | C110 | C79 | C23 | 119.7(3) |
| C84 | C34 | S1 | 129.6(2) |  | C23 | C79 | C65 | 108.4(3) |
| C10 | C36 | C50 | 125.0(2) |  | C63 | C5 | C9 | 119.3(3) |
| C10 | C36 | C60 | 120.5(3) |  | C63 | C5 | C21 | 120.6(3) |
| C50 | C36 | C60 | 110.5(3) |  | C21 | C5 | C9 | 107.9(3) |
| C1 | C38 | C30 | 120.7(2) |  | C112 | C81 | C2 | 119.8(3) |
| C1 | C38 | C44 | 124.8(3) |  | C112 | C81 | C91 | 119.7(3) |
| C44 | C38 | C30 | 110.8(2) |  | C2 | C81 | C91 | 108.2(3) |
| C54 | C40 | C74 | 120.0(3) |  | C75 | C41 | C37 | 119.9(3) |
| C68 | C40 | C54 | 122.1(3) |  | C75 | C41 | C25 | 120.2(3) |
| C68 | C40 | C74 | 115.0(3) |  | C25 | C41 | C37 | 108.1(3) |
| C18 | C42 | C28 | 121.1(3) |  | C107 | C83 | C102 | 120.1(3) |
| C32 | C42 | C18 | 115.2(2) |  | C5 | C21 | C39 | 108.3(3) |
| C32 | C42 | C28 | 120.4(3) |  | C23 | C21 | C39 | 119.7(3) |
| C38 | C44 | C58 | 106.4(2) |  | C23 | C21 | C5 | 120.1(3) |
| C56 | C44 | C38 | 118.9(2) |  | C65 | C85 | C67 | 108.2(3) |
| C56 | C44 | C58 | 134.0(2) |  | C43 | C85 | C65 | 120.0(3) |
| C32 | C46 | C82 | 106.9(2) |  | C43 | C85 | C67 | 120.1(3) |
| C62 | C46 | C32 | 119.3(3) |  | C112 | C43 | C73 | 108.1(3) |
| C62 | C46 | C82 | 132.9(3) |  | C85 | C43 | C112 | 119.8(3) |
| C72 | C48 | C68 | 117.3(3) |  | C85 | C43 | C73 | 119.8(3) |
| C36 | C50 | C98 | 107.2(2) |  | C123 | C87 | C35 | 108.2(3) |
| C84 | C50 | C36 | 119.1(3) |  | C73 | C87 | C123 | 120.2(3) |
| C84 | C50 | C98 | 132.8(3) |  | C73 | C87 | C35 | 119.7(3) |
| C18 | C52 | S1 | 108.4(2) |  | C45 | C11 | C103 | 107.6(3) |
| C18 | C52 | C62 | 120.9(3) |  | C97 | C11 | C45 | 119.8(4) |
| C62 | C52 | S1 | 129.3(2) |  | C97 | C11 | C103 | 120.4(4) |
| C30 | C54 | C8 | 120.5(3) |  | C73 | C89 | C47 | 107.7(3) |
| C30 | C54 | C40 | 115.1(3) |  | C45 | C89 | C73 | 120.3(3) |
| C40 | C54 | C8 | 121.3(2) |  | C45 | C89 | C47 | 119.9(3) |
| C44 | C56 | C76 | 118.0(2) |  | C122 | C45 | C11 | 107.8(3) |
| C72 | C58 | C44 | 101.5(2) |  | C89 | C45 | C122 | 120.0(3) |
| C78 | C58 | C44 | 114.4(2) |  | C89 | C45 | C11 | 120.3(3) |
| C78 | C58 | C72 | 114.4(2) |  | C81 | C91 | C93 | 107.8(3) |
| C78 | C58 | C92 | 109.3(2) |  | C109 | C91 | C81 | 119.8(3) |
| C92 | C58 | C44 | 108.1(2) |  | C109 | C91 | C93 | 120.1(4) |
| C92 | C58 | C72 | 108.7(2) |  | C79 | C23 | C67 | 108.0(3) |
| C66 | C60 | C36 | 110.7(2) |  | C21 | C23 | C67 | 119.4(3) |
| C100 | C60 | C36 | 120.2(3) |  | C21 | C23 | C79 | 120.7(3) |
| C100 | C60 | C66 | 124.9(3) |  | C120 | C93 | C91 | 107.9(3) |
| C46 | C62 | C52 | 117.9(2) |  | C101 | C93 | C120 | 120.7(3) |
| C86 | C64 | C80 | 117.3(3) |  | C101 | C93 | C91 | 119.9(4) |
| C60 | C66 | C98 | 107.3(2) |  | C112 | C47 | C89 | 108.1(3) |
| C70 | C66 | C60 | 119.1(3) |  | C49 | C47 | C112 | 120.2(3) |
| C70 | C66 | C98 | 132.6(3) |  | C49 | C47 | C89 | 119.7(4) |
| C40 | C68 | S2 | 108.3(2) |  | C69 | C95 | C122 | 119.7(3) |
| C40 | C68 | C48 | 121.2(3) |  | C69 | C95 | C103 | 120.2(4) |
| C48 | C68 | S2 | 129.1(2) |  | C103 | C95 | C122 | 107.8(3) |
| C66 | C70 | C90 | 117.4(3) |  | C75 | C3 | C111 | 107.7(3) |
| C30 | C72 | C58 | 106.6(2) |  | C59 | C3 | C75 | 120.5(3) |
| C48 | C72 | C30 | 119.3(3) |  | C59 | C3 | C111 | 119.7(4) |
| C48 | C72 | C58 | 133.3(3) |  | C11 | C97 | C49 | 119.8(4) |
| C20 | C74 | C40 | 120.2(3) |  | C11 | C97 | C121 | 120.1(4) |
| C80 | C74 | C20 | 122.1(3) |  | C121 | C97 | C49 | 107.8(4) |
| C80 | C74 | C40 | 115.1(3) |  | C47 | C49 | C97 | 120.5(3) |
| C16 | C76 | S3 | 108.4(2) |  | C47 | C49 | C109 | 120.1(4) |
| C16 | C76 | C56 | 120.9(3) |  | C109 | C49 | C97 | 107.8(4) |
| C56 | C76 | S3 | 129.4(2) |  | C116 | C99 | C101 | 120.3(3) |
| C64 | C80 | S2 | 129.5(2) |  | C116 | C99 | C53 | 120.0(3) |
| C74 | C80 | S2 | 108.1(2) |  | C101 | C99 | C53 | 108.0(3) |
| C74 | C80 | C64 | 121.3(3) |  | C41 | C25 | C69 | 108.2(3) |
| C46 | C82 | C86 | 101.4(2) |  | C113 | C25 | C69 | 119.5(4) |
| C94 | C82 | C46 | 113.7(2) |  | C113 | C25 | C41 | 120.1(3) |
| C94 | C82 | C86 | 113.8(2) |  | C93 | C101 | C99 | 119.6(3) |
| C94 | C82 | C71 | 109.2(2) |  | C93 | C101 | C27 | 120.3(3) |
| C71 | C82 | C46 | 109.3(2) |  | C27 | C101 | C99 | 107.7(4) |
| C71 | C82 | C86 | 109.2(2) |  | C115 | C51 | C104 | 120.6(4) |
| C50 | C84 | C34 | 117.4(3) |  | C95 | C103 | C11 | 108.6(3) |
| C22 | C86 | C82 | 106.7(2) |  | C7 | C103 | C11 | 119.8(4) |
| C64 | C86 | C22 | 119.4(3) |  | C7 | C103 | C95 | 119.8(4) |
| C64 | C86 | C82 | 132.9(3) |  | C29 | C13 | C17 | 119.8(3) |
| C24 | C90 | S3 | 107.9(2) |  | C104 | C105 | Cl3 | 119.8(3) |
| C24 | C90 | C70 | 121.6(3) |  | C104 | C105 | C119 | 120.0(3) |
| C70 | C90 | S3 | 129.3(2) |  | C119 | C105 | Cl3 | 120.2(3) |
| C110 | C96 | C106 | 107.5(3) |  | C111 | C53 | C99 | 119.4(3) |
| C39 | C96 | C106 | 120.1(3) |  | C111 | C53 | C57 | 120.9(4) |
| C39 | C96 | C110 | 120.4(3) |  | C57 | C53 | C99 | 107.9(3) |
| C50 | C98 | C66 | 100.9(2) |  | C83 | C107 | C29 | 119.3(4) |
| C88 | C98 | C50 | 114.2(2) |  | C101 | C27 | C57 | 108.5(3) |
| C88 | C98 | C66 | 114.2(2) |  | C117 | C27 | C101 | 119.7(4) |
| C88 | C98 | C31 | 108.7(2) |  | C117 | C27 | C57 | 119.6(4) |
| C31 | C98 | C50 | 109.5(2) |  | C91 | C109 | C49 | 120.0(4) |
| C31 | C98 | C66 | 109.1(2) |  | C91 | C109 | C117 | 120.2(4) |
| C24 | C100 | C4 | 120.8(2) |  | C117 | C109 | C49 | 107.9(4) |
| C60 | C100 | C4 | 120.8(2) |  | C119 | C55 | C115 | 119.7(4) |
| C60 | C100 | C24 | 114.9(3) |  | C118 | C111 | C3 | 108.2(3) |
| C17 | C102 | Cl4 | 120.5(3) |  | C53 | C111 | C118 | 120.4(3) |
| C83 | C102 | Cl4 | 119.2(2) |  | C53 | C111 | C3 | 119.4(4) |
| C83 | C102 | C17 | 120.3(3) |  | C103 | C7 | C113 | 119.8(4) |
| C51 | C104 | Cl1 | 119.7(3) |  | C103 | C7 | C15 | 119.4(4) |
| C105 | C104 | Cl1 | 120.6(3) |  | C15 | C7 | C113 | 108.2(4) |
| C105 | C104 | C51 | 119.6(3) |  | C25 | C113 | C7 | 120.2(4) |
| C116 | C106 | C96 | 108.3(3) |  | C25 | C113 | C59 | 120.1(4) |
| C118 | C106 | C96 | 119.9(3) |  | C7 | C113 | C59 | 107.8(4) |
| C118 | C106 | C116 | 119.8(3) |  | C53 | C57 | C27 | 108.0(4) |
| C77 | C108 | C9 | 120.1(3) |  | C61 | C57 | C53 | 120.0(4) |
| C77 | C108 | C39 | 119.8(3) |  | C61 | C57 | C27 | 120.1(4) |
| C39 | C108 | C9 | 107.9(3) |  | C51 | C115 | C55 | 119.9(4) |
| C33 | C110 | C96 | 108.4(3) |  | C13 | C29 | C107 | 120.8(3) |
| C79 | C110 | C96 | 119.5(3) |  | C27 | C117 | C109 | 119.8(4) |
| C79 | C110 | C33 | 119.9(3) |  | C27 | C117 | C121 | 120.4(4) |
| C81 | C112 | C43 | 120.3(3) |  | C109 | C117 | C121 | 107.9(4) |
| C81 | C112 | C47 | 120.2(4) |  | C3 | C59 | C113 | 119.5(4) |
| C47 | C112 | C43 | 108.2(3) |  | C3 | C59 | C61 | 120.7(4) |
| C69 | C114 | C37 | 108.0(3) |  | C113 | C59 | C61 | 107.8(4) |
| C19 | C114 | C69 | 120.8(3) |  | C55 | C119 | C105 | 120.2(4) |
| C19 | C114 | C37 | 119.1(3) |  | C7 | C15 | C61 | 108.2(4) |
| C106 | C116 | C33 | 107.7(3) |  | C121 | C15 | C7 | 120.3(4) |
| C99 | C116 | C106 | 120.2(3) |  | C121 | C15 | C61 | 119.7(4) |
| C99 | C116 | C33 | 119.9(3) |  | C97 | C121 | C117 | 108.6(4) |
| C106 | C118 | C77 | 119.5(3) |  | C15 | C121 | C97 | 120.1(4) |
| C106 | C118 | C111 | 120.2(3) |  | C15 | C121 | C117 | 119.8(4) |
| C111 | C118 | C77 | 108.2(3) |  | C57 | C61 | C59 | 119.3(4) |
| C33 | C120 | C2 | 120.0(3) |  | C57 | C61 | C15 | 120.5(4) |
| C33 | C120 | C93 | 119.8(3) |  | C15 | C61 | C59 | 108.0(4) |
| C93 | C120 | C2 | 108.1(3) |  |  |  |  |  |

Table 6 Torsion Angles for s.

| A | B | C | D | Angle/˚ |  | A | B | C | D | Angle/˚ |
| --- | --- | --- | --- | --- | --- | --- | --- | --- | --- | --- |
| S1 | C34 | C84 | C50 | 166.3(2) |  | C122 | C35 | C87 | C73 | 0.3(4) |
| S1 | C52 | C62 | C46 | -164.6(2) |  | C122 | C95 | C103 | C11 | -0.2(4) |
| Cl1 | C104 | C51 | C115 | -179.7(3) |  | C122 | C95 | C103 | C7 | 142.6(3) |
| Cl1 | C104 | C105 | Cl3 | -3.6(4) |  | C123 | C63 | C19 | C114 | 142.8(3) |
| Cl1 | C104 | C105 | C119 | 178.9(3) |  | C123 | C63 | C19 | C35 | 0.4(3) |
| Cl4 | C102 | C17 | Cl2 | 2.1(4) |  | C123 | C63 | C5 | C9 | -138.9(3) |
| Cl4 | C102 | C17 | C13 | -177.9(3) |  | C123 | C63 | C5 | C21 | -1.1(4) |
| Cl4 | C102 | C83 | C107 | 178.5(3) |  | C123 | C67 | C85 | C65 | -142.9(3) |
| Cl2 | C17 | C13 | C29 | 179.4(3) |  | C123 | C67 | C85 | C43 | 0.2(5) |
| Cl3 | C105 | C119 | C55 | -176.8(3) |  | C123 | C67 | C23 | C79 | 142.7(3) |
| C1 | C16 | C24 | C90 | 161.6(2) |  | C123 | C67 | C23 | C21 | -0.2(5) |
| C1 | C16 | C24 | C100 | 0.1(4) |  | C63 | C123 | C67 | C85 | 137.9(3) |
| C1 | C16 | C76 | S3 | -165.9(2) |  | C63 | C123 | C67 | C23 | -0.1(5) |
| C1 | C16 | C76 | C56 | 2.5(4) |  | C63 | C123 | C87 | C35 | 0.1(3) |
| C1 | C38 | C44 | C56 | -0.5(4) |  | C63 | C123 | C87 | C73 | -142.6(3) |
| C1 | C38 | C44 | C58 | 171.1(2) |  | C63 | C5 | C21 | C39 | -141.9(3) |
| C4 | C26 | C28 | C6 | 0.1(4) |  | C63 | C5 | C21 | C23 | 0.7(4) |
| C4 | C26 | C28 | C42 | 161.8(2) |  | C2 | C120 | C33 | C110 | 0.3(5) |
| C6 | C8 | C12 | C1 | 161.9(2) |  | C2 | C120 | C33 | C116 | 138.3(3) |
| C6 | C8 | C12 | C4 | -0.2(4) |  | C2 | C120 | C93 | C91 | 0.4(4) |
| C6 | C8 | C54 | C30 | -162.5(2) |  | C2 | C120 | C93 | C101 | -143.0(3) |
| C6 | C8 | C54 | C40 | -3.4(4) |  | C2 | C65 | C79 | C110 | -0.3(5) |
| C6 | C20 | C22 | C32 | 0.1(4) |  | C2 | C65 | C79 | C23 | -142.9(3) |
| C6 | C20 | C22 | C86 | -156.0(3) |  | C2 | C65 | C85 | C67 | 143.0(3) |
| C6 | C20 | C74 | C40 | -5.5(4) |  | C2 | C65 | C85 | C43 | -0.1(5) |
| C6 | C20 | C74 | C80 | 155.4(3) |  | C2 | C81 | C91 | C93 | 0.3(4) |
| C6 | C28 | C42 | C18 | 157.6(2) |  | C2 | C81 | C91 | C109 | 142.6(3) |
| C6 | C28 | C42 | C32 | -0.9(4) |  | C65 | C2 | C81 | C112 | 0.0(5) |
| C8 | C6 | C20 | C22 | 162.6(2) |  | C65 | C2 | C81 | C91 | -142.1(3) |
| C8 | C6 | C20 | C74 | 4.8(4) |  | C65 | C79 | C23 | C67 | -0.2(3) |
| C8 | C6 | C28 | C26 | -0.3(4) |  | C65 | C79 | C23 | C21 | 142.1(3) |
| C8 | C6 | C28 | C42 | -161.9(2) |  | C65 | C85 | C43 | C112 | 0.8(5) |
| C10 | C14 | C18 | C42 | 1.0(4) |  | C65 | C85 | C43 | C73 | 138.5(3) |
| C10 | C14 | C18 | C52 | 162.0(2) |  | C33 | C110 | C79 | C65 | 0.0(5) |
| C10 | C14 | C34 | S1 | -166.3(2) |  | C33 | C110 | C79 | C23 | 138.4(3) |
| C10 | C14 | C34 | C84 | 3.4(4) |  | C33 | C116 | C99 | C101 | 0.6(5) |
| C10 | C26 | C28 | C6 | -162.2(2) |  | C33 | C116 | C99 | C53 | -138.0(3) |
| C10 | C26 | C28 | C42 | -0.5(4) |  | C33 | C120 | C2 | C65 | -0.6(5) |
| C10 | C36 | C50 | C84 | -0.8(4) |  | C33 | C120 | C2 | C81 | -142.6(3) |
| C10 | C36 | C50 | C98 | 169.4(2) |  | C33 | C120 | C93 | C91 | 142.9(3) |
| C10 | C36 | C60 | C66 | -158.6(2) |  | C33 | C120 | C93 | C101 | -0.5(5) |
| C10 | C36 | C60 | C100 | -0.6(4) |  | C67 | C123 | C63 | C19 | -142.7(3) |
| C12 | C1 | C16 | C24 | -5.0(4) |  | C67 | C123 | C63 | C5 | 0.8(4) |
| C12 | C1 | C16 | C76 | 155.0(2) |  | C67 | C123 | C87 | C35 | 142.4(3) |
| C12 | C1 | C38 | C30 | 0.1(4) |  | C67 | C123 | C87 | C73 | -0.2(4) |
| C12 | C1 | C38 | C44 | -156.0(3) |  | C67 | C85 | C43 | C112 | -138.0(3) |
| C12 | C4 | C26 | C10 | 162.3(2) |  | C67 | C85 | C43 | C73 | -0.3(5) |
| C12 | C4 | C26 | C28 | 0.0(4) |  | C17 | C102 | C83 | C107 | -1.0(5) |
| C12 | C4 | C100 | C24 | -5.5(4) |  | C17 | C13 | C29 | C107 | -0.9(6) |
| C12 | C4 | C100 | C60 | -163.6(2) |  | C69 | C114 | C37 | C9 | 142.4(3) |
| C12 | C8 | C54 | C30 | -0.6(4) |  | C69 | C114 | C37 | C41 | -0.1(3) |
| C12 | C8 | C54 | C40 | 158.5(2) |  | C69 | C114 | C19 | C63 | -137.6(3) |
| C14 | C10 | C26 | C4 | -157.0(2) |  | C69 | C114 | C19 | C35 | 0.5(4) |
| C14 | C10 | C26 | C28 | 5.6(4) |  | C69 | C95 | C103 | C11 | -142.3(3) |
| C14 | C10 | C36 | C50 | 3.1(4) |  | C69 | C95 | C103 | C7 | 0.5(5) |
| C14 | C10 | C36 | C60 | 158.6(2) |  | C69 | C25 | C113 | C7 | -0.1(5) |
| C14 | C18 | C42 | C28 | 4.1(4) |  | C69 | C25 | C113 | C59 | 138.2(4) |
| C14 | C18 | C42 | C32 | 163.7(2) |  | C35 | C122 | C45 | C11 | 143.0(3) |
| C14 | C18 | C52 | S1 | 4.5(3) |  | C35 | C122 | C45 | C89 | 0.0(5) |
| C14 | C18 | C52 | C62 | -163.3(2) |  | C35 | C122 | C95 | C69 | -0.3(5) |
| C14 | C34 | C84 | C50 | -0.8(4) |  | C35 | C122 | C95 | C103 | -142.6(3) |
| C16 | C1 | C12 | C4 | 4.5(4) |  | C71 | C82 | C86 | C22 | 98.5(3) |
| C16 | C1 | C12 | C8 | -157.9(2) |  | C71 | C82 | C86 | C64 | -92.9(4) |
| C16 | C1 | C38 | C30 | 159.3(2) |  | C9 | C108 | C77 | C118 | 137.8(3) |
| C16 | C1 | C38 | C44 | 3.2(4) |  | C9 | C108 | C77 | C75 | -0.3(4) |
| C16 | C24 | C90 | S3 | 4.5(3) |  | C9 | C108 | C39 | C96 | -142.9(3) |
| C16 | C24 | C90 | C70 | -164.2(2) |  | C9 | C108 | C39 | C21 | 0.4(3) |
| C16 | C24 | C100 | C4 | 5.2(4) |  | C9 | C37 | C41 | C75 | 0.0(5) |
| C16 | C24 | C100 | C60 | 164.5(2) |  | C9 | C37 | C41 | C25 | -142.9(3) |
| C18 | C14 | C34 | S1 | -4.5(3) |  | C9 | C5 | C21 | C39 | 0.1(3) |
| C18 | C14 | C34 | C84 | 165.2(2) |  | C9 | C5 | C21 | C23 | 142.8(3) |
| C18 | C52 | C62 | C46 | 0.4(4) |  | C73 | C89 | C45 | C122 | 0.1(5) |
| C20 | C6 | C8 | C12 | -162.1(2) |  | C73 | C89 | C45 | C11 | -138.3(3) |
| C20 | C6 | C8 | C54 | -0.4(4) |  | C73 | C89 | C47 | C112 | 0.1(4) |
| C20 | C6 | C28 | C26 | 162.0(2) |  | C73 | C89 | C47 | C49 | 142.9(3) |
| C20 | C6 | C28 | C42 | 0.4(4) |  | C37 | C114 | C69 | C95 | -142.7(3) |
| C20 | C22 | C32 | C42 | -0.6(4) |  | C37 | C114 | C69 | C25 | 0.1(4) |
| C20 | C22 | C32 | C46 | -158.8(2) |  | C37 | C114 | C19 | C63 | 0.4(4) |
| C20 | C22 | C86 | C64 | -1.4(4) |  | C37 | C114 | C19 | C35 | 138.5(3) |
| C20 | C22 | C86 | C82 | 169.0(3) |  | C37 | C9 | C5 | C63 | -0.1(4) |
| C20 | C74 | C80 | S2 | -166.7(2) |  | C37 | C9 | C5 | C21 | -142.8(3) |
| C20 | C74 | C80 | C64 | 2.3(4) |  | C37 | C41 | C25 | C69 | 0.1(4) |
| C22 | C20 | C74 | C40 | -164.4(2) |  | C37 | C41 | C25 | C113 | 142.3(3) |
| C22 | C20 | C74 | C80 | -3.5(4) |  | C75 | C41 | C25 | C69 | -142.7(3) |
| C22 | C32 | C42 | C18 | -158.7(2) |  | C75 | C41 | C25 | C113 | -0.5(5) |
| C22 | C32 | C42 | C28 | 1.0(4) |  | C75 | C3 | C111 | C118 | 0.1(4) |
| C22 | C32 | C46 | C62 | 158.8(2) |  | C75 | C3 | C111 | C53 | -142.8(3) |
| C22 | C32 | C46 | C82 | -11.5(3) |  | C75 | C3 | C59 | C113 | -0.5(5) |
| C24 | C16 | C76 | S3 | -4.8(3) |  | C75 | C3 | C59 | C61 | 137.7(4) |
| C24 | C16 | C76 | C56 | 163.6(2) |  | C19 | C114 | C69 | C95 | -0.6(5) |
| C26 | C4 | C12 | C1 | -162.1(2) |  | C19 | C114 | C69 | C25 | 142.2(3) |
| C26 | C4 | C12 | C8 | 0.1(4) |  | C19 | C114 | C37 | C9 | -0.4(5) |
| C26 | C4 | C100 | C24 | 157.4(2) |  | C19 | C114 | C37 | C41 | -142.9(3) |
| C26 | C4 | C100 | C60 | -0.7(4) |  | C19 | C63 | C5 | C9 | 0.1(4) |
| C26 | C10 | C14 | C18 | -5.9(4) |  | C19 | C63 | C5 | C21 | 137.9(3) |
| C26 | C10 | C14 | C34 | 155.0(2) |  | C19 | C35 | C87 | C123 | 0.2(3) |
| C26 | C10 | C36 | C50 | -156.1(3) |  | C19 | C35 | C87 | C73 | 143.0(3) |
| C26 | C10 | C36 | C60 | -0.7(4) |  | C77 | C108 | C9 | C37 | 0.2(4) |
| C26 | C28 | C42 | C18 | -4.3(4) |  | C77 | C108 | C9 | C5 | -142.6(3) |
| C26 | C28 | C42 | C32 | -162.8(2) |  | C77 | C108 | C39 | C96 | -0.5(4) |
| C28 | C6 | C8 | C12 | 0.3(4) |  | C77 | C108 | C39 | C21 | 142.8(3) |
| C28 | C6 | C8 | C54 | 162.1(2) |  | C77 | C118 | C111 | C3 | -0.2(4) |
| C28 | C6 | C20 | C22 | 0.0(4) |  | C77 | C118 | C111 | C53 | 142.2(3) |
| C28 | C6 | C20 | C74 | -157.8(2) |  | C77 | C75 | C41 | C37 | -0.2(5) |
| C30 | C38 | C44 | C56 | -158.8(2) |  | C77 | C75 | C41 | C25 | 138.3(3) |
| C30 | C38 | C44 | C58 | 12.9(3) |  | C77 | C75 | C3 | C111 | 0.1(4) |
| C32 | C22 | C86 | C64 | -159.4(3) |  | C77 | C75 | C3 | C59 | -142.2(3) |
| C32 | C22 | C86 | C82 | 11.1(3) |  | C39 | C96 | C106 | C116 | 142.3(3) |
| C32 | C46 | C62 | C52 | 0.2(4) |  | C39 | C96 | C106 | C118 | -0.3(4) |
| C32 | C46 | C82 | C86 | 16.9(3) |  | C39 | C96 | C110 | C33 | -142.3(3) |
| C32 | C46 | C82 | C94 | 139.4(2) |  | C39 | C96 | C110 | C79 | 0.1(4) |
| C32 | C46 | C82 | C71 | -98.3(3) |  | C39 | C108 | C9 | C37 | 142.4(3) |
| C34 | S1 | C52 | C18 | -6.0(2) |  | C39 | C108 | C9 | C5 | -0.3(3) |
| C34 | S1 | C52 | C62 | 160.5(3) |  | C39 | C108 | C77 | C118 | 0.0(4) |
| C34 | C14 | C18 | C42 | -161.0(2) |  | C39 | C108 | C77 | C75 | -138.1(3) |
| C34 | C14 | C18 | C52 | 0.0(3) |  | C39 | C21 | C23 | C67 | 138.4(3) |
| C36 | C10 | C14 | C18 | -165.2(2) |  | C39 | C21 | C23 | C79 | 0.3(4) |
| C36 | C10 | C14 | C34 | -4.3(4) |  | C79 | C110 | C33 | C116 | -142.3(3) |
| C36 | C10 | C26 | C4 | 1.2(4) |  | C79 | C110 | C33 | C120 | 0.0(5) |
| C36 | C10 | C26 | C28 | 163.7(2) |  | C79 | C65 | C85 | C67 | 0.0(4) |
| C36 | C50 | C84 | C34 | -0.4(4) |  | C79 | C65 | C85 | C43 | -143.1(3) |
| C36 | C50 | C98 | C66 | -17.6(3) |  | C5 | C63 | C19 | C114 | -0.2(4) |
| C36 | C50 | C98 | C88 | -140.5(2) |  | C5 | C63 | C19 | C35 | -142.6(3) |
| C36 | C50 | C98 | C31 | 97.3(3) |  | C5 | C9 | C37 | C114 | 0.3(5) |
| C36 | C60 | C66 | C70 | 157.9(2) |  | C5 | C9 | C37 | C41 | 138.3(3) |
| C36 | C60 | C66 | C98 | -11.9(3) |  | C5 | C21 | C23 | C67 | -0.1(4) |
| C36 | C60 | C100 | C4 | 1.2(4) |  | C5 | C21 | C23 | C79 | -138.2(3) |
| C36 | C60 | C100 | C24 | -158.1(2) |  | C81 | C112 | C43 | C73 | -143.2(3) |
| C38 | C1 | C12 | C4 | 162.5(2) |  | C81 | C112 | C43 | C85 | -1.1(5) |
| C38 | C1 | C12 | C8 | 0.0(4) |  | C81 | C112 | C47 | C89 | 143.3(3) |
| C38 | C1 | C16 | C24 | -164.1(2) |  | C81 | C112 | C47 | C49 | 0.8(5) |
| C38 | C1 | C16 | C76 | -4.1(4) |  | C81 | C2 | C65 | C79 | 138.1(3) |
| C38 | C30 | C54 | C8 | 0.8(4) |  | C81 | C2 | C65 | C85 | -0.3(5) |
| C38 | C30 | C54 | C40 | -159.6(2) |  | C81 | C91 | C93 | C120 | -0.4(4) |
| C38 | C30 | C72 | C48 | 160.3(2) |  | C81 | C91 | C93 | C101 | 143.2(3) |
| C38 | C30 | C72 | C58 | -11.3(3) |  | C81 | C91 | C109 | C49 | 0.0(5) |
| C38 | C44 | C56 | C76 | -1.3(4) |  | C81 | C91 | C109 | C117 | -138.2(3) |
| C38 | C44 | C58 | C72 | -18.2(3) |  | C41 | C75 | C77 | C108 | 0.3(5) |
| C38 | C44 | C58 | C78 | -142.0(2) |  | C41 | C75 | C77 | C118 | -142.5(3) |
| C38 | C44 | C58 | C92 | 96.0(3) |  | C41 | C75 | C3 | C111 | 142.6(3) |
| C40 | C74 | C80 | S2 | -4.9(3) |  | C41 | C75 | C3 | C59 | 0.2(5) |
| C40 | C74 | C80 | C64 | 164.1(2) |  | C41 | C25 | C113 | C7 | -138.1(3) |
| C42 | C18 | C52 | S1 | 165.1(2) |  | C41 | C25 | C113 | C59 | 0.2(5) |
| C42 | C18 | C52 | C62 | -2.7(4) |  | C83 | C102 | C17 | Cl2 | -178.5(2) |
| C42 | C32 | C46 | C62 | 1.5(4) |  | C83 | C102 | C17 | C13 | 1.6(5) |
| C42 | C32 | C46 | C82 | -168.8(2) |  | C83 | C107 | C29 | C13 | 1.4(6) |
| C44 | C56 | C76 | S3 | 166.0(2) |  | C85 | C65 | C79 | C110 | 142.7(3) |
| C44 | C56 | C76 | C16 | 0.3(4) |  | C85 | C65 | C79 | C23 | 0.1(3) |
| C44 | C58 | C72 | C30 | 17.7(3) |  | C85 | C67 | C23 | C79 | 0.2(3) |
| C44 | C58 | C72 | C48 | -152.1(3) |  | C85 | C67 | C23 | C21 | -142.7(3) |
| C46 | C32 | C42 | C18 | -3.6(4) |  | C43 | C112 | C81 | C2 | 0.7(5) |
| C46 | C32 | C42 | C28 | 156.1(3) |  | C43 | C112 | C81 | C91 | 138.5(3) |
| C46 | C82 | C86 | C22 | -16.7(3) |  | C43 | C112 | C47 | C89 | -0.3(4) |
| C46 | C82 | C86 | C64 | 151.9(3) |  | C43 | C112 | C47 | C49 | -142.9(3) |
| C50 | C36 | C60 | C66 | 0.0(3) |  | C43 | C73 | C87 | C123 | 0.1(5) |
| C50 | C36 | C60 | C100 | 158.1(2) |  | C43 | C73 | C87 | C35 | -138.3(3) |
| C52 | S1 | C34 | C14 | 5.9(2) |  | C43 | C73 | C89 | C45 | 142.5(3) |
| C52 | S1 | C34 | C84 | -162.6(3) |  | C43 | C73 | C89 | C47 | 0.2(3) |
| C52 | C18 | C42 | C28 | -155.4(3) |  | C87 | C123 | C63 | C19 | -0.3(3) |
| C52 | C18 | C42 | C32 | 4.1(4) |  | C87 | C123 | C63 | C5 | 143.3(3) |
| C54 | C8 | C12 | C1 | 0.2(4) |  | C87 | C123 | C67 | C85 | 0.1(4) |
| C54 | C8 | C12 | C4 | -161.9(2) |  | C87 | C123 | C67 | C23 | -137.9(3) |
| C54 | C30 | C38 | C1 | -0.5(4) |  | C87 | C35 | C19 | C114 | -143.0(3) |
| C54 | C30 | C38 | C44 | 158.7(2) |  | C87 | C35 | C19 | C63 | -0.4(3) |
| C54 | C30 | C72 | C48 | 1.6(4) |  | C87 | C73 | C43 | C112 | 142.3(3) |
| C54 | C30 | C72 | C58 | -169.9(2) |  | C87 | C73 | C43 | C85 | 0.1(5) |
| C54 | C40 | C68 | S2 | 164.8(2) |  | C87 | C73 | C89 | C45 | 0.0(5) |
| C54 | C40 | C68 | C48 | -2.9(4) |  | C87 | C73 | C89 | C47 | -142.4(3) |
| C54 | C40 | C74 | C20 | 1.6(4) |  | C11 | C97 | C49 | C47 | -0.1(5) |
| C54 | C40 | C74 | C80 | -160.5(2) |  | C11 | C97 | C49 | C109 | 143.0(3) |
| C56 | C44 | C58 | C72 | 151.5(3) |  | C11 | C97 | C121 | C117 | -142.7(3) |
| C56 | C44 | C58 | C78 | 27.8(4) |  | C11 | C97 | C121 | C15 | 0.4(5) |
| C56 | C44 | C58 | C92 | -94.2(3) |  | C11 | C103 | C7 | C113 | 137.9(4) |
| C58 | C44 | C56 | C76 | -170.0(3) |  | C11 | C103 | C7 | C15 | 0.4(5) |
| C60 | C36 | C50 | C84 | -158.4(2) |  | C89 | C73 | C43 | C112 | -0.4(3) |
| C60 | C36 | C50 | C98 | 11.9(3) |  | C89 | C73 | C43 | C85 | -142.5(3) |
| C60 | C66 | C70 | C90 | 0.4(4) |  | C89 | C73 | C87 | C123 | 138.3(3) |
| C60 | C66 | C98 | C50 | 17.6(3) |  | C89 | C73 | C87 | C35 | -0.2(4) |
| C60 | C66 | C98 | C88 | 140.6(2) |  | C89 | C47 | C49 | C97 | -0.1(5) |
| C60 | C66 | C98 | C31 | -97.5(3) |  | C89 | C47 | C49 | C109 | -138.6(3) |
| C62 | C46 | C82 | C86 | -151.5(3) |  | C45 | C122 | C35 | C19 | -138.3(3) |
| C62 | C46 | C82 | C94 | -29.0(4) |  | C45 | C122 | C35 | C87 | -0.2(4) |
| C62 | C46 | C82 | C71 | 93.3(4) |  | C45 | C122 | C95 | C69 | 142.4(3) |
| C66 | C60 | C100 | C4 | 156.0(3) |  | C45 | C122 | C95 | C103 | 0.1(3) |
| C66 | C60 | C100 | C24 | -3.3(4) |  | C45 | C11 | C97 | C49 | 0.0(5) |
| C66 | C70 | C90 | S3 | -165.5(2) |  | C45 | C11 | C97 | C121 | 137.7(4) |
| C66 | C70 | C90 | C24 | 0.5(4) |  | C45 | C11 | C103 | C95 | 0.2(4) |
| C68 | S2 | C80 | C64 | -161.7(3) |  | C45 | C11 | C103 | C7 | -142.7(3) |
| C68 | S2 | C80 | C74 | 6.2(2) |  | C45 | C89 | C47 | C112 | -142.5(3) |
| C68 | C40 | C54 | C8 | -156.8(3) |  | C45 | C89 | C47 | C49 | 0.3(4) |
| C68 | C40 | C54 | C30 | 3.3(4) |  | C91 | C93 | C101 | C99 | -138.3(3) |
| C68 | C40 | C74 | C20 | 162.7(2) |  | C91 | C93 | C101 | C27 | -0.9(5) |
| C68 | C40 | C74 | C80 | 0.5(4) |  | C91 | C109 | C117 | C27 | -0.1(5) |
| C68 | C48 | C72 | C30 | -0.9(4) |  | C91 | C109 | C117 | C121 | 142.8(3) |
| C68 | C48 | C72 | C58 | 167.9(3) |  | C23 | C67 | C85 | C65 | -0.1(3) |
| C70 | C66 | C98 | C50 | -150.2(3) |  | C23 | C67 | C85 | C43 | 143.0(3) |
| C70 | C66 | C98 | C88 | -27.2(4) |  | C93 | C120 | C2 | C65 | 141.9(3) |
| C70 | C66 | C98 | C31 | 94.6(4) |  | C93 | C120 | C2 | C81 | -0.2(3) |
| C72 | C30 | C38 | C1 | -160.3(2) |  | C93 | C120 | C33 | C110 | -137.8(3) |
| C72 | C30 | C38 | C44 | -1.1(3) |  | C93 | C120 | C33 | C116 | 0.3(4) |
| C72 | C30 | C54 | C8 | 157.6(3) |  | C93 | C91 | C109 | C49 | 137.7(4) |
| C72 | C30 | C54 | C40 | -2.8(4) |  | C93 | C91 | C109 | C117 | -0.6(5) |
| C72 | C48 | C68 | S2 | -163.4(2) |  | C93 | C101 | C27 | C57 | -141.9(3) |
| C72 | C48 | C68 | C40 | 1.5(4) |  | C93 | C101 | C27 | C117 | 0.2(5) |
| C74 | C20 | C22 | C32 | 159.2(2) |  | C47 | C112 | C81 | C2 | -138.6(3) |
| C74 | C20 | C22 | C86 | 3.1(4) |  | C47 | C112 | C81 | C91 | -0.8(4) |
| C74 | C40 | C54 | C8 | 2.8(4) |  | C47 | C112 | C43 | C73 | 0.4(3) |
| C74 | C40 | C54 | C30 | 163.0(2) |  | C47 | C112 | C43 | C85 | 142.5(3) |
| C74 | C40 | C68 | S2 | 4.2(3) |  | C47 | C89 | C45 | C122 | 138.0(3) |
| C74 | C40 | C68 | C48 | -163.5(3) |  | C47 | C89 | C45 | C11 | -0.4(4) |
| C76 | S3 | C90 | C24 | -6.1(2) |  | C47 | C49 | C109 | C91 | -0.1(5) |
| C76 | S3 | C90 | C70 | 161.4(3) |  | C47 | C49 | C109 | C117 | 142.7(3) |
| C76 | C16 | C24 | C90 | 0.2(3) |  | C95 | C122 | C35 | C19 | 0.2(4) |
| C76 | C16 | C24 | C100 | -161.4(2) |  | C95 | C122 | C35 | C87 | 138.3(3) |
| C78 | C58 | C72 | C30 | 141.4(2) |  | C95 | C122 | C45 | C11 | 0.0(3) |
| C78 | C58 | C72 | C48 | -28.4(4) |  | C95 | C122 | C45 | C89 | -143.0(3) |
| C80 | S2 | C68 | C40 | -5.9(2) |  | C95 | C69 | C25 | C41 | 142.2(3) |
| C80 | S2 | C68 | C48 | 160.5(3) |  | C95 | C69 | C25 | C113 | -0.2(5) |
| C80 | C64 | C86 | C22 | -0.1(4) |  | C95 | C103 | C7 | C113 | -0.8(5) |
| C80 | C64 | C86 | C82 | -167.6(3) |  | C95 | C103 | C7 | C15 | -138.2(4) |
| C82 | C46 | C62 | C52 | 167.5(3) |  | C3 | C75 | C77 | C108 | 142.6(3) |
| C84 | C50 | C98 | C66 | 150.8(3) |  | C3 | C75 | C77 | C118 | -0.3(3) |
| C84 | C50 | C98 | C88 | 27.8(4) |  | C3 | C75 | C41 | C37 | -138.2(3) |
| C84 | C50 | C98 | C31 | -94.3(4) |  | C3 | C75 | C41 | C25 | 0.3(5) |
| C86 | C22 | C32 | C42 | 158.5(2) |  | C3 | C59 | C61 | C57 | 0.4(5) |
| C86 | C22 | C32 | C46 | 0.3(3) |  | C3 | C59 | C61 | C15 | -142.1(3) |
| C86 | C64 | C80 | S2 | 166.1(2) |  | C97 | C11 | C45 | C122 | -142.6(3) |
| C86 | C64 | C80 | C74 | -0.3(4) |  | C97 | C11 | C45 | C89 | 0.2(5) |
| C90 | S3 | C76 | C16 | 6.2(2) |  | C97 | C11 | C103 | C95 | 142.4(3) |
| C90 | S3 | C76 | C56 | -160.9(3) |  | C97 | C11 | C103 | C7 | -0.4(5) |
| C90 | C24 | C100 | C4 | -155.1(3) |  | C97 | C49 | C109 | C91 | -143.3(3) |
| C90 | C24 | C100 | C60 | 4.2(4) |  | C97 | C49 | C109 | C117 | -0.5(4) |
| C92 | C58 | C72 | C30 | -96.1(3) |  | C49 | C97 | C121 | C117 | -0.6(4) |
| C92 | C58 | C72 | C48 | 94.0(4) |  | C49 | C97 | C121 | C15 | 142.6(3) |
| C94 | C82 | C86 | C22 | -139.2(3) |  | C49 | C109 | C117 | C27 | -142.8(3) |
| C94 | C82 | C86 | C64 | 29.4(4) |  | C49 | C109 | C117 | C121 | 0.2(4) |
| C96 | C106 | C116 | C33 | 0.3(3) |  | C99 | C116 | C33 | C110 | 142.3(3) |
| C96 | C106 | C116 | C99 | -142.0(3) |  | C99 | C116 | C33 | C120 | -0.3(5) |
| C96 | C106 | C118 | C77 | -0.2(4) |  | C99 | C101 | C27 | C57 | -0.1(4) |
| C96 | C106 | C118 | C111 | 137.9(3) |  | C99 | C101 | C27 | C117 | 142.1(3) |
| C96 | C110 | C33 | C116 | -0.1(3) |  | C99 | C53 | C111 | C118 | 0.4(5) |
| C96 | C110 | C33 | C120 | 142.2(3) |  | C99 | C53 | C111 | C3 | 138.7(3) |
| C96 | C110 | C79 | C65 | -138.1(3) |  | C99 | C53 | C57 | C27 | 0.0(4) |
| C96 | C110 | C79 | C23 | 0.4(4) |  | C99 | C53 | C57 | C61 | -142.8(3) |
| C96 | C39 | C21 | C5 | 143.1(3) |  | C25 | C69 | C95 | C122 | -137.7(3) |
| C96 | C39 | C21 | C23 | 0.2(4) |  | C25 | C69 | C95 | C103 | 0.0(5) |
| C98 | C50 | C84 | C34 | -167.7(3) |  | C25 | C113 | C59 | C3 | 0.3(5) |
| C98 | C66 | C70 | C90 | 167.1(3) |  | C25 | C113 | C59 | C61 | -142.7(3) |
| C100 | C4 | C12 | C1 | 0.7(4) |  | C101 | C99 | C53 | C111 | -143.3(3) |
| C100 | C4 | C12 | C8 | 162.9(2) |  | C101 | C99 | C53 | C57 | 0.0(3) |
| C100 | C4 | C26 | C10 | -0.5(4) |  | C101 | C27 | C57 | C53 | 0.1(4) |
| C100 | C4 | C26 | C28 | -162.9(2) |  | C101 | C27 | C57 | C61 | 142.7(3) |
| C100 | C24 | C90 | S3 | 165.6(2) |  | C101 | C27 | C117 | C109 | 0.3(5) |
| C100 | C24 | C90 | C70 | -3.0(4) |  | C101 | C27 | C117 | C121 | -138.0(4) |
| C100 | C60 | C66 | C70 | 1.1(4) |  | C51 | C104 | C105 | Cl3 | 177.6(3) |
| C100 | C60 | C66 | C98 | -168.7(2) |  | C51 | C104 | C105 | C119 | 0.1(6) |
| C102 | C17 | C13 | C29 | -0.7(5) |  | C103 | C11 | C45 | C122 | -0.1(3) |
| C102 | C83 | C107 | C29 | -0.5(5) |  | C103 | C11 | C45 | C89 | 142.8(3) |
| C104 | C51 | C115 | C55 | 0.9(6) |  | C103 | C11 | C97 | C49 | -137.8(3) |
| C104 | C105 | C119 | C55 | 0.7(6) |  | C103 | C11 | C97 | C121 | 0.0(5) |
| C106 | C96 | C110 | C33 | 0.3(3) |  | C103 | C7 | C113 | C25 | 0.5(5) |
| C106 | C96 | C110 | C79 | 142.7(3) |  | C103 | C7 | C113 | C59 | -142.2(3) |
| C106 | C96 | C39 | C108 | 0.6(4) |  | C103 | C7 | C15 | C121 | 0.0(5) |
| C106 | C96 | C39 | C21 | -138.4(3) |  | C103 | C7 | C15 | C61 | 142.7(3) |
| C106 | C116 | C33 | C110 | -0.1(3) |  | C105 | C104 | C51 | C115 | -0.9(6) |
| C106 | C116 | C33 | C120 | -142.7(3) |  | C53 | C99 | C101 | C93 | 142.2(3) |
| C106 | C116 | C99 | C101 | 138.3(3) |  | C53 | C99 | C101 | C27 | 0.1(4) |
| C106 | C116 | C99 | C53 | -0.3(4) |  | C53 | C57 | C61 | C59 | -0.2(5) |
| C106 | C118 | C77 | C108 | 0.3(5) |  | C53 | C57 | C61 | C15 | 137.6(4) |
| C106 | C118 | C77 | C75 | 142.8(3) |  | C27 | C57 | C61 | C59 | -138.5(4) |
| C106 | C118 | C111 | C3 | -142.5(3) |  | C27 | C57 | C61 | C15 | -0.7(5) |
| C106 | C118 | C111 | C53 | -0.1(5) |  | C27 | C117 | C121 | C97 | 142.9(3) |
| C108 | C9 | C37 | C114 | -138.0(3) |  | C27 | C117 | C121 | C15 | -0.4(5) |
| C108 | C9 | C37 | C41 | 0.0(4) |  | C109 | C91 | C93 | C120 | -142.5(3) |
| C108 | C9 | C5 | C63 | 142.8(3) |  | C109 | C91 | C93 | C101 | 1.1(5) |
| C108 | C9 | C5 | C21 | 0.2(3) |  | C109 | C117 | C121 | C97 | 0.2(4) |
| C108 | C39 | C21 | C5 | -0.3(3) |  | C109 | C117 | C121 | C15 | -143.1(3) |
| C108 | C39 | C21 | C23 | -143.1(3) |  | C111 | C118 | C77 | C108 | -142.3(3) |
| C110 | C96 | C106 | C116 | -0.3(3) |  | C111 | C118 | C77 | C75 | 0.3(3) |
| C110 | C96 | C106 | C118 | -143.0(3) |  | C111 | C3 | C59 | C113 | -138.4(4) |
| C110 | C96 | C39 | C108 | 138.5(3) |  | C111 | C3 | C59 | C61 | -0.2(5) |
| C110 | C96 | C39 | C21 | -0.4(4) |  | C111 | C53 | C57 | C27 | 142.6(3) |
| C110 | C79 | C23 | C67 | -142.9(3) |  | C111 | C53 | C57 | C61 | -0.1(5) |
| C110 | C79 | C23 | C21 | -0.6(4) |  | C7 | C113 | C59 | C3 | 143.1(3) |
| C112 | C81 | C91 | C93 | -141.8(3) |  | C7 | C113 | C59 | C61 | 0.1(4) |
| C112 | C81 | C91 | C109 | 0.4(5) |  | C7 | C15 | C121 | C97 | -0.4(5) |
| C112 | C47 | C49 | C97 | 138.2(3) |  | C7 | C15 | C121 | C117 | 138.7(4) |
| C112 | C47 | C49 | C109 | -0.3(5) |  | C7 | C15 | C61 | C57 | -142.7(3) |
| C114 | C69 | C95 | C122 | 0.5(5) |  | C7 | C15 | C61 | C59 | -0.7(4) |
| C114 | C69 | C95 | C103 | 138.2(3) |  | C113 | C7 | C15 | C121 | -141.9(3) |
| C114 | C69 | C25 | C41 | -0.1(4) |  | C113 | C7 | C15 | C61 | 0.8(4) |
| C114 | C69 | C25 | C113 | -142.6(3) |  | C113 | C59 | C61 | C57 | 142.9(3) |
| C114 | C37 | C41 | C75 | 142.9(3) |  | C113 | C59 | C61 | C15 | 0.4(4) |
| C114 | C37 | C41 | C25 | 0.0(4) |  | C57 | C53 | C111 | C118 | -138.1(3) |
| C116 | C106 | C118 | C77 | -138.5(3) |  | C57 | C53 | C111 | C3 | 0.3(5) |
| C116 | C106 | C118 | C111 | -0.5(4) |  | C57 | C27 | C117 | C109 | 138.3(4) |
| C116 | C99 | C101 | C93 | -0.8(5) |  | C57 | C27 | C117 | C121 | 0.0(5) |
| C116 | C99 | C101 | C27 | -142.9(3) |  | C115 | C55 | C119 | C105 | -0.7(6) |
| C116 | C99 | C53 | C111 | -0.2(5) |  | C117 | C27 | C57 | C53 | -142.1(3) |
| C116 | C99 | C53 | C57 | 143.1(3) |  | C117 | C27 | C57 | C61 | 0.6(5) |
| C118 | C106 | C116 | C33 | 142.9(3) |  | C59 | C3 | C111 | C118 | 142.8(3) |
| C118 | C106 | C116 | C99 | 0.7(4) |  | C59 | C3 | C111 | C53 | -0.1(5) |
| C120 | C2 | C65 | C79 | 0.6(5) |  | C119 | C55 | C115 | C51 | -0.1(6) |
| C120 | C2 | C65 | C85 | -137.9(3) |  | C15 | C7 | C113 | C25 | 142.2(3) |
| C120 | C2 | C81 | C112 | 142.0(3) |  | C15 | C7 | C113 | C59 | -0.6(4) |
| C120 | C2 | C81 | C91 | -0.1(4) |  | C121 | C97 | C49 | C47 | -142.4(3) |
| C120 | C93 | C101 | C99 | 0.7(5) |  | C121 | C97 | C49 | C109 | 0.6(4) |
| C120 | C93 | C101 | C27 | 138.1(3) |  | C121 | C15 | C61 | C57 | 0.3(5) |
| C122 | C35 | C19 | C114 | -0.2(4) |  | C121 | C15 | C61 | C59 | 142.3(3) |
| C122 | C35 | C19 | C63 | 142.4(3) |  | C61 | C15 | C121 | C97 | -138.9(4) |
| C122 | C35 | C87 | C123 | -142.6(3) |  | C61 | C15 | C121 | C117 | 0.2(5) |

Table 7 Hydrogen Atom Coordinates (Å×104) and Isotropic Displacement Parameters (Å2×103) for s.

| Atom | *x* | *y* | *z* | U(eq) |
| --- | --- | --- | --- | --- |
| H48 | 6356.97 | 14424.87 | 3766.86 | 30 |
| H56 | 4504.52 | 11044.93 | 5324.89 | 26 |
| H62 | -335.02 | 11300.66 | 281 | 28 |
| H64 | 3848.96 | 14492.24 | 1128.54 | 30 |
| H70 | 21.6 | 7678.14 | 4450.35 | 30 |
| H78A | 6407.13 | 12287.5 | 5062.59 | 40 |
| H78B | 6942.93 | 13242.9 | 4644.9 | 40 |
| H78C | 6022.26 | 12178.21 | 4309 | 40 |
| H84 | -2306.77 | 7786.09 | 1994.83 | 28 |
| H88A | -1355.68 | 6408.28 | 3504.84 | 44 |
| H88B | -732.17 | 7052.7 | 2967.33 | 44 |
| H88C | -2015.09 | 6429.85 | 2820.76 | 44 |
| H92A | 5242.48 | 14233.27 | 5470.73 | 41 |
| H92B | 6465.1 | 14515.44 | 5332.19 | 41 |
| H92C | 5969.94 | 13574.21 | 5768.14 | 41 |
| H94A | 2622.81 | 13357.12 | 102.75 | 45 |
| H94B | 1445.5 | 12509.48 | -158.08 | 45 |
| H94C | 2204.31 | 12251.83 | 416.76 | 45 |
| H31A | -3287.95 | 7069.36 | 3338.47 | 46 |
| H31B | -2831.01 | 8115.85 | 3823.49 | 46 |
| H31C | -2634.73 | 7049.63 | 4024.85 | 46 |
| H71A | 756.67 | 14475.23 | 680.9 | 49 |
| H71B | 562.58 | 13833.69 | 0.29 | 49 |
| H71C | 1714.95 | 14727.2 | 256.11 | 49 |
| H83 | 6873.29 | 12672.15 | 613.86 | 43 |
| H51 | -821.75 | 2531.67 | 4487.39 | 54 |
| H13 | 8471.18 | 16090.63 | 1639.23 | 56 |
| H107 | 8622.53 | 13192.88 | 1185.27 | 55 |
| H55 | -1974.48 | 3448.62 | 2843.54 | 60 |
| H115 | -2137.49 | 2200.03 | 3594.21 | 60 |
| H29 | 9428.27 | 14921.56 | 1679.4 | 65 |
| H119 | -497.62 | 5025.52 | 2995.71 | 67 |

Experimental

Single crystals of C123H32Cl4S3
[s]
were
[].
A suitable crystal was selected and
[]
on a
Bruker APEX-II CCD
diffractometer. The crystal was kept at 152.00 K during data collection.
Using Olex2 [1], the structure was solved with the
SHELXT
[2] structure solution program using
Intrinsic Phasing
and refined with the
SHELXL
[3] refinement package using
Least Squares
minimisation.

1. Dolomanov, O.V., Bourhis, L.J., Gildea, R.J, Howard, J.A.K. & Puschmann, H.
   (2009), J. Appl. Cryst. 42, 339-341.
2. Sheldrick, G.M. (2015). Acta Cryst. A71, 3-8.
3. Sheldrick, G.M. (2015). Acta Cryst. C71, 3-8.

Crystal structure determination of
[s]

**Crystal Data**
for C123H32Cl4S3 (*M*=1747.46 g/mol):
triclinic, space group P-1 (no. 2),
*a* = 13.2216(10) Å, *b* = 13.4167(10) Å, *c* = 21.2250(16) Å, *α* = 92.016(3)°, *β* = 97.445(3)°, *γ* = 109.711(3)°,
*V*= 3502.0(5) Å3,
*Z* = 2,
*T* = 152.00 K,
μ(CuKα) = 2.910 mm-1,
*Dcalc* = 1.657 g/cm3,
104498 reflections measured (4.214° ≤ 2Θ ≤ 136.566°),
12460 unique (*R*int = 0.0550, Rsigma = 0.0254) which were used in all calculations.
The final *R*1 was 0.0500
(I > 2σ(I)) and *wR*2 was 0.1166 (all data).

Refinement model description

Number of restraints - 0,
number of constraints - unknown.

Details:

```
1. Fixed Uiso
```

This report has been created with Olex2, compiled on
2023.03.06 svn.rbb2c1857 for OlexSys. Please
let us know
if there are any errors or if you would like to have additional features.
