# Supplementary material for: Trichalcogenasupersumanenes and its concave-convex supramolecular assembly with fullerenes
Source: Nat Commun. 2023 Jun 10;14:3446. doi: 10.1038/s41467-023-39086-0 (PMC10257710; doi:10.1038/s41467-023-39086-0)

checkCIF/PLATON report

Structure factors have been supplied for datablock(s) z_sq_tw

THIS REPORT IS FOR GUIDANCE ONLY. IF USED AS PART OF A REVIEW PROCEDURE FOR PUBLICATION, IT SHOULD NOT REPLACE THE EXPERTISE OF AN EXPERIENCED CRYSTALLOGRAPHIC REFEREE.

No syntax errors found. CIF dictionary Interpreting this report

Datablock: z_sq_tw

Bond precision: C-C = 0.0070 A Wavelength=1.54178

Cell:	a=20.312(4)	b=33.234(9)	c=27.770(6)
	alpha=90	beta=127.915(13)	gamma=90
Temperature:	293 K		

	Calculated	Reported
Volume	14789(6)	14789(6)
Space group	P 21/c	P 1 21/c 1
Hall group	-P 2ybc	-P 2ybc
Moiety formula	C70, C51 H24 S3 [+ solvent]	C51 H24 S3, C70
Sum formula	C121 H24 S3 [+ solvent]	C121 H24 S3
Mr	1573.58	1573.58
Dx,g cm-3	1.413	1.413
Z	8	8
Mu (mm-1)	1.396	1.396
F000	6384.0	6384.0
F000'	6408.76	
h,k,lmax	24,39,32	24,39,32
Nref	25795	25643
Tmin,Tmax	0.778,0.834	0.768,0.839
Tmin'	0.756	

Correction method= # Reported T Limits: Tmin=0.768 Tmax=0.839
AbsCorr = MULTI-SCAN

Data completeness= 0.994 Theta(max)= 66.049

R(reflections)= 0.1516(15614) wR2(reflections)= 0.3173(25643)

S = 1.047 Npar= 1833

The following ALERTS were generated. Each ALERT has the format
test-name_ALERT_alert-type_alert-level.
Click on the hyperlinks for more details of the test.

Alert level C

```
PLAT082_ALERT_2_C High R1 Value .................................      0.15 Report
PLAT084_ALERT_3_C High wR2 Value (i.e. > 0.25) ..................      0.32 Report
PLAT094_ALERT_2_C Ratio of Maximum / Minimum Residual Density ....      2.29 Report
PLAT230_ALERT_2_C Hirshfeld Test Diff for    C26      --C27     .       5.7 s.u.
PLAT230_ALERT_2_C Hirshfeld Test Diff for    C38      --C39     .       5.1 s.u.
PLAT230_ALERT_2_C Hirshfeld Test Diff for    C39      --C41     .       5.5 s.u.
PLAT230_ALERT_2_C Hirshfeld Test Diff for    C65      --C66     .       6.9 s.u.
PLAT230_ALERT_2_C Hirshfeld Test Diff for    C85      --C100    .       5.3 s.u.
PLAT230_ALERT_2_C Hirshfeld Test Diff for    C86      --C87     .       6.0 s.u.
PLAT230_ALERT_2_C Hirshfeld Test Diff for    C121     --C139    .       6.0 s.u.
PLAT234_ALERT_4_C Large Hirshfeld Difference C9       --C10     .      0.18 Ang.
PLAT234_ALERT_4_C Large Hirshfeld Difference C9       --C66     .      0.20 Ang.
PLAT234_ALERT_4_C Large Hirshfeld Difference C13      --C49     .      0.16 Ang.
PLAT234_ALERT_4_C Large Hirshfeld Difference C15      --C16     .      0.17 Ang.
PLAT234_ALERT_4_C Large Hirshfeld Difference C24      --C25     .      0.18 Ang.
PLAT234_ALERT_4_C Large Hirshfeld Difference C26      --C62     .      0.18 Ang.
PLAT234_ALERT_4_C Large Hirshfeld Difference C30      --C31     .      0.19 Ang.
PLAT234_ALERT_4_C Large Hirshfeld Difference C32      --C33     .      0.16 Ang.
PLAT234_ALERT_4_C Large Hirshfeld Difference C36      --C40     .      0.18 Ang.
PLAT234_ALERT_4_C Large Hirshfeld Difference C37      --C38     .      0.17 Ang.
PLAT234_ALERT_4_C Large Hirshfeld Difference C41      --C45     .      0.16 Ang.
PLAT234_ALERT_4_C Large Hirshfeld Difference C43      --C65     .      0.17 Ang.
PLAT234_ALERT_4_C Large Hirshfeld Difference C46      --C65     .      0.18 Ang.
PLAT234_ALERT_4_C Large Hirshfeld Difference C47      --C64     .      0.19 Ang.
PLAT234_ALERT_4_C Large Hirshfeld Difference C51      --C55     .      0.19 Ang.
PLAT234_ALERT_4_C Large Hirshfeld Difference C72      --C76     .      0.16 Ang.
PLAT234_ALERT_4_C Large Hirshfeld Difference C73      --C82     .      0.18 Ang.
PLAT234_ALERT_4_C Large Hirshfeld Difference C74      --C90     .      0.19 Ang.
PLAT234_ALERT_4_C Large Hirshfeld Difference C77      --C78     .      0.18 Ang.
PLAT234_ALERT_4_C Large Hirshfeld Difference C78      --C138    .      0.18 Ang.
PLAT234_ALERT_4_C Large Hirshfeld Difference C79      --C99     .      0.18 Ang.
PLAT234_ALERT_4_C Large Hirshfeld Difference C89      --C132    .      0.16 Ang.
PLAT234_ALERT_4_C Large Hirshfeld Difference C91      --C92     .      0.18 Ang.
PLAT234_ALERT_4_C Large Hirshfeld Difference C94      --C96     .      0.16 Ang.
PLAT234_ALERT_4_C Large Hirshfeld Difference C108     --C129    .      0.16 Ang.
PLAT234_ALERT_4_C Large Hirshfeld Difference C114     --C115    .      0.17 Ang.
PLAT234_ALERT_4_C Large Hirshfeld Difference C116     --C120    .      0.17 Ang.
PLAT234_ALERT_4_C Large Hirshfeld Difference C118     --C125    .      0.20 Ang.
PLAT234_ALERT_4_C Large Hirshfeld Difference C128     --C129    .      0.16 Ang.
PLAT234_ALERT_4_C Large Hirshfeld Difference C129     --C130    .      0.18 Ang.
PLAT234_ALERT_4_C Large Hirshfeld Difference C139     --C140    .      0.18 Ang.
PLAT234_ALERT_4_C Large Hirshfeld Difference C223     --C231    .      0.16 Ang.
PLAT241_ALERT_2_C High   'MainMol' Ueq as Compared to Neighbors of       C29 Check
PLAT241_ALERT_2_C High   'MainMol' Ueq as Compared to Neighbors of       C79 Check
PLAT241_ALERT_2_C High   'MainMol' Ueq as Compared to Neighbors of       C87 Check
PLAT241_ALERT_2_C High   'MainMol' Ueq as Compared to Neighbors of       C90 Check
PLAT241_ALERT_2_C High   'MainMol' Ueq as Compared to Neighbors of      C129 Check
PLAT242_ALERT_2_C Low    'MainMol' Ueq as Compared to Neighbors of       C74 Check
PLAT242_ALERT_2_C Low    'MainMol' Ueq as Compared to Neighbors of       C78 Check
PLAT242_ALERT_2_C Low    'MainMol' Ueq as Compared to Neighbors of       C86 Check
```

```
PLAT242_ALERT_2_C Low    'MainMol' Ueq as Compared to Neighbors of        C95 Check
PLAT250_ALERT_2_C Large U3/U1 Ratio for Average U(i,j) Tensor ....        2.9 Note
PLAT250_ALERT_2_C Large U3/U1 Ratio for Average U(i,j) Tensor ....        2.1 Note
PLAT260_ALERT_2_C Large Average Ueq of Residue Including        C1      0.103 Check
PLAT260_ALERT_2_C Large Average Ueq of Residue Including       C71      0.119 Check
PLAT340_ALERT_3_C Low Bond Precision on  C-C Bonds ...............    0.00705 Ang.
PLAT368_ALERT_2_C Short  C(sp2)-C(sp2) Bond  C87      - C91      .       1.15 Ang.
PLAT368_ALERT_2_C Short  C(sp2)-C(sp2) Bond  C105     - C107     .       1.22 Ang.
PLAT369_ALERT_2_C Long   C(sp2)-C(sp2) Bond  C27      - C63      .       1.53 Ang.
PLAT369_ALERT_2_C Long   C(sp2)-C(sp2) Bond  C46      - C65      .       1.55 Ang.
PLAT369_ALERT_2_C Long   C(sp2)-C(sp2) Bond  C61      - C62      .       1.54 Ang.
PLAT369_ALERT_2_C Long   C(sp2)-C(sp2) Bond  C131     - C132     .       1.55 Ang.
PLAT369_ALERT_2_C Long   C(sp2)-C(sp2) Bond  C135     - C136     .       1.54 Ang.
PLAT906_ALERT_3_C Large K Value in the Analysis of Variance ......     21.883 Check
PLAT906_ALERT_3_C Large K Value in the Analysis of Variance ......      5.148 Check
PLAT906_ALERT_3_C Large K Value in the Analysis of Variance ......      2.672 Check
PLAT911_ALERT_3_C Missing FCF Refl Between Thmin & STh/L=    0.593        153 Report
PLAT992_ALERT_5_C Repd & Actual _reflns_number_gt Values Differ by         27 Check
```

Alert level G

```
PLAT002_ALERT_2_G Number of Distance or Angle Restraints on AtSite        126 Note
PLAT003_ALERT_2_G Number of Uiso or Uij Restrained non-H Atoms ...        248 Report
PLAT042_ALERT_1_G Calc. and Reported MoietyFormula Strings  Differ     Please Check
PLAT083_ALERT_2_G SHELXL Second Parameter in WGHT  Unusually Large      76.48 Why ?
PLAT128_ALERT_4_G Alternate Setting for Input Space Group  P21/c        P21/n Note
PLAT172_ALERT_4_G The CIF-Embedded .res File Contains DFIX Records         49 Report
PLAT177_ALERT_4_G The CIF-Embedded .res File Contains DELU Records          2 Report
PLAT178_ALERT_4_G The CIF-Embedded .res File Contains SIMU Records          1 Report
PLAT188_ALERT_3_G A Non-default SIMU Restraint Value has been used     0.0300 Report
PLAT192_ALERT_3_G A Non-default DELU Restraint Value for First Par     0.0020 Report
PLAT192_ALERT_3_G A Non-default DELU Restraint Value for SecondPar     0.0010 Report
PLAT192_ALERT_3_G A Non-default DELU Restraint Value for First Par     0.0020 Report
PLAT192_ALERT_3_G A Non-default DELU Restraint Value for SecondPar     0.0010 Report
PLAT199_ALERT_1_G Reported _cell_measurement_temperature ..... (K)        293 Check
PLAT200_ALERT_1_G Reported   _diffrn_ambient_temperature ..... (K)        293 Check
PLAT333_ALERT_2_G Large Aver C6-Ring C-C Dist C1       -C6       .       1.42 Ang.
PLAT333_ALERT_2_G Large Aver C6-Ring C-C Dist C19      -C61      .       1.43 Ang.
PLAT333_ALERT_2_G Large Aver C6-Ring C-C Dist C87      -C91      .       1.42 Ang.
PLAT333_ALERT_2_G Large Aver C6-Ring C-C Dist C92      -C134     .       1.44 Ang.
PLAT333_ALERT_2_G Large Aver C6-Ring C-C Dist C193     -C218     .       1.43 Ang.
PLAT333_ALERT_2_G Large Aver C6-Ring C-C Dist C195     -C215     .       1.42 Ang.
PLAT335_ALERT_2_G Check Large C6 Ring C-C Range C19      -C61            0.17 Ang.
PLAT335_ALERT_2_G Check Large C6 Ring C-C Range C87      -C91            0.36 Ang.
PLAT335_ALERT_2_G Check Large C6 Ring C-C Range C88      -C133           0.19 Ang.
PLAT343_ALERT_2_G Unusual sp?      Angle Range in Main Residue for         C5 Check
PLAT343_ALERT_2_G Unusual sp?      Angle Range in Main Residue for         C7 Check
PLAT343_ALERT_2_G Unusual sp?      Angle Range in Main Residue for         C8 Check
PLAT343_ALERT_2_G Unusual sp?      Angle Range in Main Residue for        C11 Check
PLAT343_ALERT_2_G Unusual sp?      Angle Range in Main Residue for        C17 Check
PLAT343_ALERT_2_G Unusual sp?      Angle Range in Main Residue for        C22 Check
PLAT343_ALERT_2_G Unusual sp?      Angle Range in Main Residue for        C30 Check
PLAT343_ALERT_2_G Unusual sp?      Angle Range in Main Residue for        C35 Check
PLAT343_ALERT_2_G Unusual sp?      Angle Range in Main Residue for        C36 Check
PLAT343_ALERT_2_G Unusual sp?      Angle Range in Main Residue for        C38 Check
PLAT343_ALERT_2_G Unusual sp?      Angle Range in Main Residue for        C39 Check
PLAT343_ALERT_2_G Unusual sp?      Angle Range in Main Residue for        C42 Check
```

```
PLAT343_ALERT_2_G Unusual sp?      Angle Range in Main Residue for        C45 Check
PLAT343_ALERT_2_G Unusual sp?      Angle Range in Main Residue for        C47 Check
PLAT343_ALERT_2_G Unusual sp?      Angle Range in Main Residue for        C49 Check
PLAT343_ALERT_2_G Unusual sp?      Angle Range in Main Residue for        C50 Check
PLAT343_ALERT_2_G Unusual sp?      Angle Range in Main Residue for        C51 Check
PLAT343_ALERT_2_G Unusual sp?      Angle Range in Main Residue for        C58 Check
PLAT343_ALERT_2_G Unusual sp?      Angle Range in Main Residue for        C59 Check
PLAT343_ALERT_2_G Unusual sp?      Angle Range in Main Residue for        C72 Check
PLAT343_ALERT_2_G Unusual sp?      Angle Range in Main Residue for        C74 Check
PLAT343_ALERT_2_G Unusual sp?      Angle Range in Main Residue for        C75 Check
PLAT343_ALERT_2_G Unusual sp?      Angle Range in Main Residue for        C79 Check
PLAT343_ALERT_2_G Unusual sp?      Angle Range in Main Residue for        C82 Check
PLAT343_ALERT_2_G Unusual sp?      Angle Range in Main Residue for        C83 Check
PLAT343_ALERT_2_G Unusual sp?      Angle Range in Main Residue for        C84 Check
PLAT343_ALERT_2_G Unusual sp?      Angle Range in Main Residue for        C85 Check
PLAT343_ALERT_2_G Unusual sp?      Angle Range in Main Residue for        C90 Check
PLAT343_ALERT_2_G Unusual sp?      Angle Range in Main Residue for        C96 Check
PLAT343_ALERT_2_G Unusual sp?      Angle Range in Main Residue for       C102 Check
PLAT343_ALERT_2_G Unusual sp?      Angle Range in Main Residue for       C109 Check
PLAT343_ALERT_2_G Unusual sp?      Angle Range in Main Residue for       C115 Check
PLAT343_ALERT_2_G Unusual sp?      Angle Range in Main Residue for       C117 Check
PLAT343_ALERT_2_G Unusual sp?      Angle Range in Main Residue for       C122 Check
PLAT343_ALERT_2_G Unusual sp?      Angle Range in Main Residue for       C123 Check
PLAT343_ALERT_2_G Unusual sp?      Angle Range in Main Residue for       C125 Check
PLAT343_ALERT_2_G Unusual sp?      Angle Range in Main Residue for       C128 Check
PLAT343_ALERT_2_G Unusual sp?      Angle Range in Main Residue for       C130 Check
PLAT367_ALERT_2_G Long?  C(sp?)-C(sp?) Bond  C47      - C64      .       1.50 Ang.
PLAT367_ALERT_2_G Long?  C(sp?)-C(sp?) Bond  C51      - C66      .       1.66 Ang.
PLAT367_ALERT_2_G Long?  C(sp?)-C(sp?) Bond  C74      - C90      .       1.53 Ang.
PLAT432_ALERT_2_G Short Inter X...Y Contact  C42      ..C208     .       3.19 Ang.
                                            1-x,1-y,1-z  =      3_666 Check
PLAT432_ALERT_2_G Short Inter X...Y Contact  C126     ..C214     .       3.05 Ang.
                                                  x,y,z  =      1_555 Check
PLAT432_ALERT_2_G Short Inter X...Y Contact  C161     ..C161     .       3.17 Ang.
                                              -x,1-y,-z  =      3_565 Check
PLAT606_ALERT_4_G Solvent Accessible VOID(S) in Structure ........          ! Info
PLAT790_ALERT_4_G Centre of Gravity not Within Unit Cell: Resd.  #          3 Note
              C51 H24 S3
PLAT860_ALERT_3_G Number of Least-Squares Restraints ............       12433 Note
PLAT869_ALERT_4_G ALERTS Related to the Use of SQUEEZE  Suppressed          ! Info
PLAT870_ALERT_4_G ALERTS Related to Twinning Effects Suppressed ..          ! Info
PLAT883_ALERT_1_G No Info/Value for _atom_sites_solution_primary .     Please Do !
PLAT909_ALERT_3_G Percentage of I>2sig(I) Data at Theta(Max) Still        35% Note
PLAT941_ALERT_3_G Average HKL Measurement Multiplicity ...........        1.0 Low
PLAT961_ALERT_5_G Dataset Contains no Negative Intensities .......     Please Check
```

---

0 **ALERT level A** = Most likely a serious problem - resolve or explain
0 **ALERT level B** = A potentially serious problem, consider carefully
68 **ALERT level C** = Check. Ensure it is not caused by an omission or oversight
77 **ALERT level G** = General information/check it is not something unexpected

4 ALERT type 1 CIF construction/syntax error, inconsistent or missing data
85 ALERT type 2 Indicator that the structure model may be wrong or deficient
14 ALERT type 3 Indicator that the structure quality may be low
40 ALERT type 4 Improvement, methodology, query or suggestion
2 ALERT type 5 Informative message, check

It is advisable to attempt to resolve as many as possible of the alerts in all categories. Often the minor alerts point to easily fixed oversights, errors and omissions in your CIF or refinement strategy, so attention to these fine details can be worthwhile. In order to resolve some of the more serious problems it may be necessary to carry out additional measurements or structure refinements. However, the purpose of your study may justify the reported deviations and the more serious of these should normally be commented upon in the discussion or experimental section of a paper or in the "special_details" fields of the CIF. checkCIF was carefully designed to identify outliers and unusual parameters, but every test has its limitations and alerts that are not important in a particular case may appear. Conversely, the absence of alerts does not guarantee there are no aspects of the results needing attention. It is up to the individual to critically assess their own results and, if necessary, seek expert advice.

Publication of your CIF in IUCr journals

A basic structural check has been run on your CIF. These basic checks will be run on all CIFs submitted for publication in IUCr journals (*Acta Crystallographica*, *Journal of Applied Crystallography*, *Journal of Synchrotron Radiation*); however, if you intend to submit to *Acta Crystallographica Section C* or *E* or *IUCrData*, you should make sure that full publication checks are run on the final version of your CIF prior to submission.

Publication of your CIF in other journals

Please refer to the *Notes for Authors* of the relevant journal for any special instructions relating to CIF submission.

PLATON version of 28/11/2022; check.def file version of 28/11/2022

Datablock z_sq_tw - ellipsoid plot

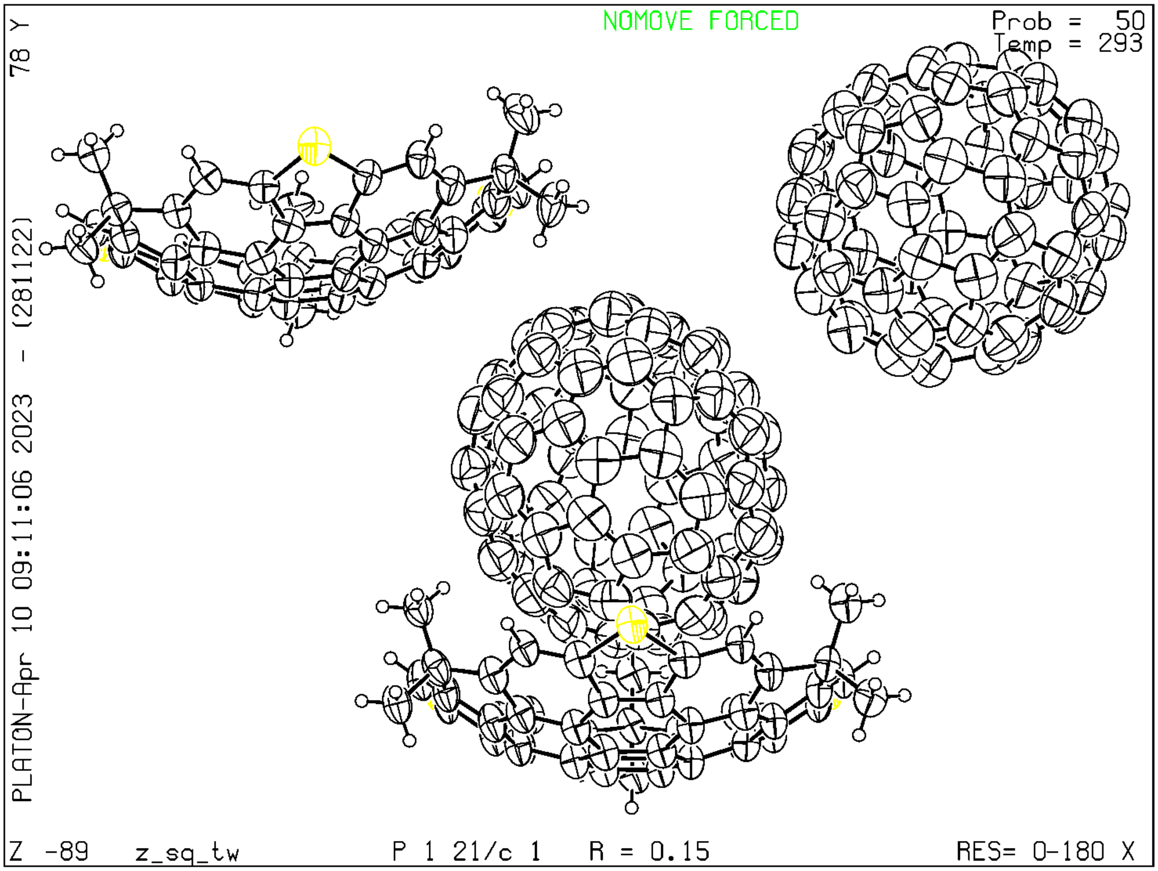

Supplement: Supplementary file 4 — Supplementary Data 1 [file 41467_2023_39086_MOESM4_ESM.zip › 1a-Me@C70/1a-Me@C70_cifreport.pdf]
