# Supplementary material for: Trichalcogenasupersumanenes and its concave-convex supramolecular assembly with fullerenes
Source: Nat Commun. 2023 Jun 10;14:3446. doi: 10.1038/s41467-023-39086-0 (PMC10257710; doi:10.1038/s41467-023-39086-0)
Supplement: Supplementary file 4 — Supplementary Data 1 [file 41467_2023_39086_MOESM4_ESM.zip › 1a-Me@C70/1a-Me@C70_tables.html]

z\_sq\_tw


# z\_sq\_tw

Table 1 Crystal data and structure refinement for z\_sq\_tw.

| Identification code | z\_sq\_tw |
| Empirical formula | C121S3H24 |
| Formula weight | 1573.58 |
| Temperature/K | 293(2) |
| Crystal system | monoclinic |
| Space group | P21/c |
| a/Å | 20.312(4) |
| b/Å | 33.234(9) |
| c/Å | 27.770(6) |
| α/° | 90 |
| β/° | 127.915(13) |
| γ/° | 90 |
| Volume/Å3 | 14789(6) |
| Z | 8 |
| ρcalcg/cm3 | 1.413 |
| μ/mm‑1 | 1.396 |
| F(000) | 6384.0 |
| Crystal size/mm3 | 0.2 × 0.15 × 0.13 |
| Radiation | CuKα (λ = 1.54178) |
| 2Θ range for data collection/° | 4.83 to 132.098 |
| Index ranges | -24 ≤ h ≤ 18, 0 ≤ k ≤ 39, 0 ≤ l ≤ 32 |
| Reflections collected | 25643 |
| Independent reflections | 25643 [Rint = ?, Rsigma = 0.0899] |
| Data/restraints/parameters | 25643/12433/1833 |
| Goodness-of-fit on F2 | 1.047 |
| Final R indexes [I>=2σ (I)] | R1 = 0.1516, wR2 = 0.2990 |
| Final R indexes [all data] | R1 = 0.1916, wR2 = 0.3173 |
| Largest diff. peak/hole / e Å-3 | 1.15/-0.50 |

Table 2 Fractional Atomic Coordinates (×104) and Equivalent Isotropic Displacement Parameters (Å2×103) for z\_sq\_tw. Ueq is defined as 1/3 of the trace of the orthogonalised UIJ tensor.

| Atom | *x* | *y* | *z* | U(eq) |
| --- | --- | --- | --- | --- |
| S1 | 1680.9(11) | 5337.5(6) | 1274.2(11) | 60.2(5) |
| S2 | -2423.5(15) | 3907.4(6) | 1273.2(13) | 72.4(7) |
| S3 | -2345.8(12) | 6796.3(6) | 1352.0(12) | 64.6(6) |
| S4 | 6882.8(12) | 2922.5(7) | 6541.5(12) | 64.6(6) |
| S5 | 2590.5(14) | 1396.6(6) | 6188.8(13) | 70.7(7) |
| S6 | 2874.1(13) | 4262.6(8) | 6734.0(13) | 80.5(7) |
| C1 | 2434.3(10) | 8035.8(5) | 6450.0(10) | 80(2) |
| C2 | 2459.5(10) | 7616.0(5) | 6530.7(9) | 80(2) |
| C3 | 3153.2(11) | 7516.8(5) | 7159.0(9) | 76(2) |
| C4 | 3565.5(12) | 7875.0(6) | 7453.4(9) | 90(2) |
| C5 | 3089.9(11) | 8206.5(6) | 7030.7(10) | 103(3) |
| C6 | 2157.0(10) | 8192.2(5) | 5894.7(9) | 92(2) |
| C7 | 2585.5(11) | 8546.6(5) | 5918.9(10) | 96(3) |
| C8 | 2657.3(11) | 8528.3(5) | 5448.0(9) | 100(3) |
| C9 | 2278.0(10) | 8166.9(4) | 5111.6(8) | 119(3) |
| C10 | 1930.6(9) | 7955.1(4) | 5393.7(8) | 106(3) |
| C11 | 3486.8(11) | 8524.9(5) | 7044.1(10) | 97(3) |
| C12 | 3248.8(11) | 8689.5(5) | 6488.6(10) | 99(3) |
| C13 | 3978.0(12) | 8827.1(5) | 6580.0(10) | 100(3) |
| C14 | 4676.5(12) | 8728.9(5) | 7182.1(10) | 97(3) |
| C15 | 4394.1(12) | 8538.7(6) | 7490.4(10) | 100(3) |
| C16 | 4824.7(12) | 8234.8(6) | 7898.4(10) | 103(3) |
| C17 | 4366.4(12) | 7885.0(6) | 7874.6(9) | 87(2) |
| C18 | 4872.5(12) | 7550.2(5) | 8019.7(8) | 102(3) |
| C19 | 5629.9(12) | 7676.6(5) | 8120.9(9) | 102(3) |
| C20 | 5578.4(12) | 8099.6(6) | 8049.7(9) | 90(2) |
| C21 | 5936.4(11) | 8268.2(6) | 7786.3(10) | 88(2) |
| C22 | 5502.1(12) | 8604.1(6) | 7376.4(10) | 100(3) |
| C23 | 5551.8(12) | 8568.4(5) | 6895.1(10) | 101(3) |
| C24 | 6044.4(12) | 8210.7(6) | 7000.6(10) | 91(2) |
| C25 | 6283.5(11) | 8037.3(6) | 7561.9(10) | 101(3) |
| C26 | 6332.8(10) | 7404.6(5) | 7196.4(10) | 111(3) |
| C27 | 6125.1(10) | 7591.8(6) | 6671.6(10) | 103(3) |
| C28 | 5689.2(10) | 7325.1(5) | 6168.1(10) | 106(3) |
| C29 | 5678.3(9) | 6935.8(5) | 6414.1(9) | 123(4) |
| C30 | 6115.0(9) | 6995.6(5) | 7061.2(9) | 102(3) |
| C31 | 5809.0(10) | 6823.5(5) | 7350.7(8) | 114(3) |
| C32 | 5742.3(11) | 7031.9(5) | 7766.0(8) | 107(3) |
| C33 | 4996.9(11) | 6901.6(5) | 7669.0(7) | 102(3) |
| C34 | 4584.7(10) | 6630.8(4) | 7165.4(7) | 113(3) |
| C35 | 5105.1(9) | 6561.8(4) | 6985.1(7) | 109(3) |
| C36 | 4747.8(8) | 6489.7(4) | 6401.8(7) | 126(4) |
| C37 | 3888.1(7) | 6480.9(4) | 5988.2(7) | 118(3) |
| C38 | 3642.5(7) | 6647.8(4) | 5424.8(7) | 105(3) |
| C39 | 4367.1(8) | 6780.6(4) | 5512.3(8) | 114(3) |
| C40 | 5055.5(8) | 6707.7(5) | 6109.9(8) | 110(3) |
| C41 | 4389.8(10) | 7148.0(5) | 5280.8(8) | 98(3) |
| C42 | 3651.5(10) | 7367.8(4) | 4862.6(7) | 114(3) |
| C43 | 3847.1(12) | 7780.2(5) | 4954.4(8) | 100(3) |
| C44 | 4733.1(12) | 7833.4(5) | 5414.8(9) | 102(3) |
| C45 | 5080.2(11) | 7422.3(5) | 5592.8(9) | 103(3) |
| C46 | 3729.6(13) | 8444.9(5) | 5301.7(9) | 102(3) |
| C47 | 4658.3(13) | 8463.2(5) | 5751.9(9) | 99(3) |
| C48 | 4834.0(13) | 8671.1(5) | 6273.2(10) | 101(3) |
| C49 | 4067.7(13) | 8811.9(5) | 6128.9(10) | 89(2) |
| C50 | 3395.5(13) | 8665.9(5) | 5549.2(9) | 101(3) |
| C51 | 2297.1(9) | 7511.8(4) | 4749.4(7) | 135(4) |
| C52 | 2048.5(8) | 7314.4(4) | 5062.2(7) | 115(3) |
| C53 | 2440.8(7) | 6928.7(4) | 5264.5(6) | 108(3) |
| C54 | 2944.0(7) | 6887.3(4) | 5061.5(6) | 104(3) |
| C55 | 2885.4(9) | 7263.6(4) | 4757.5(6) | 109(3) |
| C56 | 2578.8(9) | 6971.1(4) | 6213.9(7) | 91(2) |
| C57 | 3267.2(10) | 6881.2(5) | 6830.1(7) | 90(2) |
| C58 | 3791.4(10) | 6598.5(4) | 6822.8(7) | 103(3) |
| C59 | 3419.3(8) | 6524.8(4) | 6193.8(6) | 106(3) |
| C60 | 2695.2(8) | 6766.1(4) | 5818.9(6) | 101(3) |
| C61 | 6015.1(11) | 7440.0(5) | 7955.2(9) | 110(3) |
| C62 | 6393.5(10) | 7624.5(5) | 7665.3(9) | 104(3) |
| C63 | 5861.2(12) | 8030.8(6) | 6502.1(10) | 101(3) |
| C64 | 5214.6(13) | 8109.9(5) | 5892.4(10) | 102(3) |
| C65 | 3280.9(12) | 8062.4(5) | 4908.9(8) | 120(3) |
| C66 | 2586.6(11) | 7991.1(4) | 4883.9(7) | 136(4) |
| C67 | 1897.2(8) | 7524.9(4) | 5469.0(8) | 103(3) |
| C68 | 2197.7(9) | 7349.6(5) | 6053.6(8) | 88(2) |
| C69 | 3602.4(12) | 7164.1(5) | 7313.4(8) | 83(2) |
| C70 | 4520.0(12) | 7179.1(5) | 7771.6(8) | 99(3) |
| C71 | 1248.8(12) | 4655.2(6) | 2573.1(11) | 95(3) |
| C72 | 398.6(13) | 4749.2(6) | 2070.3(10) | 101(3) |
| C73 | 372.6(13) | 5174.6(6) | 1897.0(10) | 103(3) |
| C74 | 1234.2(13) | 5325.1(6) | 2310.6(10) | 94(2) |
| C75 | 1737.7(12) | 5002.6(6) | 2703.3(10) | 97(3) |
| C76 | -203.9(12) | 4593.4(6) | 2082.5(10) | 118(3) |
| C77 | -89.9(12) | 4336.9(6) | 2542.5(11) | 132(4) |
| C78 | -712.1(12) | 4455.1(6) | 2631.7(11) | 114(3) |
| C79 | -1227.8(12) | 4745.0(6) | 2192.8(11) | 138(4) |
| C80 | -921.2(12) | 4857.9(6) | 1867.6(10) | 121(4) |
| C81 | -944.3(13) | 5214.9(6) | 1685.9(10) | 117(3) |
| C82 | -338.6(13) | 5388.7(6) | 1671.0(9) | 116(3) |
| C83 | -222.3(13) | 5800.4(6) | 1845.7(8) | 118(3) |
| C84 | -846.5(12) | 5888.6(6) | 1945.6(8) | 125(4) |
| C85 | -1303.6(12) | 5544.6(6) | 1823.7(9) | 129(4) |
| C86 | 463.5(13) | 5988.7(6) | 2223.2(8) | 108(3) |
| C87 | 744.4(11) | 6241.0(5) | 2763.9(7) | 139(4) |
| C88 | 1581.1(10) | 6152.7(5) | 3233.2(7) | 115(3) |
| C89 | 1846.8(11) | 5843.1(5) | 3004.3(8) | 120(3) |
| C90 | 1177.5(13) | 5771.7(6) | 2408.8(9) | 132(4) |
| C91 | 200.4(10) | 6296.2(5) | 2780.8(7) | 131(4) |
| C92 | 493.1(8) | 6320.2(4) | 3427.7(7) | 110(3) |
| C93 | -174.9(8) | 6175.7(5) | 3411.5(8) | 107(3) |
| C94 | -838.7(9) | 6052.6(5) | 2800.1(9) | 106(3) |
| C95 | -635.2(11) | 6126.4(5) | 2411.5(8) | 97(3) |
| C96 | -1293.0(10) | 5708.6(6) | 2720.4(10) | 93(2) |
| C97 | -1132.9(10) | 5464.9(6) | 3206.4(10) | 101(3) |
| C98 | -1218.8(11) | 5045.1(6) | 3044.1(10) | 101(3) |
| C99 | -1464.6(11) | 5034.9(6) | 2437.1(10) | 120(3) |
| C100 | -1502.3(11) | 5442.1(6) | 2230.7(10) | 113(3) |
| C101 | -216.9(12) | 5275.9(6) | 4234.8(10) | 115(3) |
| C102 | -369.1(13) | 4858.3(6) | 4049.3(10) | 119(3) |
| C103 | 359.1(14) | 4639.4(6) | 4465.8(10) | 114(2) |
| C104 | 974.8(13) | 4911.4(5) | 4935.5(9) | 119(3) |
| C105 | 589.7(12) | 5312.7(5) | 4764.9(9) | 127(3) |
| C106 | 706.9(8) | 5930.0(4) | 4439.3(8) | 126(4) |
| C107 | 1019.1(10) | 5607.5(5) | 4880.8(8) | 134(4) |
| C108 | 1910.4(10) | 5565.1(4) | 5181.1(7) | 132(4) |
| C109 | 2117.2(8) | 5866.6(4) | 4924.3(7) | 137(3) |
| C110 | 1402.5(7) | 6090.1(4) | 4493.5(7) | 130(4) |
| C111 | 2649.3(8) | 5807.7(4) | 4773.6(7) | 130(3) |
| C112 | 2543.9(8) | 5917.7(4) | 4222.8(7) | 120(3) |
| C113 | 2835.4(9) | 5602.2(5) | 4069.1(8) | 112(3) |
| C114 | 3145.8(9) | 5283.2(5) | 4525.7(9) | 127(4) |
| C115 | 3060.3(8) | 5417.9(4) | 4967.7(8) | 135(4) |
| C116 | 3043.4(10) | 4922.0(5) | 4388.2(10) | 130(3) |
| C117 | 2870.8(11) | 4608.8(5) | 4660.3(10) | 132(3) |
| C118 | 2262.3(12) | 4348.2(5) | 4212.1(10) | 137(3) |
| C119 | 2167.9(11) | 4445.1(6) | 3654.1(11) | 124(3) |
| C120 | 2649.2(10) | 4803.6(5) | 3779.2(10) | 112(3) |
| C121 | 850.7(13) | 4120.1(6) | 3561.4(11) | 117(3) |
| C122 | 261.9(13) | 4200.0(6) | 3665.1(11) | 128(3) |
| C123 | 660.8(14) | 4310.1(6) | 4276.2(10) | 116(2) |
| C124 | 1529.9(13) | 4298.2(5) | 4589.0(10) | 119(3) |
| C125 | 1679.1(13) | 4167.0(5) | 4160.3(10) | 130(4) |
| C126 | 2068.5(13) | 4566.0(5) | 5040.4(9) | 126(4) |
| C127 | 2725.7(11) | 4712.3(5) | 5040.7(9) | 134(4) |
| C128 | 2881.9(10) | 5135.4(4) | 5233.7(8) | 134(4) |
| C129 | 2299.4(11) | 5232.1(4) | 5343.5(8) | 147(4) |
| C130 | 1845.8(13) | 4874.0(5) | 5257.2(8) | 125(4) |
| C131 | 2409.7(11) | 5065.3(6) | 3333.3(10) | 103(3) |
| C132 | 2466.1(11) | 5525.5(5) | 3431.9(9) | 102(3) |
| C133 | 1933.7(8) | 6176.5(4) | 3878.4(7) | 132(4) |
| C134 | 1327.3(8) | 6264.3(4) | 3969.3(6) | 131(4) |
| C135 | -80.2(9) | 5957.9(5) | 3915.5(8) | 99(3) |
| C136 | -611.4(10) | 5578.7(6) | 3764.7(10) | 109(3) |
| C137 | -779.2(13) | 4711.8(6) | 3439.9(11) | 126(3) |
| C138 | -489.1(13) | 4408.3(6) | 3250.8(11) | 128(4) |
| C139 | 674.9(12) | 4219.2(6) | 2992.1(11) | 120(3) |
| C140 | 1408.9(12) | 4377.3(6) | 3057.4(11) | 102(3) |
| C141 | -610(4) | 4935(2) | 379(4) | 52.5(16) |
| C142 | -2950(4) | 5787(2) | 454(4) | 49.8(16) |
| C143 | -2529(4) | 5565(2) | 249(4) | 48.1(15) |
| C144 | -3386(4) | 5147(2) | 593(4) | 51.0(16) |
| C145 | -2530(4) | 5146(2) | 248(4) | 52.7(16) |
| C146 | -1950(5) | 4928(2) | 227(4) | 55.2(16) |
| C147 | -2754(5) | 6173(2) | 658(4) | 53.9(16) |
| C148 | 344(5) | 6356(2) | 968(4) | 60.2(18) |
| C149 | -1930(4) | 5788(2) | 239(4) | 52.4(16) |
| C150 | -2994(4) | 4936(2) | 407(4) | 51.6(16) |
| C151 | -519(5) | 4525(2) | 521(4) | 57.7(17) |
| C152 | -3579(4) | 4991(2) | 972(4) | 51.6(16) |
| C153 | -1834(5) | 4511(2) | 372(4) | 57.0(17) |
| C154 | 139(4) | 5555(2) | 607(4) | 53.2(16) |
| C155 | -2127(4) | 6391(2) | 675(4) | 54.6(17) |
| C156 | 930(4) | 5729(2) | 977(4) | 58.9(18) |
| C157 | -1750(4) | 6197(2) | 455(4) | 52.1(16) |
| C158 | -1325(5) | 5572(2) | 241(4) | 53.1(16) |
| C159 | -438(5) | 6177(2) | 590(4) | 56.5(17) |
| C160 | -574(4) | 5783(2) | 424(4) | 52.7(16) |
| C161 | 121(5) | 5137(2) | 596(4) | 53.6(16) |
| C162 | -1110(5) | 4317(2) | 530(4) | 61.7(18) |
| C163 | -2923(5) | 6337(2) | 1037(4) | 56.2(18) |
| C164 | -1162(5) | 3785(2) | 1107(5) | 68(2) |
| C165 | -1354(4) | 5146(2) | 213(4) | 54.0(16) |
| C166 | 751(5) | 3753(3) | 1646(4) | 68(2) |
| C167 | 81(5) | 3897(2) | 1001(4) | 64(2) |
| C168 | -3383(4) | 5568(2) | 603(4) | 50.5(16) |
| C169 | -3557(4) | 5713(2) | 994(4) | 55.8(17) |
| C170 | -2800(5) | 4540(2) | 609(4) | 60.5(18) |
| C171 | -2204(5) | 4325(2) | 592(4) | 58.2(17) |
| C172 | 915(5) | 4955(2) | 959(4) | 57.7(18) |
| C173 | -3383(5) | 4596(2) | 1163(4) | 59.4(18) |
| C174 | -3567(5) | 5348(3) | 1788(4) | 64(2) |
| C175 | -3332(4) | 6109(2) | 1212(4) | 51.4(17) |
| C176 | 882(5) | 6913(3) | 1802(4) | 66(2) |
| C177 | -1060(5) | 6903(2) | 1209(4) | 59.0(19) |
| C178 | -1810(5) | 6725(2) | 1042(4) | 59.4(19) |
| C179 | -2966(5) | 4369(2) | 987(4) | 56.9(18) |
| C180 | 192(4) | 6787(2) | 1123(4) | 53.7(18) |
| C181 | -791(5) | 3973(2) | 860(4) | 61.9(19) |
| C182 | 1050(5) | 6139(2) | 1171(4) | 57.4(18) |
| C183 | -1901(5) | 3974(2) | 956(4) | 61.3(19) |
| C184 | 247(5) | 4318(2) | 874(4) | 59.4(18) |
| C185 | -3885(4) | 5345(2) | 1150(4) | 53.3(18) |
| C186 | 103(5) | 7111(3) | 693(5) | 72(3) |
| C187 | -1028(5) | 6395(2) | 607(4) | 55.4(17) |
| C188 | -4850(4) | 5358(3) | 715(4) | 65(2) |
| C189 | 980(5) | 4543(2) | 1081(4) | 58.9(19) |
| C190 | -679(5) | 6730(2) | 983(4) | 59.6(18) |
| C191 | -40(6) | 3611(2) | 528(5) | 67(2) |
| C192 | 5316(4) | 2744(2) | 5804(4) | 56.5(17) |
| C193 | 2120(4) | 2486(2) | 5494(4) | 50.2(16) |
| C194 | 3833(4) | 2753(2) | 5396(4) | 53.2(16) |
| C195 | 2203(4) | 3321(2) | 5662(4) | 50.8(16) |
| C196 | 2086(5) | 1873(2) | 5971(4) | 57.1(18) |
| C197 | 3871(5) | 3173(2) | 5483(4) | 56.1(17) |
| C198 | 4551(5) | 2540(2) | 5530(4) | 52.6(16) |
| C199 | 2642(4) | 3144(2) | 5454(4) | 52.2(16) |
| C200 | 4639(5) | 2124(2) | 5626(4) | 56.7(17) |
| C201 | 5355(4) | 3160(2) | 5879(4) | 51.2(16) |
| C202 | 4650(5) | 3375(2) | 5706(4) | 57.5(17) |
| C203 | 3196(4) | 2524(2) | 5342(4) | 51.9(16) |
| C204 | 3469(5) | 3762(2) | 5775(4) | 61.9(18) |
| C205 | 5384(5) | 1913(2) | 5962(4) | 51.9(17) |
| C206 | 3276(4) | 3365(2) | 5508(4) | 54.1(17) |
| C207 | 2881(5) | 1875(2) | 5612(4) | 53.8(17) |
| C208 | 6087(5) | 2556(2) | 6156(4) | 55.0(17) |
| C209 | 2602(5) | 2724(2) | 5371(4) | 53.5(16) |
| C210 | 1712(5) | 2672(2) | 5694(4) | 56.4(17) |
| C211 | 4791(5) | 3758(2) | 5933(4) | 60.9(19) |
| C212 | 1760(4) | 3087(2) | 5774(4) | 55.0(17) |
| C213 | 3309(4) | 2095(2) | 5444(4) | 50.8(16) |
| C214 | 3084(5) | 3918(2) | 6007(4) | 61.4(18) |
| C215 | 2444(5) | 3703(2) | 5947(4) | 62.8(19) |
| C216 | 3433(5) | 4223(3) | 6445(5) | 76(2) |
| C217 | 1552(5) | 3195(2) | 6154(4) | 56.9(18) |
| C218 | 2286(4) | 2076(2) | 5636(4) | 53.0(16) |
| C219 | 3166(5) | 1513(2) | 5914(4) | 55.9(18) |
| C220 | 4002(5) | 1905(2) | 5573(4) | 56.5(17) |
| C221 | 1510(5) | 2487(2) | 6044(4) | 52.5(17) |
| C222 | 1226(5) | 2819(2) | 6274(4) | 57.9(19) |
| C223 | 5178(4) | 1468(2) | 6016(4) | 54.9(18) |
| C224 | 3886(5) | 1316(2) | 6045(4) | 61.1(19) |
| C225 | 1700(5) | 2079(2) | 6194(4) | 61.4(19) |
| C226 | 6170(5) | 3318(2) | 6292(4) | 59.5(19) |
| C227 | 253(5) | 2820(3) | 5835(5) | 75(3) |
| C228 | 6152(5) | 2123(2) | 6248(4) | 56.2(18) |
| C229 | 5581(5) | 3936(3) | 6332(5) | 71(2) |
| C230 | 4295(5) | 1531(2) | 5856(4) | 57.2(18) |
| C231 | 5805(5) | 1289(3) | 6630(4) | 68(2) |
| C232 | 5454(5) | 4338(2) | 6542(5) | 71(2) |
| C233 | 5096(5) | 1214(3) | 5527(4) | 67(2) |
| C234 | 4204(5) | 3959(2) | 5971(5) | 66(2) |
| C235 | 6275(5) | 3710(2) | 6522(5) | 66(2) |
| C236 | 6137(6) | 4449(3) | 7200(6) | 100(4) |
| C237 | 2275(5) | 3836(3) | 6338(4) | 62(2) |
| C238 | 4181(5) | 4416(3) | 6623(5) | 75(2) |
| C239 | 1532(6) | 2769(3) | 6919(4) | 76(3) |
| C240 | 4568(6) | 4273(3) | 6382(5) | 75(2) |
| C241 | 5369(6) | 4686(3) | 6146(6) | 91(3) |
| C242 | 1841(5) | 3582(3) | 6467(5) | 70(2) |

Table 3 Anisotropic Displacement Parameters (Å2×103) for z\_sq\_tw. The Anisotropic displacement factor exponent takes the form: -2π2[h2a\*2U11+2hka\*b\*U12+…].

| Atom | U11 | U22 | U33 | U23 | U13 | U12 |
| --- | --- | --- | --- | --- | --- | --- |
| S1 | 44.3(9) | 58.5(11) | 94.1(16) | -6.2(11) | 50.8(11) | -1.3(8) |
| S2 | 78.4(14) | 44.4(10) | 130(2) | -8.0(12) | 82.3(16) | -7.2(10) |
| S3 | 56.7(11) | 46.4(10) | 111.2(18) | -15.5(11) | 62.0(13) | -4.7(8) |
| S4 | 51.8(10) | 66.7(12) | 95.5(17) | -0.8(11) | 55.6(12) | -4.7(9) |
| S5 | 65.5(12) | 50.0(11) | 125(2) | 11.4(12) | 72.8(14) | 1.3(9) |
| S6 | 50.3(11) | 78.4(15) | 95.3(19) | -26.4(13) | 36.0(12) | 6.7(10) |
| C1 | 69(4) | 81(5) | 120(5) | 11(4) | 74(4) | 11(4) |
| C2 | 70(4) | 87(5) | 119(5) | -1(4) | 76(4) | 5(4) |
| C3 | 80(4) | 89(5) | 113(5) | 10(4) | 87(4) | 7(4) |
| C4 | 97(5) | 93(5) | 114(5) | 6(5) | 82(4) | 0(4) |
| C5 | 87(5) | 86(5) | 138(6) | 7(5) | 70(5) | 10(4) |
| C6 | 75(4) | 99(5) | 136(6) | 17(5) | 81(4) | 16(4) |
| C7 | 87(5) | 84(5) | 142(6) | 23(5) | 83(4) | 24(4) |
| C8 | 96(5) | 88(5) | 139(6) | 34(5) | 84(4) | 23(4) |
| C9 | 91(5) | 111(6) | 147(6) | 15(6) | 69(5) | 29(5) |
| C10 | 75(5) | 112(6) | 142(6) | 19(5) | 72(4) | 26(4) |
| C11 | 89(5) | 96(6) | 137(6) | 3(5) | 86(4) | 10(4) |
| C12 | 96(5) | 80(5) | 137(6) | 26(5) | 79(4) | 25(4) |
| C13 | 104(5) | 79(5) | 138(6) | 24(5) | 85(5) | 14(4) |
| C14 | 85(5) | 89(5) | 139(6) | 8(5) | 80(4) | -6(4) |
| C15 | 94(5) | 85(5) | 122(6) | 5(5) | 68(5) | 0(5) |
| C16 | 100(5) | 104(6) | 126(6) | -10(5) | 81(4) | -4(5) |
| C17 | 97(5) | 98(5) | 109(5) | -5(5) | 84(4) | -3(4) |
| C18 | 94(5) | 95(6) | 121(6) | 16(5) | 69(4) | -12(5) |
| C19 | 106(5) | 101(6) | 107(6) | 6(5) | 70(4) | -18(5) |
| C20 | 95(5) | 94(5) | 113(5) | -18(5) | 79(4) | -8(4) |
| C21 | 72(4) | 89(5) | 123(6) | 0(4) | 69(4) | -15(4) |
| C22 | 98(5) | 90(5) | 136(6) | 4(5) | 85(4) | -14(4) |
| C23 | 89(5) | 97(6) | 135(6) | -2(5) | 79(4) | -15(4) |
| C24 | 83(4) | 94(5) | 126(6) | 8(5) | 79(4) | -17(4) |
| C25 | 81(5) | 104(6) | 133(6) | -11(5) | 73(4) | -13(4) |
| C26 | 72(5) | 109(6) | 151(6) | -11(5) | 69(5) | 16(4) |
| C27 | 80(5) | 119(6) | 141(6) | 6(5) | 84(4) | -1(4) |
| C28 | 92(5) | 114(6) | 135(6) | -2(5) | 80(5) | 2(5) |
| C29 | 99(5) | 105(6) | 150(6) | -11(6) | 68(5) | 16(5) |
| C30 | 79(5) | 96(6) | 146(6) | 13(5) | 77(4) | 13(4) |
| C31 | 91(5) | 100(6) | 155(6) | 4(5) | 78(5) | 22(5) |
| C32 | 95(5) | 86(5) | 130(6) | 23(5) | 64(5) | 5(5) |
| C33 | 80(5) | 87(5) | 142(6) | 33(5) | 70(4) | 8(4) |
| C34 | 92(5) | 83(5) | 156(6) | 16(5) | 71(5) | -1(5) |
| C35 | 97(5) | 83(5) | 148(6) | 15(5) | 75(5) | 11(5) |
| C36 | 114(6) | 91(6) | 145(7) | 17(6) | 66(5) | -3(5) |
| C37 | 111(5) | 94(6) | 153(6) | 8(5) | 83(5) | 4(5) |
| C38 | 110(5) | 107(6) | 136(6) | 1(5) | 95(4) | -13(5) |
| C39 | 112(5) | 102(6) | 141(6) | -10(5) | 84(5) | 10(5) |
| C40 | 92(5) | 104(6) | 155(6) | 7(5) | 85(5) | -3(5) |
| C41 | 118(5) | 102(6) | 124(6) | 5(5) | 99(4) | -12(5) |
| C42 | 119(6) | 127(6) | 121(6) | -8(5) | 88(5) | 1(5) |
| C43 | 107(5) | 107(6) | 122(6) | 24(5) | 88(4) | 3(5) |
| C44 | 116(5) | 116(6) | 127(6) | 0(5) | 102(4) | -10(5) |
| C45 | 102(5) | 120(6) | 131(6) | 0(5) | 94(4) | 16(5) |
| C46 | 115(5) | 86(5) | 137(6) | 30(5) | 93(5) | 6(5) |
| C47 | 106(5) | 95(5) | 140(6) | 32(5) | 97(4) | 6(4) |
| C48 | 100(5) | 87(5) | 132(6) | 23(5) | 79(4) | -20(4) |
| C49 | 108(5) | 70(5) | 125(6) | 21(4) | 89(4) | 3(4) |
| C50 | 89(5) | 75(5) | 147(6) | 32(5) | 75(5) | 13(4) |
| C51 | 125(6) | 110(6) | 129(6) | 8(6) | 57(5) | 5(6) |
| C52 | 73(5) | 115(6) | 149(6) | -24(5) | 64(4) | 7(5) |
| C53 | 105(5) | 102(6) | 132(6) | -16(5) | 81(4) | -19(5) |
| C54 | 101(5) | 98(5) | 140(5) | -8(5) | 88(4) | -7(4) |
| C55 | 117(5) | 123(6) | 116(6) | -8(5) | 86(5) | -13(5) |
| C56 | 88(5) | 81(5) | 134(6) | 4(5) | 84(4) | -17(4) |
| C57 | 89(5) | 75(5) | 138(6) | 11(5) | 86(4) | -12(4) |
| C58 | 87(5) | 75(5) | 152(6) | 15(5) | 76(5) | -4(4) |
| C59 | 108(5) | 84(5) | 146(6) | 2(5) | 88(5) | -9(5) |
| C60 | 103(5) | 91(5) | 139(6) | -4(5) | 89(4) | -12(4) |
| C61 | 80(5) | 101(6) | 134(6) | 20(5) | 59(5) | 0(5) |
| C62 | 69(4) | 96(5) | 134(6) | 13(5) | 55(4) | -5(4) |
| C63 | 84(5) | 112(6) | 142(6) | 0(5) | 88(4) | -17(4) |
| C64 | 104(5) | 117(6) | 126(6) | 22(5) | 91(4) | -17(5) |
| C65 | 118(5) | 133(6) | 134(6) | 32(5) | 90(5) | 3(5) |
| C66 | 110(6) | 119(6) | 146(6) | 3(6) | 62(5) | 12(5) |
| C67 | 77(5) | 119(6) | 124(6) | -6(5) | 68(4) | 7(4) |
| C68 | 68(4) | 86(5) | 143(6) | -3(5) | 81(4) | -12(4) |
| C69 | 77(4) | 77(5) | 129(5) | 8(4) | 81(4) | -10(4) |
| C70 | 95(5) | 87(5) | 127(6) | 25(5) | 75(4) | -9(4) |
| C71 | 94(5) | 100(6) | 128(6) | -12(5) | 87(4) | 15(4) |
| C72 | 96(5) | 103(5) | 113(5) | -29(5) | 69(4) | -1(4) |
| C73 | 100(5) | 116(5) | 102(5) | -10(5) | 67(4) | 24(4) |
| C74 | 107(5) | 105(5) | 116(5) | -11(5) | 93(4) | -11(4) |
| C75 | 82(5) | 121(6) | 129(6) | -17(5) | 86(4) | 0(4) |
| C76 | 98(5) | 102(5) | 129(6) | -26(5) | 57(4) | -4(5) |
| C77 | 105(6) | 99(6) | 142(6) | -14(6) | 51(5) | -4(5) |
| C78 | 84(5) | 92(6) | 143(6) | -6(5) | 58(5) | -18(5) |
| C79 | 88(5) | 109(6) | 154(6) | -23(6) | 43(5) | -19(5) |
| C80 | 94(5) | 97(5) | 120(5) | -17(5) | 38(4) | -7(5) |
| C81 | 93(5) | 102(5) | 115(5) | -7(5) | 43(4) | -1(5) |
| C82 | 101(5) | 107(5) | 114(5) | 0(5) | 52(4) | 0(5) |
| C83 | 110(5) | 103(5) | 107(5) | 2(5) | 50(4) | 11(5) |
| C84 | 98(5) | 102(5) | 122(6) | -7(5) | 41(5) | 14(5) |
| C85 | 93(5) | 112(5) | 127(5) | -6(5) | 40(4) | 15(5) |
| C86 | 111(5) | 97(6) | 119(6) | 10(5) | 71(5) | -18(5) |
| C87 | 106(5) | 114(6) | 164(7) | 11(6) | 67(5) | -9(5) |
| C88 | 89(5) | 95(6) | 162(7) | 8(6) | 77(5) | -23(5) |
| C89 | 104(5) | 137(7) | 142(6) | 10(6) | 88(5) | -41(5) |
| C90 | 121(6) | 127(6) | 136(6) | 4(6) | 73(5) | -15(5) |
| C91 | 112(6) | 95(6) | 157(7) | 16(6) | 69(5) | 2(5) |
| C92 | 101(5) | 88(6) | 142(6) | -9(5) | 76(5) | 5(5) |
| C93 | 93(5) | 100(6) | 146(6) | -20(5) | 82(5) | 20(5) |
| C94 | 87(5) | 108(6) | 140(6) | -9(5) | 78(4) | 16(5) |
| C95 | 89(5) | 90(5) | 130(6) | 4(5) | 77(4) | 12(4) |
| C96 | 69(4) | 103(6) | 139(6) | 0(5) | 81(4) | 11(4) |
| C97 | 76(4) | 127(6) | 143(6) | -8(5) | 89(4) | 3(4) |
| C98 | 63(4) | 101(6) | 157(6) | 2(5) | 77(4) | -5(4) |
| C99 | 63(4) | 109(6) | 152(6) | -26(6) | 48(5) | -13(4) |
| C100 | 67(4) | 111(6) | 134(6) | -6(5) | 49(4) | 10(4) |
| C101 | 98(5) | 126(6) | 141(6) | 3(6) | 84(5) | 4(5) |
| C102 | 104(5) | 127(6) | 150(6) | 13(6) | 90(5) | -10(5) |
| C103 | 109(5) | 126(6) | 138(5) | 30(5) | 92(4) | -11(5) |
| C104 | 123(6) | 130(6) | 129(6) | 20(5) | 90(5) | 13(5) |
| C105 | 115(6) | 133(7) | 140(6) | 6(6) | 82(5) | -3(5) |
| C106 | 109(6) | 114(6) | 143(6) | -20(6) | 71(5) | 15(5) |
| C107 | 131(6) | 128(7) | 142(6) | -7(6) | 83(5) | 0(6) |
| C108 | 118(6) | 114(6) | 140(6) | 1(6) | 67(5) | 9(6) |
| C109 | 107(5) | 114(6) | 160(6) | -6(5) | 66(4) | -7(5) |
| C110 | 105(5) | 111(6) | 154(6) | -19(6) | 69(5) | 4(5) |
| C111 | 101(5) | 110(6) | 151(6) | 6(5) | 63(4) | -26(5) |
| C112 | 89(5) | 105(6) | 166(6) | -7(6) | 78(5) | -16(5) |
| C113 | 75(5) | 115(6) | 162(6) | -8(6) | 82(5) | -13(4) |
| C114 | 81(5) | 130(7) | 159(7) | -5(6) | 69(5) | 6(5) |
| C115 | 99(6) | 124(6) | 153(7) | 6(6) | 63(5) | -1(5) |
| C116 | 79(5) | 128(6) | 165(7) | 2(5) | 65(5) | 18(4) |
| C117 | 101(5) | 112(6) | 142(5) | 8(4) | 53(5) | 25(4) |
| C118 | 118(6) | 108(6) | 146(5) | 8(5) | 61(5) | 14(4) |
| C119 | 102(5) | 112(6) | 158(5) | -12(5) | 81(5) | 33(5) |
| C120 | 82(5) | 117(6) | 149(6) | -6(6) | 77(5) | 20(5) |
| C121 | 120(5) | 93(6) | 131(5) | 11(5) | 74(4) | -11(5) |
| C122 | 112(5) | 113(6) | 149(5) | 3(5) | 74(4) | -15(5) |
| C123 | 117(5) | 103(5) | 141(4) | 29(5) | 86(4) | -12(4) |
| C124 | 112(5) | 92(5) | 134(6) | 18(5) | 66(5) | -2(5) |
| C125 | 114(6) | 101(6) | 161(7) | 20(6) | 78(5) | 5(5) |
| C126 | 109(5) | 115(6) | 132(6) | 27(5) | 63(5) | -3(5) |
| C127 | 101(5) | 117(6) | 143(6) | 21(5) | 54(5) | 6(5) |
| C128 | 103(6) | 117(6) | 146(7) | 6(6) | 58(5) | 0(5) |
| C129 | 123(6) | 134(7) | 140(7) | 2(6) | 58(6) | -2(6) |
| C130 | 118(6) | 120(6) | 126(6) | 19(5) | 68(5) | -7(5) |
| C131 | 85(5) | 128(6) | 144(6) | -1(5) | 95(4) | 7(5) |
| C132 | 78(4) | 117(6) | 157(6) | 5(5) | 96(4) | -13(4) |
| C133 | 95(5) | 102(6) | 163(6) | 7(6) | 62(5) | -32(5) |
| C134 | 109(5) | 100(6) | 160(6) | -10(6) | 70(5) | 0(5) |
| C135 | 90(5) | 104(6) | 140(6) | -17(5) | 90(4) | 15(4) |
| C136 | 80(5) | 128(6) | 151(6) | -12(5) | 87(4) | 8(5) |
| C137 | 97(5) | 132(6) | 157(6) | 9(6) | 81(5) | -31(5) |
| C138 | 83(5) | 97(6) | 167(7) | -1(6) | 58(5) | -25(5) |
| C139 | 103(5) | 92(6) | 151(6) | -14(5) | 70(5) | 5(5) |
| C140 | 93(5) | 98(6) | 139(6) | -15(5) | 83(4) | 12(4) |
| C141 | 49(3) | 53(4) | 79(4) | -10(3) | 51(3) | -2(3) |
| C142 | 39(3) | 50(3) | 75(4) | -14(3) | 43(3) | -2(3) |
| C143 | 43(3) | 47(3) | 75(4) | -9(3) | 48(3) | 0(3) |
| C144 | 45(3) | 52(4) | 71(4) | -11(3) | 43(3) | -7(3) |
| C145 | 47(3) | 55(4) | 77(4) | -9(3) | 49(3) | -4(3) |
| C146 | 48(3) | 51(4) | 86(4) | -10(3) | 52(3) | -5(3) |
| C147 | 49(3) | 48(4) | 85(4) | -6(3) | 51(3) | 1(3) |
| C148 | 57(4) | 50(4) | 97(5) | -4(4) | 58(4) | -9(3) |
| C149 | 45(3) | 45(3) | 84(4) | -2(3) | 48(3) | -2(3) |
| C150 | 46(3) | 47(3) | 80(4) | -12(3) | 48(3) | -7(3) |
| C151 | 55(3) | 54(4) | 89(4) | -20(3) | 56(3) | -3(3) |
| C152 | 46(3) | 56(4) | 79(4) | -12(3) | 51(3) | -8(3) |
| C153 | 53(3) | 44(3) | 93(4) | -13(3) | 55(3) | -5(3) |
| C154 | 44(3) | 53(4) | 81(4) | -7(3) | 48(3) | -4(3) |
| C155 | 48(3) | 48(4) | 87(4) | -6(3) | 51(3) | -1(3) |
| C156 | 42(3) | 63(4) | 90(5) | -4(4) | 50(3) | -5(3) |
| C157 | 45(3) | 49(3) | 80(4) | 0(3) | 48(3) | -2(3) |
| C158 | 49(3) | 55(4) | 75(4) | -7(3) | 48(3) | -1(3) |
| C159 | 51(3) | 55(4) | 84(4) | -6(3) | 52(3) | -2(3) |
| C160 | 48(3) | 47(3) | 86(4) | -5(3) | 53(3) | -1(3) |
| C161 | 53(3) | 57(4) | 78(4) | -7(3) | 54(3) | 0(3) |
| C162 | 55(3) | 49(4) | 98(5) | -19(4) | 56(3) | -2(3) |
| C163 | 48(3) | 53(4) | 84(5) | -12(4) | 49(3) | -5(3) |
| C164 | 72(4) | 50(4) | 103(5) | -11(4) | 66(4) | 0(3) |
| C165 | 48(3) | 58(4) | 84(4) | -9(3) | 54(3) | -8(3) |
| C166 | 62(5) | 54(5) | 89(6) | -15(4) | 46(5) | 2(4) |
| C167 | 54(4) | 54(4) | 91(6) | -24(4) | 47(4) | -4(3) |
| C168 | 41(3) | 51(4) | 74(4) | -11(3) | 43(3) | -5(3) |
| C169 | 43(3) | 59(4) | 83(5) | -15(4) | 47(3) | -7(3) |
| C170 | 51(3) | 45(4) | 108(5) | -18(3) | 60(3) | -7(3) |
| C171 | 52(3) | 45(4) | 99(5) | -16(3) | 58(3) | -4(3) |
| C172 | 49(3) | 60(4) | 91(5) | -11(4) | 56(4) | -3(3) |
| C173 | 57(4) | 49(4) | 92(5) | -9(4) | 56(4) | -12(3) |
| C174 | 52(4) | 71(5) | 88(6) | -10(5) | 53(4) | -14(4) |
| C175 | 35(3) | 46(4) | 80(5) | -13(3) | 39(3) | -5(3) |
| C176 | 58(4) | 65(5) | 102(7) | -6(5) | 62(5) | -8(4) |
| C177 | 57(4) | 35(3) | 101(5) | -6(4) | 56(4) | -3(3) |
| C178 | 48(3) | 42(4) | 99(5) | -2(4) | 50(4) | 6(3) |
| C179 | 59(4) | 44(4) | 91(5) | -9(4) | 58(4) | -8(3) |
| C180 | 46(3) | 44(4) | 84(5) | 4(4) | 47(4) | 0(3) |
| C181 | 62(4) | 45(4) | 96(5) | -14(4) | 58(4) | 1(3) |
| C182 | 45(3) | 49(4) | 94(5) | -6(4) | 51(4) | -7(3) |
| C183 | 70(4) | 38(4) | 99(5) | -16(4) | 64(4) | -7(3) |
| C184 | 60(4) | 56(4) | 88(5) | -15(4) | 58(4) | 1(3) |
| C185 | 44(3) | 52(4) | 85(5) | -22(4) | 50(4) | -10(3) |
| C186 | 47(4) | 70(5) | 104(7) | 7(5) | 49(5) | -9(4) |
| C187 | 52(3) | 47(4) | 85(4) | -5(3) | 51(3) | -4(3) |
| C188 | 43(4) | 70(5) | 100(6) | -20(5) | 52(4) | -18(4) |
| C189 | 49(3) | 55(4) | 88(5) | -15(4) | 50(4) | 3(3) |
| C190 | 50(3) | 43(4) | 102(5) | -1(4) | 54(4) | 0(3) |
| C191 | 79(5) | 54(5) | 100(7) | -15(4) | 70(5) | -5(4) |
| C192 | 44(3) | 67(4) | 75(4) | 2(4) | 45(3) | -5(3) |
| C193 | 39(3) | 52(4) | 71(4) | -1(3) | 40(3) | -4(3) |
| C194 | 44(3) | 56(4) | 75(4) | -1(3) | 45(3) | -2(3) |
| C195 | 44(3) | 50(4) | 68(4) | -5(3) | 39(3) | 4(3) |
| C196 | 48(3) | 53(4) | 88(5) | 7(4) | 51(4) | 1(3) |
| C197 | 49(3) | 51(4) | 77(4) | 7(3) | 44(3) | -4(3) |
| C198 | 50(3) | 61(4) | 68(4) | -5(3) | 48(3) | -6(3) |
| C199 | 41(3) | 45(3) | 73(4) | 5(3) | 36(3) | 0(3) |
| C200 | 51(3) | 62(4) | 74(4) | 1(4) | 47(3) | 3(3) |
| C201 | 48(3) | 51(4) | 69(4) | 5(3) | 43(3) | -5(3) |
| C202 | 53(3) | 52(4) | 78(4) | 6(3) | 45(3) | -7(3) |
| C203 | 46(3) | 51(4) | 72(4) | 3(3) | 43(3) | 1(3) |
| C204 | 53(3) | 47(4) | 83(4) | 4(3) | 41(3) | 1(3) |
| C205 | 51(3) | 51(4) | 73(4) | -6(3) | 48(3) | -3(3) |
| C206 | 45(3) | 52(4) | 70(4) | 5(3) | 38(3) | -1(3) |
| C207 | 52(3) | 50(4) | 79(4) | -4(3) | 50(3) | -7(3) |
| C208 | 49(4) | 59(4) | 69(5) | 0(4) | 42(4) | 2(3) |
| C209 | 48(3) | 55(4) | 74(4) | 0(3) | 46(3) | -5(3) |
| C210 | 47(3) | 56(4) | 78(4) | 7(3) | 45(3) | 2(3) |
| C211 | 50(3) | 47(4) | 85(5) | 8(4) | 41(3) | -5(3) |
| C212 | 42(3) | 54(4) | 81(4) | 3(3) | 44(3) | 6(3) |
| C213 | 48(3) | 47(3) | 72(4) | -2(3) | 45(3) | -7(3) |
| C214 | 46(3) | 51(4) | 88(5) | -6(4) | 42(3) | 4(3) |
| C215 | 45(3) | 55(4) | 85(5) | -3(4) | 39(3) | 5(3) |
| C216 | 54(4) | 73(5) | 93(5) | -20(4) | 42(4) | 7(4) |
| C217 | 48(3) | 67(4) | 73(5) | 5(4) | 46(3) | 9(3) |
| C218 | 47(3) | 50(4) | 77(4) | 1(3) | 46(3) | -1(3) |
| C219 | 53(4) | 45(4) | 80(5) | -4(3) | 46(4) | -4(3) |
| C220 | 51(3) | 51(4) | 81(5) | -1(3) | 47(3) | -2(3) |
| C221 | 48(3) | 62(4) | 64(4) | 4(3) | 42(3) | 2(3) |
| C222 | 48(4) | 70(5) | 75(5) | 6(4) | 47(4) | 11(3) |
| C223 | 44(3) | 58(4) | 73(5) | -4(4) | 41(4) | 8(3) |
| C224 | 58(4) | 46(4) | 91(5) | -3(4) | 51(4) | 2(3) |
| C225 | 56(4) | 66(4) | 88(5) | 4(4) | 58(4) | 0(3) |
| C226 | 51(4) | 52(4) | 85(5) | 6(4) | 47(4) | -2(3) |
| C227 | 52(4) | 81(6) | 111(7) | 2(5) | 59(5) | 7(4) |
| C228 | 51(4) | 66(4) | 70(5) | -8(4) | 47(4) | 0(3) |
| C229 | 51(4) | 52(4) | 109(6) | 7(4) | 48(4) | -4(3) |
| C230 | 44(3) | 53(4) | 81(5) | -9(4) | 42(4) | -2(3) |
| C231 | 64(5) | 64(5) | 92(7) | -13(5) | 57(5) | -8(4) |
| C232 | 53(4) | 48(4) | 106(6) | -7(4) | 45(4) | -8(3) |
| C233 | 52(4) | 71(5) | 86(6) | -27(5) | 47(5) | -5(4) |
| C234 | 51(4) | 53(4) | 96(5) | -3(4) | 46(4) | -1(3) |
| C235 | 55(4) | 53(4) | 99(6) | -2(4) | 52(4) | -9(3) |
| C236 | 57(5) | 81(7) | 125(9) | -34(6) | 37(6) | -15(5) |
| C237 | 48(4) | 67(5) | 76(5) | -9(4) | 40(4) | 0(3) |
| C238 | 53(4) | 57(4) | 106(6) | -20(4) | 44(4) | -2(3) |
| C239 | 62(5) | 120(8) | 54(5) | 15(5) | 39(5) | 24(5) |
| C240 | 60(4) | 50(4) | 103(6) | 0(4) | 43(4) | -6(3) |
| C241 | 59(5) | 53(5) | 147(9) | 14(6) | 56(6) | -5(4) |
| C242 | 48(4) | 77(5) | 90(5) | -8(4) | 45(4) | 12(4) |

Table 4 Bond Lengths for z\_sq\_tw.

| Atom | Atom | Length/Å |  | Atom | Atom | Length/Å |
| --- | --- | --- | --- | --- | --- | --- |
| S1 | C156 | 1.775(8) |  | C106 | C107 | 1.4487 |
| S1 | C172 | 1.769(8) |  | C106 | C110 | 1.4286 |
| S2 | C179 | 1.768(8) |  | C106 | C135 | 1.3499 |
| S2 | C183 | 1.760(8) |  | C107 | C108 | 1.4620 |
| S3 | C163 | 1.792(8) |  | C108 | C109 | 1.4344 |
| S3 | C178 | 1.771(8) |  | C108 | C129 | 1.2704 |
| S4 | C208 | 1.764(8) |  | C109 | C110 | 1.3987 |
| S4 | C226 | 1.754(8) |  | C109 | C111 | 1.3920 |
| S5 | C196 | 1.778(8) |  | C110 | C134 | 1.4845 |
| S5 | C219 | 1.787(7) |  | C111 | C112 | 1.4547 |
| S6 | C216 | 1.751(9) |  | C111 | C115 | 1.4536 |
| S6 | C237 | 1.749(9) |  | C112 | C113 | 1.3939 |
| C1 | C2 | 1.4091 |  | C112 | C133 | 1.3169 |
| C1 | C5 | 1.4341 |  | C113 | C114 | 1.4652 |
| C1 | C6 | 1.3777 |  | C113 | C132 | 1.4585 |
| C2 | C3 | 1.4548 |  | C114 | C115 | 1.4137 |
| C2 | C68 | 1.3987 |  | C114 | C116 | 1.2380 |
| C3 | C4 | 1.3948 |  | C115 | C128 | 1.3739 |
| C3 | C69 | 1.3819 |  | C116 | C117 | 1.4503 |
| C4 | C5 | 1.4597 |  | C116 | C120 | 1.4113 |
| C4 | C17 | 1.2951 |  | C117 | C118 | 1.3914 |
| C5 | C11 | 1.3171 |  | C117 | C127 | 1.3054 |
| C6 | C7 | 1.4411 |  | C118 | C119 | 1.4755 |
| C6 | C10 | 1.4079 |  | C118 | C125 | 1.2559 |
| C7 | C8 | 1.4033 |  | C119 | C120 | 1.4414 |
| C7 | C12 | 1.3871 |  | C119 | C140 | 1.4262 |
| C8 | C9 | 1.4229 |  | C120 | C131 | 1.3366 |
| C8 | C50 | 1.4205 |  | C121 | C122 | 1.4166 |
| C9 | C10 | 1.5122 |  | C121 | C125 | 1.4773 |
| C9 | C66 | 1.2731 |  | C121 | C139 | 1.4289 |
| C10 | C67 | 1.4523 |  | C122 | C123 | 1.4075 |
| C11 | C12 | 1.4125 |  | C122 | C138 | 1.4047 |
| C11 | C15 | 1.4585 |  | C123 | C124 | 1.4100 |
| C12 | C13 | 1.4162 |  | C124 | C125 | 1.4637 |
| C13 | C14 | 1.4141 |  | C124 | C126 | 1.3692 |
| C13 | C49 | 1.3734 |  | C126 | C127 | 1.4201 |
| C14 | C15 | 1.4369 |  | C126 | C130 | 1.3964 |
| C14 | C22 | 1.4710 |  | C127 | C128 | 1.4681 |
| C15 | C16 | 1.3607 |  | C128 | C129 | 1.4281 |
| C16 | C17 | 1.4650 |  | C129 | C130 | 1.4324 |
| C16 | C20 | 1.3899 |  | C131 | C132 | 1.5456 |
| C17 | C18 | 1.3958 |  | C133 | C134 | 1.4312 |
| C18 | C19 | 1.4459 |  | C135 | C136 | 1.5404 |
| C18 | C70 | 1.3792 |  | C137 | C138 | 1.4214 |
| C19 | C20 | 1.4143 |  | C139 | C140 | 1.4830 |
| C19 | C61 | 1.3729 |  | C141 | C151 | 1.400(11) |
| C20 | C21 | 1.4254 |  | C141 | C161 | 1.385(10) |
| C21 | C22 | 1.4441 |  | C141 | C165 | 1.459(9) |
| C21 | C25 | 1.4191 |  | C142 | C143 | 1.481(8) |
| C22 | C23 | 1.4058 |  | C142 | C147 | 1.360(10) |
| C23 | C24 | 1.4629 |  | C142 | C168 | 1.385(9) |
| C23 | C48 | 1.4596 |  | C143 | C145 | 1.393(10) |
| C24 | C25 | 1.4378 |  | C143 | C149 | 1.439(9) |
| C24 | C63 | 1.3337 |  | C144 | C150 | 1.378(9) |
| C25 | C62 | 1.3914 |  | C144 | C152 | 1.426(10) |
| C26 | C27 | 1.3901 |  | C144 | C168 | 1.401(10) |
| C26 | C30 | 1.4072 |  | C145 | C146 | 1.414(9) |
| C26 | C62 | 1.4311 |  | C145 | C150 | 1.443(9) |
| C27 | C28 | 1.4154 |  | C146 | C153 | 1.421(11) |
| C27 | C63 | 1.5257 |  | C146 | C165 | 1.431(9) |
| C28 | C29 | 1.4698 |  | C147 | C155 | 1.441(9) |
| C28 | C45 | 1.3260 |  | C147 | C163 | 1.403(10) |
| C29 | C30 | 1.4479 |  | C148 | C159 | 1.390(11) |
| C29 | C40 | 1.2550 |  | C148 | C180 | 1.578(10) |
| C30 | C31 | 1.4050 |  | C148 | C182 | 1.379(10) |
| C31 | C32 | 1.4214 |  | C149 | C157 | 1.440(10) |
| C31 | C35 | 1.4310 |  | C149 | C158 | 1.420(9) |
| C32 | C33 | 1.4323 |  | C150 | C170 | 1.389(11) |
| C32 | C61 | 1.4370 |  | C151 | C162 | 1.398(10) |
| C33 | C34 | 1.4239 |  | C151 | C184 | 1.407(11) |
| C33 | C70 | 1.4863 |  | C152 | C173 | 1.380(11) |
| C34 | C35 | 1.4393 |  | C152 | C185 | 1.547(9) |
| C34 | C58 | 1.2764 |  | C153 | C162 | 1.408(10) |
| C35 | C36 | 1.3277 |  | C153 | C171 | 1.375(10) |
| C36 | C37 | 1.3801 |  | C154 | C156 | 1.393(10) |
| C36 | C40 | 1.4817 |  | C154 | C160 | 1.423(9) |
| C37 | C38 | 1.4301 |  | C154 | C161 | 1.389(10) |
| C37 | C59 | 1.3863 |  | C155 | C157 | 1.395(9) |
| C38 | C39 | 1.4074 |  | C155 | C178 | 1.369(11) |
| C38 | C54 | 1.3804 |  | C156 | C182 | 1.432(11) |
| C39 | C40 | 1.3842 |  | C157 | C187 | 1.415(9) |
| C39 | C41 | 1.3939 |  | C158 | C160 | 1.458(9) |
| C41 | C42 | 1.4111 |  | C158 | C165 | 1.417(10) |
| C41 | C45 | 1.4335 |  | C159 | C160 | 1.361(10) |
| C42 | C43 | 1.4058 |  | C159 | C187 | 1.425(9) |
| C42 | C55 | 1.4381 |  | C161 | C172 | 1.409(11) |
| C43 | C44 | 1.4412 |  | C162 | C181 | 1.356(12) |
| C43 | C65 | 1.4281 |  | C163 | C175 | 1.413(10) |
| C44 | C45 | 1.4761 |  | C164 | C181 | 1.436(11) |
| C44 | C64 | 1.4020 |  | C164 | C183 | 1.433(11) |
| C46 | C47 | 1.4922 |  | C166 | C167 | 1.514(13) |
| C46 | C50 | 1.4305 |  | C167 | C181 | 1.582(10) |
| C46 | C65 | 1.5539 |  | C167 | C184 | 1.533(12) |
| C47 | C48 | 1.4375 |  | C167 | C191 | 1.514(11) |
| C47 | C64 | 1.5053 |  | C168 | C169 | 1.419(10) |
| C48 | C49 | 1.4266 |  | C169 | C175 | 1.402(10) |
| C49 | C50 | 1.4107 |  | C169 | C185 | 1.577(10) |
| C51 | C52 | 1.4068 |  | C170 | C171 | 1.433(9) |
| C51 | C55 | 1.4408 |  | C170 | C179 | 1.403(11) |
| C51 | C66 | 1.6592 |  | C171 | C183 | 1.412(12) |
| C52 | C53 | 1.4293 |  | C172 | C189 | 1.397(11) |
| C52 | C67 | 1.5124 |  | C173 | C179 | 1.427(10) |
| C53 | C54 | 1.4454 |  | C174 | C185 | 1.465(12) |
| C53 | C60 | 1.3971 |  | C176 | C180 | 1.564(12) |
| C54 | C55 | 1.4725 |  | C177 | C178 | 1.421(10) |
| C56 | C57 | 1.4254 |  | C177 | C190 | 1.384(10) |
| C56 | C60 | 1.4290 |  | C180 | C186 | 1.532(11) |
| C56 | C68 | 1.3987 |  | C180 | C190 | 1.573(9) |
| C57 | C58 | 1.4294 |  | C184 | C189 | 1.433(10) |
| C57 | C69 | 1.4232 |  | C185 | C188 | 1.546(10) |
| C58 | C59 | 1.4354 |  | C187 | C190 | 1.387(11) |
| C59 | C60 | 1.4171 |  | C192 | C198 | 1.413(10) |
| C61 | C62 | 1.5409 |  | C192 | C201 | 1.392(11) |
| C63 | C64 | 1.3885 |  | C192 | C208 | 1.384(11) |
| C65 | C66 | 1.3891 |  | C193 | C209 | 1.455(9) |
| C67 | C68 | 1.4593 |  | C193 | C210 | 1.392(10) |
| C69 | C70 | 1.4767 |  | C193 | C218 | 1.400(10) |
| C71 | C72 | 1.4375 |  | C194 | C197 | 1.409(11) |
| C71 | C75 | 1.4173 |  | C194 | C198 | 1.450(10) |
| C71 | C140 | 1.4935 |  | C194 | C203 | 1.426(9) |
| C72 | C73 | 1.4837 |  | C195 | C199 | 1.452(10) |
| C72 | C76 | 1.3484 |  | C195 | C212 | 1.361(10) |
| C73 | C74 | 1.4704 |  | C195 | C215 | 1.413(11) |
| C73 | C82 | 1.3684 |  | C196 | C218 | 1.395(10) |
| C74 | C75 | 1.4187 |  | C196 | C225 | 1.436(10) |
| C74 | C90 | 1.5262 |  | C197 | C202 | 1.461(10) |
| C75 | C131 | 1.4257 |  | C197 | C206 | 1.406(10) |
| C76 | C77 | 1.4314 |  | C198 | C200 | 1.399(11) |
| C76 | C80 | 1.4775 |  | C199 | C206 | 1.410(9) |
| C77 | C78 | 1.4826 |  | C199 | C209 | 1.407(10) |
| C77 | C139 | 1.3195 |  | C200 | C205 | 1.384(11) |
| C78 | C79 | 1.3935 |  | C200 | C220 | 1.410(10) |
| C78 | C138 | 1.4926 |  | C201 | C202 | 1.396(10) |
| C79 | C80 | 1.4267 |  | C201 | C226 | 1.415(11) |
| C79 | C99 | 1.4215 |  | C202 | C211 | 1.371(11) |
| C80 | C81 | 1.2787 |  | C203 | C209 | 1.423(9) |
| C81 | C82 | 1.3827 |  | C203 | C213 | 1.445(10) |
| C81 | C85 | 1.4909 |  | C204 | C206 | 1.443(11) |
| C82 | C83 | 1.4222 |  | C204 | C214 | 1.385(11) |
| C83 | C84 | 1.4840 |  | C204 | C234 | 1.400(11) |
| C83 | C86 | 1.2792 |  | C205 | C223 | 1.566(11) |
| C84 | C85 | 1.3774 |  | C205 | C228 | 1.426(10) |
| C84 | C95 | 1.3416 |  | C207 | C213 | 1.415(9) |
| C85 | C100 | 1.4551 |  | C207 | C218 | 1.420(9) |
| C86 | C87 | 1.4936 |  | C207 | C219 | 1.374(11) |
| C86 | C90 | 1.4037 |  | C208 | C228 | 1.456(11) |
| C87 | C88 | 1.3968 |  | C210 | C212 | 1.393(11) |
| C87 | C91 | 1.1490 |  | C210 | C221 | 1.407(10) |
| C88 | C89 | 1.4749 |  | C211 | C229 | 1.401(11) |
| C88 | C133 | 1.4670 |  | C211 | C234 | 1.424(11) |
| C89 | C90 | 1.3698 |  | C212 | C217 | 1.403(10) |
| C89 | C132 | 1.5068 |  | C213 | C220 | 1.373(10) |
| C91 | C92 | 1.5083 |  | C214 | C215 | 1.402(11) |
| C91 | C95 | 1.4531 |  | C214 | C216 | 1.395(12) |
| C92 | C93 | 1.4135 |  | C215 | C237 | 1.398(12) |
| C92 | C134 | 1.4269 |  | C216 | C238 | 1.429(12) |
| C93 | C94 | 1.4333 |  | C217 | C222 | 1.543(11) |
| C93 | C135 | 1.4795 |  | C217 | C242 | 1.458(12) |
| C94 | C95 | 1.3944 |  | C219 | C224 | 1.430(10) |
| C94 | C96 | 1.3987 |  | C220 | C230 | 1.394(11) |
| C96 | C97 | 1.4296 |  | C221 | C222 | 1.551(10) |
| C96 | C100 | 1.4504 |  | C221 | C225 | 1.402(11) |
| C97 | C98 | 1.4434 |  | C222 | C227 | 1.559(11) |
| C97 | C136 | 1.2848 |  | C222 | C239 | 1.502(12) |
| C98 | C99 | 1.4346 |  | C223 | C230 | 1.575(9) |
| C98 | C137 | 1.4235 |  | C223 | C231 | 1.488(12) |
| C99 | C100 | 1.4533 |  | C223 | C233 | 1.522(11) |
| C101 | C102 | 1.4462 |  | C224 | C230 | 1.418(11) |
| C101 | C105 | 1.3792 |  | C226 | C235 | 1.407(11) |
| C101 | C136 | 1.4398 |  | C229 | C232 | 1.541(12) |
| C102 | C103 | 1.3973 |  | C229 | C235 | 1.385(11) |
| C102 | C137 | 1.4363 |  | C232 | C236 | 1.512(14) |
| C103 | C104 | 1.4414 |  | C232 | C240 | 1.579(11) |
| C103 | C123 | 1.4989 |  | C232 | C241 | 1.531(13) |
| C104 | C105 | 1.4696 |  | C234 | C240 | 1.380(13) |
| C104 | C130 | 1.4146 |  | C237 | C242 | 1.415(12) |
| C105 | C107 | 1.2161 |  | C238 | C240 | 1.393(13) |

Table 5 Bond Angles for z\_sq\_tw.

| Atom | Atom | Atom | Angle/˚ |  | Atom | Atom | Atom | Angle/˚ |
| --- | --- | --- | --- | --- | --- | --- | --- | --- |
| C172 | S1 | C156 | 93.0(3) |  | C115 | C114 | C113 | 107.8 |
| C183 | S2 | C179 | 93.8(4) |  | C116 | C114 | C113 | 122.2 |
| C178 | S3 | C163 | 93.5(3) |  | C116 | C114 | C115 | 119.2 |
| C226 | S4 | C208 | 92.9(4) |  | C114 | C115 | C111 | 108.0 |
| C196 | S5 | C219 | 93.0(3) |  | C128 | C115 | C111 | 121.8 |
| C237 | S6 | C216 | 92.7(4) |  | C128 | C115 | C114 | 117.6 |
| C2 | C1 | C5 | 107.2 |  | C114 | C116 | C117 | 125.7 |
| C6 | C1 | C2 | 120.1 |  | C114 | C116 | C120 | 120.3 |
| C6 | C1 | C5 | 124.6 |  | C120 | C116 | C117 | 106.5 |
| C1 | C2 | C3 | 109.2 |  | C118 | C117 | C116 | 110.3 |
| C68 | C2 | C1 | 121.4 |  | C127 | C117 | C116 | 118.7 |
| C68 | C2 | C3 | 121.1 |  | C127 | C117 | C118 | 111.7 |
| C4 | C3 | C2 | 107.5 |  | C117 | C118 | C119 | 105.9 |
| C69 | C3 | C2 | 122.2 |  | C125 | C118 | C117 | 131.1 |
| C69 | C3 | C4 | 119.9 |  | C125 | C118 | C119 | 118.7 |
| C3 | C4 | C5 | 108.1 |  | C120 | C119 | C118 | 107.2 |
| C17 | C4 | C3 | 121.3 |  | C140 | C119 | C118 | 122.4 |
| C17 | C4 | C5 | 123.4 |  | C140 | C119 | C120 | 120.0 |
| C1 | C5 | C4 | 107.7 |  | C116 | C120 | C119 | 108.7 |
| C11 | C5 | C1 | 118.6 |  | C131 | C120 | C116 | 123.0 |
| C11 | C5 | C4 | 119.5 |  | C131 | C120 | C119 | 120.5 |
| C1 | C6 | C7 | 115.9 |  | C122 | C121 | C125 | 105.7 |
| C1 | C6 | C10 | 123.7 |  | C122 | C121 | C139 | 120.9 |
| C10 | C6 | C7 | 109.0 |  | C139 | C121 | C125 | 124.0 |
| C8 | C7 | C6 | 109.1 |  | C123 | C122 | C121 | 111.2 |
| C12 | C7 | C6 | 117.9 |  | C138 | C122 | C121 | 123.6 |
| C12 | C7 | C8 | 119.6 |  | C138 | C122 | C123 | 113.0 |
| C7 | C8 | C9 | 108.9 |  | C122 | C123 | C103 | 123.8 |
| C7 | C8 | C50 | 120.2 |  | C122 | C123 | C124 | 108.1 |
| C50 | C8 | C9 | 118.8 |  | C124 | C123 | C103 | 115.6 |
| C8 | C9 | C10 | 106.8 |  | C123 | C124 | C125 | 108.3 |
| C66 | C9 | C8 | 118.7 |  | C126 | C124 | C123 | 122.6 |
| C66 | C9 | C10 | 124.9 |  | C126 | C124 | C125 | 119.3 |
| C6 | C10 | C9 | 106.2 |  | C118 | C125 | C121 | 121.4 |
| C6 | C10 | C67 | 115.1 |  | C118 | C125 | C124 | 114.6 |
| C67 | C10 | C9 | 127.7 |  | C124 | C125 | C121 | 106.6 |
| C5 | C11 | C12 | 119.2 |  | C124 | C126 | C127 | 116.4 |
| C5 | C11 | C15 | 118.8 |  | C124 | C126 | C130 | 125.7 |
| C12 | C11 | C15 | 108.8 |  | C130 | C126 | C127 | 107.6 |
| C7 | C12 | C11 | 123.6 |  | C117 | C127 | C126 | 126.1 |
| C7 | C12 | C13 | 119.4 |  | C117 | C127 | C128 | 116.2 |
| C11 | C12 | C13 | 108.1 |  | C126 | C127 | C128 | 108.7 |
| C14 | C13 | C12 | 108.4 |  | C115 | C128 | C127 | 121.8 |
| C49 | C13 | C12 | 122.4 |  | C115 | C128 | C129 | 119.2 |
| C49 | C13 | C14 | 118.1 |  | C129 | C128 | C127 | 105.8 |
| C13 | C14 | C15 | 109.2 |  | C108 | C129 | C128 | 122.5 |
| C13 | C14 | C22 | 126.0 |  | C108 | C129 | C130 | 117.9 |
| C15 | C14 | C22 | 116.1 |  | C128 | C129 | C130 | 108.0 |
| C14 | C15 | C11 | 105.4 |  | C104 | C130 | C129 | 117.3 |
| C16 | C15 | C11 | 121.0 |  | C126 | C130 | C104 | 113.8 |
| C16 | C15 | C14 | 122.4 |  | C126 | C130 | C129 | 109.6 |
| C15 | C16 | C17 | 119.1 |  | C75 | C131 | C132 | 105.8 |
| C15 | C16 | C20 | 121.1 |  | C120 | C131 | C75 | 124.8 |
| C20 | C16 | C17 | 108.0 |  | C120 | C131 | C132 | 122.3 |
| C4 | C17 | C16 | 118.2 |  | C89 | C132 | C131 | 128.2 |
| C4 | C17 | C18 | 122.3 |  | C113 | C132 | C89 | 113.1 |
| C18 | C17 | C16 | 106.5 |  | C113 | C132 | C131 | 108.3 |
| C17 | C18 | C19 | 109.4 |  | C112 | C133 | C88 | 112.8 |
| C70 | C18 | C17 | 120.3 |  | C112 | C133 | C134 | 122.2 |
| C70 | C18 | C19 | 121.4 |  | C134 | C133 | C88 | 113.5 |
| C20 | C19 | C18 | 106.5 |  | C92 | C134 | C110 | 115.1 |
| C61 | C19 | C18 | 121.7 |  | C92 | C134 | C133 | 115.7 |
| C61 | C19 | C20 | 122.0 |  | C133 | C134 | C110 | 120.8 |
| C16 | C20 | C19 | 109.7 |  | C93 | C135 | C136 | 118.8 |
| C16 | C20 | C21 | 122.2 |  | C106 | C135 | C93 | 115.6 |
| C19 | C20 | C21 | 116.4 |  | C106 | C135 | C136 | 113.9 |
| C20 | C21 | C22 | 116.8 |  | C97 | C136 | C101 | 118.4 |
| C25 | C21 | C20 | 124.1 |  | C97 | C136 | C135 | 119.3 |
| C25 | C21 | C22 | 107.4 |  | C101 | C136 | C135 | 114.8 |
| C21 | C22 | C14 | 120.9 |  | C98 | C137 | C102 | 106.3 |
| C23 | C22 | C14 | 114.0 |  | C138 | C137 | C98 | 118.2 |
| C23 | C22 | C21 | 108.6 |  | C138 | C137 | C102 | 127.2 |
| C22 | C23 | C24 | 108.5 |  | C122 | C138 | C78 | 113.8 |
| C22 | C23 | C48 | 120.1 |  | C122 | C138 | C137 | 122.4 |
| C48 | C23 | C24 | 119.3 |  | C137 | C138 | C78 | 116.8 |
| C25 | C24 | C23 | 106.0 |  | C77 | C139 | C121 | 117.6 |
| C63 | C24 | C23 | 115.9 |  | C77 | C139 | C140 | 121.4 |
| C63 | C24 | C25 | 129.7 |  | C121 | C139 | C140 | 113.4 |
| C21 | C25 | C24 | 109.4 |  | C119 | C140 | C71 | 115.5 |
| C62 | C25 | C21 | 120.2 |  | C119 | C140 | C139 | 119.1 |
| C62 | C25 | C24 | 121.6 |  | C139 | C140 | C71 | 117.6 |
| C27 | C26 | C30 | 108.0 |  | C151 | C141 | C165 | 119.2(6) |
| C27 | C26 | C62 | 121.8 |  | C161 | C141 | C151 | 115.4(7) |
| C30 | C26 | C62 | 125.0 |  | C161 | C141 | C165 | 122.0(7) |
| C26 | C27 | C28 | 111.0 |  | C147 | C142 | C143 | 122.6(6) |
| C26 | C27 | C63 | 125.0 |  | C147 | C142 | C168 | 116.5(6) |
| C28 | C27 | C63 | 114.0 |  | C168 | C142 | C143 | 118.5(6) |
| C27 | C28 | C29 | 105.7 |  | C145 | C143 | C142 | 119.8(6) |
| C45 | C28 | C27 | 126.3 |  | C145 | C143 | C149 | 121.0(6) |
| C45 | C28 | C29 | 120.3 |  | C149 | C143 | C142 | 116.5(6) |
| C30 | C29 | C28 | 106.5 |  | C150 | C144 | C152 | 125.2(7) |
| C40 | C29 | C28 | 121.0 |  | C150 | C144 | C168 | 121.1(6) |
| C40 | C29 | C30 | 120.9 |  | C168 | C144 | C152 | 110.5(6) |
| C26 | C30 | C29 | 108.4 |  | C143 | C145 | C146 | 120.8(6) |
| C31 | C30 | C26 | 114.7 |  | C143 | C145 | C150 | 118.9(6) |
| C31 | C30 | C29 | 120.3 |  | C146 | C145 | C150 | 118.8(7) |
| C30 | C31 | C32 | 124.2 |  | C145 | C146 | C153 | 119.0(6) |
| C30 | C31 | C35 | 116.2 |  | C145 | C146 | C165 | 118.8(7) |
| C32 | C31 | C35 | 108.7 |  | C153 | C146 | C165 | 120.3(6) |
| C31 | C32 | C33 | 108.5 |  | C142 | C147 | C155 | 121.0(6) |
| C31 | C32 | C61 | 123.3 |  | C142 | C147 | C163 | 122.4(7) |
| C33 | C32 | C61 | 118.7 |  | C163 | C147 | C155 | 113.5(7) |
| C32 | C33 | C70 | 121.0 |  | C159 | C148 | C180 | 105.7(6) |
| C34 | C33 | C32 | 106.9 |  | C182 | C148 | C159 | 120.2(7) |
| C34 | C33 | C70 | 121.4 |  | C182 | C148 | C180 | 133.8(7) |
| C33 | C34 | C35 | 109.2 |  | C143 | C149 | C157 | 118.6(6) |
| C58 | C34 | C33 | 119.8 |  | C158 | C149 | C143 | 118.6(6) |
| C58 | C34 | C35 | 126.0 |  | C158 | C149 | C157 | 118.7(6) |
| C31 | C35 | C34 | 106.5 |  | C144 | C150 | C145 | 120.4(6) |
| C36 | C35 | C31 | 122.3 |  | C144 | C150 | C170 | 114.5(7) |
| C36 | C35 | C34 | 119.0 |  | C170 | C150 | C145 | 120.4(6) |
| C35 | C36 | C37 | 118.9 |  | C141 | C151 | C184 | 124.7(7) |
| C35 | C36 | C40 | 118.8 |  | C162 | C151 | C141 | 121.4(6) |
| C37 | C36 | C40 | 107.4 |  | C162 | C151 | C184 | 109.9(8) |
| C36 | C37 | C38 | 108.3 |  | C144 | C152 | C185 | 107.9(6) |
| C36 | C37 | C59 | 119.5 |  | C173 | C152 | C144 | 119.0(6) |
| C59 | C37 | C38 | 122.9 |  | C173 | C152 | C185 | 132.6(7) |
| C39 | C38 | C37 | 107.9 |  | C162 | C153 | C146 | 119.7(6) |
| C54 | C38 | C37 | 122.3 |  | C171 | C153 | C146 | 121.7(6) |
| C54 | C38 | C39 | 116.2 |  | C171 | C153 | C162 | 114.0(8) |
| C40 | C39 | C38 | 109.3 |  | C156 | C154 | C160 | 120.1(7) |
| C40 | C39 | C41 | 114.3 |  | C161 | C154 | C156 | 115.7(7) |
| C41 | C39 | C38 | 121.5 |  | C161 | C154 | C160 | 121.1(6) |
| C29 | C40 | C36 | 121.1 |  | C157 | C155 | C147 | 117.6(7) |
| C29 | C40 | C39 | 124.0 |  | C178 | C155 | C147 | 115.7(7) |
| C39 | C40 | C36 | 106.7 |  | C178 | C155 | C157 | 124.5(6) |
| C39 | C41 | C42 | 120.9 |  | C154 | C156 | S1 | 108.1(6) |
| C39 | C41 | C45 | 125.2 |  | C154 | C156 | C182 | 121.7(6) |
| C42 | C41 | C45 | 108.6 |  | C182 | C156 | S1 | 128.6(6) |
| C41 | C42 | C55 | 120.8 |  | C155 | C157 | C149 | 123.5(6) |
| C43 | C42 | C41 | 108.4 |  | C155 | C157 | C187 | 113.6(7) |
| C43 | C42 | C55 | 114.9 |  | C187 | C157 | C149 | 120.9(6) |
| C42 | C43 | C44 | 109.9 |  | C149 | C158 | C160 | 118.4(6) |
| C42 | C43 | C65 | 120.1 |  | C165 | C158 | C149 | 119.9(6) |
| C65 | C43 | C44 | 119.8 |  | C165 | C158 | C160 | 119.9(6) |
| C43 | C44 | C45 | 105.2 |  | C148 | C159 | C187 | 110.5(7) |
| C64 | C44 | C43 | 133.2 |  | C160 | C159 | C148 | 124.6(7) |
| C64 | C44 | C45 | 112.2 |  | C160 | C159 | C187 | 120.1(6) |
| C28 | C45 | C41 | 114.5 |  | C154 | C160 | C158 | 119.1(6) |
| C28 | C45 | C44 | 121.9 |  | C159 | C160 | C154 | 116.2(6) |
| C41 | C45 | C44 | 107.3 |  | C159 | C160 | C158 | 122.1(6) |
| C47 | C46 | C65 | 121.0 |  | C141 | C161 | C154 | 120.1(6) |
| C50 | C46 | C47 | 107.8 |  | C141 | C161 | C172 | 123.6(7) |
| C50 | C46 | C65 | 120.7 |  | C154 | C161 | C172 | 114.2(7) |
| C46 | C47 | C64 | 123.1 |  | C151 | C162 | C153 | 120.7(7) |
| C48 | C47 | C46 | 105.5 |  | C181 | C162 | C151 | 111.8(7) |
| C48 | C47 | C64 | 115.6 |  | C181 | C162 | C153 | 124.8(7) |
| C47 | C48 | C23 | 121.9 |  | C147 | C163 | S3 | 108.0(5) |
| C49 | C48 | C23 | 122.2 |  | C147 | C163 | C175 | 121.2(7) |
| C49 | C48 | C47 | 108.8 |  | C175 | C163 | S3 | 129.4(6) |
| C13 | C49 | C48 | 119.3 |  | C183 | C164 | C181 | 115.6(8) |
| C13 | C49 | C50 | 118.4 |  | C146 | C165 | C141 | 118.6(7) |
| C50 | C49 | C48 | 109.4 |  | C158 | C165 | C141 | 117.5(6) |
| C8 | C50 | C46 | 119.6 |  | C158 | C165 | C146 | 120.7(6) |
| C49 | C50 | C8 | 120.0 |  | C166 | C167 | C181 | 113.5(7) |
| C49 | C50 | C46 | 108.2 |  | C166 | C167 | C184 | 113.3(7) |
| C52 | C51 | C55 | 109.4 |  | C184 | C167 | C181 | 99.7(6) |
| C52 | C51 | C66 | 121.4 |  | C191 | C167 | C166 | 112.3(7) |
| C55 | C51 | C66 | 111.7 |  | C191 | C167 | C181 | 109.0(7) |
| C51 | C52 | C53 | 109.6 |  | C191 | C167 | C184 | 108.3(7) |
| C51 | C52 | C67 | 124.1 |  | C142 | C168 | C144 | 121.0(6) |
| C53 | C52 | C67 | 116.2 |  | C142 | C168 | C169 | 123.6(7) |
| C52 | C53 | C54 | 106.8 |  | C144 | C168 | C169 | 110.7(7) |
| C60 | C53 | C52 | 121.3 |  | C168 | C169 | C185 | 107.1(6) |
| C60 | C53 | C54 | 121.4 |  | C175 | C169 | C168 | 118.8(7) |
| C38 | C54 | C53 | 115.2 |  | C175 | C169 | C185 | 133.7(7) |
| C38 | C54 | C55 | 126.4 |  | C150 | C170 | C171 | 119.7(7) |
| C53 | C54 | C55 | 108.4 |  | C150 | C170 | C179 | 123.2(6) |
| C42 | C55 | C51 | 130.5 |  | C179 | C170 | C171 | 114.7(8) |
| C42 | C55 | C54 | 113.1 |  | C153 | C171 | C170 | 119.6(7) |
| C51 | C55 | C54 | 105.6 |  | C153 | C171 | C183 | 125.0(6) |
| C57 | C56 | C60 | 108.6 |  | C183 | C171 | C170 | 113.2(7) |
| C68 | C56 | C57 | 120.9 |  | C161 | C172 | S1 | 108.5(6) |
| C68 | C56 | C60 | 120.3 |  | C189 | C172 | S1 | 131.3(6) |
| C56 | C57 | C58 | 108.1 |  | C189 | C172 | C161 | 119.4(7) |
| C69 | C57 | C56 | 122.8 |  | C152 | C173 | C179 | 117.5(7) |
| C69 | C57 | C58 | 121.2 |  | C169 | C175 | C163 | 117.2(7) |
| C34 | C58 | C57 | 123.0 |  | C190 | C177 | C178 | 117.7(7) |
| C34 | C58 | C59 | 113.6 |  | C155 | C178 | S3 | 108.8(5) |
| C57 | C58 | C59 | 106.8 |  | C155 | C178 | C177 | 120.0(7) |
| C37 | C59 | C58 | 122.5 |  | C177 | C178 | S3 | 129.9(7) |
| C37 | C59 | C60 | 115.0 |  | C170 | C179 | S2 | 108.6(5) |
| C60 | C59 | C58 | 109.3 |  | C170 | C179 | C173 | 120.5(7) |
| C53 | C60 | C56 | 122.5 |  | C173 | C179 | S2 | 129.8(7) |
| C53 | C60 | C59 | 122.9 |  | C176 | C180 | C148 | 113.0(7) |
| C59 | C60 | C56 | 107.0 |  | C176 | C180 | C190 | 111.5(6) |
| C19 | C61 | C32 | 119.5 |  | C186 | C180 | C148 | 112.1(7) |
| C19 | C61 | C62 | 121.1 |  | C186 | C180 | C176 | 109.9(7) |
| C32 | C61 | C62 | 112.9 |  | C186 | C180 | C190 | 108.4(6) |
| C25 | C62 | C26 | 113.7 |  | C190 | C180 | C148 | 101.7(5) |
| C25 | C62 | C61 | 115.6 |  | C162 | C181 | C164 | 121.2(7) |
| C26 | C62 | C61 | 118.7 |  | C162 | C181 | C167 | 107.4(7) |
| C24 | C63 | C27 | 106.7 |  | C164 | C181 | C167 | 130.4(8) |
| C24 | C63 | C64 | 129.2 |  | C148 | C182 | C156 | 116.8(7) |
| C64 | C63 | C27 | 116.5 |  | C164 | C183 | S2 | 130.1(7) |
| C44 | C64 | C47 | 106.8 |  | C171 | C183 | S2 | 109.3(5) |
| C63 | C64 | C44 | 128.1 |  | C171 | C183 | C164 | 119.3(7) |
| C63 | C64 | C47 | 117.4 |  | C151 | C184 | C167 | 107.5(6) |
| C43 | C65 | C46 | 112.5 |  | C151 | C184 | C189 | 117.2(7) |
| C66 | C65 | C43 | 129.1 |  | C189 | C184 | C167 | 134.9(8) |
| C66 | C65 | C46 | 108.3 |  | C152 | C185 | C169 | 100.4(5) |
| C9 | C66 | C51 | 109.7 |  | C174 | C185 | C152 | 115.2(7) |
| C9 | C66 | C65 | 133.8 |  | C174 | C185 | C169 | 112.3(6) |
| C65 | C66 | C51 | 112.5 |  | C174 | C185 | C188 | 110.5(6) |
| C10 | C67 | C52 | 107.4 |  | C188 | C185 | C152 | 109.7(6) |
| C10 | C67 | C68 | 121.9 |  | C188 | C185 | C169 | 108.0(7) |
| C68 | C67 | C52 | 120.5 |  | C157 | C187 | C159 | 119.5(7) |
| C2 | C68 | C56 | 117.0 |  | C190 | C187 | C157 | 123.7(7) |
| C2 | C68 | C67 | 117.2 |  | C190 | C187 | C159 | 112.5(7) |
| C56 | C68 | C67 | 119.0 |  | C172 | C189 | C184 | 119.5(7) |
| C3 | C69 | C57 | 115.2 |  | C177 | C190 | C180 | 134.1(8) |
| C3 | C69 | C70 | 118.8 |  | C177 | C190 | C187 | 120.3(6) |
| C57 | C69 | C70 | 117.0 |  | C187 | C190 | C180 | 105.0(6) |
| C18 | C70 | C33 | 116.8 |  | C201 | C192 | C198 | 119.9(7) |
| C18 | C70 | C69 | 117.0 |  | C208 | C192 | C198 | 123.8(8) |
| C69 | C70 | C33 | 116.1 |  | C208 | C192 | C201 | 113.9(7) |
| C72 | C71 | C140 | 118.4 |  | C210 | C193 | C209 | 120.1(7) |
| C75 | C71 | C72 | 107.8 |  | C210 | C193 | C218 | 114.8(6) |
| C75 | C71 | C140 | 122.9 |  | C218 | C193 | C209 | 121.1(6) |
| C71 | C72 | C73 | 107.7 |  | C197 | C194 | C198 | 119.9(6) |
| C76 | C72 | C71 | 117.3 |  | C197 | C194 | C203 | 119.8(6) |
| C76 | C72 | C73 | 121.5 |  | C203 | C194 | C198 | 118.1(7) |
| C74 | C73 | C72 | 106.4 |  | C212 | C195 | C199 | 120.9(7) |
| C82 | C73 | C72 | 117.5 |  | C212 | C195 | C215 | 115.8(7) |
| C82 | C73 | C74 | 126.6 |  | C215 | C195 | C199 | 120.0(6) |
| C73 | C74 | C90 | 106.6 |  | C218 | C196 | S5 | 108.3(5) |
| C75 | C74 | C73 | 107.1 |  | C218 | C196 | C225 | 121.1(7) |
| C75 | C74 | C90 | 134.0 |  | C225 | C196 | S5 | 129.1(6) |
| C71 | C75 | C74 | 111.0 |  | C194 | C197 | C202 | 117.4(7) |
| C71 | C75 | C131 | 115.1 |  | C206 | C197 | C194 | 120.1(6) |
| C74 | C75 | C131 | 121.8 |  | C206 | C197 | C202 | 120.6(7) |
| C72 | C76 | C77 | 126.8 |  | C192 | C198 | C194 | 120.3(7) |
| C72 | C76 | C80 | 116.5 |  | C200 | C198 | C192 | 113.7(7) |
| C77 | C76 | C80 | 107.1 |  | C200 | C198 | C194 | 121.4(6) |
| C76 | C77 | C78 | 107.7 |  | C206 | C199 | C195 | 119.4(7) |
| C139 | C77 | C76 | 118.1 |  | C209 | C199 | C195 | 118.7(6) |
| C139 | C77 | C78 | 123.3 |  | C209 | C199 | C206 | 119.4(6) |
| C77 | C78 | C138 | 118.6 |  | C198 | C200 | C220 | 119.2(6) |
| C79 | C78 | C77 | 107.3 |  | C205 | C200 | C198 | 126.0(7) |
| C79 | C78 | C138 | 127.8 |  | C205 | C200 | C220 | 111.2(7) |
| C78 | C79 | C80 | 110.5 |  | C192 | C201 | C202 | 120.4(6) |
| C78 | C79 | C99 | 110.1 |  | C192 | C201 | C226 | 115.0(7) |
| C99 | C79 | C80 | 122.1 |  | C202 | C201 | C226 | 122.0(7) |
| C79 | C80 | C76 | 107.1 |  | C201 | C202 | C197 | 121.6(7) |
| C81 | C80 | C76 | 120.0 |  | C211 | C202 | C197 | 119.3(7) |
| C81 | C80 | C79 | 124.7 |  | C211 | C202 | C201 | 116.0(7) |
| C80 | C81 | C82 | 125.6 |  | C194 | C203 | C213 | 118.8(6) |
| C80 | C81 | C85 | 119.0 |  | C209 | C203 | C194 | 119.4(7) |
| C82 | C81 | C85 | 106.6 |  | C209 | C203 | C213 | 119.2(6) |
| C73 | C82 | C81 | 118.5 |  | C214 | C204 | C206 | 122.1(7) |
| C73 | C82 | C83 | 115.9 |  | C214 | C204 | C234 | 114.6(8) |
| C81 | C82 | C83 | 110.9 |  | C234 | C204 | C206 | 121.0(7) |
| C82 | C83 | C84 | 105.8 |  | C200 | C205 | C223 | 107.3(6) |
| C86 | C83 | C82 | 128.2 |  | C200 | C205 | C228 | 119.8(7) |
| C86 | C83 | C84 | 112.1 |  | C228 | C205 | C223 | 132.3(7) |
| C85 | C84 | C83 | 108.3 |  | C197 | C206 | C199 | 120.9(7) |
| C95 | C84 | C83 | 119.5 |  | C197 | C206 | C204 | 118.1(7) |
| C95 | C84 | C85 | 119.6 |  | C199 | C206 | C204 | 118.2(7) |
| C84 | C85 | C81 | 108.1 |  | C213 | C207 | C218 | 119.1(7) |
| C84 | C85 | C100 | 118.0 |  | C219 | C207 | C213 | 122.7(7) |
| C100 | C85 | C81 | 116.1 |  | C219 | C207 | C218 | 115.6(6) |
| C83 | C86 | C87 | 130.5 |  | C192 | C208 | S4 | 109.6(6) |
| C83 | C86 | C90 | 114.1 |  | C192 | C208 | C228 | 120.8(7) |
| C90 | C86 | C87 | 104.4 |  | C228 | C208 | S4 | 128.7(6) |
| C88 | C87 | C86 | 108.3 |  | C199 | C209 | C193 | 118.7(6) |
| C91 | C87 | C86 | 110.4 |  | C199 | C209 | C203 | 120.5(6) |
| C91 | C87 | C88 | 130.2 |  | C203 | C209 | C193 | 118.4(7) |
| C87 | C88 | C89 | 107.6 |  | C193 | C210 | C221 | 125.3(7) |
| C87 | C88 | C133 | 121.9 |  | C212 | C210 | C193 | 120.3(6) |
| C133 | C88 | C89 | 122.1 |  | C212 | C210 | C221 | 110.0(7) |
| C88 | C89 | C132 | 119.9 |  | C202 | C211 | C229 | 124.7(7) |
| C90 | C89 | C88 | 106.7 |  | C202 | C211 | C234 | 121.4(7) |
| C90 | C89 | C132 | 124.0 |  | C229 | C211 | C234 | 110.3(8) |
| C86 | C90 | C74 | 127.5 |  | C195 | C212 | C210 | 121.3(6) |
| C89 | C90 | C74 | 104.8 |  | C195 | C212 | C217 | 125.0(7) |
| C89 | C90 | C86 | 112.9 |  | C210 | C212 | C217 | 110.5(7) |
| C87 | C91 | C92 | 111.8 |  | C207 | C213 | C203 | 121.3(6) |
| C87 | C91 | C95 | 130.1 |  | C220 | C213 | C203 | 121.2(6) |
| C95 | C91 | C92 | 107.2 |  | C220 | C213 | C207 | 114.5(7) |
| C93 | C92 | C91 | 106.0 |  | C204 | C214 | C215 | 119.9(8) |
| C93 | C92 | C134 | 119.0 |  | C204 | C214 | C216 | 123.4(7) |
| C134 | C92 | C91 | 126.3 |  | C216 | C214 | C215 | 114.4(8) |
| C92 | C93 | C94 | 108.7 |  | C214 | C215 | C195 | 120.3(7) |
| C92 | C93 | C135 | 124.9 |  | C237 | C215 | C195 | 123.5(8) |
| C94 | C93 | C135 | 117.5 |  | C237 | C215 | C214 | 113.5(8) |
| C95 | C94 | C93 | 110.9 |  | C214 | C216 | S6 | 109.2(7) |
| C95 | C94 | C96 | 120.1 |  | C214 | C216 | C238 | 119.3(8) |
| C96 | C94 | C93 | 118.0 |  | C238 | C216 | S6 | 131.2(8) |
| C84 | C95 | C91 | 116.9 |  | C212 | C217 | C222 | 108.8(7) |
| C84 | C95 | C94 | 124.9 |  | C212 | C217 | C242 | 118.4(7) |
| C94 | C95 | C91 | 107.2 |  | C242 | C217 | C222 | 131.0(7) |
| C94 | C96 | C97 | 124.6 |  | C193 | C218 | C207 | 120.6(6) |
| C94 | C96 | C100 | 115.6 |  | C196 | C218 | C193 | 122.6(7) |
| C97 | C96 | C100 | 107.7 |  | C196 | C218 | C207 | 114.2(7) |
| C96 | C97 | C98 | 109.8 |  | C207 | C219 | S5 | 108.1(5) |
| C136 | C97 | C96 | 120.9 |  | C207 | C219 | C224 | 122.4(7) |
| C136 | C97 | C98 | 120.9 |  | C224 | C219 | S5 | 128.8(6) |
| C99 | C98 | C97 | 106.1 |  | C213 | C220 | C200 | 121.2(7) |
| C137 | C98 | C97 | 127.8 |  | C213 | C220 | C230 | 124.8(7) |
| C137 | C98 | C99 | 119.3 |  | C230 | C220 | C200 | 110.7(7) |
| C79 | C99 | C98 | 127.0 |  | C210 | C221 | C222 | 108.4(7) |
| C79 | C99 | C100 | 113.2 |  | C225 | C221 | C210 | 118.9(7) |
| C98 | C99 | C100 | 109.7 |  | C225 | C221 | C222 | 132.0(7) |
| C96 | C100 | C85 | 121.6 |  | C217 | C222 | C221 | 99.8(5) |
| C96 | C100 | C99 | 106.6 |  | C217 | C222 | C227 | 109.6(7) |
| C99 | C100 | C85 | 123.5 |  | C221 | C222 | C227 | 107.1(7) |
| C105 | C101 | C102 | 108.6 |  | C239 | C222 | C217 | 115.3(8) |
| C105 | C101 | C136 | 122.7 |  | C239 | C222 | C221 | 115.3(7) |
| C136 | C101 | C102 | 118.0 |  | C239 | C222 | C227 | 109.2(6) |
| C103 | C102 | C101 | 108.6 |  | C205 | C223 | C230 | 99.9(5) |
| C103 | C102 | C137 | 110.6 |  | C231 | C223 | C205 | 113.3(7) |
| C137 | C102 | C101 | 126.0 |  | C231 | C223 | C230 | 113.7(7) |
| C102 | C103 | C104 | 108.1 |  | C231 | C223 | C233 | 110.3(7) |
| C102 | C103 | C123 | 122.9 |  | C233 | C223 | C205 | 109.1(7) |
| C104 | C103 | C123 | 117.5 |  | C233 | C223 | C230 | 110.0(6) |
| C103 | C104 | C105 | 106.5 |  | C230 | C224 | C219 | 114.6(7) |
| C130 | C104 | C103 | 124.7 |  | C221 | C225 | C196 | 117.2(7) |
| C130 | C104 | C105 | 119.7 |  | C201 | C226 | S4 | 108.1(6) |
| C101 | C105 | C104 | 108.1 |  | C235 | C226 | S4 | 131.0(7) |
| C107 | C105 | C101 | 122.8 |  | C235 | C226 | C201 | 119.3(7) |
| C107 | C105 | C104 | 119.5 |  | C205 | C228 | C208 | 115.9(7) |
| C110 | C106 | C107 | 107.2 |  | C211 | C229 | C232 | 106.7(7) |
| C135 | C106 | C107 | 125.1 |  | C235 | C229 | C211 | 118.4(8) |
| C135 | C106 | C110 | 121.8 |  | C235 | C229 | C232 | 133.9(8) |
| C105 | C107 | C106 | 120.2 |  | C220 | C230 | C223 | 107.2(6) |
| C105 | C107 | C108 | 120.6 |  | C220 | C230 | C224 | 120.9(6) |
| C106 | C107 | C108 | 107.4 |  | C224 | C230 | C223 | 130.8(7) |
| C109 | C108 | C107 | 106.7 |  | C229 | C232 | C240 | 101.6(6) |
| C129 | C108 | C107 | 124.6 |  | C236 | C232 | C229 | 115.2(8) |
| C129 | C108 | C109 | 118.5 |  | C236 | C232 | C240 | 114.5(9) |
| C110 | C109 | C108 | 109.2 |  | C236 | C232 | C241 | 107.3(8) |
| C111 | C109 | C108 | 124.7 |  | C241 | C232 | C229 | 110.8(9) |
| C111 | C109 | C110 | 114.0 |  | C241 | C232 | C240 | 107.1(7) |
| C106 | C110 | C134 | 122.9 |  | C204 | C234 | C211 | 119.6(8) |
| C109 | C110 | C106 | 109.4 |  | C240 | C234 | C204 | 125.3(8) |
| C109 | C110 | C134 | 118.2 |  | C240 | C234 | C211 | 111.6(7) |
| C109 | C111 | C112 | 129.9 |  | C229 | C235 | C226 | 119.6(8) |
| C109 | C111 | C115 | 112.9 |  | C215 | C237 | S6 | 109.7(6) |
| C115 | C111 | C112 | 107.1 |  | C215 | C237 | C242 | 119.8(8) |
| C113 | C112 | C111 | 108.5 |  | C242 | C237 | S6 | 128.7(7) |
| C133 | C112 | C111 | 113.1 |  | C240 | C238 | C216 | 118.4(8) |
| C133 | C112 | C113 | 131.0 |  | C234 | C240 | C232 | 105.9(8) |
| C112 | C113 | C114 | 108.5 |  | C234 | C240 | C238 | 118.8(8) |
| C112 | C113 | C132 | 120.3 |  | C238 | C240 | C232 | 134.5(9) |
| C132 | C113 | C114 | 123.4 |  | C237 | C242 | C217 | 117.4(8) |

Table 6 Torsion Angles for z\_sq\_tw.

| A | B | C | D | Angle/˚ |  | A | B | C | D | Angle/˚ |
| --- | --- | --- | --- | --- | --- | --- | --- | --- | --- | --- |
| S1 | C156 | C182 | C148 | -164.6(7) |  | C106 | C110 | C134 | C133 | -137.3 |
| S1 | C172 | C189 | C184 | 164.2(6) |  | C106 | C135 | C136 | C97 | -142.7 |
| S3 | C163 | C175 | C169 | -164.3(6) |  | C106 | C135 | C136 | C101 | 6.5 |
| S4 | C208 | C228 | C205 | 167.1(6) |  | C107 | C106 | C110 | C109 | 3.1 |
| S4 | C226 | C235 | C229 | -165.1(7) |  | C107 | C106 | C110 | C134 | 148.7 |
| S5 | C196 | C218 | C193 | -167.7(7) |  | C107 | C106 | C135 | C93 | -151.6 |
| S5 | C196 | C218 | C207 | -5.9(9) |  | C107 | C106 | C135 | C136 | -8.9 |
| S5 | C196 | C225 | C221 | 166.3(7) |  | C107 | C108 | C109 | C110 | 0.8 |
| S5 | C219 | C224 | C230 | -167.6(7) |  | C107 | C108 | C109 | C111 | -139.3 |
| S6 | C216 | C238 | C240 | 168.6(8) |  | C107 | C108 | C129 | C128 | 135.2 |
| S6 | C237 | C242 | C217 | -165.9(7) |  | C107 | C108 | C129 | C130 | -3.6 |
| C1 | C2 | C3 | C4 | -2.0 |  | C108 | C109 | C110 | C106 | -2.5 |
| C1 | C2 | C3 | C69 | -146.5 |  | C108 | C109 | C110 | C134 | -149.8 |
| C1 | C2 | C68 | C56 | 150.8 |  | C108 | C109 | C111 | C112 | 133.5 |
| C1 | C2 | C68 | C67 | 0.0 |  | C108 | C109 | C111 | C115 | -6.7 |
| C1 | C5 | C11 | C12 | 0.7 |  | C108 | C129 | C130 | C104 | 6.8 |
| C1 | C5 | C11 | C15 | -135.9 |  | C108 | C129 | C130 | C126 | 138.5 |
| C1 | C6 | C7 | C8 | 143.0 |  | C109 | C108 | C129 | C128 | -5.3 |
| C1 | C6 | C7 | C12 | 2.2 |  | C109 | C108 | C129 | C130 | -144.1 |
| C1 | C6 | C10 | C9 | -138.8 |  | C109 | C110 | C134 | C92 | 152.3 |
| C1 | C6 | C10 | C67 | 8.2 |  | C109 | C110 | C134 | C133 | 5.4 |
| C2 | C1 | C5 | C4 | 5.4 |  | C109 | C111 | C112 | C113 | -138.9 |
| C2 | C1 | C5 | C11 | 145.1 |  | C109 | C111 | C112 | C133 | 14.6 |
| C2 | C1 | C6 | C7 | -142.9 |  | C109 | C111 | C115 | C114 | 145.0 |
| C2 | C1 | C6 | C10 | -3.6 |  | C109 | C111 | C115 | C128 | 4.1 |
| C2 | C3 | C4 | C5 | 5.3 |  | C110 | C106 | C107 | C105 | -145.6 |
| C2 | C3 | C4 | C17 | -145.9 |  | C110 | C106 | C107 | C108 | -2.6 |
| C2 | C3 | C69 | C57 | -8.5 |  | C110 | C106 | C135 | C93 | -1.9 |
| C2 | C3 | C69 | C70 | 137.7 |  | C110 | C106 | C135 | C136 | 140.8 |
| C3 | C2 | C68 | C56 | 5.9 |  | C110 | C109 | C111 | C112 | -4.9 |
| C3 | C2 | C68 | C67 | -144.9 |  | C110 | C109 | C111 | C115 | -145.1 |
| C3 | C4 | C5 | C1 | -6.7 |  | C111 | C109 | C110 | C106 | 142.3 |
| C3 | C4 | C5 | C11 | -146.0 |  | C111 | C109 | C110 | C134 | -5.1 |
| C3 | C4 | C17 | C16 | 142.1 |  | C111 | C112 | C113 | C114 | -0.7 |
| C3 | C4 | C17 | C18 | 6.1 |  | C111 | C112 | C113 | C132 | 149.5 |
| C3 | C69 | C70 | C18 | 0.1 |  | C111 | C112 | C133 | C88 | -154.4 |
| C3 | C69 | C70 | C33 | -144.3 |  | C111 | C112 | C133 | C134 | -13.4 |
| C4 | C3 | C69 | C57 | -148.9 |  | C111 | C115 | C128 | C127 | 133.2 |
| C4 | C3 | C69 | C70 | -2.6 |  | C111 | C115 | C128 | C129 | -2.3 |
| C4 | C5 | C11 | C12 | 135.7 |  | C112 | C111 | C115 | C114 | -4.1 |
| C4 | C5 | C11 | C15 | -1.0 |  | C112 | C111 | C115 | C128 | -145.0 |
| C4 | C17 | C18 | C19 | 139.0 |  | C112 | C113 | C114 | C115 | -1.8 |
| C4 | C17 | C18 | C70 | -8.6 |  | C112 | C113 | C114 | C116 | 142.0 |
| C5 | C1 | C2 | C3 | -2.2 |  | C112 | C113 | C132 | C89 | 6.7 |
| C5 | C1 | C2 | C68 | -150.8 |  | C112 | C113 | C132 | C131 | -141.4 |
| C5 | C1 | C6 | C7 | 1.6 |  | C112 | C133 | C134 | C92 | -141.6 |
| C5 | C1 | C6 | C10 | 140.9 |  | C112 | C133 | C134 | C110 | 5.1 |
| C5 | C4 | C17 | C16 | -4.6 |  | C113 | C112 | C133 | C88 | -8.4 |
| C5 | C4 | C17 | C18 | -140.6 |  | C113 | C112 | C133 | C134 | 132.5 |
| C5 | C11 | C12 | C7 | 3.3 |  | C113 | C114 | C115 | C111 | 3.6 |
| C5 | C11 | C12 | C13 | -143.6 |  | C113 | C114 | C115 | C128 | 146.4 |
| C5 | C11 | C15 | C14 | 142.5 |  | C113 | C114 | C116 | C117 | -142.6 |
| C5 | C11 | C15 | C16 | -1.9 |  | C113 | C114 | C116 | C120 | 2.8 |
| C6 | C1 | C2 | C3 | 147.8 |  | C114 | C113 | C132 | C89 | 152.3 |
| C6 | C1 | C2 | C68 | -0.8 |  | C114 | C113 | C132 | C131 | 4.2 |
| C6 | C1 | C5 | C4 | -142.9 |  | C114 | C115 | C128 | C127 | -4.2 |
| C6 | C1 | C5 | C11 | -3.2 |  | C114 | C115 | C128 | C129 | -139.7 |
| C6 | C7 | C8 | C9 | 0.3 |  | C114 | C116 | C117 | C118 | 137.9 |
| C6 | C7 | C8 | C50 | -141.8 |  | C114 | C116 | C117 | C127 | 7.3 |
| C6 | C7 | C12 | C11 | -4.8 |  | C114 | C116 | C120 | C119 | -145.5 |
| C6 | C7 | C12 | C13 | 138.6 |  | C114 | C116 | C120 | C131 | 3.6 |
| C6 | C10 | C67 | C52 | -154.1 |  | C115 | C111 | C112 | C113 | 2.9 |
| C6 | C10 | C67 | C68 | -8.8 |  | C115 | C111 | C112 | C133 | 156.5 |
| C7 | C6 | C10 | C9 | 2.8 |  | C115 | C114 | C116 | C117 | -2.7 |
| C7 | C6 | C10 | C67 | 149.8 |  | C115 | C114 | C116 | C120 | 142.8 |
| C7 | C8 | C9 | C10 | 1.5 |  | C115 | C128 | C129 | C108 | 3.0 |
| C7 | C8 | C9 | C66 | -146.5 |  | C115 | C128 | C129 | C130 | 145.3 |
| C7 | C8 | C50 | C46 | 139.7 |  | C116 | C114 | C115 | C111 | -141.5 |
| C7 | C8 | C50 | C49 | 1.6 |  | C116 | C114 | C115 | C128 | 1.3 |
| C7 | C12 | C13 | C14 | -145.4 |  | C116 | C117 | C118 | C119 | 12.0 |
| C7 | C12 | C13 | C49 | -2.3 |  | C116 | C117 | C118 | C125 | -143.7 |
| C8 | C7 | C12 | C11 | -141.4 |  | C116 | C117 | C127 | C126 | 133.8 |
| C8 | C7 | C12 | C13 | 2.1 |  | C116 | C117 | C127 | C128 | -9.4 |
| C8 | C9 | C10 | C6 | -2.7 |  | C116 | C120 | C131 | C75 | -152.5 |
| C8 | C9 | C10 | C67 | -144.1 |  | C116 | C120 | C131 | C132 | -6.1 |
| C8 | C9 | C66 | C51 | 158.9 |  | C117 | C116 | C120 | C119 | 5.8 |
| C8 | C9 | C66 | C65 | 3.7 |  | C117 | C116 | C120 | C131 | 154.9 |
| C9 | C8 | C50 | C46 | 1.3 |  | C117 | C118 | C119 | C120 | -8.2 |
| C9 | C8 | C50 | C49 | -136.8 |  | C117 | C118 | C119 | C140 | -152.9 |
| C9 | C10 | C67 | C52 | -15.6 |  | C117 | C118 | C125 | C121 | 141.2 |
| C9 | C10 | C67 | C68 | 129.8 |  | C117 | C118 | C125 | C124 | 10.9 |
| C10 | C6 | C7 | C8 | -2.0 |  | C117 | C127 | C128 | C115 | 8.6 |
| C10 | C6 | C7 | C12 | -142.8 |  | C117 | C127 | C128 | C129 | 149.1 |
| C10 | C9 | C66 | C51 | 17.1 |  | C118 | C117 | C127 | C126 | 3.8 |
| C10 | C9 | C66 | C65 | -138.0 |  | C118 | C117 | C127 | C128 | -139.4 |
| C10 | C67 | C68 | C2 | 5.0 |  | C118 | C119 | C120 | C116 | 1.3 |
| C10 | C67 | C68 | C56 | -145.1 |  | C118 | C119 | C120 | C131 | -148.7 |
| C11 | C12 | C13 | C14 | 3.2 |  | C118 | C119 | C140 | C71 | 150.9 |
| C11 | C12 | C13 | C49 | 146.2 |  | C118 | C119 | C140 | C139 | 2.6 |
| C11 | C15 | C16 | C17 | 1.6 |  | C119 | C118 | C125 | C121 | -12.0 |
| C11 | C15 | C16 | C20 | 140.0 |  | C119 | C118 | C125 | C124 | -142.3 |
| C12 | C7 | C8 | C9 | 140.2 |  | C119 | C120 | C131 | C75 | -6.8 |
| C12 | C7 | C8 | C50 | -1.8 |  | C119 | C120 | C131 | C132 | 139.6 |
| C12 | C11 | C15 | C14 | 1.8 |  | C120 | C116 | C117 | C118 | -11.4 |
| C12 | C11 | C15 | C16 | -142.6 |  | C120 | C116 | C117 | C127 | -142.0 |
| C12 | C13 | C14 | C15 | -2.0 |  | C120 | C119 | C140 | C71 | 10.5 |
| C12 | C13 | C14 | C22 | 143.9 |  | C120 | C119 | C140 | C139 | -137.8 |
| C12 | C13 | C49 | C48 | -135.2 |  | C120 | C131 | C132 | C89 | -139.8 |
| C12 | C13 | C49 | C50 | 2.1 |  | C120 | C131 | C132 | C113 | 2.0 |
| C13 | C14 | C15 | C11 | 0.2 |  | C121 | C122 | C123 | C103 | 139.1 |
| C13 | C14 | C15 | C16 | 143.9 |  | C121 | C122 | C123 | C124 | -0.9 |
| C13 | C14 | C22 | C21 | -135.1 |  | C121 | C122 | C138 | C78 | 13.6 |
| C13 | C14 | C22 | C23 | -2.8 |  | C121 | C122 | C138 | C137 | -135.9 |
| C13 | C49 | C50 | C8 | -1.8 |  | C121 | C139 | C140 | C71 | -152.8 |
| C13 | C49 | C50 | C46 | -144.1 |  | C121 | C139 | C140 | C119 | -5.0 |
| C14 | C13 | C49 | C48 | 4.5 |  | C122 | C121 | C125 | C118 | -136.9 |
| C14 | C13 | C49 | C50 | 141.8 |  | C122 | C121 | C125 | C124 | -3.3 |
| C14 | C15 | C16 | C17 | -136.7 |  | C122 | C121 | C139 | C77 | -8.8 |
| C14 | C15 | C16 | C20 | 1.7 |  | C122 | C121 | C139 | C140 | 141.4 |
| C14 | C22 | C23 | C24 | -139.5 |  | C122 | C123 | C124 | C125 | -1.3 |
| C14 | C22 | C23 | C48 | 2.9 |  | C122 | C123 | C124 | C126 | 144.2 |
| C15 | C11 | C12 | C7 | 143.8 |  | C123 | C103 | C104 | C105 | 142.0 |
| C15 | C11 | C12 | C13 | -3.1 |  | C123 | C103 | C104 | C130 | -4.4 |
| C15 | C14 | C22 | C21 | 8.8 |  | C123 | C122 | C138 | C78 | 152.4 |
| C15 | C14 | C22 | C23 | 141.1 |  | C123 | C122 | C138 | C137 | 3.0 |
| C15 | C16 | C17 | C4 | 1.6 |  | C123 | C124 | C125 | C118 | 140.1 |
| C15 | C16 | C17 | C18 | 143.8 |  | C123 | C124 | C125 | C121 | 2.9 |
| C15 | C16 | C20 | C19 | -141.9 |  | C123 | C124 | C126 | C127 | -139.3 |
| C15 | C16 | C20 | C21 | -0.4 |  | C123 | C124 | C126 | C130 | 1.3 |
| C16 | C17 | C18 | C19 | -1.3 |  | C124 | C126 | C127 | C117 | -1.5 |
| C16 | C17 | C18 | C70 | -148.9 |  | C124 | C126 | C127 | C128 | 144.0 |
| C16 | C20 | C21 | C22 | 3.3 |  | C124 | C126 | C130 | C104 | -4.3 |
| C16 | C20 | C21 | C25 | -135.0 |  | C124 | C126 | C130 | C129 | -137.9 |
| C17 | C4 | C5 | C1 | 143.7 |  | C125 | C118 | C119 | C120 | 151.2 |
| C17 | C4 | C5 | C11 | 4.4 |  | C125 | C118 | C119 | C140 | 6.5 |
| C17 | C16 | C20 | C19 | 0.5 |  | C125 | C121 | C122 | C123 | 2.6 |
| C17 | C16 | C20 | C21 | 142.0 |  | C125 | C121 | C122 | C138 | 142.1 |
| C17 | C18 | C19 | C20 | 1.6 |  | C125 | C121 | C139 | C77 | -150.4 |
| C17 | C18 | C19 | C61 | -144.8 |  | C125 | C121 | C139 | C140 | -0.2 |
| C17 | C18 | C70 | C33 | 149.4 |  | C125 | C124 | C126 | C127 | 2.7 |
| C17 | C18 | C70 | C69 | 5.2 |  | C125 | C124 | C126 | C130 | 143.2 |
| C18 | C19 | C20 | C16 | -1.2 |  | C126 | C124 | C125 | C118 | -6.8 |
| C18 | C19 | C20 | C21 | -145.3 |  | C126 | C124 | C125 | C121 | -144.0 |
| C18 | C19 | C61 | C32 | -10.0 |  | C126 | C127 | C128 | C115 | -140.7 |
| C18 | C19 | C61 | C62 | 139.6 |  | C126 | C127 | C128 | C129 | -0.2 |
| C19 | C18 | C70 | C33 | 5.6 |  | C127 | C117 | C118 | C119 | 146.2 |
| C19 | C18 | C70 | C69 | -138.5 |  | C127 | C117 | C118 | C125 | -9.5 |
| C19 | C20 | C21 | C22 | 142.5 |  | C127 | C126 | C130 | C104 | 139.0 |
| C19 | C20 | C21 | C25 | 4.2 |  | C127 | C126 | C130 | C129 | 5.5 |
| C19 | C61 | C62 | C25 | -2.7 |  | C127 | C128 | C129 | C108 | -138.8 |
| C19 | C61 | C62 | C26 | -143.2 |  | C127 | C128 | C129 | C130 | 3.5 |
| C20 | C16 | C17 | C4 | -141.7 |  | C128 | C129 | C130 | C104 | -137.4 |
| C20 | C16 | C17 | C18 | 0.5 |  | C128 | C129 | C130 | C126 | -5.7 |
| C20 | C19 | C61 | C32 | -151.3 |  | C129 | C108 | C109 | C110 | 147.7 |
| C20 | C19 | C61 | C62 | -1.6 |  | C129 | C108 | C109 | C111 | 7.6 |
| C20 | C21 | C22 | C14 | -7.7 |  | C130 | C104 | C105 | C101 | 150.2 |
| C20 | C21 | C22 | C23 | -142.2 |  | C130 | C104 | C105 | C107 | 2.9 |
| C20 | C21 | C25 | C24 | 139.0 |  | C130 | C126 | C127 | C117 | -148.7 |
| C20 | C21 | C25 | C62 | -8.8 |  | C130 | C126 | C127 | C128 | -3.2 |
| C21 | C22 | C23 | C24 | -1.5 |  | C132 | C89 | C90 | C74 | -0.2 |
| C21 | C22 | C23 | C48 | 140.9 |  | C132 | C89 | C90 | C86 | 142.4 |
| C21 | C25 | C62 | C26 | 150.0 |  | C132 | C113 | C114 | C115 | -150.9 |
| C21 | C25 | C62 | C61 | 7.6 |  | C132 | C113 | C114 | C116 | -7.0 |
| C22 | C14 | C15 | C11 | -149.5 |  | C133 | C88 | C89 | C90 | 150.9 |
| C22 | C14 | C15 | C16 | -5.8 |  | C133 | C88 | C89 | C132 | 3.2 |
| C22 | C21 | C25 | C24 | -2.6 |  | C133 | C112 | C113 | C114 | -147.8 |
| C22 | C21 | C25 | C62 | -150.4 |  | C133 | C112 | C113 | C132 | 2.4 |
| C22 | C23 | C24 | C25 | -0.1 |  | C134 | C92 | C93 | C94 | -147.6 |
| C22 | C23 | C24 | C63 | 151.3 |  | C134 | C92 | C93 | C135 | -1.5 |
| C22 | C23 | C48 | C47 | -146.8 |  | C135 | C93 | C94 | C95 | -151.1 |
| C22 | C23 | C48 | C49 | 0.4 |  | C135 | C93 | C94 | C96 | -7.0 |
| C23 | C24 | C25 | C21 | 1.7 |  | C135 | C106 | C107 | C105 | 7.8 |
| C23 | C24 | C25 | C62 | 148.9 |  | C135 | C106 | C107 | C108 | 150.7 |
| C23 | C24 | C63 | C27 | -155.0 |  | C135 | C106 | C110 | C109 | -151.3 |
| C23 | C24 | C63 | C64 | -7.1 |  | C135 | C106 | C110 | C134 | -5.7 |
| C23 | C48 | C49 | C13 | -4.4 |  | C136 | C97 | C98 | C99 | -150.3 |
| C23 | C48 | C49 | C50 | -145.2 |  | C136 | C97 | C98 | C137 | 0.0 |
| C24 | C23 | C48 | C47 | -8.4 |  | C136 | C101 | C102 | C103 | 144.5 |
| C24 | C23 | C48 | C49 | 138.9 |  | C136 | C101 | C102 | C137 | 9.9 |
| C24 | C25 | C62 | C26 | 6.2 |  | C136 | C101 | C105 | C104 | -144.1 |
| C24 | C25 | C62 | C61 | -136.2 |  | C136 | C101 | C105 | C107 | 1.8 |
| C24 | C63 | C64 | C44 | -140.9 |  | C137 | C98 | C99 | C79 | -8.8 |
| C24 | C63 | C64 | C47 | 4.4 |  | C137 | C98 | C99 | C100 | -151.3 |
| C25 | C21 | C22 | C14 | 137.1 |  | C137 | C102 | C103 | C104 | 144.2 |
| C25 | C21 | C22 | C23 | 2.6 |  | C137 | C102 | C103 | C123 | 2.2 |
| C25 | C24 | C63 | C27 | -11.7 |  | C138 | C78 | C79 | C80 | 144.2 |
| C25 | C24 | C63 | C64 | 136.2 |  | C138 | C78 | C79 | C99 | 6.5 |
| C26 | C27 | C28 | C29 | -3.9 |  | C138 | C122 | C123 | C103 | -4.9 |
| C26 | C27 | C28 | C45 | 145.1 |  | C138 | C122 | C123 | C124 | -144.9 |
| C26 | C27 | C63 | C24 | 13.2 |  | C139 | C77 | C78 | C79 | 149.1 |
| C26 | C27 | C63 | C64 | -139.5 |  | C139 | C77 | C78 | C138 | -5.1 |
| C26 | C30 | C31 | C32 | -2.1 |  | C139 | C121 | C122 | C123 | -145.1 |
| C26 | C30 | C31 | C35 | 137.9 |  | C139 | C121 | C122 | C138 | -5.6 |
| C27 | C26 | C30 | C29 | -6.1 |  | C139 | C121 | C125 | C118 | 9.5 |
| C27 | C26 | C30 | C31 | -143.7 |  | C139 | C121 | C125 | C124 | 143.1 |
| C27 | C26 | C62 | C25 | -4.2 |  | C140 | C71 | C72 | C73 | 144.4 |
| C27 | C26 | C62 | C61 | 137.0 |  | C140 | C71 | C72 | C76 | 2.9 |
| C27 | C28 | C29 | C30 | 0.1 |  | C140 | C71 | C75 | C74 | -142.3 |
| C27 | C28 | C29 | C40 | 143.5 |  | C140 | C71 | C75 | C131 | 1.2 |
| C27 | C28 | C45 | C41 | -136.9 |  | C140 | C119 | C120 | C116 | 147.0 |
| C27 | C28 | C45 | C44 | -4.8 |  | C140 | C119 | C120 | C131 | -3.0 |
| C27 | C63 | C64 | C44 | 4.5 |  | C141 | C151 | C162 | C153 | 2.5(14) |
| C27 | C63 | C64 | C47 | 149.7 |  | C141 | C151 | C162 | C181 | -160.0(8) |
| C28 | C27 | C63 | C24 | 155.4 |  | C141 | C151 | C184 | C167 | 171.3(8) |
| C28 | C27 | C63 | C64 | 2.7 |  | C141 | C151 | C184 | C189 | -2.4(12) |
| C28 | C29 | C30 | C26 | 3.6 |  | C141 | C161 | C172 | S1 | -167.0(7) |
| C28 | C29 | C30 | C31 | 138.5 |  | C141 | C161 | C172 | C189 | 4.0(12) |
| C28 | C29 | C40 | C36 | -138.6 |  | C142 | C143 | C145 | C146 | -161.8(8) |
| C28 | C29 | C40 | C39 | 5.6 |  | C142 | C143 | C145 | C150 | 4.3(12) |
| C29 | C28 | C45 | C41 | 8.1 |  | C142 | C143 | C149 | C157 | 3.2(11) |
| C29 | C28 | C45 | C44 | 140.2 |  | C142 | C143 | C149 | C158 | 160.0(8) |
| C29 | C30 | C31 | C32 | -134.3 |  | C142 | C147 | C155 | C157 | 2.2(13) |
| C29 | C30 | C31 | C35 | 5.7 |  | C142 | C147 | C155 | C178 | -161.9(8) |
| C30 | C26 | C27 | C28 | 6.3 |  | C142 | C147 | C163 | S3 | 165.7(7) |
| C30 | C26 | C27 | C63 | 149.5 |  | C142 | C147 | C163 | C175 | -1.9(13) |
| C30 | C26 | C62 | C25 | -155.5 |  | C142 | C168 | C169 | C175 | 4.6(12) |
| C30 | C26 | C62 | C61 | -14.3 |  | C142 | C168 | C169 | C185 | -168.4(7) |
| C30 | C29 | C40 | C36 | -0.4 |  | C143 | C142 | C147 | C155 | 1.1(13) |
| C30 | C29 | C40 | C39 | 143.8 |  | C143 | C142 | C147 | C163 | -157.7(8) |
| C30 | C31 | C32 | C33 | 142.9 |  | C143 | C142 | C168 | C144 | 3.5(12) |
| C30 | C31 | C32 | C61 | -2.7 |  | C143 | C142 | C168 | C169 | 157.0(8) |
| C30 | C31 | C35 | C34 | -143.0 |  | C143 | C145 | C146 | C153 | 165.1(8) |
| C30 | C31 | C35 | C36 | -1.2 |  | C143 | C145 | C146 | C165 | 0.7(13) |
| C31 | C32 | C33 | C34 | -3.6 |  | C143 | C145 | C150 | C144 | -3.9(13) |
| C31 | C32 | C33 | C70 | -148.5 |  | C143 | C145 | C150 | C170 | -158.4(8) |
| C31 | C32 | C61 | C19 | 151.5 |  | C143 | C149 | C157 | C155 | 0.0(13) |
| C31 | C32 | C61 | C62 | -0.6 |  | C143 | C149 | C157 | C187 | 163.2(8) |
| C31 | C35 | C36 | C37 | -137.7 |  | C143 | C149 | C158 | C160 | -160.8(8) |
| C31 | C35 | C36 | C40 | -3.9 |  | C143 | C149 | C158 | C165 | 4.0(13) |
| C32 | C31 | C35 | C34 | 2.9 |  | C144 | C150 | C170 | C171 | -163.2(8) |
| C32 | C31 | C35 | C36 | 144.7 |  | C144 | C150 | C170 | C179 | -1.8(12) |
| C32 | C33 | C34 | C35 | 5.4 |  | C144 | C152 | C173 | C179 | 2.3(11) |
| C32 | C33 | C34 | C58 | -150.9 |  | C144 | C152 | C185 | C169 | -17.2(8) |
| C32 | C33 | C70 | C18 | -6.2 |  | C144 | C152 | C185 | C174 | -138.1(7) |
| C32 | C33 | C70 | C69 | 138.2 |  | C144 | C152 | C185 | C188 | 96.4(7) |
| C32 | C61 | C62 | C25 | 148.8 |  | C144 | C168 | C169 | C175 | 160.5(7) |
| C32 | C61 | C62 | C26 | 8.3 |  | C144 | C168 | C169 | C185 | -12.5(9) |
| C33 | C32 | C61 | C19 | 9.1 |  | C145 | C143 | C149 | C157 | -158.1(8) |
| C33 | C32 | C61 | C62 | -142.9 |  | C145 | C143 | C149 | C158 | -1.3(13) |
| C33 | C34 | C35 | C31 | -5.2 |  | C145 | C146 | C153 | C162 | -161.2(8) |
| C33 | C34 | C35 | C36 | -148.4 |  | C145 | C146 | C153 | C171 | -6.8(13) |
| C33 | C34 | C58 | C57 | 14.2 |  | C145 | C146 | C165 | C141 | 161.4(8) |
| C33 | C34 | C58 | C59 | 145.6 |  | C145 | C146 | C165 | C158 | 2.0(13) |
| C34 | C33 | C70 | C18 | -146.1 |  | C145 | C150 | C170 | C171 | -7.3(13) |
| C34 | C33 | C70 | C69 | -1.6 |  | C145 | C150 | C170 | C179 | 154.1(8) |
| C34 | C35 | C36 | C37 | -0.4 |  | C146 | C145 | C150 | C144 | 162.4(8) |
| C34 | C35 | C36 | C40 | 133.3 |  | C146 | C145 | C150 | C170 | 8.0(12) |
| C34 | C58 | C59 | C37 | 1.9 |  | C146 | C153 | C162 | C151 | -2.8(14) |
| C34 | C58 | C59 | C60 | -137.0 |  | C146 | C153 | C162 | C181 | 157.3(9) |
| C35 | C31 | C32 | C33 | 0.4 |  | C146 | C153 | C171 | C170 | 7.6(13) |
| C35 | C31 | C32 | C61 | -145.2 |  | C146 | C153 | C171 | C183 | -154.5(9) |
| C35 | C34 | C58 | C57 | -137.8 |  | C147 | C142 | C143 | C145 | 157.7(8) |
| C35 | C34 | C58 | C59 | -6.4 |  | C147 | C142 | C143 | C149 | -3.8(12) |
| C35 | C36 | C37 | C38 | 144.2 |  | C147 | C142 | C168 | C144 | -159.5(8) |
| C35 | C36 | C37 | C59 | -3.6 |  | C147 | C142 | C168 | C169 | -5.9(12) |
| C35 | C36 | C40 | C29 | 4.9 |  | C147 | C155 | C157 | C149 | -2.8(13) |
| C35 | C36 | C40 | C39 | -144.7 |  | C147 | C155 | C157 | C187 | -167.1(8) |
| C36 | C37 | C38 | C39 | -3.4 |  | C147 | C155 | C178 | S3 | -3.5(10) |
| C36 | C37 | C38 | C54 | -142.3 |  | C147 | C155 | C178 | C177 | 164.7(8) |
| C36 | C37 | C59 | C58 | 3.0 |  | C147 | C163 | C175 | C169 | 0.3(12) |
| C36 | C37 | C59 | C60 | 139.8 |  | C148 | C159 | C160 | C154 | -6.5(13) |
| C37 | C36 | C40 | C29 | 143.3 |  | C148 | C159 | C160 | C158 | 155.3(8) |
| C37 | C36 | C40 | C39 | -6.2 |  | C148 | C159 | C187 | C157 | -156.3(8) |
| C37 | C38 | C39 | C40 | -0.7 |  | C148 | C159 | C187 | C190 | 1.1(11) |
| C37 | C38 | C39 | C41 | -137.2 |  | C148 | C180 | C190 | C177 | 151.1(10) |
| C37 | C38 | C54 | C53 | -5.1 |  | C148 | C180 | C190 | C187 | -19.7(9) |
| C37 | C38 | C54 | C55 | 136.2 |  | C149 | C143 | C145 | C146 | -1.0(13) |
| C37 | C59 | C60 | C53 | 3.7 |  | C149 | C143 | C145 | C150 | 165.0(8) |
| C37 | C59 | C60 | C56 | -146.4 |  | C149 | C157 | C187 | C159 | -4.7(13) |
| C38 | C37 | C59 | C58 | -140.0 |  | C149 | C157 | C187 | C190 | -159.5(8) |
| C38 | C37 | C59 | C60 | -3.1 |  | C149 | C158 | C160 | C154 | 161.2(8) |
| C38 | C39 | C40 | C29 | -144.3 |  | C149 | C158 | C160 | C159 | -0.1(13) |
| C38 | C39 | C40 | C36 | 4.2 |  | C149 | C158 | C165 | C141 | -164.0(8) |
| C38 | C39 | C41 | C42 | -11.1 |  | C149 | C158 | C165 | C146 | -4.4(13) |
| C38 | C39 | C41 | C45 | 140.0 |  | C150 | C144 | C152 | C173 | -1.7(12) |
| C38 | C54 | C55 | C42 | 1.1 |  | C150 | C144 | C152 | C185 | 171.1(7) |
| C38 | C54 | C55 | C51 | -147.3 |  | C150 | C144 | C168 | C142 | -3.2(13) |
| C39 | C38 | C54 | C53 | -140.9 |  | C150 | C144 | C168 | C169 | -159.8(7) |
| C39 | C38 | C54 | C55 | 0.4 |  | C150 | C145 | C146 | C153 | -1.0(12) |
| C39 | C41 | C42 | C43 | 148.4 |  | C150 | C145 | C146 | C165 | -165.3(8) |
| C39 | C41 | C42 | C55 | 12.7 |  | C150 | C170 | C171 | C153 | -0.4(13) |
| C39 | C41 | C45 | C28 | -7.6 |  | C150 | C170 | C171 | C183 | 163.7(8) |
| C39 | C41 | C45 | C44 | -146.3 |  | C150 | C170 | C179 | S2 | -167.0(7) |
| C40 | C29 | C30 | C26 | -139.8 |  | C150 | C170 | C179 | C173 | 2.7(13) |
| C40 | C29 | C30 | C31 | -5.0 |  | C151 | C141 | C161 | C154 | -165.1(8) |
| C40 | C36 | C37 | C38 | 5.8 |  | C151 | C141 | C161 | C172 | -2.6(12) |
| C40 | C36 | C37 | C59 | -142.0 |  | C151 | C141 | C165 | C146 | 2.5(12) |
| C40 | C39 | C41 | C42 | -145.7 |  | C151 | C141 | C165 | C158 | 162.6(8) |
| C40 | C39 | C41 | C45 | 5.4 |  | C151 | C162 | C181 | C164 | 158.5(8) |
| C41 | C39 | C40 | C29 | -4.4 |  | C151 | C162 | C181 | C167 | -11.0(10) |
| C41 | C39 | C40 | C36 | 144.1 |  | C151 | C184 | C189 | C172 | 3.6(12) |
| C41 | C42 | C43 | C44 | 3.3 |  | C152 | C144 | C150 | C145 | -154.6(8) |
| C41 | C42 | C43 | C65 | -141.8 |  | C152 | C144 | C150 | C170 | 1.3(12) |
| C41 | C42 | C55 | C51 | 131.0 |  | C152 | C144 | C168 | C142 | 157.7(7) |
| C41 | C42 | C55 | C54 | -7.4 |  | C152 | C144 | C168 | C169 | 1.0(9) |
| C42 | C41 | C45 | C28 | 146.4 |  | C152 | C173 | C179 | S2 | 164.4(7) |
| C42 | C41 | C45 | C44 | 7.7 |  | C152 | C173 | C179 | C170 | -2.9(12) |
| C42 | C43 | C44 | C45 | 1.4 |  | C153 | C146 | C165 | C141 | -2.8(13) |
| C42 | C43 | C44 | C64 | -141.0 |  | C153 | C146 | C165 | C158 | -162.2(8) |
| C42 | C43 | C65 | C46 | 152.9 |  | C153 | C162 | C181 | C164 | -3.1(14) |
| C42 | C43 | C65 | C66 | 12.3 |  | C153 | C162 | C181 | C167 | -172.7(8) |
| C43 | C42 | C55 | C51 | -2.2 |  | C153 | C171 | C183 | S2 | 166.8(7) |
| C43 | C42 | C55 | C54 | -140.6 |  | C153 | C171 | C183 | C164 | -1.4(13) |
| C43 | C44 | C45 | C28 | -140.5 |  | C154 | C156 | C182 | C148 | -0.6(13) |
| C43 | C44 | C45 | C41 | -5.6 |  | C154 | C161 | C172 | S1 | -3.6(9) |
| C43 | C44 | C64 | C47 | -18.4 |  | C154 | C161 | C172 | C189 | 167.5(7) |
| C43 | C44 | C64 | C63 | 129.7 |  | C155 | C147 | C163 | S3 | 5.5(9) |
| C43 | C65 | C66 | C9 | 141.2 |  | C155 | C147 | C163 | C175 | -162.1(8) |
| C43 | C65 | C66 | C51 | -13.5 |  | C155 | C157 | C187 | C159 | 160.1(8) |
| C44 | C43 | C65 | C46 | 11.2 |  | C155 | C157 | C187 | C190 | 5.3(13) |
| C44 | C43 | C65 | C66 | -129.5 |  | C156 | S1 | C172 | C161 | 5.3(6) |
| C45 | C28 | C29 | C30 | -151.2 |  | C156 | S1 | C172 | C189 | -164.2(8) |
| C45 | C28 | C29 | C40 | -7.8 |  | C156 | C154 | C160 | C158 | -157.4(8) |
| C45 | C41 | C42 | C43 | -6.9 |  | C156 | C154 | C160 | C159 | 5.0(12) |
| C45 | C41 | C42 | C55 | -142.7 |  | C156 | C154 | C161 | C141 | 163.2(8) |
| C45 | C44 | C64 | C47 | -159.0 |  | C156 | C154 | C161 | C172 | -0.9(11) |
| C45 | C44 | C64 | C63 | -10.9 |  | C157 | C149 | C158 | C160 | -4.0(12) |
| C46 | C47 | C48 | C23 | 145.0 |  | C157 | C149 | C158 | C165 | 160.7(8) |
| C46 | C47 | C48 | C49 | -6.1 |  | C157 | C155 | C178 | S3 | -166.4(7) |
| C46 | C47 | C64 | C44 | 17.2 |  | C157 | C155 | C178 | C177 | 1.8(14) |
| C46 | C47 | C64 | C63 | -134.8 |  | C157 | C187 | C190 | C177 | -3.4(14) |
| C46 | C65 | C66 | C9 | -0.7 |  | C157 | C187 | C190 | C180 | 169.0(8) |
| C46 | C65 | C66 | C51 | -155.3 |  | C158 | C149 | C157 | C155 | -156.8(8) |
| C47 | C46 | C50 | C8 | -143.5 |  | C158 | C149 | C157 | C187 | 6.5(13) |
| C47 | C46 | C50 | C49 | -1.0 |  | C159 | C148 | C180 | C176 | 140.2(7) |
| C47 | C46 | C65 | C43 | -10.3 |  | C159 | C148 | C180 | C186 | -95.0(8) |
| C47 | C46 | C65 | C66 | 138.4 |  | C159 | C148 | C180 | C190 | 20.6(9) |
| C47 | C48 | C49 | C13 | 146.6 |  | C159 | C148 | C182 | C156 | -0.6(13) |
| C47 | C48 | C49 | C50 | 5.7 |  | C159 | C187 | C190 | C177 | -159.7(8) |
| C48 | C23 | C24 | C25 | -142.8 |  | C159 | C187 | C190 | C180 | 12.7(10) |
| C48 | C23 | C24 | C63 | 8.6 |  | C160 | C154 | C156 | S1 | 165.3(6) |
| C48 | C47 | C64 | C44 | 149.0 |  | C160 | C154 | C156 | C182 | -1.7(13) |
| C48 | C47 | C64 | C63 | -3.0 |  | C160 | C154 | C161 | C141 | 3.0(13) |
| C48 | C49 | C50 | C8 | 139.5 |  | C160 | C154 | C161 | C172 | -161.1(8) |
| C48 | C49 | C50 | C46 | -2.9 |  | C160 | C158 | C165 | C141 | 0.5(12) |
| C49 | C13 | C14 | C15 | -146.9 |  | C160 | C158 | C165 | C146 | 160.1(8) |
| C49 | C13 | C14 | C22 | -1.0 |  | C160 | C159 | C187 | C157 | 0.5(13) |
| C50 | C8 | C9 | C10 | 144.1 |  | C160 | C159 | C187 | C190 | 157.9(8) |
| C50 | C8 | C9 | C66 | -3.8 |  | C161 | C141 | C151 | C162 | 157.1(8) |
| C50 | C46 | C47 | C48 | 4.3 |  | C161 | C141 | C151 | C184 | 1.9(12) |
| C50 | C46 | C47 | C64 | 140.0 |  | C161 | C141 | C165 | C146 | -155.6(8) |
| C50 | C46 | C65 | C43 | -150.8 |  | C161 | C141 | C165 | C158 | 4.5(13) |
| C50 | C46 | C65 | C66 | -2.1 |  | C161 | C154 | C156 | S1 | 4.9(9) |
| C51 | C52 | C53 | C54 | -0.6 |  | C161 | C154 | C156 | C182 | -162.0(8) |
| C51 | C52 | C53 | C60 | 144.6 |  | C161 | C154 | C160 | C158 | 1.9(13) |
| C51 | C52 | C67 | C10 | 8.5 |  | C161 | C154 | C160 | C159 | 164.3(8) |
| C51 | C52 | C67 | C68 | -137.4 |  | C161 | C172 | C189 | C184 | -4.4(12) |
| C52 | C51 | C55 | C42 | -137.1 |  | C162 | C151 | C184 | C167 | 13.6(9) |
| C52 | C51 | C55 | C54 | 3.6 |  | C162 | C151 | C184 | C189 | -160.1(7) |
| C52 | C51 | C66 | C9 | -22.6 |  | C162 | C153 | C171 | C170 | 163.3(8) |
| C52 | C51 | C66 | C65 | 138.2 |  | C162 | C153 | C171 | C183 | 1.2(13) |
| C52 | C53 | C54 | C38 | 150.7 |  | C163 | S3 | C178 | C155 | 5.6(7) |
| C52 | C53 | C54 | C55 | 2.8 |  | C163 | S3 | C178 | C177 | -161.0(8) |
| C52 | C53 | C60 | C56 | 0.2 |  | C163 | C147 | C155 | C157 | 162.8(8) |
| C52 | C53 | C60 | C59 | -145.4 |  | C163 | C147 | C155 | C178 | -1.4(11) |
| C52 | C67 | C68 | C2 | 146.0 |  | C165 | C141 | C151 | C162 | -2.4(13) |
| C52 | C67 | C68 | C56 | -4.2 |  | C165 | C141 | C151 | C184 | -157.6(8) |
| C53 | C52 | C67 | C10 | 150.2 |  | C165 | C141 | C161 | C154 | -6.3(13) |
| C53 | C52 | C67 | C68 | 4.3 |  | C165 | C141 | C161 | C172 | 156.2(8) |
| C53 | C54 | C55 | C42 | 144.5 |  | C165 | C146 | C153 | C162 | 2.9(13) |
| C53 | C54 | C55 | C51 | -3.9 |  | C165 | C146 | C153 | C171 | 157.3(8) |
| C54 | C38 | C39 | C40 | 141.0 |  | C165 | C158 | C160 | C154 | -3.6(12) |
| C54 | C38 | C39 | C41 | 4.5 |  | C165 | C158 | C160 | C159 | -164.9(8) |
| C54 | C53 | C60 | C56 | 140.4 |  | C166 | C167 | C181 | C162 | 138.4(8) |
| C54 | C53 | C60 | C59 | -5.2 |  | C166 | C167 | C181 | C164 | -29.8(12) |
| C55 | C42 | C43 | C44 | 142.0 |  | C166 | C167 | C184 | C151 | -139.3(7) |
| C55 | C42 | C43 | C65 | -3.1 |  | C166 | C167 | C184 | C189 | 32.8(12) |
| C55 | C51 | C52 | C53 | -1.9 |  | C167 | C184 | C189 | C172 | -167.9(9) |
| C55 | C51 | C52 | C67 | 141.9 |  | C168 | C142 | C143 | C145 | -4.1(12) |
| C55 | C51 | C66 | C9 | -154.1 |  | C168 | C142 | C143 | C149 | -165.7(8) |
| C55 | C51 | C66 | C65 | 6.7 |  | C168 | C142 | C147 | C155 | 163.3(8) |
| C56 | C57 | C58 | C34 | 135.2 |  | C168 | C142 | C147 | C163 | 4.5(12) |
| C56 | C57 | C58 | C59 | 1.1 |  | C168 | C144 | C150 | C145 | 3.3(13) |
| C56 | C57 | C69 | C3 | 7.4 |  | C168 | C144 | C150 | C170 | 159.2(8) |
| C56 | C57 | C69 | C70 | -139.4 |  | C168 | C144 | C152 | C173 | -161.6(7) |
| C57 | C56 | C60 | C53 | -145.5 |  | C168 | C144 | C152 | C185 | 11.2(9) |
| C57 | C56 | C60 | C59 | 4.8 |  | C168 | C169 | C175 | C163 | -1.6(11) |
| C57 | C56 | C68 | C2 | -7.0 |  | C168 | C169 | C185 | C152 | 17.8(8) |
| C57 | C56 | C68 | C67 | 143.2 |  | C168 | C169 | C185 | C174 | 140.7(7) |
| C57 | C58 | C59 | C37 | 140.8 |  | C168 | C169 | C185 | C188 | -97.1(7) |
| C57 | C58 | C59 | C60 | 1.9 |  | C170 | C171 | C183 | S2 | 3.7(9) |
| C57 | C69 | C70 | C18 | 145.8 |  | C170 | C171 | C183 | C164 | -164.5(8) |
| C57 | C69 | C70 | C33 | 1.4 |  | C171 | C153 | C162 | C151 | -159.0(8) |
| C58 | C34 | C35 | C31 | 149.3 |  | C171 | C153 | C162 | C181 | 1.1(13) |
| C58 | C34 | C35 | C36 | 6.0 |  | C171 | C170 | C179 | S2 | -4.8(9) |
| C58 | C57 | C69 | C3 | 152.8 |  | C171 | C170 | C179 | C173 | 164.9(8) |
| C58 | C57 | C69 | C70 | 5.9 |  | C172 | S1 | C156 | C154 | -5.8(6) |
| C58 | C59 | C60 | C53 | 146.0 |  | C172 | S1 | C156 | C182 | 159.9(8) |
| C58 | C59 | C60 | C56 | -4.1 |  | C173 | C152 | C185 | C169 | 154.2(8) |
| C59 | C37 | C38 | C39 | 143.1 |  | C173 | C152 | C185 | C174 | 33.3(11) |
| C59 | C37 | C38 | C54 | 4.2 |  | C173 | C152 | C185 | C188 | -92.2(11) |
| C60 | C53 | C54 | C38 | 5.6 |  | C175 | C169 | C185 | C152 | -153.7(9) |
| C60 | C53 | C54 | C55 | -142.3 |  | C175 | C169 | C185 | C174 | -30.8(11) |
| C60 | C56 | C57 | C58 | -3.7 |  | C175 | C169 | C185 | C188 | 91.3(10) |
| C60 | C56 | C57 | C69 | 145.6 |  | C176 | C180 | C190 | C177 | 30.4(13) |
| C60 | C56 | C68 | C2 | -148.2 |  | C176 | C180 | C190 | C187 | -140.4(7) |
| C60 | C56 | C68 | C67 | 2.0 |  | C178 | S3 | C163 | C147 | -6.3(7) |
| C61 | C19 | C20 | C16 | 145.0 |  | C178 | S3 | C163 | C175 | 159.9(8) |
| C61 | C19 | C20 | C21 | 1.0 |  | C178 | C155 | C157 | C149 | 159.8(9) |
| C61 | C32 | C33 | C34 | 143.8 |  | C178 | C155 | C157 | C187 | -4.5(13) |
| C61 | C32 | C33 | C70 | -1.1 |  | C178 | C177 | C190 | C180 | -169.6(9) |
| C62 | C26 | C27 | C28 | -149.2 |  | C178 | C177 | C190 | C187 | 0.1(13) |
| C62 | C26 | C27 | C63 | -6.1 |  | C179 | S2 | C183 | C164 | 161.0(8) |
| C62 | C26 | C30 | C29 | 148.5 |  | C179 | S2 | C183 | C171 | -5.5(7) |
| C62 | C26 | C30 | C31 | 10.8 |  | C179 | C170 | C171 | C153 | -163.4(8) |
| C63 | C24 | C25 | C21 | -144.3 |  | C179 | C170 | C171 | C183 | 0.8(11) |
| C63 | C24 | C25 | C62 | 3.0 |  | C180 | C148 | C159 | C160 | -169.9(8) |
| C63 | C27 | C28 | C29 | -151.4 |  | C180 | C148 | C159 | C187 | -14.4(10) |
| C63 | C27 | C28 | C45 | -2.4 |  | C180 | C148 | C182 | C156 | 171.8(8) |
| C64 | C44 | C45 | C28 | 10.8 |  | C181 | C164 | C183 | S2 | -166.0(7) |
| C64 | C44 | C45 | C41 | 145.8 |  | C181 | C164 | C183 | C171 | -0.6(12) |
| C64 | C47 | C48 | C23 | 5.4 |  | C181 | C167 | C184 | C151 | -18.4(9) |
| C64 | C47 | C48 | C49 | -145.7 |  | C181 | C167 | C184 | C189 | 153.7(9) |
| C65 | C43 | C44 | C45 | 146.7 |  | C182 | C148 | C159 | C160 | 4.4(14) |
| C65 | C43 | C44 | C64 | 4.3 |  | C182 | C148 | C159 | C187 | 160.0(8) |
| C65 | C46 | C47 | C48 | -140.6 |  | C182 | C148 | C180 | C176 | -33.0(12) |
| C65 | C46 | C47 | C64 | -4.9 |  | C182 | C148 | C180 | C186 | 91.8(11) |
| C65 | C46 | C50 | C8 | 1.6 |  | C182 | C148 | C180 | C190 | -152.6(9) |
| C65 | C46 | C50 | C49 | 144.1 |  | C183 | S2 | C179 | C170 | 5.9(7) |
| C66 | C9 | C10 | C6 | 142.7 |  | C183 | S2 | C179 | C173 | -162.6(8) |
| C66 | C9 | C10 | C67 | 1.3 |  | C183 | C164 | C181 | C162 | 2.7(13) |
| C66 | C51 | C52 | C53 | -134.4 |  | C183 | C164 | C181 | C167 | 169.6(8) |
| C66 | C51 | C52 | C67 | 9.4 |  | C184 | C151 | C162 | C153 | 161.1(8) |
| C66 | C51 | C55 | C42 | 0.2 |  | C184 | C151 | C162 | C181 | -1.4(10) |
| C66 | C51 | C55 | C54 | 140.9 |  | C184 | C167 | C181 | C162 | 17.7(9) |
| C67 | C52 | C53 | C54 | -147.5 |  | C184 | C167 | C181 | C164 | -150.5(9) |
| C67 | C52 | C53 | C60 | -2.3 |  | C185 | C152 | C173 | C179 | -168.3(8) |
| C68 | C2 | C3 | C4 | 146.7 |  | C185 | C169 | C175 | C163 | 169.2(8) |
| C68 | C2 | C3 | C69 | 2.1 |  | C186 | C180 | C190 | C177 | -90.7(12) |
| C68 | C56 | C57 | C58 | -148.9 |  | C186 | C180 | C190 | C187 | 98.5(8) |
| C68 | C56 | C57 | C69 | 0.3 |  | C187 | C159 | C160 | C154 | -159.9(8) |
| C68 | C56 | C60 | C53 | 0.0 |  | C187 | C159 | C160 | C158 | 1.9(14) |
| C68 | C56 | C60 | C59 | 150.3 |  | C190 | C177 | C178 | S3 | 166.0(7) |
| C69 | C3 | C4 | C5 | 150.8 |  | C190 | C177 | C178 | C155 | 0.6(13) |
| C69 | C3 | C4 | C17 | -0.4 |  | C191 | C167 | C181 | C162 | -95.6(9) |
| C69 | C57 | C58 | C34 | -14.6 |  | C191 | C167 | C181 | C164 | 96.2(11) |
| C69 | C57 | C58 | C59 | -148.7 |  | C191 | C167 | C184 | C151 | 95.5(8) |
| C70 | C18 | C19 | C20 | 148.8 |  | C191 | C167 | C184 | C189 | -92.4(11) |
| C70 | C18 | C19 | C61 | 2.4 |  | C192 | C198 | C200 | C205 | 1.1(12) |
| C70 | C33 | C34 | C35 | 150.1 |  | C192 | C198 | C200 | C220 | 158.0(8) |
| C70 | C33 | C34 | C58 | -6.2 |  | C192 | C201 | C202 | C197 | 4.4(13) |
| C71 | C72 | C73 | C74 | 0.4 |  | C192 | C201 | C202 | C211 | 164.1(8) |
| C71 | C72 | C73 | C82 | -148.1 |  | C192 | C201 | C226 | S4 | 3.7(9) |
| C71 | C72 | C76 | C77 | -0.9 |  | C192 | C201 | C226 | C235 | -163.5(8) |
| C71 | C72 | C76 | C80 | 140.6 |  | C192 | C208 | C228 | C205 | -0.7(11) |
| C71 | C75 | C131 | C120 | 7.5 |  | C193 | C210 | C212 | C195 | 0.0(13) |
| C71 | C75 | C131 | C132 | -143.4 |  | C193 | C210 | C212 | C217 | -160.7(8) |
| C72 | C71 | C75 | C74 | 1.2 |  | C193 | C210 | C221 | C222 | 168.8(8) |
| C72 | C71 | C75 | C131 | 144.7 |  | C193 | C210 | C221 | C225 | -2.5(13) |
| C72 | C71 | C140 | C119 | -149.7 |  | C194 | C197 | C202 | C201 | -5.6(13) |
| C72 | C71 | C140 | C139 | -0.8 |  | C194 | C197 | C202 | C211 | -164.7(8) |
| C72 | C73 | C74 | C75 | 0.3 |  | C194 | C197 | C206 | C199 | 0.8(13) |
| C72 | C73 | C74 | C90 | -148.0 |  | C194 | C197 | C206 | C204 | 161.5(8) |
| C72 | C73 | C82 | C81 | 7.7 |  | C194 | C198 | C200 | C205 | -154.8(8) |
| C72 | C73 | C82 | C83 | 143.2 |  | C194 | C198 | C200 | C220 | 2.1(13) |
| C72 | C76 | C77 | C78 | 141.9 |  | C194 | C203 | C209 | C193 | -162.2(8) |
| C72 | C76 | C77 | C139 | -3.6 |  | C194 | C203 | C209 | C199 | -0.1(13) |
| C72 | C76 | C80 | C79 | -150.2 |  | C194 | C203 | C213 | C207 | 157.2(8) |
| C72 | C76 | C80 | C81 | -0.3 |  | C194 | C203 | C213 | C220 | -2.0(13) |
| C73 | C72 | C76 | C77 | -136.8 |  | C195 | C199 | C206 | C197 | 161.3(8) |
| C73 | C72 | C76 | C80 | 4.7 |  | C195 | C199 | C206 | C204 | 0.6(12) |
| C73 | C74 | C75 | C71 | -0.9 |  | C195 | C199 | C209 | C193 | 0.3(12) |
| C73 | C74 | C75 | C131 | -141.5 |  | C195 | C199 | C209 | C203 | -161.8(8) |
| C73 | C74 | C90 | C86 | 8.8 |  | C195 | C212 | C217 | C222 | -167.6(8) |
| C73 | C74 | C90 | C89 | 144.1 |  | C195 | C212 | C217 | C242 | -1.3(12) |
| C73 | C82 | C83 | C84 | -141.8 |  | C195 | C215 | C237 | S6 | 166.0(7) |
| C73 | C82 | C83 | C86 | -5.8 |  | C195 | C215 | C237 | C242 | 0.1(13) |
| C74 | C73 | C82 | C81 | -133.8 |  | C196 | S5 | C219 | C207 | -7.3(7) |
| C74 | C73 | C82 | C83 | 1.7 |  | C196 | S5 | C219 | C224 | 163.6(8) |
| C74 | C75 | C131 | C120 | 146.6 |  | C197 | C194 | C198 | C192 | 6.4(12) |
| C74 | C75 | C131 | C132 | -4.3 |  | C197 | C194 | C198 | C200 | 160.8(8) |
| C75 | C71 | C72 | C73 | -1.0 |  | C197 | C194 | C203 | C209 | 0.5(13) |
| C75 | C71 | C72 | C76 | -142.4 |  | C197 | C194 | C203 | C213 | -160.9(8) |
| C75 | C71 | C140 | C119 | -9.8 |  | C197 | C202 | C211 | C229 | 158.1(9) |
| C75 | C71 | C140 | C139 | 139.1 |  | C197 | C202 | C211 | C234 | 1.7(14) |
| C75 | C74 | C90 | C86 | -126.8 |  | C198 | C192 | C201 | C202 | 2.4(12) |
| C75 | C74 | C90 | C89 | 8.5 |  | C198 | C192 | C201 | C226 | 164.2(7) |
| C75 | C131 | C132 | C89 | 12.0 |  | C198 | C192 | C208 | S4 | -167.6(7) |
| C75 | C131 | C132 | C113 | 153.8 |  | C198 | C192 | C208 | C228 | 2.3(13) |
| C76 | C72 | C73 | C74 | 140.0 |  | C198 | C194 | C197 | C202 | 0.2(12) |
| C76 | C72 | C73 | C82 | -8.6 |  | C198 | C194 | C197 | C206 | -163.7(8) |
| C76 | C77 | C78 | C79 | 5.7 |  | C198 | C194 | C203 | C209 | 163.7(8) |
| C76 | C77 | C78 | C138 | -148.4 |  | C198 | C194 | C203 | C213 | 2.3(12) |
| C76 | C77 | C139 | C121 | 153.5 |  | C198 | C200 | C205 | C223 | 172.2(8) |
| C76 | C77 | C139 | C140 | 5.8 |  | C198 | C200 | C205 | C228 | 0.3(13) |
| C76 | C80 | C81 | C82 | -0.3 |  | C198 | C200 | C220 | C213 | -1.7(13) |
| C76 | C80 | C81 | C85 | -143.4 |  | C198 | C200 | C220 | C230 | -162.1(8) |
| C77 | C76 | C80 | C79 | -1.5 |  | C199 | C195 | C212 | C210 | 0.0(13) |
| C77 | C76 | C80 | C81 | 148.3 |  | C199 | C195 | C212 | C217 | 157.8(8) |
| C77 | C78 | C79 | C80 | -6.8 |  | C199 | C195 | C215 | C214 | 2.5(13) |
| C77 | C78 | C79 | C99 | -144.6 |  | C199 | C195 | C215 | C237 | -157.3(8) |
| C77 | C78 | C138 | C122 | -8.5 |  | C200 | C205 | C223 | C230 | -18.4(8) |
| C77 | C78 | C138 | C137 | 142.7 |  | C200 | C205 | C223 | C231 | -139.7(7) |
| C77 | C139 | C140 | C71 | -3.8 |  | C200 | C205 | C223 | C233 | 96.9(7) |
| C77 | C139 | C140 | C119 | 143.9 |  | C200 | C205 | C228 | C208 | -0.5(11) |
| C78 | C77 | C139 | C121 | 13.7 |  | C200 | C220 | C230 | C223 | -10.5(10) |
| C78 | C77 | C139 | C140 | -134.0 |  | C200 | C220 | C230 | C224 | 158.6(8) |
| C78 | C79 | C80 | C76 | 5.3 |  | C201 | C192 | C198 | C194 | -7.7(12) |
| C78 | C79 | C80 | C81 | -142.7 |  | C201 | C192 | C198 | C200 | -164.0(8) |
| C78 | C79 | C99 | C98 | 0.8 |  | C201 | C192 | C208 | S4 | -5.1(9) |
| C78 | C79 | C99 | C100 | 142.2 |  | C201 | C192 | C208 | C228 | 164.8(7) |
| C79 | C78 | C138 | C122 | -156.7 |  | C201 | C202 | C211 | C229 | -2.1(13) |
| C79 | C78 | C138 | C137 | -5.5 |  | C201 | C202 | C211 | C234 | -158.5(8) |
| C79 | C80 | C81 | C82 | 143.9 |  | C201 | C226 | C235 | C229 | -1.3(13) |
| C79 | C80 | C81 | C85 | 0.9 |  | C202 | C197 | C206 | C199 | -162.6(8) |
| C79 | C99 | C100 | C85 | -1.0 |  | C202 | C197 | C206 | C204 | -1.8(12) |
| C79 | C99 | C100 | C96 | -149.5 |  | C202 | C201 | C226 | S4 | 165.2(6) |
| C80 | C76 | C77 | C78 | -2.5 |  | C202 | C201 | C226 | C235 | -2.0(12) |
| C80 | C76 | C77 | C139 | -148.1 |  | C202 | C211 | C229 | C232 | -171.6(9) |
| C80 | C79 | C99 | C98 | -131.2 |  | C202 | C211 | C229 | C235 | -1.0(15) |
| C80 | C79 | C99 | C100 | 10.3 |  | C202 | C211 | C234 | C204 | 0.2(14) |
| C80 | C81 | C82 | C73 | -3.7 |  | C202 | C211 | C234 | C240 | 160.1(9) |
| C80 | C81 | C82 | C83 | -141.2 |  | C203 | C194 | C197 | C202 | 163.1(8) |
| C80 | C81 | C85 | C84 | 143.3 |  | C203 | C194 | C197 | C206 | -0.8(13) |
| C80 | C81 | C85 | C100 | 8.0 |  | C203 | C194 | C198 | C192 | -156.8(8) |
| C81 | C82 | C83 | C84 | -3.0 |  | C203 | C194 | C198 | C200 | -2.4(12) |
| C81 | C82 | C83 | C86 | 133.0 |  | C203 | C213 | C220 | C200 | 1.7(13) |
| C81 | C85 | C100 | C96 | 136.1 |  | C203 | C213 | C220 | C230 | 159.3(8) |
| C81 | C85 | C100 | C99 | -7.8 |  | C204 | C214 | C215 | C195 | 1.5(14) |
| C82 | C73 | C74 | C75 | 145.1 |  | C204 | C214 | C215 | C237 | 163.3(8) |
| C82 | C73 | C74 | C90 | -3.2 |  | C204 | C214 | C216 | S6 | -167.6(7) |
| C82 | C81 | C85 | C84 | -6.0 |  | C204 | C214 | C216 | C238 | 6.8(15) |
| C82 | C81 | C85 | C100 | -141.4 |  | C204 | C234 | C240 | C232 | 170.1(9) |
| C82 | C83 | C84 | C85 | -0.9 |  | C204 | C234 | C240 | C238 | -0.9(15) |
| C82 | C83 | C84 | C95 | 140.8 |  | C205 | C200 | C220 | C213 | 158.4(8) |
| C82 | C83 | C86 | C87 | -127.3 |  | C205 | C200 | C220 | C230 | -2.0(11) |
| C82 | C83 | C86 | C90 | 10.5 |  | C205 | C223 | C230 | C220 | 17.2(9) |
| C83 | C84 | C85 | C81 | 4.2 |  | C205 | C223 | C230 | C224 | -150.5(9) |
| C83 | C84 | C85 | C100 | 138.5 |  | C206 | C197 | C202 | C201 | 158.2(8) |
| C83 | C84 | C95 | C91 | 2.2 |  | C206 | C197 | C202 | C211 | -0.9(13) |
| C83 | C84 | C95 | C94 | -137.0 |  | C206 | C199 | C209 | C193 | 162.1(8) |
| C83 | C86 | C87 | C88 | 139.1 |  | C206 | C199 | C209 | C203 | 0.1(13) |
| C83 | C86 | C87 | C91 | -8.8 |  | C206 | C204 | C214 | C215 | -4.6(14) |
| C83 | C86 | C90 | C74 | -12.5 |  | C206 | C204 | C214 | C216 | 156.9(9) |
| C83 | C86 | C90 | C89 | -144.9 |  | C206 | C204 | C234 | C211 | -3.0(14) |
| C84 | C83 | C86 | C87 | 6.6 |  | C206 | C204 | C234 | C240 | -160.0(9) |
| C84 | C83 | C86 | C90 | 144.4 |  | C207 | C213 | C220 | C200 | -158.8(8) |
| C84 | C85 | C100 | C96 | 5.3 |  | C207 | C213 | C220 | C230 | -1.3(13) |
| C84 | C85 | C100 | C99 | -138.6 |  | C207 | C219 | C224 | C230 | 2.1(13) |
| C85 | C81 | C82 | C73 | 143.1 |  | C208 | S4 | C226 | C201 | -5.5(6) |
| C85 | C81 | C82 | C83 | 5.5 |  | C208 | S4 | C226 | C235 | 159.7(8) |
| C85 | C84 | C95 | C91 | 139.6 |  | C208 | C192 | C198 | C194 | 153.8(8) |
| C85 | C84 | C95 | C94 | 0.5 |  | C208 | C192 | C198 | C200 | -2.4(12) |
| C86 | C83 | C84 | C85 | -144.9 |  | C208 | C192 | C201 | C202 | -160.9(8) |
| C86 | C83 | C84 | C95 | -3.1 |  | C208 | C192 | C201 | C226 | 1.0(10) |
| C86 | C87 | C88 | C89 | -0.3 |  | C209 | C193 | C210 | C212 | 0.2(13) |
| C86 | C87 | C88 | C133 | -149.0 |  | C209 | C193 | C210 | C221 | -153.9(8) |
| C86 | C87 | C91 | C92 | 146.5 |  | C209 | C193 | C218 | C196 | 155.3(8) |
| C86 | C87 | C91 | C95 | 7.5 |  | C209 | C193 | C218 | C207 | -5.5(13) |
| C87 | C86 | C90 | C74 | 135.7 |  | C209 | C199 | C206 | C197 | -0.4(13) |
| C87 | C86 | C90 | C89 | 3.3 |  | C209 | C199 | C206 | C204 | -161.2(8) |
| C87 | C88 | C89 | C90 | 2.3 |  | C209 | C203 | C213 | C207 | -4.2(12) |
| C87 | C88 | C89 | C132 | -145.3 |  | C209 | C203 | C213 | C220 | -163.4(8) |
| C87 | C88 | C133 | C112 | 149.3 |  | C210 | C193 | C209 | C199 | -0.3(12) |
| C87 | C88 | C133 | C134 | 4.9 |  | C210 | C193 | C209 | C203 | 162.1(8) |
| C87 | C91 | C92 | C93 | -149.6 |  | C210 | C193 | C218 | C196 | -2.3(12) |
| C87 | C91 | C92 | C134 | -2.9 |  | C210 | C193 | C218 | C207 | -163.0(8) |
| C87 | C91 | C95 | C84 | -5.4 |  | C210 | C212 | C217 | C222 | -7.8(9) |
| C87 | C91 | C95 | C94 | 140.4 |  | C210 | C212 | C217 | C242 | 158.5(7) |
| C88 | C87 | C91 | C92 | 7.8 |  | C210 | C221 | C222 | C217 | -15.7(8) |
| C88 | C87 | C91 | C95 | -131.2 |  | C210 | C221 | C222 | C227 | 98.5(8) |
| C88 | C89 | C90 | C74 | -146.2 |  | C210 | C221 | C222 | C239 | -139.8(8) |
| C88 | C89 | C90 | C86 | -3.5 |  | C210 | C221 | C225 | C196 | -0.6(12) |
| C88 | C89 | C132 | C113 | -8.8 |  | C211 | C229 | C232 | C236 | 142.9(9) |
| C88 | C89 | C132 | C131 | 131.5 |  | C211 | C229 | C232 | C240 | 18.5(10) |
| C88 | C133 | C134 | C92 | -1.0 |  | C211 | C229 | C232 | C241 | -95.1(8) |
| C88 | C133 | C134 | C110 | 145.7 |  | C211 | C229 | C235 | C226 | 2.7(14) |
| C89 | C88 | C133 | C112 | 5.2 |  | C211 | C234 | C240 | C232 | 11.5(11) |
| C89 | C88 | C133 | C134 | -139.3 |  | C211 | C234 | C240 | C238 | -159.4(9) |
| C90 | C74 | C75 | C71 | 134.6 |  | C212 | C195 | C199 | C206 | -162.0(8) |
| C90 | C74 | C75 | C131 | -6.0 |  | C212 | C195 | C199 | C209 | -0.1(12) |
| C90 | C86 | C87 | C88 | -1.7 |  | C212 | C195 | C215 | C214 | 162.1(8) |
| C90 | C86 | C87 | C91 | -149.6 |  | C212 | C195 | C215 | C237 | 2.2(12) |
| C90 | C89 | C132 | C113 | -150.6 |  | C212 | C210 | C221 | C222 | 12.4(9) |
| C90 | C89 | C132 | C131 | -10.3 |  | C212 | C210 | C221 | C225 | -158.8(8) |
| C91 | C87 | C88 | C89 | 139.0 |  | C212 | C217 | C222 | C221 | 14.0(8) |
| C91 | C87 | C88 | C133 | -9.6 |  | C212 | C217 | C222 | C227 | -98.2(8) |
| C91 | C92 | C93 | C94 | 2.1 |  | C212 | C217 | C222 | C239 | 138.0(7) |
| C91 | C92 | C93 | C135 | 148.2 |  | C212 | C217 | C242 | C237 | 3.6(12) |
| C91 | C92 | C134 | C110 | -148.7 |  | C213 | C203 | C209 | C193 | -0.9(12) |
| C91 | C92 | C134 | C133 | -0.1 |  | C213 | C203 | C209 | C199 | 161.2(8) |
| C92 | C91 | C95 | C84 | -145.8 |  | C213 | C207 | C218 | C193 | 0.3(13) |
| C92 | C91 | C95 | C94 | 0.1 |  | C213 | C207 | C218 | C196 | -162.0(8) |
| C92 | C93 | C94 | C95 | -2.2 |  | C213 | C207 | C219 | S5 | 167.0(7) |
| C92 | C93 | C94 | C96 | 142.0 |  | C213 | C207 | C219 | C224 | -4.7(13) |
| C92 | C93 | C135 | C106 | 5.6 |  | C213 | C220 | C230 | C223 | -170.1(8) |
| C92 | C93 | C135 | C136 | -135.2 |  | C213 | C220 | C230 | C224 | -1.0(14) |
| C93 | C92 | C134 | C110 | -5.8 |  | C214 | C204 | C206 | C197 | -157.8(8) |
| C93 | C92 | C134 | C133 | 142.8 |  | C214 | C204 | C206 | C199 | 3.5(13) |
| C93 | C94 | C95 | C84 | 143.6 |  | C214 | C204 | C234 | C211 | 159.8(9) |
| C93 | C94 | C95 | C91 | 1.3 |  | C214 | C204 | C234 | C240 | 2.9(14) |
| C93 | C94 | C96 | C97 | -1.2 |  | C214 | C215 | C237 | S6 | 5.0(10) |
| C93 | C94 | C96 | C100 | -139.1 |  | C214 | C215 | C237 | C242 | -160.9(8) |
| C93 | C135 | C136 | C97 | -1.3 |  | C214 | C216 | C238 | C240 | -4.3(15) |
| C93 | C135 | C136 | C101 | 147.9 |  | C215 | C195 | C199 | C206 | -3.5(12) |
| C94 | C93 | C135 | C106 | 149.1 |  | C215 | C195 | C199 | C209 | 158.3(8) |
| C94 | C93 | C135 | C136 | 8.3 |  | C215 | C195 | C212 | C210 | -159.3(8) |
| C94 | C96 | C97 | C98 | -139.5 |  | C215 | C195 | C212 | C217 | -1.6(12) |
| C94 | C96 | C97 | C136 | 8.9 |  | C215 | C214 | C216 | S6 | -5.2(10) |
| C94 | C96 | C100 | C85 | -4.4 |  | C215 | C214 | C216 | C238 | 169.2(9) |
| C94 | C96 | C100 | C99 | 144.7 |  | C215 | C237 | C242 | C217 | -3.0(12) |
| C95 | C84 | C85 | C81 | -137.5 |  | C216 | S6 | C237 | C215 | -6.7(7) |
| C95 | C84 | C85 | C100 | -3.2 |  | C216 | S6 | C237 | C242 | 157.6(8) |
| C95 | C91 | C92 | C93 | -1.3 |  | C216 | C214 | C215 | C195 | -161.6(8) |
| C95 | C91 | C92 | C134 | 145.4 |  | C216 | C214 | C215 | C237 | 0.2(12) |
| C95 | C94 | C96 | C97 | 139.5 |  | C216 | C238 | C240 | C232 | -166.3(10) |
| C95 | C94 | C96 | C100 | 1.6 |  | C216 | C238 | C240 | C234 | 1.6(15) |
| C96 | C94 | C95 | C84 | 0.3 |  | C218 | C193 | C209 | C199 | -156.7(8) |
| C96 | C94 | C95 | C91 | -142.1 |  | C218 | C193 | C209 | C203 | 5.7(12) |
| C96 | C97 | C98 | C99 | -2.0 |  | C218 | C193 | C210 | C212 | 158.0(8) |
| C96 | C97 | C98 | C137 | 148.3 |  | C218 | C193 | C210 | C221 | 3.9(12) |
| C96 | C97 | C136 | C101 | -155.1 |  | C218 | C196 | C225 | C221 | 2.0(12) |
| C96 | C97 | C136 | C135 | -7.0 |  | C218 | C207 | C213 | C203 | 4.6(13) |
| C97 | C96 | C100 | C85 | -149.0 |  | C218 | C207 | C213 | C220 | 165.1(8) |
| C97 | C96 | C100 | C99 | 0.1 |  | C218 | C207 | C219 | S5 | 5.3(9) |
| C97 | C98 | C99 | C79 | 144.6 |  | C218 | C207 | C219 | C224 | -166.3(8) |
| C97 | C98 | C99 | C100 | 2.0 |  | C219 | S5 | C196 | C218 | 7.5(7) |
| C97 | C98 | C137 | C102 | 13.0 |  | C219 | S5 | C196 | C225 | -158.4(8) |
| C97 | C98 | C137 | C138 | -137.5 |  | C219 | C207 | C213 | C203 | -156.5(8) |
| C98 | C97 | C136 | C101 | -10.2 |  | C219 | C207 | C213 | C220 | 4.0(12) |
| C98 | C97 | C136 | C135 | 137.8 |  | C219 | C207 | C218 | C193 | 162.7(8) |
| C98 | C99 | C100 | C85 | 147.1 |  | C219 | C207 | C218 | C196 | 0.4(11) |
| C98 | C99 | C100 | C96 | -1.4 |  | C219 | C224 | C230 | C220 | 0.6(12) |
| C98 | C137 | C138 | C78 | -3.1 |  | C219 | C224 | C230 | C223 | 166.8(8) |
| C98 | C137 | C138 | C122 | 145.5 |  | C220 | C200 | C205 | C223 | 13.8(9) |
| C99 | C79 | C80 | C76 | 137.1 |  | C220 | C200 | C205 | C228 | -158.2(7) |
| C99 | C79 | C80 | C81 | -10.9 |  | C221 | C210 | C212 | C195 | 157.7(8) |
| C99 | C98 | C137 | C102 | 160.0 |  | C221 | C210 | C212 | C217 | -3.0(10) |
| C99 | C98 | C137 | C138 | 9.4 |  | C222 | C217 | C242 | C237 | 166.4(8) |
| C100 | C96 | C97 | C98 | 1.1 |  | C222 | C221 | C225 | C196 | -169.4(8) |
| C100 | C96 | C97 | C136 | 149.5 |  | C223 | C205 | C228 | C208 | -170.1(7) |
| C101 | C102 | C103 | C104 | 2.2 |  | C225 | C196 | C218 | C193 | -0.5(13) |
| C101 | C102 | C103 | C123 | -139.9 |  | C225 | C196 | C218 | C207 | 161.4(8) |
| C101 | C102 | C137 | C98 | -17.4 |  | C225 | C221 | C222 | C217 | 154.0(9) |
| C101 | C102 | C137 | C138 | 129.6 |  | C225 | C221 | C222 | C227 | -91.9(11) |
| C101 | C105 | C107 | C106 | -3.5 |  | C225 | C221 | C222 | C239 | 29.9(12) |
| C101 | C105 | C107 | C108 | -141.6 |  | C226 | S4 | C208 | C192 | 6.1(6) |
| C102 | C101 | C105 | C104 | -0.5 |  | C226 | S4 | C208 | C228 | -162.8(7) |
| C102 | C101 | C105 | C107 | 145.4 |  | C226 | C201 | C202 | C197 | -156.2(8) |
| C102 | C101 | C136 | C97 | 5.8 |  | C226 | C201 | C202 | C211 | 3.6(12) |
| C102 | C101 | C136 | C135 | -143.7 |  | C228 | C205 | C223 | C230 | 152.1(8) |
| C102 | C103 | C104 | C105 | -2.5 |  | C228 | C205 | C223 | C231 | 30.8(11) |
| C102 | C103 | C104 | C130 | -148.8 |  | C228 | C205 | C223 | C233 | -92.6(10) |
| C102 | C103 | C123 | C122 | 2.4 |  | C229 | C211 | C234 | C204 | -159.2(9) |
| C102 | C103 | C123 | C124 | 139.7 |  | C229 | C211 | C234 | C240 | 0.7(12) |
| C102 | C137 | C138 | C78 | -146.8 |  | C229 | C232 | C240 | C234 | -18.1(10) |
| C102 | C137 | C138 | C122 | 1.8 |  | C229 | C232 | C240 | C238 | 150.8(11) |
| C103 | C102 | C137 | C98 | -151.3 |  | C231 | C223 | C230 | C220 | 138.2(8) |
| C103 | C102 | C137 | C138 | -4.3 |  | C231 | C223 | C230 | C224 | -29.4(12) |
| C103 | C104 | C105 | C101 | 1.8 |  | C232 | C229 | C235 | C226 | 170.1(10) |
| C103 | C104 | C105 | C107 | -145.4 |  | C233 | C223 | C230 | C220 | -97.5(8) |
| C103 | C104 | C130 | C126 | 5.9 |  | C233 | C223 | C230 | C224 | 94.8(11) |
| C103 | C104 | C130 | C129 | 135.7 |  | C234 | C204 | C206 | C197 | 3.8(13) |
| C103 | C123 | C124 | C125 | -145.0 |  | C234 | C204 | C206 | C199 | 165.1(9) |
| C103 | C123 | C124 | C126 | 0.6 |  | C234 | C204 | C214 | C215 | -167.3(9) |
| C104 | C103 | C123 | C122 | -136.4 |  | C234 | C204 | C214 | C216 | -5.8(14) |
| C104 | C103 | C123 | C124 | 0.9 |  | C234 | C211 | C229 | C232 | -13.0(11) |
| C104 | C105 | C107 | C106 | 138.8 |  | C234 | C211 | C229 | C235 | 157.6(9) |
| C104 | C105 | C107 | C108 | 0.7 |  | C235 | C229 | C232 | C236 | -25.6(15) |
| C105 | C101 | C102 | C103 | -1.1 |  | C235 | C229 | C232 | C240 | -150.0(11) |
| C105 | C101 | C102 | C137 | -135.7 |  | C235 | C229 | C232 | C241 | 96.4(13) |
| C105 | C101 | C136 | C97 | 146.2 |  | C236 | C232 | C240 | C234 | -143.0(9) |
| C105 | C101 | C136 | C135 | -3.3 |  | C236 | C232 | C240 | C238 | 25.9(15) |
| C105 | C104 | C130 | C126 | -136.4 |  | C237 | S6 | C216 | C214 | 6.8(8) |
| C105 | C104 | C130 | C129 | -6.6 |  | C237 | S6 | C216 | C238 | -166.7(10) |
| C105 | C107 | C108 | C109 | 143.9 |  | C241 | C232 | C240 | C234 | 98.2(9) |
| C105 | C107 | C108 | C129 | -0.4 |  | C241 | C232 | C240 | C238 | -92.8(14) |
| C106 | C107 | C108 | C109 | 1.2 |  | C242 | C217 | C222 | C221 | -150.0(8) |
| C106 | C107 | C108 | C129 | -143.2 |  | C242 | C217 | C222 | C227 | 97.8(10) |
| C106 | C110 | C134 | C92 | 9.6 |  | C242 | C217 | C222 | C239 | -26.0(11) |

Table 7 Hydrogen Atom Coordinates (Å×104) and Isotropic Displacement Parameters (Å2×103) for z\_sq\_tw.

| Atom | *x* | *y* | *z* | U(eq) |
| --- | --- | --- | --- | --- |
| H164 | -937.8 | 3554.91 | 1348.3 | 81 |
| H16A | 611.98 | 3489.38 | 1699.37 | 103 |
| H16B | 789.05 | 3935.68 | 1930.47 | 103 |
| H16C | 1277.43 | 3743.72 | 1718.14 | 103 |
| H173 | -3517.94 | 4481.64 | 1398.3 | 71 |
| H17A | -3749.94 | 5588.5 | 1865.53 | 96 |
| H17B | -2968.49 | 5340.56 | 2051.54 | 96 |
| H17C | -3774.97 | 5117.01 | 1863.81 | 96 |
| H175 | -3447.68 | 6217.76 | 1461.79 | 62 |
| H17D | 953.17 | 6703.27 | 2067.84 | 99 |
| H17E | 716.42 | 7156.7 | 1886.86 | 99 |
| H17F | 1398.38 | 6956.65 | 1867.03 | 99 |
| H177 | -833.04 | 7128.06 | 1460.45 | 71 |
| H182 | 1578.99 | 6254.23 | 1421.2 | 69 |
| H18A | 624.95 | 7142.81 | 762.93 | 108 |
| H18B | -52.29 | 7361.38 | 770.85 | 108 |
| H18C | -319.92 | 7031.52 | 277.73 | 108 |
| H18D | -5064.55 | 5127.7 | 785.66 | 98 |
| H18E | -5048.28 | 5356.91 | 299.67 | 98 |
| H18F | -5036.14 | 5599.15 | 789.48 | 98 |
| H189 | 1495.93 | 4415.78 | 1296.41 | 71 |
| H19A | 464.82 | 3597.32 | 569.62 | 101 |
| H19B | -486.29 | 3706.98 | 126.72 | 101 |
| H19C | -176.67 | 3348.18 | 585.87 | 101 |
| H224 | 4070.28 | 1067.6 | 6237.73 | 73 |
| H225 | 1581.67 | 1947.29 | 6427.83 | 74 |
| H22A | 53.96 | 3085.45 | 5818.29 | 113 |
| H22B | 50.94 | 2634.44 | 5982.31 | 113 |
| H22C | 53.84 | 2740.27 | 5433.48 | 113 |
| H228 | 6664.25 | 1990.67 | 6479.83 | 67 |
| H23A | 6331.8 | 1266.67 | 6703.89 | 101 |
| H23B | 5621.56 | 1027.34 | 6646.73 | 101 |
| H23C | 5867.63 | 1458.85 | 6935.77 | 101 |
| H23D | 4903.82 | 949.43 | 5525.35 | 100 |
| H23E | 5629.73 | 1193.54 | 5611.05 | 100 |
| H23F | 4703.53 | 1336.94 | 5134.91 | 100 |
| H235 | 6807.53 | 3816.21 | 6800.06 | 79 |
| H23G | 6653.3 | 4482.2 | 7261.18 | 150 |
| H23H | 6196.76 | 4239.63 | 7462.2 | 150 |
| H23I | 5994.95 | 4696.7 | 7294.62 | 150 |
| H238 | 4403.61 | 4631.93 | 6893.08 | 90 |
| H23J | 1230.98 | 2948 | 6991.97 | 114 |
| H23K | 2116.76 | 2830.89 | 7195.73 | 114 |
| H23L | 1444.36 | 2496.28 | 6981.71 | 114 |
| H24A | 5912.83 | 4763.47 | 6278.32 | 137 |
| H24B | 5108.74 | 4910.97 | 6185.67 | 137 |
| H24C | 5034.03 | 4602.28 | 5727.42 | 137 |
| H242 | 1744.34 | 3658.75 | 6740.48 | 83 |

Experimental

Single crystals of C121S3H24
[z\_sq\_tw]
were
[].
A suitable crystal was selected and
[]
on a
Bruker APEX-II CCD
diffractometer. The crystal was kept at 293(2) K during data collection.
Using Olex2 [1], the structure was solved with the
Unknown
[2] structure solution program using
Unknown
and refined with the
Unknown
[3] refinement package using
Unknown
minimisation.

1. Dolomanov, O.V., Bourhis, L.J., Gildea, R.J, Howard, J.A.K. & Puschmann, H.
   (2009), J. Appl. Cryst. 42, 339-341.

Crystal structure determination of
[z\_sq\_tw]

**Crystal Data**
for C121S3H24 (*M*=1573.58 g/mol):
monoclinic, space group P21/c (no. 14),
*a* = 20.312(4) Å, *b* = 33.234(9) Å, *c* = 27.770(6) Å, *β* = 127.915(13)°,
*V*= 14789(6) Å3,
*Z* = 8,
*T* = 293(2) K,
μ(CuKα) = 1.396 mm-1,
*Dcalc* = 1.413 g/cm3,
25643 reflections measured (4.83° ≤ 2Θ ≤ 132.098°),
25643 unique (*R*int = ?, Rsigma = 0.0899) which were used in all calculations.
The final *R*1 was 0.1516
(I > 2σ(I)) and *wR*2 was 0.3173 (all data).

Refinement model description

Number of restraints - 12433,
number of constraints - unknown.

Details:

```
1. Twinned data refinement
```

This report has been created with Olex2, compiled on
2023.03.06 svn.rbb2c1857 for OlexSys. Please
let us know
if there are any errors or if you would like to have additional features.
