# Supplementary material for: Trichalcogenasupersumanenes and its concave-convex supramolecular assembly with fullerenes
Source: Nat Commun. 2023 Jun 10;14:3446. doi: 10.1038/s41467-023-39086-0 (PMC10257710; doi:10.1038/s41467-023-39086-0)

checkCIF/PLATON report

Structure factors have been supplied for datablock(s) s_pl

THIS REPORT IS FOR GUIDANCE ONLY. IF USED AS PART OF A REVIEW PROCEDURE FOR PUBLICATION, IT SHOULD NOT REPLACE THE EXPERTISE OF AN EXPERIENCED CRYSTALLOGRAPHIC REFEREE.

No syntax errors found. CIF dictionary Interpreting this report

Datablock: s_pl

```
Bond precision:  C-C = 0.0036 A          Wavelength=1.54178

Cell:           a=16.5069(9)    b=23.6255(13)      c=29.7702(15)
                alpha=90        beta=103.380(2)    gamma=90
Temperature:    153 K

                Calculated                Reported
Volume          11294.8(10)               11294.8(10)
Space group     P 21/c                    P 1 21/c 1
Hall group      -P 2ybc                   -P 2ybc
Moiety formula  C69 H60 Se3, C H Cl3      C69 H60 Se3, C H Cl3
Sum formula     C70 H61 Cl3 Se3           C70 H61 Cl3 Se3
Mr              1245.42                   1245.41
Dx,g cm-3       1.465                     1.465
Z               8                         8
Mu (mm-1)       4.006                     4.006
F000            5072.0                    5072.0
F000'           5071.31
h,k,lmax        19,28,35                  19,28,35
Nref            20709                     20658
Tmin,Tmax       0.183,0.301               0.401,0.753
Tmin'           0.108

Correction method= # Reported T Limits: Tmin=0.401 Tmax=0.753
AbsCorr = MULTI-SCAN

Data completeness= 0.998          Theta(max)= 68.305

R(reflections)= 0.0363( 19015)    wR2(reflections)= 0.0979( 20658)

S = 1.027                   Npar= 1457
```

The following ALERTS were generated. Each ALERT has the format
test-name_ALERT_alert-type_alert-level.
Click on the hyperlinks for more details of the test.

Alert level C

```
PLAT220_ALERT_2_C NonSolvent   Resd 1  C   Ueq(max)/Ueq(min) Range        5.6 Ratio
PLAT220_ALERT_2_C NonSolvent   Resd 2  C   Ueq(max)/Ueq(min) Range        4.5 Ratio
PLAT222_ALERT_3_C NonSolvent Resd 1  H   Uiso(max)/Uiso(min) Range        5.1 Ratio
PLAT234_ALERT_4_C Large Hirshfeld Difference C67      --C141     .       0.17 Ang.
PLAT241_ALERT_2_C High   'MainMol' Ueq as Compared to Neighbors of        C67 Check
PLAT360_ALERT_2_C Short  C(sp3)-C(sp3) Bond  C136     - C138     .       1.40 Ang.
PLAT601_ALERT_2_C Unit Cell Contains Solvent Accessible VOIDS of .         35 Ang**3
PLAT911_ALERT_3_C Missing FCF Refl Between Thmin & STh/L=    0.600         10 Report
```

Alert level G

```
PLAT002_ALERT_2_G Number of Distance or Angle Restraints on AtSite         18 Note
PLAT083_ALERT_2_G SHELXL Second Parameter in WGHT  Unusually Large      14.50 Why ?
PLAT176_ALERT_4_G The CIF-Embedded .res File Contains SADI Records         13 Report
PLAT230_ALERT_2_G Hirshfeld Test Diff for    C67      --C69      .       14.0 s.u.
PLAT231_ALERT_4_G Hirshfeld Test (Solvent)   Cl1      --C139     .        6.8 s.u.
PLAT301_ALERT_3_G Main Residue  Disorder .............(Resd  1  )          1% Note
PLAT302_ALERT_4_G Anion/Solvent/Minor-Residue Disorder (Resd  3  )        75% Note
PLAT302_ALERT_4_G Anion/Solvent/Minor-Residue Disorder (Resd  4  )       100% Note
PLAT302_ALERT_4_G Anion/Solvent/Minor-Residue Disorder (Resd  5  )       100% Note
PLAT304_ALERT_4_G Non-Integer Number of Atoms in ..... (Resd  4  )       2.92 Check
PLAT304_ALERT_4_G Non-Integer Number of Atoms in ..... (Resd  5  )       2.08 Check
PLAT333_ALERT_2_G Large Aver C6-Ring C-C Dist C1       -C8       .       1.42 Ang.
PLAT333_ALERT_2_G Large Aver C6-Ring C-C Dist C1       -C6       .       1.42 Ang.
PLAT333_ALERT_2_G Large Aver C6-Ring C-C Dist C2       -C18      .       1.42 Ang.
PLAT333_ALERT_2_G Large Aver C6-Ring C-C Dist C3       -C12      .       1.42 Ang.
PLAT333_ALERT_2_G Large Aver C6-Ring C-C Dist C70      -C73      .       1.42 Ang.
PLAT333_ALERT_2_G Large Aver C6-Ring C-C Dist C70      -C82      .       1.42 Ang.
PLAT333_ALERT_2_G Large Aver C6-Ring C-C Dist C72      -C88      .       1.42 Ang.
PLAT333_ALERT_2_G Large Aver C6-Ring C-C Dist C73      -C81      .       1.42 Ang.
PLAT432_ALERT_2_G Short Inter X...Y Contact  Cl1      ..C135             3.21 Ang.
                                              2-x,1-y,1-z  =      3_766 Check
PLAT860_ALERT_3_G Number of Least-Squares Restraints ............          13 Note
PLAT912_ALERT_4_G Missing # of FCF Reflections Above STh/L=  0.600         41 Note
PLAT913_ALERT_3_G Missing # of Very Strong Reflections in FCF ....          1 Note
PLAT933_ALERT_2_G Number of OMIT Records in Embedded .res File ...          6 Note
PLAT978_ALERT_2_G Number C-C Bonds with Positive Residual Density.          0 Info
```

---

0 **ALERT level A** = Most likely a serious problem - resolve or explain
0 **ALERT level B** = A potentially serious problem, consider carefully
8 **ALERT level C** = Check. Ensure it is not caused by an omission or oversight
25 **ALERT level G** = General information/check it is not something unexpected

0 ALERT type 1 CIF construction/syntax error, inconsistent or missing data
19 ALERT type 2 Indicator that the structure model may be wrong or deficient
5 ALERT type 3 Indicator that the structure quality may be low
9 ALERT type 4 Improvement, methodology, query or suggestion
0 ALERT type 5 Informative message, check

It is advisable to attempt to resolve as many as possible of the alerts in all categories. Often the minor alerts point to easily fixed oversights, errors and omissions in your CIF or refinement strategy, so attention to these fine details can be worthwhile. In order to resolve some of the more serious problems it may be necessary to carry out additional measurements or structure refinements. However, the purpose of your study may justify the reported deviations and the more serious of these should normally be commented upon in the discussion or experimental section of a paper or in the "special_details" fields of the CIF. checkCIF was carefully designed to identify outliers and unusual parameters, but every test has its limitations and alerts that are not important in a particular case may appear. Conversely, the absence of alerts does not guarantee there are no aspects of the results needing attention. It is up to the individual to critically assess their own results and, if necessary, seek expert advice.

Publication of your CIF in IUCr journals

A basic structural check has been run on your CIF. These basic checks will be run on all CIFs submitted for publication in IUCr journals (*Acta Crystallographica*, *Journal of Applied Crystallography*, *Journal of Synchrotron Radiation*); however, if you intend to submit to *Acta Crystallographica Section C* or *E* or *IUCrData*, you should make sure that full publication checks are run on the final version of your CIF prior to submission.

Publication of your CIF in other journals

Please refer to the *Notes for Authors* of the relevant journal for any special instructions relating to CIF submission.

PLATON version of 16/07/2020; check.def file version of 12/07/2020

Datablock s_pl - ellipsoid plot

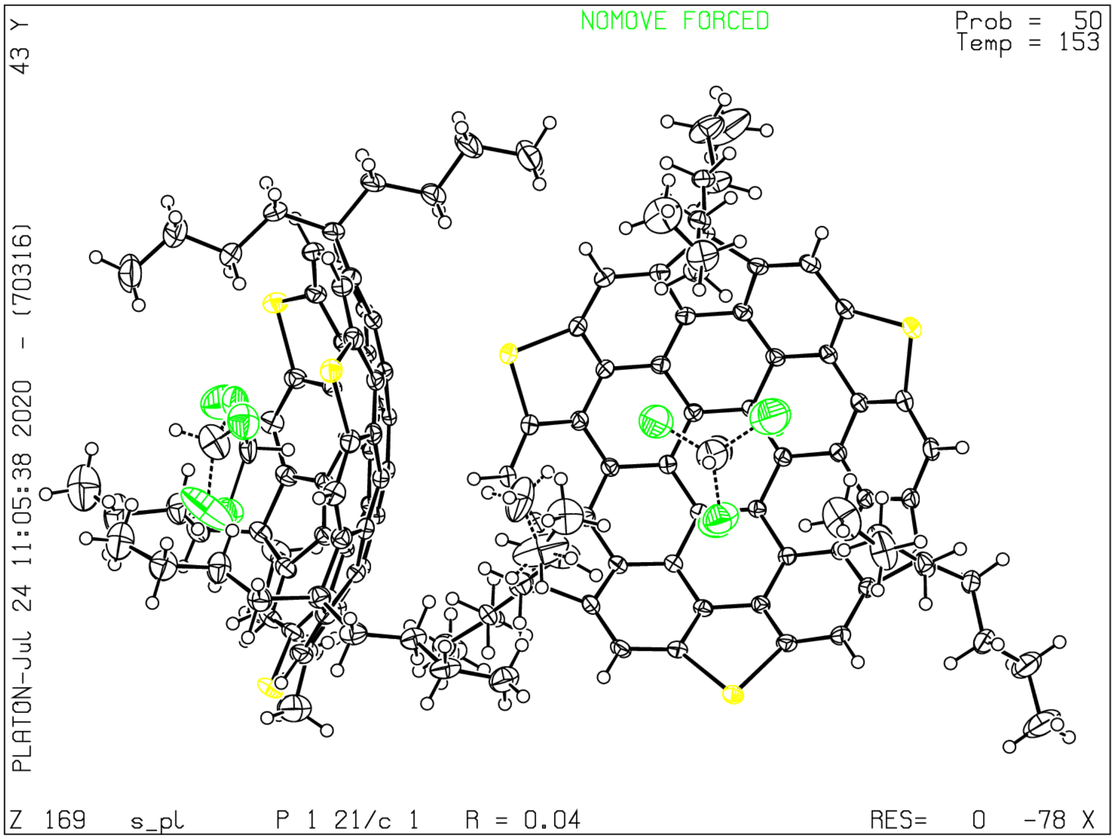

Supplement: Supplementary file 4 — Supplementary Data 1 [file 41467_2023_39086_MOESM4_ESM.zip › 1b/1b_cifreport.pdf]
