# Supplementary material for: Trichalcogenasupersumanenes and its concave-convex supramolecular assembly with fullerenes
Source: Nat Commun. 2023 Jun 10;14:3446. doi: 10.1038/s41467-023-39086-0 (PMC10257710; doi:10.1038/s41467-023-39086-0)
Supplement: Supplementary file 4 — Supplementary Data 1 [file 41467_2023_39086_MOESM4_ESM.zip › 1b/1b_tables.html]

1b


# 1b

Table 1 Crystal data and structure refinement for 1b.

| Identification code | 1b |
| Empirical formula | C70H61Cl3Se3 |
| Formula weight | 1245.41 |
| Temperature/K | 153.15 |
| Crystal system | monoclinic |
| Space group | P21/c |
| a/Å | 16.5069(9) |
| b/Å | 23.6255(13) |
| c/Å | 29.7702(15) |
| α/° | 90 |
| β/° | 103.380(2) |
| γ/° | 90 |
| Volume/Å3 | 11294.8(10) |
| Z | 8 |
| ρcalcg/cm3 | 1.465 |
| μ/mm‑1 | 4.006 |
| F(000) | 5072.0 |
| Crystal size/mm3 | 0.5 × 0.4 × 0.3 |
| Radiation | CuKα (λ = 1.54178) |
| 2Θ range for data collection/° | 4.826 to 136.61 |
| Index ranges | -19 ≤ h ≤ 19, -28 ≤ k ≤ 28, -35 ≤ l ≤ 35 |
| Reflections collected | 189744 |
| Independent reflections | 20658 [Rint = 0.0531, Rsigma = 0.0253] |
| Data/restraints/parameters | 20658/13/1457 |
| Goodness-of-fit on F2 | 1.027 |
| Final R indexes [I>=2σ (I)] | R1 = 0.0363, wR2 = 0.0952 |
| Final R indexes [all data] | R1 = 0.0394, wR2 = 0.0979 |
| Largest diff. peak/hole / e Å-3 | 1.23/-0.94 |

Table 2 Fractional Atomic Coordinates (×104) and Equivalent Isotropic Displacement Parameters (Å2×103) for 1b. Ueq is defined as 1/3 of the trace of the orthogonalised UIJ tensor.

| Atom | *x* | *y* | *z* | U(eq) |
| --- | --- | --- | --- | --- |
| Se1 | 5206.4(2) | 7188.9(2) | 5481.5(2) | 28.29(7) |
| Se2 | 2569.2(2) | 9220.0(2) | 2542.7(2) | 28.12(7) |
| Se3 | 1222.6(2) | 10342.2(2) | 5536.9(2) | 29.81(7) |
| C1 | 1592.9(13) | 8598.4(9) | 3972.9(7) | 18.0(4) |
| C2 | 2323.2(13) | 8010.8(9) | 4801.1(7) | 20.0(4) |
| C3 | 1758.1(13) | 8461.8(9) | 4810.0(7) | 19.3(4) |
| C4 | 2159.9(13) | 8149.4(9) | 3965.4(7) | 18.7(4) |
| C5 | 2530.9(13) | 7856.4(9) | 4379.6(7) | 18.9(4) |
| C6 | 1392.5(13) | 8980.9(9) | 3583.8(7) | 19.6(4) |
| C7 | 3799.4(14) | 7362.3(9) | 4820.5(8) | 22.1(4) |
| C8 | 1395.9(13) | 8757.3(9) | 4396.7(8) | 19.6(4) |
| C9 | 2359.1(13) | 8491.9(10) | 3206.4(7) | 20.5(4) |
| C10 | 1021.8(13) | 9497.0(10) | 3628.1(8) | 21.0(4) |
| C11 | 3803.3(14) | 7829.9(10) | 3317.4(8) | 23.7(5) |
| C12 | 1719.9(13) | 8715.0(10) | 5247.7(8) | 21.3(4) |
| C13 | 2897.2(14) | 8551.9(10) | 2909.2(8) | 23.1(5) |
| C14 | 3628.0(14) | 8220.9(10) | 2957.7(8) | 24.6(5) |
| C15 | 1782.3(13) | 8935.1(10) | 3211.7(7) | 20.5(4) |
| C16 | 1037.7(14) | 9555.6(10) | 4859.6(8) | 23.3(5) |
| C17 | 3265.2(14) | 7516.8(9) | 4398.2(8) | 20.3(4) |
| C18 | 2856.7(14) | 7817.3(9) | 5230.5(8) | 21.3(4) |
| C19 | 3235.2(14) | 7769.0(9) | 3600.7(8) | 21.4(4) |
| C20 | 3590.3(14) | 7514.7(9) | 5239.7(8) | 22.3(4) |
| C21 | 2799.7(14) | 8069.8(10) | 5644.5(8) | 23.1(5) |
| C22 | 1013.2(13) | 9301.0(10) | 4430.6(8) | 20.5(4) |
| C23 | 2232.7(14) | 8514.3(10) | 5654.2(8) | 23.0(5) |
| C24 | 1036.7(14) | 9970.2(10) | 3346.1(8) | 24.2(5) |
| C25 | 835.6(13) | 9658.9(10) | 4050.5(8) | 21.9(4) |
| C26 | 2527.3(13) | 8096.7(9) | 3569.2(7) | 19.0(4) |
| C27 | 1388.9(14) | 9260.4(10) | 5273.3(8) | 23.5(5) |
| C28 | 720.8(14) | 10244.7(10) | 4070.4(8) | 25.1(5) |
| C29 | 3601.4(14) | 7478.5(9) | 4011.9(8) | 22.3(4) |
| C30 | 4622.9(15) | 7207.1(10) | 4844.3(8) | 25.4(5) |
| C31 | 1586.2(15) | 9575.3(11) | 5682.2(8) | 26.9(5) |
| C32 | 921.7(14) | 10137.9(10) | 4894.5(8) | 26.2(5) |
| C33 | 3929.7(16) | 8910.4(11) | 6573.0(8) | 30.3(5) |
| C34 | 751.7(15) | 10495.6(10) | 4499.1(9) | 27.3(5) |
| C35 | 2119.6(15) | 9355.3(11) | 6089.6(8) | 28.5(5) |
| C36 | 4952.5(15) | 7193.0(10) | 4443.0(8) | 27.4(5) |
| C37 | 1424.0(15) | 9918.8(10) | 2976.8(8) | 26.0(5) |
| C38 | 4227.8(15) | 7487.2(10) | 5642.3(8) | 25.4(5) |
| C39 | 3425.6(15) | 8055.9(10) | 6052.4(8) | 25.3(5) |
| C40 | 3200.6(15) | 8504.2(11) | 6388.2(8) | 26.1(5) |
| C41 | 1796.4(14) | 9392.8(10) | 2916.5(8) | 24.2(5) |
| C42 | 2453.2(14) | 8818.6(11) | 6067.3(8) | 25.4(5) |
| C43 | 4426.9(15) | 7334.3(10) | 4023.6(8) | 25.9(5) |
| C44 | 736.8(15) | 10490.7(10) | 3584.5(8) | 27.0(5) |
| C45 | 4606.0(15) | 7501.6(11) | 3548.2(8) | 27.6(5) |
| C46 | -163.6(16) | 10656.9(12) | 3330.8(9) | 32.7(6) |
| C47 | 4152.0(15) | 7754.1(10) | 6057.3(8) | 27.5(5) |
| C48 | 4739.7(17) | 6975.0(12) | 3264.4(9) | 36.3(6) |
| C49 | 2947.7(16) | 8217.0(12) | 6802.5(8) | 30.3(5) |
| C50 | 5390.6(16) | 7875.7(13) | 3617.1(10) | 38.5(6) |
| C51 | 4293.1(18) | 9202.9(13) | 6205.9(10) | 39.0(6) |
| C52 | 1336.4(17) | 10993.5(11) | 3613.1(10) | 33.9(6) |
| C53 | 4035(2) | 6547.4(12) | 3169.6(10) | 41.1(7) |
| C54 | 2160.1(18) | 7865.8(14) | 6692.9(10) | 43.2(7) |
| C55 | 2214.4(18) | 10896.3(13) | 3903.0(11) | 40.2(6) |
| C56 | 4242(2) | 6028.1(14) | 2915.9(10) | 50.9(8) |
| C57 | -254.9(19) | 10886.7(14) | 2840.7(10) | 45.6(7) |
| C58 | 3517(3) | 5614.0(14) | 2797.9(12) | 63.0(10) |
| C59 | 2797(2) | 11380.7(14) | 3868.0(15) | 58.8(9) |
| C60 | 2023(2) | 7541.5(17) | 7106.2(12) | 58.4(10) |
| C61 | 5384(2) | 8414.4(16) | 3885.2(12) | 55.5(9) |
| C62 | 4884(2) | 9684.2(15) | 6396.5(13) | 55.5(8) |
| C63 | -1150(2) | 10924(2) | 2574.6(12) | 65.1(11) |
| C64 | 3648(2) | 11305(2) | 4196.2(18) | 83.2(14) |
| C65 | 5599(3) | 9527.6(19) | 6782.2(15) | 72.5(11) |
| C66 | 1192(3) | 7256(2) | 7039.9(17) | 94.3(18) |
| C67 | 6236(3) | 8699(3) | 4009.9(16) | 102(2) |
| C68 | -1239(3) | 11185(3) | 2094.2(15) | 102(2) |
| C69 | 6395(4) | 9118(3) | 4325(2) | 67(2) |
| Se4 | 9939.8(2) | 7894.5(2) | 5760.5(2) | 31.30(7) |
| Se5 | 7493.0(2) | 5785.6(2) | 2819.2(2) | 33.33(7) |
| Se6 | 6399.0(2) | 4484.9(2) | 5860.6(2) | 33.57(7) |
| C70 | 7015.6(13) | 6764.6(10) | 4268.8(7) | 21.5(4) |
| C71 | 6520.7(13) | 6276.8(10) | 4282.9(7) | 21.2(4) |
| C72 | 6357.4(13) | 6094.5(10) | 4709.1(7) | 21.4(4) |
| C73 | 7347.7(14) | 7074.4(10) | 4679.9(8) | 22.7(5) |
| C74 | 6728.3(14) | 5980.0(10) | 3515.4(8) | 23.9(5) |
| C75 | 7175.8(14) | 6895.5(10) | 5105.3(8) | 22.7(5) |
| C76 | 6683.9(13) | 6404.6(10) | 5119.1(7) | 22.4(5) |
| C77 | 6368.9(14) | 5892.6(10) | 3894.0(7) | 22.3(5) |
| C78 | 7228.4(14) | 6463.5(10) | 3502.8(8) | 24.7(5) |
| C79 | 8340.0(15) | 7535.0(10) | 4300.9(8) | 25.9(5) |
| C80 | 6119.2(14) | 5263.4(11) | 5178.0(8) | 25.2(5) |
| C81 | 8024.1(14) | 7464.4(10) | 4690.9(8) | 23.7(5) |
| C82 | 7354.6(14) | 6856.9(10) | 3866.6(8) | 22.6(5) |
| C83 | 8545.3(15) | 7647.4(10) | 5110.6(8) | 26.4(5) |
| C84 | 6150.4(15) | 4891.1(11) | 3660.4(8) | 27.5(5) |
| C85 | 6696.5(14) | 6138.2(10) | 5558.6(8) | 23.9(5) |
| C86 | 8302.8(16) | 6899.8(11) | 6350.7(8) | 29.2(5) |
| C87 | 7688.3(14) | 7116.2(10) | 5531.5(8) | 24.4(5) |
| C88 | 6056.6(13) | 5527.6(10) | 4747.9(8) | 22.9(5) |
| C89 | 9332.0(15) | 7864.3(10) | 5123.3(9) | 28.8(5) |
| C90 | 8004.2(15) | 7234.8(10) | 3890.0(8) | 25.4(5) |
| C91 | 8417.6(16) | 6842.5(12) | 3231.1(8) | 31.2(5) |
| C92 | 8372.2(15) | 7468.9(10) | 5533.3(8) | 26.1(5) |
| C93 | 6500.9(16) | 4986.0(11) | 3281.8(9) | 30.4(5) |
| C94 | 8979.7(16) | 7248.0(11) | 6350.0(8) | 30.4(5) |
| C95 | 7675.1(15) | 6850.6(11) | 5944.8(8) | 25.9(5) |
| C96 | 5900.2(15) | 4576.8(11) | 4391.5(8) | 27.7(5) |
| C97 | 6076.0(14) | 5352.0(10) | 3944.5(8) | 23.8(5) |
| C98 | 7750.6(15) | 6458.4(11) | 3191.1(8) | 27.2(5) |
| C99 | 6442.0(14) | 5575.6(11) | 5587.7(8) | 24.7(5) |
| C100 | 5981.7(16) | 4340.4(11) | 3913.4(9) | 29.4(5) |
| C101 | 7182.6(15) | 6368.1(11) | 5960.4(8) | 25.7(5) |
| C102 | 9129.4(15) | 7749.3(11) | 4299.9(9) | 28.6(5) |
| C103 | 8552.5(15) | 7237.1(11) | 3593.9(8) | 28.6(5) |
| C104 | 5927.2(14) | 5167.0(10) | 4368.4(8) | 24.2(5) |
| C105 | 9638.9(16) | 7925.8(11) | 4717.9(9) | 31.6(5) |
| C106 | 6076.7(15) | 4680.0(11) | 5215.4(8) | 28.7(5) |
| C107 | 6687.8(15) | 5266.4(11) | 5998.6(8) | 28.7(5) |
| C108 | 7193.5(15) | 5514.8(12) | 6402.1(8) | 30.7(5) |
| C109 | 9013.6(16) | 7525.8(11) | 5933.7(9) | 29.8(5) |
| C110 | 5965.3(16) | 4319.4(11) | 4822.8(9) | 30.8(5) |
| C111 | 8134.2(16) | 6441.3(12) | 6694.9(8) | 31.1(5) |
| C112 | 8915.2(17) | 6093.0(12) | 6905.2(8) | 34.8(6) |
| C113 | 6784.4(15) | 5535.2(11) | 3212.8(8) | 29.2(5) |
| C114 | 9298.2(16) | 7629.1(11) | 3814.1(9) | 31.2(5) |
| C115 | 8565(2) | 9103.5(12) | 3239.2(9) | 41.3(7) |
| C116 | 8482.6(17) | 8523.7(11) | 3450.6(9) | 34.1(6) |
| C117 | 10144.5(16) | 7340.9(13) | 3853.5(10) | 37.5(6) |
| C118 | 7447.5(15) | 6072.6(12) | 6377.5(8) | 29.2(5) |
| C119 | 9322.9(18) | 5785.9(13) | 6565.0(10) | 38.9(6) |
| C120 | 6727.4(17) | 3933.4(11) | 3975.8(10) | 34.7(6) |
| C121 | 9288.8(17) | 8189.5(12) | 3541.6(9) | 37.0(6) |
| C122 | 5168.7(17) | 4037.1(14) | 3671.0(10) | 39.5(6) |
| C123 | 7801.3(17) | 6719.4(14) | 7090.3(9) | 38.8(6) |
| C124 | 5114.4(17) | 3866.1(12) | 3165.8(9) | 36.4(6) |
| C125 | 7563.6(18) | 4189.4(13) | 4229.1(12) | 45.7(7) |
| C126 | 10263.8(18) | 6770.6(13) | 4091.8(12) | 45.3(7) |
| C127 | 7044.9(19) | 7103.2(14) | 6945.5(10) | 42.7(7) |
| C128 | 7743(2) | 9415.6(13) | 3098.0(11) | 52.4(8) |
| C129 | 11177(2) | 6582.4(15) | 4175.4(13) | 53.8(8) |
| C130 | 6788(2) | 7369.6(19) | 7354.3(11) | 62.2(10) |
| C131 | 4575(2) | 4259.2(16) | 2833.5(11) | 53.6(8) |
| C132 | 6068(2) | 7778.3(17) | 7221.4(13) | 59.4(9) |
| C133 | 10090(2) | 5462.9(17) | 6797.5(13) | 59.1(9) |
| C134 | 10479(2) | 5130.6(18) | 6469.5(16) | 71.9(11) |
| C135 | 11299(3) | 5987.4(17) | 4357.5(16) | 68.0(10) |
| C136 | 8311(2) | 3818.6(19) | 4219.9(18) | 76.2(12) |
| C137 | 4597(3) | 4153(2) | 2331.1(13) | 75.5(12) |
| C138 | 8317(3) | 3282(2) | 4417(2) | 95.0(16) |
| Cl1 | 8399(5) | 5275(3) | 5239(2) | 108(2) |
| Cl2 | 9397.4(6) | 6293.8(4) | 5209.2(4) | 67.4(2) |
| Cl3 | 8673(3) | 5692(2) | 4375.7(13) | 61.2(10) |
| C139 | 8608(5) | 5854(3) | 4907(2) | 45.3(19) |
| Cl4 | 3153(2) | 9758(2) | 4903.9(17) | 66.2(9) |
| Cl5 | 4301(3) | 8838.3(15) | 4916.6(17) | 64.2(9) |
| Cl6 | 3536(4) | 9369(4) | 4047(3) | 55.1(11) |
| C140 | 3535(6) | 9258(4) | 4635(2) | 61(2) |
| C141 | 6651(7) | 8600(5) | 4461(4) | 79(5) |
| Cl7 | 8287(2) | 5368.4(19) | 5229.1(17) | 51.6(9) |
| Cl8 | 8912(6) | 5555(4) | 4410(3) | 116(2) |
| C142 | 9030(7) | 5639(4) | 4977(3) | 66(3) |
| Cl9 | 3373(8) | 9888(5) | 4951(3) | 118(3) |
| Cl10 | 3543(7) | 9434(6) | 4099(4) | 70(3) |
| C143 | 3938(6) | 9483(4) | 4654(3) | 50(3) |
| Cl11 | 4510(6) | 8997(6) | 4955(3) | 106(3) |

Table 3 Anisotropic Displacement Parameters (Å2×103) for 1b. The Anisotropic displacement factor exponent takes the form: -2π2[h2a\*2U11+2hka\*b\*U12+…].

| Atom | U11 | U22 | U33 | U23 | U13 | U12 |
| --- | --- | --- | --- | --- | --- | --- |
| Se1 | 25.43(13) | 33.61(14) | 24.91(13) | 2.73(10) | 3.97(10) | 11.07(10) |
| Se2 | 32.08(14) | 32.08(14) | 24.69(13) | 7.59(10) | 15.76(10) | 4.79(10) |
| Se3 | 32.46(14) | 31.03(14) | 27.24(13) | -5.42(10) | 9.60(11) | 8.55(11) |
| C1 | 15.8(10) | 20.4(10) | 19.0(10) | 0.4(8) | 6.0(8) | -2.4(8) |
| C2 | 20.4(10) | 19.9(11) | 20.4(11) | 2.0(8) | 6.2(8) | -1.8(8) |
| C3 | 17.1(10) | 21.8(11) | 20.4(10) | 0.2(8) | 7.4(8) | -1.7(8) |
| C4 | 17.7(10) | 19.3(10) | 19.5(10) | -0.7(8) | 5.5(8) | -3.7(8) |
| C5 | 17.8(10) | 18.0(10) | 21.6(11) | 0.5(8) | 6.0(8) | -1.8(8) |
| C6 | 15.6(10) | 23.9(11) | 19.0(10) | 1.1(8) | 3.7(8) | -0.8(8) |
| C7 | 24.0(11) | 19.0(11) | 23.9(11) | 2.5(9) | 7.1(9) | 1.5(9) |
| C8 | 16.0(10) | 21.9(11) | 22.2(11) | 0.9(8) | 7.1(8) | -1.1(8) |
| C9 | 20.0(10) | 24.2(11) | 17.4(10) | -2.7(8) | 4.5(8) | -1.8(9) |
| C10 | 16.6(10) | 25.5(11) | 21.3(11) | 2.8(9) | 5.5(8) | 1.6(9) |
| C11 | 23.4(11) | 26.3(12) | 22.9(11) | -3.8(9) | 8.3(9) | 3.9(9) |
| C12 | 19.3(10) | 26.5(11) | 19.9(11) | 0.7(9) | 8.3(8) | -0.9(9) |
| C13 | 25.7(11) | 26.3(12) | 18.3(10) | 1.0(9) | 7.3(9) | 0.5(9) |
| C14 | 25.5(11) | 30.2(12) | 21.0(11) | -1.7(9) | 11.3(9) | 0.7(10) |
| C15 | 18.4(10) | 25.0(11) | 18.7(10) | 0.1(8) | 5.2(8) | -0.2(9) |
| C16 | 19.3(11) | 28.8(12) | 24.2(11) | 2.2(9) | 9.8(9) | 4.1(9) |
| C17 | 21.8(11) | 19.1(10) | 20.7(11) | -0.6(8) | 6.3(9) | 0.3(8) |
| C18 | 22.7(11) | 21.8(11) | 20.4(11) | 2.8(8) | 7.0(9) | -0.8(9) |
| C19 | 23.8(11) | 21.7(11) | 19.8(11) | -2.9(8) | 7.3(9) | 0.1(9) |
| C20 | 25.2(11) | 20.4(11) | 21.4(11) | 3.5(8) | 6.0(9) | 3.0(9) |
| C21 | 25.0(11) | 25.3(11) | 20.2(11) | 3.2(9) | 7.6(9) | 0.7(9) |
| C22 | 17.0(10) | 24.0(11) | 22.2(11) | 0.9(9) | 8.0(8) | 1.9(9) |
| C23 | 21.8(11) | 28.8(12) | 20.2(11) | 1.9(9) | 8.3(9) | 1.3(9) |
| C24 | 21.5(11) | 26.2(12) | 25.7(11) | 5.0(9) | 6.9(9) | 4.6(9) |
| C25 | 18.2(10) | 25.2(11) | 23.9(11) | 2.9(9) | 8.4(9) | 2.9(9) |
| C26 | 19.4(10) | 19.7(10) | 18.7(10) | -1.8(8) | 5.8(8) | -1.6(8) |
| C27 | 19.9(11) | 30.4(12) | 22.5(11) | -0.1(9) | 9.7(9) | 3.7(9) |
| C28 | 21.6(11) | 27.4(12) | 28.3(12) | 3.9(9) | 9.8(9) | 5.1(9) |
| C29 | 24.9(11) | 19.5(11) | 23.2(11) | -1.2(9) | 7.2(9) | 1.6(9) |
| C30 | 26.3(12) | 24.9(12) | 25.4(12) | 2.4(9) | 6.7(9) | 6.8(9) |
| C31 | 25.8(12) | 32.6(13) | 25.1(12) | -2.5(10) | 11.3(9) | 5.9(10) |
| C32 | 23.9(11) | 27.7(12) | 28.8(12) | -1.3(10) | 9.7(9) | 6.1(10) |
| C33 | 31.5(13) | 36.3(14) | 23.3(12) | -3.3(10) | 6.7(10) | 1.4(11) |
| C34 | 27.5(12) | 23.6(12) | 33.1(13) | 1.4(10) | 11.8(10) | 8.2(9) |
| C35 | 28.6(12) | 37.3(14) | 21.0(11) | -3.9(10) | 8.7(9) | 3.8(10) |
| C36 | 26.5(12) | 28.8(12) | 27.9(12) | 1.2(10) | 8.5(10) | 10.2(10) |
| C37 | 28.6(12) | 27.6(12) | 23.9(11) | 7.9(9) | 10.2(9) | 3.6(10) |
| C38 | 25.8(12) | 26.3(12) | 24.3(11) | 5.3(9) | 6.3(9) | 6.1(9) |
| C39 | 29.4(12) | 26.7(12) | 20.5(11) | 2.2(9) | 7.0(9) | 2.0(10) |
| C40 | 27.4(12) | 33.8(13) | 17.7(11) | 0.7(9) | 6.2(9) | 5.1(10) |
| C41 | 25.0(11) | 29.7(12) | 19.5(11) | 3.0(9) | 8.3(9) | 0.5(9) |
| C42 | 25.4(11) | 34.4(13) | 18.3(11) | 1.9(9) | 8.9(9) | 2.1(10) |
| C43 | 28.1(12) | 25.7(12) | 26.6(12) | -0.9(9) | 11.6(10) | 7.0(10) |
| C44 | 29.8(12) | 25.0(12) | 28.6(12) | 5.3(10) | 11.7(10) | 7.8(10) |
| C45 | 26.5(12) | 33.3(13) | 25.1(12) | 1.7(10) | 10.5(9) | 8.7(10) |
| C46 | 32.0(13) | 33.9(13) | 34.5(14) | 9.4(11) | 12.5(11) | 12.0(11) |
| C47 | 28.2(12) | 31.4(13) | 21.4(11) | 4.1(9) | 2.5(9) | 4.2(10) |
| C48 | 41.6(15) | 44.1(15) | 26.8(13) | 0.7(11) | 15.4(11) | 21.4(12) |
| C49 | 30.5(13) | 40.7(14) | 19.5(11) | 2.2(10) | 5.6(9) | 3.0(11) |
| C50 | 23.4(12) | 57.0(18) | 36.8(14) | 12.7(13) | 10.7(11) | 5.4(12) |
| C51 | 35.1(14) | 48.2(17) | 34.9(14) | 4.9(12) | 10.3(12) | -1.4(12) |
| C52 | 40.4(15) | 23.3(12) | 43.5(15) | 2.5(11) | 20.7(12) | 3.5(11) |
| C53 | 68(2) | 31.5(14) | 30.5(14) | 0.6(11) | 24.6(13) | 9.4(13) |
| C54 | 38.5(15) | 59.5(19) | 28.9(14) | 12.4(13) | 2.5(11) | -6.1(14) |
| C55 | 37.3(15) | 39.1(15) | 46.8(16) | -2.0(12) | 14.6(13) | -5.2(12) |
| C56 | 68(2) | 47.0(18) | 30.3(14) | -9.0(13) | -3.3(14) | 24.6(16) |
| C57 | 44.6(17) | 53.5(18) | 41.2(16) | 19.0(14) | 15.4(13) | 21.6(14) |
| C58 | 112(3) | 37.3(17) | 37.8(17) | -3.5(13) | 13.8(19) | 3.8(19) |
| C59 | 46.6(18) | 39.1(17) | 96(3) | -11.8(18) | 27.8(19) | -7.4(14) |
| C60 | 44.1(18) | 76(2) | 52.2(19) | 31.2(18) | 6.2(15) | -10.8(17) |
| C61 | 54.5(19) | 70(2) | 43.0(17) | -7.8(16) | 12.2(15) | -29.9(17) |
| C62 | 56(2) | 49.5(19) | 64(2) | 5.2(16) | 19.9(17) | -10.1(16) |
| C63 | 50(2) | 99(3) | 45.1(19) | 29.7(19) | 8.4(15) | 23(2) |
| C64 | 50(2) | 88(3) | 113(4) | -33(3) | 21(2) | -29(2) |
| C65 | 56(2) | 78(3) | 78(3) | -1(2) | 3(2) | -22(2) |
| C66 | 51(2) | 135(4) | 88(3) | 61(3) | -4(2) | -35(3) |
| C67 | 86(3) | 143(5) | 63(3) | 24(3) | -12(2) | -72(3) |
| C68 | 74(3) | 177(6) | 54(2) | 57(3) | 13(2) | 45(3) |
| C69 | 60(4) | 61(4) | 77(4) | -12(3) | 7(3) | -24(3) |
| Se4 | 27.50(14) | 32.06(14) | 30.61(14) | -6.12(11) | -0.92(10) | 0.33(11) |
| Se5 | 37.04(15) | 41.75(16) | 26.41(14) | -8.25(11) | 18.02(11) | -2.16(12) |
| Se6 | 37.00(15) | 37.41(16) | 26.77(14) | 5.81(11) | 8.32(11) | -1.57(12) |
| C70 | 19.0(10) | 26.1(11) | 19.3(10) | -0.4(9) | 4.2(8) | 8.2(9) |
| C71 | 16.7(10) | 27.9(12) | 18.8(10) | -2.3(9) | 3.7(8) | 7.0(9) |
| C72 | 16.1(10) | 28.2(12) | 20.1(11) | -1.3(9) | 4.5(8) | 7.5(9) |
| C73 | 20.1(11) | 25.9(11) | 21.3(11) | -0.1(9) | 2.7(9) | 9.0(9) |
| C74 | 20.1(11) | 31.2(12) | 20.5(11) | -1.6(9) | 4.9(9) | 3.7(9) |
| C75 | 20.3(10) | 27.7(12) | 19.6(11) | -2.0(9) | 3.8(8) | 10.6(9) |
| C76 | 18.0(10) | 30.4(12) | 18.7(10) | -1.4(9) | 4.5(8) | 8.9(9) |
| C77 | 18.7(10) | 30.4(12) | 18.1(10) | -1.7(9) | 4.6(8) | 4.5(9) |
| C78 | 22.8(11) | 32.6(13) | 18.3(11) | 0.7(9) | 4.2(9) | 5.6(10) |
| C79 | 25.2(12) | 24.5(12) | 27.2(12) | 1.5(9) | 4.6(9) | 6.1(9) |
| C80 | 20.4(11) | 34.9(13) | 21.7(11) | -0.9(9) | 7.6(9) | 3.5(9) |
| C81 | 23.2(11) | 22.8(11) | 24.5(11) | -0.1(9) | 4.4(9) | 6.9(9) |
| C82 | 21.5(11) | 26.5(12) | 19.7(11) | 1.2(9) | 4.3(9) | 5.4(9) |
| C83 | 26.8(12) | 23.4(11) | 27.5(12) | -4.4(9) | 3.0(9) | 7.7(9) |
| C84 | 24.7(12) | 33.3(13) | 25.4(12) | -6.2(10) | 7.5(9) | 0.2(10) |
| C85 | 21.2(11) | 32.8(12) | 18.8(11) | -1.4(9) | 7.0(9) | 8.5(9) |
| C86 | 31.6(13) | 33.2(13) | 21.1(11) | -7.1(10) | 2.2(10) | 7.9(10) |
| C87 | 23.9(11) | 26.7(12) | 21.6(11) | -4.6(9) | 3.2(9) | 9.2(9) |
| C88 | 17.5(10) | 32.3(12) | 20.1(11) | -3.0(9) | 6.4(8) | 3.3(9) |
| C89 | 26.9(12) | 26.1(12) | 31.1(13) | -3.1(10) | 1.6(10) | 2.3(10) |
| C90 | 24.5(11) | 29.0(12) | 22.2(11) | 4.1(9) | 4.7(9) | 6.2(10) |
| C91 | 29.2(12) | 42.6(15) | 24.4(12) | 2.5(10) | 11.7(10) | 2.3(11) |
| C92 | 27.6(12) | 25.6(12) | 23.4(11) | -4.8(9) | 2.3(9) | 7.9(10) |
| C93 | 32.0(13) | 34.9(14) | 27.4(12) | -11.2(10) | 13.0(10) | -1.3(11) |
| C94 | 30.2(13) | 35.2(13) | 22.6(12) | -6.6(10) | -0.6(10) | 6.6(11) |
| C95 | 25.0(11) | 32.2(13) | 19.7(11) | -4.6(9) | 3.7(9) | 9.1(10) |
| C96 | 23.0(11) | 33.8(13) | 27.0(12) | -5.7(10) | 7.6(9) | -3.4(10) |
| C97 | 19.3(11) | 31.4(12) | 21.1(11) | -4.1(9) | 5.2(9) | 2.5(9) |
| C98 | 28.1(12) | 32.7(13) | 22.2(11) | -1.3(10) | 8.6(9) | 3.6(10) |
| C99 | 20.4(11) | 34.7(13) | 20.6(11) | -0.3(9) | 7.8(9) | 5.5(10) |
| C100 | 30.7(13) | 31.2(13) | 28.0(12) | -7.7(10) | 10.2(10) | -2.9(10) |
| C101 | 25.3(11) | 33.8(13) | 18.8(11) | -3.1(9) | 7.0(9) | 9.0(10) |
| C102 | 27.7(12) | 28.3(12) | 29.9(13) | 2.9(10) | 7.0(10) | 0.8(10) |
| C103 | 25.9(12) | 33.5(13) | 27.3(12) | 4.0(10) | 7.9(10) | 1.9(10) |
| C104 | 18.4(10) | 32.3(12) | 22.3(11) | -4.1(9) | 5.4(9) | 1.6(9) |
| C105 | 28.4(13) | 29.3(13) | 35.4(14) | 0.2(10) | 4.1(10) | -2.6(10) |
| C106 | 27.0(12) | 34.9(13) | 25.5(12) | 1.1(10) | 8.9(10) | -1.1(10) |
| C107 | 27.5(12) | 37.8(14) | 22.7(11) | 2.1(10) | 9.8(9) | 3.1(10) |
| C108 | 28.8(12) | 42.5(15) | 21.2(11) | 5.3(10) | 6.6(10) | 6.0(11) |
| C109 | 28.8(12) | 28.4(12) | 29.3(12) | -9.2(10) | 0.7(10) | 4.7(10) |
| C110 | 31.0(13) | 30.0(13) | 32.3(13) | -1.0(10) | 9.3(10) | -5.2(10) |
| C111 | 31.2(13) | 42.5(15) | 18.4(11) | -3.0(10) | 3.2(10) | 5.9(11) |
| C112 | 36.3(14) | 43.9(15) | 20.8(12) | 1.8(11) | -0.1(10) | 6.2(12) |
| C113 | 27.6(12) | 41.2(14) | 20.9(11) | -6.0(10) | 10.0(9) | 2.7(11) |
| C114 | 27.4(13) | 36.4(14) | 30.9(13) | 1.4(11) | 9.2(10) | -1.7(11) |
| C115 | 58.8(18) | 37.1(15) | 25.9(13) | 3.0(11) | 5.6(12) | -10.3(13) |
| C116 | 40.0(14) | 32.7(14) | 28.3(13) | 2.0(10) | 4.9(11) | -5.0(11) |
| C117 | 26.6(13) | 48.2(16) | 38.7(15) | -1.0(12) | 9.6(11) | -0.1(12) |
| C118 | 27.1(12) | 42.5(14) | 18.1(11) | -2.1(10) | 5.6(9) | 5.4(11) |
| C119 | 36.0(14) | 46.3(16) | 32.1(14) | 3.3(12) | 3.2(11) | 12.2(12) |
| C120 | 35.9(14) | 31.1(13) | 38.8(14) | -7.3(11) | 11.8(11) | -0.5(11) |
| C121 | 35.1(14) | 43.9(16) | 33.2(14) | 3.6(12) | 10.5(11) | -8.1(12) |
| C122 | 33.2(14) | 52.2(17) | 34.6(14) | -11.2(12) | 11.0(11) | -11.8(13) |
| C123 | 37.8(14) | 57.3(18) | 19.5(12) | -7.5(12) | 3.0(10) | 7.5(13) |
| C124 | 36.2(14) | 36.7(14) | 36.6(14) | -9.9(11) | 9.1(11) | -6.6(12) |
| C125 | 33.8(15) | 44.1(17) | 56.6(19) | -9.2(14) | 4.9(13) | 2.3(13) |
| C126 | 35.0(15) | 39.8(16) | 57.8(19) | -4.1(14) | 4.1(13) | 2.2(13) |
| C127 | 45.2(16) | 53.3(18) | 28.3(14) | -11.8(12) | 6.0(12) | 9.7(14) |
| C128 | 80(2) | 33.9(16) | 37.6(16) | -1.0(12) | 2.0(15) | 5.1(15) |
| C129 | 50.7(19) | 51.5(19) | 57(2) | 0.0(16) | 8.3(15) | 2.7(15) |
| C130 | 50.1(19) | 97(3) | 37.0(17) | -28.2(18) | 4.1(14) | 19.9(19) |
| C131 | 55(2) | 62(2) | 44.2(18) | -4.4(15) | 11.6(15) | 7.0(16) |
| C132 | 52(2) | 67(2) | 60(2) | -22.2(18) | 15.7(16) | 12.8(17) |
| C133 | 49.0(19) | 67(2) | 55(2) | 1.6(17) | -1.0(16) | 23.7(17) |
| C134 | 53(2) | 72(3) | 90(3) | -1(2) | 14(2) | 29(2) |
| C135 | 60(2) | 57(2) | 84(3) | 0(2) | 8(2) | 10.8(18) |
| C136 | 47(2) | 69(3) | 106(3) | -11(2) | 5(2) | 12.5(19) |
| C137 | 73(3) | 108(4) | 46(2) | 5(2) | 13.6(19) | 17(2) |
| C138 | 76(3) | 75(3) | 128(5) | -15(3) | 11(3) | 6(3) |
| Cl1 | 143(5) | 103(4) | 71(2) | -1(2) | 10(3) | -8(3) |
| Cl2 | 64.3(5) | 59.3(5) | 74.7(6) | -15.9(4) | 7.8(5) | -1.3(4) |
| Cl3 | 60.4(16) | 78.2(19) | 43.8(15) | -8.9(13) | 9.8(13) | 2.4(13) |
| C139 | 28(3) | 47(4) | 60(4) | -3(3) | 7(3) | 18(3) |
| Cl4 | 52.9(13) | 78.3(18) | 74.7(17) | -5.3(12) | 30.2(11) | 6.3(10) |
| Cl5 | 70(2) | 60.3(15) | 61.1(17) | 15.9(10) | 11.9(14) | 2.8(11) |
| Cl6 | 51.5(18) | 66(2) | 45.0(15) | -6.0(14) | 5.8(11) | -21.4(15) |
| C140 | 45(4) | 69(5) | 70(4) | 8(3) | 16(3) | -7(4) |
| C141 | 54(6) | 50(7) | 111(10) | -9(6) | -24(6) | 7(5) |
| Cl7 | 38.2(13) | 50.9(14) | 73.3(19) | 11.2(11) | 28.3(11) | 0.7(9) |
| Cl8 | 137(5) | 116(4) | 125(4) | -74(3) | 91(3) | -62(3) |
| C142 | 60(6) | 64(5) | 78(6) | -12(4) | 25(5) | -2(5) |
| Cl9 | 168(7) | 103(5) | 78(3) | 3(3) | 17(4) | 35(5) |
| Cl10 | 85(4) | 63(3) | 56(4) | 1(3) | 3(2) | -2(3) |
| C143 | 35(5) | 55(5) | 60(5) | 1(4) | 12(4) | -16(4) |
| Cl11 | 71(4) | 179(7) | 63(2) | -3(4) | 5(2) | 51(4) |

Table 4 Bond Lengths for 1b.

| Atom | Atom | Length/Å |  | Atom | Atom | Length/Å |
| --- | --- | --- | --- | --- | --- | --- |
| Se1 | C30 | 1.919(2) |  | C70 | C71 | 1.419(3) |
| Se1 | C38 | 1.922(2) |  | C70 | C73 | 1.423(3) |
| Se2 | C13 | 1.924(2) |  | C70 | C82 | 1.451(3) |
| Se2 | C41 | 1.921(2) |  | C71 | C72 | 1.423(3) |
| Se3 | C31 | 1.926(3) |  | C71 | C77 | 1.447(3) |
| Se3 | C32 | 1.923(2) |  | C72 | C76 | 1.419(3) |
| C1 | C4 | 1.418(3) |  | C72 | C88 | 1.442(3) |
| C1 | C6 | 1.446(3) |  | C73 | C75 | 1.424(3) |
| C1 | C8 | 1.424(3) |  | C73 | C81 | 1.442(3) |
| C2 | C3 | 1.420(3) |  | C74 | C77 | 1.405(3) |
| C2 | C5 | 1.423(3) |  | C74 | C78 | 1.415(3) |
| C2 | C18 | 1.448(3) |  | C74 | C113 | 1.401(3) |
| C3 | C8 | 1.421(3) |  | C75 | C76 | 1.422(3) |
| C3 | C12 | 1.448(3) |  | C75 | C87 | 1.449(3) |
| C4 | C5 | 1.423(3) |  | C76 | C85 | 1.448(3) |
| C4 | C26 | 1.451(3) |  | C77 | C97 | 1.386(3) |
| C5 | C17 | 1.444(3) |  | C78 | C82 | 1.406(3) |
| C6 | C10 | 1.384(3) |  | C78 | C98 | 1.405(3) |
| C6 | C15 | 1.408(3) |  | C79 | C81 | 1.389(3) |
| C7 | C17 | 1.406(3) |  | C79 | C90 | 1.412(3) |
| C7 | C20 | 1.416(3) |  | C79 | C102 | 1.398(3) |
| C7 | C30 | 1.394(3) |  | C80 | C88 | 1.407(3) |
| C8 | C22 | 1.445(3) |  | C80 | C99 | 1.419(3) |
| C9 | C13 | 1.398(3) |  | C80 | C106 | 1.386(4) |
| C9 | C15 | 1.418(3) |  | C81 | C83 | 1.411(3) |
| C9 | C26 | 1.406(3) |  | C82 | C90 | 1.385(3) |
| C10 | C24 | 1.402(3) |  | C83 | C89 | 1.388(4) |
| C10 | C25 | 1.415(3) |  | C83 | C92 | 1.417(4) |
| C11 | C14 | 1.393(3) |  | C84 | C93 | 1.399(3) |
| C11 | C19 | 1.406(3) |  | C84 | C97 | 1.401(3) |
| C11 | C45 | 1.552(3) |  | C84 | C100 | 1.560(4) |
| C12 | C23 | 1.390(3) |  | C85 | C99 | 1.403(4) |
| C12 | C27 | 1.408(3) |  | C85 | C101 | 1.388(3) |
| C13 | C14 | 1.417(3) |  | C86 | C94 | 1.388(4) |
| C15 | C41 | 1.397(3) |  | C86 | C95 | 1.403(3) |
| C16 | C22 | 1.404(3) |  | C86 | C111 | 1.560(4) |
| C16 | C27 | 1.417(3) |  | C87 | C92 | 1.402(4) |
| C16 | C32 | 1.396(3) |  | C87 | C95 | 1.386(3) |
| C17 | C29 | 1.391(3) |  | C88 | C104 | 1.391(3) |
| C18 | C20 | 1.401(3) |  | C89 | C105 | 1.420(4) |
| C18 | C21 | 1.392(3) |  | C90 | C103 | 1.402(3) |
| C19 | C26 | 1.387(3) |  | C91 | C98 | 1.410(4) |
| C19 | C29 | 1.412(3) |  | C91 | C103 | 1.405(4) |
| C20 | C38 | 1.401(3) |  | C92 | C109 | 1.406(3) |
| C21 | C23 | 1.411(3) |  | C93 | C113 | 1.411(4) |
| C21 | C39 | 1.401(3) |  | C94 | C109 | 1.415(4) |
| C22 | C25 | 1.388(3) |  | C95 | C101 | 1.407(4) |
| C23 | C42 | 1.398(3) |  | C96 | C100 | 1.563(3) |
| C24 | C37 | 1.399(3) |  | C96 | C104 | 1.397(4) |
| C24 | C44 | 1.557(3) |  | C96 | C110 | 1.402(4) |
| C25 | C28 | 1.400(3) |  | C97 | C104 | 1.410(3) |
| C27 | C31 | 1.399(3) |  | C99 | C107 | 1.402(3) |
| C28 | C34 | 1.397(3) |  | C100 | C120 | 1.539(4) |
| C28 | C44 | 1.565(3) |  | C100 | C122 | 1.545(4) |
| C29 | C43 | 1.397(3) |  | C101 | C118 | 1.404(3) |
| C30 | C36 | 1.423(3) |  | C102 | C105 | 1.395(4) |
| C31 | C35 | 1.422(3) |  | C102 | C114 | 1.561(3) |
| C32 | C34 | 1.423(3) |  | C103 | C114 | 1.559(4) |
| C33 | C40 | 1.538(4) |  | C106 | C110 | 1.423(4) |
| C33 | C51 | 1.528(4) |  | C107 | C108 | 1.421(4) |
| C35 | C42 | 1.390(4) |  | C108 | C118 | 1.390(4) |
| C36 | C43 | 1.386(3) |  | C111 | C112 | 1.535(4) |
| C37 | C41 | 1.416(3) |  | C111 | C118 | 1.562(4) |
| C38 | C47 | 1.418(3) |  | C111 | C123 | 1.555(3) |
| C39 | C40 | 1.559(3) |  | C112 | C119 | 1.523(4) |
| C39 | C47 | 1.392(3) |  | C114 | C117 | 1.534(4) |
| C40 | C42 | 1.562(3) |  | C114 | C121 | 1.551(4) |
| C40 | C49 | 1.547(3) |  | C115 | C116 | 1.527(4) |
| C43 | C45 | 1.562(3) |  | C115 | C128 | 1.517(5) |
| C44 | C46 | 1.554(3) |  | C116 | C121 | 1.517(4) |
| C44 | C52 | 1.536(4) |  | C117 | C126 | 1.514(4) |
| C45 | C48 | 1.548(3) |  | C119 | C133 | 1.502(4) |
| C45 | C50 | 1.542(4) |  | C120 | C125 | 1.536(4) |
| C46 | C57 | 1.531(4) |  | C122 | C124 | 1.540(4) |
| C48 | C53 | 1.517(4) |  | C123 | C127 | 1.522(4) |
| C49 | C54 | 1.513(4) |  | C124 | C131 | 1.492(4) |
| C50 | C61 | 1.504(5) |  | C125 | C136 | 1.519(5) |
| C51 | C62 | 1.520(4) |  | C126 | C129 | 1.535(4) |
| C52 | C55 | 1.523(4) |  | C127 | C130 | 1.515(4) |
| C53 | C56 | 1.520(4) |  | C129 | C135 | 1.503(5) |
| C54 | C60 | 1.510(4) |  | C130 | C132 | 1.512(5) |
| C55 | C59 | 1.514(4) |  | C131 | C137 | 1.526(5) |
| C56 | C58 | 1.522(5) |  | C133 | C134 | 1.508(5) |
| C57 | C63 | 1.509(5) |  | C136 | C138 | 1.396(7) |
| C59 | C64 | 1.525(6) |  | Cl1 | C139 | 1.767(10) |
| C60 | C66 | 1.499(5) |  | Cl2 | C139 | 1.746(6) |
| C61 | C67 | 1.524(5) |  | Cl2 | C142 | 1.744(8) |
| C62 | C65 | 1.490(5) |  | Cl3 | C139 | 1.654(8) |
| C63 | C68 | 1.533(5) |  | Cl4 | C140 | 1.635(8) |
| C67 | C69 | 1.348(8) |  | Cl5 | C140 | 1.672(7) |
| C67 | C141 | 1.380(11) |  | Cl6 | C140 | 1.771(10) |
| Se4 | C89 | 1.931(2) |  | Cl7 | C142 | 1.703(8) |
| Se4 | C109 | 1.930(3) |  | Cl8 | C142 | 1.666(10) |
| Se5 | C98 | 1.928(2) |  | Cl9 | C143 | 1.716(11) |
| Se5 | C113 | 1.930(2) |  | Cl10 | C143 | 1.634(13) |
| Se6 | C106 | 1.927(2) |  | C143 | Cl11 | 1.620(10) |
| Se6 | C107 | 1.927(3) |  |  |  |  |

Table 5 Bond Angles for 1b.

| Atom | Atom | Atom | Angle/˚ |  | Atom | Atom | Atom | Angle/˚ |
| --- | --- | --- | --- | --- | --- | --- | --- | --- |
| C30 | Se1 | C38 | 88.82(10) |  | C70 | C71 | C72 | 120.1(2) |
| C41 | Se2 | C13 | 89.04(10) |  | C70 | C71 | C77 | 118.7(2) |
| C32 | Se3 | C31 | 89.01(10) |  | C72 | C71 | C77 | 119.3(2) |
| C4 | C1 | C6 | 118.97(19) |  | C71 | C72 | C88 | 119.5(2) |
| C4 | C1 | C8 | 119.86(19) |  | C76 | C72 | C71 | 119.9(2) |
| C8 | C1 | C6 | 119.22(19) |  | C76 | C72 | C88 | 118.7(2) |
| C3 | C2 | C5 | 120.1(2) |  | C70 | C73 | C75 | 119.7(2) |
| C3 | C2 | C18 | 119.2(2) |  | C70 | C73 | C81 | 119.4(2) |
| C5 | C2 | C18 | 118.4(2) |  | C75 | C73 | C81 | 118.8(2) |
| C2 | C3 | C8 | 119.93(19) |  | C77 | C74 | C78 | 119.8(2) |
| C2 | C3 | C12 | 119.5(2) |  | C113 | C74 | C77 | 120.6(2) |
| C8 | C3 | C12 | 118.6(2) |  | C113 | C74 | C78 | 117.6(2) |
| C1 | C4 | C5 | 120.22(19) |  | C73 | C75 | C87 | 118.4(2) |
| C1 | C4 | C26 | 118.60(19) |  | C76 | C75 | C73 | 120.0(2) |
| C5 | C4 | C26 | 119.04(19) |  | C76 | C75 | C87 | 119.3(2) |
| C2 | C5 | C4 | 119.75(19) |  | C72 | C76 | C75 | 120.1(2) |
| C2 | C5 | C17 | 118.7(2) |  | C72 | C76 | C85 | 118.4(2) |
| C4 | C5 | C17 | 119.51(19) |  | C75 | C76 | C85 | 119.5(2) |
| C10 | C6 | C1 | 119.90(19) |  | C74 | C77 | C71 | 121.5(2) |
| C10 | C6 | C15 | 115.8(2) |  | C97 | C77 | C71 | 119.4(2) |
| C15 | C6 | C1 | 121.4(2) |  | C97 | C77 | C74 | 116.4(2) |
| C17 | C7 | C20 | 119.4(2) |  | C82 | C78 | C74 | 119.8(2) |
| C30 | C7 | C17 | 121.1(2) |  | C98 | C78 | C74 | 117.5(2) |
| C30 | C7 | C20 | 117.1(2) |  | C98 | C78 | C82 | 120.7(2) |
| C1 | C8 | C22 | 119.29(19) |  | C81 | C79 | C90 | 121.1(2) |
| C3 | C8 | C1 | 120.1(2) |  | C81 | C79 | C102 | 125.1(2) |
| C3 | C8 | C22 | 118.6(2) |  | C102 | C79 | C90 | 111.1(2) |
| C13 | C9 | C15 | 117.3(2) |  | C88 | C80 | C99 | 119.1(2) |
| C13 | C9 | C26 | 120.8(2) |  | C106 | C80 | C88 | 121.3(2) |
| C26 | C9 | C15 | 119.70(19) |  | C106 | C80 | C99 | 117.6(2) |
| C6 | C10 | C24 | 125.2(2) |  | C79 | C81 | C73 | 119.8(2) |
| C6 | C10 | C25 | 121.0(2) |  | C79 | C81 | C83 | 115.7(2) |
| C24 | C10 | C25 | 110.8(2) |  | C83 | C81 | C73 | 121.7(2) |
| C14 | C11 | C19 | 118.7(2) |  | C78 | C82 | C70 | 121.3(2) |
| C14 | C11 | C45 | 132.5(2) |  | C90 | C82 | C70 | 119.8(2) |
| C19 | C11 | C45 | 107.6(2) |  | C90 | C82 | C78 | 116.1(2) |
| C23 | C12 | C3 | 119.9(2) |  | C81 | C83 | C92 | 119.2(2) |
| C23 | C12 | C27 | 115.5(2) |  | C89 | C83 | C81 | 120.8(2) |
| C27 | C12 | C3 | 121.6(2) |  | C89 | C83 | C92 | 117.7(2) |
| C9 | C13 | Se2 | 107.78(16) |  | C93 | C84 | C97 | 118.2(2) |
| C9 | C13 | C14 | 122.0(2) |  | C93 | C84 | C100 | 132.5(2) |
| C14 | C13 | Se2 | 129.19(17) |  | C97 | C84 | C100 | 107.8(2) |
| C11 | C14 | C13 | 117.7(2) |  | C99 | C85 | C76 | 121.7(2) |
| C6 | C15 | C9 | 119.7(2) |  | C101 | C85 | C76 | 119.6(2) |
| C41 | C15 | C6 | 120.8(2) |  | C101 | C85 | C99 | 116.1(2) |
| C41 | C15 | C9 | 117.1(2) |  | C94 | C86 | C95 | 118.8(2) |
| C22 | C16 | C27 | 120.0(2) |  | C94 | C86 | C111 | 132.7(2) |
| C32 | C16 | C22 | 121.0(2) |  | C95 | C86 | C111 | 107.4(2) |
| C32 | C16 | C27 | 117.1(2) |  | C92 | C87 | C75 | 121.6(2) |
| C7 | C17 | C5 | 121.7(2) |  | C95 | C87 | C75 | 119.2(2) |
| C29 | C17 | C5 | 119.8(2) |  | C95 | C87 | C92 | 116.3(2) |
| C29 | C17 | C7 | 115.8(2) |  | C80 | C88 | C72 | 122.1(2) |
| C20 | C18 | C2 | 121.6(2) |  | C104 | C88 | C72 | 119.6(2) |
| C21 | C18 | C2 | 119.6(2) |  | C104 | C88 | C80 | 115.6(2) |
| C21 | C18 | C20 | 115.9(2) |  | C83 | C89 | Se4 | 108.08(18) |
| C11 | C19 | C29 | 111.0(2) |  | C83 | C89 | C105 | 122.0(2) |
| C26 | C19 | C11 | 124.8(2) |  | C105 | C89 | Se4 | 128.65(19) |
| C26 | C19 | C29 | 121.0(2) |  | C82 | C90 | C79 | 120.8(2) |
| C18 | C20 | C7 | 119.9(2) |  | C82 | C90 | C103 | 125.2(2) |
| C18 | C20 | C38 | 121.0(2) |  | C103 | C90 | C79 | 111.2(2) |
| C38 | C20 | C7 | 117.0(2) |  | C103 | C91 | C98 | 118.3(2) |
| C18 | C21 | C23 | 121.2(2) |  | C87 | C92 | C83 | 120.1(2) |
| C18 | C21 | C39 | 124.8(2) |  | C87 | C92 | C109 | 120.5(2) |
| C39 | C21 | C23 | 111.0(2) |  | C109 | C92 | C83 | 117.2(2) |
| C16 | C22 | C8 | 121.6(2) |  | C84 | C93 | C113 | 118.5(2) |
| C25 | C22 | C8 | 119.8(2) |  | C86 | C94 | C109 | 118.1(2) |
| C25 | C22 | C16 | 115.9(2) |  | C86 | C95 | C101 | 110.9(2) |
| C12 | C23 | C21 | 120.6(2) |  | C87 | C95 | C86 | 124.5(2) |
| C12 | C23 | C42 | 124.9(2) |  | C87 | C95 | C101 | 121.6(2) |
| C42 | C23 | C21 | 111.2(2) |  | C104 | C96 | C100 | 107.5(2) |
| C10 | C24 | C44 | 107.78(19) |  | C104 | C96 | C110 | 118.8(2) |
| C37 | C24 | C10 | 118.4(2) |  | C110 | C96 | C100 | 132.4(2) |
| C37 | C24 | C44 | 132.7(2) |  | C77 | C97 | C84 | 124.7(2) |
| C22 | C25 | C10 | 120.8(2) |  | C77 | C97 | C104 | 121.3(2) |
| C22 | C25 | C28 | 124.9(2) |  | C84 | C97 | C104 | 110.9(2) |
| C28 | C25 | C10 | 111.4(2) |  | C78 | C98 | Se5 | 107.38(18) |
| C9 | C26 | C4 | 121.5(2) |  | C78 | C98 | C91 | 121.7(2) |
| C19 | C26 | C4 | 119.8(2) |  | C91 | C98 | Se5 | 130.04(18) |
| C19 | C26 | C9 | 115.9(2) |  | C85 | C99 | C80 | 119.8(2) |
| C12 | C27 | C16 | 119.3(2) |  | C107 | C99 | C80 | 117.0(2) |
| C31 | C27 | C12 | 121.2(2) |  | C107 | C99 | C85 | 121.2(2) |
| C31 | C27 | C16 | 117.4(2) |  | C84 | C100 | C96 | 101.77(19) |
| C25 | C28 | C44 | 107.2(2) |  | C120 | C100 | C84 | 110.6(2) |
| C34 | C28 | C25 | 118.8(2) |  | C120 | C100 | C96 | 110.4(2) |
| C34 | C28 | C44 | 133.0(2) |  | C120 | C100 | C122 | 110.5(2) |
| C17 | C29 | C19 | 120.8(2) |  | C122 | C100 | C84 | 113.2(2) |
| C17 | C29 | C43 | 124.7(2) |  | C122 | C100 | C96 | 110.0(2) |
| C43 | C29 | C19 | 111.1(2) |  | C85 | C101 | C95 | 120.8(2) |
| C7 | C30 | Se1 | 108.47(17) |  | C85 | C101 | C118 | 124.6(2) |
| C7 | C30 | C36 | 121.4(2) |  | C118 | C101 | C95 | 111.6(2) |
| C36 | C30 | Se1 | 128.86(18) |  | C79 | C102 | C114 | 107.6(2) |
| C27 | C31 | Se3 | 107.88(17) |  | C105 | C102 | C79 | 118.4(2) |
| C27 | C31 | C35 | 121.4(2) |  | C105 | C102 | C114 | 132.9(2) |
| C35 | C31 | Se3 | 129.40(19) |  | C90 | C103 | C91 | 118.1(2) |
| C16 | C32 | Se3 | 108.24(17) |  | C90 | C103 | C114 | 107.4(2) |
| C16 | C32 | C34 | 121.9(2) |  | C91 | C103 | C114 | 133.4(2) |
| C34 | C32 | Se3 | 129.02(19) |  | C88 | C104 | C96 | 124.9(2) |
| C51 | C33 | C40 | 115.5(2) |  | C88 | C104 | C97 | 120.7(2) |
| C28 | C34 | C32 | 117.5(2) |  | C96 | C104 | C97 | 111.6(2) |
| C42 | C35 | C31 | 117.8(2) |  | C102 | C105 | C89 | 117.9(2) |
| C43 | C36 | C30 | 118.0(2) |  | C80 | C106 | Se6 | 108.08(18) |
| C24 | C37 | C41 | 117.8(2) |  | C80 | C106 | C110 | 121.9(2) |
| C20 | C38 | Se1 | 108.19(17) |  | C110 | C106 | Se6 | 129.0(2) |
| C20 | C38 | C47 | 121.6(2) |  | C99 | C107 | Se6 | 107.81(18) |
| C47 | C38 | Se1 | 128.95(18) |  | C99 | C107 | C108 | 121.1(2) |
| C21 | C39 | C40 | 107.6(2) |  | C108 | C107 | Se6 | 130.08(19) |
| C47 | C39 | C21 | 118.8(2) |  | C118 | C108 | C107 | 118.2(2) |
| C47 | C39 | C40 | 132.3(2) |  | C92 | C109 | Se4 | 107.71(19) |
| C33 | C40 | C39 | 111.74(19) |  | C92 | C109 | C94 | 121.6(2) |
| C33 | C40 | C42 | 111.3(2) |  | C94 | C109 | Se4 | 129.62(19) |
| C33 | C40 | C49 | 108.76(19) |  | C96 | C110 | C106 | 117.4(2) |
| C39 | C40 | C42 | 101.68(18) |  | C86 | C111 | C118 | 101.81(19) |
| C49 | C40 | C39 | 111.2(2) |  | C112 | C111 | C86 | 112.3(2) |
| C49 | C40 | C42 | 112.05(19) |  | C112 | C111 | C118 | 111.8(2) |
| C15 | C41 | Se2 | 108.06(16) |  | C112 | C111 | C123 | 109.2(2) |
| C15 | C41 | C37 | 121.9(2) |  | C123 | C111 | C86 | 110.6(2) |
| C37 | C41 | Se2 | 128.75(17) |  | C123 | C111 | C118 | 111.1(2) |
| C23 | C42 | C40 | 107.5(2) |  | C119 | C112 | C111 | 116.3(2) |
| C35 | C42 | C23 | 119.1(2) |  | C74 | C113 | Se5 | 107.46(18) |
| C35 | C42 | C40 | 132.1(2) |  | C74 | C113 | C93 | 121.5(2) |
| C29 | C43 | C45 | 107.7(2) |  | C93 | C113 | Se5 | 129.68(18) |
| C36 | C43 | C29 | 119.0(2) |  | C103 | C114 | C102 | 101.82(19) |
| C36 | C43 | C45 | 131.8(2) |  | C117 | C114 | C102 | 111.4(2) |
| C24 | C44 | C28 | 101.73(18) |  | C117 | C114 | C103 | 112.6(2) |
| C46 | C44 | C24 | 110.3(2) |  | C117 | C114 | C121 | 108.5(2) |
| C46 | C44 | C28 | 108.92(19) |  | C121 | C114 | C102 | 110.3(2) |
| C52 | C44 | C24 | 111.56(19) |  | C121 | C114 | C103 | 112.1(2) |
| C52 | C44 | C28 | 112.6(2) |  | C128 | C115 | C116 | 113.0(3) |
| C52 | C44 | C46 | 111.3(2) |  | C121 | C116 | C115 | 112.5(2) |
| C11 | C45 | C43 | 101.91(18) |  | C126 | C117 | C114 | 116.6(2) |
| C48 | C45 | C11 | 112.0(2) |  | C101 | C118 | C111 | 107.0(2) |
| C48 | C45 | C43 | 111.8(2) |  | C108 | C118 | C101 | 118.8(2) |
| C50 | C45 | C11 | 112.2(2) |  | C108 | C118 | C111 | 133.0(2) |
| C50 | C45 | C43 | 110.7(2) |  | C133 | C119 | C112 | 113.0(2) |
| C50 | C45 | C48 | 108.2(2) |  | C125 | C120 | C100 | 114.6(2) |
| C57 | C46 | C44 | 115.1(2) |  | C116 | C121 | C114 | 116.1(2) |
| C39 | C47 | C38 | 117.9(2) |  | C124 | C122 | C100 | 115.2(2) |
| C53 | C48 | C45 | 115.9(2) |  | C127 | C123 | C111 | 116.6(2) |
| C54 | C49 | C40 | 116.5(2) |  | C131 | C124 | C122 | 112.0(3) |
| C61 | C50 | C45 | 116.5(2) |  | C136 | C125 | C120 | 113.9(3) |
| C62 | C51 | C33 | 113.3(2) |  | C117 | C126 | C129 | 110.5(3) |
| C55 | C52 | C44 | 115.6(2) |  | C130 | C127 | C123 | 112.5(2) |
| C48 | C53 | C56 | 112.3(3) |  | C135 | C129 | C126 | 112.0(3) |
| C60 | C54 | C49 | 112.7(2) |  | C132 | C130 | C127 | 113.9(3) |
| C59 | C55 | C52 | 112.6(3) |  | C124 | C131 | C137 | 113.7(3) |
| C53 | C56 | C58 | 112.3(3) |  | C119 | C133 | C134 | 114.0(3) |
| C63 | C57 | C46 | 112.9(3) |  | C138 | C136 | C125 | 116.3(4) |
| C55 | C59 | C64 | 111.9(3) |  | Cl2 | C139 | Cl1 | 113.0(5) |
| C66 | C60 | C54 | 115.2(3) |  | Cl3 | C139 | Cl1 | 114.9(5) |
| C50 | C61 | C67 | 112.5(4) |  | Cl3 | C139 | Cl2 | 115.1(4) |
| C65 | C62 | C51 | 115.3(3) |  | Cl4 | C140 | Cl5 | 121.0(5) |
| C57 | C63 | C68 | 112.6(3) |  | Cl4 | C140 | Cl6 | 117.8(5) |
| C69 | C67 | C61 | 120.9(5) |  | Cl5 | C140 | Cl6 | 114.2(5) |
| C141 | C67 | C61 | 112.6(6) |  | Cl7 | C142 | Cl2 | 112.3(5) |
| C109 | Se4 | C89 | 88.98(11) |  | Cl8 | C142 | Cl2 | 117.1(5) |
| C98 | Se5 | C113 | 89.33(10) |  | Cl8 | C142 | Cl7 | 118.3(5) |
| C106 | Se6 | C107 | 88.92(11) |  | Cl10 | C143 | Cl9 | 115.1(7) |
| C71 | C70 | C73 | 120.1(2) |  | Cl11 | C143 | Cl9 | 115.2(6) |
| C71 | C70 | C82 | 118.8(2) |  | Cl11 | C143 | Cl10 | 123.7(8) |
| C73 | C70 | C82 | 119.2(2) |  |  |  |  |  |

Table 6 Torsion Angles for 1b.

| A | B | C | D | Angle/˚ |  | A | B | C | D | Angle/˚ |
| --- | --- | --- | --- | --- | --- | --- | --- | --- | --- | --- |
| Se1 | C30 | C36 | C43 | -166.16(19) |  | Se4 | C89 | C105 | C102 | 164.22(19) |
| Se1 | C38 | C47 | C39 | 164.60(19) |  | Se6 | C106 | C110 | C96 | -166.85(19) |
| Se2 | C13 | C14 | C11 | 166.55(18) |  | Se6 | C107 | C108 | C118 | 166.45(19) |
| Se3 | C31 | C35 | C42 | -164.14(19) |  | C70 | C71 | C72 | C76 | 0.6(3) |
| Se3 | C32 | C34 | C28 | 166.64(18) |  | C70 | C71 | C72 | C88 | -163.2(2) |
| C1 | C4 | C5 | C2 | 0.8(3) |  | C70 | C71 | C77 | C74 | 2.7(3) |
| C1 | C4 | C5 | C17 | -162.8(2) |  | C70 | C71 | C77 | C97 | 163.7(2) |
| C1 | C4 | C26 | C9 | 2.6(3) |  | C70 | C73 | C75 | C76 | 0.9(3) |
| C1 | C4 | C26 | C19 | 162.7(2) |  | C70 | C73 | C75 | C87 | 163.7(2) |
| C1 | C6 | C10 | C24 | 158.4(2) |  | C70 | C73 | C81 | C79 | 0.3(3) |
| C1 | C6 | C10 | C25 | 0.1(3) |  | C70 | C73 | C81 | C83 | -159.9(2) |
| C1 | C6 | C15 | C9 | 2.6(3) |  | C70 | C82 | C90 | C79 | -0.3(3) |
| C1 | C6 | C15 | C41 | -159.5(2) |  | C70 | C82 | C90 | C103 | 158.9(2) |
| C1 | C8 | C22 | C16 | -160.0(2) |  | C71 | C70 | C73 | C75 | -0.5(3) |
| C1 | C8 | C22 | C25 | 0.6(3) |  | C71 | C70 | C73 | C81 | 162.8(2) |
| C2 | C3 | C8 | C1 | 0.6(3) |  | C71 | C70 | C82 | C78 | -2.5(3) |
| C2 | C3 | C8 | C22 | -163.3(2) |  | C71 | C70 | C82 | C90 | -163.0(2) |
| C2 | C3 | C12 | C23 | -0.2(3) |  | C71 | C72 | C76 | C75 | -0.2(3) |
| C2 | C3 | C12 | C27 | 159.4(2) |  | C71 | C72 | C76 | C85 | -164.2(2) |
| C2 | C5 | C17 | C7 | -3.5(3) |  | C71 | C72 | C88 | C80 | 160.5(2) |
| C2 | C5 | C17 | C29 | -163.8(2) |  | C71 | C72 | C88 | C104 | -0.2(3) |
| C2 | C18 | C20 | C7 | -3.8(3) |  | C71 | C77 | C97 | C84 | -158.8(2) |
| C2 | C18 | C20 | C38 | 159.2(2) |  | C71 | C77 | C97 | C104 | -0.4(3) |
| C2 | C18 | C21 | C23 | -0.3(3) |  | C72 | C71 | C77 | C74 | -161.7(2) |
| C2 | C18 | C21 | C39 | -158.9(2) |  | C72 | C71 | C77 | C97 | -0.7(3) |
| C3 | C2 | C5 | C4 | -1.0(3) |  | C72 | C76 | C85 | C99 | 3.8(3) |
| C3 | C2 | C5 | C17 | 162.7(2) |  | C72 | C76 | C85 | C101 | 164.7(2) |
| C3 | C2 | C18 | C20 | -159.2(2) |  | C72 | C88 | C104 | C96 | 158.5(2) |
| C3 | C2 | C18 | C21 | 0.9(3) |  | C72 | C88 | C104 | C97 | -0.9(3) |
| C3 | C8 | C22 | C16 | 4.0(3) |  | C73 | C70 | C71 | C72 | -0.2(3) |
| C3 | C8 | C22 | C25 | 164.6(2) |  | C73 | C70 | C71 | C77 | -164.5(2) |
| C3 | C12 | C23 | C21 | 0.8(3) |  | C73 | C70 | C82 | C78 | 161.8(2) |
| C3 | C12 | C23 | C42 | 158.6(2) |  | C73 | C70 | C82 | C90 | 1.2(3) |
| C3 | C12 | C27 | C16 | 3.9(3) |  | C73 | C75 | C76 | C72 | -0.5(3) |
| C3 | C12 | C27 | C31 | -159.2(2) |  | C73 | C75 | C76 | C85 | 163.3(2) |
| C4 | C1 | C6 | C10 | -163.4(2) |  | C73 | C75 | C87 | C92 | -4.0(3) |
| C4 | C1 | C6 | C15 | -3.5(3) |  | C73 | C75 | C87 | C95 | -164.2(2) |
| C4 | C1 | C8 | C3 | -0.8(3) |  | C73 | C81 | C83 | C89 | 158.9(2) |
| C4 | C1 | C8 | C22 | 162.9(2) |  | C73 | C81 | C83 | C92 | -3.6(3) |
| C4 | C5 | C17 | C7 | 160.3(2) |  | C74 | C77 | C97 | C84 | 3.1(3) |
| C4 | C5 | C17 | C29 | -0.1(3) |  | C74 | C77 | C97 | C104 | 161.5(2) |
| C5 | C2 | C3 | C8 | 0.4(3) |  | C74 | C78 | C82 | C70 | 3.1(3) |
| C5 | C2 | C3 | C12 | -163.5(2) |  | C74 | C78 | C82 | C90 | 164.3(2) |
| C5 | C2 | C18 | C20 | 3.9(3) |  | C74 | C78 | C98 | Se5 | 6.6(3) |
| C5 | C2 | C18 | C21 | 164.0(2) |  | C74 | C78 | C98 | C91 | -163.4(2) |
| C5 | C4 | C26 | C9 | -160.8(2) |  | C75 | C73 | C81 | C79 | 163.8(2) |
| C5 | C4 | C26 | C19 | -0.7(3) |  | C75 | C73 | C81 | C83 | 3.5(3) |
| C5 | C17 | C29 | C19 | 0.2(3) |  | C75 | C76 | C85 | C99 | -160.3(2) |
| C5 | C17 | C29 | C43 | 157.4(2) |  | C75 | C76 | C85 | C101 | 0.5(3) |
| C6 | C1 | C4 | C5 | 164.14(19) |  | C75 | C87 | C92 | C83 | 4.0(3) |
| C6 | C1 | C4 | C26 | 0.9(3) |  | C75 | C87 | C92 | C109 | -158.6(2) |
| C6 | C1 | C8 | C3 | -164.8(2) |  | C75 | C87 | C95 | C86 | 159.2(2) |
| C6 | C1 | C8 | C22 | -1.0(3) |  | C75 | C87 | C95 | C101 | 0.7(3) |
| C6 | C10 | C24 | C37 | 2.8(4) |  | C76 | C72 | C88 | C80 | -3.6(3) |
| C6 | C10 | C24 | C44 | -166.7(2) |  | C76 | C72 | C88 | C104 | -164.2(2) |
| C6 | C10 | C25 | C22 | -0.6(3) |  | C76 | C75 | C87 | C92 | 158.9(2) |
| C6 | C10 | C25 | C28 | 160.9(2) |  | C76 | C75 | C87 | C95 | -1.3(3) |
| C6 | C15 | C41 | Se2 | 168.14(17) |  | C76 | C85 | C99 | C80 | -3.5(3) |
| C6 | C15 | C41 | C37 | 0.0(3) |  | C76 | C85 | C99 | C107 | 160.0(2) |
| C7 | C17 | C29 | C19 | -161.3(2) |  | C76 | C85 | C101 | C95 | -1.2(3) |
| C7 | C17 | C29 | C43 | -4.1(3) |  | C76 | C85 | C101 | C118 | -159.9(2) |
| C7 | C20 | C38 | Se1 | -3.8(3) |  | C77 | C71 | C72 | C76 | 164.8(2) |
| C7 | C20 | C38 | C47 | 164.6(2) |  | C77 | C71 | C72 | C88 | 1.0(3) |
| C7 | C30 | C36 | C43 | -0.5(4) |  | C77 | C74 | C78 | C82 | -0.8(3) |
| C8 | C1 | C4 | C5 | 0.2(3) |  | C77 | C74 | C78 | C98 | 163.1(2) |
| C8 | C1 | C4 | C26 | -163.07(19) |  | C77 | C74 | C113 | Se5 | -169.29(17) |
| C8 | C1 | C6 | C10 | 0.7(3) |  | C77 | C74 | C113 | C93 | -1.3(4) |
| C8 | C1 | C6 | C15 | 160.5(2) |  | C77 | C97 | C104 | C88 | 1.2(3) |
| C8 | C3 | C12 | C23 | -164.3(2) |  | C77 | C97 | C104 | C96 | -160.7(2) |
| C8 | C3 | C12 | C27 | -4.7(3) |  | C78 | C74 | C77 | C71 | -2.2(3) |
| C8 | C22 | C25 | C10 | 0.3(3) |  | C78 | C74 | C77 | C97 | -163.7(2) |
| C8 | C22 | C25 | C28 | -158.6(2) |  | C78 | C74 | C113 | Se5 | -5.7(3) |
| C9 | C13 | C14 | C11 | -0.5(3) |  | C78 | C74 | C113 | C93 | 162.3(2) |
| C9 | C15 | C41 | Se2 | 5.5(3) |  | C78 | C82 | C90 | C79 | -161.9(2) |
| C9 | C15 | C41 | C37 | -162.6(2) |  | C78 | C82 | C90 | C103 | -2.7(3) |
| C10 | C6 | C15 | C9 | 163.3(2) |  | C79 | C81 | C83 | C89 | -2.1(3) |
| C10 | C6 | C15 | C41 | 1.1(3) |  | C79 | C81 | C83 | C92 | -164.6(2) |
| C10 | C24 | C37 | C41 | -1.4(3) |  | C79 | C90 | C103 | C91 | 163.8(2) |
| C10 | C24 | C44 | C28 | 9.9(2) |  | C79 | C90 | C103 | C114 | -5.7(3) |
| C10 | C24 | C44 | C46 | -105.5(2) |  | C79 | C102 | C105 | C89 | 1.5(4) |
| C10 | C24 | C44 | C52 | 130.2(2) |  | C79 | C102 | C114 | C103 | -9.0(3) |
| C10 | C25 | C28 | C34 | -163.3(2) |  | C79 | C102 | C114 | C117 | -129.2(2) |
| C10 | C25 | C28 | C44 | 6.8(3) |  | C79 | C102 | C114 | C121 | 110.2(2) |
| C11 | C19 | C26 | C4 | -157.3(2) |  | C80 | C88 | C104 | C96 | -3.4(3) |
| C11 | C19 | C26 | C9 | 3.9(3) |  | C80 | C88 | C104 | C97 | -162.7(2) |
| C11 | C19 | C29 | C17 | 160.3(2) |  | C80 | C99 | C107 | Se6 | -4.8(3) |
| C11 | C19 | C29 | C43 | 0.2(3) |  | C80 | C99 | C107 | C108 | 164.9(2) |
| C11 | C45 | C48 | C53 | -57.1(3) |  | C80 | C106 | C110 | C96 | 0.1(4) |
| C11 | C45 | C50 | C61 | 55.7(3) |  | C81 | C73 | C75 | C76 | -162.5(2) |
| C12 | C3 | C8 | C1 | 164.6(2) |  | C81 | C73 | C75 | C87 | 0.3(3) |
| C12 | C3 | C8 | C22 | 0.7(3) |  | C81 | C79 | C90 | C82 | -0.6(3) |
| C12 | C23 | C42 | C35 | 2.6(4) |  | C81 | C79 | C90 | C103 | -162.5(2) |
| C12 | C23 | C42 | C40 | -165.7(2) |  | C81 | C79 | C102 | C105 | -2.3(4) |
| C12 | C27 | C31 | Se3 | 167.27(17) |  | C81 | C79 | C102 | C114 | 167.5(2) |
| C12 | C27 | C31 | C35 | -0.8(4) |  | C81 | C83 | C89 | Se4 | -166.50(18) |
| C13 | C9 | C15 | C6 | -162.3(2) |  | C81 | C83 | C89 | C105 | 1.7(4) |
| C13 | C9 | C15 | C41 | 0.5(3) |  | C81 | C83 | C92 | C87 | -0.2(3) |
| C13 | C9 | C26 | C4 | 159.1(2) |  | C81 | C83 | C92 | C109 | 163.0(2) |
| C13 | C9 | C26 | C19 | -1.7(3) |  | C82 | C70 | C71 | C72 | 163.9(2) |
| C14 | C11 | C19 | C26 | -4.3(4) |  | C82 | C70 | C71 | C77 | -0.4(3) |
| C14 | C11 | C19 | C29 | -164.2(2) |  | C82 | C70 | C73 | C75 | -164.6(2) |
| C14 | C11 | C45 | C43 | 159.6(2) |  | C82 | C70 | C73 | C81 | -1.2(3) |
| C14 | C11 | C45 | C48 | -80.8(3) |  | C82 | C78 | C98 | Se5 | 170.31(18) |
| C14 | C11 | C45 | C50 | 41.1(4) |  | C82 | C78 | C98 | C91 | 0.2(4) |
| C15 | C6 | C10 | C24 | -2.6(3) |  | C82 | C90 | C103 | C91 | 2.9(4) |
| C15 | C6 | C10 | C25 | -160.8(2) |  | C82 | C90 | C103 | C114 | -166.6(2) |
| C15 | C9 | C13 | Se2 | -6.2(2) |  | C83 | C89 | C105 | C102 | -1.3(4) |
| C15 | C9 | C13 | C14 | 163.3(2) |  | C83 | C92 | C109 | Se4 | 3.8(3) |
| C15 | C9 | C26 | C4 | -3.6(3) |  | C83 | C92 | C109 | C94 | -165.4(2) |
| C15 | C9 | C26 | C19 | -164.4(2) |  | C84 | C93 | C113 | Se5 | 165.6(2) |
| C16 | C22 | C25 | C10 | 161.9(2) |  | C84 | C93 | C113 | C74 | 0.6(4) |
| C16 | C22 | C25 | C28 | 3.0(3) |  | C84 | C97 | C104 | C88 | 162.3(2) |
| C16 | C27 | C31 | Se3 | 3.9(3) |  | C84 | C97 | C104 | C96 | 0.4(3) |
| C16 | C27 | C31 | C35 | -164.2(2) |  | C84 | C100 | C120 | C125 | 55.8(3) |
| C16 | C32 | C34 | C28 | -1.3(4) |  | C84 | C100 | C122 | C124 | 57.7(3) |
| C17 | C7 | C20 | C18 | -0.1(3) |  | C85 | C99 | C107 | Se6 | -168.79(17) |
| C17 | C7 | C20 | C38 | -163.7(2) |  | C85 | C99 | C107 | C108 | 0.9(3) |
| C17 | C7 | C30 | Se1 | 167.57(18) |  | C85 | C101 | C118 | C108 | -1.9(4) |
| C17 | C7 | C30 | C36 | -0.7(4) |  | C85 | C101 | C118 | C111 | 167.2(2) |
| C17 | C29 | C43 | C36 | 3.0(4) |  | C86 | C94 | C109 | Se4 | -164.56(19) |
| C17 | C29 | C43 | C45 | -164.6(2) |  | C86 | C94 | C109 | C92 | 1.9(4) |
| C18 | C2 | C3 | C8 | 163.2(2) |  | C86 | C95 | C101 | C85 | -160.6(2) |
| C18 | C2 | C3 | C12 | -0.6(3) |  | C86 | C95 | C101 | C118 | 0.6(3) |
| C18 | C2 | C5 | C4 | -164.0(2) |  | C86 | C111 | C112 | C119 | -57.4(3) |
| C18 | C2 | C5 | C17 | -0.3(3) |  | C86 | C111 | C118 | C101 | -10.7(2) |
| C18 | C20 | C38 | Se1 | -167.18(18) |  | C86 | C111 | C118 | C108 | 156.3(3) |
| C18 | C20 | C38 | C47 | 1.2(4) |  | C86 | C111 | C123 | C127 | 53.5(3) |
| C18 | C21 | C23 | C12 | -0.6(3) |  | C87 | C75 | C76 | C72 | -163.2(2) |
| C18 | C21 | C23 | C42 | -161.2(2) |  | C87 | C75 | C76 | C85 | 0.7(3) |
| C18 | C21 | C39 | C40 | 166.5(2) |  | C87 | C92 | C109 | Se4 | 166.90(18) |
| C18 | C21 | C39 | C47 | -2.3(4) |  | C87 | C92 | C109 | C94 | -2.2(4) |
| C19 | C11 | C14 | C13 | 2.4(3) |  | C87 | C95 | C101 | C85 | 0.6(3) |
| C19 | C11 | C45 | C43 | -7.7(2) |  | C87 | C95 | C101 | C118 | 161.8(2) |
| C19 | C11 | C45 | C48 | 112.0(2) |  | C88 | C72 | C76 | C75 | 163.7(2) |
| C19 | C11 | C45 | C50 | -126.2(2) |  | C88 | C72 | C76 | C85 | -0.3(3) |
| C19 | C29 | C43 | C36 | 162.2(2) |  | C88 | C80 | C99 | C85 | -0.3(3) |
| C19 | C29 | C43 | C45 | -5.4(3) |  | C88 | C80 | C99 | C107 | -164.5(2) |
| C20 | C7 | C17 | C5 | 3.7(3) |  | C88 | C80 | C106 | Se6 | 169.13(18) |
| C20 | C7 | C17 | C29 | 164.8(2) |  | C88 | C80 | C106 | C110 | -0.2(4) |
| C20 | C7 | C30 | Se1 | 5.2(3) |  | C89 | C83 | C92 | C87 | -163.2(2) |
| C20 | C7 | C30 | C36 | -163.1(2) |  | C89 | C83 | C92 | C109 | 0.0(3) |
| C20 | C18 | C21 | C23 | 160.9(2) |  | C90 | C79 | C81 | C73 | 0.6(3) |
| C20 | C18 | C21 | C39 | 2.3(3) |  | C90 | C79 | C81 | C83 | 162.0(2) |
| C20 | C38 | C47 | C39 | -1.1(4) |  | C90 | C79 | C102 | C105 | -163.6(2) |
| C21 | C18 | C20 | C7 | -164.6(2) |  | C90 | C79 | C102 | C114 | 6.2(3) |
| C21 | C18 | C20 | C38 | -1.6(3) |  | C90 | C103 | C114 | C102 | 8.7(3) |
| C21 | C23 | C42 | C35 | 162.2(2) |  | C90 | C103 | C114 | C117 | 128.1(2) |
| C21 | C23 | C42 | C40 | -6.2(3) |  | C90 | C103 | C114 | C121 | -109.1(2) |
| C21 | C39 | C40 | C33 | -127.8(2) |  | C91 | C103 | C114 | C102 | -158.5(3) |
| C21 | C39 | C40 | C42 | -8.9(2) |  | C91 | C103 | C114 | C117 | -39.1(4) |
| C21 | C39 | C40 | C49 | 110.5(2) |  | C91 | C103 | C114 | C121 | 83.7(3) |
| C21 | C39 | C47 | C38 | 1.6(4) |  | C92 | C83 | C89 | Se4 | -3.7(3) |
| C22 | C16 | C27 | C12 | 0.8(3) |  | C92 | C83 | C89 | C105 | 164.4(2) |
| C22 | C16 | C27 | C31 | 164.5(2) |  | C92 | C87 | C95 | C86 | -2.0(3) |
| C22 | C16 | C32 | Se3 | -168.39(17) |  | C92 | C87 | C95 | C101 | -160.5(2) |
| C22 | C16 | C32 | C34 | 1.8(4) |  | C93 | C84 | C97 | C77 | -3.9(4) |
| C22 | C25 | C28 | C34 | -2.7(4) |  | C93 | C84 | C97 | C104 | -164.2(2) |
| C22 | C25 | C28 | C44 | 167.4(2) |  | C93 | C84 | C100 | C96 | 159.8(3) |
| C23 | C12 | C27 | C16 | 164.4(2) |  | C93 | C84 | C100 | C120 | 42.5(4) |
| C23 | C12 | C27 | C31 | 1.2(3) |  | C93 | C84 | C100 | C122 | -82.2(3) |
| C23 | C21 | C39 | C40 | 5.9(3) |  | C94 | C86 | C95 | C87 | 1.8(4) |
| C23 | C21 | C39 | C47 | -162.8(2) |  | C94 | C86 | C95 | C101 | 162.3(2) |
| C24 | C10 | C25 | C22 | -161.7(2) |  | C94 | C86 | C111 | C112 | -37.4(4) |
| C24 | C10 | C25 | C28 | -0.2(3) |  | C94 | C86 | C111 | C118 | -157.1(3) |
| C24 | C37 | C41 | Se2 | -165.30(18) |  | C94 | C86 | C111 | C123 | 84.8(3) |
| C24 | C37 | C41 | C15 | 0.2(4) |  | C95 | C86 | C94 | C109 | -1.7(3) |
| C24 | C44 | C46 | C57 | -67.1(3) |  | C95 | C86 | C111 | C112 | 130.8(2) |
| C24 | C44 | C52 | C55 | -63.0(3) |  | C95 | C86 | C111 | C118 | 11.1(2) |
| C25 | C10 | C24 | C37 | 162.9(2) |  | C95 | C86 | C111 | C123 | -107.0(2) |
| C25 | C10 | C24 | C44 | -6.6(3) |  | C95 | C87 | C92 | C83 | 164.8(2) |
| C25 | C28 | C34 | C32 | 1.6(3) |  | C95 | C87 | C92 | C109 | 2.1(3) |
| C25 | C28 | C44 | C24 | -10.0(2) |  | C95 | C101 | C118 | C108 | -162.3(2) |
| C25 | C28 | C44 | C46 | 106.5(2) |  | C95 | C101 | C118 | C111 | 6.9(3) |
| C25 | C28 | C44 | C52 | -129.5(2) |  | C96 | C100 | C120 | C125 | -56.1(3) |
| C26 | C4 | C5 | C2 | 163.9(2) |  | C96 | C100 | C122 | C124 | 170.8(2) |
| C26 | C4 | C5 | C17 | 0.4(3) |  | C97 | C84 | C93 | C113 | 1.9(4) |
| C26 | C9 | C13 | Se2 | -169.31(17) |  | C97 | C84 | C100 | C96 | -5.6(2) |
| C26 | C9 | C13 | C14 | 0.2(3) |  | C97 | C84 | C100 | C120 | -122.9(2) |
| C26 | C9 | C15 | C6 | 1.0(3) |  | C97 | C84 | C100 | C122 | 112.4(2) |
| C26 | C9 | C15 | C41 | 163.8(2) |  | C98 | C78 | C82 | C70 | -160.2(2) |
| C26 | C19 | C29 | C17 | -0.5(3) |  | C98 | C78 | C82 | C90 | 1.0(3) |
| C26 | C19 | C29 | C43 | -160.6(2) |  | C98 | C91 | C103 | C90 | -1.4(4) |
| C27 | C12 | C23 | C21 | -160.0(2) |  | C98 | C91 | C103 | C114 | 164.8(3) |
| C27 | C12 | C23 | C42 | -2.2(3) |  | C99 | C80 | C88 | C72 | 3.9(3) |
| C27 | C16 | C22 | C8 | -4.8(3) |  | C99 | C80 | C88 | C104 | 165.3(2) |
| C27 | C16 | C22 | C25 | -166.1(2) |  | C99 | C80 | C106 | Se6 | 5.4(3) |
| C27 | C16 | C32 | Se3 | -4.3(3) |  | C99 | C80 | C106 | C110 | -164.0(2) |
| C27 | C16 | C32 | C34 | 165.8(2) |  | C99 | C85 | C101 | C95 | 160.7(2) |
| C27 | C31 | C35 | C42 | 1.1(4) |  | C99 | C85 | C101 | C118 | 2.1(3) |
| C28 | C44 | C46 | C57 | -178.0(2) |  | C99 | C107 | C108 | C118 | -0.7(4) |
| C28 | C44 | C52 | C55 | 50.6(3) |  | C100 | C84 | C93 | C113 | -162.3(3) |
| C29 | C19 | C26 | C4 | 0.8(3) |  | C100 | C84 | C97 | C77 | 163.9(2) |
| C29 | C19 | C26 | C9 | 161.9(2) |  | C100 | C84 | C97 | C104 | 3.6(3) |
| C29 | C43 | C45 | C11 | 7.9(3) |  | C100 | C96 | C104 | C88 | -165.2(2) |
| C29 | C43 | C45 | C48 | -112.0(2) |  | C100 | C96 | C104 | C97 | -4.2(3) |
| C29 | C43 | C45 | C50 | 127.3(2) |  | C100 | C96 | C110 | C106 | 163.6(2) |
| C30 | C7 | C17 | C5 | -158.3(2) |  | C100 | C120 | C125 | C136 | -170.6(3) |
| C30 | C7 | C17 | C29 | 2.8(3) |  | C100 | C122 | C124 | C131 | -102.5(3) |
| C30 | C7 | C20 | C18 | 162.6(2) |  | C101 | C85 | C99 | C80 | -165.0(2) |
| C30 | C7 | C20 | C38 | -1.0(3) |  | C101 | C85 | C99 | C107 | -1.5(3) |
| C30 | C36 | C43 | C29 | -0.5(4) |  | C102 | C79 | C81 | C73 | -158.9(2) |
| C30 | C36 | C43 | C45 | 163.5(2) |  | C102 | C79 | C81 | C83 | 2.5(4) |
| C31 | C35 | C42 | C23 | -1.9(3) |  | C102 | C79 | C90 | C82 | 161.5(2) |
| C31 | C35 | C42 | C40 | 163.1(2) |  | C102 | C79 | C90 | C103 | -0.3(3) |
| C32 | C16 | C22 | C8 | 158.8(2) |  | C102 | C114 | C117 | C126 | 61.0(3) |
| C32 | C16 | C22 | C25 | -2.5(3) |  | C102 | C114 | C121 | C116 | -60.1(3) |
| C32 | C16 | C27 | C12 | -163.4(2) |  | C103 | C91 | C98 | Se5 | -167.66(19) |
| C32 | C16 | C27 | C31 | 0.3(3) |  | C103 | C91 | C98 | C78 | -0.1(4) |
| C33 | C40 | C42 | C23 | 128.2(2) |  | C103 | C114 | C117 | C126 | -52.7(3) |
| C33 | C40 | C42 | C35 | -38.1(3) |  | C103 | C114 | C121 | C116 | 52.6(3) |
| C33 | C40 | C49 | C54 | 171.5(2) |  | C104 | C96 | C100 | C84 | 5.8(2) |
| C33 | C51 | C62 | C65 | 56.3(4) |  | C104 | C96 | C100 | C120 | 123.3(2) |
| C34 | C28 | C44 | C24 | 158.1(3) |  | C104 | C96 | C100 | C122 | -114.4(2) |
| C34 | C28 | C44 | C46 | -85.4(3) |  | C104 | C96 | C110 | C106 | -1.5(4) |
| C34 | C28 | C44 | C52 | 38.6(4) |  | C105 | C102 | C114 | C103 | 158.7(3) |
| C36 | C43 | C45 | C11 | -157.5(3) |  | C105 | C102 | C114 | C117 | 38.4(4) |
| C36 | C43 | C45 | C48 | 82.7(3) |  | C105 | C102 | C114 | C121 | -82.1(3) |
| C36 | C43 | C45 | C50 | -38.0(4) |  | C106 | C80 | C88 | C72 | -159.6(2) |
| C37 | C24 | C44 | C28 | -157.5(3) |  | C106 | C80 | C88 | C104 | 1.7(3) |
| C37 | C24 | C44 | C46 | 87.0(3) |  | C106 | C80 | C99 | C85 | 163.8(2) |
| C37 | C24 | C44 | C52 | -37.2(4) |  | C106 | C80 | C99 | C107 | -0.4(3) |
| C39 | C21 | C23 | C12 | 160.7(2) |  | C107 | C108 | C118 | C101 | 1.2(3) |
| C39 | C21 | C23 | C42 | 0.2(3) |  | C107 | C108 | C118 | C111 | -164.6(2) |
| C39 | C40 | C42 | C23 | 9.0(2) |  | C110 | C96 | C100 | C84 | -160.5(3) |
| C39 | C40 | C42 | C35 | -157.2(2) |  | C110 | C96 | C100 | C120 | -43.1(4) |
| C39 | C40 | C49 | C54 | -65.1(3) |  | C110 | C96 | C100 | C122 | 79.2(3) |
| C40 | C33 | C51 | C62 | 168.2(2) |  | C110 | C96 | C104 | C88 | 3.4(4) |
| C40 | C39 | C47 | C38 | -163.9(2) |  | C110 | C96 | C104 | C97 | 164.3(2) |
| C40 | C49 | C54 | C60 | 172.5(3) |  | C111 | C86 | C94 | C109 | 165.5(2) |
| C42 | C40 | C49 | C54 | 47.9(3) |  | C111 | C86 | C95 | C87 | -168.3(2) |
| C43 | C45 | C48 | C53 | 56.6(3) |  | C111 | C86 | C95 | C101 | -7.8(3) |
| C43 | C45 | C50 | C61 | -57.4(3) |  | C111 | C112 | C119 | C133 | 179.1(3) |
| C44 | C24 | C37 | C41 | 164.9(2) |  | C111 | C123 | C127 | C130 | -177.9(3) |
| C44 | C28 | C34 | C32 | -165.4(2) |  | C112 | C111 | C118 | C101 | -130.7(2) |
| C44 | C46 | C57 | C63 | 167.7(3) |  | C112 | C111 | C118 | C108 | 36.2(4) |
| C44 | C52 | C55 | C59 | 172.9(3) |  | C112 | C111 | C123 | C127 | 177.5(3) |
| C45 | C11 | C14 | C13 | -163.8(2) |  | C112 | C119 | C133 | C134 | 177.0(3) |
| C45 | C11 | C19 | C26 | 165.0(2) |  | C113 | C74 | C77 | C71 | 161.1(2) |
| C45 | C11 | C19 | C29 | 5.1(3) |  | C113 | C74 | C77 | C97 | -0.5(3) |
| C45 | C48 | C53 | C56 | -176.4(2) |  | C113 | C74 | C78 | C82 | -164.5(2) |
| C45 | C50 | C61 | C67 | 169.6(3) |  | C113 | C74 | C78 | C98 | -0.7(3) |
| C46 | C44 | C52 | C55 | 173.3(2) |  | C114 | C102 | C105 | C89 | -165.1(3) |
| C46 | C57 | C63 | C68 | 176.8(4) |  | C114 | C117 | C126 | C129 | -170.7(3) |
| C47 | C39 | C40 | C33 | 38.9(4) |  | C115 | C116 | C121 | C114 | 172.3(2) |
| C47 | C39 | C40 | C42 | 157.7(3) |  | C117 | C114 | C121 | C116 | 177.6(2) |
| C47 | C39 | C40 | C49 | -82.8(3) |  | C117 | C126 | C129 | C135 | -172.9(3) |
| C48 | C45 | C50 | C61 | 179.7(2) |  | C118 | C111 | C112 | C119 | 56.3(3) |
| C48 | C53 | C56 | C58 | -176.9(2) |  | C118 | C111 | C123 | C127 | -58.8(3) |
| C49 | C40 | C42 | C23 | -109.8(2) |  | C120 | C100 | C122 | C124 | -67.0(3) |
| C49 | C40 | C42 | C35 | 84.0(3) |  | C120 | C125 | C136 | C138 | -59.8(5) |
| C49 | C54 | C60 | C66 | 171.1(4) |  | C121 | C114 | C117 | C126 | -177.4(2) |
| C50 | C45 | C48 | C53 | 178.7(2) |  | C122 | C100 | C120 | C125 | -178.0(2) |
| C50 | C61 | C67 | C69 | -166.4(5) |  | C122 | C124 | C131 | C137 | 172.2(3) |
| C50 | C61 | C67 | C141 | -101.1(7) |  | C123 | C111 | C112 | C119 | 179.6(3) |
| C51 | C33 | C40 | C39 | 53.2(3) |  | C123 | C111 | C118 | C101 | 107.1(2) |
| C51 | C33 | C40 | C42 | -59.7(3) |  | C123 | C111 | C118 | C108 | -85.9(3) |
| C51 | C33 | C40 | C49 | 176.3(2) |  | C123 | C127 | C130 | C132 | 176.8(3) |
| C52 | C44 | C46 | C57 | 57.3(3) |  | C128 | C115 | C116 | C121 | 173.7(2) |
| C52 | C55 | C59 | C64 | 174.3(3) |  |  |  |  |  |  |

Table 7 Hydrogen Atom Coordinates (Å×104) and Isotropic Displacement Parameters (Å2×103) for 1b.

| Atom | *x* | *y* | *z* | U(eq) |
| --- | --- | --- | --- | --- |
| H14 | 3986.16 | 8264.02 | 2751.96 | 30 |
| H33A | 3738.39 | 9205.22 | 6761.06 | 36 |
| H33B | 4380.2 | 8695.38 | 6780.46 | 36 |
| H34 | 662.54 | 10890.25 | 4525.68 | 33 |
| H35 | 2244.01 | 9568.17 | 6367.82 | 34 |
| H36 | 5515.68 | 7089.91 | 4461.95 | 33 |
| H37 | 1436.66 | 10226.59 | 2773.19 | 31 |
| H46A | -524.9 | 10319.67 | 3315.86 | 39 |
| H46B | -369.8 | 10947.03 | 3516.93 | 39 |
| H47 | 4583.31 | 7727.67 | 6330.1 | 33 |
| H48A | 4834.68 | 7105.41 | 2964.71 | 44 |
| H48B | 5252.97 | 6780.41 | 3430.58 | 44 |
| H49A | 3412.27 | 7970.36 | 6959.45 | 36 |
| H49B | 2881.03 | 8515.58 | 7024.54 | 36 |
| H50A | 5874.95 | 7647.17 | 3776.4 | 46 |
| H50B | 5478.61 | 7975.17 | 3308.91 | 46 |
| H51A | 4594.94 | 8919.3 | 6061.81 | 47 |
| H51B | 3830.9 | 9353.43 | 5961.77 | 47 |
| H52A | 1374.08 | 11094.59 | 3295.83 | 41 |
| H52B | 1094.29 | 11322.21 | 3741.69 | 41 |
| H53A | 3528.08 | 6729.12 | 2982.41 | 49 |
| H53B | 3914.41 | 6428.1 | 3466.17 | 49 |
| H54A | 1677.87 | 8118.14 | 6578.1 | 52 |
| H54B | 2190.22 | 7595.07 | 6443.5 | 52 |
| H55A | 2191.26 | 10846.78 | 4230.05 | 48 |
| H55B | 2438.69 | 10542.91 | 3800.35 | 48 |
| H56A | 4730.14 | 5834.19 | 3110.81 | 61 |
| H56B | 4393.02 | 6149.13 | 2627.66 | 61 |
| H57A | -1.38 | 11268.15 | 2858.15 | 55 |
| H57B | 54.99 | 10637.79 | 2671.57 | 55 |
| H58A | 3029.05 | 5805.57 | 2608.78 | 94 |
| H58B | 3385.06 | 5476.42 | 3083.25 | 94 |
| H58C | 3670.02 | 5293.5 | 2625.58 | 94 |
| H59A | 2547.34 | 11740.21 | 3940.65 | 71 |
| H59B | 2867.47 | 11405.58 | 3547.13 | 71 |
| H60A | 2463.27 | 7250.41 | 7189.69 | 70 |
| H60B | 2088.03 | 7806.09 | 7370.07 | 70 |
| H61A | 5201.4 | 8329.25 | 4172.13 | 67 |
| H61B | 4975.66 | 8679.61 | 3699.55 | 67 |
| H62A | 5106.62 | 9842.55 | 6141.5 | 67 |
| H62B | 4562 | 9987.32 | 6505.35 | 67 |
| H63A | -1467.42 | 11154.78 | 2751.94 | 78 |
| H63B | -1394.45 | 10538.91 | 2541.13 | 78 |
| H64A | 3878.82 | 10935.92 | 4141.65 | 125 |
| H64B | 3589.72 | 11325.17 | 4515.93 | 125 |
| H64C | 4023.73 | 11605.7 | 4141.97 | 125 |
| H65A | 5907.69 | 9214.24 | 6685.02 | 109 |
| H65B | 5390.14 | 9411 | 7050.64 | 109 |
| H65C | 5968.38 | 9854.73 | 6864.69 | 109 |
| H66A | 1105.61 | 7007.33 | 6769.6 | 141 |
| H66B | 751.8 | 7543.04 | 6992.17 | 141 |
| H66C | 1176.72 | 7031.69 | 7314.57 | 141 |
| H67C | 6578.17 | 8557.28 | 3801.28 | 123 |
| H67D | 6163.77 | 9111.98 | 3960.53 | 123 |
| H67A | 6652.11 | 8397.52 | 4117.12 | 123 |
| H67B | 6345.97 | 8851.65 | 3720.19 | 123 |
| H68A | -930.22 | 10955.51 | 1915.75 | 153 |
| H68B | -1014.22 | 11570.62 | 2125.49 | 153 |
| H68C | -1828.42 | 11195.64 | 1934.31 | 153 |
| H69A | 5987.36 | 9422.12 | 4233.57 | 101 |
| H69B | 6956.74 | 9265.91 | 4344.91 | 101 |
| H69C | 6357.68 | 8969.33 | 4627.43 | 101 |
| H91 | 8766.67 | 6834.57 | 3018.34 | 37 |
| H93 | 6546.89 | 4687.05 | 3075.76 | 37 |
| H94 | 9408.25 | 7298.29 | 6621.28 | 37 |
| H105 | 10174.08 | 8082.26 | 4730.98 | 38 |
| H108 | 7353.47 | 5305.32 | 6680.94 | 37 |
| H110 | 5936.11 | 3919.93 | 4851.84 | 37 |
| H11A | 9333.06 | 6349.42 | 7094.35 | 42 |
| H11B | 8764.97 | 5808.5 | 7116.28 | 42 |
| H11C | 8960.65 | 9336.41 | 3465.86 | 50 |
| H11D | 8800.79 | 9054.49 | 2964.5 | 50 |
| H11E | 8308.78 | 8574.81 | 3744.82 | 41 |
| H11F | 8042.02 | 8305.47 | 3238.83 | 41 |
| H11G | 10234.02 | 7293.22 | 3538.11 | 45 |
| H11H | 10583.07 | 7599.37 | 4021.67 | 45 |
| H11I | 8915.57 | 5519.83 | 6379.6 | 47 |
| H11J | 9474.01 | 6066.05 | 6350.91 | 47 |
| H12A | 6612.03 | 3597.23 | 4149.52 | 42 |
| H12B | 6777.76 | 3802.86 | 3667.47 | 42 |
| H12C | 9741.35 | 8434.76 | 3714.15 | 44 |
| H12D | 9418.31 | 8100.33 | 3240.9 | 44 |
| H12E | 5107.58 | 3692.14 | 3849.02 | 47 |
| H12F | 4693.38 | 4288.95 | 3680.02 | 47 |
| H12G | 7659.78 | 6413.83 | 7286.52 | 47 |
| H12H | 8258.37 | 6943.19 | 7284.06 | 47 |
| H12I | 5680.97 | 3864.37 | 3107.36 | 44 |
| H12J | 4887.83 | 3477.46 | 3114.36 | 44 |
| H12K | 7636.31 | 4559.13 | 4086.89 | 55 |
| H12L | 7546.92 | 4260.13 | 4554.62 | 55 |
| H12M | 9902.44 | 6486.45 | 3897.9 | 54 |
| H12N | 10099.26 | 6795.63 | 4390.51 | 54 |
| H12O | 7172.86 | 7406.47 | 6743.05 | 51 |
| H12P | 6573.24 | 6879.97 | 6764.68 | 51 |
| H12Q | 7365.55 | 9204.76 | 2852.12 | 79 |
| H12R | 7838.39 | 9793.93 | 2985.6 | 79 |
| H12S | 7492.63 | 9449.61 | 3364.94 | 79 |
| H12T | 11359.91 | 6606.64 | 3882.02 | 65 |
| H12U | 11527.73 | 6842.74 | 4399.43 | 65 |
| H13A | 6632.95 | 7065.26 | 7547.08 | 75 |
| H13B | 7272.33 | 7572.72 | 7544.5 | 75 |
| H13C | 4755.59 | 4653.03 | 2915.46 | 64 |
| H13D | 3994.12 | 4221.31 | 2865.46 | 64 |
| H13E | 6202.68 | 8069.65 | 7016.04 | 89 |
| H13F | 5965.66 | 7957.38 | 7500.11 | 89 |
| H13G | 5567.99 | 7572.74 | 7062.07 | 89 |
| H13H | 9942.84 | 5198.59 | 7024.31 | 71 |
| H13I | 10506.3 | 5732.9 | 6969.86 | 71 |
| H13J | 10580.89 | 5382.26 | 6226.66 | 108 |
| H13K | 10102.04 | 4825.56 | 6329.74 | 108 |
| H13L | 11008.22 | 4968.08 | 6638.98 | 108 |
| H13M | 10986.3 | 5725.01 | 4126.08 | 102 |
| H13N | 11097.22 | 5958.07 | 4641.49 | 102 |
| H13O | 11892.01 | 5891.36 | 4423.53 | 102 |
| H13P | 8344.7 | 3771.11 | 3894.27 | 91 |
| H13Q | 8819.85 | 4021.67 | 4381.78 | 91 |
| H13R | 4265.11 | 4441.44 | 2134.53 | 113 |
| H13S | 4367.07 | 3777.14 | 2237.17 | 113 |
| H13T | 5174.36 | 4170.73 | 2298.69 | 113 |
| H13U | 7831.17 | 3067.5 | 4251.8 | 142 |
| H13V | 8297.47 | 3320.37 | 4742.25 | 142 |
| H13W | 8827.02 | 3082.09 | 4395.93 | 142 |
| H139 | 8094.38 | 6091.97 | 4861.84 | 54 |
| H140 | 3063.08 | 8982.26 | 4593.06 | 73 |
| H14A | 6305.83 | 8726.67 | 4669.45 | 118 |
| H14B | 7177.93 | 8809.19 | 4529.6 | 118 |
| H14C | 6763.83 | 8194.19 | 4506.04 | 118 |
| H142 | 9514.68 | 5386.42 | 5103.72 | 79 |
| H143 | 4388.05 | 9762.94 | 4643.86 | 60 |

Table 8 Atomic Occupancy for 1b.

| Atom | *Occupancy* |  | Atom | *Occupancy* |  | Atom | *Occupancy* |
| --- | --- | --- | --- | --- | --- | --- | --- |
| H67C | 0.356(10) |  | H67D | 0.356(10) |  | H67A | 0.644(10) |
| H67B | 0.644(10) |  | C69 | 0.644(10) |  | H69A | 0.644(10) |
| H69B | 0.644(10) |  | H69C | 0.644(10) |  | Cl1 | 0.502(13) |
| Cl3 | 0.502(13) |  | C139 | 0.502(13) |  | H139 | 0.502(13) |
| Cl4 | 0.584(13) |  | Cl5 | 0.584(13) |  | Cl6 | 0.584(13) |
| C140 | 0.584(13) |  | H140 | 0.584(13) |  | C141 | 0.356(10) |
| H14A | 0.356(10) |  | H14B | 0.356(10) |  | H14C | 0.356(10) |
| Cl7 | 0.498(13) |  | Cl8 | 0.498(13) |  | C142 | 0.498(13) |
| H142 | 0.498(13) |  | Cl9 | 0.416(13) |  | Cl10 | 0.416(13) |
| C143 | 0.416(13) |  | H143 | 0.416(13) |  | Cl11 | 0.416(13) |

Experimental

Single crystals of C70H61Cl3Se3
[1b]
were
[].
A suitable crystal was selected and
[]
on a
Bruker APEX-II CCD
diffractometer. The crystal was kept at 153.15 K during data collection.
Using Olex2 [1], the structure was solved with the
Unknown
[2] structure solution program using
Unknown
and refined with the
Unknown
[3] refinement package using
Unknown
minimisation.

1. Dolomanov, O.V., Bourhis, L.J., Gildea, R.J, Howard, J.A.K. & Puschmann, H.
   (2009), J. Appl. Cryst. 42, 339-341.

Crystal structure determination of
[1b]

**Crystal Data**
for C70H61Cl3Se3 (*M*=1245.41 g/mol):
monoclinic, space group P21/c (no. 14),
*a* = 16.5069(9) Å, *b* = 23.6255(13) Å, *c* = 29.7702(15) Å, *β* = 103.380(2)°,
*V*= 11294.8(10) Å3,
*Z* = 8,
*T* = 153.15 K,
μ(CuKα) = 4.006 mm-1,
*Dcalc* = 1.465 g/cm3,
189744 reflections measured (4.826° ≤ 2Θ ≤ 136.61°),
20658 unique (*R*int = 0.0531, Rsigma = 0.0253) which were used in all calculations.
The final *R*1 was 0.0363
(I > 2σ(I)) and *wR*2 was 0.0979 (all data).

Refinement model description

Number of restraints - 13,
number of constraints - unknown.

Details:

```
1. Restrained distances
```

This report has been created with Olex2, compiled on
2022.04.07 svn.rca3783a0 for OlexSys. Please
let us know
if there are any errors or if you would like to have additional features.
